# Mantle Modularity Underlies the Plasticity of the Molluscan Shell: Supporting Data From *Cepaea nemoralis*

**DOI:** 10.3389/fgene.2021.622400

**Published:** 2021-02-05

**Authors:** Daniel J. Jackson

**Affiliations:** Department of Geobiology, Georg-August University of Göttingen, Göttingen, Germany

**Keywords:** biomineralization, plasticity, modularity, *Cepaea nemoralis*, shell formation, evolution, mantle, mollusc

## Abstract

Molluscs have evolved the capacity to fabricate a wide variety of shells over their 540+ million-year history. While modern sequencing and proteomic technologies continue to expand the catalog of molluscan shell-forming proteins, a complete functional understanding of how any mollusc constructs its shell remains an ambitious goal. This lack of understanding also constrains our understanding of how evolution has generated a plethora of molluscan shell morphologies. Taking advantage of a previous expression atlas for shell-forming genes in *Lymnaea stagnalis*, I have characterized the spatial expression patterns of seven shell-forming genes in the terrestrial gastropod *Cepaea nemoralis*, with the aim of comparing and contrasting their expression patterns between the two species. Four of these genes were selected from a previous proteomic screen of the *C. nemoralis* shell, two were targeted by bioinformatics criteria designed to identify likely shell-forming gene products, and the final one was a clear homolog of a peroxidase sequence in the *L. stagnalis* dataset. While the spatial expression patterns of all seven *C. nemoralis* genes could be recognized as falling into distinct zones within the mantle tissue similar to those established in *L. stagnalis*, some zones have apparently been modified. These similarities and differences hint at a modularity to the molluscan mantle that may provide a mechanistic explanation as to how evolution has efficiently generated a diversity of molluscan shells.

## Introduction

Animals fabricate a spectacular variety of biomineralized structures that serve almost all conceivable biological functions. From predation (Dietl and Vega, 2008), defense (Edgell et al., 2008), reproduction (Lodi and Koene, 2016) and vision (Aizenberg and Hendler, 2004), to navigation (Söllner et al., 2003), locomotion (Wilkinson, 2008) and buoyancy control (Greenwald and Ward, 2010), the evolution of the ability to precisely control the assembly of mineralized structures was a milestone in the rise of complex life (Murdock, 2020). From a molecular and cellular perspective, a complete understanding of the biomineralization process in any animal model remains elusive. Related to this incomplete functional understanding is a dearth of knowledge regarding the way in which evolution modifies the mechanisms of biomineralization to generate structures that fulfill different biological requirements. This is perhaps exemplified no better than within the phylum Mollusca. Shelled molluscs, and in particular gastropods, have evolved an impressive diversity of shells over the last 540+ million years. The evolutionary plasticity of the shell is likely one of the reasons molluscs have diversified so extensively, allowing them to occupy almost every ecological niche on the planet. Despite this, and the long-standing scientific and cultural fascination we have for molluscan shells (Sakalauskaite et al., 2019; Marin, 2020), a plausible and widely accepted hypothesis that can explain how evolution has generated this shelled diversity remains elusive.

Molluscs employ a variety of proteins (and other important biomolecules such as polysaccharides and lipids) to construct (primarily) calcified shells. Despite their paucity in the mature biomineral (often < 5% w/w), these biomolecules significantly influence many features of the shell including, but not restricted to, the crystallography (for example whether aragonite or calcite is deposited; Arroyo-Loranca et al., 2020), the mechanical properties (increased fracture resistance; Meyers et al., 2009) and pigmentation (Williams, 2017). The importance of these molecules has seen many proteomic, transcriptomic and genomic screens of conchiferans (shelled molluscs) aimed at the identification and comparison of their shell-forming protein repertoires (Joubert et al., 2010; Berland et al., 2011; Marie et al., 2011; Sleight et al., 2016; Yarra et al., 2016; Wang et al., 2017; Le Luyer et al., 2019; Malachowicz and Wenne, 2019; Xu et al., 2019). To this end, we previously surveyed and characterized the shell-forming proteome of the freshwater gastropod *Lymnaea stagnalis* (Herlitze et al., 2018). In that work we were able to spatially map the expression patterns of more than 30 shell-forming genes in developmental stages and in the adult shell-forming mantle tissue. This allowed us to recognize a modularity to the adult mantle tissue of *L. stagnalis*. We hypothesized that this modularity may be a key feature of all molluscan mantle tissues that would allow for the efficient modification and evolution of distinct regions within the mantle tissue to generate shells with novel features; for example increasing the thickness of the nacreous layer independently of the outer pigmented periostracum, or to modify the crystallographic orientation of nacre tablets independently of the prismatic layer. To further explore this idea of a modular organization of the shell-forming mantle tissue I have characterized the spatial expression patterns of seven major shell-forming genes in the terrestrial gastropod *Cepaea nemoralis*, a representative of a clade of molluscs that have received relatively little attention in terms of the molecular biology of biomineralization. These seven genes include a set of four previously identified shell-forming genes (Mann and Jackson, 2014), and three additional typical shell-forming genes. By comparing their spatial expression patterns with our previous results for *L. stagnalis* (Herlitze et al., 2018). I observe both striking similarities and differences. These observations provide further support for the notion that the molluscan mantle tissue can be subdivided into morphological modules (Eble et al., 2005; Esteve-Altava, 2016, 2017). This hypothetical framework provides a platform from which testable hypothesis of molluscan shell evolution can be built and tested.

## Materials and Methods

### Animals and *in situ* Hybridization Preparation

Juvenile *C. nemoralis* (recognized by the absence of the terminal pigmented lateral stripe in the shell) were collected from the surrounds of Göttingen in the spring of 2020. Juveniles were collected as they were assumed to be relatively rapidly depositing shell material and therefore to be expressing shell-forming genes. Total RNA was extracted from the mantle tissue of several individuals using Qiazol (Qiagen #79306) as a Trizol substitute. RNA integrity was observed via denaturing gel electrophoresis and quantified using a Nanodrop spectrophotometer. Complementary DNA (cDNA) was synthesized by first combining 1 μg of total RNA with 5 μL of 10 μM oligodT primer in a 10 μL volume and heating to 70°C for 10 min. To this mixture 5 μL of MMLV-RT buffer, 1 μL of 10 mM dNTPs, 8 μL of nuclease-free water and 1 μL Promega’s MMLV-RT H**^–^** point mutant (#M3682) was added, mixed and then incubated at 42°C for 90 min. This cDNA was used as template DNA in PCRs with primers designed to amplify 4 shell-forming genes previously identified in Mann and Jackson (2014), and 2 Glycine-rich shell forming genes, similar to the Shematrin gene family known to play a role in shell formation in oysters (Yano et al., 2006) and also an “animal heme dependent peroxidase” gene product that is a likely ortholog to *Lstag-sfc-5* that we previously studied in *L. stagnalis* (Herlitze et al., 2018). Details of the primers used to amplify these genes and PCR amplicon lengths are provided in Supplementary File 1. PCR products were cloned and confirmed by Sanger sequencing using procedures described in Herlitze et al. (2018). For *in situ* hybridization (ISH) a range of size classes (approximately 10–15 mm shell length) were studied to minimize the potential influence of age-specific gene expression patterns. Prior to fixation for ISH the shells of juvenile snails were gently cracked to allow for a more complete and rapid penetration of the fixative. Juvenile snails were fixed in 3.7% formaldehyde in PBSTw (1× PBS buffer with 0.1% Tween20) for 1 h at room temperature. After 30 min the fixative solution was renewed. Fixed snails were subsequently washed several times with PBSTw, and then dehydrated through an increasing EtOH series. Animals were given three washes in 100% EtOH and stored at −20°C. ISH was performed on at least 10 individuals for each gene.

### Paraffin Embedding, Sectioning, ISH, and Histology

Tissue preparation and ISH was broadly performed as described in Herlitze et al. (2018). Individuals selected for ISH were brought to room temperature and the shell was gently removed with a scalpel and tweezers while submerged in 100% ethanol. Once de-shelled each individual was cut sagittally using a razor blade such that two approximately equal halves (a left and right side) were produced. These halves were then further dehydrated in 100% ethanol for 1 h at room temperature to ensure all remaining water was displaced, and then incubated in xylene at room temperature overnight with gentle rocking. The next day tissue pieces were given a rinse with fresh xylene and then placed into molten paraffin which was allowed to perfuse the tissue for 24 h. The opposing halves of several individuals were then arranged in an embedding cassette such that the left and right half would be located next to each other, and the paraffin was allowed to set. Sections (12 μm thick) were then taken and collected onto polysine slides (Roth #ET10.1) and allowed to dry at 37°C overnight. Sections were de-waxed with 3× 10-min washes in xylene, and then re-hydrated through a descending ethanol series. Slides were then installed into an Intavis (now CEM) InSituPro Vsi liquid handling robot. An outline of the steps performed by the InSituPro follows: All slides received 2× 5-min washes of PBSTw before being treated with 0.1 U/mL Proteinase-K (NEB #P8107) diluted in PBSTw for 10 min at room temperature. Proteinase-K digestion was stopped with 2× 5-min washes of 0.2% glycine in PBSTw and 2× 5-min washes of PBSTw. Reactive amino groups were acetylated first with 1× 5-min wash of 1% (v/v) triethanolamine (TEA) in PBS, then with 2× 5-min washes of 1% TEA + 0.3% acetic anhydride (AA). These solutions were subsequently washed out with 2× 5-min washes of PBSTw. Tissue sections were then brought into hybridization buffer (5× SSC; 5 mM EDTA; 50% formamide; 100 μg/mL heparin; 0.1% Tween; 100 μg/mL salmon sperm; 1× Denhardt’s) with 2× 5-min washes at room temperature, followed by an elevation in temperature to 50°C for 30 min. Riboprobes were then added and the slides were brought to 75°C for 20 min to allow the probe and target to denature, followed by an 18 h incubation at 50°C. Excess probe was washed out at 50°C with one wash each of 4× (4× SSC, 50% formamide, 0.1% Tween), 2× (2× SSC, 50% formamide, 0.1% Tween) and 1× (1× SSC, 50% formamide, 0.1% Tween) wash solutions. Slides were brought into 1× SSC + 0.1% Tween and to room temperature before being rinsed 2× with PBSTw. Non-specifically bound riboprobe was digested with a single wash of 0.2 μg/mL RNAse A (NEB #T3018) in PBSTw followed by 2× PBSTw washes. Slides were brought into maleic acid buffer (MAB = 0.1M maleic acid; 0.15M NaCl; pH 7.5) with a 10-min wash, and were then blocked in 2% block (Roche #11 096 176 001) dissolved in MAB for 1 h at room temperature. Anti-Digoxigenin-AP, Fab fragments (Roche #11093274910) diluted 1/5,000 in 2% block was then applied and incubated at room temperature for 8 h. Excess antibody was washed out with 15× 15 min washes of PBSTw before tissue sections were brought into alkaline phosphatase color development buffer (AP = 0.1M NaCl; 0.1M Tris; pH 9.5). Slides were then removed from the InSituPro and 200 μL of color development solution (AP + 50 mM MgCl2 + 450 μg/mL NBT + 175 μg/mL BCIP) was applied manually to each slide and monitored for color development. Once the signal intensity was deemed adequate, the color reaction was stopped with several washes in water. Slides were finally mounted in an aqueous resin (Roth #2848) and imaged with a Zeiss StereoV8 and Axio ImagerM2.

De-waxed paraffin sections of *L. stagnalis* were also prepared (as described above) and stained simultaneously with *C. nemoralis* sections using Giemsa (Roth #T862.1). Briefly, a working stock of Giemsa stain was prepared by taking 600 μL of stock solution into 50 mL of distilled water. Sections were stained overnight at room temperature, rinsed briefly in distilled water, differentiated with 0.5% aqueous acetic acid for less than 30 s, washed in tap water for 10 min and mounted in an aqueous medium with DAPI.

### Sequence Alignments and Phylogenetic Analysis

All peroxidase sequences with similarity to Lstag_sfc_5 (Herlitze et al., 2018) were extracted from both the *L. stagnalis* genome (submitted to NCBI) and a re-assembly of our previously reported *C. nemoralis* transcriptome (Mann and Jackson, 2014) using tBLASTn. *C. nemoralis* sequences with similarity to Lstag_sfc_22 (Herlitze et al., 2018) were identified using tBLASTn. The best match (Cnem_R27072766) was aligned to Lstag_sfc_22 using Seaview v. 4.7 with default parameters (Gouy et al., 2010) and the resulting alignment submitted to MView^1^. Other sequences with similarity to Cnem_R27072766 were identified from SwissProt, GenBank’s nr database and the *L. stagnalis* genome using tBLASTn. Protein sequences were aligned using Seaview (as above) and conserved regions were identified using Gblocks (Talavera and Castresana, 2007). See the Supplementary Material for both the complete and Gblock-ed peroxidase (Supplementary Files 2, 3) and chitin-binding periotrophin-A alignments (Supplementary Files 4, 5). Phylogenetic analyses were performed with MrBayes v. 3.2.7a (Ronquist et al., 2012) with the following parameters: lset rates = gamma; prset aamodelpr = mixed; mcmcp nruns = 4, ngen = 2,000,000, nchains = 4, savebrlens = yes temp = 0.2 stoprule = yes stopval = 0.005. This number of generations was adequate for the stop value to be reached and the convergence diagnostic (Potential Scale Reduction Factor) was 1.000 for both analyses.

## Results

### Sequence Features

Four of the seven genes investigated here were previously identified by proteomic work on shells of *C. nemoralis* (Mann and Jackson, 2014). In that previous work 59 gene products accounted for > 90% of all identifiable peptides in the shell of *C. nemoralis*. Here, four of these gene products (R27072837, R27072766, R27073283, and R27075188) which accounted for a total of almost 40% of the shell-protein content (Mann and Jackson, 2014), were cloned and studied further. The remaining three genes were selected from an assembly of *C. nemoralis* transcriptome data (Mann and Jackson, 2014) because they either had features indicative of a role in shell-formation with a distinctive expression pattern (glycine-rich-2 and -3 have unusually high glycine contents and are expressed in zone 3 of the *L. stagnalis* mantle) or provided a clear example of an ortholog to a shell-forming gene previously spatially characterized in the mantle tissue of *L. stagnalis* (Herlitze et al., 2018). All seven of the derived protein sequences possess a signal sequence and are therefore likely to be secreted from the mantle tissue (Supplementary Files 6–8).

#### R27072766 (Chitin Binding Periotrophin-A Domain)

**FIGURE 1:**
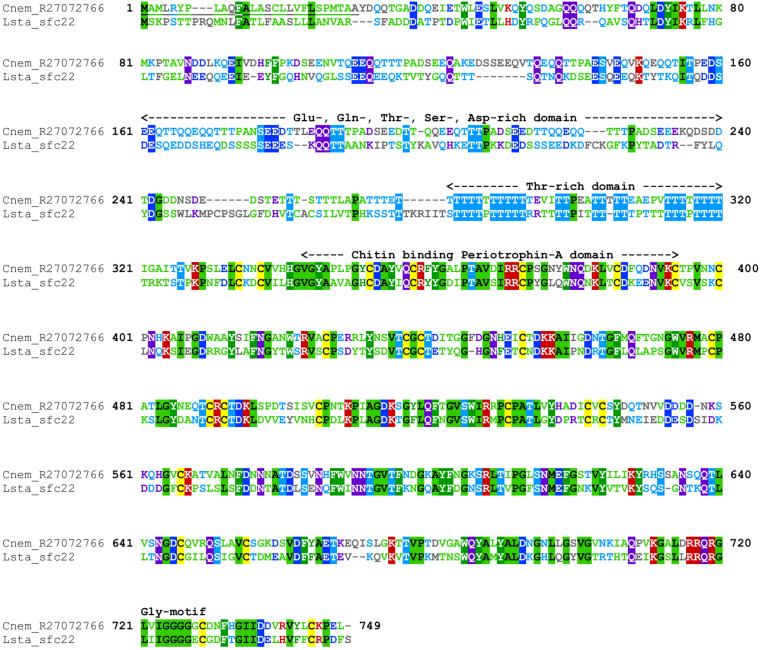
Annotated alignment of Cnem-R27072766 and Lstag-sfc_22. These orthologous shell-forming proteins (see Figure 2) each possess a signal sequence (underlined) and domains rich in Glu, Gln, Thr, Ser, and Asp. The locations of 24 Cys residues are conserved, and the chitin binding domain displays a high degree of sequence conservation. Cnem-R27072766 was identified from a proteomic screen of the *C. nemoralis* shell (Mann and Jackson, 2014) and Lstag_sfc_22 was identified from a proteomic screen of the *L. stagnalis* shell (Herlitze et al., 2018).

**FIGURE 2:**
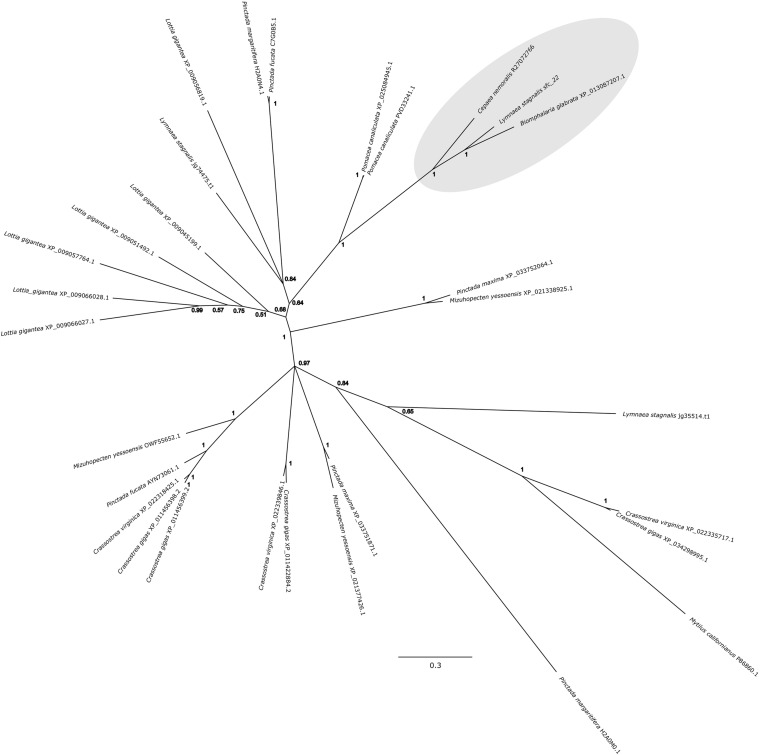
Bayesian phylogenetic analysis of homologous chitin binding Periotrophin-A sequences. The tree presented here is midpoint rooted and posterior probabilities for each node are indicated. The previously studied peroxidase sequence from *L. stagnalis* Lsta_sfc_22 (see Herlitze et al., 2018) and the *C. nemoralis* peroxidase sequence reported here, Cnem-R27072766, along with a sequence from *Biomphalaria glabrata* are highlighted in gray. See Supplementary Files 4, 5 for the aligned sequences used to generate this phylogeny.

*Cepaea nemoralis* contig R27072766 is 2,716 nucleotides long and encodes an open reading frame (ORF) of 727 amino acid residues. BLASTp searches against SwissProt returned sequences with significant similarity to several shell-associated proteins from bivalves implicated in the formation of nacre (Supplementary File 9) including Pif (Suzuki et al., 2009). A search for conserved domains revealed a clear chitin binding periotrophin-A domain (Figure 1 and Supplementary File 10). Searching the Cnem-R27072766 sequence against the *L. stagnalis* transcriptome reported in Herlitze et al. (2018) returned Lstag_sfc_22 as the top hit. A phylogenetic analysis of all of these sequences grouped the *C. nemoralis* R27072766 and *L. stagnalis* jg75923.t1 sequences (along with a *Biomphalaria glabrata*) sequence with strong support (Figure 2), indicating that our proteomic screen of the *C. nemoralis* shell (Mann and Jackson, 2014) identified the ortholog of Lstag_sfc_22, a protein identified by our proteomic screen of the *L. stagnalis* shell (Herlitze et al., 2018).

#### Peroxidase

**FIGURE 3:**
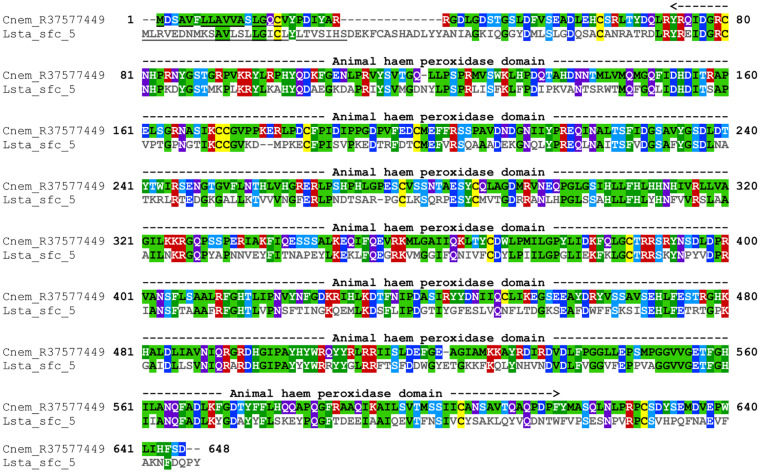
Annotated alignment of Cnem-R37577449 and Lsta_sfc_5. These orthologous shell-forming proteins (see Figure 4) each possess a signal sequence (underlined), and conserved “Animal haem peroxidase” domains (see Supplementary File 10). Cnem-R37577449 was identified from a proteomic screen of the *C. nemoralis* shell (Mann and Jackson, 2014) and Lstag_sfc_5 was identified from a proteomic screen of the *L. stagnalis* shell (Herlitze et al., 2018).

**FIGURE 4:**
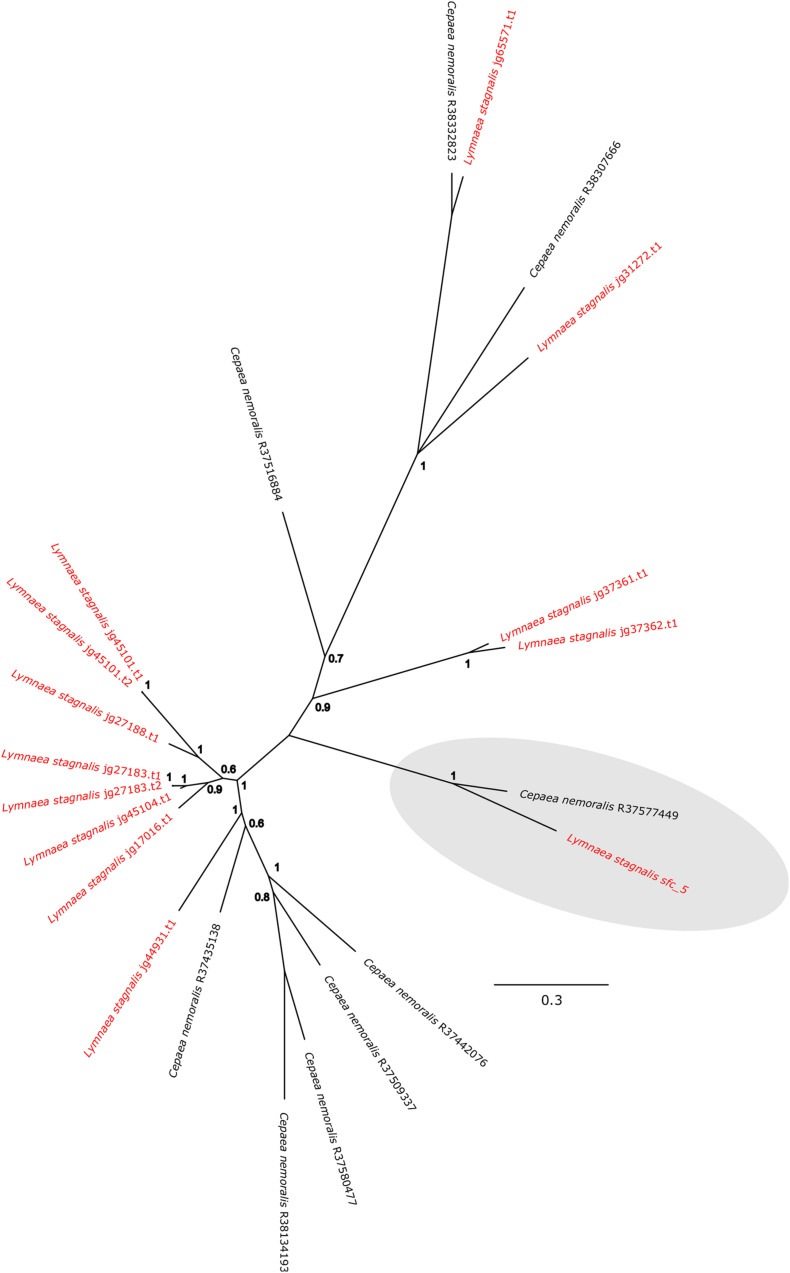
Bayesian phylogenetic analysis of *L. stagnalis* and *C. nemoralis* peroxidase sequences. The tree presented here is midpoint rooted and all *L. stagnalis* sequences are highlighted in red. Posterior probabilities for each node are indicated and the previously studied peroxidase sequence from *L. stagnalis* Lsta_sfc_5 (see Herlitze et al., 2018) and the *C. nemoralis* peroxidase sequence reported here Cnem-R37577449 are highlighted in gray. See Supplementary Files 2, 3 for the aligned sequences used to generate this phylogeny.

*Cepaea nemoralis* contig R37577449 is 2,200 nucleotides long, contains an “animal haem dependent peroxidase” domain (Figure 3 and Supplementary File 10) and shares sequence similarity with a diverse range of peroxidases from vertebrates and invertebrates (Supplementary File 9). There are a number of peroxidase-domain containing contigs in both the *C. nemoralis* transcriptome and the *L. stagnalis* genome (Supplementary File 11), however, a phylogenetic analysis revealed that *Cnem_*R37577449 was more closely related to *Lsta_jg27188.t1* [the gene model for the previously reported Lstag_sfc_5 in Herlitze et al. (2018)] than to any other sequence (Figure 4).

#### Glycine-Rich-2 and -3

**FIGURE 5:**
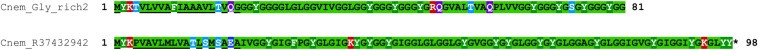
Sequence features of *C. nemoralis* glycine-rich proteins expressed in the mantle. These contigs were identified from a *C. nemoralis* mantle transcriptome after searching for translations that gave proteins with a detectable signal sequence and high glycine contents. For each sequence the signal sequence is underlined. Note that Cnem-Gly-rich 2 is apparently not full-length.

A screen of a previous *C. nemoralis* mantle transcriptome assembly (Mann and Jackson, 2014) for ORFs that possess a signal sequence and mature protein sequences with anomalous amino acid contents revealed several secreted glycine-rich contigs. Two of these were cloned and apparently possess glycine contents of >50% and high tyrosine contents (13.1 and 19.2%), however, it must be noted that *Cnem*-gly-rich-2 (339 nucleotides long) is apparently not full length as a stop codon could not be identified (Figure 5 and Supplementary Files 7, 8). This sequence was nonetheless selected for further characterization because of the extremely glycine-rich domain evident at the amino-terminus of the secreted protein.

#### Novel Genes R27072837, R27073283, and R27075188

Cnem-R27072837 (2,379 nucleotides), Cnem-R27073283 (1,878 nucleotides) and Cnem-R27075188 (1,827 nucleotides) encode proteins that were previously identified in a proteomic screen of the *C. nemoralis* shell (Mann and Jackson, 2014). None of the translated products of these genes possessed recognizable domains or shared similarity with sequences in the SwissProt database (however, these sequences did return hits against the nr database that were strictly gastropod, see Supplementary File 12). Cnem-R27072837 is notable as it was previously identified as being the most abundant recognizable protein in the shell of *C. nemoralis* (Mann and Jackson, 2014) accounting for more than 26% (by iBaq abundance) of all identifiable proteins. The mature (secreted) protein is also predicted to have unusually high glycine (12.4%) and proline (20.7%) contents (Supplementary Files 6, 8). Cnem-R27072837 and Cnem-R27075188 are also likely orthologs to proteins we previously identified in a proteomic screen of the *L. stagnalis* shell [Lsta_sfc_27 and Lsta_sfc_20, respectively, see Supplementary Files 13, 14; (Herlitze et al., 2018)]. While Cnem-R27073283 has a likely ortholog in the *L. stagnalis* genome (jg37438.t1) we have not yet studied the expression pattern of that gene in *L. stagnalis*.

### Comparative Histology

**FIGURE 6:**
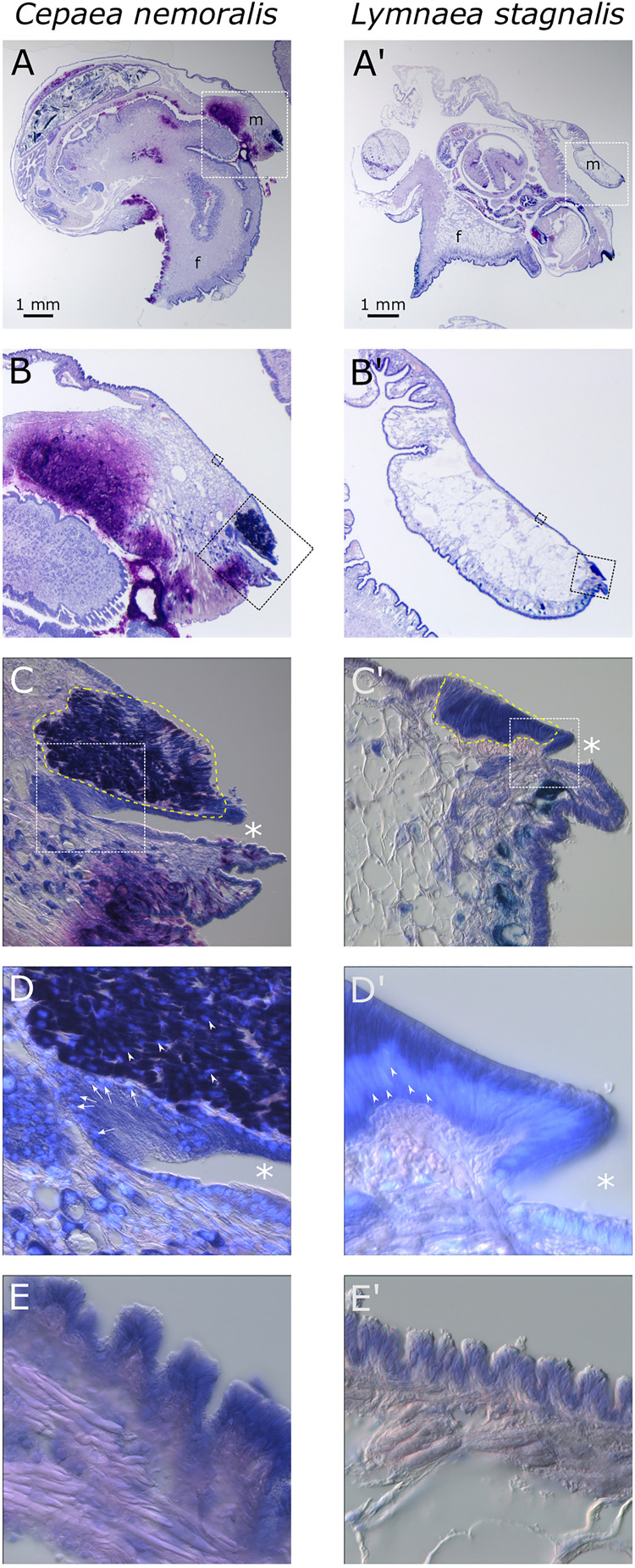
Comparison of *C. nemoralis* and *L. stagnalis* mantle morphologies. Giemsa stained paraffin sections of juvenile snails of each species reveal similarities and differences in some of the main feature so the mantle tissue. **(A,A’)** An overview of representative sections of each species and the location of the mantle tissue in relation to the rest of the body. The white dashed boxes indicate the magnified region shown in **(B,B’)**. **(B,B’)** The edge of the mantle tissue where new shell material is deposited. The black dashed boxes indicate the magnified region shown in **(C,C’,E,E’)**. **(C,C’)** The mantle edge and the periostracal groove (indicated by asterisks) contains the distinctive “belt” region (yellow dashed outline). The white dashed boxes indicate the magnified regions shown in **(D,D’)**. **(D,D’)** Magnified view of the periostracal groove (indicated by asterisks) and the belt. In *C. nemoralis* a histologically distinct population of cells at the base of the periostracal groove possess basal nuclei (stained sky blue with DAPI and indicated with white arrows). Nuclei in the belt in *C. nemoralis* are not basally oriented (white arrow heads in **D**). In *L. stagnalis* nuclei in belt cells are clearly basally oriented (white arrow heads in **D’**) and the cells themselves are distinctly columnar. **(E,E’)** Proximal regions of the mantle epithelia in zone 5 appear broadly similar between the two species.

Giemsa stained paraffin sections of *C. nemoralis* and *L. stagnalis* tissue sections revealed broad similarities and subtle differences in the arrangement of cells within the mantle tissue of each species (Figure 6). While zone 5 of the mantle (the proximal, squamous epithelium that covers most of the animal) appears to be largely similar between the two species (Figures 6E,E’), the distal-most leading edge of the mantle that is comprised of zones 1–4 and is responsible for the growth of the shell at its very edge, revealed clear differences. Perhaps the most noticeable difference was in the morphology of the belt (zones 2 and 3; Figures 6C,D’). In *C. nemoralis* the darkly stained belt appears to be comprised of cells that are not oriented in any appreciable way. The nuclei of these cells are not located basally, and the cells themselves do not have a classic columnar morphology (Figure 6D). In contrast, the belt of *L. stagnalis* is comprised of tall columnar cells with the nucleus clearly basal to the cell (Figure 6D’). In addition, in *C. nemoralis* at the base of the periostracal groove (which is responsible for the secretion of the periostracum) there is a population of cells with basally located nuclei that is not apparent in *L. stagnalis* (*cf.* Figures 6D,D’).

### ISH

**FIGURE 7:**
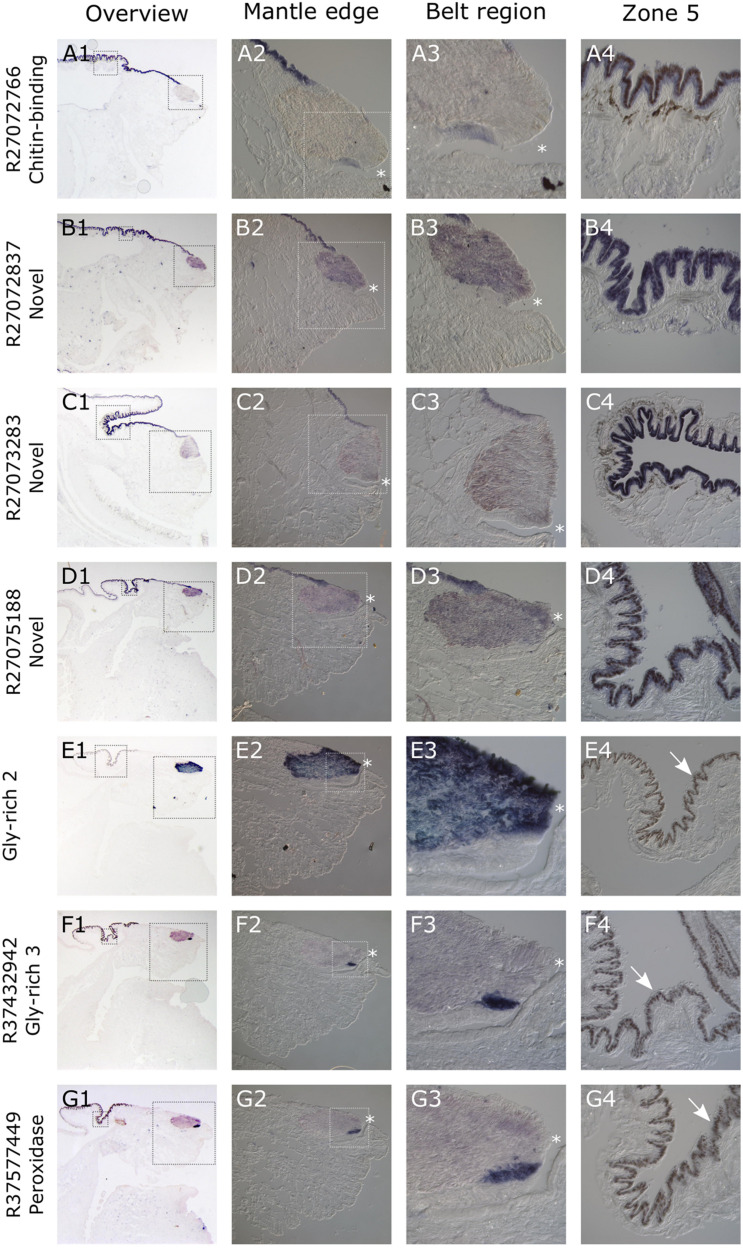
Overview of *C. nemoralis* and *L. stagnalis* mantle tissue and ISH of seven shell-forming genes in *C. nemoralis* juvenile mantle tissue sections. The first image in each row provides an overview of the staining pattern for each gene, with dashed boxes indicating regions that are magnified in columns 2 and 4. The dashed box regions in all column 2 panels are magnified in column 3. For Cnem-glycine-rich-2 and -3 and Cnem_R37577449, the lack of expression in zone 5 reveals naturally brown pigmented mantle epithelium (white arrows in E4–G4). For all panels a dark blue color can be interpreted as the result of alkaline phosphatase activity (i.e., ISH signal). The opening of the periostracal groove is indicated by a white asterisk in columns 2 and 3.

All seven of the genes studied here gave consistent, clear and distinct expression patterns in the mantle tissue of all of the *C. nemoralis* individuals investigated. The four genes that were previously identified by a proteomic screen of the *C. nemoralis* shell (updated contig names R27072837, R27072766, R27073283, R27075188) are all within the top 8 most abundant proteins identified within the *C. nemoralis* shell, with contig R27072837 as the most abundant accounting for more than 25% of the identifiable protein content of the shell (Mann and Jackson, 2014). All four of these genes are expressed exclusively in zone 5 of the mantle (Figures 7A1–D4). Cnem-R27072766, which is the ortholog of Lstag_sfc_22 (Herlitze et al., 2018) and possesses a chitin binding Periotrophin-A domain, appears to have a homologous expression pattern to *Lstag_sfc_22* in zone 5. The three genes bioinformatically targeted for characterization due to their likely role in molluscan shell-formation, (glycine-rich 2 and 3) and Cnem_R37577449 (peroxidase) due to its orthology with Lstag_sfc5 (Herlitze et al., 2018), were all expressed within zones 1–4 (Figures 7D1–G4). Glycine-rich 2 was broadly expressed throughout the “belt” region (zones 2–4) while glycine-rich 3 and the peroxidase homolog were restricted to zone 1 and appear to be spatially co-expressed (Figures 7F1–G4).

## Discussion

The catalog of proteins involved in molluscan shell-formation continues to grow at an exponential rate, and many exciting discoveries continue to be made based on high-throughput sequence analyses of the shell itself, the mantle tissue and the genomes of various molluscs (Zhang et al., 2012; Kocot et al., 2016; McDougall et al., 2016; Aguilera et al., 2017; Der Sarkissian et al., 2020; Marin, 2020). Due to the general lack of *in vivo* gene manipulation assays for most molluscan models, additional insight into the functions of these genes (many of which share little to no sequence similarity with non-molluscan species) can be gained by characterizing their spatial expression patterns (Nederbragt et al., 2002; Jackson et al., 2006; Grande and Patel, 2008; Samadi and Steiner, 2009). With this approach we previously characterized the expression patterns of 31 genes identified from a proteomic screen of the *L. stagnalis* shell (Herlitze et al., 2018). In that previous work, coupled with a previous histological analysis of *L. stagnalis* mantle tissue (Timmermans, 1969), we were able to categorize the spatial expression patterns of those 31 genes into five distinct domains (Herlitze et al., 2018). The striking modularity of those expression domains led us to hypothesize that this may be a general feature of the molluscan mantle that facilitates the evolution of new shell morphologies. The expression patterns of the seven *C. nemoralis* genes I investigated here (four of which are orthologs to *L. stagnalis* shell-forming genes) provides an opportunity to explore this hypothesis further. While *L. stagnalis* and *C. nemoralis* are both pulmonates, as respective representatives of the families Lymnaeidae and Helicidae they share an ancestor that lived ∼200 million years ago (Teasdale, 2017) placing this comparison in context.

As a first step toward characterizing the architecture of the *C. nemoralis* mantle on a molecular level, I cloned four genes that give rise to some of the most abundant proteins we previously detected in the *C. nemoralis* shell (Mann and Jackson, 2014). Three of these genes (Cnem_R27072837, Cnem_R27073283, and Cnem_R27075188) have no recognizable domains and share no sequence similarity with SwissProt sequences, while Cnem_R27072766 contains a chitin binding Periotrophin-A domain, shares sequence similarity with other molluscan shell-forming proteins and is the ortholog of Lsta_sfc_22 (Figure 2 and Supplementary File 9; Herlitze et al., 2018). Interestingly all four of these abundant genes were expressed in zone 5 of the *C. nemoralis* mantle (Figure 7), as was *Lsta_sfc_22* in *L. stagnalis* (Herlitze et al., 2018). While similar molluscan shell-forming genes (notably Pif from *Pinctada fucata*) have been associated with the production of nacre (Suzuki et al., 2009), neither *L. stagnalis* nor *C. nemoralis* construct nacre and so the functions of Cnem_R27072766 and Lsta_sfc_22 remain unknown. Nonetheless it is a striking reminder that oysters and pulmonates do share such similar downstream effector genes in their biomineralization toolkits, along with other proteins such as carbonic anhydrases, tyrosinases and peroxidases (Zhang et al., 2006; Hohagen and Jackson, 2013; Le Roy et al., 2014; Liu et al., 2014; Herlitze et al., 2018). The similarity in the spatial expression patterns of *Lsta_sfc_22* and *Cnem_R27072766* (both within zone 5), and their clear orthology (Figure 2), also supports the overall approach of comparing two mantle tissues separated by ∼200 million years of evolution (Teasdale, 2017). In addition, two of these 4 genes I selected from our previous proteomic analysis of the *C. nemoralis* shell (Cnem_R27072837 and Cnem_R27075188) also appear to have orthologs in the set of *L. stagnalis* shell-forming proteins we previously identified (Lsta_sfc_27 and Lsta_sfc_20, respectively; Herlitze et al., 2018, #91913). Cnem_R27072837 was the most abundant protein we could identify in the shell of *C. nemoralis* (Mann and Jackson, 2014) and it is expressed exclusively in zone 5 (Figure 7). This protein has high and exclusive sequence similarity with Lsta_sfc_27 (Supplementary File 13; Herlitze et al., 2018) and is therefore likely to be the ortholog of this protein. Lsta_sfc_27 is also exclusively expressed in zone 5 (Herlitze et al., 2018). In contrast, Cnem_R27075188 which also has a very high sequence similarity with Lsta_sfc_20 (Supplementary File 14) is expressed exclusively in zone 5 (Figure 7), while *Lsta_sfc_20* is expressed in zone 4 (Herlitze et al., 2018), a subtle but noticeable difference. BLASTp searches against SwissProt revealed no similar sequences to Cnem_R27075188 (Supplementary File 9), while searches against nr only returned gastropod sequences (Supplementary File 12) suggesting that this is a lineage restricted gene.

Secreted, glycine-rich proteins are typical members of molluscan shell-forming proteomes (Yano et al., 2006; Herlitze et al., 2018) and may provide similar mechanical properties to the shell as silk proteins do for spider silk (McDougall et al., 2016). In addition, certain shell-forming proteins are known to possess distinct domains rich in glycine (for example Lustrin (Shen et al., 1997), and Nacrein (Miyamoto et al., 1996)]. As we previously reported the spatial expression patterns for several of these genes in *L. stagnalis* (Herlitze et al., 2018), all of which were restricted to zone 3, I was interested to identify potential orthologous glycine-rich shell-forming genes in *C. nemoralis*. I searched the *C. nemoralis* mantle transcriptome and was able to identify several secreted glycine-rich genes expressed in the mantle tissue. I then cloned and determined the spatial expression patterns for two of these. In *L. stagnalis* the “belt” zone (a narrow zone of high columnar cells that is continuous with a low columnar epithelium, which covers the remaining outer surface of the mantle as described by Timmermans (1969) encompasses zones 2 and 3. All three of the glycine-rich genes we previously studied in *L. stagnalis* were exclusively expressed in zone 3 of the belt (Herlitze et al., 2018). While assigning homology to regions of the mantle between species should currently be done with caution, if we assume these high columnar cells in the anterior region of the mantles of *C. nemoralis* and *L. stagnalis* are homologous “belts,” then there are significant differences in the expression of the two glycine-rich genes I studied here: *Cnem_Gly-rich-2* was exclusively expressed throughout the belt (zones 2 and 3), while *Cnem_Gly-rich-3* (Cnem_R37432942) was exclusively expressed outside of the belt in the periostracal groove in zone 1 (Figure 7). The remaining problem in this comparison of glycine-rich genes between *C. nemoralis* and *L. stagnalis* is the question of homology between the genes themselves. Sequences such as these that are so biased in composition cannot be confidently homologized without additional information, for example gene synteny (Vakirlis et al., 2020) that would require at least a draft quality genome assembly for each species. Nonetheless, one might provisionally assume that such extreme glycine-rich proteins may be serving similar functions in the shells of their respective species, and the observed differences in their spatial expression patterns would therefore impart observable differences to their shells. Due to these uncertainties I also studied another protein for which the question of homology was clear. Lsta_sfc_5 was identified in the shell of *L. stagnalis* and has a peroxidase domain (Herlitze et al., 2018). I searched the *C. nemoralis* transcriptome for similar sequences, and although I was able to identify more than a dozen peroxidase-like sequences (Supplementary File 11) Cnem_R37577449 was clearly the ortholog of Lsta_sfc_5 (Figure 4). In *L. stagnalis* mantle tissue *Lsta_sfc_5* is exclusively expressed in zones 1 and 2, a relatively broad expression domain that partly includes the belt (Herlitze et al., 2018). In contrast *Cnem_R37577449* is expressed in the mantle tissue of *C. nemoralis* in a relatively restricted pattern in zone 1 that is distal to the belt (Figure 7). This expression domain apparently overlaps that of *Cnem_Gly-rich-3* (Cnem_R37432942; Figure 7). While the precise functions of these enzymes in the mantle tissues of molluscs are not accurately known, these significant differences in spatial expression patterns could be expected to influence the overall structure of the mature biomineral. Specific gene function analyses are required to verify this hypothesis. In addition to a larger collection of shell-forming gene expression patterns from more diverse species, draft genome sequences would allow for deeper inspection of the loci that encode these genes, and would allow for the identification of orthologous *cis*-regulatory elements (CREs) that presumably drive shell-forming gene expression in zones 1–5. The identification of “mantle-zone-specific” CREs would lend strong support to the model of mantle modularity we previously proposed (Herlitze et al., 2018). In this regard a comprehensive study of such CREs across the Gastropoda (and beyond) would be a stimulating exercise.

**FIGURE 8:**
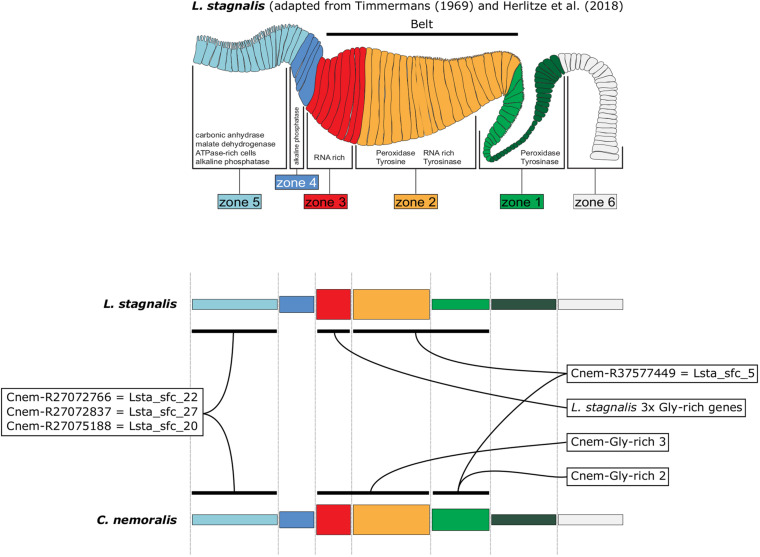
A schematic representation of *L. stagnalis* and *C. nemoralis* mantle tissues and orthologous shell-forming gene expression patterns highlights the modularity of the molluscan mantle. The uppermost panel is adapted from Herlitze et al. (2018) and indicates the main mantle zones that Timmermans described in her histological examination of *L. stagnalis* mantle tissue (Timmermans, 1969). Domains of enzymatic activity are shown and correlate well with cases where we were able to locate the gene expression of responsible genes (for example peroxidase activity was detected in zones 1 and 2 and this is where we observed the expression of Lsta_sfc_5, a peroxidase homolog). A more highly schematized representation of the mantle is presented in the lower panels, and facilitates the comparison of orthologous shell-forming genes. Note the differences in the expression of the peroxidase orthologs (*Cnem-R37577449* and *Lsta_sfc_5*) and the glycine-rich genes.

By characterizing the spatial expression patterns of the *C. nemoralis* genes I have studied here, and comparing them with those we previously studied in *L. stagnalis* (Herlitze et al., 2018), a conceptually appealing modularity to the molluscan mantle presents itself. When the expression patterns of orthologous shell-forming genes are compared on a highly schematized representation of the mantle (Figure 8) it appears as though the spatial regulation of certain genes have been significantly modified, most noticeably *Cnem-R37577449 cf. Lsta_sfc_5* (homologs of a peroxidase gene) and the glycine rich genes. Others (*Cnem-R27072766 cf. Lsta_sfc_22, Cnem-R27072837 cf. Lsta_sfc_27*, and *Cnem-R27075188 cf. Lsta_sfc_20*) appear to have been largely conserved over their ∼200 million years of independent evolution (Teasdale, 2017). While these intriguing observations require further investigation and an expansion of the comparative gene-expression datasets, I propose that this apparent modularity to the mantle tissue would have greatly facilitated the evolution of novel molluscan shell types. With the growing availability of conchiferan genomes, coupled with advanced sequencing methods such as single cell RNASeq and the development of gene-editing methods for more diverse species, it will be possible to rigorously test this hypothesis, and to gain further insight into the mechanisms by which evolution has generated the diversity of molluscan shells we admire today.

## Data Availability Statement

The datasets presented in this study can be found in online repositories. The names of the repository/repositories and accession number(s) can be found in the article/Supplementary Material.

## Author Contributions

DJ performed the lab work, sequence analyses, wrote and drafted the manuscript.

## Conflict of Interest

The author declares that the research was conducted in the absence of any commercial or financial relationships that could be construed as a potential conflict of interest. The handling editor declared a past co-authorship with one of the authors DJ.

## Footnotes

1 https://www.ebi.ac.uk/Tools/msa/mview/
