# Supplementary material for: Mantle Modularity Underlies the Plasticity of the Molluscan Shell: Supporting Data From Cepaea nemoralis
Source: Front Genet. 2021 Feb 5;12:622400. doi: 10.3389/fgene.2021.622400 (PMC7894901; doi:10.3389/fgene.2021.622400)
Supplement: Supplementary file 2 [file Data_Sheet_2.docx]

>Cnem_R37577449

------------------------------------------------------------

------------------------------------------------------------

------------------------------------------------------------

------------------------------------------------------------

------------------------------------------------------------

------------------------------------------------------------

------------------------------------------------------------

------------------------------------------------------------

------------------------------------------------------------

------------------------------------------------------------

----------------------------------------------------MDSAVFLL

AVVASLGQCVYPDIYAR-----------------R---GDLGDSTGSLD-----FVS---

----------EADLEHC--------SRLTYDQLRYRQIDGRCNHPRN--YGSTGRPVKRY

LRPHYQDKFGENLPRVYSVTGQ-LLPSPRMVSWKLH----------PDQTA-HDNNTMLV

MQMGQFIDHDITRAPELSGRNA-SIKCCGVPP---------KERLPDCFPIDIPPGDPVF

E------DCMEFFRSSP-----AVDNDGNIIYPREQINALTSFI----------------

--------DGSAVYGSDLDTYTWIRSENGTGVFLNTHL---VHGRERLPSHPHLGPESCV

SS------NTAESYCQLAGDMRVNEQPGLGSIHLLFHLHHNHIVRLLVAGILKKRGQPSS

PERIAKFIQESSSALKEQIFQEVRKMLGAIIQKLTYCDWLPMILGPYLI-DKF-------

-QLG-CTRRSRYNSDLDPRVANSFLSAALRFGHTLIPNVYNFGDK---------------

---------------RIHLKDTFNIPDASI----RYYDNIIQCLIKE------GSEEA--

YDRYVSSAVSEHLFESTRGH-KHALDLIAVNIQRGRDHGIPAYHYWRQYYRLRRIISLDE

FGE---AGI---AMKKAY-----RDIRDVDLFPGGLLEPSMP--GGVVGETFGHILANQF

ADLKFGDTYFFLHQQAPQGFR------AAQIKAILSVTMSSIICANSA------VTQAQP

DPFYMASQLNLPRPCSDYSEMDV---------------EPWLIHFSD-------------

------------------------------------------------------------

------------------------------------------------------------

------------------------------------------------------------

------------------------------------------------------------

------------------------------------------------------------

------------------------------------------------------------

------------------------------------------------------------

------------------------------------------------------------

------------------------------------------------------------

------------------------------------------------------------

------------------------------------------------------------

------------------------------------------------------------

------------------------------------------------------------

------------------------------------------------------------

------------------------------------------------------------

------------------------------------------------

>Cnem_R37442076

------------------------------------------------------------

------------------------------------------------------------

------------------------------------------------------------

------------------------------------------------------------

------------------------------------------------------------

------------------------------------------------------------

------------------------------------------------------------

------------------------------------------------------------

------------------------------------------------------------

------------------------------------------------------------

--------------------------------------------------MAAYNCVM--

-LLMSVILMA---VCSG--QDTCKNGTNNTSQFYEMADNIEAASTGTVGEMSRRFKE---

----------ERSE-FCQ----------DAGLRMYRTFNGTCNHPKN--LGASFTIQDRF

LPPAYSDGIN--SPRMFGVDGL-PLPSARTVSSKIH----------VSKDRKASKFSIAL

MAWGQWVDHDIISTAVSTRPGK-RIQCCGPNG-----TCPPFMTNPNCFPIEIPSDDRVF

E-----GTCLEFVRSLA-----ATDDNGYQKFPRAQINTVTSFL----------------

--------DASTVYGSTNELAESIRDPDG--YLLR------MKTDGFPPEAQNA---SCV

KR------EERQDYCFHAGDFRVNQHPFIGAIHTLFLREHNRICRRFRL-----------

---------LYPDLSDEKIFQMGRKLVGAMNQHFTYNVFLPLIMGDE-A-HVW-------

-KLISDSERSHYNPGIDPRIAVAFGSAAFRFGHSTIPSYLPVGNA---------------

---------------QVQLRYLFNTNGYVM----DDFEGILAGIAGI-TNSPDHSIQS--

VDKYVEDDVSKFLFFNNK--TKRGLDLISINLQRARDHGVQPYYKWRAFCGLRPLTGFDD

VEALGPDVG---ELAKVY-----RDVRDIDLFTGMLHEPVKT---GIVGPTISCIVGMQF

KRLKHGDRFFFNNNEEYTRFT------DEQLAALLNVTLGDIMCQNSY------LEAVPE

NVFLTVSESNPLIPCTRLRQMGL---------------DLTLFDY---------------

------------------------------------------------------------

------------------------------------------------------------

------------------------------------------------------------

------------------------------------------------------------

------------------------------------------------------------

------------------------------------------------------------

------------------------------------------------------------

------------------------------------------------------------

------------------------------------------------------------

------------------------------------------------------------

------------------------------------------------------------

------------------------------------------------------------

------------------------------------------------------------

------------------------------------------------------------

------------------------------------------------------------

------------------------------------------------

>Cnem_R37435138

------------------------------------------------------------

------------------------------------------------------------

------------------------------------------------------------

------------------------------------------------------------

------------------------------------------------------------

------------------------------------------------------------

------------------------------------------------------------

------------------------------------------------------------

------------------------------------------------------------

-------------------------------------------------------MQQFG

PP----ILRSQQ-NASSNNV-QQTGFIAVPI-QHPPNIVGLEPIDNVGKV----------

----TEEALT---VAEPPPEPECCQNEPDTGVFYSVAEKHGAAVTGSVAGKGHVFSQ---

----------ERRD-WCP----------DFGKSKYRTFDGTCNHPRN--LGSSFTPVSRL

LPPVYDDGLN--SPRTKGEDGS-PLPSPRVVSSSIH----------PSESN-GEDFTHML

MQWGQWVDHDMTAIITASNRSN-PTRCCGPDG-----ALPGVNTNKDCFPIELPETEKNF

R-----GRCMEFVRSLA-----ATNATGQPLQPREQVNSLNAFI----------------

--------DGSQIYGATSERAALLREKDD--YMLR------TK-GDFPPESPDG---GCI

LR------PGSKDYCFITGDLRTHENPALTALHVIWMREHNRMARELHA-----------

---------LKPQADKEEIYQIIRKIVGALQQIFTLNHYLPIVLGKD-A-YRL-------

-RLVSKEGRTVYNRDTDPRIFNAFTAAAFRFGHSSIPSAYAVGDK---------------

---------------LVPMREMFNRPALVL----DDMDSLLRGLVGM-TKYKEKASQK--

VDRNFVEEITQHLFEPNSTLPGRGLDLVSLNIQRARDHGLPPYTKWRTFCGLRPVYGFND

TEALGPYSS---QLGKVY-----RSVNDIDLFTAIVHEPIDT---GLLGPTGRCLVGIQF

ARAKFGDRFIFDTDDYRIAFT------EEQLAAMRKMTLSHVICQNTK------IDSLPV

DVFRFSDPTNPVVTCAALRAESL---------------DLSLFN----------------

------------------------------------------------------------

------------------------------------------------------------

------------------------------------------------------------

------------------------------------------------------------

------------------------------------------------------------

------------------------------------------------------------

------------------------------------------------------------

------------------------------------------------------------

------------------------------------------------------------

------------------------------------------------------------

------------------------------------------------------------

------------------------------------------------------------

------------------------------------------------------------

------------------------------------------------------------

------------------------------------------------------------

------------------------------------------------

>Cnem_R37509337

------------------------------------------------------------

------------------------------------------------------------

------------------------------------------------------------

------------------------------------------------------------

------------------------------------------------------------

------------------------------------------------------------

------------------------------------------------------------

------------------------------------------------------------

------------------------------------------------------------

------------------------------------------------------------

--------------------------------------------------MASLKCLT--

-ILLLTTLLN---MSVGTDNQYCRNKDKNRDC---------------------ASSK---

----------DTCN-KCV----------SPSTIKYRTFDGTCNHPRN--LGAESDLFDNL

LPQEYDDGSN--TPRQRGVDSQ-PLTSARNLSLNIH----------KQKDV-SAAFPILM

MQWGQWLAHDFTATKT---AIG-TISCCGPNG-----GCPPFVTDRDCFPIQVPPSDPTF

A-----GICLNFVRSAA-----ANGSDCLPLRPRRQINTVTSFL----------------

--------DCSQIYGSTNDVAASLREQGG--FLLR------TKFGKFPPEANSS---VCT

KR------NNTNDYCFLAGDFRANQHTYLSAFHTLWLREHNYIATELHK-----------

---------LKPDMSAEDKFQTTRKIIIGMHQVFTYNTFLPIMLGED-A-KRW-------

-QLTSTRGRSFYNPSLSPRIASAFSVGAFRFGHSTTPSLFPIGNR---------------

---------------SVPLRLLFNRPGYVL----DEFDGIMAGMVGL-TYAKDGRVQT--

SDKFITDELSRFLFFNNV--TGRGFDLNSVDIQRGRDHGIPPYVKWRSFCGLRPLTGFND

TEALGPHAS---ELANYY-----RSIDDMDLFTGMLHEPITS---GLVGPTIACLMRIQF

HNTKYCDRFFFDNTEPNSGFT------NAQLASIRAVTLSQIICRHTS------LTSIQR

NVFKLASADNPLIPCSKLRSQGL---------------KLSLFVETSKEY----------

------------------------------------------------------------

------------------------------------------------------------

------------------------------------------------------------

------------------------------------------------------------

------------------------------------------------------------

------------------------------------------------------------

------------------------------------------------------------

------------------------------------------------------------

------------------------------------------------------------

------------------------------------------------------------

------------------------------------------------------------

------------------------------------------------------------

------------------------------------------------------------

------------------------------------------------------------

------------------------------------------------------------

------------------------------------------------

>Cnem_R37580477

------------------------------------------------------------

------------------------------------------------------------

------------------------------------------------------------

------------------------------------------------------------

------------------------------------------------------------

------------------------------------------------------------

------------------------------------------------------------

------------------------------------------------------------

------------------------------------------------------------

------------------------------------------------------------

--------------------------------------------------MMTRHALS--

-FLILLTTMG---SFCPTIY------GWGRRCPADITE--------------LALTS---

----------SLHT-DCNNIGSC--TRCKQTTNTYRTNDGTCNHKDN--KGSQFAEIDRL

LPPTYADGSS--VPRQHGVGGQ-DLPSARNLTVKLL----------TSKEA-FSGNPIIL

FQWGQWTSHDILSTSVNTG----GIQCCGQQG-----KRL---FVPDCFPIDVPPNDPTL

S-----GTCMNFVRTRA-----AKCCDNSTVKPRIQTNDVTTFL----------------

--------DSSNVYGSDDEVAQAVREPGG--YLLR------TKFGLFPPVS-NG---TCN

IP------DGSREYCFLTGDHRPNQHTYLSAIHTFWLREHNRIATELKK-----------

---------LKPCLHNETIFQTAKQIVNAVNQAVTYNEYLPTLLGDD-A-TRW-------

-KLTSTAGRSTYNPQVNPSLVTEFTTAAFRLGHSQAPSKFPIGNE---------------

---------------LVHLRYLYNRPGLVL----DHFEDIIAAMAGH-SSVANQGSQR--

VDKHFTEELTQFLLYNPK--TGRGGDLLSTNIQRGRDHGLPTYVQVRDFCCLPPITGLND

T-ALGPEGY---KLGQFY-----KSVKDIDLYTGMLHELADI---GLAGPTIRCLVGLQF

YRLKYGDKYFFDNDRP-SGFS------DEQLESIRSVTLGHVMCHHTN------LESIQR

NVFEFTSLTNKEETCSKLKREGV---------------NLKLFV----------------

------------------------------------------------------------

------------------------------------------------------------

------------------------------------------------------------

------------------------------------------------------------

------------------------------------------------------------

------------------------------------------------------------

------------------------------------------------------------

------------------------------------------------------------

------------------------------------------------------------

------------------------------------------------------------

------------------------------------------------------------

------------------------------------------------------------

------------------------------------------------------------

------------------------------------------------------------

------------------------------------------------------------

------------------------------------------------

>Cnem_R38134193

------------------------------------------------------------

------------------------------------------------------------

------------------------------------------------------------

------------------------------------------------------------

------------------------------------------------------------

------------------------------------------------------------

------------------------------------------------------------

------------------------------------------------------------

------------------------------------------------------------

------------------------------------------------------------

--------------------------------------------MTTKTKMLRRLSVL--

-IMLVIVQHS---SSANASL------TKERKCPYDITK--------------LAFTS---

----------ALQA-QCKQTKQ---KTCGCSYDKYRSIDGSCNHVLT-DSGAAGSVIDKL

LPPTYDDVWH--APRKVGVGGE-PLPSPRQISVEIL----------TDKHV-FSIYTLLL

FLFGQWTAHDMISTTTS-S----GRNCCGANG-----TNPTIVQPTNCFPYAVASNDPTF

P-----GTCVSFVRSQA-----ANCTNWCQIGFRVNTNEVTAFI----------------

--------DSSQIYGSSHEVEQSVREQGG--YLLR------TNRGNFLPES-NL---TCN

KP------NVTRDYCVLAGDNRCNQHSILTFFHTFSMRAHNRMATQLHE-----------

---------LNPKLSNETIYQTVKSIINAIYQSILYNEYLPIVLGYDSV-RRW-------

-NLLSTSGKSVYNPDLRPRMIQEAVTAAMRFGHSQIPGEIPVGNE---------------

---------------MIPLRYLFNNPGIVL----DHFEDLMDALIGR-SQLPNQGSRK--

VDQYFTEDVSRYLFFNNK--TGRGSDLPATNIQRGRDQGLATYSQVREICCLPPMTGFND

TKALGPEGY---KLEKHY-----KSVDDIDLYVGGLLEVPES---GLLGPTFNCLVGLQY

YTLKHSDRFFFDNDQ--RHFT------DEQVKEIRKVKLGEMICHQTN------LTSVQK

NVFEKPSSTNEDVSCSWLKKNGL---------------DLRLFAGL--------------

------------------------------------------------------------

------------------------------------------------------------

------------------------------------------------------------

------------------------------------------------------------

------------------------------------------------------------

------------------------------------------------------------

------------------------------------------------------------

------------------------------------------------------------

------------------------------------------------------------

------------------------------------------------------------

------------------------------------------------------------

------------------------------------------------------------

------------------------------------------------------------

------------------------------------------------------------

------------------------------------------------------------

------------------------------------------------

>Cnem_R37516884

MEISIFVSAFLVFVMCCLDTARASACPERCLCFRTHVRCMFMNLESIPNVLPDTTTLDLR

FNKISKIPRGSFPHLPHLHTLFLNNNEIQELEDGYFDGLPELRKLYLYKNKIQKIHPNAF

RSIPHLEELYLHHNELSKFPEHLFDGNSNLKKLRLDSNSLVCDCDMMWLVDMLKEKGPET

QAAVYCRYPSKFQGRSLMSMAKEDFHCAEPKITMRPKDVDVSFGNTVYFTCRAEGAPNPE

IMWFHNDNMINTNEEERYSILDDGTLMIEAAQDSDKGVYECMARNAVGMAKANKVELRYM

GDQEIPKFMETPSDITVIEGDDAQLVCHVMGNPRPDITWTLNSDPVSGNIRAQVLESGSL

VITNIR-----LSDAGTYECSASNSVKTISSSARVRVLVKPVIISAPADVSVVQGTQVNF

TCEARGDSQPVLTWTKDGEFLRNNGRYEVLKNGQMLKIHNAAASDQGKFTCKAENAAGST

TASGTLRIVENAAPSFSQSSDLISAAAGSDVTLHCIADAR----PDALYDWMRDGRVLQN

RNRISVQAGELKITNVHIHDAGRYD---CVAENSLGKATKAIFLQVQGANIGRIGDQFVS

-----------------NAIPQATRQVNTAVNTTLSHLFDPNREHT--------------

-VQDLLAAFRYPTPE-------ALDLARAEEIFEQTLEIIQRHVSEGHSYNLTGFETSYH

ELISPAHIHMIANMSGCLRRTHAADCSDTCFHRRYRTMDGTCNNLQNPTWGAANTAFNRL

LPAIYENGFNA-------PVGW-NRHKLYKGTHLPSARLVSSLVISTEHITSDETFTHML

MQWGQFIDHD-MDLSPQAISFA-----RFSDGRRCNETCE---NTNPCFPIAVPT-S---

DPRIRNHECLGFTRSSATCNTDSTSLFFNTVSPRQQLNSLTSFI----------------

--------DASNVYGSTDRMASTLRNLASNRGLLREGPTTATARRLLPFNEDTLEMPDCQ

IQPSKRH----I-PCFIAGDVRVNEQLALIAMHTLWMRQHNQMAVELNR-----------

-------I--NPHWDGNKIYHEARKIMGALFQHITYKHWLPKVLGPK-------------

-GMAMIGPYEGYKPDVNPSIVNEFAVAAMRFGHTLIQPMIFRLNET--------------

FQEIPEG--------HLSLHQVFFAPH-RI-IEEGGLDPLIRGLFAK-------AAKKKM

PGELLNSELTEKLFQLAN---AIGQDLASLNIQRGRDHGTQFYNDYRVLCGLPRARTFDD

LRSEIPHKDTRDKLQALY-----SHPDNIDLFVGGVSEYVVE--GAKVGPTFLCILSDQF

KRLRDGDRFWYENKKN-NIYT------QEEVDEIEATMFYDVINNVTLALRDTNLINVTR

EEIYFCSSSSDPKGCECNDPTVD---DNDVIEVCVPLQHYDYFTGSEVSFVITIVVIVLS

LPVTI----AIMMCMSWQRSHSLNASGE-GSK-----AKFEVGPNHFYATEWVGRSTHGF

LTSRDVKINLDDTRLKILVSNMQKQVVRMIDLRRRLTNEQEKPRASVTRSNDKGHKLMMV

GVPGEIDLVLHFASQNDRDSAFRQLQGFFNKHNWDFHEGPVMAENVMWREAVTVDQRKEL

LAKFFRSALAEISGEIPDTS------N-QKHMEEILATQLTRTEFADALGLQPQSLFVRN

MFLLVDSSGDGFISFDEFKAYFGILSSGNPEQKAEMFFKMFDTSRTGKITKEDYKKMIIV

LFEMNETDRSKEFNINEIVDKVFKQVGKDQQGFLTLQDFKSIVLSDTADVWKFAVLNLEV

AGETATLGYKK--SVKDRAKSFIEGYKTNLRPTSMINLKND-----VRLPSTLESAPKSK

FDEFVQKWSRYVNNHRQQIFWVTLYTLVTIGIFVERAYYYSFEREHAGLRRLAGYGVSVT

RGAASAQMFTYASLLVTMCRNTLTFFRETFLHRFIPFDNFHDMHIYVAFLAVLFSVIHVI

GHIINFFHICTQSSEDLNCYFREYFRPTDVLASFHYWTYQTITGLTGVALILVLVVIYVF

AMPYARRNVFKYFRTTHNLYIALYILMFLHGHHRLVQPPLWPWYFIGPVVLFVLDKLVSV

SRNKVLLPVHKARLLPSGVIQLVFKRPLTFDYQSGQWVRIACPKLGKGEYHPFTLTSAPH

EEHLSLHIRAVGPWTSNLRKLFDTNKLTTTK-IPKYQIYLDGPFGESHQDWYRYSVSVLI

GGGIGVTPFASILKDIANKTVQ--VKRLPVQKVYFIWVTRTQQSFEWMTDIIRETEAADV

HDFLDVQICITQLKEQFDFRTTMLYICERHFQKFA-GASMFTGLRAKTHFGRPKFNPFFQ

ALKVVHKNVGQIGVFSCGPPAMTNSVQKSCTEQNSFTGPSLIHHYENF

>Cnem_R38332823

------------------------------------------------------------

------------------------------------------------------------

------------------------------------------------------------

------------------------------------------------------------

------------------------------------------------------------

------------------------------------------------------------

------------------------------------------------------------

------------------------------------------------------------

------------------------------------------------------------

------------------------------------------------------------

------------------------------------------------------------

-------------------------------------------------MKVCHFV----

---------IAA--AIC----SACICGNSVKDIPVQRFDGWYNNPLNPSWGGVGRPLERN

ITPTYEDETYR-------PSGW-DRPNPRYISNELF----GELR-GRSGIPNKRNLTALF

AFFGQIIQHEILDTDD--------------------VTCP---VE--ILDVTVPRGDPHF

DPNSEGDKTMPYERSA-----YEKTSGQSPNNPRRQINWQS-------------------

-----SFIDGSFLYGKSLVRTEFLRDPHSGKLACED------KWGKFPLRND-VGLPYNS

FT----YSFNTLENLWRLGDPDVFENPAILALNMIFYRYHNAKAEEFEQ-----------

---------LHNYMSNEEVFDKTRRWVIGCIQKIIVYDWLPMLIEKE-------------

-----LPAYSGYKPWVKSDITDIFDAGAINYIMTLIPSAIHQIDSS---CQ---------

------------ERATDRLCNVFWKAMDVVYKHESGILEILRGLSAQ-------YAEN--

SDTFVVEDYRSKFYGPLY---HSKHDAVLLTIMKGRDYGLPDYNTVRVTMGLKEMTNFEE

VNPWLNQTNP--ELFAAFKSVHKGNLSTIDMFVGGMMESTPD--G--PGELFTHILYDQF

IRLRDGDRFWFENSAN-GLFS------SEEVQRLRNTTLADIFRTAG-IDM-Q---DIQD

NVFT------LTDKSPCGHRMIF---NDSTLEPCEKHHGYDYFHGSEIPYIIIWTCLGLL

PLLCILAAYILAKCKKWRHERRIRVLKAEQEKRRNFRRSLSCSVSVHDGVEWRGPHFSG-

---RPIELHLTTKGCLEIFS-ATGTHLRAIK-------ISSLSNIGVKISSNKKRNIMAL

ILPREYDLVVEFRTEVLRNEFVSMLCQFVQNHSVDITVIE-LKLRDLYNAATTKEKRNKM

LEKFFKTVFSEAFQMDYDPSLDSGQQDLKKHSQEILEIELSKEEFAEAMAVKPNSDFVEH

FFSLIDSDRNGYISFREFLNAVVLFSKGSIQDKLQTMFYMYDIDGTGYMNLMQIGQMFRS

LLELAQSDLT-HGDVDLLVHSLCKQSGVGPKEQYSFDDFCQLLSPQMDKLWNASFDW---

RGIPKVIPSKVKSPKKTGSVNSETDMRRRGSSASLSGID--GAKSSVVKKFEAVREQYTP

VKAKVKLVKHFIENYRHHIFFVVLFYGICLGLFAERFYYYTVEQESAGFRKLMSYGISMT

RGAAASMSFTFSLMLVTMCRNTITYLRSTPLNLFIPFDSHVSFHKVVAWTSLFFSAIHII

GYSFNFYHLATQPTRFL-CIFTSLIFRTEHSMSFQEWVFGTMTGFTGVLLVLILCILYVF

ATQTARRHIFSMFWMTHKLFIVLYILVIIHGASVVVQKPMFFAYLTGPAIWFMVDKLISL

SRKKTELCIIKAQNLPSDVTMIEFKRPPRFEYKSGQWVRIACLAQGKDEYHPFTLTSAPH

EDTLKLHIRALGPWTWNLRHIVDNVNIKNTKDYPK--LYLDGPYGAGQQDWYQYDVSVLV

GAGIGVTPYASILKDFVHMASINMRYKVKCQKLYFIWITGSQRHFEWLIDILREVEEVDT

QGMVSIDIFITQFFQNFDLRTALLYIFEEHFQKLNGGKSVFTGLKATTHFGRPQLNKIMG

AVHKAHPQARKVGVFSCGPPGVTKGVERACVDASKATKALFEHHFENF

>Cnem_R38307666

------------------------------------------------------------

------------------------------------------------------------

------------------------------------------------------------

------------------------------------------------------------

------------------------------------------------------------

------------------------------------------------------------

------------------------------------------------------------

------------------------------------------------------------

------------------------------------------------------------

------------------------------------------------------------

------------------------------------------------------------

-----------------------------------------MMIASSYSL----------

----------YLCLTICLTFY---AKESHGDNTEYAPVDGFRNNLGQPTLGIAGSNFARV

SPAAYSDGAYG-------MAGL-DRPHPMEISLKVH--------AGRSGQASTFSRNAMQ

AYFAQLVLDEVALVT---------------------PGCP---PE--YENMPVPDQHPLG

N---LTKRPQVYQRSL-----YDQSTGYVSGSPRQQVNMASSFL----------------

--------DGGIIYGTSKTWTSLVRSYSGGKLLADTDN----ITSSFPTYND-VGLPLLN

APIPREHVMKPADRLFRLGNAKGHENPFLLALQVIWFRWHNTIAGDIAE-----------

-------S-SAGNLTDEQIFQTAKKMVVAHFQKILVTDWLPAFLYGNQM----------N

ATMEVLPAYTKYDSRQHAGVTQEFL-AAMDFRHTFTPAAVWNLKAM---CKLGNTT----

---GRDNMGRSRQVKSLRLCNNFWDPQTAV---KDDLEMILRGMLYT-------LGKR--

EDGRFVSDFTEFFYGPFE---YSRTDHVAAQIQRYRDHGLSGLNTIRRSYGLATLSNWTS

LGGLN------GTLHRLY---GGTKPDNVDILTGGLLDQSGQ--MAGLSEIFRKILIDQF

TILRDGDRFWYQR--S-GLFT------ANEIKEIESVTFKKILLATTRLSE----NEIPD

NVFLCR----DTNNCKCLTPPDVDNIASTDIDVCSNLATHDYIEGSKLSFALSFLALALV

VPASY----LVLFCLVKKRKTVIQEVQKRKST-----FVSKRNQNKFMAREFLGKKSGF-

---RDIKGEFKADEGMIIICDGGGKILRYINIKGE---VENAPPINIHMSSDKDLSLLAI

KVPEDIDLILDFVDQGARQLFIQSLERYLSAVGLNFETER-MSESHLYSRCTDKDQRQNL

LDKFFRAVCI--------------------------------------------------

------------------------------------------------------------

------------------------------------------------------------

------------------------------------------------------------

------------------------------------------------------------

------------------------------------------------------------

------------------------------------------------------------

------------------------------------------------------------

------------------------------------------------------------

------------------------------------------------------------

------------------------------------------------------------

------------------------------------------------------------

------------------------------------------------

>Lsta_jg60788.t1

------------------------------------------------------------

------------------------------------------------------------

------------------------------------------------------------

------------------------------------------------------------

------------------------------------------------------------

------------------------------------------------------------

------------------------------------------------------------

------------------------------------------------------------

------------------------------------------------------------

------------------------------------------------------------

--------------------------------------------------MLRVEDNMKS

AVLSLLGICLYLTVSIHSDEKFCAS---HADLYYA---NIAGKIQGGYD-----MLS---

----------LGDQSAC--------ANRATRDLRYREIDGRCNHPKD--YGSTMKPLKRY

LKAHYQDAEGKDAPRIYSVMGDNYLPSPRLISFKLF----------PDIPK-VANTSRWT

MQFGQLIDHDITSAPVPTGPNG-TIKCCGVKD-----------MPKECFPISVPKEDTRF

D------TCMEFVRSQA-----AADEKGNQLYPREQLNAITSFV----------------

--------DGSAFYGSDLNATKRLRTEDGKGALLKTVV---VNGFERLPNDTSAR-PGCL

KS------QRPESYCMVTGDRRANLHPGLSSAHLLFHLYHNFVVRSLAAAILNKRGQPYA

PNNVEYFITNAPEYLKEKLFQEGRKVMGGIFQNIVFCDYLPIILGPGLI-EKF-------

-KLG-CTRRSKYNPYVDPRIANSFTAAAFRFGHTLVPNSFTINGK---------------

---------------QEMLKDSFLIPDGTI----YGFESLVQNFLTD------GKSEA--

FDWFFSKSISEHLFETRTGP-KGAIDLLSVNIQRARDHGIPAYYYWRRYYGLRRFTSFDD

WGY--ETGK---KFKQLY-----NHVNDVDLFVGGVFEPPVA--GGVVGETFGHIIANQF

ADLKYGDAYYFLSKEYPQGFTDGKGFTDEEIAAIQEVTFNSIVCYSAK------LQYVQD

NTWFVPSESNPVRPCSVHPQFNA---------------EVFAKNFDQ-PY----------

------------------------------------------------------------

------------------------------------------------------------

------------------------------------------------------------

------------------------------------------------------------

------------------------------------------------------------

------------------------------------------------------------

------------------------------------------------------------

------------------------------------------------------------

------------------------------------------------------------

------------------------------------------------------------

------------------------------------------------------------

------------------------------------------------------------

------------------------------------------------------------

------------------------------------------------------------

------------------------------------------------------------

------------------------------------------------

>Lsta_jg17016.t1

------------------------------------------------------------

------------------------------------------------------------

------------------------------------------------------------

-------------------MC-LESICLEPVCLKS-Y----------SFLVNLLGVNLLG

INL-LGVNLLGVN--------LFGVSLLEVIQ----FLVNLLGVN---LLGINLLGVKLF

GEMTIIQEIHIPLAAFPLSLIMSRDCCP---------------PPIPTTPDTIIFSSGGG

RISNYFKNLSGPRDSGANHCLAMICARNWTL---VCMCLMALAIQLDALTSHLSGSDN--

---------------------NGNGRGQGLV-----------------------------

NDFRTFEDERDAGTTFSDARSLIDAQDAGSAPIGVIELNGKENGKGNGFGKVNSGNNQ--

-GRGNSQ-GK-------GNNQGRGDQQDSASNRGNG------NSQGKGTNQGKKSSQDSG

--------KGKVKEHVK-----------------VDARNEQGTNHNNGRFLANIVHI---

--WWSLGTTT---SVAGSINGVC---RRQINGFYNLAQRSSAITTGSVRSVGLRFAQ---

----------DFRD-LCT----------DVALRKFRTTDGNCNHPKN--WGTSFKPVTRL

LPADYSDGSG--SPRKTSVSGS-PLPSPRAVSRYVH----------PARTD-LLARTIMI

MQWGQWLDHDVTGFPVASELDR-SIKCCGPNG-----TLPALNTDPNCFPITLPKDEDNF

L-----GKCMEFVRSIP-----ATDNNGCALQPREQVNSITSFI----------------

--------DASQVYGSTEETAAKVRNRKG--FLLK------TKNGDFLPENVKG---GCI

VR------PGSSDYCFLAGDFRVNEHPALAAMHTIWLRLHNKIASNLKT-----------

---------LRPADSDDDVFQLTRKIVGALQQVITYNEWLPIILGKQ-A-SEL-------

-KLVSRAGRTRRQAGVDPRILNEFSTAAMRFGHSLIPDVFPIGDR---------------

---------------RVPLRQLFSRPAEVI----DNFDKVVAGIAGV-GTPGNRNAQK--

IDRNFVTEITNHLFEPQSGPAGRGLDLVALNIQRARDHGIPPYKAYRAFCGLRPLTGFND

VEALGPNVA---QLARAY-----SSVDDIDLFTGLVHEPPVHGSNALVGPTLSCILGTQF

YNLKFGDRFFFDTDEQVIAFT------NDQLASLRNTTLAKVICATTK------IPELPN

NVFSFPTKGNPLVPCPELEASGL---------------HLSLFA----------------

------------------------------------------------------------

------------------------------------------------------------

------------------------------------------------------------

------------------------------------------------------------

------------------------------------------------------------

------------------------------------------------------------

------------------------------------------------------------

------------------------------------------------------------

------------------------------------------------------------

------------------------------------------------------------

------------------------------------------------------------

------------------------------------------------------------

------------------------------------------------------------

------------------------------------------------------------

------------------------------------------------------------

------------------------------------------------

>Lsta_jg27188.t1

------------------------------------------------------------

------------------------------------------------------------

------------------------------------------------------------

------------------------------------------------------------

------------------------------------------------------------

------------------------------------------------------------

------------------------------------------------------------

------------------------------------------------------------

------------------------------------------------------------

-----------------------------------------------------MFYYCVL

ALTLFALILGQDYDISLVAEEEEVYDINVLTQTDNADVVESPPSKGNGR-----------

--FSSLEAFQ---QAAPRPAEVC---LPSSGEFYNMPERLSAMSTGSISSSAFRLSQ---

----------DYRE-LCL----------DFSSKKFRTIDGSCNNLRN--WGTSFKPVSRI

LPARYQDGVG--SPRTKASDGT-PLPGARFVSLAVH----------PAVTD-LTSRPIIA

MQWGQWIDHDITGFPAATSPNGP-LVCCGPNN-----TSPPANTDPNCFPILLPASEEDF

V-----GTCMSFVRSNA-----ATDANGCQLKPRRQVNSVTPFL----------------

--------DASQVYGSTDAVAASVRDAGD--FLLK------TKNEKFLPENVNS---SCI

RR------PGTNDYCFLAGDFRVNQHPYLQSLHTVFLRDHNRIARKLRA-----------

---------LRPNDSNENIFQLSRKIIGALQQMITYNEWLPVILGQT-A-KKL-------

-GLVSKTGRTKFQSRVDPRILVEFSSAAMRFGHSLIPAEFPIGDR---------------

---------------RVPLRQLFNRPGDVL----DNLDDVTAGMVGV-GTPGKRNTQK--

VDRNFVVEITKHLFEPPTAP-KRGLDLVSFNIQRGRDHGIAPYTTYRALCGLRPVRGFKD

VKALGPNVA---DLAKIY-----KSVNDIDLFTGLVHEPVLPGSNGLVGPTLACILGTQF

YNLKFGDRFFFDTDDKSVGFT------DKQLQSLRKVTLAKVICANTD------ITALPK

DVFNFPSRSNPLIPCDRHEGLDL---------------SL--FA----------------

------------------------------------------------------------

------------------------------------------------------------

------------------------------------------------------------

------------------------------------------------------------

------------------------------------------------------------

------------------------------------------------------------

------------------------------------------------------------

------------------------------------------------------------

------------------------------------------------------------

------------------------------------------------------------

------------------------------------------------------------

------------------------------------------------------------

------------------------------------------------------------

------------------------------------------------------------

------------------------------------------------------------

------------------------------------------------

>Lsta_jg45104.t1

------------------------------------------------------------

------------------------------------------------------------

------------------------------------------------------------

------------------------------------------------------------

------------------------------------------------------------

------------------------------------------------------------

------------------------------------------------------------

------------------------------------------------------------

------------------------------------------------------------

------------------------------------------------------MSRAMS

--------TGSV--------------------------TDAGTVLR--QDFRDINDV---

----NYGLVL---TVDRFIQGFC---EVNKNGFFNLPEMSRAMSTGSVTDAGTVLRQ---

----------DFRD-LCT----------DVTSKKYRTIDGNCNHPKN--WGTSFKPLARM

LPAEYDDGVS--SPRVRGSLGT-PLPSPRAISRFVH----------PSTPD-LISRTIMI

MQWGQWLDHDLSVSPVASEVNH-IIQCCGPNG-----TLPRNPTDPNCFPIILPSDEENF

A-----GQCMEFVRSIP-----AFDANNCMLRPREQINVLNSFV----------------

--------DASQVYGSTDDLAARLRVEGD--FLMK------TKDEIFLPEDVAS---ACI

KR------PGTKDYCFLAGDIRVNEHASLGAMHTIWLRAHNKIAKQLRQ-----------

---------LRPKDSNEEIFQLTRKIIGALQQVITYNEWLPIILGKH-A-TTQ-------

-KLPSKTGRTQRLLSADPRILNEFSTAALRFGHSFIPDVFPIGDR---------------

---------------RVPLRQLFNRPAEVL----DNLDDLVAGVTGV---SGSKSAQK--

IDRNFVAEITNHLFEPPTGPRGHGLDLIALNIQRGRDHGIPPYTAYRAACGLRPLTGFDD

VDGLGPNVA---QLARVY-----RSVDDVDLFTGLVHEPPAPGSNALVGPTLACILGTQF

YNLKFGDRFFFDNDDKLIAFT------DNQIKSLRNTTLAKVICSNTN------IEALPN

NVFSFSAKSNPLIPCKQLGEESL---------------SLSLFS----------------

------------------------------------------------------------

------------------------------------------------------------

------------------------------------------------------------

------------------------------------------------------------

------------------------------------------------------------

------------------------------------------------------------

------------------------------------------------------------

------------------------------------------------------------

------------------------------------------------------------

------------------------------------------------------------

------------------------------------------------------------

------------------------------------------------------------

------------------------------------------------------------

------------------------------------------------------------

------------------------------------------------------------

------------------------------------------------

>Lsta_jg27183.t2

------------------------------------------------------------

------------------------------------------------------------

------------------------------------------------------------

------------------------------------------------------------

------------------------------------------------------------

------------------------------------------------------------

------------------------------------------------------------

------------------------------------------------------------

------------------------------------------------------------

------------------------------------------------------MFYYFG

--------I-AL--------------------------FVVGQCHG--QDAEK-------

-------------VASAPYSAVC---ELRTHGFYNLPQMSRALSTGSVSDAGAAFRQ---

----------DFRD-LCT----------DVNSKKYRTFDGSCNHPKN--WGTSFKPLARM

LPAEYDNGVS--SPRRRGSLGT-PLPSPRAISRFVH----------PSTPD-LISRTIMI

MQWGQWLDHDLSVSPVASEVNR-VIKCCGPNG-----TRPVYPIDPNCFPIILPSDEENF

A-----GQCMEFVRSSP-----ALDKNNCTLRPREQINVLNSFV----------------

--------DASQVYGSTDELAARLRVEGD--FLLK------TKDEIFLPEDVAS---ACI

KR------PGTNDYCFLAGDVRVNEHASLGAMHTIWLRAHNKIAKQLRA-----------

---------LRPKDSNEEIFQLTRKIIGALQQVITYNEWLPIILGKH-A-TTQ-------

-KLPSKTGRTQRLLSADPRILNEFSTAALRFGHSFIPDVFPIGDR---------------

---------------RVPLRQLFNRPAEVL----DNFDDLVAGITGV---AGGKSAQK--

IDRNFVVEITNHLFEPPTGSPGRGLDLIALNIQRSRDHGIPPYTAYRAACGLRPLTGFDD

VDGLGPNVA---QLARVY-----RSIDDIDLFTGLVHEPASPGSNALVGPTLACILGTQF

YNLKFGDRFFFDTNDKLIAFT------DNQLKSLRNTTLAKVICSNTN------IEALTN

NVFSFSAKANPLVPCQQLEEEGL---------------NLSLFA----------------

------------------------------------------------------------

------------------------------------------------------------

------------------------------------------------------------

------------------------------------------------------------

------------------------------------------------------------

------------------------------------------------------------

------------------------------------------------------------

------------------------------------------------------------

------------------------------------------------------------

------------------------------------------------------------

------------------------------------------------------------

------------------------------------------------------------

------------------------------------------------------------

------------------------------------------------------------

------------------------------------------------------------

------------------------------------------------

>Lsta_jg27183.t1

------------------------------------------------------------

------------------------------------------------------------

------------------------------------------------------------

------------------------------------------------------------

------------------------------------------------------------

------------------------------------------------------------

------------------------------------------------------------

------------------------------------------------------------

------------------------------------------------------------

------------------------------------------------------MFYYFG

--------I-AL--------------------------FVVGQCHG--QDAEK-------

-------------VASAPYS-VC---ELRTHGFYNLPQMSRALSTGSVSDAGAAFRQ---

----------DFRD-LCT----------DVNSKKYRTFDGSCNHPKN--WGTSFKPLARM

LPAEYDNGVS--SPRRRGSLGT-PLPSPRAISRFVH----------PSTPD-LISRTIMI

MQWGQWLDHDLSVSPVASEVNR-VIKCCGPNG-----TRPVYPIDPNCFPIILPSDEENF

A-----GQCMEFVRSSP-----ALDKNNCTLRPREQINVLNSFV----------------

--------DASQVYGSTDELAARLRVEGD--FLLK------TKDEIFLPEDVAS---ACI

KR------PGTNDYCFLAGDVRVNEHASLGAMHTIWLRAHNKIAKQLRA-----------

---------LRPKDSNEEIFQLTRKIIGALQQVITYNEWLPIILGKH-A-TTQ-------

-KLPSKTGRTQRLLSADPRILNEFSTAALRFGHSFIPDVFPIGDR---------------

---------------RVPLRQLFNRPAEVL----DNFDDLVAGITGV---AGGKSAQK--

IDRNFVVEITNHLFEPPTGSPGRGLDLIALNIQRSRDHGIPPYTAYRAACGLRPLTGFDD

VDGLGPNVA---QLARVY-----RSIDDIDLFTGLVHEPASPGSNALVGPTLACILGTQF

YNLKFGDRFFFDTNDKLIAFT------DNQLKSLRNTTLAKVICSNTN------IEALTN

NVFSFSAKANPLVPCQQLEEEGL---------------NLSLFA----------------

------------------------------------------------------------

------------------------------------------------------------

------------------------------------------------------------

------------------------------------------------------------

------------------------------------------------------------

------------------------------------------------------------

------------------------------------------------------------

------------------------------------------------------------

------------------------------------------------------------

------------------------------------------------------------

------------------------------------------------------------

------------------------------------------------------------

------------------------------------------------------------

------------------------------------------------------------

------------------------------------------------------------

------------------------------------------------

>Lsta_jg45101.t2

------------------------------------------------------------

------------------------------------------------------------

------------------------------------------------------------

------------------------------------------------------------

------------------------------------------------------------

------------------------------------------------------------

------------------------------------------------------------

------------------------------------------------------------

------------------------------------------------------------

--------------------------------------------------MGTLSFSYVI

ALTVV-ISRGQ--DVFPADAGDQDFWIRSLNATDMADFADLGLAA---------------

------HRRT---IQSDFITGMC---MKDSDVLYNLPERVGAMSTGSISGFGVRLSQ---

----------NYLQ-DCP----------NYYSKRFRTIDGTCNNPFN--LGASATPVPRI

VPPQYGDGVG--APRTKAADGS-PLPGARFVSSAVH----------PAVNN-LTNRPIIA

MQWGQWIDHDMAGIAASLGPNGTALQCCGPNK-----TAPPAVTSPNCFPILLPPDEKDF

V-----GTCMNFVRSLA-----ATNEQGCEMKQRQQVNTITSFI----------------

--------DASPVYGSTENVARILRNPGD--FLLK------TKNTNFLPENVNG---TCI

RR------PGTKDYCFLAGDFRVNQLPFLQSLHTIWLRAHNTIARRLRV-----------

---------LRPKDSNEETFERTRKLIGALTQAVTYNEWLPFVLGKE-A-TKF-------

-NLVSRTGRTKTSLKVDPRIRQEFSTAAMRFGHSTVPDVFPIGQR---------------

---------------RVPISQLFNRPGEVL----DNFDDVVAGMVGV-GTPGQNLIQQ--

VDRNFVAGLTKHLFEPPNSP-KNGLDLISFNIQRGRDHGIGPYTTYRKLCGLRPLTGFND

VSALGPNVA---QLAKVY-----KSVNDIDLFTGLVHEPVDPSNQGLVGPTLTCILCQQF

LNLKFGDRFFFDTDEKLIAFN------DNQLKWIRQATLSKIICATTN------IKALPN

DIFNFPSKSNPLVSCDRLANDML---------------NLSAFA----------------

------------------------------------------------------------

------------------------------------------------------------

------------------------------------------------------------

------------------------------------------------------------

------------------------------------------------------------

------------------------------------------------------------

------------------------------------------------------------

------------------------------------------------------------

------------------------------------------------------------

------------------------------------------------------------

------------------------------------------------------------

------------------------------------------------------------

------------------------------------------------------------

------------------------------------------------------------

------------------------------------------------------------

------------------------------------------------

>Lsta_jg45101.t1

------------------------------------------------------------

------------------------------------------------------------

------------------------------------------------------------

------------------------------------------------------------

------------------------------------------------------------

------------------------------------------------------------

------------------------------------------------------------

------------------------------------------------------------

------------------------------------------------------------

--------------------------------------------------MGTLSFSYVI

ALTVV-ISRGQ-ADVFPADAGDQDFWIRSLNATDMADFADLGLAA---------------

------HRRT---IQSDFITGMC---MKDSDVLYNLPERVGAMSTGSISGFGVRLSQ---

----------NYLQ-DCP----------NYYSKRFRTIDGTCNNPFN--LGASATPVPRI

VPPQYGDGVG--APRTKAADGS-PLPGARFVSSAVH----------PAVNN-LTNRPIIA

MQWGQWIDHDMAGIAASLGPNGTALQCCGPNK-----TAPPAVTSPNCFPILLPPDEKDF

V-----GTCMNFVRSLA-----ATNEQGCEMKQRQQVNTITSFI----------------

--------DASPVYGSTENVARILRNPGD--FLLK------TKNTNFLPENVNG---TCI

RR------PGTKDYCFLAGDFRVNQLPFLQSLHTIWLRAHNTIARRLRV-----------

---------LRPKDSNEETFERTRKLIGALTQAVTYNEWLPFVLGKE-A-TKF-------

-NLVSRTGRTKTSLKVDPRIRQEFSTAAMRFGHSTVPDVFPIGQR---------------

---------------RVPISQLFNRPGEVL----DNFDDVVAGMVGV-GTPGQNLIQQ--

VDRNFVAGLTKHLFEPPNSP-KNGLDLISFNIQRGRDHGIGPYTTYRKLCGLRPLTGFND

VSALGPNVA---QLAKVY-----KSVNDIDLFTGLVHEPVDPSNQGLVGPTLTCILCQQF

LNLKFGDRFFFDTDEKLIAFN------DNQLKWIRQATLSKIICATTN------IKALPN

DIFNFPSKSNPLVSCDRLANDML---------------NLSAFA----------------

------------------------------------------------------------

------------------------------------------------------------

------------------------------------------------------------

------------------------------------------------------------

------------------------------------------------------------

------------------------------------------------------------

------------------------------------------------------------

------------------------------------------------------------

------------------------------------------------------------

------------------------------------------------------------

------------------------------------------------------------

------------------------------------------------------------

------------------------------------------------------------

------------------------------------------------------------

------------------------------------------------------------

------------------------------------------------

>Lsta_jg44931.t1

------------------------------------------------------------

------------------------------------------------------------

------------------------------------------------------------

------------------------------------------------------------

------------------------------------------------------------

------------------------------------------------------------

------------------------------------------------------------

------------------------------------------------------------

------------------------------------------------------------

------------------------------------------------------------

--------------------------------------------MQLRQICLCYLCI---

--LNNLEIGR---TQEPTPEQT---NPKPVQEFYVLASTHAAMSSGMFSGRGFKLTR---

----------RWYT-WCL----------NATSYKYRTYDGSCNNVRN--LGAAITAVPTL

LKSEYADGMD--SPRQYGEDGL-PLPSARLVSTSVF----------PSAIE-LTNRTHMV

MQWGQWVIHDLSAIPISTGANG-TIKCCGPKG-----QPPPFINNRNCFPIEIPIHDRIS

K-----GRCMEFVRSLA-----ATSRMGFTLKPRQQINIITSFI----------------

--------DASQIYGSGTIQTNGLR-YGG--YLLK------TGLHGALPKAETQ---NCI

TR------PNTTDYCQLAGDTRVNEQPALGVIHTFWVREHNRISQQLRV-----------

---------LKGNSSEEEIFQITRKIIAALQQIINYNEYLPIILGKD-A-HTW-------

-NLVSKVGRTAYNPALDPSVFNEFSTAAFRFGHSTIPDVLSFSDK---------------

---------------AVKMRFLLHRPAECL----NNCEGLMAGLAGLVGKKKKKALKK--

VDRNFVKEVTRFLFEAQASP-GHKLDLAALNIQRGRDHGLAPYIKYRTFCGLPPVKGFDD

EEALGPNVR---DLALVY-----RSIADIDLFVGLLYEPVKG--DASVGPTLKCLLGIQF

FNLKFGDRFFFDTTDETLGFN------DEQLASLRNMTLSKVMCHNSD------LRELQL

DVFTLPSKQNPTYTCQQLRSESI---------------DLTLFA----------------

------------------------------------------------------------

------------------------------------------------------------

------------------------------------------------------------

------------------------------------------------------------

------------------------------------------------------------

------------------------------------------------------------

------------------------------------------------------------

------------------------------------------------------------

------------------------------------------------------------

------------------------------------------------------------

------------------------------------------------------------

------------------------------------------------------------

------------------------------------------------------------

------------------------------------------------------------

------------------------------------------------------------

------------------------------------------------

>Lsta_jg37361.t1

------------------------------------------------------------

------------------------------------------------------------

------------------------------------------------------------

------------------------------------------------------------

------------------------------------------------------------

------------------------------------------------------------

------------------------------------------------------------

------------------------------------------------------------

------------------------------------------------------------

------------------------------------------------------------

--------------------------------------------------MGEIWELR--

-ALWLLGVCVV--LCARWPGVGACR-----------------------------FKD---

----------EIRERRHNVRRDV--AACDP-TSRYRSFDGSCNNLRHPTFGVAGSTFRRI

LAPKYENGDGR-TPRLTGVTGA-KLPSPRLVSVAVH----------DPGNHTAVNVNLMV

MQWAQFLDHDVTRTPTTVA----SAPCCTAELVRSGVLHPDVTTGGPCFPIIVGSNDRLF

TD--LTTRCRDFTRSDP-----AVDDNG----IRQQYNAITPWI----------------

--------DGSQIYGHTEELARSLRTLVN--GKLRVVT---INGEDFLPADEAATDETCF

K-------LQPGDYCFKAGDVRVNAYPGLSALHTMFLRYHNKICDRLKA-----------

---------IHGDWSDEILYQEARRLVIAVIQRISYDEFMVQILGQA-A-SKY-------

-GLL--FGNYSYNSSIDPALSNVFSTAAYRFGHSLVSDSLTINGQ---------------

---------------VVETGDLFMRPKFVL----NSLKNLTEALLT-------ENCQR--

ADRWYTKGMTDRMFEKPGGP-KTGQDIVALNIQRGRDHGLPPYNEWLAYFGLPIKT-F--

-DTMEQGAI---RYRGVY-----SSVDDIDLYSGAIGEFPVN--GGSVGGLYSLILGDQF

RDLKFGDRFWFENLGDVSSFT------SDQVKQIARIKFSKVICDTVTGAD--GIKKVQL

NAFRPVSSTNVLTDCAAFPDLDI---------------ERFWV-----------------

------------------------------------------------------------

------------------------------------------------------------

------------------------------------------------------------

------------------------------------------------------------

------------------------------------------------------------

------------------------------------------------------------

------------------------------------------------------------

------------------------------------------------------------

------------------------------------------------------------

------------------------------------------------------------

------------------------------------------------------------

------------------------------------------------------------

------------------------------------------------------------

------------------------------------------------------------

------------------------------------------------------------

------------------------------------------------

>Lsta_jg37362.t1

------------------------------------------------------------

------------------------------------------------------------

------------------------------------------------------------

------------------------------------------------------------

------------------------------------------------------------

------------------------------------------------------------

------------------------------------------------------------

------------------------------------------------------------

------------------------------------------------------------

------------------------------------------------------------

-----------------------------------------------------MLAFS--

-VNRMFLSLLF--LCVCWRHVSCYD-----------------------------EDH---

----------EEERHGVDPSGRN--VTCDP-TSRYRSFDGSCNNLRHPTFGVAGSTFRRI

LPPKYENGDGR-TPRLTGVTGA-KLPSPRLVSVTVH----------GPGNDTADDANMML

TQWGQFLAHDLTRTLQASF----NGSCCSSDLVTSGVLHPDVVNGGPCFPIIVDRMDRHF

DD--VSTRCMEFQRSDH-----RTDLTN----TRQQYNEATSWI----------------

--------DASHIYGHTEDRARSLRSFNN--GKLKVIT---VNGEDFLPENVEATEKTCF

K-------LQAGDYCFKAGDVRVNFYPGLSALHTMFLRYHNKICDRLKP-----------

---------IHGDWSDEILYQEARRLVIAVIQRITYDEFMVQILGQA-A-SKY-------

-GLL--FGNYSYNSSIDPTLSNVFSTAAYRFGHSLVSDSLTINGQ---------------

---------------VVETGDVFTRPKFVL----NSLKGVTEAFLR-------GRSHG--

ADRWFAKGMTDRMFEKPNKP-KTGFDIVARNIQRGRDHGIRPYNDWLEHFGFPRAT-F--

-ETIGQ------VYGRVY-----SSVDDVDLYGGAAGETRAY--QGRVGQLYSAILGAQF

RDLKFGDRFWFENLDDVSSFT------ADQVSQIAGLKLSRVICDTVAG-----ITKIQL

NAFQTVSPENSLADCSTLADVDI---------------ERFWV-----------------

------------------------------------------------------------

------------------------------------------------------------

------------------------------------------------------------

------------------------------------------------------------

------------------------------------------------------------

------------------------------------------------------------

------------------------------------------------------------

------------------------------------------------------------

------------------------------------------------------------

------------------------------------------------------------

------------------------------------------------------------

------------------------------------------------------------

------------------------------------------------------------

------------------------------------------------------------

------------------------------------------------------------

------------------------------------------------

>Lsta_jg31272.t1

------------------------------------------------------------

------------------------------------------------------------

------------------------------------------------------------

------------------------------------------------------------

------------------------------------------------------------

------------------------------------------------------------

------------------------------------------------------------

------------------------------------------------------------

------------------------------------------------------------

------------------------------------------------------------

------------------------------------------------------------

--------------------------------------------MKSWSLLLTGFI----

--------------VCCSVQT------LIGIEQERSPGDGWYNNLLHPDWGAIDTHLLRR

SKVSYSDGVYE-------PSGI-NRPNPLTISRIAF--------NGTNNLSSVRNRNALL

VFFGQQLVEEIMDAQR--------------------PGCP---IE--YLNIPVPK-DHKY

NPDQIDDLEMPFLRSR-----YDQRTGFSPNNPRQQLNEITPYM----------------

--------DGNLIYGSGKSVEDAVRSFRDGELLADNDD----IKKSFPMKND-IRLPFAN

PPSPRDHVLRPVSRFRRFGNPRTHENPLLYSLAVVWFRYHNVIARQLKT-----------

-------T--FPQLDDEQLFNAARKRVLAQYQKIVMYEWLPAWLSISEKGEKFNITGDYP

YNGGGQNPYKGYDPNVHPGISTEFQAAAFRFGHTLVPPGIFTKKFESGTCINSTRPVQAK

FTSKPNGAEENVDIEGIRLCNAYWVPQETV-ETETGMDEIIRGLTFT-------KATK--

EDNIIVTDLREDVFGPLD---WSRRDLGALNIQRARDLGLPGYNDVREAYGLKRISNWTQ

INAEGLYGTQLRELKRLYN--NSESPDDLDLFVGGLLETVPN--G--PGPLFQAIILDQF

LRIRHGDRFWYENTQN-GLFT------ADEIRDIENTNFYDVLKNVTNAFSMPQLVELGN

DVFSCSNVNRTSKECQCVDPFLD---IQDPHEQCVPLQHYDYFTGSAFPFIITIAAIVVS

LPLTI----GIMLLIARLRRMSMTPKTS-KTK-----EKPQNGPNYFYATEWVGRTTYGT

LNSRDVKIELDNQRMKILVSNISGQVVRMIDVRQRNNAEQKKPRASVTRSSDKGNRLMMM

AAPGEIDLVLHFASRTDRDEAFEKLHEFFKKHGWEYHEAPNMAEQIMWREAMTIDQRKEV

LARFFKSILVELSGQDSTLN------RDPALVDEALNTRLTRTEFADALGLQPHSLFVRN

MFLLVDSSGDGFVSFDEFKTYFGILASGKPEDKAKMFFQMFDTSRTGKLTKDNYKKMIMS

LMELNEAGDGQNMNINNMVDAVFKQLGKDKVGFLTLEEFKSIMFSDTDDVWKSAVLNLDV

GGETATIGKHKRSTVRDRAKSFIQGYQRNTKATSMVNFRSS---QHVRLSSKADTAPKTN

Y----QKFCRYVSNHNRQIFWVTLYTLVTLGIFVERAFYYSFEREHAGLRRMAGYGVSVT

RGAASAQMFTYASLLVTMSRNTLTFFRETFLHRYIPFDNAHDMHFYVAGLAVLFTVIHVI

GHVINFYHISTQPSSDLNCYFTEFFRPTHVLASFQYWTYNTITGLTGVGLVFVLVVLYVF

AIPYARRNVFNFFRATHNLYIVVYIMLFLHGHARLVQVPLWPYYFLGPMVLFVLDKLVSV

SRNKILLPVVRATLLPSGVINLIIKRPLTFNYQSGQWVRIACPNLGKGEYHPFTLTSAPH

EQHLSLHIRAVGPWTSNFRHLFDPNVQQRSE-IPK--IYLDGPFGESHQDWYRYPVSVLV

GGGIGITPFASILKDIANRSRE--VGRLPCRKVYFVWVTRTQQSFEWMTEIIRQVEAADT

QDFVDINICITQIKEKFDLRTTMLYICERHFQKIA-GMSMFTGLRARTHFGRPKFQDFFE

ALKFVHKEVGEIGVFSCGPPAMTNSVQQACTEQNAFTGPSLIHHFENF

>Lsta_jg65571.t1

------------------------------------------------------------

------------------------------------------------------------

------------------------------------------------------------

------------------------------------------------------------

------------------------------------------------------------

------------------------------------------------------------

------------------------------------------------------------

------------------------------------------------------------

------------------------------------------------------------

------------------------------------------------------------

------------------------------------------------------------

-----------------------------------------------MELSLRG-V----

---------VLA--GFI----IALSGGVSFEEVPVQRFDGWYNNPLNPSWGGVGRALERN

ITPAYADDTYR-------PSGW-DRPNPRWISNELF----GADHLPRGQVANGRNLTALF

AFFGQIVQHELIDTDD--------------------VTCP---IE--ILETPVPRGDPEF

DPEAEGNKHLPYERTS-----YDQNTGQSPNNPRRQLNRASSYIDGRFLLLNKFNSCLRS

LNRASSYIDGSFLYGNSLVRTEFLRQPNSGKLACED------KWGKFPMRNN-VNLPYHA

FS----FKFTRSEQLWRLGDTHVFENPAVLALNLAFYRYHNKVVDDILA-----------

-------GGDHRDKNSDELFDLARRQVIANIQNVMVYEWLPTLIGME-------------

-----LPPYTSYKSWVKSDVTDIFDAGAINYIMTLIPSGISQLEKTN--CLVR-------

---------NDGHFPAKRLCKTYWNAMETVYEHEDSTAEIILGLAHQ-------MAEN--

DDTYVVEDYRNKFYGPAY---HTNHDAVLLTIMKGRDYGLPDYNTVRRTMGLEPKKSFED

VNIELNRTNP--ELMEAFKRMHKNDLSTVDMFVGGMMETTPD--G--PGELFRHILYDQF

IRLRDGDRFWFENKEN-KLFT------DDQIKAIRNTTLADILNKATDIPK-K---RLKD

VFVN------YGTDKECGPQRFF---KTEELDDCPMNTSHDFFAGSEIPYIIIWTCLGLL

PLVCIFVAYLLAKCKKWRHQRLLNSLKVEKEKRRILRRTISCSVSVHDALEWRGHDVAP-

---RPVELHLTNKGCLEIFS-TAGAHLRSIK-------VSTMPTLAIKMSSNKKRNVLGI

IMPREYDLIVEFPSEPMRIEFGTMLCTFSQEHNVDLSVIE-LRLQDLYNAATTKEKRNRM

LEKFFKTVFSEAFQMDYDPSLDAGSLDMKKHSQEILEVELSKEEFAEAMAVKANSDFVEH

FFSLIDSDRNGYISFREFLNAVVLFSKGSIQEKLQTMFYMYDIDGTGYMSTKQIGQMFRS

LLELAQSNLD-TDDIDCLLESLSSQSGMEKKEQYSFEDFCQLLSPQMDKLWNASFDW---

KGLSNVMPPKTKSPKKAA--NGEPNLKKRGSVASLGALENGGSGSRFAISFEAVREKYTP

LKAKVKLVKHFIENYRQHIFFMILFYGICAGLFAERFYYYTVEQEHKGFRKLMSYGISFT

RGAAASMSFTFSFLLLTMCRNTITFLRSTPLNLFIPFDSHVSFHKIVAWTALFFSAIHII

GYSFNFYHLVSQPTRFL-CIFTSLVFRPEFPLSFQQWVFGTMPGFTGVLLVLVLCILYVF

ATQTARRHIFSLFWLTHKLFIILYVLTIIHGASVVVQKPMFFAYLSGPAILFMIDKLVSL

SRKKTELCIINAKNLPSDVTMIEFKRPPRFEYKSGQWVRIACLAQGKDEYHPFTLTSAPH

EDTLKLHIRALGPWTWNLRHIFETESLKSSKSYPK--LYLDGPYGAGQQDWYQYDVSVLV

GAGIGVTPYASILKDFVHMASINMRYKVKCQKLYFIWITGSQRHFEWLLDIIREVEEVDT

QGMVSIDIFITQFFQNFDLRTSLLYIFEEHFQKMNGGKSVFTGLKATTHFGRPQMNKIME

AVHRAHPQVRKVGVFSCGPPGVTKGVERACVDTSKVTKAMFEHHFENF
