# Supplementary material for: Mantle Modularity Underlies the Plasticity of the Molluscan Shell: Supporting Data From Cepaea nemoralis
Source: Front Genet. 2021 Feb 5;12:622400. doi: 10.3389/fgene.2021.622400 (PMC7894901; doi:10.3389/fgene.2021.622400)
Supplement: Supplementary file 3 [file Data_Sheet_3.docx]

>Cnem_R37577449

RQIDGRCNHP RNGSTGRPVK RYLRPHYQDD GSAVYGSDLD TYTWIRCQLA GDMRVNEQPG

LGSIHLLFHL HHNHIVRLLE QIFQEVRKML GAIIQKLTYC DWLPMILGAA LRFGHTLIPD

RYVSSAVSEH LFESALDLIA VNIQRGRDHG IPAYHYWRQY YRLRRIIDIR DVDLFPGGLL

EPVGETFGHI LANQFADLKF GDTYFFL

>Cnem_R37442076

RTFNGTCNHP KNGASFTIQD RFLPPAYSDD ASTVYGSTNE LAESIRCFHA GDFRVNQHPF

IGAIHTLFLR EHNRICRRFE KIFQMGRKLV GAMNQHFTYN VFLPLIMGAA FRFGHSTIPD

KYVEDDVSKF LFFNGLDLIS INLQRARDHG VQPYYKWRAF CGLRPLTDVR DIDLFTGMLH

EPVGPTISCI VGMQFKRLKH GDRFFFN

>Cnem_R37435138

RTFDGTCNHP RNGSSFTPVS RLLPPVYDDD GSQIYGATSE RAALLRCFIT GDLRTHENPA

LTALHVIWMR EHNRMARELE EIYQIIRKIV GALQQIFTLN HYLPIVLGAA FRFGHSSIPD

RNFVEEITQH LFEPGLDLVS LNIQRARDHG LPPYTKWRTF CGLRPVYSVN DIDLFTAIVH

EPLGPTGRCL VGIQFARAKF GDRFIFD

>Cnem_R37509337

RTFDGTCNHP RNGAESDLFD NLLPQEYDDD CSQIYGSTND VAASLRCFLA GDFRANQHTY

LSAFHTLWLR EHNYIATELE DKFQTTRKII IGMHQVFTYN TFLPIMLGGA FRFGHSTTPD

KFITDELSRF LFFNGFDLNS VDIQRGRDHG IPPYVKWRSF CGLRPLTSID DMDLFTGMLH

EPVGPTIACL MRIQFHNTKY CDRFFFD

>Cnem_R37580477

RTNDGTCNHK DNGSQFAEID RLLPPTYADD SSNVYGSDDE VAQAVRCFLT GDHRPNQHTY

LSAIHTFWLR EHNRIATELE TIFQTAKQIV NAVNQAVTYN EYLPTLLGAA FRLGHSQAPD

KHFTEELTQF LLYNGGDLLS TNIQRGRDHG LPTYVQVRDF CCLPPITSVK DIDLYTGMLH

ELAGPTIRCL VGLQFYRLKY GDKYFFD

>Cnem_R38134193

RSIDGSCNHV LTGAAGSVID KLLPPTYDDD SSQIYGSSHE VEQSVRCVLA GDNRCNQHSI

LTFFHTFSMR AHNRMATQLE TIYQTVKSII NAIYQSILYN EYLPIVLGAA MRFGHSQIPD

QYFTEDVSRY LFFNGSDLPA TNIQRGRDQG LATYSQVREI CCLPPMTSVD DIDLYVGGLL

EVLGPTFNCL VGLQYYTLKH SDRFFFD

>Cnem_R37516884

RTMDGTCNNL QNGAANTAFN RLLPAIYEND ASNVYGSTDR MASTLRCFIA GDVRVNEQLA

LIAMHTLWMR QHNQMAVELN KIYHEARKIM GALFQHITYK HWLPKVLGAA MRFGHTLIQG

ELLNSELTEK LFQLGQDLAS LNIQRGRDHG TQFYNDYRVL CGLPRARHPD NIDLFVGGVS

EYVGPTFLCI LSDQFKRLRD GDRFWYE

>Cnem_R38332823

QRFDGWYNNP LNGGVGRPLE RNITPTYEDD GSFLYGKSLV RTEFLRLWRL GDPDVFENPA

ILALNMIFYR YHNAKAEEFE EVFDKTRRWV IGCIQKIIVY DWLPMLIEGA INYIMTLIPD

TFVVEDYRSK FYGPKHDAVL LTIMKGRDYG LPDYNTVRVT MGLKEMTNLS TIDMFVGGMM

ESPGELFTHI LYDQFIRLRD GDRFWFE

>Cnem_R38307666

APVDGFRNNL GQGIAGSNFA RVSPAAYSDD GGIIYGTSKT WTSLVRLFRL GNAKGHENPF

LLALQVIWFR WHNTIAGDIE QIFQTAKKMV VAHFQKILVT DWLPAFLYAA MDFRHTFTPD

GRFVSDFTEF FYGPRTDHVA AQIQRYRDHG LSGLNTIRRS YGLATLSKPD NVDILTGGLL

DQLSEIFRKI LIDQFTILRD GDRFWYQ

>Lsta_jg60788.t1

REIDGRCNHP KDGSTMKPLK RYLKAHYQDD GSAFYGSDLN ATKRLRCMVT GDRRANLHPG

LSSAHLLFHL YHNFVVRSLE KLFQEGRKVM GGIFQNIVFC DYLPIILGAA FRFGHTLVPD

WFFSKSISEH LFETAIDLLS VNIQRARDHG IPAYYYWRRY YGLRRFTHVN DVDLFVGGVF

EPVGETFGHI IANQFADLKY GDAYYFL

>Lsta_jg17016.t1

RTTDGNCNHP KNGTSFKPVT RLLPADYSDD ASQVYGSTEE TAAKVRCFLA GDFRVNEHPA

LAAMHTIWLR LHNKIASNLD DVFQLTRKIV GALQQVITYN EWLPIILGAA MRFGHSLIPD

RNFVTEITNH LFEPGLDLVA LNIQRARDHG IPPYKAYRAF CGLRPLTSVD DIDLFTGLVH

EPVGPTLSCI LGTQFYNLKF GDRFFFD

>Lsta_jg27188.t1

RTIDGSCNNL RNGTSFKPVS RILPARYQDD ASQVYGSTDA VAASVRCFLA GDFRVNQHPY

LQSLHTVFLR DHNRIARKLE NIFQLSRKII GALQQMITYN EWLPVILGAA MRFGHSLIPD

RNFVVEITKH LFEPGLDLVS FNIQRGRDHG IAPYTTYRAL CGLRPVRSVN DIDLFTGLVH

EPVGPTLACI LGTQFYNLKF GDRFFFD

>Lsta_jg45104.t1

RTIDGNCNHP KNGTSFKPLA RMLPAEYDDD ASQVYGSTDD LAARLRCFLA GDIRVNEHAS

LGAMHTIWLR AHNKIAKQLE EIFQLTRKII GALQQVITYN EWLPIILGAA LRFGHSFIPD

RNFVAEITNH LFEPGLDLIA LNIQRGRDHG IPPYTAYRAA CGLRPLTSVD DVDLFTGLVH

EPVGPTLACI LGTQFYNLKF GDRFFFD

>Lsta_jg27183.t2

RTFDGSCNHP KNGTSFKPLA RMLPAEYDND ASQVYGSTDE LAARLRCFLA GDVRVNEHAS

LGAMHTIWLR AHNKIAKQLE EIFQLTRKII GALQQVITYN EWLPIILGAA LRFGHSFIPD

RNFVVEITNH LFEPGLDLIA LNIQRSRDHG IPPYTAYRAA CGLRPLTSID DIDLFTGLVH

EPVGPTLACI LGTQFYNLKF GDRFFFD

>Lsta_jg27183.t1

RTFDGSCNHP KNGTSFKPLA RMLPAEYDND ASQVYGSTDE LAARLRCFLA GDVRVNEHAS

LGAMHTIWLR AHNKIAKQLE EIFQLTRKII GALQQVITYN EWLPIILGAA LRFGHSFIPD

RNFVVEITNH LFEPGLDLIA LNIQRSRDHG IPPYTAYRAA CGLRPLTSID DIDLFTGLVH

EPVGPTLACI LGTQFYNLKF GDRFFFD

>Lsta_jg45101.t2

RTIDGTCNNP FNGASATPVP RIVPPQYGDD ASPVYGSTEN VARILRCFLA GDFRVNQLPF

LQSLHTIWLR AHNTIARRLE ETFERTRKLI GALTQAVTYN EWLPFVLGAA MRFGHSTVPD

RNFVAGLTKH LFEPGLDLIS FNIQRGRDHG IGPYTTYRKL CGLRPLTSVN DIDLFTGLVH

EPVGPTLTCI LCQQFLNLKF GDRFFFD

>Lsta_jg45101.t1

RTIDGTCNNP FNGASATPVP RIVPPQYGDD ASPVYGSTEN VARILRCFLA GDFRVNQLPF

LQSLHTIWLR AHNTIARRLE ETFERTRKLI GALTQAVTYN EWLPFVLGAA MRFGHSTVPD

RNFVAGLTKH LFEPGLDLIS FNIQRGRDHG IGPYTTYRKL CGLRPLTSVN DIDLFTGLVH

EPVGPTLTCI LCQQFLNLKF GDRFFFD

>Lsta_jg44931.t1

RTYDGSCNNV RNGAAITAVP TLLKSEYADD ASQIYGSGTI QTNGLRCQLA GDTRVNEQPA

LGVIHTFWVR EHNRISQQLE EIFQITRKII AALQQIINYN EYLPIILGAA FRFGHSTIPD

RNFVKEVTRF LFEAKLDLAA LNIQRGRDHG LAPYIKYRTF CGLPPVKSIA DIDLFVGLLY

EPVGPTLKCL LGIQFFNLKF GDRFFFD

>Lsta_jg37361.t1

RSFDGSCNNL RHGVAGSTFR RILAPKYEND GSQIYGHTEE LARSLRCFKA GDVRVNAYPG

LSALHTMFLR YHNKICDRLE ILYQEARRLV IAVIQRISYD EFMVQILGAA YRFGHSLVSD

RWYTKGMTDR MFEKGQDIVA LNIQRGRDHG LPPYNEWLAY FGLPIKTSVD DIDLYSGAIG

EFVGGLYSLI LGDQFRDLKF GDRFWFE

>Lsta_jg37362.t1

RSFDGSCNNL RHGVAGSTFR RILPPKYEND ASHIYGHTED RARSLRCFKA GDVRVNFYPG

LSALHTMFLR YHNKICDRLE ILYQEARRLV IAVIQRITYD EFMVQILGAA YRFGHSLVSD

RWFAKGMTDR MFEKGFDIVA RNIQRGRDHG IRPYNDWLEH FGFPRATSVD DVDLYGGAAG

ETVGQLYSAI LGAQFRDLKF GDRFWFE

>Lsta_jg31272.t1

SPGDGWYNNL LHGAIDTHLL RRSKVSYSDD GNLIYGSGKS VEDAVRFRRF GNPRTHENPL

LYSLAVVWFR YHNVIARQLE QLFNAARKRV LAQYQKIVMY EWLPAWLSAA FRFGHTLVPD

NIIVTDLRED VFGPRRDLGA LNIQRARDLG LPGYNDVREA YGLKRISSPD DLDLFVGGLL

ETPGPLFQAI ILDQFLRIRH GDRFWYE

>Lsta_jg65571.t1

QRFDGWYNNP LNGGVGRALE RNITPAYADD GSFLYGNSLV RTEFLRLWRL GDTHVFENPA

VLALNLAFYR YHNKVVDDID ELFDLARRQV IANIQNVMVY EWLPTLIGGA INYIMTLIPD

TYVVEDYRNK FYGPNHDAVL LTIMKGRDYG LPDYNTVRRT MGLEPKKDLS TVDMFVGGMM

ETPGELFRHI LYDQFIRLRD GDRFWFE
