# Supplementary material for: Mantle Modularity Underlies the Plasticity of the Molluscan Shell: Supporting Data From Cepaea nemoralis
Source: Front Genet. 2021 Feb 5;12:622400. doi: 10.3389/fgene.2021.622400 (PMC7894901; doi:10.3389/fgene.2021.622400)
Supplement: Supplementary file 4 [file Data_Sheet_4.docx]

>Cnem_R27072766 TransAbyss assembly 2 (filtered min reads 10, dedupe95) len=2716 num_reads=5670257 avg_cov=209491.4 contig_cov=100.0% (contig_821 from old CLC assemly 9)cds start = 286 cds stop = 2466 strand = -protein length = 727 strand = +

------------------------------------------------------------

------------------------------------------------------------

------------------------------------------------------------

------------------------------------------------------------

------------------------------------------------------------

------------------------------------------------------------

------------------------------------------------------------

------------------------------------------------------------

------------------------------------------------------------

------------------------------------------------------------

------------------------------------------------------------

------------------------------------------------------------

------------------------------------------------------------

------------------------------------------------------------

------------------------------------------------------------

------------------------------------------------------------

------------------------------------------------------------

------------------------------------------------------------

------------------------------------------------------------

------------------------------------------------------------

------MAMLRYP-LAQFALA-------------------------------SCLLVFLS

PMTAAYDQQTGADDQEIET---WLESL----VKQYQSDAGQQQQTH--------------

--------------YFTQDQLDYIK-----------TLLNKM----KPTAVNDD------

--LK-----------------------------------------QEI------------

------------------------------------------------------------

----------------------------------------VDHFFPK----DSEENVTQ-

-EEQQ---------TT----------------------TPAD-----SEEQ---------

AKEDSS--------------------EEQVTQEQQTTPAES----VEQVKQEQQTITPE-

DSEEQT--TQQEQQTTTPA----------NSEEDTTL----EQQTTTPADSEEDTT-QQE

EQ----------------T-----TTPADSEEDTTQQE--QQ---------TTTPADSEE

------EKQDSDDTDGDDNSDE-----------D------------STE-T---TT-S-T

TTLAPATTTET-------------------------------------------------

------------------------------------------------TTTTTTTTTTTT

-------EV--------ITTP--------------------------EAT---T-TTTEA

EPV-----TTTTTTTTIGAITT----VKPSLELCNNCVVHHGVGYAPLPGYCDAYVQCRF

YGAL-PTAVDIRRCPSGNYWNQDKLVCDFQDNVKCTP-VNNCPNH------KAIPGDWAA

YSIFNGANWTRVACPERRLYNSVTC--GCTDITGGFDGNHEICTDKKAIIGDNTGFMQFT

GN--GWVRMACPATLGYNEQTCRCTDKLSPDTSISVC-----PNTKPIAGDKSGYLQF-T

GV-SWIRRPCPATLVYHADICVCSYDQTNVVD------------------------DDD-

NKSKQHGVCKATVALNFDNNNATDSSVNHFWVNNTGVT--FNDGKAYFNGKSRLTIPGLS

NMEFGSTVYILIKYRHSSA-----------------------------------------

------------------------------------------------------------

--------NSQQTLVSNGDCQ--------------V-RQSLAVCSGKD---SVDFYAETK

EQISLGKTTVP-------------------------------------------------

------------------------------------------------------------

------------------------------------------------------------

----------------------------------------TDVGAWQYALYALDNGNLLG

SVGVNKIAQPVK--GALDRRQRGLVIG----------GGGGCDNFHGII-----------

-DDVRVYLCKPEL-----------------------------------------------

------------------------------------------------------------

------------------------------------------------------------

------------------------------------------------------------

------------------------------------------------------------

------------------

>Lsta_jg75923.t1 gene=jg75923 CDS=1-2154 protein length = 717 strand = + Lstag_sfc_22

------------------------------------------------------------

------------------------------------------------------------

------------------------------------------------------------

------------------------------------------------------------

------------------------------------------------------------

------------------------------------------------------------

------------------------------------------------------------

------------------------------------------------------------

------------------------------------------------------------

------------------------------------------------------------

------------------------------------------------------------

------------------------------------------------------------

------------------------------------------------------------

------------------------------------------------------------

------------------------------------------------------------

------------------------------------------------------------

------------------------------------------------------------

------------------------------------------------------------

------------------------------------------------------------

------------------------------------------------------------

--------------MNLFATL-------------------------------FAASLLLA

NVAR---SFADDDATPTDP---WIETL----LHDYRPQGLQQR--Q--------------

--------------AVSQHTLDYIK-----------RLFHGL----TFGELNEE------

--QQ-----------------------------------------EEI------------

------------------------------------------------------------

----------------------------------------E-EYFGQ----HNVQGLSS-

-EEQE---------EQ----------------------KTVT-----YGQQ---------

TTT---------------------------SQTNQKDSEES----QEEQKTYTKQITQD-

DSDESQ--EDDSHEQDSSS----------SSEEES------KQQTTAANKIPTSTYKAVQ

HK----------------E-----TTPKKDEDSSSEED--KDF------CKGFKPYTADT

------R--FYLQYDGSSWLKM-----PCPSGLG------------FDH-V---TCAC-S

ILVTPHKSSTTTKRI-------IT------------------------------------

------------------------------------------------STTTTPTTTTTR

-------RT--------TTTP--------------------------ITT---T--TTTP

TTT-----TTTPTTTTTRKTST----PKPNFDLCKDCVILHGVGYAAVAGHCDAYIQCRY

YGDI-PTAVSIRRCPYGLQWNQNKLTCDKEENVKCVS-VSKCLNQ------KSIEGDRRG

YLAFNGYTWSRVSCPSDYTYSDVTC--GCTETYQG-HGNFETCNDKKAIPNDRTGFLQLA

PS--GWVRMPCPKSLGYDANTCRCTDKLDVVEYVNHC-----PDLKPLAGDKTGFLQF-N

GV-SWIRMPCPATLGYDPRTCRCTYMNEIEDD------------------------ESDS

IDKDDDGFCKPSLSLSFDDNTATDLSENQFWINNTGVT--FKNGQAYFDGNSRLTVPGFS

NMEFGNKVYVTVKYSQS-G-----------------------------------------

------------------------------------------------------------

--------NTKQTLLTNGDCG--------------I-LQSIGVCTDME---AVDFFAETE

V--KQVKVTVP-------------------------------------------------

------------------------------------------------------------

------------------------------------------------------------

----------------------------------------KMTNSWQYAMYALDKGHLQG

YVGTRTHTQEIK--GSLLRRQRGLIIG----------GGGECGDFTGII-----------

-DELHVFFCRPDFS----------------------------------------------

------------------------------------------------------------

------------------------------------------------------------

------------------------------------------------------------

------------------------------------------------------------

------------------

>Lsta_jg74475.t1 gene=jg74475 CDS=1-2505 protein length = 834 strand = +

------------------------------------------------------------

------------------------------------------------------------

------------------------------------------------------------

------------------------------------------------------------

------------------------------------------------------------

------------------------------------------------------------

------------------------------------------------------------

------------------------------------------------------------

------------------------------------------------------------

------------------------------------------------------------

------------------------------------------------------------

------------------------------------------------------------

------------------------------------------------------------

------------------------------------------------------------

------------------------------------------------------------

------------------------------------------------------------

------------------------------------------------------------

------------------------------------------------------------

----------------------MDLAKM--------------------------------

------------------------------------------------------------

------------HL-LAIVLL-------------------------------VCRYGARA

D---------------DDDFPGWPS--IG-MGGDSDSGEGGYAWGGSWNPSA--------

-HNLFGNAHHAGYNIFQHHQDQQLA-----------DPSPPKATTVQPQNLYSYNQAPAG

TDQTSFRFP-----------YGLQT---GSSPSKNPYARLQPVRQHHNLHPHAMYTDRNP

FRFLAMGGTSDSGENFGGYNIQ-NLLLNHNAR--KSPAGG------DSSQELSGLVAKGA

GKNKGGKARG----------GQKV-----S---GKSYPLFEGDLHAE-----SSSE-ESQ

VRR-----------------DGVLPGSDSSGVIIGSGSQSSSEGSSENGNN---------

GGGRSTHRPYP------SPATK----WPS-----TSD-----------------------

-------------------------------------------RSSKATPGPETTT-P--

---------------------------HITSKRTS-------------------------

---------------------SSHT--PRPPTAPT-----------STA-T---TAPTST

T-TKSPSTTTRRRTTTTTTTTTITAAT-SSTTQKITT------TRPTTTASP-TTR--TT

AKVT-----RPTTKP--------------------------STSPSTTRSTTRPATTTTT

------R--TSTRPTPA-TTP-----AKP---------RLTTT---TTSTTPRPATNTTT

-----T--ARTTSTERLTTTER----PNI-PEICEGGAMVNGVSYRSHWADCTKFIQCSF

TPDGA-VNVFIKSCRHGMYWDQTKLACDNARNVDCPYDLCKVPGY---ER-APSTTNCRG

YWICEAGKSVGHCCPPQHSYDATV---GCV--PDP--RCNDPCDEGN-------------

--------------------------------PLNVC------DKRGVNRQPLVFEQSVA

GQ-GWDRKSCSPGTAFNIKACACIFRTEIN------------------------------

---KTEIHCEPELYLPFNGDIKD-ESGNNNYLQVDGVK--VVDGTGYFDGKSLIRVPRFS

NMDFGSTLIIKLRYKEEGGGSA--------------------------------------

------------------------------------------------------------

-------GGQPQALVANGDC--------------NR-PSSLYMVTGQG---RAEMGLRTS

QGD-NVSVSIPS------------------------------------------------

------------------------------------------------------------

------------------------------------------------------------

-----------------------------------------NGAGWKEAVLIADDSVLQG

TVTGVSYQTIFT--GSIETSRCAMQIG----------RGTEFKHFTGYM-----------

-DDVTVYLCRPKGL----------------------------------------------

------------------------------------------------------------

------------------------------------------------------------

------------------------------------------------------------

------------------------------------------------------------

------------------

>Lsta_jg35514.t1 gene=jg35514 CDS=1-4107 protein length = 1368 strand = +

------------------------------------------------------------

------------------------------------------------------------

------------------------------------------------------------

------------------------------------------------------------

------------------------------------------------------------

------------------------------------------------------------

------------------------------------------------------------

------------------------------------------------------------

------------------------------------------------------------

------------------------------------------------------------

------------------------------------------------------------

------------------------------------------------------------

------------------------------------------------------------

------------------------------------------------------------

------------------------------------------------------------

----------------MYQTS-----V---------------------------------

---------------LISLLL--------SLIVHQSSTQERRCDVPADVV----------

-FLMDASDSIKDTEWEQEKNFVSVLIDNLEVER--YAIHVGVIVYSTDIGM-VIDLQPFK

TKAQLKSLLQQAVQINQGTDTAKAISKLIEMSDSQ--GRIEGDAKQIAVIITDGRSTDVS

ATIREARRARSQ---YKID-MIALGVG-----------NETFAEELEAIAGVEKNNRLFQ

VQN---FAQLHYVI-KQLEDIICEIVPTSTTTIATSPITTTT--AEPTTSP-FIPPFPCS

RPADVVFLIDGSYSITAADWIKGKQFVSY-LINSIDIGIDSIHVGIIVYGSS--------

-IGDVVSLTPFRSKA----QLK--------------------------QAASDLIQPPVG

RTNTALGL----------------------------------------QTVRDMFDTQAR

IGVPHIA-----------------IVITDGMSSNPSE---TRDQASRAKLGGVTLFVVGV

GNRVLRQEVE----------DMAS-----SLQT-----LF--------------------

------------------------------------------------------------

------------------------------------------------------------

-------------------------------------------------DA---------

------------------------------------------------------------

------PDFKYLVGMVELLRDNICSAI---------------------------------

------------------------------------------------------------

------------------------------------------------------------

----------------------------------------QE--------TT--------

------TTSSSSTTTTTARMTT----RPPVPELCLQCLVEGGVGFNPLPEDCEKYVLCFP

VGPTY--EPHIKSCPFGMFWSNESVSCLDARYVDCPWDKCAGPQKFETYSSTTDTANCRS

YMRCVNGRSTPTCCETGYRYKDGA---GCV--ADT--QCQDVCPLDVTI-----------

-------------------------------FNQGSC------RFRPDHDSYYSYLEVIP

GK-GNVKRNCDPGQVYSPALCACTYDVGKV------------------------------

---IPDVACLPAVNLTFDGSFED-SSTNALHIDVKKVG-LTDLGTAYFDGDGYITLPTTA

NLDLGDKFSIRLRYRLKTKTDPGQI-------LTNNEWGFQNWFKNASNQGANI------

--------------------------NIIT---GNKQVNLSDPGNISGAE----------

-----SKPLEVITITKNGTVLRDPRYDFTKNETWGNTGPSVVHVVQLG---K--------

----LGNITTPTIKNGIVYIPNGQGKLLIQESD----G-LTAGSIKHKWSVLSVNPDGKT

GKLEETGQGGVPQNIQQKFNFDLRSPVLERWFIMAP---GGIVLQKGEGPIPADVLNRYA

GFSIGTLVKVFDRGDRTERWQISKPDGSELQEGFGEIPKTLINDLKLTSALTPHQVTSTW

SVIAQNGEVLETGKGTIPSETVKTYSSDPTITVLKSTSRDGGAPVWKITRA---------

--NG---VEIVEGIGALPQGIAQFVKGSILLPQIATVNGWKVKLPNGDIRSGDGPIPQSI

LDLFTKHYSSPANKVA-------------D--TNNQVQTAGTNNPVVVSKWSVTLPDGT-

---VRSGFGEMPAELKNAIKSQGQRWEVTLGD-GTV---RSGEGNVPVD--LLNSPVGRG

TSASSTGSNQEKRWSLTMPDGTVKSGQGDVPLELQKRLQESGAKQQWEITLPDGKVQRGS

GPIPEHLRQFLGGRRRRRAAVHTVTSSDLLSNCGTDPSVTPSLHLVASDSDITMTVVTSQ

SPNGVTVSLPVHDAMNDVLFQYDGSHLSGIVNGYIRKLPLSGTVVKMPSPLTLGQCLFRD

RNHFIGQIDLFELHACLP

>Bgla_XP_013087207.1 PREDICTED: uncharacterized protein LOC106071610

------------------------------------------------------------

------------------------------------------------------------

------------------------------------------------------------

------------------------------------------------------------

------------------------------------------------------------

------------------------------------------------------------

------------------------------------------------------------

------------------------------------------------------------

------------------------------------------------------------

------------------------------------------------------------

------------------------------------------------------------

------------------------------------------------------------

------------------------------------------------------------

------------------------------------------------------------

------------------------------------------------------------

------------------------------------------------------------

------------------------------------------------------------

------------------------------------------------------------

------------------------------------------------------------

------------------------------------------------------------

-----MSKLNAQP-RQTFVFV-------------------------------FAASFLLA

STFSAVSA----ED---QA---YTGGL----------DEQSQQLLL--------------

--------------QFSQSQLDYIR-----------SLLGNA----LQGTTNQANEPPTN

EEQS-----------------------------------------EEVQEPQQQYEP---

--------P---------------------------------------------------

-------------------------------------AVLLPEYLPP----TQQQNQPS-

-STQQ---------PTQYQ-----PTQ-----------EPMD-----KESE---------

DDDQQEPQPYY------PPTQP----PTQSPQYQQTTQQSQQYQQTTQQPQYQAPQEQEE

DDDDSQ--E-AVQQPNVPEKD--------DSEETSYTTQPQVHQTPEVDDSEETSYTRQP

QVQQTPEVDDSEEAGQTPQ-----QVKQEKEEDDDDSD--EEH------CDGYKAYPENI

--------AYYLQFDGTNWFKR-----PCPLGLG------------FDA-D---KCAC-V

IILSDYKTSTYPTTTTVPTTTTVR------------------------------------

------------------------------------------------TTTTSPTTTTTT

-------TTTTTQPTTTTTTP--------------------------TTTTTTP-TTTTT

RPT-----TTTVTSTTTRTTTT----GKPSLDLCRDCVILHGVGYAPYPGYCDAYIQCQF

SGSV-PSSAIIRRCPTGLQWNQNKLTCDFPEIVQCKSVVAKCPRQ------KEIVGNKQE

YLSFNGYTWTQLRCPVNQVYSDLVC--GCTENWGG-YGNFETCADKRAIAGDKTGYLQLS

SL--GWIRMPCPKTLGYDAATCRCTDILETSDLYTNC-----PNTKPIPGDVTGYLQF-N

GV-SWIRMPCPASVGYDSRTCQCTYRLTVKGD----------------------------

----KEDKCEPSLALTFEDNSATDTSINQFWVNNTGVT--FRNGKAYFDGQSRLTVPGFS

NMDLGNTATDT-------------------------------------------------

------------------------------------------------------------

------------------------------------------------------------

------------------------------------------------------------

------------------------------------------------------------

------------------------------------------------------------

------------------------------------------------------------

------------------------------------------------------------

------------------------------------------------------------

------------------------------------------------------------

------------------------------------------------------------

------------------------------------------------------------

------------------------------------------------------------

------------------

>Pcan_XP_025084945.1 uncharacterized protein LOC112558610

------------------------------------------------------------

------------------------------------------------------------

------------------------------------------------------------

------------------------------------------------------------

------------------------------------------------------------

------------------------------------------------------------

------------------------------------------------------------

------------------------------------------------------------

------------------------------------------------------------

------------------------------------------------------------

------------------------------------------------------------

------------------------------------------------------------

------------------------------------------------------------

------------------------------------------------------------

------------------------------------------------------------

------------------------------------------------------------

------------------------------------------------------------

------------------------------------------------------------

---------------------------MKRPLRPPQQLQHLQRLLTQGLVSYYSISRYER

A--NEGKLVTSQDMCDGCELRHGVGYKPHPSDCTLYVQCQADKNGLPVVAGVRPCPHGLY

WNQDKLTCDYRH--NVNCVDD-------------------------------ICRAGAVR

KTKASAA-----------------------------------SCRGYWDCNS--------

-GTALAKCCPIN---YSYNPYIARC-----------TYNPTC----RDDCL---------

----TSTAP-----------FVAECP-NGMRPVPGD---------------RTKYEVKTG

NTWTLMSCPANLG--FSTLPCGCNVHLNTNVQQECVPELYLPFISDTQDQSGRQVFVKNE

GVQ----VRD----------GKAF-----F---DGKSRLTVPRFSNTWWGSTVTRRLALV

SNGDCQV----------------RPSLAVCAGPAGVE-----------------------

FYAETENSPQP------VNFT-------------V-STFDQGYGWQDGWQDVLYR-----

-----------------------------------------------LDA----GS-LY-

--------------------------GHVSLNRDSRV-----A------LANLEVRQRGL

VIGS-GGGCDDFNGFIDEVTVYLCR--PKEKDDD--------------------------

------------ESTTVEL---------------VD---------TVTTQSP-GSD--DD

DDD--------------------------------------------DD-----------

---------DTTTPVVTTFAPDS----------------RET--------DDSEE-----

-----NEETTRTTTTTAAPTVS----ANTVPNMCDGCELRHGVGYKPHPSDCTLYVQCQA

DKNGLPVVAGVRPCPHGLYWNQDKLTCDYRHNVNCVDDICRAGAVRKT---KASAASCRG

YWDCNSGTALAKCCPINYSYNPYIA--SCT--FNP--TCRDDCLTSTA------------

-------------------------------PFVAEC----PNGMRPVPGDRTKYEVK-T

GN-TWTLMSCPANLGFSTLPCGCNVHLNT-------------------------------

---NVQQECVPELYLPFISDTQD-QSGRQVFVKNEGVQ--VRDGKAFFDGKSRLTVPRFS

NTWWGSTVYVHLRYKSALTS----------------------------------------

------------------------------------------------------------

--------TRRLALVSNGDC--------------QV-RPSLAVCAGPA---GVEFYAETE

NSPQPVNFTVSTFDQG--------------------------------------------

------------------------------------------------------------

------------------------------------------------------------

---------------------------------------YGWQDGWQDVLYRLDAGSLYG

HVSLNRDSRVAL--GNLEVRQRGLVIG----------SGGGCDDFNGFI-----------

-DEVTVYLCRPELK----------------------------------------------

------------------------------------------------------------

------------------------------------------------------------

------------------------------------------------------------

------------------------------------------------------------

------------------

>Pcan_PVD33241.1 hypothetical protein C0Q70_04492

------------------------------------------------------------

------------------------------------------------------------

------------------------------------------------------------

------------------------------------------------------------

------------------------------------------------------------

------------------------------------------------------------

------------------------------------------------------------

------------------------------------------------------------

------------------------------------------------------------

------------------------------------------------------------

------------------------------------------------------------

------------------------------------------------------------

------------------------------------------------------------

------------------------------------------------------------

------------------------------------------------------------

------------------------------------------------------------

------------------------------------------------------------

------------------------------------------------------------

------------------------------------------------------------

------------------------------------------------------------

------------------------------------------------------------

------------------------------------------------------------

------------------------------------------------------------

------------------------------------------------------------

------------------------------------------------------------

-----------------------M-----M---EVRSPAPLVKFL-----------ILTL

SVTPCTS----------FT----YPPRASYPQTGGYG-----------------------

NKGTQLQQQMP------IGFLY----PNL-----L-KNLNSGYNWQALQQALQQR-----

-----------------------------------------------QYA----GQ-QQ-

------------------------------------------------------------

----------QQQQYLDRLLQTLYG--TSHTGQE--------------------------

-------EEAGQQTQTYED---------------IE---------DLQEAI---------

TSV--------------------------------------------LD-----------

---------AVYGHSSRTFAPDS---------------SNED--------DDSEE-----

-----NEETTTTTTTTAAPTAS----ANTGPNMCDGCELRHGVGYKPHPSDCTLYVQCQA

DKNGLPVVAGVRPCPHGLYWNQDKLTCDYRHNVNCVDDICRAGAVRKT---KASAASCRG

YWDCNSGTALAKCCPINYSYNPYIA--SCT--FNP--TCRDDCLTSTA------------

-------------------------------PFVAEC----PNGMRPVPGDRTKYEVK-T

GN-TWTLMSCPANLGFSTLPCGCNVHLNT-------------------------------

---NVQQECVPELYLPFISDTQD-QSGRQVFVKNEGVQ--VRDGKAFFDGKSRLTVPRFS

NTWWGSTVYVHLRYKSALTS----------------------------------------

------------------------------------------------------------

--------TRRLALVSNGDC--------------QV-RPSLAVCAGPA---GVEFYAETE

NSPQPVNFTVSTFDQG--------------------------------------------

------------------------------------------------------------

------------------------------------------------------------

---------------------------------------YGWQDGWQDVLYRLDAGSLYG

HVSLNRDSRVAL--GNLEVRQRGLVIG----------SGGGCDDFNGFI-----------

-DEVRIS-----------------------------------------------------

------------------------------------------------------------

------------------------------------------------------------

------------------------------------------------------------

------------------------------------------------------------

------------------

>Pmax_XP_033751871.1 uncharacterized protein LOC117335790

-MASYKRVAILLMS-F-M----CCLWLSAHAQLWWDDDEISLDDLVDIGFPIKRPRNDKY

RDASNIIHYKTIIDDDESEKAHDGGKGDEIGDDKDDDDTKFTDKADDNPAGTWND---GN

NDDNNITDKDDDILAKTGEDNDDIGNNNTENAGENDDTQADSGNDEFNDDDKITDIVNDI

LAK----------TGEDNDDIGNNN------TENAGENDDTQADSGNDEFDDDDKITDIV

NDILAMTGE--------------------DNDDFGYNNIENAGENDDTQA-DSGNDENND

DDK---MPDKDDDTPANIGDDDNDDDKTKNTGRD-DDNPADETS--GD---NGDDENTEV

TGKEIGNDDNANTGITGGDED-----NVDGGDGSNN-AENT-GKDDDTQADSGD--DENN

DDNKLTDKDNDTPANIGSDGDDDKTKNTGKANENSAEIGNGDNDNTESTGGDDDDQVKTG

D----DNIDGGGSNND-----ENTGKDDDTPAETSDDNTSDDGGDSGKGDDSPTKSGDNG

NGNT-----ESMRKDDDKPKDSSLDQTTQNVVTSLEKPE---------ENDDSTEDRVPK

SAS--PSEDSDDSSE-------------SGESND----S--STQVPSSSSSSSSSSSSS-

-SSSSSS-SSSSSSSSSSSSSSENSDDS------TERTTPMRFIPRRTS-----------

-------------------------------------------PKTTPTPIPPKGSS--E

ETSKVTTPTPLPPKGSSE----ETEETSKVTTPTPLPPKGS-S------EET--------

-----EETS--------KV----------TTPTPLPPKGSSEETEETSKVTTP-------

-TPLPPKGSSEETEET----------SK------------------VTTPTPLPPKGSSE

EPE-----ETSPSTTQIPLPPEKSTT-PMTFTP----SPLFRCPLVMDIV----------

-IVIHGSNTITRQNFTDIKCGVVSLIESMTPPIYPRNSHIGFIQYSDAKHVEKHNISA--

DKEGLKVFVYETKTSGTGTRTDLGLLEMNRMFASN----FRPGVRRIGVIVTYGLSPSPV

NVAQQAFL-ARR---AGID-IFVVGIT-----------SNTREYELNVMTGTVGRHHVYY

LPR---FDEFKDFVNDRLVHE-------------------------------ICAAPALP

T-----------------------------------------------------------

------------------PRPTPRL-----------TPKPTPRPTPRPTPLL---TPKPT

PQPTTR--P-----------YPKLTPKPTPRPTPRPTPKSTPR-----------------

-------PTPRPTPK---------LTPKPTPR--PTPRPTPKPTPRPTPRPTPKLTPKPT

P---------------------------------RPTPAPTPKLTPK----------PTP

RPTPRPTPKLTPRPTPKLTP---KPT----P---RPTPRPTP--------K---------

LTPRPTPRPTP------RPTPK----LT---------------------PKPTPRPTPR-

-------------------------------------------PTPKITPKPPTKR-PT-

--------------------------PKPTPRPTPRPT------------------PRP-

TPKL-TPKP-------TPRPTPRPT--PKPTPRPT-----------PRP-T---LRPTPR

LTPK-PTPRPTPRPTTRPTPKLTPRPTPRPTPRPTPK------LTPKPTPRP-TPR--PT

PKLTPRPTPRPTPRPTP-------RPTP--------RPTPRPTPNPTPRPTPRPTPIPTL

RPTLRPIPYPTPKPRPTTQKP-----TIPPMWTWFWRR-KTT-------TTPTPKPT-TT

VPTT-TTRRFTTTTG---ELET----PPPGFNPCLGCKMVNGAGFNPHPTDCSKFVQCFF

RGDDV--IAFYKNCPVGHFWNQEKLTCDYAFRVNCVHDMCHNLLV---HR-YANRGHCQA

FWDCENGHAIQRCCPAGQAYNTSVA--MCV--KDP--SCPPTCSWSA-------------

----------------------------HAPPVKTVC------KDRAVPGNSAFYEQEVE

GH-GWLMMPCAPGTAFDQKECRCSEFSA--------------------------------

SLNDHDSDCTAEVILNFDNDVQD-SSGKYVWVTNKGVE--VKNGSAVFDGKSELLIQRFT

NDDFGFTFIVRMRYRETGSTP---------------------------------------

------------------------------------------------------------

--------YSNSALISNGDC--------------GR-DGSIVIATDPV---SIRFGVDTD

KTRGLYSFRVKK------------------------------------------------

------------------------------------------------------------

------------------------------------------------------------

-----------------------------------------PATEWKTVEFKLADGKLQG

RANELVFSRNVP--GRIERRACAIQIG----------HGWGLENFVGDI-----------

-DQLEIYKCKPMKGYTFHKYKG--------------------------------------

------------------------------------------------------------

------------------------------------------------------------

------------------------------------------------------------

------------------------------------------------------------

------------------

>Cgig_XP_011422884.2 uncharacterized protein LOC105325156

-MDLHTSLPFVSIIFM-L----CCGFVHCDTEDATENV----ESVM-THAPIVS------

--DSPVGQYQTTV-----------G-GGEYTDYQEDD-----------------------

----NMYLFGDYFNKQDTDDN--------QNAAQADDR---SATQSFNVADSRT-ISNNV

VQVTESPNFI--EDSEEAYDLGAYD-----------------------------------

-------------------------------KDYGTEA---------TQM------EANE

GSR---VAE----------------------G--------AQSD--GA---GGDSTETSG

SGSSSSSGETKQ--SGGGDSNNGNSKNSDSKSSSENSGSSSSGTDDNG--SGGNGKDDGN

D---IVDV--ETYHNLQGSASS---EDSSYSDESSGE------------SGTKKKNCKCK

K----N-KNGKGG---------DFGSDDS-SDDSSEDQSSSSGSGSGNRDKDKSREGNYG

DQGDSSSDESN---------YDSY------------------------------------

-----DSEDHESSSESREEPDKPDIPDYDKQR----------------------------

------------------TGNGLDRNRN------EYTTSIHRFFTSKT------------

------------------------------------------------------------

----------------NTLFNVVNR-LTSNTPNTPSSPGGG-D------VSL--------

-----RTNYR-------LS----------YQPKSTPS-----------------------

-----------TTTAKSYIIN-----L---------------------TTTPPPLGGDP-

-PNVKDI--------------TDQSV-PKWFRDYIPKREYVEQPIARDYILTPRTSFWEP

PDVAKPPDWQEQKEWINIKINLEKPADWIDDPNSNGSIPHWNIPYIPPDWFKKN------

NEKLTTEFHFELTTPSPKTVR-----------WTP----KPEGVDRTTHV----------

----PEYLAKK-------------------------------------------------

--------------------------------------------------------RVCD

RKFDIVLAIDGTGN--EKDFGYLKGAIVQ-LLDRLIMGEDKVKVGVVLLGNT--------

-DG-------LQIPIS-GNR-VELE-----------D------------QVARLGLPED-

----SNRYD-----------IALKSA-----------G--------------ELLEREGR

E----------------GIPKV-AIMIVNGKS--RYQYHT-RMEAARLHRSGISVFTVGV

GYNTDEEEL---------------------------------------------------

------------------------------------------------------------

------------------------------------------------------------

------------------------------------------------------------

-------------------------------KTISSSQ---NE--------VIRV-----

------KNYIHLVYLMTGYVQLFCN-----------------------------------

-------------------------------------------------------V--ED

NYLT-----PPTLAP---------------------------------------------

-------------P-H---------------------------------QTATPKAK---

-----I---IAKLTTDVMSEPS----PREAKVLCEGCKMINGAGFNSHPNECDLFVHCYF

GELGL--RAIIRKCPFGQFWNQTILSCEYSERAYCPMDRCAYIKD---RD-YEASENCRA

YWECSNGHSRGKCCRYGYRYVRGE---GCALDSDN--ICKESCPMEMPTDLHS-------

----------------------------YNNAPAQSC------DKIPIANDRSHYQQLLP

GT-GYVTMPCAEGTHYNERKCTCTDQEPSYPT------------------------FKEP

LVQEPPAGCRPEVKLDFKNGVTD-SSGKWTYVNNQGVL--IQNGEAIFNGDSRLLIPRFT

NVEFGKTFVIRLRYKEEEKLKF--------------------------------------

------------------------------------------------------------

--------NESQALVNNGDC--------------GD-FGSIQIFTKRN---SIGYVVKTT

KEPSHVSLQIHK------------------------------------------------

------------------------------------------------------------

------------------------------------------------------------

-----------------------------------------PHGEWKDVEYIVSDGKFEG

YLNGVQATKWSM--GSVESRQCAVQIG----------FGHGYNNFRGRL-----------

-SLLEIYFCKPEKAMKYS------------------------------------------

------------------------------------------------------------

------------------------------------------------------------

------------------------------------------------------------

------------------------------------------------------------

------------------

>Myes_XP_021377426.1 mucin-17-like

MASCHATVVFLVMS-F-V----CRLWVTAHAQLWWDDDEISLDDLVDTGFPIKRPRYDSH

RDASNIVHYKTINDDYESEKMHVVNDNADVTSNGDDSTTE--------------------

----TMVDKTTPFLQIDSSDNDRVSYGLSQQV---------SSNHVISKPDSIISTKNTV

SQT----------DSKETDESGDSNKLKQNPTVNNKESQDTQK------------STASK

NTVSETDSK--------------------EKDDHT---------DPNTQT-KTGNSEGNK

NNL---ESTAS-----------------KN--------TVSETD--SK---ENDDHTDPN

T--------QTKTGNSEGNKN-----NLESTASKNT-VSQSDSKERDDPADPNTHTQTEN

AEGKTETLKSADSKNIVSQTDSNETDDPADPYEH---------------SQTENEVVK--

-------IETRKSSAS-----KK---TVSQP----DSKEKDDSAD----PNTHTQTGNTG

GSKEHKVSEYVPPQPDSKEADDSFEHTTPTTVTSLDKPE---------EKDDSSQERVPK

SAN--PSEDSEDSSE----NSSPSAPDNSGESDD----S--STQVPSSSSSSSSSSSSS-

-SSSSSS---SSSSSSSSSESSDDSGIA------TKRTTPVYFVPRRTP-----------

-------------------------------------------KTS--------------

------------------------------------------------------------

------------------------------------------------------------

-------------EKP----------LQ------------------RTTPTPIPSRGSSE

ETE-----ETSPGSTPTPVPLTRGTS-PMTFTS----HPIFRCPLVMDII----------

-VVIHGSNTITPLNFTDIKCGVINFIESMTSPRYPANTHIGFIQYSDATNVEKRTISA--

DKDGLKVFIYETKTTGVGTRTDLGLLEMNRMFASN----FRPGVRRVGLIVTYGLSPSPV

SVAQQAFL-ARR---EGID-MFAVGIT-----------GNTRMYELNVMTGAIGRHHVYY

MSR---FDEFKDFVRDRLVYD-------------------------------VCVAPTMT

SPQPITHP----------------------------------------------------

-----------------TPRPTPRQ-----------TPRPTMRPTPRPTPRL---TPRPT

PRQTPM--P-----------IPLFTPRPTPRPTRRPTPRPIPRQTP---RP---------

IPWLTLRPTPRPTPI---------LTPKPTPR--YTPRPTPRPTPLQTPRPTPRLTPKPT

P---------------------------------RPTPLKTPRPTPG----------PTP

SHTPKPTPRLTPRPTPHPTP---RPTPKPTP---RYTPRPMP--------L---------

LTPRPTPHPTP------RPTPK----PTL-----R--------HT----PRPTPRPTPK-

-------------------------------------------PTPRLTPRPS-PY-PT-

--------------------------PRPTPKQTPRPTPRPTP------IQALRPASQP-

TPRL-TPRP-------TPRPTPRQT--PRPTPRPT-----------PRP-T---PRQTP-

----------------RPTPRPTPRPTPRPTPRPTPR------QTPRPTPRP-TPR--PT

PRQTPRPTQRPTPRPTP-------RPTP--------RLTPRPTPRPTPRPTPKPTPRPTP

RPTPRPTPRPTPRP---TPRP-----TKPPLWTWFWKT-RGTTKRPPITTTPTPKTT-TT

LPAT-TTRRFTTTTG---VLET----PPPGFNPCLGCKMVNGAGFNPHPTDCSKYVQCFF

TGKNV--TAFYKNCPVGQFWSQEELTCDYAVRVNCIHDMCHNLLV---HR-YANNAHCQA

YWFCEDGHAIQRCCPPGYAYKAS-D--GCV--RDR--TCPPTCTWSY-------------

-----------------------------TPPKKTVC------QDKALPGNAAFYQRNVE

GH-GLMTLPCAPGTNFDIKECRCSEFSA--------------------------------

SLSNKDTDCKAEVILDFDKDVQD-TSGNYLWVTNKGVK--VENGKAIFDGKSELLIQRFT

NDDFGHTFTVRIRYREKEGSP---------------------------------------

------------------------------------------------------------

--------HSNSALISNGDC--------------GR-DGSIVIATDPI---GIRFGVDTD

RTRDMYSFRIKK------------------------------------------------

------------------------------------------------------------

------------------------------------------------------------

-----------------------------------------PKTDWKMVELKLADGKLQG

RANERVFTRTVP--GLIERRACAIQIG----------HGWGLNNFIGEM-----------

-DNLEIYKCKPMSVYRP-------------------------------------------

------------------------------------------------------------

------------------------------------------------------------

------------------------------------------------------------

------------------------------------------------------------

------------------

>Cgig_XP_011456399.2 protein PIF isoform X2

------------------------------------------------------------

------------------------------------------------------------

------------------------------------------------------------

------------------------------------------------------------

------------------------------------------------------------

------------------------------------------------------------

------------------------------------------------------------

------------------------------------------------------------

------------------------------------------------------------

------------------------------------------------------------

------------------------------------------------------------

------------------------------------------------------------

------------------------------------------------------------

-------------------MNIVLLVFCLIGFCRSIPNPGGIR------VQLP----DSP

ITINMEPQKI-------NVESPVSRIKPPKARFEAPKTGLEPVRA---RLEPP---KA--

-RMEPPKARIEPPKARIE--S-----TK--------------------ARIELPKA----

---RIESRIESPNSVVAGIDSSRSKLEQAKTTFESKQ----GCRNLLDVV----------

-AVVDGSDSITSPDFQTLKSSLVDLMDGLQLA--EDQARFGVVLYSSDVA-AEIPLSA--

DRRQLKSKIMGLRHPRDGTRTDLGIKSLRKMF-SQ---QGRPGVPRVGVVITDGISKNPR

DTAKESELARS----EGVK-LYAVGVS-----------DLIAENELKSIASNG--TRVLS

VTS---FDQLKLVF-SSLVVQ-------------------------------VCPTTTTT

T-----------------------------------------------------------

------------------------------------------------------------

------------------------------------------------------------

------------------------------------------------------------

------------------------------------------------------------

------------------------------------------------------------

------------------------------------------------------------

------------------------------------------------------------

------------------------------------------------------------

------------------------------------------------------------

------------TTTTPAPT----------------------------------------

------------T-----------------------------------------------

------------------------------------------------------------

---------TTTTKTTTTLPPT----TKKPKNPCDSCKMSNGAGFTRHPTDCSKFIQCYF

GNNGL-KKMSYQECPWGNFWDQSSLTCQPAHRVKCPTDRCLDPEVLT----YDLPGSCRS

FWACDGGESIPMCCPYGTSYHS-GI--GCL--PDN--KCKDPCPPRPTQHLNPRAIM---

SDKIDSKKNAPLREMSLP----GLKPKLNIKPRKPVC------DKKAVRGDSNSFEQFVE

RY-GWIKMPCAPGTQYSQADCECTTTVPYSS-----------------------------

---NKTAECTSKLKLNFSDGFEDERDKRPVYIVNNNVS--FSGGVAKFSGKSRLRVPQLS

NVDYGDSVMLRIRFRDSTN-----------------------------------------

------------------------------------------------------------

------SSGRPQALISNADC--------------GN-NASILIAKDKD---RIIFGAHTE

NGG-YNQIELP-------------------------------------------------

------------------------------------------------------------

------------------------------------------------------------

----------------------------------------KPKTEWRNVKYSYNLGRLQG

SVNTDKVTRWIPGGGKIQSRPCALQIG----------HGENLDDFEGDI-----------

-DDLEIFTQCMPPDDMYFDEDYGGGFL---------------------------------

------------------------------------------------------------

------------------------------------------------------------

------------------------------------------------------------

------------------------------------------------------------

------------------

>Cgig_XP_011456398.2 protein PIF isoform X1

------------------------------------------------------------

------------------------------------------------------------

------------------------------------------------------------

------------------------------------------------------------

------------------------------------------------------------

------------------------------------------------------------

------------------------------------------------------------

------------------------------------------------------------

------------------------------------------------------------

------------------------------------------------------------

------------------------------------------------------------

------------------------------------------------------------

------------------------------------------------------------

-------------------MNIVLLVFCLIGFCRSIPNPGGIR------VQLP----DSP

ITINMEPQKI-------NVESPVSRIKPPKARFEAPKTGLEPVRA---RLEPP---KA--

-RMEPPKARIEPPKARIE--S-----TK--------------------ARIELPKA----

---RIESRIESPNSVVAGIDSSRSKLEQAKTTFESKQAINPGCRNLLDVV----------

-AVVDGSDSITSPDFQTLKSSLVDLMDGLQLA--EDQARFGVVLYSSDVA-AEIPLSA--

DRRQLKSKIMGLRHPRDGTRTDLGIKSLRKMF-SQ---QGRPGVPRVGVVITDGISKNPR

DTAKESELARS----EGVK-LYAVGVS-----------DLIAENELKSIASNG--TRVLS

VTS---FDQLKLVF-SSLVVQ-------------------------------VCPTTTTT

T-----------------------------------------------------------

------------------------------------------------------------

------------------------------------------------------------

------------------------------------------------------------

------------------------------------------------------------

------------------------------------------------------------

------------------------------------------------------------

------------------------------------------------------------

------------------------------------------------------------

------------------------------------------------------------

------------TTTTPAPT----------------------------------------

------------T-----------------------------------------------

------------------------------------------------------------

---------TTTTKTTTTLPPT----TKKPKNPCDSCKMSNGAGFTRHPTDCSKFIQCYF

GNNGL-KKMSYQECPWGNFWDQSSLTCQPAHRVKCPTDRCLDPEVLT----YDLPGSCRS

FWACDGGESIPMCCPYGTSYHS-GI--GCL--PDN--KCKDPCPPRPTQHLNPRAIM---

SDKIDSKKNAPLREMSLP----GLKPKLNIKPRKPVC------DKKAVRGDSNSFEQFVE

RY-GWIKMPCAPGTQYSQADCECTTTVPYSS-----------------------------

---NKTAECTSKLKLNFSDGFEDERDKRPVYIVNNNVS--FSGGVAKFSGKSRLRVPQLS

NVDYGDSVMLRIRFRDSTN-----------------------------------------

------------------------------------------------------------

------SSGRPQALISNADC--------------GN-NASILIAKDKD---RIIFGAHTE

NGG-YNQIELP-------------------------------------------------

------------------------------------------------------------

------------------------------------------------------------

----------------------------------------KPKTEWRNVKYSYNLGRLQG

SVNTDKVTRWIPGGGKIQSRPCALQIG----------HGENLDDFEGDI-----------

-DDLEIFTQCMPPDDMYFDEDYGGGFL---------------------------------

------------------------------------------------------------

------------------------------------------------------------

------------------------------------------------------------

------------------------------------------------------------

------------------

>Lgig_XP_009066027.1 hypothetical protein LOTGIDRAFT_236718

------------------------------------------------------------

------------------------------------------------------------

------------------------------------------------------------

------------------------------------------------------------

------------------------------------------------------------

------------------------------------------------------------

------------------------------------------------------------

------------------------------------------------------------

------------------------------------------------------------

------------------------------------------------------------

------------------------------------------------------------

------------------------------------------------------------

------------------------------------------------------------

------------------------------------------------------------

------------------------------------------------------------

------------------------------------------------------------

------------------------------------------------------------

------------------------------------------------------------

------------------------------------------------------------

------------------------------------------------------------

------------------------------------------------------------

------------------------------------------------------------

------------------------------------------------------------

------------------------------------------------------------

------------------------------------------------------------

------------------------------------------------------------

------------------------------------------------------------

------------------------------------------------------------

------------------------------------------------------------

------------------------------------------------------------

------------------------------------------------------------

------------------------------------------------------------

------------------------------------------------------------

------------------------------------------------------------

--------------------------------------MRNGVGFNSVAGSCQKFIQCIF

NLEML-ISTILKDCPAGLFWDQDKLTCNYASEVDCTEDPCYSKPDGD-I---AHPTNCRE

YYTCFNSVSFEKCCLPGYAFNAAAG--KCE--SNS--ACVDSCKWGN-------------

--------------------------------PEEGC------TRREIADKH-SYEQ-AV

GD-EWIVMSCPLGALYSQEECKCGIYDE-DYV------------------------NTPP

PPAARDAECKPSVQLSFDSGTYD-ESGNFNYVQNNGVV--VESGVAYFDGQSFLRIPRFA

NVDFRKTVTIKMKYKLDGAA----------------------------------------

------------------------------------------------------------

--------TGQEALVTNGDC--------------GE-KQSIYIVAEQS---QTVLGLISA

DSA-DEKSATIA------------------------------------------------

------------------------------------------------------------

------------------------------------------------------------

-----------------------------------------SSSDWNEVEYKVVDGELIS

AVNGRQANTIVD--GIIKRKHCSLQIG----------RGDGFDNFKGWI-----------

-DELSVYLCAA-------------------------------------------------

------------------------------------------------------------

------------------------------------------------------------

------------------------------------------------------------

------------------------------------------------------------

------------------

>Pfuc_AYN73061.1 VWA and chitin binding domain-containing protein 1

------------------------------------------------------------

------------------------------------------------------------

------------------------------------------------------------

------------------------------------------------------------

------------------------------------------------------------

------------------------------------------------------------

------------------------------------------------------------

------------------------------------------------------------

------------------------------------------------------------

------------------------------------------------------------

------------------------------------------------------------

------------------------------------------------------------

------------------------------------------------------------

--------------MDYKMLFLVTLIFCLL------------------------------

------------------------------------------------------------

------------------------------------------------------------

-----DISISAPP----------------GGIKVHFPATSDGCRDLLDVI----------

-VVVDGSDSITSKDYVTLKNSLVDLMDHLTLA--EDQARLGAVLYSSDVA-NTLPLSA--

DRSWLRKSLLSLRHPRDGTRTDLGIKAMREMF-KT---QGRPDVPRVGVVVTDGMSKDPT

QTARQAKLARD----EGVR-LYAVGVS-----------RFIDEDELLSIASNG--TTVYS

ASS---FDQLKLVF-ESLVVQ-------------------------------VCPTTTTT

T-----------------------------------------------------------

------------------------------------------------------------

------------------------------------------------------------

------------------------------------------------------------

------------------------------------------------------------

------------------------------------------------------------

------------------------------------------------------------

------------------------------------------------------------

------------------------------------------------------------

------------------------------------------------------------

------------TTTTTTTT----------------------------------------

------------TTP---------------------------------------AP----

------------------------------------------------------------

--T-----TTTTTTTTTTVPTT----TPKPKNPCDQCKMSNGAGFTRHPTDCDKFVQCYF

GNNGL-KKMVFQQCPWGNFWEQSSLTCKPAHRVQCPTDKCRDPEVLT----YELPGSCRS

FWACDQGDSIPMCCPEGTMYAE-GI--GCL--PDE--NCKDPCPPRPSGYQSPRTKKKLL

SQLSKDKKEAVLKSLGAP----VS----PSKVIPKVC------DKKPVFDDPSHFEQFVD

RF-GWVKMPCAPGTAYDTKDCECTVRAKYTA-----------------------------

---TKEQSCKAKVKLNFTEGFEDERDKRPVYVVNNNVT--FGQGVAKFDGTSRLRVPQLS

NVDYGEAVVLKIKFRETRD-----------------------------------------

------------------------------------------------------------

------TSNKAQAIISNGDC--------------GN-NASILVAKDAE---KITFGAETV

GGE-YTQVEIP-------------------------------------------------

------------------------------------------------------------

------------------------------------------------------------

----------------------------------------KPKTEWKTVKYAYNDGRLQG

TVNGHSKSAWIPSGGRIQARPCALQIG----------HGEGLGDFQGDI-----------

-EDIEIFTQCFPPEEYIEDY-F--------------------------------------

------------------------------------------------------------

------------------------------------------------------------

------------------------------------------------------------

------------------------------------------------------------

------------------

>Lgig_XP_009056819.1 hypothetical protein LOTGIDRAFT_239574

------------------------------------------------------------

------------------------------------------------------------

------------------------------------------------------------

------------------------------------------------------------

------------------------------------------------------------

------------------------------------------------------------

------------------------------------------------------------

------------------------------------------------------------

------------------------------------------------------------

------------------------------------------------------------

------------------------------------------------------------

------------------------------------------------------------

------------------------------------------------------------

------------------------------------------------------------

------------------------------------------------------------

------------------------------------------------------------

------------------------------------------------------------

------------------------------------------------------------

------------------------------------------------------------

--------------------MFSA------------------------------------

---------------TILSLL-------------------------------FCTQFVLC

DET---------------QGTQWDN------LMNKKFGDGQVPQHWNWKPTF--------

-GGKGSE-----------NQLVQTL-----------V-----------------LHPDDG

REATPTVLA-----------INSVINTEDEDKSRGD------------MLRHALFDL---

------ATLPVSKPD---------IPLLDSPHI-EIPLLDLQ-----------NL-----

---IVDQNDG----------GSKQ-----SVSDDKKTQQPTEDIKTEQTASDDSQ--TKQ

SSSDETVEKQTIVVPTIYQPQTAVPEVLR-------------------------------

-------SLAA------IPTI-------------Q------QLKT--VEPITQQPITEE-

-------------------------------------------PTTQQPPQPITEY-LI-

----------------------------------SKA----------------------A

RNTL-GSKPYRLLPDSDVVVANEANGQPSVMVAVG-----------NIPTI---QLPGSG

IIIARPDSWTPTTTTVAPTTT----------------------------T----------

-----------TVAP--------------------------TT----T--TSQPAP----

--------TTTVALTTTTVAPTTTTTTVA-----------------PITTTTKA--ATTT

TPTT-KVAPTTTTTTTQKPVPY----KNVIDGECNRCIYRNGVGFLSHTSDCTKFFKCQR

LSNGG-FRAAELQCPFGLYWNNEIFSCDYPRNTNCTNHPCTNTNT---RY-AEMTGHCAG

YWRCDWATPVAYCCPQGHRFQQSSQ--LCEVDRTR--TCRDDCSGPQQ----K-------

----------------------------PSGVVEKLC------DKRSVSDSKTAFEQRVP

VN-RWIRLDCAPSTGFNPKTCLCSDRVNAPV-----------------------------

TQKTSNKDCDPMLHISFNNGVRD-ESRHRFWIENVGVT--SKAGVGLFNGNNKLLVNRFA

NAPLGRDLVIEVVYEPADR-----------------------------------------

------------------------------------------------------------

--------RRDEVLVSNGDC--------------GV-KPSLYIVTGPT---GVTFSVKTT

RSSTPSVVTVPI------------------------------------------------

------------------------------------------------------------

------------------------------------------------------------

----------------------------------------SMPKGLIKARLSLANGRLTG

EVGGLSKSTPAS--GSVELRKSSLIIG----------SGDGMKKFDGIM-----------

-DDVKMFFCQNDGK----------------------------------------------

------------------------------------------------------------

------------------------------------------------------------

------------------------------------------------------------

------------------------------------------------------------

------------------

>Lgig_XP_009066028.1 hypothetical protein LOTGIDRAFT_236719

------------------------------------------------------------

------------------------------------------------------------

------------------------------------------------------------

------------------------------------------------------------

------------------------------------------------------------

------------------------------------------------------------

------------------------------------------------------------

------------------------------------------------------------

------------------------------------------------------------

-------------------MNTSL-----FIILALVVPG---------ILS---------

-------QDPENQC---------------KELLD----V--VAVVDGSDSISY-------

-----KN-FDILKQFLKTLVNDLNIGVG------ETRLGIILYS----------------

------------------------------------------------------------

--SDITITIPFNEDKDYLLYQISILEHARDGTNTALAI-----------AEM--------

-----NQMI--------DV----------YGRDGVPKVGI--------------------

---VITDGISKDPNATVYQAS-----LA------------------HQRNIDLFAVGVTE

KVNLVELQGLASSDSKVFMVSKFSNL-NTILRKVVLRKCPDQCKDYLEIV----------

-ALVDGSDSISRDDFVKLKLALASLVEDIDVS--ENKTRFGLILYSSDIT-EVISLSH--

DKKYLLDQIALLPHPRDGTKTDLGLARMNQLFREE----ARAGVQKIGLVITDGISKDRN

ATQKEAYALKTD---LNVN-MFTVGIS-----------DNIDVTELSEIATLN--RNILT

IDI---FDDLQHIL-ENVVRM-------------------------------FCPLPTCT

SALDVLTVVDGSDSISAEEFKELKDTLKN-LVLDLDIGQSGARFGLILYSSN--------

-IT--------AVITFTNNKNLILS-----------E-------------IDSLDQPRDG

TET-ALAIQ----------------------------------------EMIKMFTKLGR

SSFQRIG-----------------IVITDGMS--KDPSQTKQ-IAQEAKREGINMFAVGV

GNRIHQQEL------------KDI-----A----SNNQVFISDSFLE-----LSRSLMDV

VRKACPDPNLTTSPTVIYSTDTVTPTIKISSSTTGSTSGPSTTIASTSS-T---------

ASGSTTESTAS------PPSSL----SPV-----ISSSTTSVYTTSESSTSPSQSTTSP-

-------------------------------------------SQSTTSPSPSTTS----

----------------------------LSESTTSQSQ---ST------IIPFRVRLDPQ

RVRL-DPQRVRLIYYTTSLSQSTES--PSQSTASP-----------SDY-T---TSPSQS

IVSQSQSTTSPPQSTTSPS---------QSTASLSQS------TSPSQSTSS-LSQ--ST

SSLS-----QSTTSP-----------SQ--------STTSPSQSTTTLSQSTSPSQSTSS

------LSQSTTSPLQSTTSP-----SQSTMSLSQSTSSLSQSTTSTLQSTTSPSQSTTS

LSQSTTSRPGSTTLNIIRISAT----PSFPSYVCDGCLYKNGVYLKDHPTDCNKFLQCNQ

KYDGS-YDVIVKDCPQGLFWDQDLLLCNYPENTNCTKDPCYTLADFS-TY-SDIENNCRQ

YFKCVNGISYLECCESGYGFDKVTR--MCS--PST--TCTEACPSAY-------------

--------------------------------VMDVC------DKRAVVGNALVFEQKIP

SF-GWIRMNCALGTAFNETACYCSSFVDVGYL------------------------IPVS

SGPTPSNVCTPELYLPFDNNTRD-QSGNGFYVQNNGVV--VEDGVAYFDGNSSLRIPRFT

NVDFGTKLRITFKYKVDGIR----------------------------------------

------------------------------------------------------------

--------STPQALVTNSDC--------------GE-EGSLYIVSDPD---QVTFSTRTD

NLNSPLPALTTL------------------------------------------------

------------------------------------------------------------

------------------------------------------------------------

-----------------------------------------SSSEWNEVEFKYDNGVFTG

TVNGASNTQIIM--GSIKRNHYALQIG----------RGDQFSNFKGWI-----------

-DEVYVYMC---------------------------------------------------

------------------------------------------------------------

------------------------------------------------------------

------------------------------------------------------------

------------------------------------------------------------

------------------

>Pmax_XP_033752064.1 protein PIF-like

------------------------------------------------------------

------------------------------------------------------------

------------------------------------------------------------

------------------------------------------------------------

------------------------------------------------------------

------------------------------------------------------------

------------------------------------------------------------

------------------------------------------------------------

------------------------------------------------------------

------------------------------------------------------------

------------------------------------------------------------

------------------------------------------------------------

------------------------------------------------------------

------------------------------------------------------------

------------------------------------------------------------

----------------------------------------------------MTAME---

-------------NAVILVMIVFL------SLQMIVSADHSKCRRLLDFI----------

-CVVDGSDSINEENFQTLREVVEEMVDEFKIS--PGEVRMGIVVFSTSIAM-IQNLTS--

DRVKLKIEIGRLPHPQEGTNTARGIKMMHELFTSDPLREKRPDIPAVGMVLTDGRSKSMT

KTLRQARR-ARN---DGIE-MFAVGIS-----------DLVNKTELNGIASRE--ENVLT

VKS---FAQLKDSI-DRLVKM-------------------------------VCPTPTTT

TTTPTTTT----------------------------------------------------

---------------------T--------------------------------------

------------------------------------------------------------

------------------------------------------------------------

------------------------------------------------------------

-----------------------TP-----------------------------------

--------------------------------------------------------T---

------------------------------------------------------------

--------------------------------T---------------------------

------------------------------------------------------------

-------T------T----------------T----------------------------

-------------TP---------------------------T------------T--TT

---------------------TPTTTKQPTFVKSAFRKQLKI------------------

---V-NIRKMSIKQKKEQSIKA----PGWVKGPCDDCSMMNGVGYNPHPKICSKFTQCYF

GPHGN-MRAAYRECPFGFFFDTNVLSCRLSFQSHCNNDKCMLRPKLKSYP-YKGIQNCRA

YWLCKNGRSAARCCDKGFRYRRWGGKHKCV--KDP--GCQDECPPTI-------------

-------------------------------TSISIC------ETRLVFGNPKIYEQFIK

GWNMWIPRPCAPGSQYNADKCACTGHTTHV------------------------------

---PDKRTCKPEVYLPFTKNLKD-QSGSHSYVQNYNVR-QTKEGYAYFNGRSKLIIPRYS

NAEFKD-LVIKIRFKAKMSHTTL-------------------------------------

------------------------------------------------------------

-----AMRRKLTALVSNSDC------------C-ND-DVSLLMILGKS---SLHYMAISQ

NRK-MSKF-ILP------------------------------------------------

------------------------------------------------------------

------------------------------------------------------------

-----------------------------------------VCDDWNTAYFVHDTKTLYG

RCNSKEKTRPLR--GNIKRTHTGIHIG----------YGRGFNSFKGYI-----------

-DEIKIYRCQPDLDEI--------------------------------------------

------------------------------------------------------------

------------------------------------------------------------

------------------------------------------------------------

------------------------------------------------------------

------------------

>Cvir_XP_022318425.1 LOW QUALITY PROTEIN: protein PIF-like

------------------------------------------------------------

------------------------------------------------------------

------------------------------------------------------------

------------------------------------------------------------

------------------------------------------------------------

------------------------------------------------------------

------------------------------------------------------------

------------------------------------------------------------

------------------------------------------------------------

------------------------------------------------------------

------------------------------------------------------------

------------------------------------------------------------

------------------------------------------------------------

------------------------------------------------------------

------------------------------------------------------------

------------------------------------------------------------

------------------------------------------------------------

-------------------------MDGLHLA--EDQARFGVVLFSTDVA-XEISLSA--

DRQQLRTRIMALQHPRDGTRTDLGNKSLRRMF-TE---QGRPGVPRVGVIITDGISKDPK

ATAKESELARS----EGIK-LYAVGVS-----------DLIAENELKSIASNG--TKVLS

VTS---FDQLKLVF-SSLVVQ-------------------------------VCPTTTST

T-----------------------------------------------------------

------------------------------------------------------------

------------------------------------------------------------

------------------------------------------------------------

------------------------------------------------------------

------------------------------------------------------------

------------------------------------------------------------

------------------------------------------------------------

------------------------------------------------------------

------------------------------------------------------------

------------TTTTTTTT----------------------------------------

------------TT-----------------------------------------T----

------------------------------------------------------------

--T-----TPPPTTTTTTLPPS----TKNQKNPCDSCKMSNGAGFTRHPTDCSKFIQCYF

GNNGL-KKMSYQECXWGNFWDQTSLTCQPAHRVKCPTDRCLDPELLT----YDLPGSCRS

FWXCDGGESIPMCCPYGTSYHS-GX--GCL--PDN--KCKDPCPPRPTQYLNPRAIM---

SDKTNPEKNAPLSELSLP----GQKPKLKIKPRKPVC------DKKAVRGDSNSFEQFVE

RY-XWIKMPCAPGTQFSQGDCECTTTVPYSS-----------------------------

---NKTAECTSKVKLNFSDGFEDERDKRPVYIVNNNVS--FSGGVAKFSGSSRLRVPQLS

NVDYGNSVMLKIRFRDSNN-----------------------------------------

------------------------------------------------------------

------SSGRPQAIXSNADC--------------GN-NASILIAKDKD---RIIFGAQTE

NGD-YTQIELP-------------------------------------------------

------------------------------------------------------------

------------------------------------------------------------

----------------------------------------KPKTEWKNVKYSYNLGRLQG

TVNTDKVTRWIPVEVKS-SPDRALQIG----------HGENFEDFEGDI-----------

-DDLEIFTQCMPTDDMFIDEDYSGF-----------------------------------

------------------------------------------------------------

------------------------------------------------------------

------------------------------------------------------------

------------------------------------------------------------

------------------

>Lgig_XP_009051492.1 hypothetical protein LOTGIDRAFT_228264

------------------------------------------------------------

------------------------------------------------------------

------------------------------------------------------------

------------------------------------------------------------

------------------------------------------------------------

------------------------------------------------------------

------------------------------------------------------------

------------------------------------------------------------

------------------------------------------------------------

------------------------------------------------------------

------------------------------------------------------------

------------------------------------------------------------

------------------------------------------------------------

------------------------------------------------------------

------------------------------------------------------------

--------------------------MV--------------------CKLFL-------

-----AFL--------LALTVSVYSDEHQNVQYVAPISSYTECKSLLDVV----------

-VVVDGSDSIAADDFVTLKLALESLVLDLNVR--PDNTRFGVVLYSSTIA-GKIDISG--

NAGHIIPGIRALPHPRDGTNTALAIAEMNDMV-AA---QRRPGVPVVGVVITDGISKDQA

ATAQQAAIARN----QGIN-MFAIGVG-----------INVDTTELKSIASND--QQVLT

TVN---FNQLGSLL-SNFIQV-------------------------------VCPTTTTS

T-----------------------------------------------------------

------------------------------------------------------------

------------------------------------------------------------

------------------------------------------------------------

------------------------------------------------------------

------------------------------------------------------------

------------------------------------------------------------

------------------------------------------------------------

------------------------------------------------------------

------------------------------------------------------------

------------TTTTTTTT----------------------------------------

-------------TP---------------------------------------AP----

------------------------------------------------------------

------------TTSTTVPTTT----TTVKPDPCANCKMSNGIGFNPHPTDCDKYFQCEF

SLEGL-VNSVLRQCGQGLFWDQDLLTCNYPAAVQCRADPCQNYHISS----YKKAGNCRE

YYSCSNGTSMPECCKKGFAYVS-G---QCV--PSY--NCNAHCKGD--------------

-------------------------------FINPYC------EMRAVSDDISSYEQFVR

GV-GWVRKPCAPGSAFSPVECSCTVAIDPLP-----------------------------

---INAGECKAEVYIPFDDDVAIDKSGNGNYVENEGVF--VIGGKGYFNGTSGLRIPRFS

NIEFGSKVVITMRYKAES------------------------------------------

------------------------------------------------------------

-------IYGSQGLISNGDC--------------GK-PGSLLVAIDNT---NTLFGLQTV

SGT-AGIVTIP-------------------------------------------------

------------------------------------------------------------

------------------------------------------------------------

----------------------------------------S-ANGWNEIIYQVEGDVLTG

SVNGNSAHKTID--GAVKRSQCALQVG----------RATHLSNFRGYV-----------

-DELTVYLC---------------------------------------------------

------------------------------------------------------------

------------------------------------------------------------

------------------------------------------------------------

------------------------------------------------------------

------------------

>Lgig_XP_009045199.1 hypothetical protein LOTGIDRAFT_237510

------------------------------------------------------------

------------------------------------------------------------

------------------------------------------------------------

------------------------------------------------------------

------------------------------------------------------------

------------------------------------------------------------

------------------------------------------------------------

------------------------------------------------------------

------------------------------------------------------------

------------------------------------------------------------

------------------------------------------------------------

------------------------------------------------------------

------------------------------------------------------------

------------------------------------------------------------

------------------------------------------------------------

------------------------------------------------------------

------------------------------------------------------------

------------------------------------------------------------

------------------------------------------------------------

------------------------------------------------------------

------------------------------------------------------------

------------------------------------------------------------

------------------------------------------------------------

------------------------------------------------------------

------------------------------------------------------------

------------------------------------------------------------

------------------------------------------------------------

------------------------------------------------------------

------------------------------------------------------------

------------------------------------------------------------

------------------------------------------------------------

------------------------------------------------------------

------------------------------------------------------------

------------------------------------------------------------

------------------------------------------------------------

------------------------------------------------------------

------------------------------------------------------------

------------------------------------------------------------

-------MPCAPGTNYFMDTCSCSTFAKPV------------------------------

SYQPQKPDCRPLLYLPFDSDVRD-HSGNYNYVQNDGVT--IHNGAAYFNGHTGLRIPRFA

NMDFGSQLMIKFRYKKETQI----------------------------------------

------------------------------------------------------------

--------TRKQAVISNGDC--------------ST-SGSLYIIAADK---FTSFGIKTI

SHRA-AAVTVPS------------------------------------------------

------------------------------------------------------------

------------------------------------------------------------

-----------------------------------------EDTEWRDTMFYLDGNLLSG

SVNGDSS-QTLA--GPIQRKNCAIQIG----------RGTGFGNFKGYI-----------

-DDLKIYLCKPNKK----------------------------------------------

------------------------------------------------------------

------------------------------------------------------------

------------------------------------------------------------

------------------------------------------------------------

------------------

>Cvir_XP_022339846.1 asparagine-rich protein-like

------------------------------------------------------------

------------------------------------------------------------

------------------------------------------------------------

------------------------------------------------------------

------------------------------------------------------------

------------------------------------------------------------

------------------------------------------------------------

------------------------------------------------------------

------------------------------------------------------------

------------------------------------------------------------

------------------------------------------------------------

------------------------------------------------------------

------------------------------------------------------------

------------------------------------------------------------

------------------------------------------------------------

------------------------------------------------------------

------------------------------------------------------------

------------------------------------------------------------

------------------------------------------------------------

------------------------------------------------------------

------------------------------------------------------------

------------------------------------------------------------

------------------------------------------------------------

------------------------------------------------------------

------------------------------------------------------------

------------------------------------------------------------

------------------------------------------------------------

------------------------------------------------------------

------------------------------------------------------------

------------------------------------------------------------

------------------------------------------------------------

------------------------------------------------------------

------------------------------------------------------------

------------------------------------------------------------

------------------------------------------------------------

------------------------------------------------------------

--------------------------------------------MDSPLDLRW-------

----------------------------NDNVPTPAC------DKIPIANDMKHYQQLLP

GT-GYVTMPCAEGTHYNERKCKCTQQEPGYPT------------------------FKEP

SVQEPVAGCQPEVRLDFKNGVTD-SSGKWTYVNNQGVE--VKNGEAFFNGQGRLLIPRFT

NVDFGKTFVIRLRYRELEKLKF--------------------------------------

------------------------------------------------------------

--------NESQALVNNGDC--------------GD-FGSIQIFTKRN---SIGYVVKTT

KEPSHVSLQLHK------------------------------------------------

------------------------------------------------------------

------------------------------------------------------------

-----------------------------------------PHTEWKDVEYVVSDEKFEG

YLNGEQATKWSL--GSVESRQCAIQIG----------FGHGYNNFRGYI-----------

-SMLEIYLCKPDKAMKYS------------------------------------------

------------------------------------------------------------

------------------------------------------------------------

------------------------------------------------------------

------------------------------------------------------------

------------------

>Lgig_XP_009057764.1 hypothetical protein LOTGIDRAFT_233460

------------------------------------------------------------

------------------------------------------------------------

------------------------------------------------------------

------------------------------------------------------------

------------------------------------------------------------

------------------------------------------------------------

------------------------------------------------------------

------------------------------------------------------------

------------------------------------------------------------

------------------------------------------------------------

------------------------------------------------------------

------------------------------------------------------------

------------------------------------------------------------

------------------------------------------------------------

------------------------------------------------------------

------------------------------------------------------------

--------MESKPTLLSVFLS--------MLLVLPVTPAILPCQQSFDVV----------

-VMIDGSDSINADDFLLFKNALENLVGQLEIG--VDKTRMGLVLYSATVT-KSIDFSG--

NSFLLRQEIRSLVQPRDGTMTHLALDDAYNMVLRA---TSRQGVPKIGLVITDGISKFPS

LTAASAAKLKN----LGVN-VYAIGVT-----------NNVNAKELVDIASSP--SQVKY

SST---FSDLTTTL-KELLPE-------------------------------ICPATTTP

T-----------------------------------------------------------

------------------------------------------------------------

------------------------------------------------------------

------------------------------------------------------------

------------------------------------------------------------

------------------------------------------------------------

------------------------------------------------------------

------------------------------------------------------------

------------------------------------------------------------

------------------------------------------------------------

------------PTTPTPRTTRI-------------------------------------

------------TPP---------------------------------------GP----

------------------------------------------------------------

--------------GGRVDTTP----NPINGSLCDGCKEDNGIGFLPHPTDCHRFIQCIF

DKDGS-AEGMIKNCGMGLFWDQDDFTCKYPSQVTCVNDKCNNPGTTH----FKSIHNCRQ

YYSCDDRFGAALCCPQGTSYSDTSR--QCV--SDN--TCNDLCGTSSNP-----------

-----------------------------APTAQAQC------NKEAVPGLPGYYMEDVP

GF-GKIQRPCAVGTTFSPTKCDCSTFYDSNPAAPTRFPPVNNEFLNCDWVTTCSPTARQR

SVDSVTVHCKPILDLQFENDARD-TSGNNNWVQNVGVS--FQNGWAYFNGDAILRVPRFS

NFGLGSTFMVKMKYRAEAP-----------------------------------------

------------------------------------------------------------

-------IQQQQALFTNRDC--------------GK-PGSVAILLNRG---TDTFLMRNE

ASE-LKTVDVNV------------------------------------------------

------------------------------------------------------------

------------------------------------------------------------

----------------------------------------QDQTQWREVIYRVDNGLLKG

TVDGVSQQI--QIVGDIRKSECALQIG----------HGNTYENFVGYI-----------

-DYVQVSMC---------------------------------------------------

------------------------------------------------------------

------------------------------------------------------------

------------------------------------------------------------

------------------------------------------------------------

------------------

>Myes_XP_021338925.1 uncharacterized protein LOC110440276

------------------------------------------------------------

------------------------------------------------------------

------------------------------------------------------------

------------------------------------------------------------

------------------------------------------------------------

------------------------------------------------------------

------------------------------------------------------------

------------------------------------------------------------

------------------------------------------------------------

------------------------------------------------------------

------------------------------------------------------------

------------------------------------------------------------

------------------------------------------------------------

------------------------------------------------------------

------------------------------------------------------------

----------------------------------------------------MPNMG---

-------------RLVIGVIIAVM------SIKTIVTANHGQCRRLLDFV----------

-CVVDGSDSINEEDFQTLCDVVEKMVDEFEIS--PGEVRMGLVVFSTRIAT-TKPLTS--

NRAKLKVKIRNFPHPQEGTNTALGIKTMLDIFTSDKLRRKRPDIPAVGMVLTDGRSKSML

KTLKQARK-AKR---NGIE-MFAVGIS-----------DHVNMTELNGIASRP--ENVLT

VKT---FNQLRDSI-DRLVKM-------------------------------VCPTTTTT

TTTTTTTT----------------------------------------------------

---------------------TPTT-----------T----T-------------TPTTT

TTPTTTTTP---------------------------------------------------

-----------------------------TTT--TTPTTTTT------------------

------------------------------------------------------------

-------PTTTTTPTTTT-----TPTTTTTP-----------------------------

--------------------------------------------------------TTT-

-------------------------------------------TTPTTTTAPTTT-----

----------------------------TTPTTTT-------------------------

------------------------T--P-------------------TT-T---TTPTTT

---TTPTTTTTPTTTTTPTTT----------TTPTTT------TTPTTTTTP-TTT--TT

STTT--------TTP---------------------------TTTTTPTTTTTPTT--TT

------TPTTTTTPTTTTTTPTTTTIKQEIYHASSFRKTLKI------------------

---V-NIRKLAIKQQKEQRKKT----TGWIKGPCDDCSMMNGVGYNSHPNICSKFIQCYF

GPHGK-MRASYRECPFGFFFDPDVMACRLSYQCNCKRDKCKMRPHLKSYP-YKGIQNCRA

YWLCKRDVSTARCCAKGYRYRRGYGKNKCV--KDP--GCQDECPPTL-------------

-------------------------------TSVSKC------ETRMVFSNAKVYEQFIT

GWNMWVPRPCAPGSLYDADKCACTGHVSHI------------------------------

---PPKRKCKPEVYLPFTKDLKD-HSGSHSYVQNYNVK-RTKKGYAYFNGRSKLIIPRYS

NADFKE-IVIKIRFKVKKCKSVP-------------------------------------

------------------------------------------------------------

-----RRRPKLMALVSNSDC------------C-ND-DASLMMVLSQF---SIHYMAITK

HHE-LASF-HIA------------------------------------------------

------------------------------------------------------------

------------------------------------------------------------

-----------------------------------------KNDGWNDAYFIHDTKNLCG

RCNGAVKTAPSP--GSIKRTHTGIHIG----------YGRGFDNFKGYI-----------

-DEIKIYRCRPDFLKSQKKY----------------------------------------

------------------------------------------------------------

------------------------------------------------------------

------------------------------------------------------------

------------------------------------------------------------

------------------

>Cvir_XP_022335717.1 mucin-2-like

-MACLKQCLLL-VAAAGLIKGQSTPLFQANGKVVWQTDGFKFEENTSPTVGGTAVRFTSP

VPRSPVQNERTYVI--------SHGEGTIPSNLATELENVHMESVDNTIQGKSSLTESTV

NDSMN---PTDDIVDASVVDTLSQPNGDS-TAGRLEGSA-TNINTTVPANVTVESVNTDV

TMESTNPAVT--VTSTRNSDLETPSQNST-KTTSAAPIVITKSGAGFAILSASPKLNDIS

TTTVTYIPEEEFSIPKEAEEKITTVLPSSSTTSFSVDSFVDMLLPNEIKNQATGGVDVSK

NQTQQVFEESKAELPTTPGAIKESS----------TTEPTQTKQ--------------TT

FELESTPEHVTEKTTT--FEPIVKEMS--EPATKQEPTSES-TTKKATTPESKT------

--EQVT----------TSE--------PTTKLATTLK-----------------------

--PTTEQITSSGPTTKQTTTSEPTTEKTTTPESTTEQTTSTEPLT---------------

---------------T--EKTTTLEPTSEQTTTSEPTPYVGTTIEQTTEQATISEKTTEK

TTT----------SESTIEQ----KTTSEQ----------ITTTEPTTEQATGSTQPTTE

KATTPKPTSEQTTTSEPTPDVGTTIEQTTEQ-----------------------------

------------------------ATISELTTEKTSTSEPTTQKPTTPVPTSQKPSTPEP

TTQKLTT-----------------------------------------------------

----------------------------------------SEPTT--QKP----------

---------------------------------------TTPEPTTQKQTTPEPTTQETT

TPESTTQKPTTPE------P-----------TT--------------QEP----------

--TTPESTSQEQTIFVQTTQKPTTPEPTTQKPTTPEPTTQEPT-TSEPTTQEPTSPEPTT

QEPPEPT-TQKPTSTKPTTQEPTTPEPTTQEP-----TTPEPTTQK--------------

------------------------PTSPKPTTQE--------PTTPEPTTQ-EPTTPEPT

TQ------------------EPTTPEPTTQEPTTPEPLTQEPTTPEPTTQKPTTPEPQ-T

-------QE---------------------------------------------------

---------------PTTPELTTQKPTTSE----------------------------PT

TQKPTTSKP---------TTQE--------------------------------PT--TP

EPLTQEPTTPEPTTQ--K-----PTTPEPTTEVPTTPEPTTQDPTTPE------------

---------------------------PSTQEPTTPEPLTQEPTTPEP----TTQKP---

-TTPEPTTEVPTT-----------------------------------------------

--------------------------PKPTTQEPT-SPEPTTQEPTTSEPTTQKPTTPEP

TTQKPTTPEPTTQKPTTPEPTTQEPTTPEPTTQEPTSPEPTTQEPTTTEPTTQDP-----

------------------------TTPEPTTQEPTTPEPTTQ------------------

--EPTSPEPTTQEP-TT-----------------------------PEPTTQKSTTPEP-

TTQKPTKPEPTSQKPTTPEPTTQEPTTPEQTTQ--------ELTSPEPTTQEP-------

---------------------TTPEPTTQKPTTSEPTTQKPTAPEPTTLKPTTPEPTTQE

PTTSEPSTRKSTTPESTSQKP-------------TTPGPTTQKPTTPKPTTQKP---ITP

EPSVQAAVVSTKAPEAIVVTTK----APPLEDLCYNSVYIDSIGYNKYPGHCNKFVQCFS

NYEQT--KAVLRECPAGLFWHQDYAMCKSPEKVPCYEDHCLNLGVYA----YKRSGGCRS

YYSCDVGVSVPTCCKKGFRFDGQTC----V--QDS--SCNDPCQTPEDL-----------

----------------------------KRRLSQQMC------RFLPDKDNRFGYLTLEH

S--GIRFRACPYGTVFSARQCGCIWFQ------------------------------I-A

FQTPRKEVCKPDFKMNFDTNSFRELSGSNMAFYVENGA--VQNGAAKFGGNGKITIWGFM

NKELGHDFAVRVRFKPYQTEG---------------------------------------

------------------------------------------------------------

-------GYG--MLVSNCGH---------------EGLPTVEISMQDK---KARLIAKSV

HSNAPSILE---------------------------------------------------

------------------------------------------------------------

------------------------------------------------------------

--------------------------------------YHILPYEWNEISYHYNGNTFTA

EINGNSISEQLI--GGIETSPNPMFIGGCP---------QPGSGFNGLI-----------

-DYVEIYSTCVPSDFLQGRN----------------------------------------

------------------------------------------------------------

------------------------------------------------------------

------------------------------------------------------------

------------------------------------------------------------

------------------

>Cgig_XP_034298995.1 mucin-2-like

-MAFLKQCLIFLLVWIGLINGQSTPLFKANGKVVWQSEGFKFEENTSSTVGGTAGRFTSP

IPVSPMVRESTFVI--------LPAEGTQSGDLTPDFDNVHMESIDNKIQGKSSLTESVI

NESVDQIQTTNDVAEASIVDKLSQPEELTSTAGNIDDAATENKTTAVPADVIIEGERLDT

NMKSTNPDVIFTTSSTRNSDVETPAHNST-KTTSAAPIQISKSDDGVAILSARPKLNDIR

TTTVTYIPEEDFQTTDTLDNSDNTMTTSSTTGPFSIDSFVNMLLSEKMQAEVTGTDEIQQ

REKET-NPESIAKLETALGLTTEKTTTPKPTEKATTPEPTTEKTTTSEPTTEKATTPKPT

TGKAFTPEPTTEKATT--YEPTTEKANIPKPTTKKATTPEP-TTEKATTPEPKT------

--EKATTPEPTTEKTITSE--------PITELETTPEL----------------TTVKTT

TPKPTEKAFTPGPTTEKATTSEPTTEKATTPEPTTEKATTPEPTTETATDPEPTTE----

--------KATTPKPT--EKATTPEPTTEKAITPEPTTEKATTPEPTTEKATTPELTTEK

ATTPKPSTEKATNPEPTTEKAVTPEPTTEKAITPEPTTVKATTPEPTTEK-TTTPEPTTE

KTTTPKPTTEKATTSEPTTENATTPEPTTEKATTPEPKTETAFTPEPTTEKATDPEPTTK

KATTPKPTEKATTPEPTTEKATEKATTPEPTTEKATTPEPTTEKATTPEPTTEKATTPEP

TTEKIITPEPITELETAP-----ELTTEKATTPEPTTEKAITTEPTTEKATTPEPTTEKT

TTLKLTEKAITPEPTTEKATTPEQTTEKSTTPEPTKKVTTSEPTT--EKATTPEPTKETA

FTPEPTTEKANDPEPTTEKTTTPKSTEKATTPEPTTEKAITPEPTTEKATTPEPTTEKAT

TPELTTEKATTPK------L-----------TT--------------EKA----------

--TTPESTTEKATTSESTTEKVTTPEPTTEKATTPEPTTEKTT-TLRPTPEKAIDSERTT

EKATTPTPTEKATTPEPTTEKAITPEPTTEKA-----TTPEPRTEKAA---TTESTTEKS

TTLKPTE----------------KAVTPEPTTEK--------ATTLEPTTE-KANTPEPT

TE------------------KATTPEPTTEKAITPEPITEKATTPEPTTEKAITPEPT-S

-------ELETNPDPTTENTTKSKQTEKA------TTPEPTTEKAITTEPTTEKATSPEP

TTEKKVTLEPTT-ELETMPELTLTKTTTPKPTEKTTTPEPTIEKTTTPEPTEKATTPEPT

TEKTTTPKPTEKATTPEPTTEKAFTPEPTTEKTTTPK----PTEKAITPEPTEKAT--TP

EPTTEKATTPEPTTE--K-----IITSEPITELETAPELTTEKATSPEPTTEKAITTEPT

TEKATTPEPTTEKTTTPKPTEKATTPEPTTEKATTPEPTTEKEFTPEP----TTEKI---

-TTPEPTTEKTTTPKPTE--KAITPE----PTQKATTPEPTTEKAITPEPTEKATTPELT

TEKATTPEPTTEEIITSEPITELETAPELTTEKAT-SPEPTTEKAITTEPTTEKATTPEP

TTEKTTTPKPTK-KATTPEPTTEKATTPEPTTEKTFTPEPTTEKTTTLEPTTEKTT----

----TPKP---TEKAITPEPTQKATTPEPTTEKATTPEPTTEKIITSEPITELETAPKLT

TEKATSPEPTTEKA-ITTEPTTEKATTPEPTTEKTTTPKPTEKATTPEPTTEKATTPEP-

TTEKEFTPEPTTEKTTTPEPTTEKTTTPKPTEKAITPEPTEKATTPEPTTEKATTPEPTE

KATTPEPTTEKTTTPKPTEKATTPEPTTEKATTHEPTTEKAFTPEPTTEKTTTPEPTTEK

TTTLKP-TEKTTTPEQTTEKA-------------FTPEPTTEKTTTPGPTTQKP---TTA

APTTQTS------VTAKILTST----AAPLENLCVNSVYIDSIGYNKYPGSCNKFVQCYN

NYQNT--KAVLRECPAGLFWHQDHAMCKSPDKVPCFEDHCLNLGVDA----YKRSGGCRS

YFSCEYGVSVPTCCKKGFRFDGKAC----V--KDS--SCNDICVTPNDL-----------

----------------------------KRKLSQQMC------RFLPDKNNRFGYLTLEH

S--GIRFRACPYGTEFSARQCGCTWIQ------------------------------FTA

YQNPRKEVCKPDFKMNFDTNSFRELSGSNMAFYVENAV--AQNGAAKFRGNGKITIWGFM

NKELGHDFAVRIRFKPFNTEG---------------------------------------

------------------------------------------------------------

-------GYG--MLVSNCGH---------------EGLPTVEISMQDK---KARLIAKSM

HSSSPSVLE---------------------------------------------------

------------------------------------------------------------

------------------------------------------------------------

--------------------------------------YHIVPYEWNEISYHYNGNTFTA

EINGNSISEQLI--GGIETSPNPMFIGGCP---------KPGSGFNGLI-----------

-DNVEIYSTCVPSNFLQ-------------------------------------------

------------------------------------------------------------

------------------------------------------------------------

------------------------------------------------------------

------------------------------------------------------------

------------------

>Myes_OWF55652.1 Protein PIF

------------------------------------------------------------

------------------------------------------------------------

------------------------------------------------------------

------------------------------------------------------------

------------------------------------------------------------

------------------------------------------------------------

------------------------------------------------------------

------------------------------------------------------------

------------------------------------------------------------

------------------------------------------------------------

------------------------------------------------------------

------------------------------------------------------------

------------------------------------------------------------

------------------------------------------------------------

------------------------------------------------------------

--------------------------MK--------------------V-----------

----REINPGSPHNLK-VMPRER----ERHERTEVRDAVGICCRDLLDVI----------

-VVVDGSDSITAKDFVTLKRSLVDLIDGLQLA--DDQARFGLVLYSSDVP-AKLPLSA--

DRRRLRIDTLALNHPRDGTRTDLGIQAMRQMF-SR---QGRRNVPRVGIVITDGISKIPT

QTARQANAAQS----EGID-MFAVGVT-----------QLIDNDELRSIASNS--SNVMS

VSS---FDQLKSVV-SSLVRQ-------------------------------VCPTTTTT

T-----------------------------------------------------------

------------------------------------------------------------

------------------------------------------------------------

------------------------------------------------------------

------------------------------------------------------------

------------------------------------------------------------

------------------------------------------------------------

------------------------------------------------------------

------------------------------------------------------------

------------------------------------------------------------

------------TTTTTTTTTTTTT-----------------------------------

------------TTP---------------------------------------APTT--

---------TTTTPTTTTTTPTT----------------------------TTP------

--T-----TTTPTTTTTTPTPT----TTKPIDPCDGCRMRNGAGFNRHPTDCDKFVQCYF

TADGK-RQHSIQECPWGNFWDQETMTCKPAHMVFCLTDKCGDPYMQA----HKHTSDCRS

FWYCNNRKSFPMCCPMNTSFVE-NV--GCV--PDN--SCHEHCPPRDS------------

-----------------------------------EC------DKKEIADAPMHFEQFVA

RS-GWIRMPCAPGTSYNQRDCQCTVISEYVK-----------------------------

----KDEGCKSKVSLNFATYIEEN-SDKSVYVVNENVS--VAHGVAIFDGKSSLRVPQLS

NMDLGDTVVLKIRYKDTPKRHP--------------------------------------

------------------------------------------------------------

-----RATDRPQALISNGDC--------------GN-NASVLLAKDDT---SIMFGAETD

KGG-YTSFSIP-------------------------------------------------

------------------------------------------------------------

------------------------------------------------------------

----------------------------------------TPVSHVV-------------

------------------------------------------------------------

------------------------------------------------------------

------------------------------------------------------------

------------------------------------------------------------

------------------------------------------------------------

------------------------------------------------------------

------------------

>Mcal_P86860.1 PSM_MYTCA RecName: Full=Shell matrix protein

------------------------------------------------------------

------------------------------------------------------------

------------------------------------------------------------

------------------------------------------------------------

------------------------------------------------------------

------------------------------------------------------------

------------------------------------------------------------

------------------------------------------------------------

------------------------------------------------------------

------------------------------------------------------------

------------------------------------------------------------

------------------------------------------------------------

------------------------------------------------------------

------------------------------------------------------------

------------------------------------------------------------

------------------------------------------------------------

------------------------------------------------------------

------------------------------------------------------------

------------------------------------------------------------

------------------------------------------------------------

------------------------------------------------------------

------------------------------------------------------------

------------------------------------------------------------

------------------------------------------------------------

------------------------------------------------------------

------------------------------------------------------------

------------------------------------------------------------

------------------------------------------------------------

------------------------------------------------------------

------------------------------------------------------------

------------------------------------------------------------

------------------------------------------------------------

------------------------------------------------------------

------------------------------------------------------------

------------------------------------------------------------

--------------------------------------------------------KGYQ

YLPVVLKVIAMTTIKNVSEHQAGIC----V--T-------MSVKLPAIL-----------

----------------------------TRQLAQTSC------PSLPDPMNRYGYLAPQY

G--GLRIRACPSGTIYSENQCRYKSNMNGNGG-------------------------LRG

SARKQFRQCSAEFKINFDD-GFKDISKGGLAFDYSHIS--LRRGKGVFVGNSKLYIWGFQ

SRFLGKTFAIRMKVKIKRGAG---------------------------------------

------------------------------------------------------------

-------KYRPEPIISNCGP---------------NGDSSVEIVVHRG---KVIFKAKTS

DNPEAVFIT---------------------------------------------------

------------------------------------------------------------

------------------------------------------------------------

--------------------------------------EDYDDDKWTDLTYYYDGNHFGG

SCNGRPFRQRTG--GNLEIRDNPMTIGLCT---------G-QNGFHGEI-----------

-DELEIYTACIPKDM---------------------------------------------

------------------------------------------------------------

------------------------------------------------------------

------------------------------------------------------------

------------------------------------------------------------

------------------

>Pmar_H2A0N4.1 PIF_PINMG RecName: Full=Protein PIF; Contains: RecName: Full=Protein Pif97; Contains: RecName: Full=Protein Pif80; AltName: Full=Aragonite-binding protein; Flags: Precursor

------------------------------------------------------------

------------------------------------------------------------

------------------------------------------------------------

------------------------------------------------------------

------------------------------------------------------------

------------------------------------------------------------

------------------------------------------------------------

------------------------------------------------------------

------------------------------------------------------------

------------------------------------------------------------

------------------------------------------------------------

------------------------------------------------------------

------------------------------------------------------------

------------------------------------------------------------

------------------------------------------------------------

------------------------------------------------------------

------------------------------------------------------------

------------------------------------------------------------

------------------------------------------------------------

-------------------------------------------------------MQVPY

LQI---------VFLLTAVFG-------------------------------IGVKSDDC

KTADLVVNVDGSDDVSDREFDKLKRAMLM-LVRGLSIDDSQIRLGMVTYGSE--------

-IGDSI---P-----LQGDRLDLAR-----------T--------------IRYMKKPGG

PC-----KP-----------FKGI------------------------GETRKMFSSRGR

FNVPHV-------------------TLNLGGDIVDSEVRDLMDETDKARDEDIKVMAIGL

GTKVERDEIE----------GIAW-----D----KEQAYFM-------------------

------------------------------------------------------------

------------------------------------------------------------

------------------------------------------------------------

------------------------------------------------------------

------DDADDLVRRVKEIPDYLCKI----------------------------------

----------------------------------IKA------KKPRKSASK--------

---------KSKTKP---------------------------------------AKKPDS

------------DI--VGKSP---------GFH-----SLQR-------TDDKP------

------------------KMSK----KVEVKELCDDAEWVEDVGYGSVPTRCEDFVMCQN

VSGSL--RKTLKTCPYGQFWSRARTSCVLTEDEDCSDDLCKTMLLPS----RDYDVSCRA

YWKCENGKSVARCCPSGMAYEPGK---GCV--LDS--DCDEECPPKGDSDNGDDDDDDND

DD--------------DN----EYDDDDDEMEYNPNC------PLRPIKGSPEKFKQH-T

GDDNWEEFDCAPGTLFSSRDCACSILGRPEKD------------------------DNGK

NEDDTSKVCEPELYLPFCDDLHD-YSGKETHVENEGDAVIIENGKAYFNGRAGLKIPRFS

GVPYGKSVFIKMKYKEDEDDDKKRNDDDKKLRIKRDERGRKGYRKGGRKDDRNGKRRDDR

IGKRRDDRIFDDRKGRRTDDRKGDRRDRIDDRNGRRTDDRKDNRRDDRKDNRRDDIKDNK

RDDKKDNADEPMTLISNGECD-----NFELHDC-FE-KPSLAITTGKK---FAGFSVSST

ERD-EVDLEVDNDKKGYLWNKDKKDKDDKNRRDKDKNGDRTDDKKSLDDLVKEIE-----

---------RRK--SDDKKSFDDLVKEIERRKSDDKKSFDDLVKEIE-------RRKSDD

KISLDDLVKEIKRRKSD-------------------------------------------

----------DKGNGRRKDDN--------------KNDEDDDKKGWKTVSLKINNGHIRG

RRDDREDKDIMD--GDLKTTFSGFQIG----------QGASNKNFKGYM-----------

-DEVYIYFCDPGKEADFDEEDDNGDDDDDDDDDDDKDNDAGDDNKD------DNNNNGRK

DDNNNDGRKDD----NN---KKD----------DKDRSDKNGGKDDK-------------

------DKDTKDKFSDK--DNGKDNEDADRDINDNDKL--------YRR------AMKKC

DFVNKNVEKWLDKR----------------------------------------------

------------------------------------------------------------

------------------

>Pfuc_C7G0B5.1 PIF_PINFU RecName: Full=Protein PIF; Contains: RecName: Full=Protein Pif97; Contains: RecName: Full=Protein Pif80; AltName: Full=Aragonite-binding protein; Flags: Precursor

------------------------------------------------------------

------------------------------------------------------------

------------------------------------------------------------

------------------------------------------------------------

------------------------------------------------------------

------------------------------------------------------------

------------------------------------------------------------

------------------------------------------------------------

------------------------------------------------------------

------------------------------------------------------------

------------------------------------------------------------

------------------------------------------------------------

------------------------------------------------------------

------------------------------------------------------------

------------------------------------------------------------

------------------------------------------------------------

------------------------------------------------------------

------------------------------------------------------------

------------------------------------------------------------

-------------------------------------------------------MQVPS

IRV---------VFLLTAVFC-------------------------------VGVKSDEC

KTADVVVNVDASDDVSDQDFDKLKRAMLM-MVRGLSIDDNQIRLGMVTYGSE--------

-VCDSI---P-----LQGDRLDLAR-----------T--------------IRYMKKPTG

PS-----KP-----------FKGM------------------------GEARRMFSSRGR

YNVPHI-------------------TMNLGGDIVDTEVKDLMDETDKARDEDIKVMAIGL

GAKVDRDEIE----------SIAY-----D----RDQAYFM-------------------

------------------------------------------------------------

------------------------------------------------------------

------------------------------------------------------------

------------------------------------------------------------

------DDEDDLIRKVKEIPDYLCKI----------------------------------

----------------------------------IKA------KKPKVSGGK--------

---------K--SKP---------------------------------------AKKVDN

------------GP--AGKSP---------GFD-----ALKQ-------SDDKS------

------------------DKAK----KVEVKELCDDAEWVDGVGYGSVPTRCEDFVMCQN

VSGSL--RKTLKSCPFGQYWSKRQTSCVLTEDEDCSDDLCKTMLLPS----REYDVSCRA

YWKCEKGKSVARCCPSGMAYEPGK---GCV--LDL--DCDEECPPKNDGDDDDD------

-----------------S----SDEDDDDEIEYNPNC------PLRPIKGHPEKFKQH-T

GDDNWEDFDCAPGTLFSARDCACSILGTAKKD------------------------DKND

DGGDAHKVCEPELYLPFCDDLHD-YSGKETHVENEGDAVIIENGKAYFNGRAGLKIPRFS

GVPYGKSVFIKMKYKEDEDDDKNKNDDDKKLRMKRDERSRKDYLKAILKRDDRKDKTDDT

-------------KGR----RIIDRNDIIDDRRGRRKDDRKDGGRDDGKDGRRDDRKDNR

LDDIKDKNDEPMTLISNGECD-----NFELNDC-FE-KPSIAITTGKK---SAGFSVTSS

EKD-EVDLEIDEDKKGYLWDKEDG--PDRNGKDKDRNGDRSDDRRGYYWKKKDKDDNGKD

KDKK-----GDK--SDDKKGYYWK---------KDKDDKNGKDKDKR-------RDKSDD

KKSLDDIVREIERSK---------------------------------------------

--------------GNGKDDN--------------KDDEDDDKKGWKTVSLKISNGHIRG

RRDDREDKDVLD--GDLKTTFSGFQIG----------QGASNKNFKGYM-----------

-DEVYIYFCDPGKEADYDDEDDDDDDDDSDENDKNDDKKDGKKTDDKDK---KDRKDGRD

--DRKDGRDDR----KD---RRDDRKDDRKGGKDDRKDDRKGGKDDRKDGRDVRDDRDRG

DKYDKKDDKDNDRLSDK--DDRKDVDD-NDKDDDNEKL--------YKR------AMKKC

DYVNKNVAKWLDKR----------------------------------------------

------------------------------------------------------------

------------------

>Pmar_H2A0M0.1 NRP_PINMG RecName: Full=Asparagine-rich protein; AltName: Full=Prism uncharacterized shell protein 1; Short=PUSP1; Flags: Precursor

------------------------------------------------------------

------------------------------------------------------------

------------------------------------------------------------

------------------------------------------------------------

------------------------------------------------------------

------------------------------------------------------------

------------------------------------------------------------

------------------------------------------------------------

------------------------------------------------------------

------------------------------------------------------------

------------------------------------------------------------

------------------------------------------------------------

------------------------------------------------------------

------------------------------------------------------------

------------------------------------------------------------

------------------------------------------------------------

------------------------------------------------------------

------------------------------------------------------------

------------------------------------------------------------

------------------------------------------------------------

------------------------------------------------------------

----------------------MKGTSALLLIGFFHATISQDPGGTVVIGSL--------

-SGRKRGN------LNTGGQITSNS-----------A----I--LGDVNAGSKLSEPPKR

RN---------------------------------------------TDKTARMLENNPR

IGGS--------------------------------------------------------

------------------------------------------------------------

---------LIPPPGVIMEP----------------------------------------

-------NPYD------INPPY----FV--------SNKNSQSTNSATN-TMLQSLTSD-

-------------------------------------------TK--------TTT-RT-

--------------------------SQTSSTRASSSI-----------TQGINTMNRN-

SMRFNQVDRNKISGFVNSGGQIQTN--P------L-----------N---T---NSQSSP

IL----------------------AA-----QRQITR------QKSENTQGNSIVRNGGT

NSL---------NIP---------------------SSTRRSQPPNMAV---------Q-

------I-----GQNTATFNMG----TDGKVLHKFLPTNLFE----NINSVSKEPRNTAS

VPGIGGMRNPGPSISIRNIFGTNNIEGSSVQLTGSSGVFVSDPGPKGNPTDVPQFVPSGI

SPTV---RDPNALDPFKSIRNQIVP-DIKRNEVNRGNSMISAPVI-------DNPTNSNS

MV---ELNSILQNVQNGFLSEALGN----S---NN--QNNRVTN----------------

----------IV-----------NQINSADPQPVRRCQFLPYRDLRTNKIDRRYFRQLVN

G--KWLNLKCADGAGFNETTCLCSIHLT--------------------------------

----GDAQCSPEVRLNFNDGTIQNLTPINVHIDAEGVD--ASKGWAHFNGSTQMKFEYFN

AYDVQRDFLIKLRFKADSYIPNQ-------------------------------------

------------------------------------------------------------

----------SHPIVTNCVAG--Q----------ENTDPSIGVFLTGNYPHKIVFILQTD

KSK---------------------------------------------------------

------------------------------------------------------------

------------------------------------------------------------

--------------------------------LLQHLIFDVPRDGWHDITYKYDGSTLTG

ILDGKEKSLPTE--GRIENRQAVLVFG-----------GCGNRIFRGNI-----------

-DDIQIYTCIPPSHRNKG------------------------------------------

------------------------------------------------------------

------------------------------------------------------------

------------------------------------------------------------

------------------------------------------------------------

------------------
