# Supplementary material for: Mantle Modularity Underlies the Plasticity of the Molluscan Shell: Supporting Data From Cepaea nemoralis
Source: Front Genet. 2021 Feb 5;12:622400. doi: 10.3389/fgene.2021.622400 (PMC7894901; doi:10.3389/fgene.2021.622400)
Supplement: Supplementary file 5 [file Data_Sheet_5.docx]

>Cnem_R27072766

CNNCVVHHGV GYAPLPGYCD AYVQCRFCPS GNYWNQDKLV CDFQDNVKCT P-VNWAAYSI

FNGANWTRVA CPERRLYNHE ICYLQF-TGS WIRRPCPATL VYHADICVCC KATVALNFDN

NNATSVNHFW VNNTGVGKAY FNGKSRLTIP GLSNMEFGST VYILIKYRTL VSNGDCFYAE

TWQYALYALD NGNLLGSVGV GGGCDNFHGI IDDVRVYLC

>Lsta_jg75923.t1

CKDCVILHGV GYAAVAGHCD AYIQCRYCPY GLQWNQNKLT CDKEENVKCV S-VSRRGYLA

FNGYTWSRVS CPSDYTYNFE TCFLQF-NGS WIRMPCPATL GYDPRTCRCC KPSLSLSFDD

NTATSENQFW INNTGVGQAY FDGNSRLTVP GFSNMEFGNK VYVTVKYSTL LTNGDCFFAE

TWQYAMYALD KGHLQGYVGT GGECGDFTGI IDELHVFFC

>Lsta_jg74475.t1

CEGGAMVNGV SYRSHWADCT KFIQCSFCRH GMYWDQTKLA CDNARNVDCP YDLCCRGYWI

CEAGKSVGHC CPPQHSYCND PCFEQSVAGG WDRKSCSPGT AFNIKACACC EPELYLPFNG

DIKDSGNNNY LQVDGVGTGY FDGKSLIRVP RFSNMDFGST LIIKLRYKAL VANGDCMGLR

TWKEAVLIAD DSVLQGTVTG GTEFKHFTGY MDDVTVYLC

>Lsta_jg35514.t1

CLQCLVEGGV GFNPLPEDCE KYVLCFPCPF GMFWSNESVS CLDARYVDCP WDKCCRSYMR

CVNGRSTPTC CETGYRYCQD VCYLEVIPGG NVKRNCDPGQ VYSPALCACC LPAVNLTFDG

SFEDSTNALH IDVKKVGTAY FDGDGYITLP TTANLDLGDK FSIRLRYRTI TKNGTV----

-WKITRA--- --------NG GWKVKLPNGD IDLFTKHYS

>Bgla_XP_013087207.1

CRDCVILHGV GYAPYPGYCD AYIQCQFCPT GLQWNQNKLT CDFPEIVQCK SVVAKQEYLS

FNGYTWTQLR CPVNQVYNFE TCYLQF-NGS WIRMPCPASV GYDSRTCQCC EPSLALTFED

NSATSINQFW VNNTGVGKAY FDGQSRLTVP GFSNMDLGNT ATDT------ ----------

---------- ---------- ---------- ---------

>Pcan_XP_025084945.1

CDGCELRHGV GYKPHPSDCT LYVQCQACPH GLYWNQDKLT CDYRHNVNCV DDICCRGYWD

CNSGTALAKC CPINYSYCRD DCYEVK-TGT WTLMSCPANL GFSTLPCGCC VPELYLPFIS

DTQDSGRQVF VKNEGVGKAF FDGKSRLTVP RFSNTWWGST VYVHLRYKAL VSNGDCFYAE

TWQDVLYRLD AGSLYGHVSL GGGCDDFNGF IDEVTVYLC

>Pcan_PVD33241.1

CDGCELRHGV GYKPHPSDCT LYVQCQACPH GLYWNQDKLT CDYRHNVNCV DDICCRGYWD

CNSGTALAKC CPINYSYCRD DCYEVK-TGT WTLMSCPANL GFSTLPCGCC VPELYLPFIS

DTQDSGRQVF VKNEGVGKAF FDGKSRLTVP RFSNTWWGST VYVHLRYKAL VSNGDCFYAE

TWQDVLYRLD AGSLYGHVSL GGGCDDFNGF IDEVRIS--

>Pmax_XP_033751871.1

CLGCKMVNGA GFNPHPTDCS KFVQCFFCPV GHFWNQEKLT CDYAFRVNCV HDMCCQAFWD

CENGHAIQRC CPAGQAYCPP TCYEQEVEGG WLMMPCAPGT AFDQKECRCC TAEVILNFDN

DVQDSGKYVW VTNKGVGSAV FDGKSELLIQ RFTNDDFGFT FIVRMRYRAL ISNGDCFGVD

TWKTVEFKLA DGKLQGRANE GWGLENFVGD IDQLEIYKC

>Cgig_XP_011422884.2

CEGCKMINGA GFNSHPNECD LFVHCYFCPF GQFWNQTILS CEYSERAYCP MDRCCRAYWE

CSNGHSRGKC CRYGYRYCKE SCYQQLLPGG YVTMPCAEGT HYNERKCTCC RPEVKLDFKN

GVTDSGKWTY VNNQGVGEAI FNGDSRLLIP RFTNVEFGKT FVIRLRYKAL VNNGDCYVVK

TWKDVEYIVS DGKFEGYLNG GHGYNNFRGR LSLLEIYFC

>Myes_XP_021377426.1

CLGCKMVNGA GFNPHPTDCS KYVQCFFCPV GQFWSQEELT CDYAVRVNCI HDMCCQAYWF

CEDGHAIQRC CPPGYAYCPP TCYQRNVEGG LMTLPCAPGT NFDIKECRCC KAEVILDFDK

DVQDSGNYLW VTNKGVGKAI FDGKSELLIQ RFTNDDFGHT FTVRIRYRAL ISNGDCFGVD

TWKMVELKLA DGKLQGRANE GWGLNNFIGE MDNLEIYKC

>Cgig_XP_011456399.2

CDSCKMSNGA GFTRHPTDCS KFIQCYFCPW GNFWDQSSLT CQPAHRVKCP TDRCCRSFWA

CDGGESIPMC CPYGTSYCKD PCFEQFVERG WIKMPCAPGT QYSQADCECC TSKLKLNFSD

GFEDDKRPVY IVNNNVGVAK FSGKSRLRVP QLSNVDYGDS VMLRIRFRAL ISNADCFGAH

TWRNVKYSYN LGRLQGSVNT GENLDDFEGD IDDLEIFTQ

>Cgig_XP_011456398.2

CDSCKMSNGA GFTRHPTDCS KFIQCYFCPW GNFWDQSSLT CQPAHRVKCP TDRCCRSFWA

CDGGESIPMC CPYGTSYCKD PCFEQFVERG WIKMPCAPGT QYSQADCECC TSKLKLNFSD

GFEDDKRPVY IVNNNVGVAK FSGKSRLRVP QLSNVDYGDS VMLRIRFRAL ISNADCFGAH

TWRNVKYSYN LGRLQGSVNT GENLDDFEGD IDDLEIFTQ

>Lgig_XP_009066027.1

-----MRNGV GFNSVAGSCQ KFIQCIFCPA GLFWDQDKLT CNYASEVDCT EDPCCREYYT

CFNSVSFEKC CLPGYAFCVD SCYEQ-AVGE WIVMSCPLGA LYSQEECKCC KPSVQLSFDS

GTYDSGNFNY VQNNGVGVAY FDGQSFLRIP RFANVDFRKT VTIKMKYKAL VTNGDCLGLI

SWNEVEYKVV DGELISAVNG GDGFDNFKGW IDELSVYLC

>Pfuc_AYN73061.1

CDQCKMSNGA GFTRHPTDCD KFVQCYFCPW GNFWEQSSLT CKPAHRVQCP TDKCCRSFWA

CDQGDSIPMC CPEGTMYCKD PCFEQFVDRG WVKMPCAPGT AYDTKDCECC KAKVKLNFTE

GFEDDKRPVY VVNNNVGVAK FDGTSRLRVP QLSNVDYGEA VVLKIKFRAI ISNGDCFGAE

TWKTVKYAYN DGRLQGTVNG GEGLGDFQGD IEDIEIFTQ

>Lgig_XP_009056819.1

CNRCIYRNGV GFLSHTSDCT KFFKCQRCPF GLYWNNEIFS CDYPRNTNCT NHPCCAGYWR

CDWATPVAYC CPQGHRFCRD DCFEQRVPVR WIRLDCAPST GFNPKTCLCC DPMLHISFNN

GVRDSRHRFW IENVGVGVGL FNGNNKLLVN RFANAPLGRD LVIEVVYEVL VSNGDCFSVK

TLIKARLSLA NGRLTGEVGG GDGMKKFDGI MDDVKMFFC

>Lgig_XP_009066028.1

CDGCLYKNGV YLKDHPTDCN KFLQCNQCPQ GLFWDQDLLL CNYPENTNCT KDPCCRQYFK

CVNGISYLEC CESGYGFCTE ACFEQKIPSG WIRMNCALGT AFNETACYCC TPELYLPFDN

NTRDSGNGFY VQNNGVGVAY FDGNSSLRIP RFTNVDFGTK LRITFKYKAL VTNSDCFSTR

TWNEVEFKYD NGVFTGTVNG GDQFSNFKGW IDEVYVYMC

>Pmax_XP_033752064.1

CDDCSMMNGV GYNPHPKICS KFTQCYFCPF GFFFDTNVLS CRLSFQSHCN NDKCCRAYWL

CKNGRSAARC CDKGFRYCQD ECYEQFIKGM WIPRPCAPGS QYNADKCACC KPEVYLPFTK

NLKDSGSHSY VQNYNVGYAY FNGRSKLIIP RYSNAEFKD- LVIKIRFKAL VSNSDCYMAI

SWNTAYFVHD TKTLYGRCNS GRGFNSFKGY IDEIKIYRC

>Cvir_XP_022318425.1

CDSCKMSNGA GFTRHPTDCS KFIQCYFCXW GNFWDQTSLT CQPAHRVKCP TDRCCRSFWX

CDGGESIPMC CPYGTSYCKD PCFEQFVERX WIKMPCAPGT QFSQGDCECC TSKVKLNFSD

GFEDDKRPVY IVNNNVGVAK FSGSSRLRVP QLSNVDYGNS VMLKIRFRAI XSNADCFGAQ

TWKNVKYSYN LGRLQGTVNT GENFEDFEGD IDDLEIFTQ

>Lgig_XP_009051492.1

CANCKMSNGI GFNPHPTDCD KYFQCEFCGQ GLFWDQDLLT CNYPAAVQCR ADPCCREYYS

CSNGTSMPEC CKKGFAYCNA HCYEQFVRGG WVRKPCAPGS AFSPVECSCC KAEVYIPFDD

DVAISGNGNY VENEGVGKGY FNGTSGLRIP RFSNIEFGSK VVITMRYKGL ISNGDCFGLQ

TWNEIIYQVE GDVLTGSVNG ATHLSNFRGY VDELTVYLC

>Lgig_XP_009045199.1

---------- ---------- ---------- ---------- ---------- ----------

---------- ---------- ---------- ---MPCAPGT NYFMDTCSCC RPLLYLPFDS

DVRDSGNYNY VQNDGVGAAY FNGHTGLRIP RFANMDFGSQ LMIKFRYKAV ISNGDCFGIK

TWRDTMFYLD GNLLSGSVNG GTGFGNFKGY IDDLKIYLC

>Cvir_XP_022339846.1

---------- ---------- ---------- ---------- ---------- ----------

---------- ---------- --YQQLLPGG YVTMPCAEGT HYNERKCKCC QPEVRLDFKN

GVTDSGKWTY VNNQGVGEAF FNGQGRLLIP RFTNVDFGKT FVIRLRYRAL VNNGDCYVVK

TWKDVEYVVS DEKFEGYLNG GHGYNNFRGY ISMLEIYLC

>Lgig_XP_009057764.1

CDGCKEDNGI GFLPHPTDCH RFIQCIFCGM GLFWDQDDFT CKYPSQVTCV NDKCCRQYYS

CDDRFGAALC CPQGTSYCND LCYMEDVPGG KIQRPCAVGT TFSPTKCDCC KPILDLQFEN

DARDSGNNNW VQNVGVGWAY FNGDAILRVP RFSNFGLGST FMVKMKYRAL FTNRDCFLMR

NWREVIYRVD NGLLKGTVDG GNTYENFVGY IDYVQVSMC

>Myes_XP_021338925.1

CDDCSMMNGV GYNSHPNICS KFIQCYFCPF GFFFDPDVMA CRLSYQCNCK RDKCCRAYWL

CKRDVSTARC CAKGYRYCQD ECYEQFITGM WVPRPCAPGS LYDADKCACC KPEVYLPFTK

DLKDSGSHSY VQNYNVGYAY FNGRSKLIIP RYSNADFKE- IVIKIRFKAL VSNSDCYMAI

TWNDAYFIHD TKNLCGRCNG GRGFDNFKGY IDEIKIYRC

>Cvir_XP_022335717.1

CYNSVYIDSI GYNKYPGHCN KFVQCFSCPA GLFWHQDYAM CKSPEKVPCY EDHCCRSYYS

CDVGVSVPTC CKKGFRFCND PCYLTLEHSG IRFRACPYGT VFSARQCGCC KPDFKMNFDT

NSFRSGSNMA FYVENGGAAK FGGNGKITIW GFMNKELGHD FAVRVRFKML VSNCGHLIAK

SWNEISYHYN GNTFTAEING -QPGSGFNGL IDYVEIYST

>Cgig_XP_034298995.1

CVNSVYIDSI GYNKYPGSCN KFVQCYNCPA GLFWHQDHAM CKSPDKVPCF EDHCCRSYFS

CEYGVSVPTC CKKGFRFCND ICYLTLEHSG IRFRACPYGT EFSARQCGCC KPDFKMNFDT

NSFRSGSNMA FYVENAGAAK FRGNGKITIW GFMNKELGHD FAVRIRFKML VSNCGHLIAK

SWNEISYHYN GNTFTAEING -KPGSGFNGL IDNVEIYST

>Myes_OWF55652.1

CDGCRMRNGA GFNRHPTDCD KFVQCYFCPW GNFWDQETMT CKPAHMVFCL TDKCCRSFWY

CNNRKSFPMC CPMNTSFCHE HCFEQFVARG WIRMPCAPGT SYNQRDCQCC KSKVSLNFAT

YIEESDKSVY VVNENVGVAI FDGKSSLRVP QLSNMDLGDT VVLKIRYKAL ISNGDCFGAE

TVV------- ---------- ---------- ---------

>Mcal_P86860.1

---------- ---------- ---------- ---------- ---------- ----GYQYLP

VVLKVIAMTT IKNVSEH--M SVYLAPQYGG LRIRACPSGT IYSENQCRYC SAEFKINFDD

-GFKSKGGLA FDYSHIGKGV FVGNSKLYIW GFQSRFLGKT FAIRMKVKPI ISNCGPFKAK

TWTDLTYYYD GNHFGGSCNG -G-QNGFHGE IDELEIYTA

>Pmar_H2A0N4.1

CDDAEWVEDV GYGSVPTRCE DFVMCQNCPY GQFWSRARTS CVLTEDEDCS DDLCCRAYWK

CENGKSVARC CPSGMAYCDE ECFKQH-TGN WEEFDCAPGT LFSSRDCACC EPELYLPFCD

DLHDSGKETH VENEGDGKAY FNGRAGLKIP RFSGVPYGKS VFIKMKYKTL ISNGECFSVS

SWKTVSLKIN NGHIRGRRDD GASNKNFKGY MDEVYIYFC

>Pfuc_C7G0B5.1

CDDAEWVDGV GYGSVPTRCE DFVMCQNCPF GQYWSKRQTS CVLTEDEDCS DDLCCRAYWK

CEKGKSVARC CPSGMAYCDE ECFKQH-TGN WEDFDCAPGT LFSARDCACC EPELYLPFCD

DLHDSGKETH VENEGDGKAY FNGRAGLKIP RFSGVPYGKS VFIKMKYKTL ISNGECFSVT

SWKTVSLKIS NGHIRGRRDD GASNKNFKGY MDEVYIYFC

>Pmar_H2A0M0.1

GSSGVFVSDP GPKGNPTDVP QFVPSGIDPF KSIRNQIVP- DIKRNEVNRG NSMISNSMV-

--ELNSILQN VQNGFLSNNR VTFRQLVNGK WLNLKCADGA GFNETTCLCC SPEVRLNFND

GTIQTPINVH IDAEGVGWAH FNGSTQMKFE YFNAYDVQRD FLIKLRFKPI VTNCVAFILQ

TWHDITYKYD GSTLTGILDG GCGNRIFRGN IDDIQIYTC
