# Supplementary material for: Mantle Modularity Underlies the Plasticity of the Molluscan Shell: Supporting Data From Cepaea nemoralis
Source: Front Genet. 2021 Feb 5;12:622400. doi: 10.3389/fgene.2021.622400 (PMC7894901; doi:10.3389/fgene.2021.622400)
Supplement: Supplementary file 7 [file Data_Sheet_7.docx]

>Cnem_R27072766 [organism=Cepaea nemoralis] TransAbyss assembly 2 (filtered min reads 10, dedupe95) len=2716 num_reads=5670257 avg_cov=209491.4 contig_cov=100.0% (contig_821 from old CLC assemly 9)

TTTTTTGTTTAAGGAAAGGTGTTTTTATTTCAGTGTAACAACACTGCATGTGAGCGAATATCTTTCTTTGACAATTTAGGTTATGGATATTTCGTAAATCTAGGCAGGCATAAAACAAGAGATTAAATTTGTTGCTCCAATTGCTGATGAGTACTCTAAGCTTGTAACATTGGATTTCCTTCTATTGACTGGAGACTGAAAGGTAATTGATAGTGTGTTCGTTGGCTCCTTATCTGTTCCATTACAATCATAACTCTGGTTTACACAGGTAAACTCGGACGTCATCAATGATTCCATGGAAGTTATCACAACCACCACCTCCACCAATAACCAGGCCTCTTTGGCGTCTGTCCAGTGCACCTTTGACTGGCTGTGCAATTTTATTAACACCAACACTTCCAAGCAAGTTACCGTTGTCCAGGGCATAAAGTGCATATTGCCATGCACCAACGTCAGTGGGAACTGTAGTTTTGCCGAGAGAAATCTGCTCCTTGGTTTCAGCATAGAAGTCTACAGAGTCCTTGCCGGAGCAGACTGCCAACGACTGTCGCACCTGGCAGTCTCCATTAGACACCAGAGTTTGCTGGGAGTTCGCTGAGCTGTGTCTGTACTTGATTAAGATGTACACTGTACTTCCAAACTCCATGTTAGAAAGTCCAGGAATGGTCAGTCGACTTTTTCCATTGAAGTATGCCTTGCCATCATTGAAGGTCACGCCTGTATTGTTGACCCAGAAGTGGTTCACGGACGAGTCGGTGGCATTATTATTGTCAAAATTCAAGGCCACAGTTGCTTTGCACACGCCATGTTGCTTGCTCTTGTTATCATCATCATCAACAACATTTGTCTGATCATAGGAGCAGACGCATATGTCTGCGTGGTAGACAAGAGTCGCAGGGCAGGGTCTACGAATCCAAGACACACCAGTGAACTGCAAGTATCCAGATTTGTCACCAGCGATTGGTTTTGTGTTTGGGCACACACTGATTGAGGTATCAGGAGACAGTTTGTCAGTACATCTGCACGTCTGTTCATTGTAGCCTAGAGTTGCAGGACAGGCCATGCGCACCCAGCCATTTCCGGTGAACTGCATGAATCCCGTGTTGTCTCCAATGATGGCTTTCTTGTCAGTGCAGATTTCATGGTTGCCATCAAAACCTCCAGTTATGTCGGTACAACCACACGTAACACTGTTGTACAATCTGCGTTCAGGGCAAGCTACACGCGTCCAGTTTGCTCCATTGAAGATGCTATATGCGGCCCAGTCTCCAGGGATTGCTTTGTGATTTGGGCAGTTGTTCACTGGTGTGCACTTGACATTGTCCTGAAAGTCACAGACCAGCTTGTCCTGGTTCCAGTAGTTGCCGCTTGGGCAGCGTCTGATGTCAACTGCTGTAGGCAGAGCTCCATAGAAGCGGCACTGCACGTAGGCGTCACAGTATCCGGGCAACGGGGCATAACCAACACCATGATGAACCACACAGTTATTGCAGAGTTCTAGACTTGGCTTCACCGTTGTGATAGCTCCAATTGTAGTTGTTGTCGTGGTGGTAGTTACAGGCTCCGCTTCTGTGGTTGTAGTTGTGGCTTCTGGGGTGGTTATCACTTCAGTTGTTGTAGTTGTAGTTGTGGTGGTGGTAGTTGTAGTTTCGGTAGTAGTAGCTGGTGCCAGAGTTGTAGTTGATGTTGTAGTCTCTGTGGAATCCTCGTCGCTATTATCGTCACCATCGGTATCATCAGAGTCTTGCTTCTCTTCTTCGGAGTCCGCAGGCGTTGTTGTCTGTTGTTCCTGTTGTGTAGTGTCTTCTTCCGAGTCTGCAGGCGTTGTTGTCTGTTCTTCCTGTTGTGTAGTGTCTTCTTCGGAGTCTGCAGGCGTTGTTGTCTGTTGTTCTAGTGTGGTATCTTCTTCTGAGTTTGCAGGCGTTGTTGTCTGTTGTTCTTGTTGCGTAGTTTGTTCCTCAGAGTCTTCAGGCGTTATTGTCTGTTGTTCTTGTTTCACTTGCTCCACAGATTCAGCAGGAGTTGTCTGTTGTTCTTGGGTTACTTGTTCCTCGGAGGAGTCTTCTTTGGCTTGTTCCTCAGAGTCAGCAGGCGTTGTTGTCTGTTGCTCTTCTTGAGTAACATTTTCTTCCGAGTCTTTAGGAAAGAAATGATCCACTATTTCCTGTTTCAGGTCGTCATTCACAGCTGTTGGTTTCATTTTATTAAGCAGAGTCTTGATGTAGTCCAGTTGATCTTGTGTGAAGTAATGTGTTTGTTGTTGCTGTCCAGCATCAGATTGATACTGCTTAACCAATGACTCCAGCCAGGTTTCAATTTCCTGGTCGTCGGCTCCAGTTTGCTGGTCATAAGCAGCGGTCATTGGGGACAGAAAAACCAGCAAACAACTTGCAAGAGCAAATTGGGCCAGTGGGTATCTCAGCATAGCCATACTTAGATTAATTATGCTAGTTATTTTATTTCTTATCTTTAATAACGTTCTTTCGTAGTTTACTTGCTTGTAAACTGTTTTGAAAATAGGATTTGCTCTTTTCTTTAATAGTTCAGCTGGCGAGAATTATCTGAAGTATCTCGAAAAAAATAAGTTTTTTCTTCTTTTCTTTTAAGAATTGAAAATTATAAATTTCTCTTTTACTTTTTTTTTTTTAAAGAGTTGAAAATTATAAAATAAATTCTAAGTTAGCTCTAGTTACTCGCCAGACCGAAGCTTATGTTC

>Cnem_R37577449 [organism=Cepaea nemoralis] len=2200 num_reads=4834 avg_cov=229.2 contig_cov=99.8%

TTTGACGACTATTGAATGAGTTGTAATTCTGTTCTGTATCCGTGAATGCACAAACCAGAAATAATATTGTAGTTCTGTATCCGTGAAGGCACCAACTAGAATTAATACTGTAGTTCTGTATTCGTGAATTCACAAGTCACAAATAATCGACAAGTGAATGGTTTGTCTTGACCGTGTTAAAAAGTGATTCTTGCCGGAGTTGAATATTGATCACCTACTGTTTGTCGAACGATTTAGTTCGTACATTCGTTGTCTTCTGTCCGCCATCAGTGGCTAAACCAGTTTTGGACATGGATTCTGCGGTCTTCTTGCTGGCTGTGGTTGCTTCACTGGGTCAGTGTGTGTATCCAGACATCTACGCCAGGAGAGGTGACCTCGGTGACAGCACAGGCAGTCTTGACTTTGTCAGTGAAGCAGACCTGGAGCATTGTTCCAGACTGACCTATGACCAGCTGCGCTATCGGCAGATCGACGGTCGGTGCAACCATCCTAGGAACTACGGCAGTACAGGGAGGCCGGTCAAGCGCTACCTGAGGCCTCACTACCAGGACAAGTTTGGTGAGAACTTGCCACGTGTTTACAGTGTGACCGGCCAACTGTTACCTTCCCCGAGGATGGTCAGCTGGAAGCTACACCCCGACCAGACGGCCCACGACAACAACACCATGCTGGTCATGCAGATGGGCCAGTTCATCGACCATGACATCACACGCGCTCCCGAGTTGTCAGGGAGGAACGCCTCTATCAAATGTTGTGGAGTACCACCGAAAGAGCGTCTACCAGATTGCTTCCCCATCGATATCCCGCCCGGCGACCCGGTGTTTGAGGACTGCATGGAGTTTTTCAGATCCAGTCCCGCTGTGGACAATGATGGCAACATTATCTATCCCAGGGAACAGATCAACGCACTGACCTCATTCATTGACGGGTCAGCGGTCTATGGATCTGACCTGGACACTTACACGTGGATTCGATCAGAAAATGGAACAGGTGTATTTCTCAACACCCACCTGGTGCACGGGCGAGAGCGACTTCCCTCGCACCCACACCTGGGTCCCGAGAGCTGTGTGTCCTCCAACACTGCAGAATCGTATTGTCAGTTGGCAGGCGATATGCGAGTGAACGAACAGCCAGGGTTAGGATCAATCCATCTGCTCTTCCATCTTCACCACAATCACATAGTTCGACTTCTTGTAGCTGGTATCCTTAAGAAAAGAGGACAGCCCTCGTCTCCTGAAAGAATTGCAAAGTTTATTCAAGAATCGTCCAGCGCTTTGAAAGAACAGATTTTCCAGGAAGTCAGGAAAATGCTCGGAGCAATTATCCAAAAATTAACCTACTGCGATTGGCTACCAATGATCCTGGGACCTTACCTCATTGACAAGTTTCAGCTGGGCTGTACCAGACGCAGCCGGTATAATTCAGACCTTGACCCTAGAGTTGCAAACAGCTTCCTGTCAGCCGCTTTACGGTTTGGGCATACTCTCATTCCCAACGTGTACAATTTTGGAGACAAGAGAATTCACCTCAAGGACACATTTAACATTCCGGATGCCAGTATTCGTTACTATGACAACATCATCCAGTGCCTGATTAAGGAGGGCAGCGAGGAAGCCTATGATAGATATGTCAGCAGTGCTGTCAGCGAACATCTGTTTGAGTCTACCAGGGGCCACAAACATGCCTTGGACCTCATCGCTGTCAACATCCAGAGAGGGCGGGATCACGGTATACCAGCCTACCACTACTGGAGGCAGTACTACCGTCTGAGACGAATCATAAGCCTTGACGAGTTCGGCGAGGCAGGGATAGCTATGAAGAAAGCCTACAGGGACATCAGAGACGTGGACTTGTTTCCGGGCGGCTTGCTGGAGCCCTCCATGCCAGGGGGCGTGGTCGGGGAGACGTTTGGTCACATCCTGGCCAACCAGTTCGCAGATCTGAAGTTTGGCGACACCTATTTCTTTCTGCACCAACAGGCTCCCCAGGGTTTTCGCGCCGCTCAGATCAAGGCCATCCTCAGTGTGACCATGTCCAGTATCATCTGCGCTAACTCGGCCGTGACTCAGGCCCAGCCCGACCCCTTCTACATGGCTTCACAACTCAACCTTCCCCGGCCGTGTTCAGATTACTCCGAGATGGACGTGGAGCCATGGCTGATTCACTTTAGCGACTGAGTACTGGGGTGTGGAGTACTGGA

>Cnem_R27073283 [organism=Cepaea nemoralis] TransAbyss assembly 2 (filtered min reads 10, dedupe95) len=1878 num_reads=1258727 avg_cov=63568.7 contig_cov=100.0% (contig_1265 from old CLC assemly 9)

GTAGTAGTAGTAGTAGTAGTAGTAGTAGTAGGAGGAGGAGGAGGAGAAGGATGAGTATAAGGTGGACAAACAGAAAAAGAAGAATGAGGGGAGTGAGGAGGGGGAGGAGAAGACACACGGGTGTTGTAAAGAAGATGGAACGCCTACGCCACAAGACAAAACACTTGATGATGATGATTTTCATTACTTTCAGTCTTGCTTTTTTCCAGTGATATCTTTTATTTGTTGTATGTACTTTGTGCTCCGTGCCGGCCCAATTAATTCTAAATACCGAAACATTGCTAGAAGTGAGAATCTACCTTCAACGAGAGCTAGTTGCTGTCACTCGCTCACGTTTTCTATCCAATCAGTGTATGCAACAACTCCACCAAGTCTATCGCTCCGGAAAACGTTGAGCCATTCCACGGATTTTTTTCTGGTGGTTAAAAACGAGCCAAACCTTTTATCCCGAAGAGATCTACAGGAACGTCGCCAAATCTGAAGACATCTTTGTAGCTGGCGTCTGGTGACCTCTTTCTTTTCTTCTTCCCTGGAAGAGTATCGCCAAAGAGCCCGTTTCCATTTTGCTTCTTGGCCGAGCCGGGCGAGGCTGAGTTGTAGAGCAGAAGTGGGGCAACAGATTTAATGAAATCTCCTGGTTCCCTGCCTGGTGGTGGCTGGAAGCCTGGAGGTAGGAAGGACATCAGGGTGCTCCCAAATGCTCTTTTCATGACATCGCCTCCAAATTCTAACCTTTTGCCGGTGTTTGGATCCAGTGCGGTGCCTTGGAATATACTGAACAAAGACGGAGTTGCTGGGAACTCGGAAGGTTCCGTTACAAACATAAATCTTTTCTTTCTCAATTTTCCTTCGGCTAAGGCTTCAGGTCCCATTCCAGCTTCATTGTATGCTATGTAGTTTTCCACTTCTCGTTTTTTCTTCCCCGGGTAGCTATCGCCATACATTCCTGAACCGAAGCTCACCCGATCTTCGATGTAGCTATTTCGCCCAATGTTGCGGTATTCTTGGGCAACCCTAGACTTCTGCAGTTTGGTGAGCATCAGCAGATTGCTCATCATTTGAGCCGACTCGCTTATTTCTCTTCCTGGCCCAGGATTCGCACTTCCTAATGACATTGAAGTAAAGTCGGGAACCGGTACAGAAGATCCTGGGACAATGAAGCGTTTGTGTCGATACAAGACAGCCGAACCATCCTTATCGTTAGCGGCATGGCCCCTGTGAGTGTTTCGTCTTCCACGCAAGATCGCCTTTAGTTCATCGTCATCTGCAGTCAAAATCGATCTTTTGAGCCGATGTACTTCATTTTCGAAATTGCCGTCAACGACCTCTCGCGTGTGTCTTATCTTGGAGCTCATTGCGTAAATCCCCTTGGCGTCCATGGATGGACGGCTGATGTGTTTCCCATAGCTCCGTCGTTTACCACAGCTGGCAGGCATGTGGGACTCAACCCAATGTGGCAAGAGCTTCTTGAAGTTGTTTGGCCAGGCTTTCTTGTCGTAGACAAGGCGAAGCAACATCGCCAGTTCTCGCCTGAGTTCATCGCAAGCTCTCTCTTCGACAGTACGGCCCCCAGCAGGCGCCAGGACAGTGCTGTCACCGGGTGTCAGAGACATCAAGCAGATCAGCGTCGCATTCAGCAAGTAGACTTTCATCTTCCTATTTCTGCCTACGTCAGTTCTGCTTTCTGTATAGTGGCTTGTATGCGTTCAGAAGGCAGCTTTCCGTCACTGATGTAGAAGTTCTGGTCAAGGCGTTCAGCAACGTGTTTTTAAGATGTTGCCTGCTACCGTAAACCTCTCACCTTGCCGATGTGTACCAGCAAGCTTGACGAGGTTCTTCGGGCCCACAACTTCTACATCAGTGACGGAAAGCTGCCTT

>Cnem_R27072837 [organism=Cepaea nemoralis] TransAbyss assembly 2 (filtered min reads 10, dedupe95) len=2379 num_reads=1735589 avg_cov=69372.3 contig_cov=100.0% (contig_123 from old CLC assemly 9)

TTTTTTTTTTTTAGCTGTTGCAGTAAGGTTTATTCATGTTGAAAGAAGCAGCGCTGAGAACAATTGTCAGTCTACTGTAATAAAATCAGATGAAGCCGTTACAGTAACGCAAGCAACACGTGTTTGACTACTGGTAGCACGATTCTGTGAAACCGCCAGTCACTCAAGCATCATTACGCGCAACATGACTCTCCAACCAATCAGCGCTGGTACAATTTGGAAACTCATTAACTAATTTACATACCGTTAGATATAATCCATTATGTTGCTTTAAGATGTTTATTGTAATTAAGAAAATCCATTATTAAGCATACGTTATTGATTTATTAATGACACTAGTCCCAGTAATGTAATATTAAAGTAAATATCTGTAATGTACAATTATTTCCCAGAACTGACCACACTTGTACAGAAAAGACTATTTCTAAATTATTCGTCTAACATTTTCAGATTTCTGTTATTCATGCAATGTTTTCAGATTTCGTAAGACGCCATTTTCTGCCCAAGTTGAACAAGTCCTCTTGTCGACTCACATAATCAACTCATCCTTTGTGGAATCCGAACCGCCAAGACACGACTTCATTACGGAAACAACACCACATAGAATTCAAAATCACACAATTTCGTAGTTGTAGCTACTTTGTTGGCGGTACAATGTTATTTTTTACTTTTTATAACTAATTTAAGTCAAACAGTAATTGATTACCTAGTACACCGCTATGTTAACATATCCAGTCCTTATCTGCATGGTGTTTACTTGACACAAGTTGGTGATCCACACGCTCGCCCTTCAAACACCCTGACGGTTATGATAGTTGCCGCCAGTCAGTTTACCTCTGAATATGTCCCTGTCACTCATACTCTGTCGGCCATTAACATAAATGAAAATAATGAACATGTTTCCATGGCTGATCCCCTACACCAATTTGGCATATTCCCTAAAGATGAACATTTCTGCTGGTCGGAGATGAGAACTGTAATAGGAGGATGTTTCTGTAGCTCTTCTAAAAGTGAGTCTTGTTTAGGTACATTCGAGTTCTACGACCAGGTCCTGCACTTATACTCTCTCGTGATCTCTAATGCACTTATACCATCCCGTGACCTCTATAGATTCTTTAGCGACCGGCGAACGGGTTGTTGGCCGCTGGTCCTTGTTCCTTGGGGTCGCCCAGCAACGACATCAGCTCCCACTCCTTCGGTGTGGGTATCCTACTTGGTCTGGAGTTGACTTTCATCATTAAAATATTCTTGGCAGTCTCTTTGACTGTGTCAGGGAGACTTGTGTCCCACGGGTTCTTGGGCGGCACCCAGTTGTAACGAGGGGTCCCATCAGGAAAGTACAAAGCTTCTTGTAACTTCTGGTCTTTACAATGATCTGCAGTTGTGGGGCCCGGCGGGCAGTAGTGGTAGCTCCTCATGACAGGGGGACCCATGTACGGGCCACCGCCATATTGTGGGCCACCGCCATATTGTGGACCCCCACCGTACGGCGCCCCGCCTCCATAATGCGCCCCTCCTGCATGGGGCAAGTCCATGAAGAGACCGTCGTTCACTGTTCGGGGTTGTGGGGGACCCGGGGGTGCCATCTGCATGGGAGGGGGCAGGAATGGGCTGGATGGCTTCATGGGCATCGGCGGTGGCAGCATTGGCTTGAGGTCCGGCTTGTAGTTCTGTGGAACCGGGGCGAGGCCTCCAGCTCCCTGAGATAATGCCGTAGAGAACAATCCGACCAACACCAGCGTCAGTAAATTCATCATGGTACCACGTTTGCTAAAGTCGCTTCTGTAGAACTGGAAAGTTTTTCTCACACTTGATGTTCCCAAATGTGAATAAACAACCGCCGAGTTGCTACACTTCTCTCATAGATGTTGACAATAGTGACTTGACGTGCTGCAGCTTCGACGTACCAGCGCCAGCCCTTGGAGGAGATCTGAGGCAACTCGAAATGTTCCTGAGCATAAAACTAGACAGCGGCTGAAATAGGCGAAGTGAGGAGACGAAAAGGTCGTTCGTGACTGAAGATACTTGCCCGGCTAATCCAGTTTGTATAATTGTTTTAATCCTGTGTGCTCGTGGAGAAGCTGGCTGACAAGCGGAGATAAGTAGGTGTTTGTAGGCAACTCAAGGAATTTCAATTCCAATGCATATGTATTATATACATTTAATTTCATAACCCAATTATACAACAGAATGGATGAACAGGGCAATGATGAAACATAAGAAGTAGTGTCATTTCAATTCCCTTAAGGACATCCGGCTCCAAGACAAGATCAAAGTGTAGATTTATCAGCTCTTGATCTGGCCAGGGTGTAGGGGCGGTCATACAAACTGGAAACTCGAATCACAAAATCTCTGTCCACTAACACTGGCAGACGGGCC

>Cnem_R27075188 [organism=Cepaea nemoralis] TransAbyss assembly 2 (filtered min reads 10, dedupe95) len=1827 num_reads=212895 avg_cov=10763.9 contig_cov=100.0% (contig_7508 from old CLC assemly 9)

TAAGAAGGTAAGCGAGATGGAAATCAACTGGTATTTACTTCATCTCTAGTGGTACAACAGGTAGGAATCCTCTTGCCAGATGTTAGTGCAGAACAGGACAGGACATGACTCTATTTAAATAAAACAAGCATTACAAGATGACAAGAAATGTATGGCCCACTTGCCAGCCAAGAAGGTAACTGTCGTCACTTTGTTTTTTGAGCTGGCGTTTAAGCTTGTTGGAAAGTCGGCTGCACGCTGACGAGGTTGAATACATTTTACCAAGGGCTCAGTGTGCTGTCCGGAAGCCCTGGGAAAGACTACTTGCCGCAGCCGTGCATAGCAGAGCTGCATCCGCCACTGATAACTGCTTGTTGAGCGAATCGTTTCCCGAAGATCTCTGAAACAAAATGGCAACTGCAGGATTGATTGTGGCCGTTAGTAGTTTAGCGTGGTTCATTGCCGGGGTGGCGTCTCAAGCAACAGATGCTAGTAGTCACTGCAGCTACTTGATCAACTCTGTGTCCCGGTACCAGCCCAACGCAGCTGCCTTCAAGATCTACACCAGGTCAAGGTCGCCTCGGGTCACCAGTGGAGAACCGATTGAAGTAACCATCGGCCCGTTCAGCAGCTCCCTGAACTTCTTCAACTTCACCGACTTCATCTTGTATGCCACTCCATCCAACATTGCCAACCTGGAGGTCGAGTTTATCGGCCCCACCTCGCCACACGTCGGGGTCTTCCAGCTCTTCGACAAGTGGCGGGCGGGGGCTGGCGGGTTGAACTGCAACCCAAGGTCCAGGGCCGAGGACTCGGTGGGCGCATTCGAGGACCGGCTGTTGGCTACGTTCAAGCAGTACTACCCCAACAACCCCATGCTGCTGCGCTACCACGCCCCCGCCCGCAACCAGGTCTCTGTCTTATGGTGGCCCACCAAGGAAGCTTTGATGTATCCGGAGATTAAGTTTGTGGCCAACATTAAGTCCATGGGCAACTGGTTCAAGCTTCAGTCCACGCCCTGGAAAGTCAACAGACCCGTCGACCAGTGGGCAAATACGGAGAGTATGCTAGCCCAGTACCAAGCGATGCAGAACAACATGCGCGCCATGGAGCGGCGGTTGGAGCAGCCCATTTAGATTGGTTTAAAGCAAACCCAAACTACAAATACCTTTCTAGATCACGGACAAGATATTCTTTGAAAAATAAAGCAAAATATACATTAACGTAGACCCTCTCACGTAATTACAACTATAGCCAATATGCGTCAATATGCGTCTAAATTTATAATATGACAGGTTTTCTTTCGTCGAGAATAATTAATCACTGTTCGCGCTTATCCAGCTGTAACCATAATTCAAATGTTGGCATGCATATGCGGTGAGCTTAGTTTGATAAGAACGAAAGGTGATATATTATTTCTTAGTGTTGTGTTCCTGATGAAGAGATTGTCAATTTCCTTGAAGCACAGCGTGGATGTGGCATGGGTGCTACCTTTCGTGAAGGTCTTTGACCTTCGTGTGCTTAGGGGAGATGTTTCACAGGTCATTTGGGGGTGCGTAGAAATTGGAGAATTAAAATGTTAGCTTGCAATGCACCATCATTGGAACACTGGAGATGCACAGGAGGATATATTGCGAGGAATATATGCAAGCTTGTTGTATGTTTACACATCTCTTGATACGTGTTCTTTAACACGGTTGATGGAATGGGGAGTGGAGTAAAACATCTCTTGGTGGATGTTCTCTAACACGGCTGTTTGAGTGGAGAATCCAGCAATACATCTCGTGACACAAGTTCTATTACACGGCTGTTGGAGCAGGGAGTCCATTAACACAACTCTTCATGGAT

>Cnem_R37432942 [organism=Cepaea nemoralis] len=697 num_reads=360970 avg_cov=65037.1 contig_cov=100.0%

TCGAACATCAACTTTGCCCCGGAGGTGGTCGTGTTTTTCGGGGGATTAAGTTTATGATATTTGAAATGTTGTAATGGTCAGCCCGCGGGTTATGAAGGGTTGTTGTCTAAGGTGTGGCCAGGTGAAGATCTCCTATAAAAGGGTCGGCAACGATGTAACCAGCACGGATTTCCATACAGTCAACCGGTACTCTCGTCTGCTAGCCGCTGTTACCGACATTTAACATCATGTACAAGCCAGTAGCTGTCCTCATGCTGGTAGCCACTCTCTCCATGAGTGCCGAGGCTATTGTAGGCGGGTACGGAATCGGTTTCCCTGGATACGGCCTTGGTATCGGTAAATATGGATACGGTGGATATGGGATCGGTGGACTTGGACTTGGAGGCCTTGGGTATGGAGTTGGTGGTTATGGATATGGACTTGGTGGTTATGGATACGGACTAGGTGGCGCTGGATACGGACTAGGTGGCATTGGAGTTGGATATGGAATTGGTGGCATTTACGGCAAAGGGCTGTACTATTAAAGGGCACAGAGGACTGGTGGAAACATCGAGAATTTTCTGAGAAACTACCATCCTGGGTTATGCTAATGCGTTCACGTGCTATGGATACGGCCCTTGGTGACGGTACAACACAGAGAAGCACGCCGTGTTTATAAATAAATGACACATGTCTAACTAGTAAAAAAAAAAAAAAA

>Cnem_Gly_rich2 [organism=Cepaea nemoralis]

CAAGGACTGTCTTGTACATGATGTCAGAGTTTTGGATCTTCACCGCAATCTACCACCAGACGTTGTCAGTACCAAGATCCAAAACTCTGACATCATGTACAAGACAGTCCTTGTAGTTGCTTTCATTGCCGCCGCTGTATTGACAGTTCAAGGCGGAGGTTACGGAGGAGGAGGTCTAGGCCTTGGTGGCGTCATTGTTGGAGGTTTAGGCGGTTATGGCGGTGGTTACGGCGGCGGTTACGGTAGACAAGGCGTTGCACTCACTGTTGCTCAACCTCTTGTAGTTGGAGGTTACGGTGGTGGCTACGGAAGCGGTTATGGAGGTGGTTATGGAGGAGG
