# Supplementary material for: Mantle Modularity Underlies the Plasticity of the Molluscan Shell: Supporting Data From Cepaea nemoralis
Source: Front Genet. 2021 Feb 5;12:622400. doi: 10.3389/fgene.2021.622400 (PMC7894901; doi:10.3389/fgene.2021.622400)
Supplement: Supplementary file 8 [file Data_Sheet_8.docx]

>Cnem_R27072766 TransAbyss assembly 2 (filtered min reads 10, dedupe95) len=2716 num_reads=5670257 avg_cov=209491.4 contig_cov=100.0% (contig_821 from old CLC assemly 9) cds start = 286 cds stop = 2466 strand = - protein length = 727 strand = +

MAMLRYPLAQFALASCLLVFLSPMTAAYDQQTGADDQEIETWLESLVKQYQSDAGQQQQTHYFTQDQLDYIKTLLNKMKPTAVNDDLKQEIVDHFFPKDSEENVTQEEQQTTTPADSEEQAKEDSSEEQVTQEQQTTPAESVEQVKQEQQTITPEDSEEQTTQQEQQTTTPANSEEDTTLEQQTTTPADSEEDTTQQEEQTTTPADSEEDTTQQEQQTTTPADSEEEKQDSDDTDGDDNSDEDSTETTTSTTTLAPATTTETTTTTTTTTTTTTEVITTPEATTTTTEAEPVTTTTTTTTIGAITTVKPSLELCNNCVVHHGVGYAPLPGYCDAYVQCRFYGALPTAVDIRRCPSGNYWNQDKLVCDFQDNVKCTPVNNCPNHKAIPGDWAAYSIFNGANWTRVACPERRLYNSVTCGCTDITGGFDGNHEICTDKKAIIGDNTGFMQFTGNGWVRMACPATLGYNEQTCRCTDKLSPDTSISVCPNTKPIAGDKSGYLQFTGVSWIRRPCPATLVYHADICVCSYDQTNVVDDDDNKSKQHGVCKATVALNFDNNNATDSSVNHFWVNNTGVTFNDGKAYFNGKSRLTIPGLSNMEFGSTVYILIKYRHSSANSQQTLVSNGDCQVRQSLAVCSGKDSVDFYAETKEQISLGKTTVPTDVGAWQYALYALDNGNLLGSVGVNKIAQPVKGALDRRQRGLVIGGGGGCDNFHGIIDDVRVYLCKPEL

>Cnem_R37577449 len=2200 num_reads=4834 avg_cov=229.2 contig_cov=99.8% ORF=628

MDSAVFLLAVVASLGQCVYPDIYARRGDLGDSTGSLDFVSEADLEHCSRLTYDQLRYRQIDGRCNHPRNYGSTGRPVKRYLRPHYQDKFGENLPRVYSVTGQLLPSPRMVSWKLHPDQTAHDNNTMLVMQMGQFIDHDITRAPELSGRNASIKCCGVPPKERLPDCFPIDIPPGDPVFEDCMEFFRSSPAVDNDGNIIYPREQINALTSFIDGSAVYGSDLDTYTWIRSENGTGVFLNTHLVHGRERLPSHPHLGPESCVSSNTAESYCQLAGDMRVNEQPGLGSIHLLFHLHHNHIVRLLVAGILKKRGQPSSPERIAKFIQESSSALKEQIFQEVRKMLGAIIQKLTYCDWLPMILGPYLIDKFQLGCTRRSRYNSDLDPRVANSFLSAALRFGHTLIPNVYNFGDKRIHLKDTFNIPDASIRYYDNIIQCLIKEGSEEAYDRYVSSAVSEHLFESTRGHKHALDLIAVNIQRGRDHGIPAYHYWRQYYRLRRIISLDEFGEAGIAMKKAYRDIRDVDLFPGGLLEPSMPGGVVGETFGHILANQFADLKFGDTYFFLHQQAPQGFRAAQIKAILSVTMSSIICANSAVTQAQPDPFYMASQLNLPRPCSDYSEMDVEPWLIHFSD

>Cnem_R27073283 TransAbyss assembly 2 (filtered min reads 10, dedupe95) len=1878 num_reads=1258727 avg_cov=63568.7 contig_cov=100.0% (contig_1265 from old CLC assemly 9) cds start = 227 cds stop = 1453 strand = - protein length = 409 strand = +

MKVYLLNATLICLMSLTPGDSTVLAPAGGRTVEERACDELRRELAMLLRLVYDKKAWPNNFKKLLPHWVESHMPASCGKRRSYGKHISRPSMDAKGIYAMSSKIRHTREVVDGNFENEVHRLKRSILTADDDELKAILRGRRNTHRGHAANDKDGSAVLYRHKRFIVPGSSVPVPDFTSMSLGSANPGPGREISESAQMMSNLLMLTKLQKSRVAQEYRNIGRNSYIEDRVSFGSGMYGDSYPGKKKREVENYIAYNEAGMGPEALAEGKLRKKRFMFVTEPSEFPATPSLFSIFQGTALDPNTGKRLEFGGDVMKRAFGSTLMSFLPPGFQPPPGREPGDFIKSVAPLLLYNSASPGSAKKQNGNGLFGDTLPGKKKRKRSPDASYKDVFRFGDVPVDLFGIKGLARF

>Cnem_R27072837 TransAbyss assembly 2 (filtered min reads 10, dedupe95) len=2379 num_reads=1735589 avg_cov=69372.3 contig_cov=100.0% (contig_123 from old CLC assemly 9) cds start = 623 cds stop = 1261 strand = - protein length = 213 strand = +

MMNLLTLVLVGLFSTALSQGAGGLAPVPQNYKPDLKPMLPPPMPMKPSSPFLPPPMQMAPPGPPQPRTVNDGLFMDLPHAGGAHYGGGAPYGGGPQYGGGPQYGGGPYMGPPVMRSYHYCPPGPTTADHCKDQKLQEALYFPDGTPRYNWVPPKNPWDTSLPDTVKETAKNILMMKVNSRPSRIPTPKEWELMSLLGDPKEQGPAANNPFAGR

>Cnem_R27075188 TransAbyss assembly 2 (filtered min reads 10, dedupe95) len=1827 num_reads=212895 avg_cov=10763.9 contig_cov=100.0% (contig_7508 from old CLC assemly 9) cds start = 390 cds stop = 1112 strand = + protein length = 241 strand = +

MATAGLIVAVSSLAWFIAGVASQATDASSHCSYLINSVSRYQPNAAAFKIYTRSRSPRVTSGEPIEVTIGPFSSSLNFFNFTDFILYATPSNIANLEVEFIGPTSPHVGVFQLFDKWRAGAGGLNCNPRSRAEDSVGAFEDRLLATFKQYYPNNPMLLRYHAPARNQVSVLWWPTKEALMYPEIKFVANIKSMGNWFKLQSTPWKVNRPVDQWANTESMLAQYQAMQNNMRAMERRLEQPI

>Cnem_Gly_rich2

MYKTVLVVAFIAAAVLTVQGGGYGGGGLGLGGVIVGGLGGYGGGYGGGYGRQGVALTVAQPLVVGGYGGGYGSGYGGGYGG

>Cnem_R37432942 len=697 num_reads=360970 avg_cov=65037.1 contig_cov=100.0% ORF=98

MYKPVAVLMLVATLSMSAEAIVGGYGIGFPGYGLGIGKYGYGGYGIGGLGLGGLGYGVGGYGYGLGGYGYGLGGAGYGLGGIGVGYGIGGIYGKGLYY*
