# Supplementary material for: Mantle Modularity Underlies the Plasticity of the Molluscan Shell: Supporting Data From Cepaea nemoralis
Source: Front Genet. 2021 Feb 5;12:622400. doi: 10.3389/fgene.2021.622400 (PMC7894901; doi:10.3389/fgene.2021.622400)
Supplement: Supplementary file 9 [file Data_Sheet_9.docx]

**BLASTP 2.10.1+**

[**Reference**](https://www.ncbi.nlm.nih.gov/entrez/query.fcgi?db=PubMed&cmd=Retrieve&list_uids=9254694&dopt=Citation)**:**

Stephen F. Altschul, Thomas L. Madden, Alejandro A. Schäffer,

Jinghui Zhang, Zheng Zhang, Webb Miller, and David J. Lipman (1997),

"Gapped BLAST and PSI-BLAST: a new generation of protein database

search programs", Nucleic Acids Res. 25:3389-3402.

**[Reference for](https://www.ncbi.nlm.nih.gov/entrez/query.fcgi?db=PubMed&cmd=Retrieve&list_uids=11452024&dopt=Citation)**

**[composition-based statistics](https://www.ncbi.nlm.nih.gov/entrez/query.fcgi?db=PubMed&cmd=Retrieve&list_uids=11452024&dopt=Citation):**

Alejandro A. Schäffer, L. Aravind, Thomas L. Madden, Sergei

Shavirin, John L. Spouge, Yuri I. Wolf, Eugene V. Koonin, and

Stephen F. Altschul (2001), "Improving the accuracy of PSI-BLAST

protein database searches with composition-based statistics and

other refinements", Nucleic Acids Res. 29:2994-3005.

Database: Non-redundant UniProtKB/SwissProt sequences

473,533 sequences; 178,974,751 total letters

**Query=** R27072766 chitin binding Periotrophin-A TransAbyss assembly 2

(filtered min reads 10, dedupe95) len=2716 num_reads=5670257

avg_cov=209491.4 contig_cov=100.0% (contig_821 from old CLC assemly

9) cds start = 286 cds stop = 2466 strand = - protein length = 727

strand = +

Length=727

Score E

Sequences producing significant alignments: (Bits) Value

[P86860.1](https://www.ncbi.nlm.nih.gov/protein/P86860.1?report=genbank&log$=prottop&blast_rank=1&RID=) RecName: Full=Shell matrix protein [63.5](#322966920) 1e-09

[H2A0N4.1](https://www.ncbi.nlm.nih.gov/protein/H2A0N4.1?report=genbank&log$=prottop&blast_rank=2&RID=) RecName: Full=Protein PIF; Contains: RecName: Full=Prote... [58.9](#391359324) 1e-07

[C7G0B5.1](https://www.ncbi.nlm.nih.gov/protein/C7G0B5.1?report=genbank&log$=prottop&blast_rank=3&RID=) RecName: Full=Protein PIF; Contains: RecName: Full=Prote... [57.4](#269969412) 4e-07

[H2A0M0.1](https://www.ncbi.nlm.nih.gov/protein/H2A0M0.1?report=genbank&log$=prottop&blast_rank=4&RID=) RecName: Full=Asparagine-rich protein; AltName: Full=Pri... [55.1](#391359317) 2e-06

[Q9JM99.2](https://www.ncbi.nlm.nih.gov/protein/Q9JM99.2?report=genbank&log$=prottop&blast_rank=5&RID=) RecName: Full=Proteoglycan 4; AltName: Full=Lubricin; Al... [53.5](#83288394) 5e-06

>[P86860.1](https://www.ncbi.nlm.nih.gov/protein/P86860.1?report=genbank&log$=protalign&blast_rank=1&RID=0) RecName: Full=Shell matrix protein

Length=294

Score = 63.5 bits (153), Expect = 1e-09, Method: Compositional matrix adjust.

Identities = 64/248 (26%), Positives = 100/248 (40%), Gaps = 10/248 (4%)

Query 485 CPNTKPIAGDKSGYL--QFTGVSWIRRPCPATLVYHADICVCSYDQTNVVDDDDNKSKQH 542

CP+ P ++ GYL Q+ G+ R CP+ +Y + C + + KQ

Sbjct 49 CPSL-PDPMNRYGYLAPQYGGLRI--RACPSGTIYSENQCRYKSNMNGNGGLRGSARKQF 105

Query 543 GVCKATVALNFDNNNATDSSVNHFWVNNTGVTFNDGKAYFNGKSRLTIPGLSNMEFGSTV 602

C A +NFD+ D S + + ++ GK F G S+L I G + G T

Sbjct 106 RQCSAEFKINFDDG-FKDISKGGLAFDYSHISLRRGKGVFVGNSKLYIWGFQSRFLGKTF 164

Query 603 YILIKY---RHSSANSQQTLVSNGDCQVRQSLAVCSGKDSVDFYAETKEQISLGKTTVPT 659

I +K R + + ++SN S+ + + V F A+T + T

Sbjct 165 AIRMKVKIKRGAGKYRPEPIISNCGPNGDSSVEIVVHRGKVIFKAKTSDNPEAVFITEDY 224

Query 660 DVGAWQYALYALDNGNLLGSVGVNKIAQPVKGALDRRQRGLVIGGGGGCDNFHGIIDDVR 719

D W Y D + GS Q G L+ R + IG G + FHG ID++

Sbjct 225 DDDKWTDLTYYYDGNHFGGSCNGRPFRQRTGGNLEIRDNPMTIGLCTGQNGFHGEIDELE 284

Query 720 VY-LCKPE 726

+Y C P+

Sbjct 285 IYTACIPK 292

>[H2A0N4.1](https://www.ncbi.nlm.nih.gov/protein/H2A0N4.1?report=genbank&log$=protalign&blast_rank=2&RID=0) RecName: Full=Protein PIF; Contains: RecName: Full=Protein Pif97;

Contains: RecName: Full=Protein Pif80; AltName: Full=Aragonite-binding

protein; Flags: Precursor [Pinctada margaritifera]

Length=1014

Score = 58.9 bits (141), Expect = 1e-07, Method: Compositional matrix adjust.

Identities = 42/130 (32%), Positives = 63/130 (48%), Gaps = 6/130 (5%)

Query 485 CPNTKPIAGDKSGYLQFTGV-SWIRRPCPATLVYHADICVCSYDQTNVVDDD-DNKSKQH 542

CP +PI G + Q TG +W C ++ + C CS DD+ N+

Sbjct 412 CP-LRPIKGSPEKFKQHTGDDNWEEFDCAPGTLFSSRDCACSILGRPEKDDNGKNEDDTS 470

Query 543 GVCKATVALNFDNNNATDSSVNHFWVNNTG--VTFNDGKAYFNGKSRLTIPGLSNMEFGS 600

VC+ + L F ++ D S V N G V +GKAYFNG++ L IP S + +G

Sbjct 471 KVCEPELYLPF-CDDLHDYSGKETHVENEGDAVIIENGKAYFNGRAGLKIPRFSGVPYGK 529

Query 601 TVYILIKYRH 610

+V+I +KY+

Sbjct 530 SVFIKMKYKE 539

>[C7G0B5.1](https://www.ncbi.nlm.nih.gov/protein/C7G0B5.1?report=genbank&log$=protalign&blast_rank=3&RID=0) RecName: Full=Protein PIF; Contains: RecName: Full=Protein Pif97;

Contains: RecName: Full=Protein Pif80; AltName: Full=Aragonite-binding

protein; Flags: Precursor

Length=1007

Score = 57.4 bits (137), Expect = 4e-07, Method: Compositional matrix adjust.

Identities = 44/128 (34%), Positives = 63/128 (49%), Gaps = 6/128 (5%)

Query 485 CPNTKPIAGDKSGYLQFTGV-SWIRRPCPATLVYHADICVCSYDQT-NVVDDDDNKSKQH 542

CP +PI G + Q TG +W C ++ A C CS T D +D+ H

Sbjct 401 CP-LRPIKGHPEKFKQHTGDDNWEDFDCAPGTLFSARDCACSILGTAKKDDKNDDGGDAH 459

Query 543 GVCKATVALNFDNNNATDSSVNHFWVNNTG--VTFNDGKAYFNGKSRLTIPGLSNMEFGS 600

VC+ + L F ++ D S V N G V +GKAYFNG++ L IP S + +G

Sbjct 460 KVCEPELYLPF-CDDLHDYSGKETHVENEGDAVIIENGKAYFNGRAGLKIPRFSGVPYGK 518

Query 601 TVYILIKY 608

+V+I +KY

Sbjct 519 SVFIKMKY 526

>[H2A0M0.1](https://www.ncbi.nlm.nih.gov/protein/H2A0M0.1?report=genbank&log$=protalign&blast_rank=4&RID=0) RecName: Full=Asparagine-rich protein; AltName: Full=Prism uncharacterized

shell protein 1; Short=PUSP1; Flags: Precursor

[Pinctada margaritifera]

Length=686

Score = 55.1 bits (131), Expect = 2e-06, Method: Compositional matrix adjust.

Identities = 58/244 (24%), Positives = 98/244 (40%), Gaps = 28/244 (11%)

Query 494 DKSGYLQFTGVSWIRRPCPATLVYHADICVCSYDQTNVVDDDDNKSKQHGVCKATVALNF 553

D+ + Q W+ C ++ C+CS T C V LNF

Sbjct 452 DRRYFRQLVNGKWLNLKCADGAGFNETTCLCSIHLTG-----------DAQCSPEVRLNF 500

Query 554 DNNNATDSSVNHFWVNNTGVTFNDGKAYFNGKSRLTIPGLSNMEFGSTVYILIKYRHSS- 612

++ + + + ++ GV + G A+FNG +++ + + I ++++ S

Sbjct 501 NDGTIQNLTPINVHIDAEGVDASKGWAHFNGSTQMKFEYFNAYDVQRDFLIKLRFKADSY 560

Query 613 -ANSQQTLVSN---GDCQVRQSLAV-CSGK--DSVDFYAETKEQISLGKTT--VPTDVGA 663

N +V+N G S+ V +G + F +T + L VP D

Sbjct 561 IPNQSHPIVTNCVAGQENTDPSIGVFLTGNYPHKIVFILQTDKSKLLQHLIFDVPRD--G 618

Query 664 WQYALYALDNGNLLGSVGVNKIAQPVKGALDRRQRGLVIGGGGGCDN--FHGIIDDVRVY 721

W Y D L G + + + P +G ++ RQ LV GGC N F G IDD+++Y

Sbjct 619 WHDITYKYDGSTLTGILDGKEKSLPTEGRIENRQAVLVF---GGCGNRIFRGNIDDIQIY 675

Query 722 LCKP 725

C P

Sbjct 676 TCIP 679

>[Q9JM99.2](https://www.ncbi.nlm.nih.gov/protein/Q9JM99.2?report=genbank&log$=protalign&blast_rank=5&RID=0) RecName: Full=Proteoglycan 4; AltName: Full=Lubricin; AltName:

Full=Megakaryocyte-stimulating factor; AltName: Full=Superficial

zone proteoglycan; Contains: RecName: Full=Proteoglycan

4 C-terminal part; Flags: Precursor

Length=1054

Score = 53.5 bits (127), Expect = 5e-06, Method: Compositional matrix adjust.

Identities = 42/124 (34%), Positives = 63/124 (51%), Gaps = 3/124 (2%)

Query 102 ENVTQEEQQTTTPADSEEQAKEDSSEEQVTQEQQTTPAESVEQVKQEQQTITPEDSEEQT 161

E T +E + TTP + E ++ + + TTP E +E + TP++ E T

Sbjct 478 EPTTPKEPEPTTPKEPEPTTPKEPEPTTPKEPEPTTPKEPEPTTPKEPEPTTPKEPE-PT 536

Query 162 TQQEQQTTTPANSEEDTTLEQQTTTPADSEEDTTQQEEQTTTPADSEEDTTQQEQQTTTP 221

T +E + TTP E T E TTP + E TT +E + TTP + E TT++E + TTP

Sbjct 537 TPKEPEPTTPKKPEPTTPKEPVPTTPKEP-EPTTPKEPEPTTPKEP-EPTTRKEPEPTTP 594

Query 222 ADSE 225

+ E

Sbjct 595 KEPE 598

Lambda K H a alpha

0.311 0.126 0.375 0.792 4.96

Gapped

Lambda K H a alpha sigma

0.267 0.0410 0.140 1.90 42.6 43.6

Effective search space used: 72109441852

**Query=** R27073283 TransAbyss assembly 2 (filtered min reads 10, dedupe95)

len=1878 num_reads=1258727 avg_cov=63568.7 contig_cov=100.0%

(contig_1265 from old CLC assemly 9) cds start = 227 cds stop = 1453

strand = - protein length = 409 strand = +

Length=409

***** No hits found *****

Lambda K H a alpha

0.318 0.136 0.401 0.792 4.96

Gapped

Lambda K H a alpha sigma

0.267 0.0410 0.140 1.90 42.6 43.6

Effective search space used: 35301578599

**Query=** R27072837 TransAbyss assembly 2 (filtered min reads 10, dedupe95)

len=2379 num_reads=1735589 avg_cov=69372.3 contig_cov=100.0%

(contig_123 from old CLC assemly 9) cds start = 623 cds stop = 1261

strand = - protein length = 213 strand = +

Length=213

***** No hits found *****

Lambda K H a alpha

0.315 0.140 0.461 0.792 4.96

Gapped

Lambda K H a alpha sigma

0.267 0.0410 0.140 1.90 42.6 43.6

Effective search space used: 12719844555

**Query=** R27075188 TransAbyss assembly 2 (filtered min reads 10, dedupe95)

len=1827 num_reads=212895 avg_cov=10763.9 contig_cov=100.0%

(contig_7508 from old CLC assemly 9) cds start = 390 cds stop = 1112

strand = + protein length = 241 strand = +

Length=241

***** No hits found *****

Lambda K H a alpha

0.320 0.131 0.406 0.792 4.96

Gapped

Lambda K H a alpha sigma

0.267 0.0410 0.140 1.90 42.6 43.6

Effective search space used: 15873982603

**Query=** Cnem_R37432942 Gly_rich3 len=697 num_reads=360970 avg_cov=65037.1

contig_cov=100.0% ORF=98

Length=99

***** No hits found *****

Lambda K H a alpha

0.325 0.163 0.530 0.792 4.96

Gapped

Lambda K H a alpha sigma

0.267 0.0410 0.140 1.90 42.6 43.6

Effective search space used: 4389029220

**Query=** Cnem_Gly_rich2

Length=81

***** No hits found *****

Lambda K H a alpha

0.318 0.154 0.480 0.792 4.96

Gapped

Lambda K H a alpha sigma

0.267 0.0410 0.140 1.90 42.6 43.6

Effective search space used: 4476180015

**Query=** Cnem_R37577449 Peroxidase len=2200 num_reads=4834 avg_cov=229.2

contig_cov=99.8% ORF=628

Length=628

Score E

Sequences producing significant alignments: (Bits) Value

[Q7QH73.3](https://www.ncbi.nlm.nih.gov/protein/Q7QH73.3?report=genbank&log$=prottop&blast_rank=1&RID=) RecName: Full=Chorion peroxidase; Flags: Precursor [267](#93140516) 2e-78

[P11247.2](https://www.ncbi.nlm.nih.gov/protein/P11247.2?report=genbank&log$=prottop&blast_rank=2&RID=) RecName: Full=Myeloperoxidase; Short=MPO; Contains: RecN... [263](#341941245) 2e-77

[P82600.3](https://www.ncbi.nlm.nih.gov/protein/P82600.3?report=genbank&log$=prottop&blast_rank=3&RID=) RecName: Full=Chorion peroxidase; Flags: Precursor [258](#166208492) 5e-75

[A4IGL7.1](https://www.ncbi.nlm.nih.gov/protein/A4IGL7.1?report=genbank&log$=prottop&blast_rank=4&RID=) RecName: Full=Peroxidasin; Flags: Precursor [251](#172044151) 3e-70

[P49290.2](https://www.ncbi.nlm.nih.gov/protein/P49290.2?report=genbank&log$=prottop&blast_rank=5&RID=) RecName: Full=Eosinophil peroxidase; Short=EPO; Contains... [243](#341941244) 6e-70

[P11678.2](https://www.ncbi.nlm.nih.gov/protein/P11678.2?report=genbank&log$=prottop&blast_rank=6&RID=) RecName: Full=Eosinophil peroxidase; Short=EPO; Contains... [243](#1352738) 6e-70

[P05164.1](https://www.ncbi.nlm.nih.gov/protein/P05164.1?report=genbank&log$=prottop&blast_rank=7&RID=) RecName: Full=Myeloperoxidase; Short=MPO; Contains: RecN... [243](#129825) 1e-69

[Q9VEG6.3](https://www.ncbi.nlm.nih.gov/protein/Q9VEG6.3?report=genbank&log$=prottop&blast_rank=8&RID=) RecName: Full=Chorion peroxidase; AltName: Full=Peroxine... [241](#229462993) 1e-68

[Q3UQ28.2](https://www.ncbi.nlm.nih.gov/protein/Q3UQ28.2?report=genbank&log$=prottop&blast_rank=9&RID=) RecName: Full=Peroxidasin homolog; Flags: Precursor [244](#341941778) 6e-68

[Q92626.2](https://www.ncbi.nlm.nih.gov/protein/Q92626.2?report=genbank&log$=prottop&blast_rank=10&RID=) RecName: Full=Peroxidasin homolog; AltName: Full=Melanom... [237](#172045828) 2e-65

[Q8R481.1](https://www.ncbi.nlm.nih.gov/protein/Q8R481.1?report=genbank&log$=prottop&blast_rank=11&RID=) RecName: Full=Lactoperoxidase; Short=LPO; AltName: Full=... [226](#81901688) 7e-64

[P35419.1](https://www.ncbi.nlm.nih.gov/protein/P35419.1?report=genbank&log$=prottop&blast_rank=12&RID=) RecName: Full=Thyroid peroxidase; Short=TPO; Flags: Prec... [225](#548478) 2e-62

[Q9VZZ4.1](https://www.ncbi.nlm.nih.gov/protein/Q9VZZ4.1?report=genbank&log$=prottop&blast_rank=13&RID=) RecName: Full=Peroxidasin; Flags: Precursor [226](#74871953) 6e-62

[P14650.1](https://www.ncbi.nlm.nih.gov/protein/P14650.1?report=genbank&log$=prottop&blast_rank=14&RID=) RecName: Full=Thyroid peroxidase; Short=TPO; Flags: Prec... [220](#129832) 1e-60

[Q23490.1](https://www.ncbi.nlm.nih.gov/protein/Q23490.1?report=genbank&log$=prottop&blast_rank=15&RID=) RecName: Full=Peroxidase mlt-7; AltName: Full=Molting de... [216](#74966834) 3e-60

[P22079.2](https://www.ncbi.nlm.nih.gov/protein/P22079.2?report=genbank&log$=prottop&blast_rank=16&RID=) RecName: Full=Lactoperoxidase; Short=LPO; AltName: Full=... [216](#12643419) 5e-60

[H2A0M7.1](https://www.ncbi.nlm.nih.gov/protein/H2A0M7.1?report=genbank&log$=prottop&blast_rank=17&RID=) RecName: Full=Peroxidase-like protein; Flags: Precursor [217](#391359325) 8e-60

[A0A452E9Y6.1](https://www.ncbi.nlm.nih.gov/protein/A0A452E9Y6.1?report=genbank&log$=prottop&blast_rank=18&RID=) RecName: Full=Lactoperoxidase; Flags: Precursor [215](#1746696558) 8e-60

[A8WQH2.1](https://www.ncbi.nlm.nih.gov/protein/A8WQH2.1?report=genbank&log$=prottop&blast_rank=19&RID=) RecName: Full=Peroxidasin homolog; Flags: Precursor [220](#172048149) 9e-60

[P09933.1](https://www.ncbi.nlm.nih.gov/protein/P09933.1?report=genbank&log$=prottop&blast_rank=20&RID=) RecName: Full=Thyroid peroxidase; Short=TPO; Flags: Prec... [218](#129831) 9e-60

[P80025.1](https://www.ncbi.nlm.nih.gov/protein/P80025.1?report=genbank&log$=prottop&blast_rank=21&RID=) RecName: Full=Lactoperoxidase; Short=LPO; Flags: Precursor [213](#129823) 6e-59

[Q1ENI8.1](https://www.ncbi.nlm.nih.gov/protein/Q1ENI8.1?report=genbank&log$=prottop&blast_rank=22&RID=) RecName: Full=Peroxidasin homolog; Flags: Precursor [217](#122119348) 7e-59

[Q8HYB7.2](https://www.ncbi.nlm.nih.gov/protein/Q8HYB7.2?report=genbank&log$=prottop&blast_rank=23&RID=) RecName: Full=Thyroid peroxidase; Short=TPO; Flags: Prec... [215](#408360185) 1e-58

[A5JUY8.1](https://www.ncbi.nlm.nih.gov/protein/A5JUY8.1?report=genbank&log$=prottop&blast_rank=24&RID=) RecName: Full=Lactoperoxidase; Short=LPO; Short=WBLP; Fl... [210](#576011186) 6e-58

[A1KZ92.3](https://www.ncbi.nlm.nih.gov/protein/A1KZ92.3?report=genbank&log$=prottop&blast_rank=25&RID=) RecName: Full=Peroxidasin-like protein; AltName: Full=Ca... [211](#317373515) 7e-57

[Q01603.2](https://www.ncbi.nlm.nih.gov/protein/Q01603.2?report=genbank&log$=prottop&blast_rank=26&RID=) RecName: Full=Peroxidase; Short=DmPO; AltName: Full=Chor... [206](#290457651) 2e-56

[P07202.4](https://www.ncbi.nlm.nih.gov/protein/P07202.4?report=genbank&log$=prottop&blast_rank=27&RID=) RecName: Full=Thyroid peroxidase; Short=TPO; Flags: Prec... [196](#160281455) 3e-52

[B3A0Q8.1](https://www.ncbi.nlm.nih.gov/protein/B3A0Q8.1?report=genbank&log$=prottop&blast_rank=28&RID=) RecName: Full=Peroxidase-like protein 3 [174](#374110746) 3e-48

[P90820.3](https://www.ncbi.nlm.nih.gov/protein/P90820.3?report=genbank&log$=prottop&blast_rank=29&RID=) RecName: Full=Heme peroxidase 2; Contains: RecName: Full... [181](#74961220) 9e-48

[Q20616.1](https://www.ncbi.nlm.nih.gov/protein/Q20616.1?report=genbank&log$=prottop&blast_rank=30&RID=) RecName: Full=Peroxidase skpo-1; AltName: Full=ShKT and ... [173](#74964722) 5e-45

[B3A0P3.1](https://www.ncbi.nlm.nih.gov/protein/B3A0P3.1?report=genbank&log$=prottop&blast_rank=31&RID=) RecName: Full=Peroxidase-like protein 2 [157](#374110745) 4e-39

[Q6TMK4.1](https://www.ncbi.nlm.nih.gov/protein/Q6TMK4.1?report=genbank&log$=prottop&blast_rank=32&RID=) RecName: Full=Peroxinectin A; Flags: Precursor [143](#74914842) 3e-35

[Q9VQH2.2](https://www.ncbi.nlm.nih.gov/protein/Q9VQH2.2?report=genbank&log$=prottop&blast_rank=33&RID=) RecName: Full=Dual oxidase [130](#380865378) 3e-30

[O61213.2](https://www.ncbi.nlm.nih.gov/protein/O61213.2?report=genbank&log$=prottop&blast_rank=34&RID=) RecName: Full=Dual oxidase 1; Short=DUOX1; AltName: Full... [130](#74959793) 6e-30

[Q8HZK2.2](https://www.ncbi.nlm.nih.gov/protein/Q8HZK2.2?report=genbank&log$=prottop&blast_rank=35&RID=) RecName: Full=Dual oxidase 2; AltName: Full=NADH/NADPH t... [124](#75047044) 5e-28

[Q9MZF4.1](https://www.ncbi.nlm.nih.gov/protein/Q9MZF4.1?report=genbank&log$=prottop&blast_rank=36&RID=) RecName: Full=Dual oxidase 1; AltName: Full=NADPH thyroi... [94.7](#75050486) 9e-19

[Q9ES45.1](https://www.ncbi.nlm.nih.gov/protein/Q9ES45.1?report=genbank&log$=prottop&blast_rank=37&RID=) RecName: Full=Dual oxidase 2; AltName: Full=Large NOX 2;... [89.4](#81868356) 4e-17

[Q8HZK3.1](https://www.ncbi.nlm.nih.gov/protein/Q8HZK3.1?report=genbank&log$=prottop&blast_rank=38&RID=) RecName: Full=Dual oxidase 1; Flags: Precursor [85.5](#75047045) 7e-16

[Q9NRD8.2](https://www.ncbi.nlm.nih.gov/protein/Q9NRD8.2?report=genbank&log$=prottop&blast_rank=39&RID=) RecName: Full=Dual oxidase 2; AltName: Full=Large NOX 2;... [83.6](#296434485) 2e-15

[Q9NRD9.1](https://www.ncbi.nlm.nih.gov/protein/Q9NRD9.1?report=genbank&log$=prottop&blast_rank=40&RID=) RecName: Full=Dual oxidase 1; AltName: Full=Large NOX 1;... [81.3](#74719102) 1e-14

[Q8CIY2.1](https://www.ncbi.nlm.nih.gov/protein/Q8CIY2.1?report=genbank&log$=prottop&blast_rank=41&RID=) RecName: Full=Dual oxidase 1; Flags: Precursor [78.6](#81866480) 1e-13

>[Q7QH73.3](https://www.ncbi.nlm.nih.gov/protein/Q7QH73.3?report=genbank&log$=protalign&blast_rank=1&RID=0) RecName: Full=Chorion peroxidase; Flags: Precursor

Length=767

Score = 267 bits (682), Expect = 2e-78, Method: Compositional matrix adjust.

Identities = 189/602 (31%), Positives = 296/602 (49%), Gaps = 65/602 (11%)

Query 56 RYRQIDGRCNHP----RNYGSTGRPVKRYLRPHYQDKFGENLPRVYS-VTGQLLPSPRMV 110

RYR +DG CN+P ++G+ G P +R L P Y+D G PRV+S V+G+LL S R +

Sbjct 199 RYRSLDGSCNNPVPARSSWGAAGYPFERLLPPAYED--GVWAPRVHSSVSGRLLASARDI 256

Query 111 SWKLHPDQTAHDNN-TMLVMQMGQFIDHDITRAPELSGRNASIKCCGVPPKERLPD---- 165

S + PD D +L+MQ GQF+ HD TR+ + ++CC L

Sbjct 257 SVAVFPDVDRRDRKFNLLLMQFGQFMSHDFTRSASVRIGQEEVQCCNAEHSGALRGEQAH 316

Query 166 --CFPIDIPPGDPVFED----CMEFFRSSPAVDNDGNIIYPREQINALTSFIDGSAVYGS 219

C PI + P DP + C+ F R + A D + Y + Q+N +T FIDGSAVYGS

Sbjct 317 FACMPIAVSPADPFYSRFGIRCLNFVRLALARDGKCRLGYGK-QLNRVTHFIDGSAVYGS 375

Query 220 DLDTYTWIRSENGTGVFLNTHLVHGRERLPSHPHLGPESCVSSNTAESYCQLAGDMRVNE 279

+ +R+ G L + G E LP + + C AGD RVN+

Sbjct 376 NEALAASLRTFEGGR--LRSSFPTGEELLPF-----ARTRAACEPWAKACFRAGDDRVNQ 428

Query 280 QPGLGSIHLLFHLHHNHIVRLLVAGILKKRGQPSSPERIAKFIQESSSALKEQIFQEVRK 339

L +H LF HN + L A L + E+++QE R+

Sbjct 429 IVSLTEMHTLFLREHNRVATALAA--LNRHWD------------------DERLYQETRR 468

Query 340 MLGAIIQKLTYCDWLPMILGPYLIDKFQLGCTRRSR---YNSDLDPRVANSFLSAALRFG 396

++GA++QK+ Y ++LP I+G ++ L + + Y+ D+ P V N AA RFG

Sbjct 469 IVGAVMQKIFYNEYLPSIVGHSKARQYGLLDSHGEQTDFYSPDVKPAVFNELSGAAFRFG 528

Query 397 HTLIPNVYNFGDKR-----IHLKDTFNIPDASIR--YYDNIIQCLIKEGSEEAYDRYVSS 449

H+ + + + + +++ F P ++ ++D+ + L+ + ++ D ++

Sbjct 529 HSTVDGAFLIQHRHRRTELVPIQEVFLNPSRLLQRSFFDDFLFSLMDQ-PQQQLDDSITF 587

Query 450 AVSEHLFESTRGHKHALDLIAVNIQRGRDHGIPAYHYWRQYYRLRRIISLDEFGEAGIAM 509

++ LF + DL ++NIQRGRDH + Y+ +R + L R+ S ++FG G +

Sbjct 588 GLTRLLFAGR--NPFGSDLASLNIQRGRDHALRPYNDYRSWAGLERLTSFEQFGPVGARL 645

Query 510 KKAYRDIRDVDLFPGGLLE-PSMPGGVVGETFGHILANQFADLKFGDTYFFLH--QQAPQ 566

Y DVDL+ GGLLE P+ G + GETF I++ QFA LKFGD Y++ + + P

Sbjct 646 ASVYEFPDDVDLWVGGLLEPPTQDGALFGETFAAIISEQFARLKFGDRYYYTNGPRTNPG 705

Query 567 GFRAAQIKAILSVTMSSIICAN---SAVTQAQPDPFYMASQLNLPRPCSDYSEMDVEPWL 623

F Q++ + V+++S+ICAN + A D F S+ N P PC MD+ W

Sbjct 706 FFTGEQLRELSKVSLASVICANLDQADGFSAPRDAFRQPSEHNPPVPCQTLVGMDLSAWR 765

Query 624 IH 625

H

Sbjct 766 GH 767

>[P11247.2](https://www.ncbi.nlm.nih.gov/protein/P11247.2?report=genbank&log$=protalign&blast_rank=2&RID=0) RecName: Full=Myeloperoxidase; Short=MPO; Contains: RecName:

Full=Myeloperoxidase light chain; Contains: RecName: Full=Myeloperoxidase

heavy chain; Flags: Precursor

Length=718

Score = 263 bits (672), Expect = 2e-77, Method: Compositional matrix adjust.

Identities = 201/615 (33%), Positives = 307/615 (50%), Gaps = 93/615 (15%)

Query 56 RYRQIDGRCNHPRN--YGSTGRPVKRYLRPHYQDKFGENLPRVYSV----TGQLLPSPRM 109

+YR I G CN+ R+ G++ R R+L Y+D G ++P ++ G +P R

Sbjct 146 KYRTITGHCNNRRSPTLGASNRAFVRWLPAEYED--GVSMPFGWTPGVNRNGFKVPLARQ 203

Query 110 VS---WKLHPDQTAHDNN-TMLVMQMGQFIDHDITRAPE-------LSGRNASIKCCGVP 158

VS + DQ D ++ MQ GQF+DHDIT PE +G N C P

Sbjct 204 VSNAIVRFPNDQLTKDQERALMFMQWGQFLDHDITLTPEPATRFSFFTGLNCETSCLQQP 263

Query 159 PKERLPDCFPIDIPPGDPVFE---DCMEFFRSSPAVDNDGNIIYPREQINALTSFIDGSA 215

P CFP+ IPP DP + DC+ FFRS PA N I R QINALTSF+D S

Sbjct 264 P------CFPLKIPPNDPRIKNQKDCIPFFRSCPACTR--NNITIRNQINALTSFVDASG 315

Query 216 VYGSDLDTYTWIRS-ENGTGVF-LNTHLV-HGRERLPSHPHLGPESCVSSN-TAESYCQL 271

VYGS+ +R+ N G+ +NT +GR +P L + C+ +N +A C L

Sbjct 316 VYGSEDPLARKLRNLTNQLGLLAINTRFQDNGRALMPFD-SLHDDPCLLTNRSARIPCFL 374

Query 272 AGDMRVNEQPGLGSIHLLFHLHHNHIVRLLVAGILKKRGQPSSPERIAKFIQESSSALKE 331

AGDMR +E P L S+H LF HN + L KR P E

Sbjct 375 AGDMRSSEMPELTSMHTLFVREHNRLATQL------KRLNPRWN--------------GE 414

Query 332 QIFQEVRKMLGAIIQKLTYCDWLPMILGPYLIDKFQLGCTRRSRYNSDLDPRVANSFLSA 391

+++QE RK++GA++Q +TY D+LP++LGP + K+ + YN +DPR+AN F +

Sbjct 415 KLYQEARKIVGAMVQIITYRDYLPLVLGPAAMKKY---LPQYRSYNDSVDPRIANVF-TN 470

Query 392 ALRFGHTLI-PNVYNFGDK--------RIHLKDTFNIPDASIRYY-----DNIIQCLIKE 437

A R+GHTLI P ++ ++ R+ L F AS R D I++ L+

Sbjct 471 AFRYGHTLIQPFMFRLNNQYRPTGPNPRVPLSKVF---FASWRVVLEGGIDPILRGLMAT 527

Query 438 GSE-EAYDRYVSSAVSEHLFESTRGHKHALDLIAVNIQRGRDHGIPAYHYWRQYYRLRRI 496

++ ++ V + E LFE + LDL A+N+QR RDHG+P Y+ WR++ L +

Sbjct 528 PAKLNRQNQIVVDEIRERLFEQV--MRIGLDLPALNMQRSRDHGLPGYNAWRRFCGLPQP 585

Query 497 ISLDEFG------EAGIAMKKAYRDIRDVDLFPGGLLEPSMPGGVVGETFGHILANQFAD 550

++ E G E + Y ++D++ GG+ EP P G VG+ ++ QF

Sbjct 586 STVGELGTVLKNLELARKLMAQYGTPNNIDIWMGGVSEPLEPNGRVGQLLACLIGTQFRK 645

Query 551 LKFGDTYFFLHQQAPQGFRAAQIKAILSVTMSSIICANSAVTQAQPDPFYMASQLNLPR- 609

L+ GD +++ + P F Q +A+ S+++ IIC N+ +T + +M++ PR

Sbjct 646 LRDGDRFWW---ENPGVFSKQQRQALASISLPRIICDNTGITTVSKNNIFMSN--TYPRD 700

Query 610 --PCSDYSEMDVEPW 622

C+ ++++ W

Sbjct 701 FVSCNTLPKLNLTSW 715

>[P82600.3](https://www.ncbi.nlm.nih.gov/protein/P82600.3?report=genbank&log$=protalign&blast_rank=3&RID=0) RecName: Full=Chorion peroxidase; Flags: Precursor

Length=790

Score = 258 bits (660), Expect = 5e-75, Method: Compositional matrix adjust.

Identities = 189/601 (31%), Positives = 291/601 (48%), Gaps = 70/601 (12%)

Query 56 RYRQIDGRCNHP----RNYGSTGRPVKRYLRPHYQDKFGENLPRVYSVTGQLLPSPRMVS 111

RYR IDG CN+P ++G G P R L P Y+D G PR++SVTG LLPS R++S

Sbjct 221 RYRTIDGSCNNPLPDRTSWGMEGYPFDRVLEPAYED--GVWAPRIHSVTGNLLPSARVIS 278

Query 112 WKLHPDQTAHDNN-TMLVMQMGQFIDHDITRAPELSGRNA-SIKCCGVPPKERLP----- 164

L PD+ D +L MQMGQFI HD T + + ++ +I+CC P P

Sbjct 279 VALFPDEYRPDPRLNILFMQMGQFISHDFTLSRGFTTKHGQAIECCT--PNCTAPLFGPH 336

Query 165 ---DCFPIDIPPGDPVFED----CMEFFRSSPAVDNDGNIIYPREQINALTSFIDGSAVY 217

CFPI++PP DP + C+ R A + + Y + Q + +T F+D S VY

Sbjct 337 RHFACFPIEVPPNDPFYSRFGVRCLNLVRIRLAQGPECQLGYAK-QADLVTHFLDASTVY 395

Query 218 GSDLDTYTWIRS-ENGTGVFLNTHLVHGRERLPSHPHLGPESCVSSNTAESYCQLAGDMR 276

GS D +R+ + G L +G E LP + +CV C GD+R

Sbjct 396 GSTNDVAAELRAFQQGR---LKDSFPNGIELLPFARNR--TACVPWARV---CYEGGDIR 447

Query 277 VNEQPGLGSIHLLFHLHHNHIVRLLVAGILKKRGQPSSPERIAKFIQESSSALKEQIFQE 336

N+ GL +H LF HN L G+ K E+++QE

Sbjct 448 TNQLLGLTMVHTLFMREHNR----LAVGLSKINPHWDD----------------ERLYQE 487

Query 337 VRKMLGAIIQKLTYCDWLPMILGPYLIDKFQLGC---TRRSRYNSDLDPRVANSFLSAAL 393

R++L A Q + Y ++LP++LG + + L T + Y+ +L P +AA

Sbjct 488 ARRILIAEYQNVVYNEFLPILLGHERVQQLGLADPFDTYTNYYDPNLRPMTLAEVGAAAH 547

Query 394 RFGHTLIPNVYNFGDKR-----IHLKDTFNIPDASIRYYD-NIIQCLIKEGSEEAYDRYV 447

R+GH+L+ + F + + +KD FN P ++ +++ + E DR++

Sbjct 548 RYGHSLVEGFFRFLTRESPPEDVFIKDIFNDPSKTLEPNSFDVMMFSFNQQPMEQMDRFL 607

Query 448 SSAVSEHLFESTRGHKHALDLIAVNIQRGRDHGIPAYHYWRQYYRLRRIISLDEFGEAGI 507

+ ++ LF+ + DL ++NIQRGRD + Y+ +R++ L RI ++ GE G

Sbjct 608 TYGLTRFLFKERK--PFGSDLASLNIQRGRDFAVRPYNDYREWAGLGRITDFNQLGEVGA 665

Query 508 AMKKAYRDIRDVDLFPGGLLEPSMPGGVVGETFGHILANQFADLKFGDTYFFLH--QQAP 565

+ + Y DVDL+PGG+LEP G VVG TF +L+ + K D Y+F + + P

Sbjct 666 LLAQVYESPDDVDLWPGGVLEPPAEGAVVGSTFVALLSAGYTRYKRADRYYFTNGPEVNP 725

Query 566 QGFRAAQIKAILSVTMSSIICANS----AVTQAQPDPFYMASQLNLPRPCSDYSEMDVEP 621

F Q+ I T++ IICAN+ QAQ + +S N+P PC+ Y +++

Sbjct 726 GAFTLQQLGEIRRTTLAGIICANADHKEDFYQAQ-EALRQSSADNVPVPCTRYDTVNLGL 784

Query 622 W 622

W

Sbjct 785 W 785

>[A4IGL7.1](https://www.ncbi.nlm.nih.gov/protein/A4IGL7.1?report=genbank&log$=protalign&blast_rank=4&RID=0) RecName: Full=Peroxidasin; Flags: Precursor

Length=1457

Score = 251 bits (640), Expect = 3e-70, Method: Compositional matrix adjust.

Identities = 195/622 (31%), Positives = 300/622 (48%), Gaps = 82/622 (13%)

Query 44 LEHCSRLTYDQLRYRQIDGRCN---HPRNYGSTGRPVKRYLRPHYQDKFGENLPRVYS-- 98

+ +CS + + Q +YR DG CN HP +G++ +R L+ Y++ F NLPR S

Sbjct 719 INNCSNMCFHQ-KYRTHDGTCNNLQHPM-WGASLTAFERLLKSVYENGF--NLPRGISGR 774

Query 99 -VTGQLLPSPRMVSWKLHPDQTAHDNN--TMLVMQMGQFIDHDI-------TRAPELSGR 148

G LP PR+VS L T + T ++MQ GQF+DHD+ ++A G+

Sbjct 775 IYNGFPLPLPRLVSTTLIGTHTITPDEQFTHMLMQWGQFLDHDLDSTVVALSQARFSDGQ 834

Query 149 NASIKCCGVPPKERLPDCFPIDIPPGDPVFED---CMEFFRSSPAVDNDG-----NIIYP 200

+ S+ C P CFPI +PP DP + CM RSSP + N +YP

Sbjct 835 DCSVVCTNDAP------CFPIMVPPNDPRVRNNARCMSMVRSSPVCGSGMTSLLMNSVYP 888

Query 201 REQINALTSFIDGSAVYGSDLDTYTWIRSENGTGVFLNTHLVH--GRERLPSHPHLGPES 258

REQ+N LTS+ID S VYGS IR L +V G+ LP E

Sbjct 889 REQMNQLTSYIDASNVYGSSDHESNEIRDSASHRGLLKQGIVQRSGKPLLPFATGPPTEC 948

Query 259 CVSSNTAESYCQLAGDMRVNEQPGLGSIHLLFHLHHNHIVRLLVAGILKKRGQPSSPERI 318

N + C LAGD R NEQ GL S+H L+ HN I

Sbjct 949 MRDENESPIPCFLAGDHRANEQLGLTSMHTLWFREHNRIA-------------------- 988

Query 319 AKFIQESSSALKEQIFQEVRKMLGAIIQKLTYCDWLPMILGPYLIDKFQLGCTRRSRYNS 378

+ ++ + + I+ E RK++GA +Q +TY WLP I G + LG Y+

Sbjct 989 TELLRLNPHWDGDTIYHETRKIVGAQMQHITYSHWLPKIFGD--VGMKMLG--EYKSYDP 1044

Query 379 DLDPRVANSFLSAALRFGHTLI-PNVYNFGDK-------RIHLKDTFNIPDASIRY--YD 428

+++ + N F +AA RFGHTLI P +Y +K + L F P + D

Sbjct 1045 NVNAGILNEFATAAFRFGHTLINPILYRLDEKFEPIPQGHVPLHRAFFSPFRIVNEGGID 1104

Query 429 NIIQCLIKEGSE-EAYDRYVSSAVSEHLFESTRGHKHALDLIAVNIQRGRDHGIPAYHYW 487

+++ LI ++ + +++ ++E LF + H ALDL A+N+QRGRDHGIP YH +

Sbjct 1105 PLLRGLIGVAAKMRVTSQLLNTELTEKLF--SMAHAVALDLAALNVQRGRDHGIPPYHDF 1162

Query 488 RQYYRLRRIISLDEFG------EAGIAMKKAYRDIRDVDLFPGGLLEPSMPGGVVGETFG 541

R + L + + D+ + +K+ Y ++DLFP ++E +PG +G T

Sbjct 1163 RVFCNLSTVQTFDDLRNEIKNPDVREKLKRLYGSPLNIDLFPALMVEDLIPGSRLGPTLM 1222

Query 542 HILANQFADLKFGDTYFFLHQQAPQGFRAAQIKAILSVTMSSIICANS-AVTQAQPDPFY 600

+L QF +++ GD +++ + P F AAQ+ I +++ ++C N +T+ Q D F

Sbjct 1223 CLLTTQFRNIRDGDRFWY---ENPGVFTAAQLTQIKQTSLARVLCDNGDNITKVQHDLFR 1279

Query 601 MASQLNLPRPCSDYSEMDVEPW 622

+A + C + ++MD+ W

Sbjct 1280 VAEFPHGYVSCKNIAKMDLRVW 1301

>[P49290.2](https://www.ncbi.nlm.nih.gov/protein/P49290.2?report=genbank&log$=protalign&blast_rank=5&RID=0) RecName: Full=Eosinophil peroxidase; Short=EPO; Contains: RecName:

Full=Eosinophil peroxidase light chain; Contains: RecName:

Full=Eosinophil peroxidase heavy chain; Flags: Precursor

Length=716

Score = 243 bits (620), Expect = 6e-70, Method: Compositional matrix adjust.

Identities = 191/624 (31%), Positives = 305/624 (49%), Gaps = 96/624 (15%)

Query 45 EHCSRLTYDQLRYRQIDGRCNHPRN--YGSTGRPVKRYLRPHYQD-------------KF 89

E CS +YR I GRCN+ ++ G++ + + R+L Y+D +

Sbjct 140 ERCSN------KYRTITGRCNNKKHPWLGASNQALARWLPAEYEDHRSLPFGWTPGKRRN 193

Query 90 GENLPRVYSVTGQLLPSPRMVSWKLHPDQTAHDNNTMLVMQMGQFIDHDITRAPELSGRN 149

G LP V V+ Q++ R S KL D+ ++ MQ GQFIDHD+ +PE R

Sbjct 194 GFLLPLVRDVSNQIV---RFPSKKLTSDR----GRALMFMQWGQFIDHDLDFSPESPARV 246

Query 150 A------SIKCCGVPPKERLPDCFPIDIPPGDPVFE---DCMEFFRSSPAVDNDGNIIYP 200

A K C +LP CFPI IP DP + DC+ FFRS+PA + N +

Sbjct 247 AFSMGVDCEKTCA-----QLPPCFPIKIPRNDPRIKNQRDCIPFFRSAPACPQNRNKV-- 299

Query 201 REQINALTSFIDGSAVYGSDLDTYTWIRSENGTGVFLNTHLV---HGRERLPSHPHLGPE 257

R QINALTSF+D S VYGS++ +R+ L T+ +GR LP +L +

Sbjct 300 RNQINALTSFVDASMVYGSEVTLALRLRNRTNFLGLLATNQRFQDNGRALLP-FDNLHED 358

Query 258 SCVSSN-TAESYCQLAGDMRVNEQPGLGSIHLLFHLHHNHIVRLLVAGILKKRGQPSSPE 316

C+ +N +A C LAGD R +E P L ++H LF HN +

Sbjct 359 PCLLTNRSARIPCFLAGDTRSSETPKLTALHTLFVREHNRLA------------------ 400

Query 317 RIAKFIQESSSALKEQIFQEVRKMLGAIIQKLTYCDWLPMILGPYLIDKFQLGCTRRSRY 376

A+ + + ++++ E RK++GA++Q +TY D+LP++LG I + LG R Y

Sbjct 401 --AELRRLNPHWSGDKLYNEARKIVGAMVQIITYRDFLPLVLGRARIRR-TLGPYRG--Y 455

Query 377 NSDLDPRVANSFLSAALRFGHTLI-PNVYNFGDKRIHLKDTFNIPDASIRY--------- 426

S++DPRVAN F + A RFGHT++ P ++ + ++P +S+ +

Sbjct 456 CSNVDPRVANVF-TLAFRFGHTMLQPFMFRLDSQYRASAPNSHVPLSSVFFASWRIIHEG 514

Query 427 -YDNIIQCLIKEGSE-EAYDRYVSSAVSEHLFESTRGHKHALDLIAVNIQRGRDHGIPAY 484

D I++ L+ ++ D + + + LF+ R + LDL A+N+QR RDHG+P Y

Sbjct 515 GIDPILRGLMATPAKLNRQDSMLVDELRDKLFQQVR--RIGLDLAALNMQRSRDHGLPGY 572

Query 485 HYWRQYYRLRRIISLDEFGEA----GIAMK--KAYRDIRDVDLFPGGLLEPSMPGGVVGE 538

+ WR++ L + +L + +A K + Y+ ++D++ G + EP +PG VG

Sbjct 573 NAWRRFCGLSQPRNLAQLSRVLKNQDLARKFLRLYKTPDNIDIWVGAIAEPLLPGARVGP 632

Query 539 TFGHILANQFADLKFGDTYFFLHQQAPQGFRAAQIKAILSVTMSSIICANSAVTQAQPDP 598

+ NQF + GD +++ Q F Q KA+ +++S I+C N+ +T D

Sbjct 633 LLACLFENQFRRARDGDRFWW---QKWGVFTKRQRKALRRISLSRIVCDNTGITTVSRDI 689

Query 599 FYMASQLNLPRPCSDYSEMDVEPW 622

F CS ++++ W

Sbjct 690 FRANIYPQGFVSCSRIPKLNLSAW 713

>[P11678.2](https://www.ncbi.nlm.nih.gov/protein/P11678.2?report=genbank&log$=protalign&blast_rank=6&RID=0) RecName: Full=Eosinophil peroxidase; Short=EPO; Contains: RecName:

Full=Eosinophil peroxidase light chain; Contains: RecName:

Full=Eosinophil peroxidase heavy chain; Flags: Precursor

Length=715

Score = 243 bits (620), Expect = 6e-70, Method: Compositional matrix adjust.

Identities = 195/612 (32%), Positives = 306/612 (50%), Gaps = 88/612 (14%)

Query 56 RYRQIDGRCNHPRN--YGSTGRPVKRYLRPHYQDKFGENLPRVYSVT----GQLLPSPRM 109

+YR I GRCN+ R G++ + + R+L Y+D G +LP ++ + G LLP R

Sbjct 144 KYRTITGRCNNKRRPLLGASNQALARWLPAEYED--GLSLPFGWTPSRRRNGFLLPLVRA 201

Query 110 VSWKL--HPDQ--TAHDNNTMLVMQMGQFIDHDITRAPELSGR---NASIKC---CGVPP 159

VS ++ P++ T+ ++ MQ GQFIDHD+ +PE R A + C C

Sbjct 202 VSNQIVRFPNERLTSDRGRALMFMQWGQFIDHDLDFSPESPARVAFTAGVDCERTCA--- 258

Query 160 KERLPDCFPIDIPPGDPVFE---DCMEFFRSSPAVDNDGNIIYPREQINALTSFIDGSAV 216

+LP CFPI IPP DP + DC+ FFRS+P+ + N + R QINALTSF+D S V

Sbjct 259 --QLPPCFPIKIPPNDPRIKNQRDCIPFFRSAPSCPQNKNRV--RNQINALTSFVDASMV 314

Query 217 YGSDLDTYTWIRSE-NGTGVF-LNTHLV-HGRERLPSHPHLGPESCVSSN-TAESYCQLA 272

YGS++ +R+ N G+ +N +GR LP +L + C+ +N +A C LA

Sbjct 315 YGSEVSLSLRLRNRTNYLGLLAINQRFQDNGRALLP-FDNLHDDPCLLTNRSARIPCFLA 373

Query 273 GDMRVNEQPGLGSIHLLFHLHHNHIVRLLVAGILKKRGQPSSPERIAKFIQESSSALKEQ 332

GD R E P L ++H LF HN + L +R P ++

Sbjct 374 GDTRSTETPKLAAMHTLFMREHNRLATEL------RRLNPRWN--------------GDK 413

Query 333 IFQEVRKMLGAIIQKLTYCDWLPMILGPYLIDKFQLGCTRRSRYNSDLDPRVANSFLSAA 392

++ E RK++GA++Q +TY D+LP++LG + LG R Y S++DPRVAN F + A

Sbjct 414 LYNEARKIMGAMVQIITYRDFLPLVLGKARARR-TLGHYR--GYCSNVDPRVANVF-TLA 469

Query 393 LRFGHTLI-PNVYNFGDKRIHLKDTFNIPDASIRY----------YDNIIQCLIKEGSE- 440

RFGHT++ P ++ + ++P +S + D I++ L+ ++

Sbjct 470 FRFGHTMLQPFMFRLDSQYRASAPNSHVPLSSAFFASWRIVYEGGIDPILRGLMATPAKL 529

Query 441 EAYDRYVSSAVSEHLFESTRGHKHALDLIAVNIQRGRDHGIPAYHYWRQYYRLRRIISLD 500

D + + + LF R + LDL A+N+QR RDHG+P Y+ WR++ L + +L

Sbjct 530 NRQDAMLVDELRDRLFRQVR--RIGLDLAALNMQRSRDHGLPGYNAWRRFCGLSQPRNLA 587

Query 501 EFGEA----GIAMK--KAYRDIRDVDLFPGGLLEPSMPGGVVGETFGHILANQFADLKFG 554

+ +A K Y ++D++ G + EP +PG VG + NQF + G

Sbjct 588 QLSRVLKNQDLARKFLNLYGTPDNIDIWIGAIAEPLLPGARVGPLLACLFENQFRRARDG 647

Query 555 DTYFFLHQQAPQGFRAAQIKAILSVTMSSIICANSAVTQAQPDPF----YMASQLNLPRP 610

D +++ Q F Q KA+ +++S IIC N+ +T D F Y +N

Sbjct 648 DRFWW---QKRGVFTKRQRKALSRISLSRIICDNTGITTVSRDIFRANIYPRGFVN---- 700

Query 611 CSDYSEMDVEPW 622

CS +++ W

Sbjct 701 CSRIPRLNLSAW 712

>[P05164.1](https://www.ncbi.nlm.nih.gov/protein/P05164.1?report=genbank&log$=protalign&blast_rank=7&RID=0) RecName: Full=Myeloperoxidase; Short=MPO; Contains: RecName:

Full=Myeloperoxidase; Contains: RecName: Full=89 kDa myeloperoxidase;

Contains: RecName: Full=84 kDa myeloperoxidase; Contains:

RecName: Full=Myeloperoxidase light chain; Contains:

RecName: Full=Myeloperoxidase heavy chain; Flags: Precursor

Length=745

Score = 243 bits (620), Expect = 1e-69, Method: Compositional matrix adjust.

Identities = 188/620 (30%), Positives = 305/620 (49%), Gaps = 97/620 (16%)

Query 53 DQLRYRQIDGRCNHPRN--YGSTGRPVKRYLRPHYQDKF-------------GENLPRVY 97

+Q +YR I G CN+ R+ G++ R R+L Y+D F G +

Sbjct 169 EQDKYRTITGMCNNRRSPTLGASNRAFVRWLPAEYEDGFSLPYGWTPGVKRNGFPVALAR 228

Query 98 SVTGQLLPSPRMVSWKLHPDQTAHDNNTMLVMQMGQFIDHDITRAPE-------LSGRNA 150

+V+ +++ R + +L PDQ +++ MQ GQ +DHD+ PE ++G N

Sbjct 229 AVSNEIV---RFPTDQLTPDQ----ERSLMFMQWGQLLDHDLDFTPEPAARASFVTGVNC 281

Query 151 SIKCCGVPPKERLPDCFPIDIPPGDPVFE---DCMEFFRSSPAVDNDGNIIYPREQINAL 207

C PP CFP+ IPP DP + DC+ FFRS PA G+ I R QINAL

Sbjct 282 ETSCVQQPP------CFPLKIPPNDPRIKNQADCIPFFRSCPACP--GSNITIRNQINAL 333

Query 208 TSFIDGSAVYGSDLDTYTWIRS-ENGTGVFLNTHLV--HGRERLPSHPHLGPESCVSSN- 263

TSF+D S VYGS+ +R+ N G+ +GR LP +L + C+ +N

Sbjct 334 TSFVDASMVYGSEEPLARNLRNMSNQLGLLAVNQRFQDNGRALLPFD-NLHDDPCLLTNR 392

Query 264 TAESYCQLAGDMRVNEQPGLGSIHLLFHLHHNHIVRLLVAGILKKRGQPSSPERIAKFIQ 323

+A C LAGD R +E P L S+H L HN + L + + G

Sbjct 393 SARIPCFLAGDTRSSEMPELTSMHTLLLREHNRLATELKSLNPRWDG------------- 439

Query 324 ESSSALKEQIFQEVRKMLGAIIQKLTYCDWLPMILGPYLIDKFQLGCTRRSRYNSDLDPR 383

E+++QE RK++GA++Q +TY D+LP++LGP + K+ T RS YN +DPR

Sbjct 440 -------ERLYQEARKIVGAMVQIITYRDYLPLVLGPTAMRKYL--PTYRS-YNDSVDPR 489

Query 384 VANSFLSAALRFGHTLI-PNVYNFGDKRIHLKDTFNIPDASIRY----------YDNIIQ 432

+AN F + A R+GHTLI P ++ ++ ++ +P + + + D I++

Sbjct 490 IANVF-TNAFRYGHTLIQPFMFRLDNRYQPMEPNPRVPLSRVFFASWRVVLEGGIDPILR 548

Query 433 CLIKEGSE-EAYDRYVSSAVSEHLFESTRGHKHALDLIAVNIQRGRDHGIPAYHYWRQYY 491

L+ ++ ++ + E LFE + LDL A+N+QR RDHG+P Y+ WR++

Sbjct 549 GLMATPAKLNRQNQIAVDEIRERLFEQV--MRIGLDLPALNMQRSRDHGLPGYNAWRRFC 606

Query 492 RLRRIISLDEFGEAGIAMKKA------YRDIRDVDLFPGGLLEPSMPGGVVGETFGHILA 545

L + ++ + G +K A Y ++D++ GG+ EP G VG I+

Sbjct 607 GLPQPETVGQLGTVLRNLKLARKLMEQYGTPNNIDIWMGGVSEPLKRKGRVGPLLACIIG 666

Query 546 NQFADLKFGDTYFFLHQQAPQGFRAAQIKAILSVTMSSIICANSAVTQAQPDPFYMASQL 605

QF L+ GD +++ ++ F Q +A+ +++ IIC N+ +T + +M++

Sbjct 667 TQFRKLRDGDRFWWENEGV---FSMQQRQALAQISLPRIICDNTGITTVSKNNIFMSN-- 721

Query 606 NLPRP---CSDYSEMDVEPW 622

+ PR CS +++ W

Sbjct 722 SYPRDFVNCSTLPALNLASW 741

>[Q9VEG6.3](https://www.ncbi.nlm.nih.gov/protein/Q9VEG6.3?report=genbank&log$=protalign&blast_rank=8&RID=0) RecName: Full=Chorion peroxidase; AltName: Full=Peroxinectin-related

protein; Short=Dpxt; Flags: Precursor

Length=809

Score = 241 bits (615), Expect = 1e-68, Method: Compositional matrix adjust.

Identities = 176/574 (31%), Positives = 283/574 (49%), Gaps = 63/574 (11%)

Query 57 YRQIDGRCNHPRN----YGSTGRPVKRYLRPHYQDKFGENLPRVYSVTGQLLPSPRMVSW 112

YR +DG CN+P +G+ G+P++R L P Y+D G PR +S G L R +S

Sbjct 237 YRSMDGTCNNPEPQRSLWGAAGQPMERMLPPAYED--GIWTPRAHSSDGTPLLGARKISR 294

Query 113 KLHPD-QTAHDNNTMLVMQMGQFIDHDITRAPELSGRNAS-IKCC------GVPPKERLP 164

L D H ++VMQ GQ + HDI++ + + S ++CC + P++

Sbjct 295 TLLSDVDRPHPKYNLMVMQFGQVLAHDISQTSSIRLEDGSLVQCCSPEGKVALSPQQSHF 354

Query 165 DCFPIDIPPGDPVFED----CMEFFRSSPAVDNDGNIIYPREQINALTSFIDGSAVYGSD 220

C PI + P D F C+ F R S D + Y + Q+ +T F+D S VYGS

Sbjct 355 ACMPIHVEPDDEFFSAFGVRCLNFVRLSLVPSPDCQLSYGK-QLTKVTHFVDASPVYGSS 413

Query 221 LDTYTWIRSENGTGVFLNTHLVHGRERLPSHPHLGPESCVSSNTAESYCQLAGDMRVNEQ 280

+ +R+ G + + GR+ LP ++C S +S C +GD R N+

Sbjct 414 DEASRSLRAFRGGRLRMMNDF--GRDLLPLTND--KKACPSEEAGKS-CFHSGDGRTNQI 468

Query 281 PGLGSIHLLFHLHHNHIVRLLVAGILKKRGQPSSPERIAKFIQESSSALKEQIFQEVRKM 340

L ++ +L HN VAG L + + SA E +FQE R++

Sbjct 469 ISLITLQILLAREHNR-----VAGALH---------------ELNPSASDETLFQEARRI 508

Query 341 LGAIIQKLTYCDWLPMILGPYLIDKFQLGCTRRS---RYNSDLDPRVANSFLSAALRFGH 397

+ A +Q +TY ++LP+I+GP + +F+L + YN +++P + N F AA R GH

Sbjct 509 VIAEMQHITYNEFLPIIIGPQQMKRFRLVPLHQGYSHDYNVNVNPAITNEFSGAAYRMGH 568

Query 398 TLIPNVYNFGDKRIHLKDTFNIPDASI--------RYYDNIIQCLIKEGSEEAYDRYVSS 449

+ + + + + + NIPD +YD++++ L + ++ D +S

Sbjct 569 SSVDGKFQIRQEHGRIDEVVNIPDVMFNPSRMRKREFYDDMLRTLYSQPMQQV-DSSISQ 627

Query 450 AVSEHLFESTRG-HKHALDLIAVNIQRGRDHGIPAYHYWRQYYRLRRIISLDEFG-EAGI 507

+S LF RG + LDL A+NIQRGRD G+ +Y+ + + ++ S ++F E

Sbjct 628 GLSRFLF---RGDNPFGLDLAAINIQRGRDQGLRSYNDYLELMGAPKLHSFEQFPIEIAQ 684

Query 508 AMKKAYRDIRDVDLFPGGLLEPSMPGGVVGETFGHILANQFADLKFGDTYFFLHQQA--P 565

+ + YR D+DL+ GGLLE ++ GGVVG TF I+A+QFA K GD Y++ + P

Sbjct 685 KLSRVYRTPDDIDLWVGGLLEKAVEGGVVGVTFAEIIADQFARFKQGDRYYYEYDNGINP 744

Query 566 QGFRAAQIKAILSVTMSSIICANSAVTQAQPDPF 599

F Q++ I VT++ ++C NS Q P

Sbjct 745 GAFNPLQLQEIRKVTLARLLCDNSDRLTLQAVPL 778

>[Q3UQ28.2](https://www.ncbi.nlm.nih.gov/protein/Q3UQ28.2?report=genbank&log$=protalign&blast_rank=9&RID=0) RecName: Full=Peroxidasin homolog; Flags: Precursor

Length=1475

Score = 244 bits (622), Expect = 6e-68, Method: Compositional matrix adjust.

Identities = 199/623 (32%), Positives = 297/623 (48%), Gaps = 83/623 (13%)

Query 44 LEHCSRLTYDQLRYRQIDGRCN---HPRNYGSTGRPVKRYLRPHYQDKF----GENLPRV 96

+ +CS + + Q +YR DG CN HP +G++ +R L+ Y++ F G N R

Sbjct 726 VNNCSDMCFHQ-KYRTHDGTCNNLQHPM-WGASLTAFERLLKAVYENGFNTPRGINSQRQ 783

Query 97 YSVTGQLLPSPRMVSWKLHPDQ--TAHDNNTMLVMQMGQFIDHDI-------TRAPELSG 147

Y+ G +LP PR+VS L + T + T ++MQ GQF+DHD+ ++A G

Sbjct 784 YN--GHVLPMPRLVSTTLIGTEVITPDEQFTHMLMQWGQFLDHDLDSTVVALSQARFSDG 841

Query 148 RNASIKCCGVPPKERLPDCFPIDIPPGDPVFED---CMEFFRSSPAVDNDG-----NIIY 199

++ S C PP CF + IPP DP CM F RSSP + N +Y

Sbjct 842 QHCSSVCSNDPP------CFSVMIPPNDPRVRSGARCMFFVRSSPVCGSGMTSLLMNSVY 895

Query 200 PREQINALTSFIDGSAVYGSDLDTYTWIRSENGTGVFLNTHLVH--GRERLPSHPHLGPE 257

PREQIN LTS+ID S VYGS IR L +V G+ LP E

Sbjct 896 PREQINQLTSYIDASNVYGSTDHEARSIRDLASHRGLLRQGIVQRSGKPLLPFATGPPTE 955

Query 258 SCVSSNTAESYCQLAGDMRVNEQPGLGSIHLLFHLHHNHIVRLLVAGILKKRGQPSSPER 317

N + C LAGD R NEQ GL S+H L+ HN I

Sbjct 956 CMRDENESPIPCFLAGDHRANEQLGLTSMHTLWFREHNRIA------------------- 996

Query 318 IAKFIQESSSALKEQIFQEVRKMLGAIIQKLTYCDWLPMILGPYLIDKFQLGCTRRSRYN 377

A+ ++ + + ++ E RK++GA IQ +TY WLP ILG + LG R Y+

Sbjct 997 -AELLKLNPHWDGDTVYHETRKIVGAEIQHITYRHWLPKILGE--VGMKMLGEYRG--YD 1051

Query 378 SDLDPRVANSFLSAALRFGHTLI-PNVYNFGDK-------RIHLKDTFNIPDASIRY--Y 427

++ + N+F +AA RFGHTLI P +Y + + L F P +

Sbjct 1052 PSVNAGIFNAFATAAFRFGHTLINPLLYRLDENFEPIPQGHVPLHKAFFSPFRIVNEGGI 1111

Query 428 DNIIQCLIK-EGSEEAYDRYVSSAVSEHLFESTRGHKHALDLIAVNIQRGRDHGIPAYHY 486

D +++ L G + +++ ++E LF + H ALDL A+NIQRGRDHGIP YH

Sbjct 1112 DPLLRGLFGVAGKMRIPSQLLNTELTERLF--SMAHTVALDLAAINIQRGRDHGIPPYHD 1169

Query 487 WRQYYRLRRIISLDEFGEAGIA------MKKAYRDIRDVDLFPGGLLEPSMPGGVVGETF 540

+R Y L + ++ + +++ Y ++DLFP ++E +PG +G T

Sbjct 1170 YRVYCNLSAAYTFEDLKNEIKSPVIREKLQRLYGSTLNIDLFPALMVEDLVPGSRLGPTL 1229

Query 541 GHILANQFADLKFGDTYFFLHQQAPQGFRAAQIKAILSVTMSSIICANS-AVTQAQPDPF 599

+L+ QF L+ GD ++ + P F AQ+ + +++ I+C NS +T+ Q D F

Sbjct 1230 MCLLSTQFRRLRDGDRLWY---ENPGVFSPAQLTQLKQTSLARILCDNSDNITRVQQDVF 1286

Query 600 YMASQLNLPRPCSDYSEMDVEPW 622

+A + C D +D+ W

Sbjct 1287 RVAEFPHGYSSCEDIPRVDLRVW 1309

>[Q92626.2](https://www.ncbi.nlm.nih.gov/protein/Q92626.2?report=genbank&log$=protalign&blast_rank=10&RID=0) RecName: Full=Peroxidasin homolog; AltName: Full=Melanoma-associated

antigen MG50; AltName: Full=Vascular peroxidase 1; AltName:

Full=p53-responsive gene 2 protein; Flags: Precursor

Length=1479

Score = 237 bits (604), Expect = 2e-65, Method: Compositional matrix adjust.

Identities = 199/623 (32%), Positives = 295/623 (47%), Gaps = 83/623 (13%)

Query 44 LEHCSRLTYDQLRYRQIDGRCN---HPRNYGSTGRPVKRYLRPHYQDKF----GENLPRV 96

+ +CS + + Q +YR DG CN HP +G++ +R L+ Y++ F G N R+

Sbjct 729 VNNCSDMCFHQ-KYRTHDGTCNNLQHPM-WGASLTAFERLLKSVYENGFNTPRGINPHRL 786

Query 97 YSVTGQLLPSPRMVSWKLHPDQTAHDNN--TMLVMQMGQFIDHDI-------TRAPELSG 147

Y+ G LP PR+VS L +T + T ++MQ GQF+DHD+ ++A G

Sbjct 787 YN--GHALPMPRLVSTTLIGTETVTPDEQFTHMLMQWGQFLDHDLDSTVVALSQARFSDG 844

Query 148 RNASIKCCGVPPKERLPDCFPIDIPPGDPVFED---CMEFFRSSPAVDNDG-----NIIY 199

++ S C PP CF + IPP D CM F RSSP + N +Y

Sbjct 845 QHCSNVCSNDPP------CFSVMIPPNDSRARSGARCMFFVRSSPVCGSGMTSLLMNSVY 898

Query 200 PREQINALTSFIDGSAVYGSDLDTYTWIRSENGTGVFLNTHLVH--GRERLPSHPHLGPE 257

PREQIN LTS+ID S VYGS IR L +V G+ LP E

Sbjct 899 PREQINQLTSYIDASNVYGSTEHEARSIRDLASHRGLLRQGIVQRSGKPLLPFATGPPTE 958

Query 258 SCVSSNTAESYCQLAGDMRVNEQPGLGSIHLLFHLHHNHIVRLLVAGILKKRGQPSSPER 317

N + C LAGD R NEQ GL S+H L+ HN I

Sbjct 959 CMRDENESPIPCFLAGDHRANEQLGLTSMHTLWFREHNRIA------------------- 999

Query 318 IAKFIQESSSALKEQIFQEVRKMLGAIIQKLTYCDWLPMILGPYLIDKFQLGCTRRSRYN 377

+ ++ + + I+ E RK++GA IQ +TY WLP ILG + LG Y+

Sbjct 1000 -TELLKLNPHWDGDTIYYETRKIVGAEIQHITYQHWLPKILGE--VGMRTLG--EYHGYD 1054

Query 378 SDLDPRVANSFLSAALRFGHTLI-PNVY----NF---GDKRIHLKDTFNIPDASIRY--Y 427

++ + N+F +AA RFGHTL+ P +Y NF + L F P +

Sbjct 1055 PGINAGIFNAFATAAFRFGHTLVNPLLYRLDENFQPIAQDHLPLHKAFFSPFRIVNEGGI 1114

Query 428 DNIIQCLIK-EGSEEAYDRYVSSAVSEHLFESTRGHKHALDLIAVNIQRGRDHGIPAYHY 486

D +++ L G + +++ ++E LF + H ALDL A+NIQRGRDHGIP YH

Sbjct 1115 DPLLRGLFGVAGKMRVPSQLLNTELTERLF--SMAHTVALDLAAINIQRGRDHGIPPYHD 1172

Query 487 WRQYYRLRRIISLDEFG------EAGIAMKKAYRDIRDVDLFPGGLLEPSMPGGVVGETF 540

+R Y L + ++ E +K+ Y ++DLFP ++E +PG +G T

Sbjct 1173 YRVYCNLSAAHTFEDLKNEIKNPEIREKLKRLYGSTLNIDLFPALVVEDLVPGSRLGPTL 1232

Query 541 GHILANQFADLKFGDTYFFLHQQAPQGFRAAQIKAILSVTMSSIICANS-AVTQAQPDPF 599

+L+ QF L+ GD ++ + P F AQ+ I +++ I+C N+ +T+ Q D F

Sbjct 1233 MCLLSTQFKRLRDGDRLWY---ENPGVFSPAQLTQIKQTSLARILCDNADNITRVQSDVF 1289

Query 600 YMASQLNLPRPCSDYSEMDVEPW 622

+A + C + +D+ W

Sbjct 1290 RVAEFPHGYGSCDEIPRVDLRVW 1312

>[Q8R481.1](https://www.ncbi.nlm.nih.gov/protein/Q8R481.1?report=genbank&log$=protalign&blast_rank=11&RID=0) RecName: Full=Lactoperoxidase; Short=LPO; AltName: Full=Lacrimal

gland peroxidase; Flags: Precursor

Length=710

Score = 226 bits (577), Expect = 7e-64, Method: Compositional matrix adjust.

Identities = 187/607 (31%), Positives = 292/607 (48%), Gaps = 78/607 (13%)

Query 57 YRQIDGRCNHPRN--YGSTGRPVKRYLRPHYQDKFGENLPRVYSV----TGQLLPSPRMV 110

YR I G CN+ +N GS R + R+L Y+D G +LP ++ G LP PR V

Sbjct 136 YRTITGDCNNRKNPELGSANRALARWLPAEYED--GLSLPFGWTPGKTRNGFPLPQPRDV 193

Query 111 SWK----LHPDQTAHDNNTMLVMQMGQFIDHDITRAPE--LSGRNASIKCCGVPPKERLP 164

S + L+ ++ N ++L MQ GQ +DHD+ APE + N S C +

Sbjct 194 SNQVLDYLNEEEILDQNRSLLFMQWGQIVDHDLDFAPETEMGSDNYSKAQCDELCIQG-D 252

Query 165 DCFPIDIPPGDPVFE---DCMEFFRSSPAVDNDGNIIYPREQINALTSFIDGSAVYGSDL 221

+CFPI P GDP + C+ FFR+ REQINALTSF+D S VYGS+

Sbjct 253 NCFPIMFPKGDPKLKTQGKCLPFFRAGFVCPTSPYQSLAREQINALTSFMDASMVYGSEP 312

Query 222 DTYTWIRS-ENGTGVFLNTHLV--HGRERLPSHPHLGPESC-VSSNTAESYCQLAGDMRV 277

+R+ + G+ V HGR LP ++ P C V + TA C LAGD R

Sbjct 313 SLANRLRNLSSPLGLMAVNEEVSDHGRPLLP-FVNVKPSPCEVINRTAGVPCFLAGDSRA 371

Query 278 NEQPGLGSIHLLFHLHHNHIVRLLVAGILKKRGQPSSPERIAKFIQESSSALKEQIFQEV 337

+EQ L + H LF HN + R L R P E+++QE

Sbjct 372 SEQILLATSHTLFLREHNRLAREL------SRLNPQWD--------------GEKLYQEA 411

Query 338 RKMLGAIIQKLTYCDWLPMILGPYLIDKFQLGCTRRSRYNSDLDPRVANSFLSAALRFGH 397

R+++GA+IQ +T+ D+LP++LG D+ Q Y +DPR++N F + A RFGH

Sbjct 412 RRIMGALIQIITFRDYLPILLG----DELQKWIPPYQGYKETVDPRISNVF-TFAFRFGH 466

Query 398 TLIPNVYNFGDKR-----------IHLKDTFN----IPDASIRYYDNIIQCLIKEGSEEA 442

+P+ + D+ +H K FN + D I D +++ L+ + ++ A

Sbjct 467 LEVPSTVSRLDENYQPWGSEPELPLH-KLFFNTWRVVKDGGI---DPLVRGLLAKKAKLA 522

Query 443 Y-DRYVSSAVSEHLFESTRGHKHALDLIAVNIQRGRDHGIPAYHYWRQYYRLRRIISLDE 501

+ D+ ++ + LF+ H DL A+NIQR RDHG P Y+ WR + L + +L+E

Sbjct 523 HQDKMMTGELRNMLFQPNH-TVHGFDLAAINIQRCRDHGQPGYNSWRAFCGLSQPKTLEE 581

Query 502 FGEA--GIAMKKAYRDI----RDVDLFPGGLLEPSMPGGVVGETFGHILANQFADLKFGD 555

+ K D+ ++D++ G + EP + G VG +L QF ++ GD

Sbjct 582 LSAVLRNEVLAKKLMDLYGTPDNIDIWLGAIAEPLVRRGRVGPLLTCLLGQQFQRIRDGD 641

Query 556 TYFFLHQQAPQGFRAAQIKAILSVTMSSIICANSAVTQAQPDPFYMASQLNLPRPCSDYS 615

+++ + P F Q ++ ++ S ++C N+ + + +PF S + CS

Sbjct 642 RFWW---ENPGVFTEKQRDSLQKMSFSRLVCDNTGINKVPLNPFQPNSYPHSFVDCSAIE 698

Query 616 EMDVEPW 622

++D+ PW

Sbjct 699 KLDLTPW 705

>[P35419.1](https://www.ncbi.nlm.nih.gov/protein/P35419.1?report=genbank&log$=protalign&blast_rank=12&RID=0) RecName: Full=Thyroid peroxidase; Short=TPO; Flags: Precursor

Length=914

Score = 225 bits (574), Expect = 2e-62, Method: Compositional matrix adjust.

Identities = 186/620 (30%), Positives = 287/620 (46%), Gaps = 88/620 (14%)

Query 56 RYRQIDGRCN---HPRNYGSTGRPVKRYLRPHYQDKFGENLPRVYSVT----GQLLPSPR 108

+YR I G CN HPR +G++ + R+L P Y+D F + P+ ++ G LP R

Sbjct 144 KYRPITGACNNRDHPR-WGASNTALARWLPPVYEDGFSQ--PKGWNPNFLYHGFPLPPVR 200

Query 109 MVSWKL----HPDQTAHDNNTMLVMQMGQFIDHDITRAPELS-------GRNASIKCCGV 157

V+ L + T D + + GQ+IDHDI P+ + G + + C

Sbjct 201 EVTRHLIQVSNEAVTEDDQYSDFLPVWGQYIDHDIALTPQSTSTAAFWGGVDCQLTC--- 257

Query 158 PPKERLPDCFPIDIPPGDPVFEDCMEFFRSSPAVDND------GNI--IYPREQINALTS 209

E CFPI +P C+ F+RSS A GN+ PR+Q+N LTS

Sbjct 258 ---ENQNPCFPIQLPSNSSGTTACLPFYRSSAACGTGDQGALFGNLSAANPRQQMNGLTS 314

Query 210 FIDGSAVYGSDLDTYTWIRSENGTGVFLNTHLVH---GRERLP-SHPHLGPESCVSSNTA 265

F+D S VYGS +R+ + + L + +H GR LP + PE + T

Sbjct 315 FLDASTVYGSSPGVEKQLRNWSSSAGLLRVNTLHLDAGRAYLPFATAACAPEP-GTPRTN 373

Query 266 ESYCQLAGDMRVNEQPGLGSIHLLFHLHHNHIVRLLVAGILKKRGQPSSPERIAKFIQES 325

+ C LAGD R +E P L ++H L+ HN + K I +

Sbjct 374 RTPCFLAGDGRASEVPALAAVHTLWLREHNRLASAF------------------KAINKH 415

Query 326 SSALKEQIFQEVRKMLGAIIQKLTYCDWLPMILGPYLIDKFQLGCTRRSRYNSDLDPRVA 385

SA +QE RK++GA+ Q +T D++P ILGP D F+ YN ++P V+

Sbjct 416 WSA--NTAYQEARKVVGALHQIITMRDYIPKILGP---DAFRQYVGPYEGYNPTVNPTVS 470

Query 386 NSFLSAALRFGH-TLIPNVYNFGDK--------RIHLKDTFNIPDASIRY--YDNIIQCL 434

N F +AA RFGH T+ P V R+ L+D F P I+ D I++ L

Sbjct 471 NIFSTAAFRFGHATVHPLVRRLNTDFQEHTELPRLQLRDVFFRPWRLIQEGGLDPIVRGL 530

Query 435 IKEGSE-EAYDRYVSSAVSEHLFESTRGHKHALDLIAVNIQRGRDHGIPAYHYWRQYYRL 493

+ ++ + + ++ ++E LF + LDL ++N+QRGRDHG+P Y+ WR++ L

Sbjct 531 LARAAKLQVQGQLMNEELTERLF--VLSNVGTLDLASLNLQRGRDHGLPDYNEWREFCGL 588

Query 494 RRIISLDEFGEAGIAMKKAYRDIRD-------VDLFPGGLLEPSMPGGVVGETFGHILAN 546

R+ + E +A IA + I D +D++ GGL E +PG G F I+

Sbjct 589 SRLETPAELNKA-IANRSMVNKIMDLYKHADNIDVWLGGLAEKFLPGARTGPLFACIIGK 647

Query 547 QFADLKFGDTYFFLHQQAPQGFRAAQIKAILSVTMSSIICANSAVTQAQPDPFYMASQLN 606

Q L+ GD +++ + F AQ + + ++ +IC N+ +T+ D F +

Sbjct 648 QMKALRDGDRFWWENTNV---FTDAQRQELEKHSLPRVICDNTGLTRVPVDAFRIGKFPQ 704

Query 607 LPRPCSDYSEMDVEPWLIHF 626

C D MD+E W F

Sbjct 705 DFESCEDIPSMDLELWRETF 724

>[Q9VZZ4.1](https://www.ncbi.nlm.nih.gov/protein/Q9VZZ4.1?report=genbank&log$=protalign&blast_rank=13&RID=0) RecName: Full=Peroxidasin; Flags: Precursor

Length=1527

Score = 226 bits (577), Expect = 6e-62, Method: Compositional matrix adjust.

Identities = 188/626 (30%), Positives = 295/626 (47%), Gaps = 88/626 (14%)

Query 43 DLEHCSRLTYDQLRYRQIDGRCN---HPRNYGSTGRPVKRYLRPHYQDKF----GENLPR 95

++ +C+ + + RYR IDG CN HP +G++ +R P Y++ F G

Sbjct 764 EMPNCTDMCFHS-RYRSIDGTCNNLQHP-TWGASLTAFRRLAPPIYENGFSMPVGWTKGM 821

Query 96 VYSVTGQLLPSPRMVSWKLHPDQ--TAHDNNTMLVMQMGQFIDHDITRA-PELSGRNA-S 151

+YS G PS R+VS L + T T +VMQ GQF+DHD+ A P +S +

Sbjct 822 LYS--GHAKPSARLVSTSLVATKEITPDARITHMVMQWGQFLDHDLDHAIPSVSSESWDG 879

Query 152 IKCCGVPPKERLPDCFPIDIPPGDPVFED--CMEFFRSSPAVDNDG------NIIYPREQ 203

I C E P C+PI++PP DP + C++ RSS A+ G + + REQ

Sbjct 880 IDC--KKSCEMAPPCYPIEVPPNDPRVRNRRCIDVVRSS-AICGSGMTSLFFDSVQHREQ 936

Query 204 INALTSFIDGSAVYGSDLDTYTWIRSENGTGVFL--NTHLVHGRERLP-SHPHLGPESCV 260

IN LTS+ID S VYG +R+ L H ++ LP + P G +

Sbjct 937 INQLTSYIDASQVYGYSTAFAQELRNLTSQEGLLRVGVHFPRQKDMLPFAAPQDGMDCRR 996

Query 261 SSNTAESYCQLAGDMRVNEQPGLGSIHLLFHLHHNHIVRLLVAGILKKRGQPSSPERIAK 320

+ + C ++GD+RVNEQ GL ++H ++ HN I +K

Sbjct 997 NLDENTMSCFVSGDIRVNEQVGLLAMHTIWMREHNRIA--------------------SK 1036

Query 321 FIQESSSALKEQIFQEVRKMLGAIIQKLTYCDWLPMILGPYLIDKFQLGCTRRSRYNSDL 380

Q +S + ++QE RK++GA +Q +T+ WLP+I+G ++ YN L

Sbjct 1037 LKQINSHWDGDTLYQEARKIVGAQMQHITFKQWLPLIIGESGMEMM----GEYQGYNPQL 1092

Query 381 DPRVANSFLSAALRFGHTLIPNVYNFGDKRIHLKDTFN-IPDASIRY-----------YD 428

+P +AN F +AALRFGHT+I + + L +TF IP + Y+

Sbjct 1093 NPSIANEFATAALRFGHTIINPILH------RLNETFQPIPQGHLLLHKAFFAPWRLAYE 1146

Query 429 NIIQCLIK-----EGSEEAYDRYVSSAVSEHLFESTRGHKHALDLIAVNIQRGRDHGIPA 483

+ L++ + D+ +++ ++E LF++ H ALDL A+NIQRGRDHG+P

Sbjct 1147 GGVDPLMRGFLAVPAKLKTPDQNLNTELTEKLFQT--AHAVALDLAAINIQRGRDHGMPG 1204

Query 484 YHYWRQYYRLRRIISLDEF-GEAGIA-----MKKAYRDIRDVDLFPGGLLEPSMPGGVVG 537

Y+ +R+ L ++ GE A MK+ Y +VD++ GG+LE + GG VG

Sbjct 1205 YNVYRKLCNLTVAQDFEDLAGEISSAEIRQKMKELYGHPDNVDVWLGGILEDQVEGGKVG 1264

Query 538 ETFGHILANQFADLKFGDTYFFLHQQAPQGFRAAQIKAILSVTMSSIIC-ANSAVTQAQP 596

F +L QF L+ GD ++ + P F Q+ I ++C Q

Sbjct 1265 PLFQCLLVEQFRRLRDGDRLYY---ENPGVFSPEQLTQIKQANFGRVLCDVGDNFDQVTE 1321

Query 597 DPFYMASQLNLPRPCSDYSEMDVEPW 622

+ F +A + C D +++ W

Sbjct 1322 NVFILAKHQGGYKKCEDIIGINLYLW 1347

>[P14650.1](https://www.ncbi.nlm.nih.gov/protein/P14650.1?report=genbank&log$=protalign&blast_rank=14&RID=0) RecName: Full=Thyroid peroxidase; Short=TPO; Flags: Precursor

Length=914

Score = 220 bits (561), Expect = 1e-60, Method: Compositional matrix adjust.

Identities = 185/620 (30%), Positives = 290/620 (47%), Gaps = 88/620 (14%)

Query 56 RYRQIDGRCN---HPRNYGSTGRPVKRYLRPHYQDKFGENLPRVYSVT----GQLLPSPR 108

+YR I G CN HPR +G++ + R+L P Y+D F + PR ++ G LP R

Sbjct 144 KYRPITGVCNNRDHPR-WGASNTALARWLPPVYEDGFSQ--PRGWNPNFLYHGFPLPPVR 200

Query 109 MVSWKL----HPDQTAHDNNTMLVMQMGQFIDHDITRAPELS-------GRNASIKCCGV 157

V+ L + T D + + GQ+IDHDI P+ + G + + C

Sbjct 201 EVTRHLIQVSNEAVTEDDQYSDFLPVWGQYIDHDIALTPQSTSTAAFWGGVDCQLTC--- 257

Query 158 PPKERLPDCFPIDIPPGDPVFEDCMEFFRSSPAVDND------GNI--IYPREQINALTS 209

E CFPI +P C+ F+RSS A GN+ PR+Q+N LTS

Sbjct 258 ---ENQNPCFPIQLPSNSSRTTACLPFYRSSAACGTGDQGALFGNLSAANPRQQMNGLTS 314

Query 210 FIDGSAVYGSDLDTYTWIRSENGTGVFLNTHLVH---GRERLP-SHPHLGPESCVSSNTA 265

F+D S VYGS +R+ + + L + +H GR LP + PE + +

Sbjct 315 FLDASTVYGSSPGVEKQLRNWSSSAGLLRVNTLHLDSGRAYLPFASAACAPEPG-APHAN 373

Query 266 ESYCQLAGDMRVNEQPGLGSIHLLFHLHHNHIVRLLVAGILKKRGQPSSPERIAKFIQES 325

+ C LAGD R +E P L ++H L+ HN + K I

Sbjct 374 RTPCFLAGDGRASEVPALAAVHTLWLREHNRLATAF------------------KAINTH 415

Query 326 SSALKEQIFQEVRKMLGAIIQKLTYCDWLPMILGPYLIDKFQLGCTRRSRYNSDLDPRVA 385

SA +QE RK++GA+ Q +T D++P ILGP D F+ YN ++P V+

Sbjct 416 WSA--NTAYQEARKVVGALHQIITMRDYIPKILGP---DAFRQYVGPYEGYNPTVNPTVS 470

Query 386 NSFLSAALRFGH-TLIPNV----YNFGDK----RIHLKDTFNIPDASIRY--YDNIIQCL 434

N F +AA RFGH T+ P V +F D R+ L D F P I+ D I++ L

Sbjct 471 NVFSTAAFRFGHATVHPLVRRLNTDFQDHTELPRLQLHDVFFRPWRLIQEGGLDPIVRGL 530

Query 435 IKEGSE-EAYDRYVSSAVSEHLFESTRGHKHALDLIAVNIQRGRDHGIPAYHYWRQYYRL 493

+ ++ + ++ ++ ++E LF + LDL ++N+QRGRDHG+P Y+ WR++ L

Sbjct 531 LARPAKLQVQEQLMNEELTERLF--VLSNVGTLDLASLNLQRGRDHGLPGYNEWREFCGL 588

Query 494 RRIISLDEFGEAGIA-------MKKAYRDIRDVDLFPGGLLEPSMPGGVVGETFGHILAN 546

R+ + E +A IA + + Y+ ++D++ GGL E +PG G F I+

Sbjct 589 SRLDTGAELNKA-IANRSMVNKIMELYKHADNIDVWLGGLAEKFLPGARTGPLFACIIGK 647

Query 547 QFADLKFGDTYFFLHQQAPQGFRAAQIKAILSVTMSSIICANSAVTQAQPDPFYMASQLN 606

Q L+ GD +++ + F AQ + + ++ +IC N+ +T+ D F +

Sbjct 648 QMKALRDGDRFWWENSHV---FTDAQRQELEKHSLPRVICDNTGLTRVPVDAFRIGKFPQ 704

Query 607 LPRPCSDYSEMDVEPWLIHF 626

C + MD+ W F

Sbjct 705 DFESCEEIPSMDLRLWRETF 724

>[Q23490.1](https://www.ncbi.nlm.nih.gov/protein/Q23490.1?report=genbank&log$=protalign&blast_rank=15&RID=0) RecName: Full=Peroxidase mlt-7; AltName: Full=Molting defective

protein 7; Short=MoLT-7; Contains: RecName: Full=Peroxidase

mlt-7 light chain; Contains: RecName: Full=Peroxidase mlt-7

heavy chain; Flags: Precursor

Length=724

Score = 216 bits (551), Expect = 3e-60, Method: Compositional matrix adjust.

Identities = 181/621 (29%), Positives = 280/621 (45%), Gaps = 98/621 (16%)

Query 33 TGSLDFVSEADLEHCSRLTYDQLRYRQIDGRCNHPRN--YGSTGRPVKRYLRPHYQDKFG 90

TG + +S+ ++ L Y L YR +DG CN+ G+ R R+ Y D G

Sbjct 168 TGCVPQLSDVGVDCRKSLCY-HLMYRTLDGTCNNLEKPMQGAAFRRFNRHFPAQYDDGKG 226

Query 91 ENLPRVYSVTGQLLPSPRMVSWKL--HPDQTAHDNNTMLVMQMGQFIDHDITRAPELSGR 148

E + S Q PS R + + HD ++MQ GQF+ HD+++

Sbjct 227 EPI----SSLNQSRPSAREANRVMLSSAQSVVHDKFNNMMMQWGQFMSHDMSKTTLQPSA 282

Query 149 NASIKCCGVPPKERLPDCFPIDIPPGDPVF----EDCMEFFRSSPAVDNDGNIIYPREQI 204

N C VP K C PI I DP + C++ RS+P + PREQ+

Sbjct 283 NCKT-CDPVPSK-----CMPIPIGEKDPNLGFKSKQCLKVSRSAPICR-----VEPREQL 331

Query 205 NALTSFIDGSAVYGSDLDTYTWIRSENGTGVFLNTHLVHGRERLP--SHPHLGPESCVSS 262

N T++IDGS +YGS L R +G FL + + LP + C +S

Sbjct 332 NENTAYIDGSMIYGSSLKDLHKFR--DGRTGFLRVTRFNNQNVLPFDQSKCANKDKCTAS 389

Query 263 NTAESYCQLAGDMRVNEQPGLGSIHLLFHLHHNHIVRLLVAGILKKRGQPSSPERIAKFI 322

TA GD+R N GL S+H++F HN I + K

Sbjct 390 FTA-------GDIRANLFIGLSSLHIMFAREHNRIAQ--------------------KLT 422

Query 323 QESSSALKEQIFQEVRKMLGAIIQKLTYCDWLPMILGPYLIDKFQLGCTRRSRYNSDLDP 382

+ + + +++FQE RK++GA IQ + Y ++LP +LG F Y++++D

Sbjct 423 ELNPTWSGDRVFQEARKIVGAQIQNVLYKEYLPKLLGV----SFDKVIGPYKGYDTNVDA 478

Query 383 RVANSFLSAALRFGHTLIPNVYNFGDKRIHLKDTFNIPDASIR-----------YYDNII 431

+AN F ++A RFGH +I Y KR+ L NI ++ +

Sbjct 479 TIANEFTTSAFRFGHGMIEEFY----KRVDLSGN-NITHGGFFFGDGVFKSGKILFEGGV 533

Query 432 QCLIKEGSEEAYDR--YVSSAVSEHLFESTRGHKHALDLIAVNIQRGRDHGIPAYHYWRQ 489

+I+ A R ++ A++E +F ST DL ++NIQRGRDHGIP+Y+ RQ

Sbjct 534 DPIIRGFMTTAVKRPHRMTPAITEKMFGST-------DLGSLNIQRGRDHGIPSYNKMRQ 586

Query 490 YYRLRRIISLDEFGE--------AGIAMKKAYRDIRDVDLFPGGLLEPSMPGGVVGETFG 541

+ L+ + D+F + AG+A + Y DVD + G +LE + GG+VG T

Sbjct 587 FCGLKSANTFDDFADMILDRNLRAGLA--RNYNTTNDVDFYVGSMLEDPVIGGLVGTTLS 644

Query 542 HILANQFADLKFGDTYFFLHQQAPQGFRAAQIKAILSVTMSSIICANSAVTQAQPDPFYM 601

+ QF + GD ++F + P F +Q++ I ++S IIC N+ + ++

Sbjct 645 CAIGEQFKRARDGDRFYF---ENPGIFTRSQMEEIKKSSLSRIICDNADNFELVSQDAFL 701

Query 602 ASQLNLPRPCSDYSEMDVEPW 622

NL PCS +MD+ W

Sbjct 702 LPGSNLT-PCSKIPKMDLSKW 721

>[P22079.2](https://www.ncbi.nlm.nih.gov/protein/P22079.2?report=genbank&log$=protalign&blast_rank=16&RID=0) RecName: Full=Lactoperoxidase; Short=LPO; AltName: Full=Salivary

peroxidase; Short=SPO; Flags: Precursor

Length=712

Score = 216 bits (549), Expect = 5e-60, Method: Compositional matrix adjust.

Identities = 188/607 (31%), Positives = 284/607 (47%), Gaps = 78/607 (13%)

Query 57 YRQIDGRCNHPRN--YGSTGRPVKRYLRPHYQDKFGENLPRVYSV----TGQLLPSPRMV 110

YR I G CN+ R G+ R + R+L Y+D G +LP ++ G LP R V

Sbjct 138 YRTITGDCNNRRKPALGAANRALARWLPAEYED--GLSLPFGWTPGKTRNGFPLPLAREV 195

Query 111 SWK----LHPDQTAHDNNTMLVMQMGQFIDHDITRAP--ELSGRNASIKCCGVPPKERLP 164

S K L+ + N ++L MQ GQ +DHD+ AP EL S C +

Sbjct 196 SNKIVGYLNEEGVLDQNRSLLFMQWGQIVDHDLDFAPDTELGSSEYSKAQCDEYCIQG-D 254

Query 165 DCFPIDIPPGDP---VFEDCMEFFRSSPAVDNDGNIIYPREQINALTSFIDGSAVYGSDL 221

+CFPI PP DP CM FFR+ REQINALTSF+D S VY S+

Sbjct 255 NCFPIMFPPNDPKAGTQGKCMPFFRAGFVCPTPPYKSLAREQINALTSFLDASFVYSSEP 314

Query 222 DTYTWIRS-ENGTGVFLNTHLV--HGRERLPSHPHLGPESCVSSNT-AESYCQLAGDMRV 277

+ +R+ + G+ V HG LP + P C NT A C LAGD R

Sbjct 315 SLASRLRNLSSPLGLMAVNQEVSDHGLPYLP-YDSKKPSPCEFINTTARVPCFLAGDSRA 373

Query 278 NEQPGLGSIHLLFHLHHNHIVRLLVAGILKKRGQPSSPERIAKFIQESSSALKEQIFQEV 337

+E L + H LF HN + R L KR P E+++QE

Sbjct 374 SEHILLATSHTLFLREHNRLAREL------KRLNPQWD--------------GEKLYQEA 413

Query 338 RKMLGAIIQKLTYCDWLPMILGPYLIDKFQLGCTRRSRYNSDLDPRVANSFLSAALRFGH 397

RK+LGA +Q +T+ D+LP++LG D Q Y+ +DPR++N F + A RFGH

Sbjct 414 RKILGAFVQIITFRDYLPILLG----DHMQKWIPPYQGYSESVDPRISNVF-TFAFRFGH 468

Query 398 TLIP--------NVYNFG-DKRIHLKDTF-----NIPDASIRYYDNIIQCLIKEGSE-EA 442

+P N +G + + L F + D I D +++ L+ + S+

Sbjct 469 LEVPSSMFRLDENYQPWGPEPELPLHTLFFNTWRMVKDGGI---DPLVRGLLAKKSKLMK 525

Query 443 YDRYVSSAVSEHLFESTRGHK-HALDLIAVNIQRGRDHGIPAYHYWRQYYRLRRIISLDE 501

++ ++ + LF+ T H+ H DL A+N QR RDHG P Y+ WR + L + +L+E

Sbjct 526 QNKMMTGELRNKLFQPT--HRIHGFDLAAINTQRCRDHGQPGYNSWRAFCDLSQPQTLEE 583

Query 502 FG---EAGIAMKK---AYRDIRDVDLFPGGLLEPSMPGGVVGETFGHILANQFADLKFGD 555

++ + KK Y ++D++ G + EP + G VG +L QF ++ GD

Sbjct 584 LNTVLKSKMLAKKLLGLYGTPDNIDIWIGAIAEPLVERGRVGPLLACLLGKQFQQIRDGD 643

Query 556 TYFFLHQQAPQGFRAAQIKAILSVTMSSIICANSAVTQAQPDPFYMASQLNLPRPCSDYS 615

+++ + P F Q ++ ++ S ++C N+ +T+ DPF+ S CS

Sbjct 644 RFWW---ENPGVFTNEQKDSLQKMSFSRLVCDNTRITKVPRDPFWANSYPYDFVDCSAID 700

Query 616 EMDVEPW 622

++D+ PW

Sbjct 701 KLDLSPW 707

>[H2A0M7.1](https://www.ncbi.nlm.nih.gov/protein/H2A0M7.1?report=genbank&log$=protalign&blast_rank=17&RID=0) RecName: Full=Peroxidase-like protein; Flags: Precursor

Length=793

Score = 217 bits (552), Expect = 8e-60, Method: Compositional matrix adjust.

Identities = 181/583 (31%), Positives = 275/583 (47%), Gaps = 88/583 (15%)

Query 54 QLRYRQIDGRCNH---------PRNYGSTGRPVKRYLRPHYQDKFGENLPRVYSVT-GQL 103

Q RYR+ DG+CN+ G+ R+L P Y D G + PR+ SV G L

Sbjct 164 QQRYRETDGQCNNLVFPSFPSGAFKLGAAFTAQGRFLFPAYDD--GVSSPRIRSVIPGFL 221

Query 104 LPSPRMVSWKLHPDQTAHDNN--TMLVMQMGQFIDHDITRAPELSGRNA--SIKCCGVPP 159

LP+ R+VS +H TA D++ T + GQFIDHDI PE + + CC P

Sbjct 222 LPNARLVSRNVH-SGTAFDSDRHTPFLTHFGQFIDHDIVSTPETEPKFTMPNSHCCLEP- 279

Query 160 KERLPDCFPIDIPPGDPVFE-DCMEFFRSSPAVD---NDGNIIYPREQINALTSFIDGSA 215

L +CF I+ P DP+ + C+ F R+ A N G PR Q N +SF+DG+

Sbjct 280 --NLEECFNINFEP-DPLLQGSCIRFNRADTAPSYFCNPG----PRLQQNQRSSFVDGTM 332

Query 216 VYGSDLDTYTWIRSENGTGVFLNTHLVHGRERLPSHPHLGPES--CVSSNTAESYCQLAG 273

VYG D++ +R E GTG ++ G ++L P P + C ++ C AG

Sbjct 333 VYGWDVEQENRLR-EPGTGRLIS----EGDDQLKLEPVADPLNPPCFP---VDNRCFEAG 384

Query 274 DMRVNEQPGLGSIHLLFHLHHNHIVRLLVAGILKKRGQPSSPERIAKFIQESSSALKEQI 333

D R E L +H++F HN IV+ L + P +PE +

Sbjct 385 DHRSLETVPLTVMHIMFLRRHNLIVQEL-----QNLPLPWTPE---------------LL 424

Query 334 FQEVRKMLGAIIQKLTYCDWLPMILGPYLIDKFQL--GCTRRSRYNSDLDPRVANSFLSA 391

FQE ++++ A +Q +TY ++LP +LGP + F+L Y+ +DPR + F A

Sbjct 425 FQEAKRIVVAELQHITYNEFLPRVLGPQFMTIFRLWPAPLFSDTYSPLVDPRTTSGFSVA 484

Query 392 ALRFGHTLIPNVYN------FGDKRIHLKDTFNIPDASIRYY----DNIIQCLIKEGSEE 441

A RFGH+L+ NV++ + L+D F+ + + +K +

Sbjct 485 AYRFGHSLVRNVHDQIGPGGLPVNNLLLQDHFDRLQTHLNVFPGGNTEGFARWMKLSQKS 544

Query 442 AYDRYVSSAVSEHLFESTR-------GHKHALDLIAVNIQRGRDHGIPAYHYWRQYYRLR 494

DR + + +LF G + DL A+NIQRGRDHG+P Y WR + R

Sbjct 545 RADRTLVDGLQNNLFPCEDPDCPMGGGVTKSFDLAALNIQRGRDHGLPPYTAWRYWCTGR 604

Query 495 R--IISLDEFG-------EAGIAMKKAYRDIRDVDLFPGGLLEPSMPGGVVGETFGHILA 545

R + + + G EA I + YR + D+DLF GG+ E PG ++G T I+

Sbjct 605 RAFVFTPNAVGLSDHSPFEANI-LSNTYRHVDDIDLFTGGMTEMRRPGALLGPTLSCIIG 663

Query 546 NQFADLKFGDTYFFLHQQAPQGFRAAQIKAILSVTMSSIICAN 588

QF++ K GD +F+ F Q++AI +++ I+C+

Sbjct 664 LQFSNYKRGDRFFYERPDPVMAFTPGQLQAIKETSLAKILCST 706

>[A0A452E9Y6.1](https://www.ncbi.nlm.nih.gov/protein/A0A452E9Y6.1?report=genbank&log$=protalign&blast_rank=18&RID=0) RecName: Full=Lactoperoxidase; Flags: Precursor

Length=712

Score = 215 bits (548), Expect = 8e-60, Method: Compositional matrix adjust.

Identities = 186/609 (31%), Positives = 280/609 (46%), Gaps = 82/609 (13%)

Query 57 YRQIDGRCNHPRN--YGSTGRPVKRYLRPHYQDKFGENLPRVYSVT--GQLLPSPRMVSW 112

YR I G CN+ R+ G+ R + R+L Y+D T G +P R VS

Sbjct 138 YRTITGDCNNRRSPALGAANRALARWLPAEYEDGLAVPFGWTQRKTRNGFRVPLAREVSN 197

Query 113 K----LHPDQTAHDNNTMLVMQMGQFIDHDITRAPEL---SGRNASIKCCGVPPKERLPD 165

K L + N ++L MQ GQ +DHD+ APE S ++ ++C + +

Sbjct 198 KIVGYLDEEGVLDQNRSLLFMQWGQIVDHDLDFAPETELGSSEHSKVQCEEYCIQGD--N 255

Query 166 CFPIDIPPGDPVFE---DCMEFFRSSPAVDNDGNIIYPREQINALTSFIDGSAVYGSDLD 222

CFPI P DP + CM FFR+ R+QINA+TSF+D S VYGS+

Sbjct 256 CFPIMFPKNDPKLKTQGKCMPFFRAGFVCPTPPYQSLARDQINAVTSFLDASLVYGSEPS 315

Query 223 TYTWIR---SENGTGVFLNTHLVHGRERLPSHPHLGPESCVSSNT-AESYCQLAGDMRVN 278

+ +R S G HG P ++ P C NT A C AGD R +

Sbjct 316 LASRLRNLSSPLGLMAVNQEAWDHGLA-YPPFNNVKPSPCEFINTTAHVPCFQAGDSRAS 374

Query 279 EQPGLGSIHLLFHLHHNHIVRLLVAGILKKRGQPSSPERIAKFIQESSSALKEQIFQEVR 338

EQ L ++H L HN + R L KR P E ++QE R

Sbjct 375 EQILLATVHTLLLREHNRLAREL------KRLNPHWD--------------GEMLYQEAR 414

Query 339 KMLGAIIQKLTYCDWLPMILGPYLIDKFQLGCTRRSRYNSDLDPRVANSFLSAALRFGHT 398

K+LGA IQ +T+ D+LP++LG + Q YN+ +DPR++N F + A RFGH

Sbjct 415 KILGAFIQIITFRDYLPIVLG----SEMQKWIPPYQGYNNSVDPRISNVF-TFAFRFGHM 469

Query 399 LIPNVYNFGDKRIH-------------LKDTFNI-PDASIRYYDNIIQCLIKEGSE-EAY 443

+P+ + D+ +T+ I D I D +++ L+ + S+

Sbjct 470 EVPSTVSRLDENYQPWGPEAELPLHTLFFNTWRIIKDGGI---DPLVRGLLAKKSKLMNQ 526

Query 444 DRYVSSAVSEHLFESTRGHK-HALDLIAVNIQRGRDHGIPAYHYWRQYYRL---RRIISL 499

++ V+S + LF+ T HK H DL A+N+QR RDHG+P Y+ WR + L + + L

Sbjct 527 NKMVTSELRNKLFQPT--HKIHGFDLAAINLQRCRDHGMPGYNSWRGFCGLSQPKTLKGL 584

Query 500 DEFGEAGIAMKK---AYRDIRDVDLFPGGLLEPSMPGGVVGETFGHILANQFADLKFGDT 556

+ I KK Y+ ++D++ GG EP + G VG +L QF ++ GD

Sbjct 585 QAVLKNKILAKKLLDLYKTPDNIDIWIGGNAEPMVERGRVGPLLACLLGRQFQQIRDGDR 644

Query 557 YFFLHQQAPQGFRAAQIKAILSVTMSSIICANSAVTQAQPDPFYMASQLNLPR---PCSD 613

+++ + P F Q ++ V+ S +IC N+ VT+ P + N P CS

Sbjct 645 FWW---ENPGVFTEKQRDSLQKVSFSRLICDNTHVTKV---PLHAFQANNYPHDFVDCSA 698

Query 614 YSEMDVEPW 622

++D+ PW

Sbjct 699 VDKLDLSPW 707

>[A8WQH2.1](https://www.ncbi.nlm.nih.gov/protein/A8WQH2.1?report=genbank&log$=protalign&blast_rank=19&RID=0) RecName: Full=Peroxidasin homolog; Flags: Precursor

Length=1288

Score = 220 bits (560), Expect = 9e-60, Method: Compositional matrix adjust.

Identities = 188/615 (31%), Positives = 302/615 (49%), Gaps = 83/615 (13%)

Query 56 RYRQIDGRCNH---PRNYGSTGRPVKRYLRPHYQDKF----GENLPRVYSVTGQLLPSPR 108

+YR DG+CN+ P N G + P++R L+P Y++ F G ++Y+ G +P+ R

Sbjct 632 KYRSFDGQCNNKNKPMN-GVSLMPLRRLLKPVYENGFNTPVGWEKGKLYN--GYPMPNVR 688

Query 109 MVSWKLHPDQTA--HDNNTMLVMQMGQFIDHDITRAPELSGRN--ASIKCCGVPPKERLP 164

VS +L +T H + +VMQ GQF+DHD+T R+ A+ C + L

Sbjct 689 EVSRQLVATETITPHRKLSSMVMQWGQFVDHDLTHTVTALSRHSYATGAFCNRTC-DNLD 747

Query 165 DCFPIDIPPGDP-VFED-----CMEFFRSSPAVDNDG------NIIYPREQINALTSFID 212

CF I + P DP V + C+EF RS+ AV G N + REQ+NALTSF+D

Sbjct 748 PCFNIPLSPSDPRVISESAKYPCIEFERSA-AVCGSGETSLVFNRVTYREQMNALTSFLD 806

Query 213 GSAVYGSDLDTYTWIRS--ENGTGVFLNTHLVHGRERLPSHPHLGPESCVSSNTAES--Y 268

S VYGS+ +R N + + G+E LP + C + + E+

Sbjct 807 ASNVYGSNEVQAQELRDTYNNNGQLRYDITSAAGKEYLPFEKDSNMD-CRRNFSEENPIR 865

Query 269 CQLAGDMRVNEQPGLGSIHLLFHLHHNHIVRLLVAGILKKRGQPSSPERIAKFIQESSSA 328

C LAGD+R NEQ L + H +F HN RIAK +++ +

Sbjct 866 CFLAGDLRANEQLALAATHTIFVREHN---------------------RIAKKLKKMNGN 904

Query 329 LK-EQIFQEVRKMLGAIIQKLTYCDWLPMILG-PYLIDKFQLGCTRRSRYNSDLDPRVAN 386

E I+ E RK++GA++Q +T+ WLP++ G +DKF + Y+ +D V N

Sbjct 905 WDGEVIYHETRKIIGAMMQHITFKHWLPVVFGGQEQMDKF---VGKYQGYDPAIDSSVTN 961

Query 387 SFLSAALRFGHTLI-PNVYNFGDK-------RIHLKDTFNIPDASIRY--YDNIIQCLIK 436

+F +AA RFGHT+I P ++ G+ I L F P+ + D +++ L

Sbjct 962 AFATAAFRFGHTIINPTLFRLGNDFMSIKQGHIALHKAFFTPELVLTEGGIDPLLRGLFA 1021

Query 437 EGSEEAY-DRYVSSAVSEHLFESTRGHKHALDLIAVNIQRGRDHGIPAYHYWRQYYRL-- 493

+ + ++ + E LF +GH+ +LDL +NIQR RDHG+P+Y +RQ+ L

Sbjct 1022 SPLKHPMPTQLLNMELIEKLF--MKGHEVSLDLAVMNIQRSRDHGLPSYTEYRQFCNLPV 1079

Query 494 -RRIISLDEFGEAGIAMKK---AYRDIRDVDLFPGGLLEPSMPGGVVGETFGHILANQFA 549

R + + + + ++K Y +++DL+ GG++E + G+ G TF I+ QF

Sbjct 1080 PARWEDMKGYIKDDMIIQKLRGLYGVPQNIDLWVGGIVEEKLENGLFGPTFACIIGEQFR 1139

Query 550 DLKFGDTYFFLHQQAPQGFRAAQIKAILSVTMSSIICANS-AVTQAQPDPF-YMASQLNL 607

++ GD +++ + F Q+K I VT++ ++C N + + Q D F Y +

Sbjct 1140 KMRDGDRFWY---EKDGVFTPEQMKEIKKVTLARLLCDNGDEIDRIQKDVFMYPGKEKEN 1196

Query 608 PRPCSDYSEMDVEPW 622

C D MD++ W

Sbjct 1197 YGRCEDTEMMDLKAW 1211

>[P09933.1](https://www.ncbi.nlm.nih.gov/protein/P09933.1?report=genbank&log$=protalign&blast_rank=20&RID=0) RecName: Full=Thyroid peroxidase; Short=TPO; Flags: Precursor

Length=926

Score = 218 bits (555), Expect = 9e-60, Method: Compositional matrix adjust.

Identities = 183/613 (30%), Positives = 287/613 (47%), Gaps = 78/613 (13%)

Query 56 RYRQIDGRCN---HPRNYGSTGRPVKRYLRPHYQDKFGENLPRVYS----VTGQLLPSPR 108

+YR I G CN HPR +G++ + R+L P Y+D E PR ++ G LP R

Sbjct 150 KYRLITGACNNRDHPR-WGASNTALARWLPPAYEDGVTE--PRGWNPHFLYNGLPLPPVR 206

Query 109 MVSWK-LHPDQTAHDNNTM---LVMQMGQFIDHDITRAPELSGRNASIKC--CGVPPKER 162

V+ + +H A + L+M GQ+IDHDI P+ + + A C + + R

Sbjct 207 EVTRQVIHVSNEAVTEDGQYSDLLMAWGQYIDHDIAFTPQSTSKAAFAGGADCQLTCENR 266

Query 163 LPDCFPIDIP--PGDPVFEDCMEFFRSSPAVDND------GNIIY--PREQINALTSFID 212

P CFPI +P C+ F+RSS A + GN+ + PR+Q+N LTSF+D

Sbjct 267 SP-CFPIQLPTNASGAAGATCLPFYRSSAACGSGRQGALVGNLSWAAPRQQMNGLTSFLD 325

Query 213 GSAVYGSDLDTYTWIRSENGTGVFLNTHLVH---GRERLPSHPHLGPESCV---SSNTAE 266

S VYGS +R+ L + H GR LP P P +C + A

Sbjct 326 ASTVYGSSPAQEQRLRNWTSAEGLLRVNTRHRDAGRAFLPFAPPPAPPACAPEPGTPAAR 385

Query 267 SYCQLAGDMRVNEQPGLGSIHLLFHLHHNHIVRLLVAGILKKRGQPSSPERIAKFIQESS 326

+ C LAGD R +E PGL ++H L+ HN + A F ++

Sbjct 386 APCFLAGDSRASEVPGLTALHTLWLREHNRLA--------------------AAFKALNA 425

Query 327 SALKEQIFQEVRKMLGAIIQKLTYCDWLPMILGPYLIDKFQLGCTRRSRYNSDLDPRVAN 386

+ ++QE RK++GA+ Q +T D++P ILG + F Y+ +DP V+N

Sbjct 426 HWSADTVYQEARKVVGALHQIVTLRDYVPKILG---AEAFGQHVGPYQGYDPAVDPTVSN 482

Query 387 SFLSAALRFGHTLIPNVYNFGDKRIH--------LKDTFNIPDASIRY--YDNIIQCLIK 436

F +AA RFGH I + D R L+ F P +R D +++ L+

Sbjct 483 VFSTAAFRFGHATIHPLVRRLDARFQEHPGSHLPLRAAFFQPWRLLREGGVDPVLRGLLA 542

Query 437 EGSE-EAYDRYVSSAVSEHLFESTRGHKHALDLIAVNIQRGRDHGIPAYHYWRQYYRLRR 495

++ + D+ ++ ++E LF + LDL ++N+QRGRDHG+P Y+ WR++ L R

Sbjct 543 RPAKLQVQDQLMNEELTERLF--VLSNSGTLDLASINLQRGRDHGLPGYNEWREFCGLSR 600

Query 496 IISLDEFGEAGIAMKKA------YRDIRDVDLFPGGLLEPSMPGGVVGETFGHILANQFA 549

+ + + A + A Y+ ++D++ GGL E +PG G F I+ Q

Sbjct 601 LETWADLSAATANGRVADRILGLYQHPDNIDVWLGGLAESFLPGARTGPLFACIIGKQMR 660

Query 550 DLKFGDTYFFLHQQAPQGFRAAQIKAILSVTMSSIICANSAVTQAQPDPFYMASQLNLPR 609

L+ GD +++ + P F AQ + + +MS +IC NS ++ D F +

Sbjct 661 ALRDGDRFWW---ENPGVFTEAQRRELSRHSMSRVICDNSGLSHVPLDAFRVGQWPQEFE 717

Query 610 PCSDYSEMDVEPW 622

PC+ MD+ W

Sbjct 718 PCASIQGMDLGAW 730

>[P80025.1](https://www.ncbi.nlm.nih.gov/protein/P80025.1?report=genbank&log$=protalign&blast_rank=21&RID=0) RecName: Full=Lactoperoxidase; Short=LPO; Flags: Precursor

Length=712

Score = 213 bits (542), Expect = 6e-59, Method: Compositional matrix adjust.

Identities = 191/609 (31%), Positives = 283/609 (46%), Gaps = 82/609 (13%)

Query 57 YRQIDGRCNHPRN--YGSTGRPVKRYLRPHYQDKFGENLPRVYSV----TGQLLPSPRMV 110

YR I G CN+ R+ G+ R + R+L Y+D G LP ++ G +P R V

Sbjct 138 YRTITGDCNNRRSPALGAANRALARWLPAEYED--GLALPFGWTQRKTRNGFRVPLAREV 195

Query 111 SWK----LHPDQTAHDNNTMLVMQMGQFIDHDITRAPELS-GRNASIKCCGVPPKERLPD 165

S K L + N ++L MQ GQ +DHD+ APE G N K + +

Sbjct 196 SNKIVGYLDEEGVLDQNRSLLFMQWGQIVDHDLDFAPETELGSNEHSKTQCEEYCIQGDN 255

Query 166 CFPIDIPPGDPVFE---DCMEFFRSSPAVDNDGNIIYPREQINALTSFIDGSAVYGSDLD 222

CFPI P DP + CM FFR+ REQINA+TSF+D S VYGS+

Sbjct 256 CFPIMFPKNDPKLKTQGKCMPFFRAGFVCPTPPYQSLAREQINAVTSFLDASLVYGSEPS 315

Query 223 TYTWIR---SENGTGVFLNTHLVHGRERLPSHPHLGPESCVSSNT-AESYCQLAGDMRVN 278

+ +R S G HG LP + + P C NT A C LAGD R +

Sbjct 316 LASRLRNLSSPLGLMAVNQEAWDHGLAYLPFN-NKKPSPCEFINTTARVPCFLAGDFRAS 374

Query 279 EQPGLGSIHLLFHLHHNHIVRLLVAGILKKRGQPSSPERIAKFIQESSSALKEQIFQEVR 338

EQ L + H L HN + R L K+ P E+++QE R

Sbjct 375 EQILLATAHTLLLREHNRLAREL------KKLNPHWN--------------GEKLYQEAR 414

Query 339 KMLGAIIQKLTYCDWLPMILGPYLIDKFQLGCTRRSRYNSDLDPRVANSFLSAALRFGHT 398

K+LGA IQ +T+ D+LP++LG + Q YN+ +DPR++N F + A RFGH

Sbjct 415 KILGAFIQIITFRDYLPIVLG----SEMQKWIPPYQGYNNSVDPRISNVF-TFAFRFGHM 469

Query 399 LIPNVYNFGDKRIH-------------LKDTFNI-PDASIRYYDNIIQCLIKEGSE-EAY 443

+P+ + D+ +T+ I D I D +++ L+ + S+

Sbjct 470 EVPSTVSRLDENYQPWGPEAELPLHTLFFNTWRIIKDGGI---DPLVRGLLAKKSKLMNQ 526

Query 444 DRYVSSAVSEHLFESTRGHK-HALDLIAVNIQRGRDHGIPAYHYWRQYYRL---RRIISL 499

D+ V+S + LF+ T HK H DL A+N+QR RDHG+P Y+ WR + L + + L

Sbjct 527 DKMVTSELRNKLFQPT--HKIHGFDLAAINLQRCRDHGMPGYNSWRGFCGLSQPKTLKGL 584

Query 500 DEFGEAGIAMKK---AYRDIRDVDLFPGGLLEPSMPGGVVGETFGHILANQFADLKFGDT 556

+ I KK Y+ ++D++ GG EP + G VG +L QF ++ GD

Sbjct 585 QTVLKNKILAKKLMDLYKTPDNIDIWIGGNAEPMVERGRVGPLLACLLGRQFQQIRDGDR 644

Query 557 YFFLHQQAPQGFRAAQIKAILSVTMSSIICANSAVTQAQPDPFYMASQLNLPR---PCSD 613

+++ + P F Q ++ V+ S +IC N+ +T+ P + N P CS

Sbjct 645 FWW---ENPGVFTEKQRDSLQKVSFSRLICDNTHITKV---PLHAFQANNYPHDFVDCST 698

Query 614 YSEMDVEPW 622

++D+ PW

Sbjct 699 VDKLDLSPW 707

>[Q1ENI8.1](https://www.ncbi.nlm.nih.gov/protein/Q1ENI8.1?report=genbank&log$=protalign&blast_rank=22&RID=0) RecName: Full=Peroxidasin homolog; Flags: Precursor

Length=1285

Score = 217 bits (553), Expect = 7e-59, Method: Compositional matrix adjust.

Identities = 188/615 (31%), Positives = 304/615 (49%), Gaps = 83/615 (13%)

Query 56 RYRQIDGRCNHPRNYGSTG---RPVKRYLRPHYQDKF----GENLPRVYSVTGQLLPSPR 108

RYR DG+CN+ +N TG P++R L+P Y++ F G R+Y+ G LP+ R

Sbjct 633 RYRSFDGQCNN-KNKPMTGVSLMPLRRLLKPVYENGFNTPVGWEKGRLYN--GYPLPNVR 689

Query 109 MVSWKLHPDQ--TAHDNNTMLVMQMGQFIDHDITRAPELSGRN--ASIKCCGVPPKERLP 164

VS +L + T H + +VMQ GQF+DHD+T R+ A+ C E L

Sbjct 690 EVSRQLVATENITPHSKLSSMVMQWGQFVDHDLTHTVTALSRHSYATGAFCNRTC-ENLD 748

Query 165 DCFPIDIPPGDPVFED------CMEFFRSSPAVDNDG------NIIYPREQINALTSFID 212

CF I + P DP + C+EF RS+ AV G N + REQ+NALTSF+D

Sbjct 749 PCFNIPLSPNDPRVKSGSAKYPCIEFERSA-AVCGSGETSLVFNRVTYREQMNALTSFLD 807

Query 213 GSAVYGSDLDTYTWIR-SENGTGVF-LNTHLVHGRERLPSHPHLGPESCVSSNTAES--Y 268

S VYGS+ +R + N G+ + G+E LP + C + + E+

Sbjct 808 ASNVYGSNEVQAQELRDTYNNNGMLRFDITSEAGKEYLPFEKDSNMD-CRRNFSEENPIR 866

Query 269 CQLAGDMRVNEQPGLGSIHLLFHLHHNHIVRLLVAGILKKRGQPSSPERIAKFIQESSSA 328

C LAGD+R NEQ L + H +F HN RIAK ++ +

Sbjct 867 CFLAGDLRANEQLALAATHTIFIREHN---------------------RIAKKLKSMNGN 905

Query 329 LK-EQIFQEVRKMLGAIIQKLTYCDWLPMILGPYL-IDKFQLGCTRRSRYNSDLDPRVAN 386

E I+ E RK++GA++Q +TY W+P+I G ++KF +G + Y+ D+D V N

Sbjct 906 WDGEIIYHETRKIVGAMMQHITYKHWMPIIFGGQAQMNKF-VGTYQG--YDPDVDASVTN 962

Query 387 SFLSAALRFGHTLI-PNVYNFGDK-------RIHLKDTFNIPDASIRY--YDNIIQCLIK 436

+F +AA RFGHT+I P+++ G+ I L F P+ + D +++ L

Sbjct 963 AFATAAFRFGHTIINPSLFRLGNDFMPIKEGHIALHKAFFTPELVLTQGGVDPLLRGLFA 1022

Query 437 EGSEEAY-DRYVSSAVSEHLFESTRGHKHALDLIAVNIQRGRDHGIPAYHYWRQYYRLRR 495

+ + ++ + E LF +GH+ +LDL +NIQR RDHG+P+Y +R++ L

Sbjct 1023 SPLKHPMPTQLLNMELIEKLF--MKGHEVSLDLAVMNIQRSRDHGLPSYTEYRKFCNLPV 1080

Query 496 IISLDE---FGEAGIAMKK---AYRDIRDVDLFPGGLLEPSMPGGVVGETFGHILANQFA 549

+ ++ + + + ++K Y +++DL+ GG++E + G+ G TF I+ QF

Sbjct 1081 PVQWEDMKGYIKDDMIIQKLRGLYGVPQNIDLWVGGIVEEKLENGLFGPTFACIIGEQFR 1140

Query 550 DLKFGDTYFFLHQQAPQGFRAAQIKAILSVTMSSIICANS-AVTQAQPDPF-YMASQLNL 607

++ GD +++ + F Q++ I +T++ + C N + + Q D F Y

Sbjct 1141 KIRDGDRFWY---EKDGVFTPEQLREIKKITLARLFCDNGDNIDRIQKDVFMYPGMDKEN 1197

Query 608 PRPCSDYSEMDVEPW 622

C + M++ W

Sbjct 1198 YGTCQETEMMNLRAW 1212

>[Q8HYB7.2](https://www.ncbi.nlm.nih.gov/protein/Q8HYB7.2?report=genbank&log$=protalign&blast_rank=23&RID=0) RecName: Full=Thyroid peroxidase; Short=TPO; Flags: Precursor

Length=944

Score = 215 bits (548), Expect = 1e-58, Method: Compositional matrix adjust.

Identities = 179/613 (29%), Positives = 290/613 (47%), Gaps = 80/613 (13%)

Query 56 RYRQIDGRCN---HPRNYGSTGRPVKRYLRPHYQDKFGENLPRVYS----VTGQLLPSPR 108

+YR I G CN HPR +G++ + R+L P Y+D E PR ++ +G LP R

Sbjct 162 KYRLITGACNNRDHPR-WGASNTALARWLPPAYEDGISE--PRGWNPHVLYSGFPLPPVR 218

Query 109 MVSWKL--HPDQ--TAHDNNTMLVMQMGQFIDHDITRAPELSGRNASIKC--CGVPPKER 162

V+ ++ P++ T D + L+ GQ+IDHD+ P+ + A C + + R

Sbjct 219 EVTRQVIRVPNEAVTEDDQYSDLLTVWGQYIDHDVAFTPQSASGAAFGAGADCQLTCENR 278

Query 163 LPDCFPIDIPPGDPVFEDCMEFFRSSPAVDND------GNI--IYPREQINALTSFIDGS 214

P CFPI +PP D C+ F RSS A GN+ PR+Q+N LTSF+D S

Sbjct 279 SP-CFPIQLPP-DASGPACLPFSRSSAACGTGIQGAFFGNLSSANPRQQMNGLTSFLDAS 336

Query 215 AVYGSDLDTYTWIRSENGTGVFLNTHLVH---GRERLPSHPHLGPESCV----SSNTAES 267

VYGS +R+ L + H GR LP P +CV + TA +

Sbjct 337 TVYGSSPALEKQLRNWTSAEGLLRVNTRHWDAGRAHLPFMRPPAPLACVPEPGTRGTAGA 396

Query 268 YCQLAGDMRVNEQPGLGSIHLLFHLHHNHIVRLLVAGILKKRGQPSSPERIAKFIQESSS 327

C LAGD R +E P L ++H L+ HN + L K + S

Sbjct 397 PCFLAGDSRASEVPTLAALHTLWLREHNRLASAL------------------KALNAHWS 438

Query 328 ALKEQIFQEVRKMLGAIIQKLTYCDWLPMILGPYLIDKFQLGCTRRSRYNSDLDPRVANS 387

A + +QE RK++GA+ Q +T D++P +LGP + FQ Y+ +DP V+N

Sbjct 439 A--DTAYQEARKVVGALHQIITLRDYVPKVLGP---EAFQQHVGPYEGYDPTMDPTVSNV 493

Query 388 FLSAALRFGHTLIPNVYNFGDKR---------IHLKDTFNIPDASIRY--YDNIIQCLIK 436

F +AA R GH + + D R + L+D F P ++ D +++ L+

Sbjct 494 FSTAAFRLGHATVHPLVRRLDARFQEHPGLPPLGLQDAF-FPWRLLKEGGLDPLLRGLLA 552

Query 437 EGSE-EAYDRYVSSAVSEHLFESTRGHKHALDLIAVNIQRGRDHGIPAYHYWRQYYRLRR 495

++ ++ ++ ++E LF G +LDL ++N+QRGRDHG+P Y+ WR++ L R

Sbjct 553 SPAKLPVQEQLMNEELTERLF--VLGSSGSLDLASINLQRGRDHGLPGYNAWREFCGLGR 610

Query 496 IISLDEFGEAGIAMKKAYRDI------RDVDLFPGGLLEPSMPGGVVGETFGHILANQFA 549

+ + E A A R + ++D++ GGL EP +P G F ++ Q

Sbjct 611 LHTRAELRSAVANATLAGRIMDLYGHPDNIDVWLGGLAEPLLPRARTGPLFACLIGRQMK 670

Query 550 DLKFGDTYFFLHQQAPQGFRAAQIKAILSVTMSSIICANSAVTQAQPDPFYMASQLNLPR 609

L+ GD +++ ++ F Q + + ++S +IC N+ + D F ++

Sbjct 671 ALRDGDRFWW---ESSGVFTDEQRRELARHSLSRVICDNTGLPSVPADAFQVSRFPQDFE 727

Query 610 PCSDYSEMDVEPW 622

PC + ++++ W

Sbjct 728 PCENIPGLNLDVW 740

>[A5JUY8.1](https://www.ncbi.nlm.nih.gov/protein/A5JUY8.1?report=genbank&log$=protalign&blast_rank=24&RID=0) RecName: Full=Lactoperoxidase; Short=LPO; Short=WBLP; Flags:

Precursor

Length=712

Score = 210 bits (534), Expect = 6e-58, Method: Compositional matrix adjust.

Identities = 190/609 (31%), Positives = 281/609 (46%), Gaps = 82/609 (13%)

Query 57 YRQIDGRCNHPRN--YGSTGRPVKRYLRPHYQDKFGENLPRVYSV----TGQLLPSPRMV 110

YR I G CN+ R+ G+ R + R+L Y+D G LP ++ G +P R V

Sbjct 138 YRTITGDCNNRRSPALGAANRALARWLPAEYED--GLALPFGWTQRKTRNGFRVPLAREV 195

Query 111 SWK----LHPDQTAHDNNTMLVMQMGQFIDHDITRAPELS-GRNASIKCCGVPPKERLPD 165

S K L + N ++L MQ GQ +DHD+ APE G N K + +

Sbjct 196 SNKIVGYLDEEGVLDQNRSLLFMQWGQIVDHDLDFAPETELGSNEHSKTQCEEYCIQGDN 255

Query 166 CFPIDIPPGDPVFE---DCMEFFRSSPAVDNDGNIIYPREQINALTSFIDGSAVYGSDLD 222

CFPI P DP + CM FFR+ REQINA+TSF+D S VYGS+

Sbjct 256 CFPIMFPKNDPKLKTQGKCMPFFRAGFVCPTPPYQSLAREQINAVTSFLDASLVYGSEPS 315

Query 223 TYTWIR---SENGTGVFLNTHLVHGRERLPSHPHLGPESCVSSNT-AESYCQLAGDMRVN 278

+ +R S G HG LP + + P C NT A C LAGD R +

Sbjct 316 LASRLRNLSSPLGLMAVNQEAWDHGLAYLPFN-NKKPSPCEFINTTARVPCFLAGDFRAS 374

Query 279 EQPGLGSIHLLFHLHHNHIVRLLVAGILKKRGQPSSPERIAKFIQESSSALKEQIFQEVR 338

EQ L + H L HN + R L K+ P E+++QE R

Sbjct 375 EQILLATAHTLLLREHNRLAREL------KKLNPHWN--------------GEKLYQEAR 414

Query 339 KMLGAIIQKLTYCDWLPMILGPYLIDKFQLGCTRRSRYNSDLDPRVANSFLSAALRFGHT 398

K+LGA IQ +T+ D+LP++LG + Q YN+ +DPR++N F + A RFGH

Sbjct 415 KILGAFIQIITFRDYLPIVLG----SEMQKWIPPYQGYNNSVDPRISNVF-TFAFRFGHM 469

Query 399 LIPNVYNFGDKRIH-------------LKDTFNI-PDASIRYYDNIIQCLIKEGSE-EAY 443

+P+ + D+ +T+ I D I D + + L+ + S+

Sbjct 470 EVPSTVSRLDENYQPWGPEAELPLHTLFFNTWRIIKDGGI---DPLTRGLLAKKSKLMNQ 526

Query 444 DRYVSSAVSEHLFESTRGHK-HALDLIAVNIQRGRDHGIPAYHYWRQYYRL---RRIISL 499

D+ V+S + LF+ T HK H DL A+N+QR RDHG+P Y+ WR + L + + L

Sbjct 527 DKMVTSELRNKLFQPT--HKIHGFDLAAINLQRCRDHGMPGYNSWRGFCGLSQPKTLKGL 584

Query 500 DEFGEAGIAMKK---AYRDIRDVDLFPGGLLEPSMPGGVVGETFGHILANQFADLKFGDT 556

+ I KK Y+ ++D++ GG EP + G VG +L QF ++ GD

Sbjct 585 QTVLKNKILAKKLMDLYKTPDNIDIWIGGNAEPMVERGRVGPLLACLLGRQFQQIRDGDR 644

Query 557 YFFLHQQAPQGFRAAQIKAILSVTMSSIICANSAVTQAQPDPFYMASQLNLPR---PCSD 613

+++ + P F Q ++ + S +IC N+ +T+ P + N P CS

Sbjct 645 FWW---ENPGVFTEKQRDSLQKFSFSRLICDNTHITKV---PLHAFQANNYPHDFVDCST 698

Query 614 YSEMDVEPW 622

++D+ PW

Sbjct 699 VDKLDLSPW 707

>[A1KZ92.3](https://www.ncbi.nlm.nih.gov/protein/A1KZ92.3?report=genbank&log$=protalign&blast_rank=25&RID=0) RecName: Full=Peroxidasin-like protein; AltName: Full=Cardiac

peroxidase; AltName: Full=Vascular peroxidase 2; AltName: Full=polysomal

ribonuclease 1; Short=PRM1; Flags: Precursor

Length=1463

Score = 211 bits (538), Expect = 7e-57, Method: Compositional matrix adjust.

Identities = 183/619 (30%), Positives = 276/619 (45%), Gaps = 78/619 (13%)

Query 44 LEHCSRLTYDQLRYRQIDGRCNHPR--NYGSTGRPVKRYLRPHYQDKFGENLPRVYSV-- 99

L +CS + +YR DG CN+ + +G+ R L+P Y+D G PR +

Sbjct 715 LPNCSNRCF-HAKYRAHDGTCNNLQQPTWGAALTAFARLLQPAYRD--GIRAPRGLGLPV 771

Query 100 -TGQLLPSPRMVS--WKLHPDQTAHDNNTMLVMQMGQFIDHDITRA-PELS------GRN 149

+ Q LP PR+V+ W T + T ++M G F++HD+ P LS GR

Sbjct 772 GSRQPLPPPRLVATVWARAAAVTPDHSYTRMLMHWGWFLEHDLDHTVPALSTARFSDGRP 831

Query 150 ASIKCCGVPPKERLPDCFPIDIPPGDP--VFEDCMEFFRSSPAVDNDG-----NIIYPRE 202

S C PP CFP++ DP CM F RSSPA + + +Y RE

Sbjct 832 CSSVCTNDPP------CFPMNTRHADPRGTHAPCMLFARSSPACASGRPSATVDSVYARE 885

Query 203 QINALTSFIDGSAVYGSDLDTYTWIRSENGTGVFLNTHLVHGRERLPSHP-HLGPESCVS 261

QIN T++IDGS VYGS +R + L T P P GP + +

Sbjct 886 QINQQTAYIDGSNVYGSSERESQALRDPSVPRGLLKTGFPWPPSGKPLLPFSTGPPTECA 945

Query 262 SNTAESYCQLAGDMRVNEQPGLGSIHLLFHLHHNHIVRLLVAGILKKRGQPSSPERIAKF 321

ES C LAGD R NE L ++H L+ HN + L A G

Sbjct 946 RQEQESPCFLAGDHRANEHLALAAMHTLWFREHNRMATELSALNPHWEGN---------- 995

Query 322 IQESSSALKEQIFQEVRKMLGAIIQKLTYCDWLPMILGPYLIDKFQLGCTRRSRYNSDLD 381

++QE RK++GA +Q +TY WLP +LG D YN +++

Sbjct 996 ----------TVYQEARKIVGAELQHITYSHWLPKVLG----DPGTRMLRGYRGYNPNVN 1041

Query 382 PRVANSFLSAALRFGHTLI-PNVYNF-------GDKRIHLKDTFNIPDASIRY--YDNII 431

+ NSF +AA RFGHTLI P +Y + + P I+ D ++

Sbjct 1042 AGIINSFATAAFRFGHTLINPILYRLNATLGEISEGHLPFHKALFSPSRIIKEGGIDPVL 1101

Query 432 QCLIKEGSEEAYDRY-VSSAVSEHLFESTRGHKHALDLIAVNIQRGRDHGIPAYHYWRQY 490

+ L ++ Y +S +++ LF + + A+D A IQRGRDHGIP Y +R +

Sbjct 1102 RGLFGVAAKWRAPSYLLSPELTQRLFSA--AYSAAVDSAATIIQRGRDHGIPPYVDFRVF 1159

Query 491 YRLRRIISLDEF------GEAGIAMKKAYRDIRDVDLFPGGLLEPSMPGGVVGETFGHIL 544

L + + ++ E ++K Y D+DL+P ++E +PG VG T +

Sbjct 1160 CNLTSVKNFEDLQNEIKDSEIRQKLRKLYGSPGDIDLWPALMVEDLIPGTRVGPTLMCLF 1219

Query 545 ANQFADLKFGDTYFFLHQQAPQGFRAAQIKAILSVTMSSIICANS-AVTQAQPDPFYMAS 603

QF L+ GD +++ + P F AQ+ + ++S ++C N ++ Q Q D F A

Sbjct 1220 VTQFQRLRDGDRFWY---ENPGVFTPAQLTQLKQASLSRVLCDNGDSIQQVQADVFVKAE 1276

Query 604 QLNLPRPCSDYSEMDVEPW 622

CS+ ++D+ W

Sbjct 1277 YPQDYLNCSEIPKVDLRVW 1295

>[Q01603.2](https://www.ncbi.nlm.nih.gov/protein/Q01603.2?report=genbank&log$=protalign&blast_rank=26&RID=0) RecName: Full=Peroxidase; Short=DmPO; AltName: Full=Chorion peroxidase;

Flags: Precursor

Length=690

Score = 206 bits (523), Expect = 2e-56, Method: Compositional matrix adjust.

Identities = 177/608 (29%), Positives = 276/608 (45%), Gaps = 71/608 (12%)

Query 53 DQLRYRQIDGRCNHPRN--YGSTGRPVKRYLRPHYQDKFGENLPRVYSVTGQLLPSPRMV 110

++ YR +DG CNH G R L P Y D G + P SVTG LPS R+V

Sbjct 101 EKTAYRTLDGSCNHLEQPGLGVANSKYGRLLTPKYAD--GISAP-TRSVTGDELPSARLV 157

Query 111 SWKLHPDQTAHDNN-TMLVMQMGQFIDHDIT-RAPELSGRNASIKCC-------GVPPKE 161

S +Q D T+ MQ GQ + HD++ +A + +CC G+

Sbjct 158 SLVAFGEQDVPDPEFTLHNMQWGQIMTHDMSMQAGGTQSKKHPTRCCTDDGRLIGLDTAH 217

Query 162 RLPDCFPIDIPPGDPVFE----DCMEFFRSSPAVDNDGNIIY---PREQINALTSFIDGS 214

+ CF I +PP DP + +C+ F R+ D D N Y P EQ+ +TS++D S

Sbjct 218 KT--CFAIIVPPHDPAYSQVGTECLNFVRT--LTDRDSNCQYQGGPAEQLTVVTSYLDLS 273

Query 215 AVYGSDLDTYTWIRSENGTGVFLNTHLVHGRERLPSHPHLGPESCVSSNTAESYCQLAGD 274

VYG+ + + IR G + + +G + LP ++ + C + + +E C +GD

Sbjct 274 LVYGNSIQQNSDIREFQGGRMIVEER--NGAKWLPLSRNVTGD-CDAVDASE-VCYRSGD 329

Query 275 MRVNEQPGLGSIHLLFHLHHNHIVRLLVAGILKKRGQPSSPERIAKFIQESSSALKEQIF 334

+RVN+ PGL + + HN I L A P +R +F

Sbjct 330 VRVNQNPGLAILQTILLREHNRIADALSAL------NPHYDDRT--------------LF 369

Query 335 QEVRKMLGAIIQKLTYCDWLPMILGPYLIDKFQL-----GCTRRSRYNSDLDPRVANSFL 389

QE RK+ A Q+++Y +WLP+ LG + K +L + + ++ ++DP V N

Sbjct 370 QEARKINIAQYQQISYYEWLPIFLGGENMLKNRLIYKAPSGSYINDFDPNIDPSVLNEHA 429

Query 390 SAALRFGHTLIPNVYNFGDK------RIHLKDTFNIPDASIRYYDNIIQCLIKEGS--EE 441

+AA R+ H+ I + + + L D FN P I DN + EE

Sbjct 430 TAAFRYFHSQIEGRLDLLSELRQVLGSLTLSDWFNRP-GIIEVGDNFDSLTRGHATQPEE 488

Query 442 AYDRYVSSAVSEHLFESTRGHKHALDLIAVNIQRGRDHGIPAYHYWRQYYRLRRIISLDE 501

D + LF R DL +++IQR RDHG+ +Y+ R++ LRR S +

Sbjct 489 LTDINFDRQIKHFLFR--RNMPFGSDLRSLDIQRNRDHGLASYNDMREFCGLRRAHSWEG 546

Query 502 FGEAGI-----AMKKAYRDIRDVDLFPGGLLEPSMPGGVVGETFGHILANQFADLKFGDT 556

+G+ +K Y DVDL G LE + G + G TF IL QF + GD

Sbjct 547 YGDLISPPILEKLKSLYPSHEDVDLTVGASLEAHVAGALAGPTFLCILTEQFYRTRVGDR 606

Query 557 YFFLHQQAPQGFRAAQIKAILSVTMSSIICAN-SAVTQAQPDPFYMASQLNLPRPCSDYS 615

+FF + GF Q++ + +M+ ++C N + ++ QP+ F S N PCS+

Sbjct 607 FFFENGDKLTGFTPDQLEELRKASMARLLCDNGNHISSMQPEAFRTVSHSNPIIPCSNIP 666

Query 616 EMDVEPWL 623

++D+ W+

Sbjct 667 QVDLTKWI 674

>[P07202.4](https://www.ncbi.nlm.nih.gov/protein/P07202.4?report=genbank&log$=protalign&blast_rank=27&RID=0) RecName: Full=Thyroid peroxidase; Short=TPO; Flags: Precursor

Length=933

Score = 196 bits (499), Expect = 3e-52, Method: Compositional matrix adjust.

Identities = 179/619 (29%), Positives = 281/619 (45%), Gaps = 80/619 (13%)

Query 56 RYRQIDGRCN---HPRNYGSTGRPVKRYLRPHYQDKFGENLPRVYS----VTGQLLPSPR 108

+YR I G CN HPR +G++ + R+L P Y+D F + PR ++ G LP R

Sbjct 150 KYRPITGACNNRDHPR-WGASNTALARWLPPVYEDGFSQ--PRGWNPGFLYNGFPLPPVR 206

Query 109 MVSWKL----HPDQTAHDNNTMLVMQMGQFIDHDITRAPELSGRNA--SIKCCGVPPKER 162

V+ + + T D + L+M GQ+IDHDI P+ + + A C + + +

Sbjct 207 EVTRHVIQVSNEVVTDDDRYSDLLMAWGQYIDHDIAFTPQSTSKAAFGGGADCQMTCENQ 266

Query 163 LPDCFPIDIPP-GDPVF-EDCMEFFRSSPAVDND------GNI--IYPREQINALTSFID 212

P CFPI +P P C+ F+RSS A GN+ PR+Q+N LTSF+D

Sbjct 267 NP-CFPIQLPEEARPAAGTACLPFYRSSAACGTGDQGALFGNLSTANPRQQMNGLTSFLD 325

Query 213 GSAVYGSDLDTYTWIRSENGTGVFLNTHLV---HGRERLPSHPHLGPESCVSS----NTA 265

S VYGS +R+ L H GR LP P P +C

Sbjct 326 ASTVYGSSPALERQLRNWTSAEGLLRVHARLRDSGRAYLPFVPPRAPAACAPEPGIPGET 385

Query 266 ESYCQLAGDMRVNEQPGLGSIHLLFHLHHNHIVRLLVAGILKKRGQPSSPERIAKFIQES 325

C LAGD R +E P L ++H L+ HN + L A

Sbjct 386 RGPCFLAGDGRASEVPSLTALHTLWLREHNRLAAALKALNAHWSA--------------- 430

Query 326 SSALKEQIFQEVRKMLGAIIQKLTYCDWLPMILGPYLIDKFQLGCTRRSRYNSDLDPRVA 385

+ ++QE RK++GA+ Q +T D++P ILGP + FQ Y+S +P V+

Sbjct 431 -----DAVYQEARKVVGALHQIITLRDYIPRILGP---EAFQQYVGPYEGYDSTANPTVS 482

Query 386 NSFLSAALRFGHTLIPNVYNFGDKR---------IHLKDTFNIPDASIRY--YDNIIQCL 434

N F +AA RFGH I + D + L F P +R D +I+ L

Sbjct 483 NVFSTAAFRFGHATIHPLVRRLDASFQEHPDLPGLWLHQAFFSPWTLLRGGGLDPLIRGL 542

Query 435 IKEGSE-EAYDRYVSSAVSEHLFESTRGHKHALDLIAVNIQRGRDHGIPAYHYWRQYYRL 493

+ ++ + D+ ++ ++E LF + LDL ++N+QRGRDHG+P Y+ WR++ L

Sbjct 543 LARPAKLQVQDQLMNEELTERLF--VLSNSSTLDLASINLQRGRDHGLPGYNEWREFCGL 600

Query 494 RRIISLDEFGEAGIAMKKA------YRDIRDVDLFPGGLLEPSMPGGVVGETFGHILANQ 547

R+ + + A + A Y+ ++D++ GGL E +P G F ++ Q

Sbjct 601 PRLETPADLSTAIASRSVADKILDLYKHPDNIDVWLGGLAENFLPRARTGPLFACLIGKQ 660

Query 548 FADLKFGDTYFFLHQQAPQGFRAAQIKAILSVTMSSIICANSAVTQAQPDPFYMASQLNL 607

L+ GD +++ + F AQ + + ++S +IC N+ +T+ D F +

Sbjct 661 MKALRDGDWFWWENSHV---FTDAQRRELEKHSLSRVICDNTGLTRVPMDAFQVGKFPED 717

Query 608 PRPCSDYSEMDVEPWLIHF 626

C + M++E W F

Sbjct 718 FESCDSITGMNLEAWRETF 736

>[B3A0Q8.1](https://www.ncbi.nlm.nih.gov/protein/B3A0Q8.1?report=genbank&log$=protalign&blast_rank=28&RID=0) RecName: Full=Peroxidase-like protein 3

Length=294

Score = 174 bits (440), Expect = 3e-48, Method: Compositional matrix adjust.

Identities = 96/295 (33%), Positives = 164/295 (56%), Gaps = 8/295 (3%)

Query 331 EQIFQEVRKMLGAIIQKLTYCDWLPMILGPYLIDKFQLGCTRRSRYNSDLDPRVANSFLS 390

++IF+E +K++ A IQ++TY ++LP IL + +L Y DPR++N F +

Sbjct 2 DKIFEETKKIINAFIQRITYVEFLPEILNAETLKNNELNEGIYG-YEEFTDPRISNVFST 60

Query 391 AALRFGHTLIPNVYNFGDKRIHLKDTFNIPDASIRYYDN-IIQCLIKEGSEEAYDRYVSS 449

AA +F H+L P+ F L D FN + + + D + + + E DR+ S

Sbjct 61 AAFQFIHSLTPSSIEFDGTETPLMDLFN--NQNFLFLDTEKVAVYMMSSAGEPMDRFFSK 118

Query 450 AVSEHLFESTRGHKHALDLIAVNIQRGRDHGIPAYHYWRQYYRLRRIISLDEFGEAGIAM 509

+++H F+S + DL+A IQRGRDHG+P+Y+ +R++ L R+ EA +

Sbjct 119 QLTDHYFQSG---NISFDLVAQIIQRGRDHGLPSYNTFRRHCGLPRLPHFYAM-EAANVL 174

Query 510 KKAYRDIRDVDLFPGGLLEPSMPGGVVGETFGHILANQFADLKFGDTYFFLHQQAPQGFR 569

K Y +I DVD+F GG++E +PG ++G TF ++A QF D KFGD++++ +GF

Sbjct 175 KAVYHNIDDVDVFVGGMVEIPLPGSLLGPTFSCLIARQFRDTKFGDSHWYESADPKKGFN 234

Query 570 AAQIKAILSVTMSSIICANSAVTQAQPDPFYMASQLNLPRPCSDYSEMDVEPWLI 624

Q+K+I +++ + I+C ++ +PF + S N C+D +D +PW +

Sbjct 235 EGQLKSIKAMSAAKILCDGFGLSLIPENPFRVTSPSNPMVVCADLPGLDFQPWFL 289

>[P90820.3](https://www.ncbi.nlm.nih.gov/protein/P90820.3?report=genbank&log$=protalign&blast_rank=29&RID=0) RecName: Full=Heme peroxidase 2; Contains: RecName: Full=Heme

peroxidase 2 light chain; Contains: RecName: Full=Heme peroxidase

2 heavy chain; Flags: Precursor

Length=718

Score = 181 bits (460), Expect = 9e-48, Method: Compositional matrix adjust.

Identities = 164/566 (29%), Positives = 252/566 (45%), Gaps = 74/566 (13%)

Query 58 RQIDGRCNHPRN--YGSTGRPVKRYL-RPHYQDKFGENLPRVYSVTGQLLPSPRMVSWKL 114

R I G CN+ +N G++ P++R L Y D G R SV G LPS R++S ++

Sbjct 158 RSITGLCNNRQNSDLGNSVSPLRRILGAASYADGLGR--IRTRSVNGGELPSARLISNRI 215

Query 115 HPD---QTAHDNNTMLVMQMGQFIDHDITRAPELSGRNASIKCCGV--PPKERLPDCFPI 169

H D Q + L M +GQFI HD+ P R+ C P+ P+C PI

Sbjct 216 HDDRNNQVFSPSINHLHMIIGQFIAHDVVFMPSSVARDGGALDCSACNSPQRVSPNCAPI 275

Query 170 DIPPGDPVFED-CMEFFRSSPAVDNDGNIIYPREQINALTSFIDGSAVYGSDLDTYTWIR 228

IP DP F CM R+ +N G R QI + F+D S VYGS +R

Sbjct 276 TIPRNDPYFNTPCMRLTRALNGQENFG----VRSQIGQNSHFLDLSPVYGSADCEAETVR 331

Query 229 SENGTGVFLNTHLVHGRERLPSHPHLGPESCVSSNTAES---YCQLAGDMRVNEQPGLGS 285

S + L + L P++ SN S +C GD R + P L

Sbjct 332 SFQEGKMLTFDDLGYT---------LPPQNANDSNCQSSAPFHCFTCGDFRNSLHPALIP 382

Query 286 IHLLFHLHHNHIVRLLVAGILKKRGQPSSPERIAKFIQESSSALKEQIFQEVRKMLGAII 345

+H + HN + + R+A+ EQIFQ VRK++ +

Sbjct 383 VHTILIKEHNRLAEQV---------------RVAR-----PRFNDEQIFQLVRKIMIGMW 422

Query 346 QKLTYCDWLPMILGPYLIDKFQLGCTRRS---RYNSDLDPRVANSFLSAALRFGHT---- 398

Q + Y +++P L I F L R Y++ +DP ++ F AA RFGH+

Sbjct 423 QHIVYNEYIPKYLPRRTIRNFALRPLRNGVHRGYSTSVDPSISAEFAGAAFRFGHSQSRF 482

Query 399 ----LIPN-----VYNFGDKRIHLKDTFNIPDASIRYYDNIIQCLIKEGSEEAYDRYVSS 449

L N Y+ G+ I D + I ++ ++ +++ + ++ DRY S

Sbjct 483 DFPRLTENGRPAGNYDLGND-IFYADQMYL--TRIGGWEPVMNGMVRMPAMKS-DRYFSF 538

Query 450 AVSEHLFESTRGHKHALDLIAVNIQRGRDHGIPAYHYWRQYYRLRRIISLDE----FGEA 505

+ +FE + +DL+++NIQRGRD G+ Y +RQ L + S +E F +

Sbjct 539 GIRNQMFEIRGRNGSGVDLVSINIQRGRDMGLFPYIQYRQLVGLPTVTSFNELNTTFSQE 598

Query 506 GI-AMKKAYRDIRDVDLFPGGLLEPSMPGGVVGETFGHILANQFADLKFGDTYFFLH-QQ 563

I A++ Y D D+DL+ G +LE + GG +G T ++ QF LK GD +F+ +

Sbjct 599 NIQALRNVYSDPADIDLYVGIMLEEPLSGGQLGPTASFMIGEQFRALKRGDRFFYESIAE 658

Query 564 APQGFRAAQIKAILSVT-MSSIICAN 588

F +I + + T ++ IIC N

Sbjct 659 GTDNFTQEEISELRNKTSLAKIICTN 684

>[Q20616.1](https://www.ncbi.nlm.nih.gov/protein/Q20616.1?report=genbank&log$=protalign&blast_rank=30&RID=0) RecName: Full=Peroxidase skpo-1; AltName: Full=ShKT and peroxidase

domain-containing protein 1; Flags: Precursor

Length=655

Score = 173 bits (438), Expect = 5e-45, Method: Compositional matrix adjust.

Identities = 162/580 (28%), Positives = 264/580 (46%), Gaps = 81/580 (14%)

Query 56 RYRQIDGRCNHPRN--YGSTGRPVKRYLRPHYQDKFGENLPRVYSVTGQLLPSPRMVS-W 112

+YR +DG CN+ +N G+ R + Y D F + S + + P+PR VS +

Sbjct 142 KYRSMDGTCNNLQNPVKGAAFTAFTRLMPAAYDDGFNT----LVSASRRNRPNPREVSVF 197

Query 113 KLHPDQTAHDNNTMLVMQMGQFIDHDITRAPELSGRNASIKCCGVPPKERLPDCFPIDIP 172

L +++ + L+M GQF+ HDIT NA+ CG + P C I P

Sbjct 198 LLSSERSLPGHVNSLLMLFGQFVSHDITS-------NAAQNFCGC--QNSGPMCASIFAP 248

Query 173 PGDPVFEDCMEFFRSSPAVDNDGNIIYPREQINALTSFIDGSAVYGSDLDTYTWIRSENG 232

P D C+ F RS P + G REQ+N T+ ID S +YGS+ T +R

Sbjct 249 PSDRS-RRCIPFTRSFP-ICGTGQFGRVREQLNMNTAAIDASLIYGSEAITARSLR---- 302

Query 233 TGVFLNTHLVHGRERLPSHPHLGPESCVSSNTAESYCQLAGDMRVNEQPGLGSIHLLFHL 292

L T ++ GR P P+ P S AGD R GL ++H F

Sbjct 303 FAAMLRTSMIGGR-MFP--PNTNPGSLT-----------AGDGRAILFVGLAALHTSFLR 348

Query 293 HHNHIVRLLVAGILKKRGQPSSPERIAKFIQESSSALKEQIFQEVRKMLGAIIQKLTYCD 352

HN+ VA L+ + + +RI FQE RK++G I+Q +TY +

Sbjct 349 LHNN-----VAARLQNMNRHWNADRI---------------FQESRKIVGGIVQVITYQE 388

Query 353 WLPMILGPYLIDKFQLGCTRRSRYNSDLDPRVANSFLSAALRFGHTLIPNVYNFGDKRIH 412

++P ++G K LG + YN +++ V N F + A R H +I Y + +

Sbjct 389 FVPELIGD--ASKTILGAY--NGYNPNVEIGVLNEFAAGAYRL-HGMIQETYPLVNSQFQ 443

Query 413 LKDTFNIPDA--SIRYYDNIIQCLIKEGSEEAYDRY---VSSAVSEHLFESTRGHKHALD 467

+ + D +I + N I + + G R ++++V+E LF + +D

Sbjct 444 EVNRYRFIDGVNNINHVLNNIDAIYR-GMMTVPVRSPQRLTTSVTERLFGGS------VD 496

Query 468 LIAVNIQRGRDHGIPAYHYWRQYYRLRRIISLDEFGE-----AGIAMKKAYRDIRDVDLF 522

+ AVNIQRGRDHG+ +Y+ +R++ LR I S +++ E + + YR D+D +

Sbjct 497 MAAVNIQRGRDHGLRSYNDYRRFCNLRPITSFNDWPEVPDENVRQRIGQLYRTPDDLDFY 556

Query 523 PGGLLEPSMPGGVVGETFGHILANQFADLKFGDTYFFLHQQAPQGFRAAQIKAILSVTMS 582

GG+LE G ++G TF ++ QF L+ GD +++ + P F + Q+ + T+S

Sbjct 557 VGGILEQPAAGSLLGATFACVIGKQFERLRDGDRFYY---ENPGVFTSPQLAELKRTTLS 613

Query 583 SIICANSAVTQAQPDPFYMASQLNLPRPCSDYSEMDVEPW 622

++C + + PCS + +++E W

Sbjct 614 WVLCQTGDNMVRVGRRAFDIENGSRAVPCSSITGLNLEAW 653

>[B3A0P3.1](https://www.ncbi.nlm.nih.gov/protein/B3A0P3.1?report=genbank&log$=protalign&blast_rank=31&RID=0) RecName: Full=Peroxidase-like protein 2

Length=884

Score = 157 bits (398), Expect = 4e-39, Method: Compositional matrix adjust.

Identities = 79/237 (33%), Positives = 141/237 (59%), Gaps = 9/237 (4%)

Query 383 RVANSFLSAALRFGHTLIPNVYNFGDKRIHLKDTFNIPDASIRYYDNIIQCLIKEGSEEA 442

R++N F +AA++F L P+ + ++I L+DT+N P+ +I+ ++++ +EE

Sbjct 284 RISNVFATAAIKFVAALSPDYIDIRGRKIRLRDTYNNPEMVFDELPELIEEMLQQPTEE- 342

Query 443 YDRYVSSAVSEHLFESTRGHKHALDLIAVNIQRGRDHGIPAYHYWRQYYRLRRIISLDE- 501

+R++S +++H E + D +A IQRGRDHG+P Y+++RQ+ L + S +

Sbjct 343 RNRFISKELTDHFLED---GSRSFDEVASIIQRGRDHGVPPYNWFRQFCGLPIVRSFNSR 399

Query 502 -FGEAGIAMKKAYRDIRDVDLFPGGLLEPSMPGGVVGETFGHILANQFADLKFGDTYFFL 560

FG+AG ++K Y+ + D+D++ G + EP++PG ++GETF I A QF DLKFGD++F+L

Sbjct 400 VFGDAGPYLRKVYKSVDDIDIYTGAMSEPNLPGSLLGETFSCIFARQFRDLKFGDSFFYL 459

Query 561 HQQAPQGFRAAQIKAILSVTMSSIICANSAVTQAQPDPFYMASQLNLPRPCSDYSEM 617

+GF Q + + ++T+S +C + Q +P + S N P SD ++

Sbjct 460 SDDPLRGFSKEQRRELDTITLSKAMCFVFGLEAVQMNPLRVPSAQN---PLSDCEQI 513

>[Q6TMK4.1](https://www.ncbi.nlm.nih.gov/protein/Q6TMK4.1?report=genbank&log$=protalign&blast_rank=32&RID=0) RecName: Full=Peroxinectin A; Flags: Precursor

Length=531

Score = 143 bits (360), Expect = 3e-35, Method: Compositional matrix adjust.

Identities = 156/577 (27%), Positives = 251/577 (44%), Gaps = 105/577 (18%)

Query 57 YRQIDGRCNHPRN--YGSTGRPVKRYLRPHYQDKFGEN-LPRVYSVTGQLLPSPRMVSWK 113

+R G N+ +N GS P R P KF +N P ++T Q PS R +S

Sbjct 24 FRSYTGEGNNKQNPKQGSIFTPFIRLANPI---KFNKNGFP---NITNQ--PS-RAISNI 74

Query 114 LHPDQT---AHDNNTMLVMQMGQFIDHDITRA-PELSGRNASIKCCGVPPKERLPDCFPI 169

+ QT + ++ T + GQF+ H++ + PE P+ +PI

Sbjct 75 IFDQQTHIGSKEHLTDMFNMWGQFLIHNMALSKPE-------------------PNSWPI 115

Query 170 DIPPGDPVFE------DCMEFFRS----------SPAVDNDGNIIYPREQINALTSFIDG 213

+P D F+ M +FR+ VD DG EQIN+L S+IDG

Sbjct 116 KVPKCDQYFDPACIGNKTMNYFRTRATEVPCDVGKTVVDEDGKCY---EQINSLGSYIDG 172

Query 214 SAVYGSDLDTYTWIRSENGTGVFLNTHLVHGR--ERLPSHPHLGPESCVSSNTAESYCQL 271

+ +YG+ + +RS +G + + V + +P P + + S

Sbjct 173 NVLYGNSEEICKNLRSLSGGEMKMTVTDVGDLPPKNVPGVPMDNDANLFPIDQLYS---- 228

Query 272 AGDMRVNEQPGLGSIHLLFHLHHNHIVRLLVAGILKKRGQPSSPERIAKFIQESSSALKE 331

G+ R NE PGL SIH L HN + R KF + E

Sbjct 229 VGERRGNENPGLLSIHTLLLRDHNRLAR--------------------KFARLHPEWDDE 268

Query 332 QIFQEVRKMLGAIIQKLTYCDWLPMILGPYLIDKFQLGCTRRSRYNSDLDPRVANSFLSA 391

++FQ+ R + IQK+TY ++LP LG + + Y+++++ +V+N F +

Sbjct 269 RVFQQSRSCIIEQIQKITYDEYLPTTLGSF---------PSYTGYDANVNAQVSNEFTTT 319

Query 392 ALRFGHTLIP---NVYNFGDKRIH---LKDTFNIPDASIRYYDNIIQCLIKEGSEEAYDR 445

A RFGH+ + Y+ R+ +K ++ P A R + +I+ LI EE D

Sbjct 320 AFRFGHSEVGPFMEYYSENGTRLQPLPIKFSYFNPHALNRGVEPLIRGLII-NEEENIDI 378

Query 446 YVSSAVSEHLFESTRGHKHALDLIAVNIQRGRDHGIPAYHYWRQYYRLRRIISLDEFG-- 503

Y+ S + LF + + LDL + N+QR RDHGIP Y+ R+ LR + + +

Sbjct 379 YMISDLRNFLF--GKPGQGGLDLASRNLQRNRDHGIPPYNSLRRQLGLRPVQTWSDITSD 436

Query 504 -EAGIAMKKAYRDIRDVDLFPGGLLEPSMPGGVVGETFGHILANQFADLKFGDTYFFLHQ 562

+ +K AY+ + D+D + GGL E M G VG+TF I+ QF + GD +++

Sbjct 437 PQIQNRLKNAYKSVDDIDSYVGGLAEDHMEGSCVGQTFYLIIYEQFFRTRAGDRFWY--- 493

Query 563 QAPQGFRAAQIKAILSVTMSSIICANSAVTQAQPDPF 599

+ P+ R + + I S + QP+ F

Sbjct 494 ETPE-MRMVNRECETTTFAEVIKRTTSNIGYVQPNVF 529

>[Q9VQH2.2](https://www.ncbi.nlm.nih.gov/protein/Q9VQH2.2?report=genbank&log$=protalign&blast_rank=33&RID=0) RecName: Full=Dual oxidase

Length=1537

Score = 130 bits (328), Expect = 3e-30, Method: Compositional matrix adjust.

Identities = 165/620 (27%), Positives = 263/620 (42%), Gaps = 109/620 (18%)

Query 46 HCSRLTYDQLRYRQIDGRCN---HPRNYGSTGRPVKRYLRPHYQDKFGENLPRVYSVTGQ 102

HC Y Q ++ DG N HP ++GS + R P Y D VY++ G

Sbjct 58 HCYEKMYSQTEKQRYDGWYNNLAHP-DWGSVDSHLVRKAPPSYSDG-------VYAMAGA 109

Query 103 LLPSPRMVSWKLHPDQT---AHDNNTMLVMQMGQFIDHDITRAPELSGRNASIKCCGVPP 159

PS R +S + + N T L+ GQ + ++I A E G P

Sbjct 110 NRPSTRRLSRLFMRGKDGLGSKFNRTALLAFFGQLVANEIVMASE----------SGCPI 159

Query 160 KERLPDCFPIDIPPGDPVFE-DC-----MEFFRSSPAVDNDGNIIYPREQINALTSFIDG 213

+ I+I D +++ +C + F R++ D + PREQIN +T++IDG

Sbjct 160 -----EMHRIEIEKCDEMYDRECRGDKYIPFHRAAYDRDTGQSPNAPREQINQMTAWIDG 214

Query 214 SAVYGSDLDTYTWIRS-ENGTGVFLNTHLVHGRERLPSHPHLGPESCVSSNTAESYCQLA 272

S +Y + +RS NGT + + R + P V + L

Sbjct 215 SFIYSTSEAWLNAMRSFHNGTLLTEKDGKLPVRNTMRVPLFNNPVPSVMKMLSPERLFLL 274

Query 273 GDMRVNEQPGLGSIHLLFHLHHNHIVRLLVAGILKKRGQPSSPERIAKFIQESSSALKEQ 332

GD R N+ P + S +LF HN + + + KR P + E

Sbjct 275 GDPRTNQNPAILSFAILFLRWHNTLAQRI------KRVHPDWSD--------------ED 314

Query 333 IFQEVRKMLGAIIQKLTYCDWLPMILG----PYLIDKFQLGCTRRSRYNSDLDPRVANSF 388

I+Q R + A +Q + ++LP LG PY Y D+ P + + F

Sbjct 315 IYQRARHTVIASLQNVIVYEYLPAFLGTSLPPY------------EGYKQDIHPGIGHIF 362

Query 389 LSAALRFGHTLIPNVYNFGDKRIHLKDT-FNIP---------DASIRYYDNII-QCLIKE 437

+AA RFGHT+IP D + + K+T P D+S + D + + L+

Sbjct 363 QAAAFRFGHTMIPPGIYRRDGQCNFKETPMGYPAVRLCSTWWDSSGFFADTSVEEVLMGL 422

Query 438 GSE--EAYDRYVSSAVSEHLF---ESTRGHKHALDLIAVNIQRGRDHGIPAYHYWRQYYR 492

S+ E D + S V + LF E TR DL A+NI RGRD+G+P Y+ R+ Y

Sbjct 423 ASQISEREDPVLCSDVRDKLFGPMEFTRR-----DLGALNIMRGRDNGLPDYNTARESYG 477

Query 493 LRRIISLDEFG--------EAGIAMKKAYRD-IRDVDLFPGGLLEPSMPGGVVGETFGHI 543

L+R + + E +K+AY + + DVD++ GG+LE G GE F +

Sbjct 478 LKRHKTWTDINPPLFETQPELLDMLKEAYDNKLDDVDVYVGGMLESY---GQPGEFFTAV 534

Query 544 LANQFADLKFGDTYFFLHQQAPQGFRAAQIKAILSVTMSSIICANSAVTQA--QPDPFYM 601

+ QF L+ D ++F +++ F +I + +T+ II ++ V + Q D F

Sbjct 535 IKEQFQRLRDADRFWFENERNGI-FTPEEIAELRKITLWDIIVNSTDVKEEEIQKDVFMW 593

Query 602 ASQLNLPRPCSDYSEMDVEP 621

+ P+P + ++EP

Sbjct 594 RTGDPCPQPMQ-LNATELEP 612

>[O61213.2](https://www.ncbi.nlm.nih.gov/protein/O61213.2?report=genbank&log$=protalign&blast_rank=34&RID=0) RecName: Full=Dual oxidase 1; Short=DUOX1; AltName: Full=Blistered

cuticle protein 3; AltName: Full=NADPH thyroid oxidase

1; Flags: Precursor

Length=1497

Score = 130 bits (326), Expect = 6e-30, Method: Compositional matrix adjust.

Identities = 162/600 (27%), Positives = 262/600 (44%), Gaps = 110/600 (18%)

Query 57 YRQIDGRCNHPRN--YGSTGRPVKRYLRPHYQDKFGENLPRVYSVTGQLLPSPRMVSWKL 114

+++ DG N+ N +GS G + R R +Y D VYSV L PS R +S L

Sbjct 28 FQRYDGWYNNLANSEWGSAGSRLHRDARSYYSDG-------VYSVNNSL-PSARELSDIL 79

Query 115 HPDQTAHDNN---TMLVMQMGQFIDHDITRAPELSGRNASIKCCGVPPKERLPDCFPIDI 171

++ N T L+ Q + ++I ++ +S C P E L I +

Sbjct 80 FKGESGIPNTRGCTTLLAFFSQVVAYEIMQSNGVS--------C---PLETLK----IQV 124

Query 172 PPGDPVFEDCME------FFRSSPAVDNDGNIIYPREQINALTSFIDGSAVYGSDLDTYT 225

P D VF+ E F R+ + PREQIN TS+IDGS +YG+ T

Sbjct 125 PLCDNVFDKECEGKTEIPFTRAKYDKATGNGLNSPREQINERTSWIDGSFIYGT---TQP 181

Query 226 WIRSENGTGVFLNTHLVHGRERLPSHPHLGPESCVSSNTAESYCQ---------LAGDMR 276

W+ S F L G +P +P L +N A + GD R

Sbjct 182 WVSSLRS---FKQGRLAEG---VPGYPPLNNPHIPLNNPAPPQVHRLMSPDRLFMLGDSR 235

Query 277 VNEQPGLGSIHLLFHLHHNHIVRLLVAGILKKRGQPSSPERIAKFIQESSSALKEQIFQE 336

VNE PGL S L+ HN+ + +E EQIFQ

Sbjct 236 VNENPGLLSFGLILFRWHNY--------------------NANQIHREHPDWTDEQIFQA 275

Query 337 VRKMLGAIIQKLTYCDWLPMILGPYLIDKFQLGCTRRSRYNSDLDPRVANSFLSAALRFG 396

R+++ A +QK+ D++P +LG + + ++Y + P ++++F +AA RF

Sbjct 276 ARRLVIASMQKIIAYDFVPGLLGE------DVRLSNYTKYMPHVPPGISHAFGAAAFRFP 329

Query 397 HTLIP---------NVYNF-----GDKRIHL-KDTFNIPDASIRY-YDNIIQCLIKEGSE 440

H+++P N F G + L ++ +N D Y D II + + +E

Sbjct 330 HSIVPPAMLLRKRGNKCEFRTEVGGYPALRLCQNWWNAQDIVKEYSVDEIILGMASQIAE 389

Query 441 EAYDRYVSSAVSEHLFESTRGHKHALDLIAVNIQRGRDHGIPAYHYWRQYYRL--RRIIS 498

D V + +++F H LD++A +I RGRD+G+P Y+ R+ + L + +

Sbjct 390 RD-DNIVVEDLRDYIFGPM--HFSRLDVVASSIMRGRDNGVPPYNELRRTFGLAPKTWET 446

Query 499 LDE-FGEAGIA----MKKAY-RDIRDVDLFPGGLLEPSMPGGVVGETFGHILANQFADLK 552

++E F + A +K+ Y +I +D + GG+LE G GE F I+ +QF ++

Sbjct 447 MNEDFYKKHTAKVEKLKELYGGNILYLDAYVGGMLEGGENGP--GELFKEIIKDQFTRIR 504

Query 553 FGDTYFFLHQQAPQGFRAAQIKAILSVTMSSIICANSAV--TQAQPDPFYMASQLNLPRP 610

GD ++F + F +++ I S+T+ II A + + T Q D F+ P+P

Sbjct 505 DGDRFWF-ENKLNGLFTDEEVQMIHSITLRDIIKATTDIDETMLQKDVFFFKEGDPCPQP 563

>[Q8HZK2.2](https://www.ncbi.nlm.nih.gov/protein/Q8HZK2.2?report=genbank&log$=protalign&blast_rank=35&RID=0) RecName: Full=Dual oxidase 2; AltName: Full=NADH/NADPH thyroid

oxidase p138-tox; Flags: Precursor

Length=1545

Score = 124 bits (310), Expect = 5e-28, Method: Compositional matrix adjust.

Identities = 165/634 (26%), Positives = 256/634 (40%), Gaps = 105/634 (17%)

Query 17 CVYPDIYARRGDLGDSTGSLDFVSEADLEHCSRLTYDQLRYRQIDGRCNHPRNY--GSTG 74

C+ P+ G L T LD V D LT++ RY DG N+ R + G+ G

Sbjct 3 CIRPEALVLLGAL--LTVPLDPVGGQD---ALSLTWEVQRY---DGWFNNLRQHEHGAAG 54

Query 75 RPVKRYLRPHYQDKFGENLPRVYSVTGQ-LLPSPRMVS---WKLHPDQTAHDNNTMLVMQ 130

P++R + +Y D VY G+ LLP+PR +S + + N T+L +

Sbjct 55 SPLRRLVPANYADG-------VYQALGEPLLPNPRQLSHTTMRGPAGLRSIRNRTVLGVF 107

Query 131 MGQFIDHDITRAPELSGRNASIKCCGVPPKERLPDCFPIDIPPGDPVFED------CMEF 184

G + D+ SI+ G P + I IPPGDPVF+ + F

Sbjct 108 FGYHVLSDLV----------SIEKPGCPA-----EFLNIHIPPGDPVFDPHKSGDVVLPF 152

Query 185 FRSSPAVDNDGNIIYPREQINALTSFIDGSAVYGSDLDTYTWIRSENGTGVFLNTHLVHG 244

RS + + PR+ N +T ++DGSA+YGS +RS +G +

Sbjct 153 QRSRWDPNTGQSPSNPRDLTNEVTGWLDGSAIYGSSHSWSDELRSFSGGQLASGPDPAFP 212

Query 245 RERLPSHPHLGPESCVSSNTAESYCQLAGDMRVNEQPGLGSIHLLFHLHHNHIVRLLVAG 304

R+ P + G + N +P L ++ LL+ +HN +

Sbjct 213 RQAQDPLFMWTPPDPATGQRGPQGLYAFGAEQGNREPFLQALGLLWFRYHNLCAQ----- 267

Query 305 ILKKRGQPSSPERIAKFIQESSSALKEQIFQEVRKMLGAIIQKLTYCDWLPMILGPYLID 364

K +E E++FQ RK + A Q +T +WLP L

Sbjct 268 ---------------KLAREHPLWGDEELFQHARKRVIATYQSITMYEWLPSFL------ 306

Query 365 KFQLGCTRRSRYNSDLDPRVANSFLSAALRFGHTLIPNVYNFGDKRIHLKDTFNIPDASI 424

Q + Y LDP ++ FL+A+ +F T++P + H + N S

Sbjct 307 --QQTPPNYTEYRPFLDPSISPEFLAASEQFFSTMVPPGVYMRNASCHFQMVLNESYGSF 364

Query 425 RYYDNIIQCLIKE----GSEEAYDRYV---SSAVSEH----LFESTR------GHKHALD 467

I+E S EA ++ + +S +SE + E R G D

Sbjct 365 PALRVCNSYWIRENPNLNSAEAVNQLLLGMASQISELEDWIVVEDLRDYWPGPGKFSRTD 424

Query 468 LIAVNIQRGRDHGIPAYHYWRQYYRLRRIISLDEFG--------EAGIAMKKAYRDIRDV 519

+A +IQRGRD G+P+Y Q L + +F EA A+ +D+ +

Sbjct 425 YVASSIQRGRDMGLPSYTQALQALGLNTPKNWSDFNPNVDPQVLEATAALYN--QDLSRL 482

Query 520 DLFPGGLLEPSMPGGVVGETFGHILANQFADLKFGDTYFFLHQQAPQG-FRAAQIKAILS 578

+LF GGLLE G G F I+ +QF L+ GD Y+F + G F +I I S

Sbjct 483 ELFSGGLLESY---GDPGPLFSTIVLDQFVRLRDGDRYWF--ENTKNGLFSKEEIAEIRS 537

Query 579 VTMSSIICANSAVTQA--QPDPFYMASQLNLPRP 610

T+ ++ A + V+ + QP+ F P+P

Sbjct 538 TTLRDVLVAVTNVSSSALQPNVFIWNEDSPCPQP 571

>[Q9MZF4.1](https://www.ncbi.nlm.nih.gov/protein/Q9MZF4.1?report=genbank&log$=protalign&blast_rank=36&RID=0) RecName: Full=Dual oxidase 1; AltName: Full=NADPH thyroid oxidase

1; Short=Thyroid oxidase 1; Flags: Precursor

Length=1551

Score = 94.7 bits (234), Expect = 9e-19, Method: Compositional matrix adjust.

Identities = 140/593 (24%), Positives = 227/593 (38%), Gaps = 94/593 (16%)

Query 58 RQIDGRCNH--PRNYGSTGRPVKRYLRPHYQDKFGENLPRVYSVTGQ-LLPSPRMVS--- 111

++ DG N+ +GS G ++R + Y D VY G+ LP+PR +S

Sbjct 30 QRFDGWYNNLMEHKWGSKGSRLQRLVPASYADG-------VYQPLGEPHLPNPRDLSNAA 82

Query 112 WKLHPDQTAHDNNTMLVMQMGQFIDHDITRAPELSGRNASIKCCGVPPKERLPDCFPIDI 171

+ Q + N T+L + G + D+ S++ G P + I I

Sbjct 83 MRGPAGQASLRNRTVLGVFFGYHVLSDLV----------SVETPGCPA-----EFLNIRI 127

Query 172 PPGDPVFED------CMEFFRSSPAVDNDGNIIYPREQINALTSFIDGSAVYGSDLDTYT 225

PPGDPVF+ + F RS ++ + PR+ NA+T ++DGSA+YGS

Sbjct 128 PPGDPVFDPNGRGDVVLPFQRSRWDPESGQSPSNPRDLTNAVTGWLDGSAIYGSSHSWSD 187

Query 226 WIRSENGTGVFLNTHLVHGRERLPSHPHLGPESCVSSNTAESYCQLAGDMRVNEQPGLGS 285

+RS +G + R P S G R N P L +

Sbjct 188 ALRSFSGGQLASGPDPAFPRNAQPPLLMWSAPDPASGQRGPGGLYAFGAERGNRDPFLQA 247

Query 286 IHLLFHLHHNHIVRLLVAGILKKRGQPSSPERIAKFIQESSSALKEQIFQEVRKMLGAII 345

+ LL+ +HN + L R P + E++FQ RK + A

Sbjct 248 LGLLWFRYHNLCAQRLA------RQHPHWGD--------------EELFQHARKRVIATY 287

Query 346 QKLTYCDWLPMILGPYLIDKFQLGCTRRSRYNSDLDPRVANSFLSAALRFGHTLIPNVYN 405

Q + +WLP L Q + + YN LDP ++ FL A+ +F T++P

Sbjct 288 QNIALYEWLPSFL--------QQAPVKYAGYNPFLDPSISPEFLVASEQFFSTMVPPGIY 339

Query 406 FGDKRIHLKDTFNIPDA-----------------SIRYYDNIIQCLIKEGSE--EAYDRY 446

+ H ++ N + ++R +++ L+ S+ E D

Sbjct 340 MRNASCHFQEVINRNSSISRALRVCNSYWSRKHPNLRRAEDVDALLLGMASQIAEREDHV 399

Query 447 VSSAVSEHLFESTRGHKHALDLIAVNIQRGRDHGIPAYHYWRQYYRLRRIISLDEFGEA- 505

V V + S + + D +A +QRGRD G+P+Y R L I + A

Sbjct 400 VVEDVLDFWPGSLKFSR--TDHVAGCLQRGRDLGLPSYTKARAALGLPPITRWQDINPAL 457

Query 506 -----GIAMKKAYRDIRDVDLFPGGLLEPSMPGGVVGETFGHILANQFADLKFGDTYFFL 560

+ A +D+ G G F I+ NQF L+ GD Y+F

Sbjct 458 SQNNHTVLEATAALYNQDLSQLELLPGGLLESHGDPGPLFSAIVLNQFVRLRDGDRYWF- 516

Query 561 HQQAPQG-FRAAQIKAILSVTMSSIICA--NSAVTQAQPDPFYMASQLNLPRP 610

+ G F +I I + ++ ++ A N + QP+ F+ P+P

Sbjct 517 -ENTRNGLFSEEEIAEIRNTSLRDVLVAVTNMNPSTLQPNVFFWHMGDPCPQP 568

>[Q9ES45.1](https://www.ncbi.nlm.nih.gov/protein/Q9ES45.1?report=genbank&log$=protalign&blast_rank=37&RID=0) RecName: Full=Dual oxidase 2; AltName: Full=Large NOX 2; AltName:

Full=Long NOX 2; AltName: Full=NADH/NADPH thyroid oxidase

THOX2; AltName: Full=Thyroid oxidase 2; Flags: Precursor

Length=1517

Score = 89.4 bits (220), Expect = 4e-17, Method: Compositional matrix adjust.

Identities = 138/599 (23%), Positives = 238/599 (40%), Gaps = 109/599 (18%)

Query 58 RQIDGRCNHPRNY--GSTGRPVKRYLRPHYQDKFGENLPRVYSVTGQLLPSPRMVSWKLH 115

++ DG N+ + + G+ G ++R + +Y D + L LLP+ R++S +

Sbjct 36 QRYDGWFNNLKYHQRGAAGSQLRRLVPANYADGVYQALQE------PLLPNARLLSDAVS 89

Query 116 PDQ----TAHDNNTMLVMQMGQFIDHDITRAPELSGRNASIKCCGVPPKERLPDCFPIDI 171

+ +AH N T+L + G + D+ S++ G P + I I

Sbjct 90 KGKAGLPSAH-NRTVLGLFFGYHVLSDLV----------SVETPGCPA-----EFLNIYI 133

Query 172 PPGDPVFED------CMEFFRSSPAVDNDGNIIYPREQINALTSFIDGSAVYGSDLDTYT 225

P GDPVF+ + F RS + PR+ N +T ++DGSA+YGS

Sbjct 134 PRGDPVFDPDKRGNVVLPFQRSRWDRSTGQSPSNPRDLTNQVTGWLDGSAIYGSSHSWSD 193

Query 226 WIRSENGTGVFLNTHLVHGRERLPSHPH---------LGPESCVSSNTAESYCQLAGDMR 276

+RS +G L G + P+ P + P+ + G R

Sbjct 194 TLRSFSG------GQLASGPD--PAFPRNSQNSLLMWMAPDPATGQGGPQGLYAF-GAQR 244

Query 277 VNEQPGLGSIHLLFHLHHNHIVRLLVAGILKKRGQPSSPERIAKFIQESSSALKEQIFQE 336

N +P L ++ LL+ +HN + L QE E++FQ

Sbjct 245 GNREPFLQALGLLWFRYHNLCAKRLA--------------------QEHPHWGDEELFQH 284

Query 337 VRKMLGAIIQKLTYCDWLPMILGPYLIDKFQLGCTRRSRYNSDLDPRVANSFLSAALRFG 396

RK + A Q + WLP L Q S Y +DP ++ F++A+ +F

Sbjct 285 ARKRVIATYQNIALYQWLPSFL--------QKTPPEYSGYRPFMDPSISPEFVAASEQFL 336

Query 397 HTLIPNVYNFGDKRIHLKD-----TFNIP------------DASIRYYDNIIQCLIKEGS 439

T++P + H ++ + + P + S++ ++ Q L+ S

Sbjct 337 STMVPPGVYMRNSSCHFREFPKEGSSSSPALRVCNNYWIRENPSLKTAQDVDQLLLGMAS 396

Query 440 E--EAYDRYVSSAVSEHLFESTRGHKHALDLIAVNIQRGRDHGIPAYHYWRQYYRL---R 494

+ E DR V + ++ R + D +A +IQ GRD G+P+Y Q L +

Sbjct 397 QISELEDRIVIEDLRDYWPGPDRYSR--TDYVASSIQSGRDMGLPSYSQALQALGLEPPK 454

Query 495 RIISLDEFGEAGIAMKKAYRDIRDVDLFPGGLLEPSMPGGVVGETFGHILANQFADLKFG 554

+L+ + + A +D+ L G G F +I+ +QF L+ G

Sbjct 455 NWSALNPKVDPQVLEATAALYNQDLSRLELFLGGLLESHGDPGPLFSNIILDQFVRLRDG 514

Query 555 DTYFFLHQQAPQG-FRAAQIKAILSVTMSSIICANSAV--TQAQPDPFYMASQLNLPRP 610

D Y+F + G F +I I + T+ ++ A S V + QP+ F+ P+P

Sbjct 515 DRYWF--ENTRNGLFSKEEIAEIRNTTLRDVLVAVSNVDPSALQPNVFFWQEGAPCPQP 571

>[Q8HZK3.1](https://www.ncbi.nlm.nih.gov/protein/Q8HZK3.1?report=genbank&log$=protalign&blast_rank=38&RID=0) RecName: Full=Dual oxidase 1; Flags: Precursor

Length=1553

Score = 85.5 bits (210), Expect = 7e-16, Method: Compositional matrix adjust.

Identities = 136/593 (23%), Positives = 229/593 (39%), Gaps = 94/593 (16%)

Query 58 RQIDGRCNH--PRNYGSTGRPVKRYLRPHYQDKFGENLPRVYSVTGQ-LLPSPRMVS--- 111

++ DG N+ +GS G ++R + Y D VY G+ LP+PR +S

Sbjct 30 QRFDGWYNNLMEHKWGSKGSRLQRLVPASYADG-------VYQPLGEPHLPNPRDLSNTA 82

Query 112 WKLHPDQTAHDNNTMLVMQMGQFIDHDITRAPELSGRNASIKCCGVPPKERLPDCFPIDI 171

+ Q + N T+L + G + D+ SI+ G P + I I

Sbjct 83 MRGPAGQASLRNRTVLGVFFGYHVLSDLV----------SIEKPGCPA-----EFLNIHI 127

Query 172 PPGDPVFED------CMEFFRSSPAVDNDGNIIYPREQINALTSFIDGSAVYGSDLDTYT 225

PPGDPVF+ + F RS + + PR+ N +T ++DGSA+YGS

Sbjct 128 PPGDPVFDPHKSGDVVLPFQRSRWDPNTGQSPSNPRDLTNEVTGWLDGSAIYGSSHSWSD 187

Query 226 WIRSENGTGVFLNTHLVHGRERLPSHPHLGPESCVSSNTAESYCQLAGDMRVNEQPGLGS 285

+RS +G + R+ P + G + N +P L +

Sbjct 188 ELRSFSGGQLASGPDPAFPRQAQDPLFMWTPPDPATGQRGPQGLYAFGAEQGNREPFLQA 247

Query 286 IHLLFHLHHNHIVRLLVAGILKKRGQPSSPERIAKFIQESSSALKEQIFQEVRKMLGAII 345

+ LL+ +HN + K +E E++FQ RK + A

Sbjct 248 LGLLWFRYHNLCAQ--------------------KLAREHPLWGDEELFQHARKRVIATY 287

Query 346 QKLTYCDWLPMILGPYLIDKFQLGCTRRSRYNSDLDPRVANSFLSAALRFGHTLIPNVYN 405

Q +T +WLP L + + Y LDP ++ FL+A+ +F T++P

Sbjct 288 QSITMYEWLPSFL--------RKMPQEYTGYRPFLDPSISPEFLAASEQFFSTMVPPGVY 339

Query 406 FGDKRIHLKDTFNIPDASIR-------YY----------DNIIQCLIKEGSE--EAYDRY 446

+ H + N + R Y+ +++ L+ S+ E D

Sbjct 340 MRNASCHFQGVINRNSSVSRALRVCNSYWSREHPNLQRAEDVDALLLGMASQIAEREDHM 399

Query 447 VSSAVSEHLFESTRGHKHALDLIAVNIQRGRDHGIPAYHYWRQYYRLRRIISLDEFGEA- 505

V V + + + D +A +QRGRD G+P+Y R L + + A

Sbjct 400 VVEDVQDFWPGPLKFSR--TDHLASCLQRGRDLGLPSYTKARARLGLPPVTRWQDINPAL 457

Query 506 ----GIAMK-KAYRDIRDVDLFPGGLLEPSMPGGVVGETFGHILANQFADLKFGDTYFFL 560

GI ++ A +D+ G G F I+ +QF L+ GD Y+F

Sbjct 458 SRSDGIVLEATAALYNQDLSRLELLPGGLLESYGDPGPLFSTIVLDQFVRLRDGDRYWF- 516

Query 561 HQQAPQG-FRAAQIKAILSVTMSSIICANSAVTQA--QPDPFYMASQLNLPRP 610

+ G F +I I + ++ ++ A + +T QP+ F+ + P+P

Sbjct 517 -ENTKNGLFSEKEIAEIRNTSLRDVLVAVTNMTPGALQPNVFFWHAGDPCPQP 568

>[Q9NRD8.2](https://www.ncbi.nlm.nih.gov/protein/Q9NRD8.2?report=genbank&log$=protalign&blast_rank=39&RID=0) RecName: Full=Dual oxidase 2; AltName: Full=Large NOX 2; AltName:

Full=Long NOX 2; AltName: Full=NADH/NADPH thyroid oxidase

p138-tox; AltName: Full=NADPH oxidase/peroxidase DUOX2; AltName:

Full=NADPH thyroid oxidase 2; AltName: Full=Thyroid

oxidase 2; AltName: Full=p138 thyroid oxidase; Flags: Precursor

Length=1548

Score = 83.6 bits (205), Expect = 2e-15, Method: Compositional matrix adjust.

Identities = 140/596 (23%), Positives = 233/596 (39%), Gaps = 90/596 (15%)

Query 50 LTYDQLRYRQIDGRCNHPRNY--GSTGRPVKRYLRPHYQDKFGENLPRVYSVTGQLLPSP 107

L ++ RY DG N+ R++ G+ G ++R + +Y D + L LP+P

Sbjct 31 LPWEVQRY---DGWFNNLRHHERGAVGCRLQRRVPANYADGVYQALEE------PQLPNP 81

Query 108 RMVS---WKLHPDQTAHDNNTMLVMQMGQFIDHDITRAPELSGRNASIKCCGVPPKERLP 164

R +S + + N T+L + G + D+ S++ G P

Sbjct 82 RRLSNAATRGIAGLPSLHNRTVLGVFFGYHVLSDVV----------SVETPGCPA----- 126

Query 165 DCFPIDIPPGDPVFED------CMEFFRSSPAVDNDGNIIYPREQINALTSFIDGSAVYG 218

+ I IPPGDPVF+ + F RS + + PR+ N +T ++DGSA+YG

Sbjct 127 EFLNIRIPPGDPVFDPDQRGDVVLPFQRSRWDPETGRSPSNPRDLANQVTGWLDGSAIYG 186

Query 219 SDLDTYTWIRSENGTGVFLNTHLVHGRE-RLPSHPHLGPESCVSSNTAESYCQLAGDMRV 277

S +RS +G + R+ + P P+ N + R

Sbjct 187 SSHSWSDALRSFSGGQLASGPDPAFPRDSQNPLLMWAAPDPATGQNGPRGLYAFGAE-RG 245

Query 278 NEQPGLGSIHLLFHLHHNHIVRLLVAGILKKRGQPSSPERIAKFIQESSSALKEQIFQEV 337

N +P L ++ LL+ +HN + L R P + E++FQ

Sbjct 246 NREPFLQALGLLWFRYHNLWAQRLA------RQHPDWED--------------EELFQHA 285

Query 338 RKMLGAIIQKLTYCDWLPMILGPYLIDKFQLGCTRRSRYNSDLDPRVANSFLSAALRFGH 397

RK + A Q + +WLP L Q + Y LDP ++ F+ A+ +F

Sbjct 286 RKRVIATYQNIAVYEWLPSFL--------QKTLPEYTGYRPFLDPSISPEFVVASEQFFS 337

Query 398 TLIPNVYNFGDKRIHLKDTFNIPDASIRYYDNIIQCLIKE-----GSEEAYDRYV--SSA 450

T++P + H + N S + I+E ++E + + +S

Sbjct 338 TMVPPGVYMRNASCHFRKVLNKGFQSSQALRVCNNYWIRENPNLNSTQEVNELLLGMASQ 397

Query 451 VSEH----LFESTR------GHKHALDLIAVNIQRGRDHGIPAYHYWRQYYRL---RRII 497

+SE + E R G D +A +IQRGRD G+P+Y + L R

Sbjct 398 ISELEDNIVVEDLRDYWPGPGKFSRTDYVASSIQRGRDMGLPSYSQALLAFGLDIPRNWS 457

Query 498 SLDEFGEAGIAMKKAYRDIRDVDLFPGGLLEPSMPGGVVGETFGHILANQFADLKFGDTY 557

L+ + + A +D+ L G G F I+ +QF L+ GD Y

Sbjct 458 DLNPNVDPQVLEATAALYNQDLSQLELLLGGLLESHGDPGPLFSAIVLDQFVRLRDGDRY 517

Query 558 FFLHQQAPQG-FRAAQIKAILSVTMSSIICA--NSAVTQAQPDPFYMASQLNLPRP 610

+F + G F +I+ I + T+ ++ A N + QP+ F P+P

Sbjct 518 WF--ENTRNGLFSKKEIEDIRNTTLRDVLVAVINIDPSALQPNVFVWHKGAPCPQP 571

>[Q9NRD9.1](https://www.ncbi.nlm.nih.gov/protein/Q9NRD9.1?report=genbank&log$=protalign&blast_rank=40&RID=0) RecName: Full=Dual oxidase 1; AltName: Full=Large NOX 1; AltName:

Full=Long NOX 1; AltName: Full=NADPH thyroid oxidase 1;

AltName: Full=Thyroid oxidase 1; Flags: Precursor

Length=1551

Score = 81.3 bits (199), Expect = 1e-14, Method: Compositional matrix adjust.

Identities = 139/598 (23%), Positives = 227/598 (38%), Gaps = 104/598 (17%)

Query 58 RQIDGRCNH--PRNYGSTGRPVKRYLRPHYQDKFGENLPRVYSVTGQ-LLPSPRMVSWKL 114

++ DG N+ +GS G ++R + Y D VY G+ LP+PR +S +

Sbjct 30 QRFDGWYNNLMEHRWGSKGSRLQRLVPASYADG-------VYQPLGEPHLPNPRDLSNTI 82

Query 115 H--PDQTAH-DNNTMLVMQMGQFIDHDITRAPELSGRNASIKCCGVPPKERLPDCFPIDI 171

P A N T+L + G + D+ S++ G P + I I

Sbjct 83 SRGPAGLASLRNRTVLGVFFGYHVLSDLV----------SVETPGCPA-----EFLNIRI 127

Query 172 PPGDPVFED------CMEFFRSSPAVDNDGNIIYPREQINALTSFIDGSAVYGSDLDTYT 225

PPGDP+F+ + F RS + + PR+ N +T ++DGSA+YGS +++

Sbjct 128 PPGDPMFDPDQRGDVVLPFQRSRWDPETGRSPSNPRDPANQVTGWLDGSAIYGS---SHS 184

Query 226 WIRSENGTGVFLNTHLVHGRE----RLPSHPHL---GPESCVSSNTAESYCQLAGDMRVN 278

W + F L G + R +P L P+ N + R N

Sbjct 185 W---SDALRSFSRGQLASGPDPAFPRDSQNPLLMWAAPDPATGQNGPRGLYAFGAE-RGN 240

Query 279 EQPGLGSIHLLFHLHHNHIVRLLVAGILKKRGQPSSPERIAKFIQESSSALKEQIFQEVR 338

+P L ++ LL+ +HN + L R P + E++FQ R

Sbjct 241 REPFLQALGLLWFRYHNLWAQRLA------RQHPDWED--------------EELFQHAR 280

Query 339 KMLGAIIQKLTYCDWLPMILGPYLIDKFQLGCTRRSRYNSDLDPRVANSFLSAALRFGHT 398

K + A Q + +WLP L Q + Y LDP +++ F++A+ +F T

Sbjct 281 KRVIATYQNIAVYEWLPSFL--------QKTLPEYTGYRPFLDPSISSEFVAASEQFLST 332

Query 399 LIPNVYNFGDKRIHLKDTFNIPDASIR-------YYDNIIQCLIKEGSEEAYDRYVSSAV 451

++P + H + N + R Y+ L +A ++S +

Sbjct 333 MVPPGVYMRNASCHFQGVINRNSSVSRALRVCNSYWSREHPSLQSAEDVDALLLGMASQI 392

Query 452 SEH----LFESTRGHKHA------LDLIAVNIQRGRDHGIPAYHYWRQYYRLRRIISLDE 501

+E L E R D +A +QRGRD G+P+Y R L I +

Sbjct 393 AEREDHVLVEDVRDFWPGPLKFSRTDHLASCLQRGRDLGLPSYTKARAALGLSPITRWQD 452

Query 502 FGEA------GIAMKKAYRDIRDVDLFPGGLLEPSMPGGVVGETFGHILANQFADLKFGD 555

A + A +D+ G F I+ QF L+ GD

Sbjct 453 INPALSRSNDTVLEATAALYNQDLSWLELLPGGLLESHRDPGPLFSTIVLEQFVRLRDGD 512

Query 556 TYFFLHQQAPQG-FRAAQIKAILSVTMSSIICA--NSAVTQAQPDPFYMASQLNLPRP 610

Y+F + G F +I+ I + T+ ++ A N + QP+ F P+P

Sbjct 513 RYWF--ENTRNGLFSKKEIEEIRNTTLQDVLVAVINIDPSALQPNVFVWHKGDPCPQP 568

>[Q8CIY2.1](https://www.ncbi.nlm.nih.gov/protein/Q8CIY2.1?report=genbank&log$=protalign&blast_rank=41&RID=0) RecName: Full=Dual oxidase 1; Flags: Precursor

Length=1551

Score = 78.6 bits (192), Expect = 1e-13, Method: Compositional matrix adjust.

Identities = 132/600 (22%), Positives = 226/600 (38%), Gaps = 108/600 (18%)

Query 58 RQIDGRCNH--PRNYGSTGRPVKRYLRPHYQDKFGENLPRVYSVTGQLLPSPRMVSWKLH 115

++ DG N+ +GS G ++R + Y D + L Y LP+PR +S ++

Sbjct 30 QRFDGWYNNLMEHRWGSKGSRLQRLVPASYADGVYQPLREPY------LPNPRHLSNRVM 83

Query 116 ---PDQTAHDNNTMLVMQMGQFIDHDITRAPELSGRNASIKCCGVPPKERLPDCFPIDIP 172

Q + N T+L + G + D+ S++ G P + I IP

Sbjct 84 RGPAGQPSLRNRTVLGVFFGYHVLSDLV----------SVETPGCPA-----EFLNIYIP 128

Query 173 PGDPVFED------CMEFFRSSPAVDNDGNIIYPREQINALTSFIDGSAVYGSDLDTYTW 226

GDPVF+ + F RS + PR+ N +T ++DGSA+YGS

Sbjct 129 RGDPVFDPDKRGNVVLPFQRSRWDRSTGQSPSNPRDLTNQVTGWLDGSAIYGSSHSWSDT 188

Query 227 IRSENGTGVFLNTHLVHGRERLPSHPH---------LGPESCVSSNTAESYCQLAGDMRV 277

+RS +G L G + P+ P + P+ + G R

Sbjct 189 LRSFSG------GQLASGPD--PAFPRNSQNSLLMWMAPDPATGQGGPQGLYAF-GAQRG 239

Query 278 NEQPGLGSIHLLFHLHHNHIVRLLVAGILKKRGQPSSPERIAKFIQESSSALKEQIFQEV 337

N +P L ++ LL+ +HN + L QE E++FQ

Sbjct 240 NREPFLQALGLLWFRYHNLCAKRLA--------------------QEHPHWGDEELFQHA 279

Query 338 RKMLGAIIQKLTYCDWLPMILGPYLIDKFQLGCTRRSRYNSDLDPRVANSFLSAALRFGH 397

RK + A Q + +WLP L + Y+ LDP ++ F+ A+ +F

Sbjct 280 RKRVIATYQNIAMYEWLPSFL--------KQTPPEYPGYHPFLDPSISPEFVVASEQFLS 331

Query 398 TLIPNVYNFGDKRIHLKDTFN-----------------IPDASIRYYDNIIQCLIKEGSE 440

T++P + H + N + ++ +++ L+ S+

Sbjct 332 TMVPPGVYMRNASCHFQGIANRNSSVSGALRVCNSYWSRENPKLQRAEDVDALLLGMASQ 391

Query 441 --EAYDRYVSSAVSEHLFESTRGHKHALDLIAVNIQRGRDHGIPAYHYWRQYYRLRRIIS 498

E D V V + + + D +A +QRGRD G+P+Y R+ L +

Sbjct 392 IAEREDHLVVEDVQDFWPGPLKFSR--TDYLASCLQRGRDLGLPSYTKAREALGLPPVSH 449

Query 499 LDEFGEA------GIAMKKAYRDIRDVDLFPGGLLEPSMPGGVVGETFGHILANQFADLK 552

+ A + A +D+ G G F I+ +QF L+

Sbjct 450 WQDINPALSRSNGTVLEATAALYNQDLSRLELLAGGLLESHGDPGPLFSAIVLDQFVRLR 509

Query 553 FGDTYFFLHQQAPQGFRAAQIKAILSVTMSSIICANSAV--TQAQPDPFYMASQLNLPRP 610

GD Y+F + + F +I I + ++ I+ A + V + QP F+ + P+P

Sbjct 510 DGDRYWFENNRNGL-FSKEEIAEIRNTSLRDILVAVTNVDPSALQPSVFFWLAGDPCPQP 568

Lambda K H a alpha

0.322 0.139 0.431 0.792 4.96

Gapped

Lambda K H a alpha sigma

0.267 0.0410 0.140 1.90 42.6 43.6

Effective search space used: 60968746960

Database: Non-redundant UniProtKB/SwissProt sequences

Posted date: Dec 13, 2019 3:34 AM

Number of letters in database: 178,974,751

Number of sequences in database: 473,533

Matrix: BLOSUM62

Gap Penalties: Existence: 11, Extension: 1

Neighboring words threshold: 11

Window for multiple hits: 40
