# Supplementary material for: Mantle Modularity Underlies the Plasticity of the Molluscan Shell: Supporting Data From Cepaea nemoralis
Source: Front Genet. 2021 Feb 5;12:622400. doi: 10.3389/fgene.2021.622400 (PMC7894901; doi:10.3389/fgene.2021.622400)
Supplement: Supplementary file 10 [file Data_Sheet_10.docx]

# hmmscan :: search sequence(s) against a profile database

# HMMER 3.3 (Nov 2019); http://hmmer.org/

# Copyright (C) 2019 Howard Hughes Medical Institute.

# Freely distributed under the BSD open source license.

# - - - - - - - - - - - - - - - - - - - - - - - - - - - - - - - - - - - -

# query sequence file: /Users/djackson/Documents/Data/Manuscripts/2020/Cepaea_biomin_ISH/Genes/Mantle_reference_(R27072837)/Cnemoralis_Mantle_reference_R27072837_faa.txt

# target HMM database: /usr/local/bin/Pfam_latest/Pfam-A.hmm

# output directed to file: pfam_out.txt

# per-seq hits tabular output: pfam_tblout.txt

# per-dom hits tabular output: pfam_domtblout.txt

# number of worker threads: 4

# - - - - - - - - - - - - - - - - - - - - - - - - - - - - - - - - - - - -

Query: R27072837 [L=213]

Description: TransAbyss assembly 2 (filtered min reads 10, dedupe95) len=2379 num_reads=1735589 avg_cov=69372.3 contig_cov=100.0% (contig_123 from old CLC assemly 9)cds start = 623 cds stop = 1261 strand = -protein length = 213 strand = +

Scores for complete sequence (score includes all domains):

--- full sequence --- --- best 1 domain --- -#dom-

E-value score bias E-value score bias exp N Model Description

------- ------ ----- ------- ------ ----- ---- -- -------- -----------

[No hits detected that satisfy reporting thresholds]

Domain annotation for each model (and alignments):

[No targets detected that satisfy reporting thresholds]

Internal pipeline statistics summary:

-------------------------------------

Query sequence(s): 1 (213 residues searched)

Target model(s): 18259 (3090017 nodes)

Passed MSV filter: 239 (0.0130894); expected 365.2 (0.02)

Passed bias filter: 153 (0.00837943); expected 365.2 (0.02)

Passed Vit filter: 10 (0.000547675); expected 18.3 (0.001)

Passed Fwd filter: 0 (0); expected 0.2 (1e-05)

Initial search space (Z): 18259 [actual number of targets]

Domain search space (domZ): 0 [number of targets reported over threshold]

# CPU time: 0.33u 0.68s 00:00:01.01 Elapsed: 00:00:04.52

# Mc/sec: 145.33

//

[ok]

# hmmscan :: search sequence(s) against a profile database

# HMMER 3.3 (Nov 2019); http://hmmer.org/

# Copyright (C) 2019 Howard Hughes Medical Institute.

# Freely distributed under the BSD open source license.

# - - - - - - - - - - - - - - - - - - - - - - - - - - - - - - - - - - - -

# query sequence file: /Users/djackson/Documents/Data/Manuscripts/2020/Cepaea_biomin_ISH/Genes/Pif_like_(R27072766)/Cnemoralis_Pif_like_R27072766_faa.txt

# target HMM database: /usr/local/bin/Pfam_latest/Pfam-A.hmm

# output directed to file: pfam_out.txt

# per-seq hits tabular output: pfam_tblout.txt

# per-dom hits tabular output: pfam_domtblout.txt

# number of worker threads: 4

# - - - - - - - - - - - - - - - - - - - - - - - - - - - - - - - - - - - -

Query: R27072766 [L=727]

Description: Pif TransAbyss assembly 2 (filtered min reads 10, dedupe95) len=2716 num_reads=5670257 avg_cov=209491.4 contig_cov=100.0% (contig_821 from old CLC assemly 9)cds start = 286 cds stop = 2466 strand = -protein length = 727 strand = +

Scores for complete sequence (score includes all domains):

--- full sequence --- --- best 1 domain --- -#dom-

E-value score bias E-value score bias exp N Model Description

------- ------ ----- ------- ------ ----- ---- -- -------- -----------

5.9e-11 42.4 17.8 2.6e-07 30.7 3.7 4.8 4 CBM_14 Chitin binding Peritrophin-A domain

------ inclusion threshold ------

0.095 12.4 4.8 0.36 10.5 0.1 3.2 4 Baculo_VP91_N Viral capsid protein 91 N-terminal

0.66 8.8 34.4 0.92 8.3 34.4 1.2 1 Dapper Dapper

5.1 6.2 40.2 7.6 5.6 40.2 1.2 1 SOG2 RAM signalling pathway protein

Domain annotation for each model (and alignments):

>> CBM_14 Chitin binding Peritrophin-A domain

# score bias c-Evalue i-Evalue hmmfrom hmm to alifrom ali to envfrom env to acc

--- ------ ----- --------- --------- ------- ------- ------- ------- ------- ------- ----

1 ! 30.7 3.7 5.6e-11 2.6e-07 6 53 .] 323 374 .. 317 374 .. 0.87

2 ? 1.3 0.1 0.085 3.9e+02 27 46 .. 402 421 .. 394 424 .. 0.84

3 ! 9.8 0.4 0.00019 0.86 14 47 .. 442 475 .. 426 481 .. 0.85

4 ! 7.2 0.1 0.0012 5.7 26 49 .. 506 529 .. 492 533 .. 0.86

Alignments for each domain:

== domain 1 score: 30.7 bits; conditional E-value: 5.6e-11

CBM_14 6 dglfadptdCskYyvCs.ngepv....eftCpeglvFdeetqtCdypenvvqC 53

g+ + p+ C+ Y++C +g + ++Cp+g ++++++ +Cd+++n v+C

R27072766 323 VGYAPLPGYCDAYVQCRfYGALPtavdIRRCPSGNYWNQDKLVCDFQDN-VKC 374

578888***********7444447889**********************.*99 PP

== domain 2 score: 1.3 bits; conditional E-value: 0.085

CBM_14 27 veftCpeglvFdeetqtCdy 46

++ Cpe +++ t C+

R27072766 402 TRVACPERRLYNSVTCGCTD 421

5778**********999986 PP

== domain 3 score: 9.8 bits; conditional E-value: 0.00019

CBM_14 14 dCskYyvCsngepveftCpeglvFdeetqtCdyp 47

d++++++ + +++v++ Cp+ l ++e+t +C+ +

R27072766 442 DNTGFMQFTGNGWVRMACPATLGYNEQTCRCTDK 475

56667777788999*****************976 PP

== domain 4 score: 7.2 bits; conditional E-value: 0.0012

CBM_14 26 pveftCpeglvFdeetqtCdypen 49

+++++Cp+ lv++++ +C y+++

R27072766 506 WIRRPCPATLVYHADICVCSYDQT 529

56899****************986 PP

>> Baculo_VP91_N Viral capsid protein 91 N-terminal

# score bias c-Evalue i-Evalue hmmfrom hmm to alifrom ali to envfrom env to acc

--- ------ ----- --------- --------- ------- ------- ------- ------- ------- ------- ----

1 ? -2.4 0.0 0.73 3.3e+03 35 63 .. 43 75 .. 10 121 .. 0.57

2 ? -2.1 0.1 0.6 2.7e+03 146 160 .. 371 385 .. 360 387 .. 0.79

3 ? 2.4 0.3 0.024 1.1e+02 118 150 .. 388 420 .. 367 439 .. 0.76

4 ? 10.5 0.1 8e-05 0.36 119 152 .. 442 475 .. 426 492 .. 0.81

Alignments for each domain:

== domain 1 score: -2.4 bits; conditional E-value: 0.73

Baculo_VP91_N 35 rrlvleeyl....ksvgadestfdvlsyvskvl 63

+ ++++y ++ +++ t d l y+ +l

R27072766 43 LESLVKQYQsdagQQQQTHYFTQDQLDYIKTLL 75

334445554333334444455555555555543 PP

== domain 2 score: -2.1 bits; conditional E-value: 0.6

Baculo_VP91_N 146 qlkCvsrpvCdnksa 160

++kC +++ C n++a

R27072766 371 NVKCTPVNNCPNHKA 385

458*******99876 PP

== domain 3 score: 2.4 bits; conditional E-value: 0.024

Baculo_VP91_N 118 kdaekFvlrvDdgdvkvkCPalavFdgvqlkCv 150

d ++++ ++ +v CP+ ++ v C

R27072766 388 GDWAAYSIFNGANWTRVACPERRLYNSVTCGCT 420

56667777777899************9995553 PP

== domain 4 score: 10.5 bits; conditional E-value: 8e-05

Baculo_VP91_N 119 daekFvlrvDdgdvkvkCPalavFdgvqlkCvsr 152

d++ F+ + +g+v++ CPa+ ++ +C +

R27072766 442 DNTGFMQFTGNGWVRMACPATLGYNEQTCRCTDK 475

788899999******************9999754 PP

>> Dapper Dapper

# score bias c-Evalue i-Evalue hmmfrom hmm to alifrom ali to envfrom env to acc

--- ------ ----- --------- --------- ------- ------- ------- ------- ------- ------- ----

1 ? 8.3 34.4 0.0002 0.92 275 504 .. 24 254 .. 5 300 .. 0.51

Alignments for each domain:

== domain 1 score: 8.3 bits; conditional E-value: 0.0002

Dapper 275 k....dteseaeldgqkpetksllsseslssvqqssstkavleassldgYIdkLlqrraqkvrtnkprtsvetd..psksil..rqkslclrqpsgqksq 366

+ d+ ++ d q+ et + ++++ ++ +++ ++ + +++ YI+ Ll++ + + + ++++ + p+ s+ q++++ +p++ +

R27072766 24 MtaayDQ--QTGADDQEIETWLESLVKQYQ-SDAGQQQQTHYFTQDQLDYIKTLLNKMKPTAVNDDLKQEIVDHffPKDSEEnvTQEEQQTTTPAD---S 117

2333333..344555555554444333333.333555666556666899*******9988777777777765542222222211444444444444...3 PP

Dapper 367 sqtadskeskqklllsagresaeeastssplkqlskeskaeqqeskkvlvsatkkekpasakelqskelnksskakkasqeealsevakkseek..ksps 464

+++a++ +s+++++++++++ ae +++ ++ +q+ + +++e+q +++ ++++t +++++ ++ +q+++++ +++++q+e++++++ +see+ ++++

R27072766 118 EEQAKEDSSEEQVTQEQQTTPAESVEQVKQEQQTITPEDSEEQTTQQEQQTTTPANSEEDTTLEQQTTTPAD-SEEDTTQQEEQTTTPADSEEDttQQEQ 216

344444445555566666666666666666566555455555555554333332233333333333344444.345555555555555555555354555 PP

Dapper 465 pesspkeskkleerpaleqssegsssqsleessskassel 504

++ p +s+ ee+++++ ++ ++s + ++++++++ l

R27072766 217 QTTTPADSE--EEKQDSDDTDGDDNSDEDSTETTTSTTTL 254

555555553..44444444444443333332222222222 PP

>> SOG2 RAM signalling pathway protein

# score bias c-Evalue i-Evalue hmmfrom hmm to alifrom ali to envfrom env to acc

--- ------ ----- --------- --------- ------- ------- ------- ------- ------- ------- ----

1 ? 5.6 40.2 0.0017 7.6 176 362 .. 99 288 .. 65 315 .. 0.57

Alignments for each domain:

== domain 1 score: 5.6 bits; conditional E-value: 0.0017

SOG2 176 ksksisttssgssst.lvssaesstesrassvtytarrRhagsfsaqdveqGaaltplrsd.qipfnrsrrssssgnssspasngaqsetsersassdg. 272

+s+ + t++++++ t + s++++++ s++++vt+ +++ +a+s + q ++ + +tp s+ q+++ ++++++++++s+ ++ +q++t++ +++d+

R27072766 99 DSEENVTQEEQQTTTpADSEEQAKEDSSEEQVTQEQQTTPAESVE-QVKQEQQTITPEDSEeQTTQ--QEQQTTTPANSEEDTTLEQQTTTPADSEEDTt 195

344444444444444044566667778888899999889999888.55555566777888866666..67777777778888888887777777888887 PP

SOG2 273 vqssasstPlgegstspp..salsrsslsttgatstltsssksrs........ssrsntnasastprslattpappasaesfastlt.asrinslaakdv 361

q ++++tP ++++++ +++++++ s+++++ + ++ s ++t +s++t ++ +tt+ +++++++ ++t+t +++ + ++++

R27072766 196 QQEEQTTTPADSEEDTTQqeQQTTTPADSEEEKQDSDDT-----DgddnsdedS--TETTTSTTTLAPATTTETTTTTTTT-TTTTTeVITTPEATTTTT 287

999999999999888873112222222222222222222.....2344444431..1123333333333333333333333.233333444444444333 PP

SOG2 362 D 362

R27072766 288 E 288

3 PP

Internal pipeline statistics summary:

-------------------------------------

Query sequence(s): 1 (727 residues searched)

Target model(s): 18259 (3090017 nodes)

Passed MSV filter: 2935 (0.160743); expected 365.2 (0.02)

Passed bias filter: 592 (0.0324224); expected 365.2 (0.02)

Passed Vit filter: 263 (0.0144039); expected 18.3 (0.001)

Passed Fwd filter: 104 (0.00569582); expected 0.2 (1e-05)

Initial search space (Z): 18259 [actual number of targets]

Domain search space (domZ): 4 [number of targets reported over threshold]

# CPU time: 1.42u 0.61s 00:00:02.02 Elapsed: 00:00:03.98

# Mc/sec: 563.70

//

[ok]

# hmmscan :: search sequence(s) against a profile database

# HMMER 3.3 (Nov 2019); http://hmmer.org/

# Copyright (C) 2019 Howard Hughes Medical Institute.

# Freely distributed under the BSD open source license.

# - - - - - - - - - - - - - - - - - - - - - - - - - - - - - - - - - - - -

# query sequence file: /Users/djackson/Documents/Data/Manuscripts/2020/Cepaea_biomin_ISH/Genes/Novel_(R27073283)/Cnemoralis_Novel_R27073283_faa.txt

# target HMM database: /usr/local/bin/Pfam_latest/Pfam-A.hmm

# output directed to file: pfam_out.txt

# per-seq hits tabular output: pfam_tblout.txt

# per-dom hits tabular output: pfam_domtblout.txt

# number of worker threads: 4

# - - - - - - - - - - - - - - - - - - - - - - - - - - - - - - - - - - - -

Query: R27073283 [L=409]

Description: Novel TransAbyss assembly 2 (filtered min reads 10, dedupe95) len=1878 num_reads=1258727 avg_cov=63568.7 contig_cov=100.0% (contig_1265 from old CLC assemly 9)cds start = 227 cds stop = 1453 strand = -protein length = 409 strand = +

Scores for complete sequence (score includes all domains):

--- full sequence --- --- best 1 domain --- -#dom-

E-value score bias E-value score bias exp N Model Description

------- ------ ----- ------- ------ ----- ---- -- -------- -----------

[No hits detected that satisfy reporting thresholds]

Domain annotation for each model (and alignments):

[No targets detected that satisfy reporting thresholds]

Internal pipeline statistics summary:

-------------------------------------

Query sequence(s): 1 (409 residues searched)

Target model(s): 18259 (3090017 nodes)

Passed MSV filter: 377 (0.0206474); expected 365.2 (0.02)

Passed bias filter: 354 (0.0193877); expected 365.2 (0.02)

Passed Vit filter: 17 (0.000931048); expected 18.3 (0.001)

Passed Fwd filter: 0 (0); expected 0.2 (1e-05)

Initial search space (Z): 18259 [actual number of targets]

Domain search space (domZ): 0 [number of targets reported over threshold]

# CPU time: 0.37u 0.42s 00:00:00.79 Elapsed: 00:00:02.42

# Mc/sec: 521.50

//

[ok]

# hmmscan :: search sequence(s) against a profile database

# HMMER 3.3 (Nov 2019); http://hmmer.org/

# Copyright (C) 2019 Howard Hughes Medical Institute.

# Freely distributed under the BSD open source license.

# - - - - - - - - - - - - - - - - - - - - - - - - - - - - - - - - - - - -

# query sequence file: /Users/djackson/Documents/Data/Manuscripts/2020/Cepaea_biomin_ISH/Genes/Kelch_domain_(R27075188)/Cnemoralis_Kelch_domain_R27075188_faa.txt

# target HMM database: /usr/local/bin/Pfam_latest/Pfam-A.hmm

# output directed to file: pfam_out.txt

# per-seq hits tabular output: pfam_tblout.txt

# per-dom hits tabular output: pfam_domtblout.txt

# number of worker threads: 4

# - - - - - - - - - - - - - - - - - - - - - - - - - - - - - - - - - - - -

Query: R27075188 [L=241]

Description: Kelch_domain TransAbyss assembly 2 (filtered min reads 10, dedupe95) len=1827 num_reads=212895 avg_cov=10763.9 contig_cov=100.0% (contig_7508 from old CLC assemly 9)cds start = 390 cds stop = 1112 strand = +protein length = 241 strand = +

Scores for complete sequence (score includes all domains):

--- full sequence --- --- best 1 domain --- -#dom-

E-value score bias E-value score bias exp N Model Description

------- ------ ----- ------- ------ ----- ---- -- -------- -----------

[No hits detected that satisfy reporting thresholds]

Domain annotation for each model (and alignments):

[No targets detected that satisfy reporting thresholds]

Internal pipeline statistics summary:

-------------------------------------

Query sequence(s): 1 (241 residues searched)

Target model(s): 18259 (3090017 nodes)

Passed MSV filter: 515 (0.0282053); expected 365.2 (0.02)

Passed bias filter: 416 (0.0227833); expected 365.2 (0.02)

Passed Vit filter: 32 (0.00175256); expected 18.3 (0.001)

Passed Fwd filter: 0 (0); expected 0.2 (1e-05)

Initial search space (Z): 18259 [actual number of targets]

Domain search space (domZ): 0 [number of targets reported over threshold]

# CPU time: 0.36u 0.44s 00:00:00.80 Elapsed: 00:00:02.56

# Mc/sec: 290.82

//

[ok]

# hmmscan :: search sequence(s) against a profile database

# HMMER 3.3 (Nov 2019); http://hmmer.org/

# Copyright (C) 2019 Howard Hughes Medical Institute.

# Freely distributed under the BSD open source license.

# - - - - - - - - - - - - - - - - - - - - - - - - - - - - - - - - - - - -

# query sequence file: /Users/djackson/Documents/Data/Manuscripts/2020/Cepaea_biomin_ISH/Genes/Gly_rich2/Cnem_Gly_rich2_faa.txt

# target HMM database: /usr/local/bin/Pfam_latest/Pfam-A.hmm

# output directed to file: pfam_out.txt

# per-seq hits tabular output: pfam_tblout.txt

# per-dom hits tabular output: pfam_domtblout.txt

# number of worker threads: 4

# - - - - - - - - - - - - - - - - - - - - - - - - - - - - - - - - - - - -

Query: Cnem_Gly_rich2 [L=81]

Scores for complete sequence (score includes all domains):

--- full sequence --- --- best 1 domain --- -#dom-

E-value score bias E-value score bias exp N Model Description

------- ------ ----- ------- ------ ----- ---- -- -------- -----------

[No hits detected that satisfy reporting thresholds]

Domain annotation for each model (and alignments):

[No targets detected that satisfy reporting thresholds]

Internal pipeline statistics summary:

-------------------------------------

Query sequence(s): 1 (81 residues searched)

Target model(s): 18259 (3090017 nodes)

Passed MSV filter: 526 (0.0288077); expected 365.2 (0.02)

Passed bias filter: 267 (0.0146229); expected 365.2 (0.02)

Passed Vit filter: 30 (0.00164303); expected 18.3 (0.001)

Passed Fwd filter: 2 (0.000109535); expected 0.2 (1e-05)

Initial search space (Z): 18259 [actual number of targets]

Domain search space (domZ): 0 [number of targets reported over threshold]

# CPU time: 0.32u 0.38s 00:00:00.70 Elapsed: 00:00:01.61

# Mc/sec: 154.94

//

[ok]

# hmmscan :: search sequence(s) against a profile database

# HMMER 3.3 (Nov 2019); http://hmmer.org/

# Copyright (C) 2019 Howard Hughes Medical Institute.

# Freely distributed under the BSD open source license.

# - - - - - - - - - - - - - - - - - - - - - - - - - - - - - - - - - - - -

# query sequence file: /Users/djackson/Documents/Data/Manuscripts/2020/Cepaea_biomin_ISH/Genes/Gly_rich3/Cnem_Gly_rich3_faa.txt

# target HMM database: /usr/local/bin/Pfam_latest/Pfam-A.hmm

# output directed to file: pfam_out.txt

# per-seq hits tabular output: pfam_tblout.txt

# per-dom hits tabular output: pfam_domtblout.txt

# number of worker threads: 4

# - - - - - - - - - - - - - - - - - - - - - - - - - - - - - - - - - - - -

Query: Cnem_R37432942 [L=99]

Description: Gly_rich3 len=697 num_reads=360970 avg_cov=65037.1 contig_cov=100.0% ORF=136

Scores for complete sequence (score includes all domains):

--- full sequence --- --- best 1 domain --- -#dom-

E-value score bias E-value score bias exp N Model Description

------- ------ ----- ------- ------ ----- ---- -- -------- -----------

[No hits detected that satisfy reporting thresholds]

Domain annotation for each model (and alignments):

[No targets detected that satisfy reporting thresholds]

Internal pipeline statistics summary:

-------------------------------------

Query sequence(s): 1 (99 residues searched)

Target model(s): 18259 (3090017 nodes)

Passed MSV filter: 299 (0.0163755); expected 365.2 (0.02)

Passed bias filter: 169 (0.00925571); expected 365.2 (0.02)

Passed Vit filter: 24 (0.00131442); expected 18.3 (0.001)

Passed Fwd filter: 3 (0.000164303); expected 0.2 (1e-05)

Initial search space (Z): 18259 [actual number of targets]

Domain search space (domZ): 0 [number of targets reported over threshold]

# CPU time: 0.34u 0.37s 00:00:00.71 Elapsed: 00:00:01.05

# Mc/sec: 290.11

//

[ok]

# hmmscan :: search sequence(s) against a profile database

# HMMER 3.3 (Nov 2019); http://hmmer.org/

# Copyright (C) 2019 Howard Hughes Medical Institute.

# Freely distributed under the BSD open source license.

# - - - - - - - - - - - - - - - - - - - - - - - - - - - - - - - - - - - -

# query sequence file: /Users/djackson/Documents/Data/Manuscripts/2020/Cepaea_biomin_ISH/Genes/Peroxidase/Cnem_peroxidase_(R37577449)_faa.txt

# target HMM database: /usr/local/bin/Pfam_latest/Pfam-A.hmm

# output directed to file: pfam_out.txt

# per-seq hits tabular output: pfam_tblout.txt

# per-dom hits tabular output: pfam_domtblout.txt

# number of worker threads: 4

# - - - - - - - - - - - - - - - - - - - - - - - - - - - - - - - - - - - -

Query: Cnem_R37577449 [L=628]

Description: len=2200 num_reads=4834 avg_cov=229.2 contig_cov=99.8% ORF=628

Scores for complete sequence (score includes all domains):

--- full sequence --- --- best 1 domain --- -#dom-

E-value score bias E-value score bias exp N Model Description

------- ------ ----- ------- ------ ----- ---- -- -------- -----------

6.8e-134 447.4 0.0 7.9e-134 447.2 0.0 1.0 1 An_peroxidase Animal haem peroxidase

Domain annotation for each model (and alignments):

>> An_peroxidase Animal haem peroxidase

# score bias c-Evalue i-Evalue hmmfrom hmm to alifrom ali to envfrom env to acc

--- ------ ----- --------- --------- ------- ------- ------- ------- ------- ------- ----

1 ! 447.2 0.0 4.3e-138 7.9e-134 1 528 [] 57 599 .. 57 599 .. 0.91

Alignments for each domain:

== domain 1 score: 447.2 bits; conditional E-value: 4.3e-138

S--TTSTTSSTTSTTTT-TTEE-BBSS---.-T..CG.GTTS........SHHHHHHHHCTB-SS--.B-SCBHHHHHHHHHHHCCC--B-EEE. CS

An_peroxidase 1 yrtidGscNnlknpewGsagtpfaRllpaaYad..gvseprgss..gselPsprevsnkllaqdeslpdakltlllaqwgqfvdhDltstaest. 90

yr+idG cN ++n +Gs+g+p++R l ++Y+d g + pr s g+ lPspr vs kl+ d ++ +++ t+l++q gqf+dhD+t ++e +

Cnem_R37577449 57 YRQIDGRCNHPRN--YGSTGRPVKRYLRPHYQDkfGENLPRVYSvtGQLLPSPRMVSWKLHP-D-QTAHDNNTMLVMQMGQFIDHDITRAPELSg 147

9********9987..9*****************5555677775456****************.4.4445789********************999 PP

...EEEEE-...-CC.GCCE.SEE..TT.TCC.T.TT.EEEEEEEEE.TTSSSS.SSEEEEEESS-SSSSSHHHH-SSHHHHHHHB-SST.T-B- CS

An_peroxidase 91 ...kiecce..seen.hpeCfpIeipkdDpvfskkgercmpfvRsaadctltgs.napreqlnqvTsflDasqvYGsseeeakklRsfkg.GkLk 177

+i+cc + + p+CfpI+ip++Dpvf+ cm+f+Rs+++ +++g+ preq+n++Tsf+D+s+vYGs+ ++ ++Rs +g G

Cnem_R37577449 148 rnaSIKCCGvpPK-ErLPDCFPIDIPPGDPVFE----DCMEFFRSSPAVDNDGNiIYPREQINALTSFIDGSAVYGSDLDTYTWIRSENGtGVFL 237

9966*****7433.347***************4....5*******977777788899*****************************998546544 PP

-EEETT.CCCS-BSTTT...TT..S....GCSB-SSSCCGCCBHHHHHHHHHHHHHHHHHHHHHHHH....................-TTS-HHH CS

An_peroxidase 178 vsrskegkellpldedg..eecasa..eeaecflaGdsranenpgltalhtlflReHNriAdeLkkl....................nphwsdee 248

++ +g+e lp +++ e+c s+ +e++c laGd r+ne+pgl ++h lf +HN+i++ L + ++ +e+

Cnem_R37577449 238 NTHLVHGRERLPSHPHLgpESCVSSntAESYCQLAGDMRVNEQPGLGSIHLLFHLHHNHIVRLLVAGilkkrgqpssperiakfiqeSSSALKEQ 332

433445899***9664467889888777778*********************************987899***************9999999*** PP

HHHHHHHHHHHHHHHHHHCTHHHHHCSSHHHHHH-TT-H.HHST-STTS----BHHHHHTHCGGHHHHS-SEEEEE--GCCS-EEEEEEGGGCCC CS

An_peroxidase 249 lfqeARkiviAliqkItynewlpalLgkenanklglllkgeyegydesvdpsisneFataafrfghslipdeierlneekevseeqelrlkdsff 343

+fqe Rk+ +A+iqk ty +wlp +Lg+ ++k++l + + y++++dp+++n+F +aa+rfgh+lip+ +++ + + +++lkd+f+

Cnem_R37577449 333 IFQEVRKMLGAIIQKLTYCDWLPMILGPYLIDKFQLGCT-RRSRYNSDLDPRVANSFLSAALRFGHTLIPNVYNFGD------K--RIHLKDTFN 418

***********************************9999.5689***************************998888......3..489****** PP

HHHHHTTH-CHHHHHHHHCSB-B-S.TT-B-HHHHTBBCSTT..TTT-B-HHHHHHHHHHHCT--BHHHHHHHTT----SSCCCC--..SHHHHH CS

An_peroxidase 344 npaelleggldellrgllsqpaeav.dnniteelrnklfetk..eesglDlaalniqRgRDhGlpsYnefRrlcglkkaksfedlteeideeele 435

p+ + +d++++ l+++ +e+ d+++++ ++++lfe++ ++++lDl+a+niqRgRDhG+p Y +R++++l+++ s +++ e +

Cnem_R37577449 419 IPDASIR-YYDNIIQCLIKEGSEEAyDRYVSSAVSEHLFESTrgHKHALDLIAVNIQRGRDHGIPAYHYWRQYYRLRRIISLDEFGE-----AGI 507

***6666.69************9999**************99655999*************************************85.....556 PP

HHHHHTTSCCCSBHHHHHHHS-B-TTBSSCHHHHHHHHHHHHHHHHT-CCGTTTHCHHHHHTHHHHCCHH.CSHHHHHHHHSTT...HHC.TT CS

An_peroxidase 436 klkevYgsvediDllvGglaEkplpgglvgptfaciiadqfrrlrdgDRFfyenkkqpglfteeqleeirktslarvicdntdeltevqpnvf 528

++k++Y+++ d+Dl+ Ggl+E +pgg+vg+tf +i+a+qf+ l+ gD +f+ +++ p+ f +q++ i ++++++ic+n+ +++ qp++f

Cnem_R37577449 508 AMKKAYRDIRDVDLFPGGLLEPSMPGGVVGETFGHILANQFADLKFGDTYFFLHQQAPQGFRAAQIKAILSVTMSSIICANSAVTQ-AQPDPF 599

8***********************************************************************************88.9*9998 PP

Internal pipeline statistics summary:

-------------------------------------

Query sequence(s): 1 (628 residues searched)

Target model(s): 18259 (3090017 nodes)

Passed MSV filter: 428 (0.0234405); expected 365.2 (0.02)

Passed bias filter: 412 (0.0225642); expected 365.2 (0.02)

Passed Vit filter: 21 (0.00115012); expected 18.3 (0.001)

Passed Fwd filter: 1 (5.47675e-05); expected 0.2 (1e-05)

Initial search space (Z): 18259 [actual number of targets]

Domain search space (domZ): 1 [number of targets reported over threshold]

# CPU time: 0.41u 0.44s 00:00:00.85 Elapsed: 00:00:02.75

# Mc/sec: 703.74

//

[ok]
