# Supplementary material for: Mantle Modularity Underlies the Plasticity of the Molluscan Shell: Supporting Data From Cepaea nemoralis
Source: Front Genet. 2021 Feb 5;12:622400. doi: 10.3389/fgene.2021.622400 (PMC7894901; doi:10.3389/fgene.2021.622400)
Supplement: Supplementary file 11 [file Data_Sheet_11.docx]

>Cnem_R37577449 len=2200 num_reads=4834 avg_cov=229.2 contig_cov=99.8% ORF=628protein length = 628 strand = +

MDSAVFLLAVVASLGQCVYPDIYARRGDLGDSTGSLDFVSEADLEHCSRLTYDQLRYRQIDGRCNHPRNYGSTGRPVKRYLRPHYQDKFGENLPRVYSVTGQLLPSPRMVSWKLHPDQTAHDNNTMLVMQMGQFIDHDITRAPELSGRNASIKCCGVPPKERLPDCFPIDIPPGDPVFEDCMEFFRSSPAVDNDGNIIYPREQINALTSFIDGSAVYGSDLDTYTWIRSENGTGVFLNTHLVHGRERLPSHPHLGPESCVSSNTAESYCQLAGDMRVNEQPGLGSIHLLFHLHHNHIVRLLVAGILKKRGQPSSPERIAKFIQESSSALKEQIFQEVRKMLGAIIQKLTYCDWLPMILGPYLIDKFQLGCTRRSRYNSDLDPRVANSFLSAALRFGHTLIPNVYNFGDKRIHLKDTFNIPDASIRYYDNIIQCLIKEGSEEAYDRYVSSAVSEHLFESTRGHKHALDLIAVNIQRGRDHGIPAYHYWRQYYRLRRIISLDEFGEAGIAMKKAYRDIRDVDLFPGGLLEPSMPGGVVGETFGHILANQFADLKFGDTYFFLHQQAPQGFRAAQIKAILSVTMSSIICANSAVTQAQPDPFYMASQLNLPRPCSDYSEMDVEPWLIHFSD

>Cnem_R37442076 len=2191 num_reads=41397 avg_cov=2301.1 contig_cov=100.0% ORF=624protein length = 624 strand = -

MAAYNCVMLLMSVILMAVCSGQDTCKNGTNNTSQFYEMADNIEAASTGTVGEMSRRFKEERSEFCQDAGLRMYRTFNGTCNHPKNLGASFTIQDRFLPPAYSDGINSPRMFGVDGLPLPSARTVSSKIHVSKDRKASKFSIALMAWGQWVDHDIISTAVSTRPGKRIQCCGPNGTCPPFMTNPNCFPIEIPSDDRVFEGTCLEFVRSLAATDDNGYQKFPRAQINTVTSFLDASTVYGSTNELAESIRDPDGYLLRMKTDGFPPEAQNASCVKREERQDYCFHAGDFRVNQHPFIGAIHTLFLREHNRICRRFRLLYPDLSDEKIFQMGRKLVGAMNQHFTYNVFLPLIMGDEAHVWKLISDSERSHYNPGIDPRIAVAFGSAAFRFGHSTIPSYLPVGNAQVQLRYLFNTNGYVMDDFEGILAGIAGITNSPDHSIQSVDKYVEDDVSKFLFFNNKTKRGLDLISINLQRARDHGVQPYYKWRAFCGLRPLTGFDDVEALGPDVGELAKVYRDVRDIDLFTGMLHEPVKTGIVGPTISCIVGMQFKRLKHGDRFFFNNNEEYTRFTDEQLAALLNVTLGDIMCQNSYLEAVPENVFLTVSESNPLIPCTRLRQMGLDLTLFDY

>Cnem_R37435138 len=2114 num_reads=123800 avg_cov=7535.7 contig_cov=100.0% ORF=682protein length = 662 strand = -

MQQFGPPILRSQQNASSNNVQQTGFIAVPIQHPPNIVGLEPIDNVGKVTEEALTVAEPPPEPECCQNEPDTGVFYSVAEKHGAAVTGSVAGKGHVFSQERRDWCPDFGKSKYRTFDGTCNHPRNLGSSFTPVSRLLPPVYDDGLNSPRTKGEDGSPLPSPRVVSSSIHPSESNGEDFTHMLMQWGQWVDHDMTAIITASNRSNPTRCCGPDGALPGVNTNKDCFPIELPETEKNFRGRCMEFVRSLAATNATGQPLQPREQVNSLNAFIDGSQIYGATSERAALLREKDDYMLRTKGDFPPESPDGGCILRPGSKDYCFITGDLRTHENPALTALHVIWMREHNRMARELHALKPQADKEEIYQIIRKIVGALQQIFTLNHYLPIVLGKDAYRLRLVSKEGRTVYNRDTDPRIFNAFTAAAFRFGHSSIPSAYAVGDKLVPMREMFNRPALVLDDMDSLLRGLVGMTKYKEKASQKVDRNFVEEITQHLFEPNSTLPGRGLDLVSLNIQRARDHGLPPYTKWRTFCGLRPVYGFNDTEALGPYSSQLGKVYRSVNDIDLFTAIVHEPIDTGLLGPTGRCLVGIQFARAKFGDRFIFDTDDYRIAFTEEQLAAMRKMTLSHVICQNTKIDSLPVDVFRFSDPTNPVVTCAALRAESLDLSLFN

>Cnem_R37509337 len=2030 num_reads=6433 avg_cov=407.5 contig_cov=100.0% ORF=606protein length = 606 strand = +

MASLKCLTILLLTTLLNMSVGTDNQYCRNKDKNRDCASSKDTCNKCVSPSTIKYRTFDGTCNHPRNLGAESDLFDNLLPQEYDDGSNTPRQRGVDSQPLTSARNLSLNIHKQKDVSAAFPILMMQWGQWLAHDFTATKTAIGTISCCGPNGGCPPFVTDRDCFPIQVPPSDPTFAGICLNFVRSAAANGSDCLPLRPRRQINTVTSFLDCSQIYGSTNDVAASLREQGGFLLRTKFGKFPPEANSSVCTKRNNTNDYCFLAGDFRANQHTYLSAFHTLWLREHNYIATELHKLKPDMSAEDKFQTTRKIIIGMHQVFTYNTFLPIMLGEDAKRWQLTSTRGRSFYNPSLSPRIASAFSVGAFRFGHSTTPSLFPIGNRSVPLRLLFNRPGYVLDEFDGIMAGMVGLTYAKDGRVQTSDKFITDELSRFLFFNNVTGRGFDLNSVDIQRGRDHGIPPYVKWRSFCGLRPLTGFNDTEALGPHASELANYYRSIDDMDLFTGMLHEPITSGLVGPTIACLMRIQFHNTKYCDRFFFDNTEPNSGFTNAQLASIRAVTLSQIICRHTSLTSIQRNVFKLASADNPLIPCSKLRSQGLKLSLFVETSKEY

>Cnem_R37580477 len=2483 num_reads=7998 avg_cov=331.8 contig_cov=100.0% ORF=603protein length = 603 strand = +

MMTRHALSFLILLTTMGSFCPTIYGWGRRCPADITELALTSSLHTDCNNIGSCTRCKQTTNTYRTNDGTCNHKDNKGSQFAEIDRLLPPTYADGSSVPRQHGVGGQDLPSARNLTVKLLTSKEAFSGNPIILFQWGQWTSHDILSTSVNTGGIQCCGQQGKRLFVPDCFPIDVPPNDPTLSGTCMNFVRTRAAKCCDNSTVKPRIQTNDVTTFLDSSNVYGSDDEVAQAVREPGGYLLRTKFGLFPPVSNGTCNIPDGSREYCFLTGDHRPNQHTYLSAIHTFWLREHNRIATELKKLKPCLHNETIFQTAKQIVNAVNQAVTYNEYLPTLLGDDATRWKLTSTAGRSTYNPQVNPSLVTEFTTAAFRLGHSQAPSKFPIGNELVHLRYLYNRPGLVLDHFEDIIAAMAGHSSVANQGSQRVDKHFTEELTQFLLYNPKTGRGGDLLSTNIQRGRDHGLPTYVQVRDFCCLPPITGLNDTALGPEGYKLGQFYKSVKDIDLYTGMLHELADIGLAGPTIRCLVGLQFYRLKYGDKYFFDNDRPSGFSDEQLESIRSVTLGHVMCHHTNLESIQRNVFEFTSLTNKEETCSKLKREGVNLKLFV

>Cnem_R38134193 len=1962 num_reads=664 avg_cov=35.0 contig_cov=75.3% ORF=620protein length = 614 strand = -

MTTKTKMLRRLSVLIMLVIVQHSSSANASLTKERKCPYDITKLAFTSALQAQCKQTKQKTCGCSYDKYRSIDGSCNHVLTDSGAAGSVIDKLLPPTYDDVWHAPRKVGVGGEPLPSPRQISVEILTDKHVFSIYTLLLFLFGQWTAHDMISTTTSSGRNCCGANGTNPTIVQPTNCFPYAVASNDPTFPGTCVSFVRSQAANCTNWCQIGFRVNTNEVTAFIDSSQIYGSSHEVEQSVREQGGYLLRTNRGNFLPESNLTCNKPNVTRDYCVLAGDNRCNQHSILTFFHTFSMRAHNRMATQLHELNPKLSNETIYQTVKSIINAIYQSILYNEYLPIVLGYDSVRRWNLLSTSGKSVYNPDLRPRMIQEAVTAAMRFGHSQIPGEIPVGNEMIPLRYLFNNPGIVLDHFEDLMDALIGRSQLPNQGSRKVDQYFTEDVSRYLFFNNKTGRGSDLPATNIQRGRDQGLATYSQVREICCLPPMTGFNDTKALGPEGYKLEKHYKSVDDIDLYVGGLLEVPESGLLGPTFNCLVGLQYYTLKHSDRFFFDNDQRHFTDEQVKEIRKVKLGEMICHQTNLTSVQKNVFEKPSSTNEDVSCSWLKKNGLDLRLFAGL

>Cnem_R37516884 len=7246 num_reads=26703 avg_cov=399.0 contig_cov=100.0% ORF=2234protein length = 2234 strand = +

MEISIFVSAFLVFVMCCLDTARASACPERCLCFRTHVRCMFMNLESIPNVLPDTTTLDLRFNKISKIPRGSFPHLPHLHTLFLNNNEIQELEDGYFDGLPELRKLYLYKNKIQKIHPNAFRSIPHLEELYLHHNELSKFPEHLFDGNSNLKKLRLDSNSLVCDCDMMWLVDMLKEKGPETQAAVYCRYPSKFQGRSLMSMAKEDFHCAEPKITMRPKDVDVSFGNTVYFTCRAEGAPNPEIMWFHNDNMINTNEEERYSILDDGTLMIEAAQDSDKGVYECMARNAVGMAKANKVELRYMGDQEIPKFMETPSDITVIEGDDAQLVCHVMGNPRPDITWTLNSDPVSGNIRAQVLESGSLVITNIRLSDAGTYECSASNSVKTISSSARVRVLVKPVIISAPADVSVVQGTQVNFTCEARGDSQPVLTWTKDGEFLRNNGRYEVLKNGQMLKIHNAAASDQGKFTCKAENAAGSTTASGTLRIVENAAPSFSQSSDLISAAAGSDVTLHCIADARPDALYDWMRDGRVLQNRNRISVQAGELKITNVHIHDAGRYDCVAENSLGKATKAIFLQVQGANIGRIGDQFVSNAIPQATRQVNTAVNTTLSHLFDPNREHTVQDLLAAFRYPTPEALDLARAEEIFEQTLEIIQRHVSEGHSYNLTGFETSYHELISPAHIHMIANMSGCLRRTHAADCSDTCFHRRYRTMDGTCNNLQNPTWGAANTAFNRLLPAIYENGFNAPVGWNRHKLYKGTHLPSARLVSSLVISTEHITSDETFTHMLMQWGQFIDHDMDLSPQAISFARFSDGRRCNETCENTNPCFPIAVPTSDPRIRNHECLGFTRSSATCNTDSTSLFFNTVSPRQQLNSLTSFIDASNVYGSTDRMASTLRNLASNRGLLREGPTTATARRLLPFNEDTLEMPDCQIQPSKRHIPCFIAGDVRVNEQLALIAMHTLWMRQHNQMAVELNRINPHWDGNKIYHEARKIMGALFQHITYKHWLPKVLGPKGMAMIGPYEGYKPDVNPSIVNEFAVAAMRFGHTLIQPMIFRLNETFQEIPEGHLSLHQVFFAPHRIIEEGGLDPLIRGLFAKAAKKKMPGELLNSELTEKLFQLANAIGQDLASLNIQRGRDHGTQFYNDYRVLCGLPRARTFDDLRSEIPHKDTRDKLQALYSHPDNIDLFVGGVSEYVVEGAKVGPTFLCILSDQFKRLRDGDRFWYENKKNNIYTQEEVDEIEATMFYDVINNVTLALRDTNLINVTREEIYFCSSSSDPKGCECNDPTVDDNDVIEVCVPLQHYDYFTGSEVSFVITIVVIVLSLPVTIAIMMCMSWQRSHSLNASGEGSKAKFEVGPNHFYATEWVGRSTHGFLTSRDVKINLDDTRLKILVSNMQKQVVRMIDLRRRLTNEQEKPRASVTRSNDKGHKLMMVGVPGEIDLVLHFASQNDRDSAFRQLQGFFNKHNWDFHEGPVMAENVMWREAVTVDQRKELLAKFFRSALAEISGEIPDTSNQKHMEEILATQLTRTEFADALGLQPQSLFVRNMFLLVDSSGDGFISFDEFKAYFGILSSGNPEQKAEMFFKMFDTSRTGKITKEDYKKMIIVLFEMNETDRSKEFNINEIVDKVFKQVGKDQQGFLTLQDFKSIVLSDTADVWKFAVLNLEVAGETATLGYKKSVKDRAKSFIEGYKTNLRPTSMINLKNDVRLPSTLESAPKSKFDEFVQKWSRYVNNHRQQIFWVTLYTLVTIGIFVERAYYYSFEREHAGLRRLAGYGVSVTRGAASAQMFTYASLLVTMCRNTLTFFRETFLHRFIPFDNFHDMHIYVAFLAVLFSVIHVIGHIINFFHICTQSSEDLNCYFREYFRPTDVLASFHYWTYQTITGLTGVALILVLVVIYVFAMPYARRNVFKYFRTTHNLYIALYILMFLHGHHRLVQPPLWPWYFIGPVVLFVLDKLVSVSRNKVLLPVHKARLLPSGVIQLVFKRPLTFDYQSGQWVRIACPKLGKGEYHPFTLTSAPHEEHLSLHIRAVGPWTSNLRKLFDTNKLTTTKIPKYQIYLDGPFGESHQDWYRYSVSVLIGGGIGVTPFASILKDIANKTVQVKRLPVQKVYFIWVTRTQQSFEWMTDIIRETEAADVHDFLDVQICITQLKEQFDFRTTMLYICERHFQKFAGASMFTGLRAKTHFGRPKFNPFFQALKVVHKNVGQIGVFSCGPPAMTNSVQKSCTEQNSFTGPSLIHHYENF

>Cnem_R38332823 len=5597 num_reads=1230 avg_cov=26.9 contig_cov=100.0% ORF=1519protein length = 1519 strand = +

MKVCHFVIAAAICSACICGNSVKDIPVQRFDGWYNNPLNPSWGGVGRPLERNITPTYEDETYRPSGWDRPNPRYISNELFGELRGRSGIPNKRNLTALFAFFGQIIQHEILDTDDVTCPVEILDVTVPRGDPHFDPNSEGDKTMPYERSAYEKTSGQSPNNPRRQINWQSSFIDGSFLYGKSLVRTEFLRDPHSGKLACEDKWGKFPLRNDVGLPYNSFTYSFNTLENLWRLGDPDVFENPAILALNMIFYRYHNAKAEEFEQLHNYMSNEEVFDKTRRWVIGCIQKIIVYDWLPMLIEKELPAYSGYKPWVKSDITDIFDAGAINYIMTLIPSAIHQIDSSCQERATDRLCNVFWKAMDVVYKHESGILEILRGLSAQYAENSDTFVVEDYRSKFYGPLYHSKHDAVLLTIMKGRDYGLPDYNTVRVTMGLKEMTNFEEVNPWLNQTNPELFAAFKSVHKGNLSTIDMFVGGMMESTPDGPGELFTHILYDQFIRLRDGDRFWFENSANGLFSSEEVQRLRNTTLADIFRTAGIDMQDIQDNVFTLTDKSPCGHRMIFNDSTLEPCEKHHGYDYFHGSEIPYIIIWTCLGLLPLLCILAAYILAKCKKWRHERRIRVLKAEQEKRRNFRRSLSCSVSVHDGVEWRGPHFSGRPIELHLTTKGCLEIFSATGTHLRAIKISSLSNIGVKISSNKKRNIMALILPREYDLVVEFRTEVLRNEFVSMLCQFVQNHSVDITVIELKLRDLYNAATTKEKRNKMLEKFFKTVFSEAFQMDYDPSLDSGQQDLKKHSQEILEIELSKEEFAEAMAVKPNSDFVEHFFSLIDSDRNGYISFREFLNAVVLFSKGSIQDKLQTMFYMYDIDGTGYMNLMQIGQMFRSLLELAQSDLTHGDVDLLVHSLCKQSGVGPKEQYSFDDFCQLLSPQMDKLWNASFDWRGIPKVIPSKVKSPKKTGSVNSETDMRRRGSSASLSGIDGAKSSVVKKFEAVREQYTPVKAKVKLVKHFIENYRHHIFFVVLFYGICLGLFAERFYYYTVEQESAGFRKLMSYGISMTRGAAASMSFTFSLMLVTMCRNTITYLRSTPLNLFIPFDSHVSFHKVVAWTSLFFSAIHIIGYSFNFYHLATQPTRFLCIFTSLIFRTEHSMSFQEWVFGTMTGFTGVLLVLILCILYVFATQTARRHIFSMFWMTHKLFIVLYILVIIHGASVVVQKPMFFAYLTGPAIWFMVDKLISLSRKKTELCIIKAQNLPSDVTMIEFKRPPRFEYKSGQWVRIACLAQGKDEYHPFTLTSAPHEDTLKLHIRALGPWTWNLRHIVDNVNIKNTKDYPKLYLDGPYGAGQQDWYQYDVSVLVGAGIGVTPYASILKDFVHMASINMRYKVKCQKLYFIWITGSQRHFEWLIDILREVEEVDTQGMVSIDIFITQFFQNFDLRTALLYIFEEHFQKLNGGKSVFTGLKATTHFGRPQLNKIMGAVHKAHPQARKVGVFSCGPPGVTKGVERACVDASKATKALFEHHFENF

>Cnem_S37647837 len=897 num_reads=264 avg_cov=28.9 contig_cov=99.3% ORF=281protein length = 281 strand = -

MTTKTKMLRRLSVLIMLVIVQHSSSANASLTKERKCPYDITKLAFTSALQAQCKQTKQKTCGCSYDKYRSIDGSCNHVLTDSGAAGSVIDKLLPPTYDDVWHAPRKVGVGGEPLPSPRQISVEILTDKHVFSIYTLLLFLFGQWTAHDMISTTTSSGRNCCGANGTNPTIVQPTNCFPYAVASNDPTFPGTCVSFVRSQAANCTNWCQIGFRVNTNEVTAFIDSSQIYGSSHEVEQSVREQGGYLLRTNRGNFLPESNLTCNKPNVTRDYCVLAGDNRCNQ

>Cnem_R39974694 len=666 num_reads=26 avg_cov=4.1 contig_cov=99.1% ORF=199protein length = 198 strand = -

MAGLANVRNTNMQNADRNFVEDIARFLFKNQVTGRGLDLVTLNIQRGRDHGIQPYVRWRSFCGLRPLTGFNDTEALGPFASLFGSLYRSVDDIDLYTGMLHEPVETGLVGPTIRCILRIQFHRLKYGDRFFFNNDEIYSGFTKEQLESLLRVTLSKVVCQNTNIPNMPRDAFKFRSRKNSLLSCSSLKAQDLDLAVFA

>Cnem_R38307666 len=5275 num_reads=1758 avg_cov=36.6 contig_cov=100.0% ORF=787protein length = 787 strand = +

MMIASSYSLYLCLTICLTFYAKESHGDNTEYAPVDGFRNNLGQPTLGIAGSNFARVSPAAYSDGAYGMAGLDRPHPMEISLKVHAGRSGQASTFSRNAMQAYFAQLVLDEVALVTPGCPPEYENMPVPDQHPLGNLTKRPQVYQRSLYDQSTGYVSGSPRQQVNMASSFLDGGIIYGTSKTWTSLVRSYSGGKLLADTDNITSSFPTYNDVGLPLLNAPIPREHVMKPADRLFRLGNAKGHENPFLLALQVIWFRWHNTIAGDIAESSAGNLTDEQIFQTAKKMVVAHFQKILVTDWLPAFLYGNQMNATMEVLPAYTKYDSRQHAGVTQEFLAAMDFRHTFTPAAVWNLKAMCKLGNTTGRDNMGRSRQVKSLRLCNNFWDPQTAVKDDLEMILRGMLYTLGKREDGRFVSDFTEFFYGPFEYSRTDHVAAQIQRYRDHGLSGLNTIRRSYGLATLSNWTSLGGLNGTLHRLYGGTKPDNVDILTGGLLDQSGQMAGLSEIFRKILIDQFTILRDGDRFWYQRSGLFTANEIKEIESVTFKKILLATTRLSENEIPDNVFLCRDTNNCKCLTPPDVDNIASTDIDVCSNLATHDYIEGSKLSFALSFLALALVVPASYLVLFCLVKKRKTVIQEVQKRKSTFVSKRNQNKFMAREFLGKKSGFRDIKGEFKADEGMIIICDGGGKILRYINIKGEVENAPPINIHMSSDKDLSLLAIKVPEDIDLILDFVDQGARQLFIQSLERYLSAVGLNFETERMSESHLYSRCTDKDQRQNLLDKFFRAVCI

>Cnem_J37533192 len=971 num_reads=115 avg_cov=12.0 contig_cov=99.1% ORF=154protein length = 154 strand = -

MTTKTKMLRRLSVLIMLVIVQHSSSANASLTKERKCPYDITKLAFTSALQAQCKQTKQKTCGCSYDKYRSIDGSCNHVLTDSGAAGSVIDKLLPPTYDDVWHAPRKVGVGGEPLPSPRQISVEILTDKHVFSIYTLLLFLFGQWTAHDMISTNE

>Cnem_R37651366 len=1821 num_reads=4517 avg_cov=281.4 contig_cov=100.0% ORF=553protein length = 553 strand = -

MLVRLKRTMWGFVFLVSCTVILSGVWSKDVERHPNDGWYNNLIHPEWGAIDSHLLRRSLTNYSDGVYLPSGGNRPNPLTISEITFNGSNSLGSVRKRNALLVFFGQQLVEEILDAQRPACPVEFFNIPVPVDHKYNPKRKENLELPFLRARFDQSTGYSANNPRQQINEITPYMDGSLVYSASKATEDTLREFSGGRLAASSKNIYESFPIINDIKLPLANPPPPRDHVLRPVSRFRRYGNPRTHENPFMLTLATIWFRYHNHIAEKLANKTPGMDDEQLFFAAKKRVIAQYQKIAMYEWLPHWLQITENFTTFDIRGQYPYEGGNMNKYTGYDPNVHPGISTEFQTAAMRFGHTLVTSGVVTKTVENGTCLVTYRNVKAKFTNSSDSNGENDVKVKGIRLCNTFWVPQETLEGNPGIDAIIRGLVTTRTAMEDNIIVTDIRENVFGPLEWSRRDIGALNIQRARDEGLPSYNDIREAYGLPRITNWADINRAYTNITDKLKILYGNTEAPDNLDLFPGGLLETTFDGPGPLFRAILLDQFLRIRHGDRFWYE

>Lsta_jg60788.t1_sfc5 gene=jg60788 CDS=1-1956protein length = 651 strand = +

MLRVEDNMKSAVLSLLGICLYLTVSIHSDEKFCASHADLYYANIAGKIQGGYDMLSLGDQSACANRATRDLRYREIDGRCNHPKDYGSTMKPLKRYLKAHYQDAEGKDAPRIYSVMGDNYLPSPRLISFKLFPDIPKVANTSRWTMQFGQLIDHDITSAPVPTGPNGTIKCCGVKDMPKECFPISVPKEDTRFDTCMEFVRSQAAADEKGNQLYPREQLNAITSFVDGSAFYGSDLNATKRLRTEDGKGALLKTVVVNGFERLPNDTSARPGCLKSQRPESYCMVTGDRRANLHPGLSSAHLLFHLYHNFVVRSLAAAILNKRGQPYAPNNVEYFITNAPEYLKEKLFQEGRKVMGGIFQNIVFCDYLPIILGPGLIEKFKLGCTRRSKYNPYVDPRIANSFTAAAFRFGHTLVPNSFTINGKQEMLKDSFLIPDGTIYGFESLVQNFLTDGKSEAFDWFFSKSISEHLFETRTGPKGAIDLLSVNIQRARDHGIPAYYYWRRYYGLRRFTSFDDWGYETGKKFKQLYNHVNDVDLFVGGVFEPPVAGGVVGETFGHIIANQFADLKYGDAYYFLSKEYPQGFTDGKGFTDEEIAAIQEVTFNSIVCYSAKLQYVQDNTWFVPSESNPVRPCSVHPQFNAEVFAKNFDQPY

>Lsta_jg60788.t2_sfc5 gene=jg60788 CDS=1-2013protein length = 670 strand = +

MLRVEDNMKSAVLSLLGICLYLTVSIHSDEKFCASHADLYYANIAGKIQGGYDMLSLGDQSACANRATRDLRYREIDGRCNHPKDYGSTMKPLKRYLKAHYQDAEGKDAPRIYSVMGDNYLPSPRLISFKLFPDIPKVANTSRWTMQFGQLIDHDITSAPVPTGKLTSLITSAAVPTAPVPTGPNGTIKCCGVKDMPKECFPISVPKEDTRFDTCMEFVRSQAAADEKGNQLYPREQLNAITSFVDGSAFYGSDLNATKRLRTEDGKGALLKTVVVNGFERLPNDTSARPGCLKSQRPESYCMVTGDRRANLHPGLSSAHLLFHLYHNFVVRSLAAAILNKRGQPYAPNNVEYFITNAPEYLKEKLFQEGRKVMGGIFQNIVFCDYLPIILGPGLIEKFKLGCTRRSKYNPYVDPRIANSFTAAAFRFGHTLVPNSFTINGKQEMLKDSFLIPDGTIYGFESLVQNFLTDGKSEAFDWFFSKSISEHLFETRTGPKGAIDLLSVNIQRARDHGIPAYYYWRRYYGLRRFTSFDDWGYETGKKFKQLYNHVNDVDLFVGGVFEPPVAGGVVGETFGHIIANQFADLKYGDAYYFLSKEYPQGFTDGKGFTDEEIAAIQEVTFNSIVCYSAKLQYVQDNTWFVPSESNPVRPCSVHPQFNAEVFAKNFDQPY

>Lsta_jg60788.t3_sfc5 gene=jg60788 CDS=1-2082protein length = 693 strand = +

MLRVEDNMKSAVLSLLGICLYLTVSIHSDEKFCASHADLYYANIAGKIQGGYDMLSLGDQSACANRATRDLRYREIDGRCNHPKDYGSTMKPLKRYLKAHYQDAEGKDAPRIYSVMGDNYLPSPRLISFKLFPDIPKVANTSRWTMQFGQLIDHDITSAPVPTGKLTSLITSAAVPTGNLTSLITSAPIPTGKLTSLITSAPVPTGPNGTIKCCGVKDMPKECFPISVPKEDTRFDTCMEFVRSQAAADEKGNQLYPREQLNAITSFVDGSAFYGSDLNATKRLRTEDGKGALLKTVVVNGFERLPNDTSARPGCLKSQRPESYCMVTGDRRANLHPGLSSAHLLFHLYHNFVVRSLAAAILNKRGQPYAPNNVEYFITNAPEYLKEKLFQEGRKVMGGIFQNIVFCDYLPIILGPGLIEKFKLGCTRRSKYNPYVDPRIANSFTAAAFRFGHTLVPNSFTINGKQEMLKDSFLIPDGTIYGFESLVQNFLTDGKSEAFDWFFSKSISEHLFETRTGPKGAIDLLSVNIQRARDHGIPAYYYWRRYYGLRRFTSFDDWGYETGKKFKQLYNHVNDVDLFVGGVFEPPVAGGVVGETFGHIIANQFADLKYGDAYYFLSKEYPQGFTDGKGFTDEEIAAIQEVTFNSIVCYSAKLQYVQDNTWFVPSESNPVRPCSVHPQFNAEVFAKNFDQPY

>Lsta_jg17016.t1 gene=jg17016 CDS=1-2808protein length = 935 strand = +

MCLESICLEPVCLKSYSFLVNLLGVNLLGINLLGVNLLGVNLFGVSLLEVIQFLVNLLGVNLLGINLLGVKLFGEMTIIQEIHIPLAAFPLSLIMSRDCCPPPIPTTPDTIIFSSGGGRISNYFKNLSGPRDSGANHCLAMICARNWTLVCMCLMALAIQLDALTSHLSGSDNNGNGRGQGLVNDFRTFEDERDAGTTFSDARSLIDAQDAGSAPIGVIELNGKENGKGNGFGKVNSGNNQGRGNSQGKGNNQGRGDQQDSASNRGNGNSQGKGTNQGKKSSQDSGKGKVKEHVKVDARNEQGTNHNNGRFLANIVHIWWSLGTTTSVAGSINGVCRRQINGFYNLAQRSSAITTGSVRSVGLRFAQDFRDLCTDVALRKFRTTDGNCNHPKNWGTSFKPVTRLLPADYSDGSGSPRKTSVSGSPLPSPRAVSRYVHPARTDLLARTIMIMQWGQWLDHDVTGFPVASELDRSIKCCGPNGTLPALNTDPNCFPITLPKDEDNFLGKCMEFVRSIPATDNNGCALQPREQVNSITSFIDASQVYGSTEETAAKVRNRKGFLLKTKNGDFLPENVKGGCIVRPGSSDYCFLAGDFRVNEHPALAAMHTIWLRLHNKIASNLKTLRPADSDDDVFQLTRKIVGALQQVITYNEWLPIILGKQASELKLVSRAGRTRRQAGVDPRILNEFSTAAMRFGHSLIPDVFPIGDRRVPLRQLFSRPAEVIDNFDKVVAGIAGVGTPGNRNAQKIDRNFVTEITNHLFEPQSGPAGRGLDLVALNIQRARDHGIPPYKAYRAFCGLRPLTGFNDVEALGPNVAQLARAYSSVDDIDLFTGLVHEPPVHGSNALVGPTLSCILGTQFYNLKFGDRFFFDTDEQVIAFTNDQLASLRNTTLAKVICATTKIPELPNNVFSFPTKGNPLVPCPELEASGLHLSLFA

>Lsta_jg27188.t1 gene=jg27188 CDS=1-2013protein length = 670 strand = +

MFYYCVLALTLFALILGQDYDISLVAEEEEVYDINVLTQTDNADVVESPPSKGNGRFSSLEAFQQAAPRPAEVCLPSSGEFYNMPERLSAMSTGSISSSAFRLSQDYRELCLDFSSKKFRTIDGSCNNLRNWGTSFKPVSRILPARYQDGVGSPRTKASDGTPLPGARFVSLAVHPAVTDLTSRPIIAMQWGQWIDHDITGFPAATSPNGPLVCCGPNNTSPPANTDPNCFPILLPASEEDFVGTCMSFVRSNAATDANGCQLKPRRQVNSVTPFLDASQVYGSTDAVAASVRDAGDFLLKTKNEKFLPENVNSSCIRRPGTNDYCFLAGDFRVNQHPYLQSLHTVFLRDHNRIARKLRALRPNDSNENIFQLSRKIIGALQQMITYNEWLPVILGQTAKKLGLVSKTGRTKFQSRVDPRILVEFSSAAMRFGHSLIPAEFPIGDRRVPLRQLFNRPGDVLDNLDDVTAGMVGVGTPGKRNTQKVDRNFVVEITKHLFEPPTAPKRGLDLVSFNIQRGRDHGIAPYTTYRALCGLRPVRGFKDVKALGPNVADLAKIYKSVNDIDLFTGLVHEPVLPGSNGLVGPTLACILGTQFYNLKFGDRFFFDTDDKSVGFTDKQLQSLRKVTLAKVICANTDITALPKDVFNFPSRSNPLIPCDRHEGLDLSLFA

>Lsta_jg45104.t1 gene=jg45104 CDS=1-1923protein length = 640 strand = +

MSRAMSTGSVTDAGTVLRQDFRDINDVNYGLVLTVDRFIQGFCEVNKNGFFNLPEMSRAMSTGSVTDAGTVLRQDFRDLCTDVTSKKYRTIDGNCNHPKNWGTSFKPLARMLPAEYDDGVSSPRVRGSLGTPLPSPRAISRFVHPSTPDLISRTIMIMQWGQWLDHDLSVSPVASEVNHIIQCCGPNGTLPRNPTDPNCFPIILPSDEENFAGQCMEFVRSIPAFDANNCMLRPREQINVLNSFVDASQVYGSTDDLAARLRVEGDFLMKTKDEIFLPEDVASACIKRPGTKDYCFLAGDIRVNEHASLGAMHTIWLRAHNKIAKQLRQLRPKDSNEEIFQLTRKIIGALQQVITYNEWLPIILGKHATTQKLPSKTGRTQRLLSADPRILNEFSTAALRFGHSFIPDVFPIGDRRVPLRQLFNRPAEVLDNLDDLVAGVTGVSGSKSAQKIDRNFVAEITNHLFEPPTGPRGHGLDLIALNIQRGRDHGIPPYTAYRAACGLRPLTGFDDVDGLGPNVAQLARVYRSVDDVDLFTGLVHEPPAPGSNALVGPTLACILGTQFYNLKFGDRFFFDNDDKLIAFTDNQIKSLRNTTLAKVICSNTNIEALPNNVFSFSAKSNPLIPCKQLGEESLSLSLFS

>Lsta_jg27183.t2 gene=jg27183 CDS=1-1890protein length = 629 strand = +

MFYYFGIALFVVGQCHGQDAEKVASAPYSAVCELRTHGFYNLPQMSRALSTGSVSDAGAAFRQDFRDLCTDVNSKKYRTFDGSCNHPKNWGTSFKPLARMLPAEYDNGVSSPRRRGSLGTPLPSPRAISRFVHPSTPDLISRTIMIMQWGQWLDHDLSVSPVASEVNRVIKCCGPNGTRPVYPIDPNCFPIILPSDEENFAGQCMEFVRSSPALDKNNCTLRPREQINVLNSFVDASQVYGSTDELAARLRVEGDFLLKTKDEIFLPEDVASACIKRPGTNDYCFLAGDVRVNEHASLGAMHTIWLRAHNKIAKQLRALRPKDSNEEIFQLTRKIIGALQQVITYNEWLPIILGKHATTQKLPSKTGRTQRLLSADPRILNEFSTAALRFGHSFIPDVFPIGDRRVPLRQLFNRPAEVLDNFDDLVAGITGVAGGKSAQKIDRNFVVEITNHLFEPPTGSPGRGLDLIALNIQRSRDHGIPPYTAYRAACGLRPLTGFDDVDGLGPNVAQLARVYRSIDDIDLFTGLVHEPASPGSNALVGPTLACILGTQFYNLKFGDRFFFDTNDKLIAFTDNQLKSLRNTTLAKVICSNTNIEALTNNVFSFSAKANPLVPCQQLEEEGLNLSLFA

>Lsta_jg27183.t1 gene=jg27183 CDS=1-1887protein length = 628 strand = +

MFYYFGIALFVVGQCHGQDAEKVASAPYSVCELRTHGFYNLPQMSRALSTGSVSDAGAAFRQDFRDLCTDVNSKKYRTFDGSCNHPKNWGTSFKPLARMLPAEYDNGVSSPRRRGSLGTPLPSPRAISRFVHPSTPDLISRTIMIMQWGQWLDHDLSVSPVASEVNRVIKCCGPNGTRPVYPIDPNCFPIILPSDEENFAGQCMEFVRSSPALDKNNCTLRPREQINVLNSFVDASQVYGSTDELAARLRVEGDFLLKTKDEIFLPEDVASACIKRPGTNDYCFLAGDVRVNEHASLGAMHTIWLRAHNKIAKQLRALRPKDSNEEIFQLTRKIIGALQQVITYNEWLPIILGKHATTQKLPSKTGRTQRLLSADPRILNEFSTAALRFGHSFIPDVFPIGDRRVPLRQLFNRPAEVLDNFDDLVAGITGVAGGKSAQKIDRNFVVEITNHLFEPPTGSPGRGLDLIALNIQRSRDHGIPPYTAYRAACGLRPLTGFDDVDGLGPNVAQLARVYRSIDDIDLFTGLVHEPASPGSNALVGPTLACILGTQFYNLKFGDRFFFDTNDKLIAFTDNQLKSLRNTTLAKVICSNTNIEALTNNVFSFSAKANPLVPCQQLEEEGLNLSLFA

>Lsta_jg45101.t2 gene=jg45101 CDS=1-1998protein length = 665 strand = +

MGTLSFSYVIALTVVISRGQDVFPADAGDQDFWIRSLNATDMADFADLGLAAHRRTIQSDFITGMCMKDSDVLYNLPERVGAMSTGSISGFGVRLSQNYLQDCPNYYSKRFRTIDGTCNNPFNLGASATPVPRIVPPQYGDGVGAPRTKAADGSPLPGARFVSSAVHPAVNNLTNRPIIAMQWGQWIDHDMAGIAASLGPNGTALQCCGPNKTAPPAVTSPNCFPILLPPDEKDFVGTCMNFVRSLAATNEQGCEMKQRQQVNTITSFIDASPVYGSTENVARILRNPGDFLLKTKNTNFLPENVNGTCIRRPGTKDYCFLAGDFRVNQLPFLQSLHTIWLRAHNTIARRLRVLRPKDSNEETFERTRKLIGALTQAVTYNEWLPFVLGKEATKFNLVSRTGRTKTSLKVDPRIRQEFSTAAMRFGHSTVPDVFPIGQRRVPISQLFNRPGEVLDNFDDVVAGMVGVGTPGQNLIQQVDRNFVAGLTKHLFEPPNSPKNGLDLISFNIQRGRDHGIGPYTTYRKLCGLRPLTGFNDVSALGPNVAQLAKVYKSVNDIDLFTGLVHEPVDPSNQGLVGPTLTCILCQQFLNLKFGDRFFFDTDEKLIAFNDNQLKWIRQATLSKIICATTNIKALPNDIFNFPSKSNPLVSCDRLANDMLNLSAFA

>Lsta_jg45101.t1 gene=jg45101 CDS=1-2001protein length = 666 strand = +

MGTLSFSYVIALTVVISRGQADVFPADAGDQDFWIRSLNATDMADFADLGLAAHRRTIQSDFITGMCMKDSDVLYNLPERVGAMSTGSISGFGVRLSQNYLQDCPNYYSKRFRTIDGTCNNPFNLGASATPVPRIVPPQYGDGVGAPRTKAADGSPLPGARFVSSAVHPAVNNLTNRPIIAMQWGQWIDHDMAGIAASLGPNGTALQCCGPNKTAPPAVTSPNCFPILLPPDEKDFVGTCMNFVRSLAATNEQGCEMKQRQQVNTITSFIDASPVYGSTENVARILRNPGDFLLKTKNTNFLPENVNGTCIRRPGTKDYCFLAGDFRVNQLPFLQSLHTIWLRAHNTIARRLRVLRPKDSNEETFERTRKLIGALTQAVTYNEWLPFVLGKEATKFNLVSRTGRTKTSLKVDPRIRQEFSTAAMRFGHSTVPDVFPIGQRRVPISQLFNRPGEVLDNFDDVVAGMVGVGTPGQNLIQQVDRNFVAGLTKHLFEPPNSPKNGLDLISFNIQRGRDHGIGPYTTYRKLCGLRPLTGFNDVSALGPNVAQLAKVYKSVNDIDLFTGLVHEPVDPSNQGLVGPTLTCILCQQFLNLKFGDRFFFDTDEKLIAFNDNQLKWIRQATLSKIICATTNIKALPNDIFNFPSKSNPLVSCDRLANDMLNLSAFA

>Lsta_jg44931.t1 gene=jg44931 CDS=1-1884protein length = 627 strand = +

MQLRQICLCYLCILNNLEIGRTQEPTPEQTNPKPVQEFYVLASTHAAMSSGMFSGRGFKLTRRWYTWCLNATSYKYRTYDGSCNNVRNLGAAITAVPTLLKSEYADGMDSPRQYGEDGLPLPSARLVSTSVFPSAIELTNRTHMVMQWGQWVIHDLSAIPISTGANGTIKCCGPKGQPPPFINNRNCFPIEIPIHDRISKGRCMEFVRSLAATSRMGFTLKPRQQINIITSFIDASQIYGSGTIQTNGLRYGGYLLKTGLHGALPKAETQNCITRPNTTDYCQLAGDTRVNEQPALGVIHTFWVREHNRISQQLRVLKGNSSEEEIFQITRKIIAALQQIINYNEYLPIILGKDAHTWNLVSKVGRTAYNPALDPSVFNEFSTAAFRFGHSTIPDVLSFSDKAVKMRFLLHRPAECLNNCEGLMAGLAGLVGKKKKKALKKVDRNFVKEVTRFLFEAQASPGHKLDLAALNIQRGRDHGLAPYIKYRTFCGLPPVKGFDDEEALGPNVRDLALVYRSIADIDLFVGLLYEPVKGDASVGPTLKCLLGIQFFNLKFGDRFFFDTTDETLGFNDEQLASLRNMTLSKVMCHNSDLRELQLDVFTLPSKQNPTYTCQQLRSESIDLTLFA

>Lsta_jg37361.t1 gene=jg37361 CDS=1-1824protein length = 607 strand = +

MGEIWELRALWLLGVCVVLCARWPGVGACRFKDEIRERRHNVRRDVAACDPTSRYRSFDGSCNNLRHPTFGVAGSTFRRILAPKYENGDGRTPRLTGVTGAKLPSPRLVSVAVHDPGNHTAVNVNLMVMQWAQFLDHDVTRTPTTVASAPCCTAELVRSGVLHPDVTTGGPCFPIIVGSNDRLFTDLTTRCRDFTRSDPAVDDNGIRQQYNAITPWIDGSQIYGHTEELARSLRTLVNGKLRVVTINGEDFLPADEAATDETCFKLQPGDYCFKAGDVRVNAYPGLSALHTMFLRYHNKICDRLKAIHGDWSDEILYQEARRLVIAVIQRISYDEFMVQILGQAASKYGLLFGNYSYNSSIDPALSNVFSTAAYRFGHSLVSDSLTINGQVVETGDLFMRPKFVLNSLKNLTEALLTENCQRADRWYTKGMTDRMFEKPGGPKTGQDIVALNIQRGRDHGLPPYNEWLAYFGLPIKTFDTMEQGAIRYRGVYSSVDDIDLYSGAIGEFPVNGGSVGGLYSLILGDQFRDLKFGDRFWFENLGDVSSFTSDQVKQIARIKFSKVICDTVTGADGIKKVQLNAFRPVSSTNVLTDCAAFPDLDIERFWV

>Lsta_jg37362.t1 gene=jg37362 CDS=1-1797protein length = 598 strand = +

MLAFSVNRMFLSLLFLCVCWRHVSCYDEDHEEERHGVDPSGRNVTCDPTSRYRSFDGSCNNLRHPTFGVAGSTFRRILPPKYENGDGRTPRLTGVTGAKLPSPRLVSVTVHGPGNDTADDANMMLTQWGQFLAHDLTRTLQASFNGSCCSSDLVTSGVLHPDVVNGGPCFPIIVDRMDRHFDDVSTRCMEFQRSDHRTDLTNTRQQYNEATSWIDASHIYGHTEDRARSLRSFNNGKLKVITVNGEDFLPENVEATEKTCFKLQAGDYCFKAGDVRVNFYPGLSALHTMFLRYHNKICDRLKPIHGDWSDEILYQEARRLVIAVIQRITYDEFMVQILGQAASKYGLLFGNYSYNSSIDPTLSNVFSTAAYRFGHSLVSDSLTINGQVVETGDVFTRPKFVLNSLKGVTEAFLRGRSHGADRWFAKGMTDRMFEKPNKPKTGFDIVARNIQRGRDHGIRPYNDWLEHFGFPRATFETIGQVYGRVYSSVDDVDLYGGAAGETRAYQGRVGQLYSAILGAQFRDLKFGDRFWFENLDDVSSFTADQVSQIAGLKLSRVICDTVAGITKIQLNAFQTVSPENSLADCSTLADVDIERFWV

>Lsta_jg44932.t1 gene=jg44932 CDS=1-1365protein length = 454 strand = +

MVMQWGQWVIHDLSAIPITTGPNGAIKCCGPNGEPPPFINNRHCFPIEIPQNDTFSGGRCMEFVRSVAAHSHQGIPLRPRAQINSITSFIDASQVYGSETTQASGLRSGGYLLKTGEHGTLPKSATHNCVLRPNSTDYCQLAGDSRVNEQPGLCVLHTLWLREHNRISKQLRTLNFKSSDEEIFQVARKIIGEQIINYNDVFIEFSTAAFRFGHATIPEVLPFSDKVVRMRHLFHRPAECLNNFEGIMAGLAAVVNKQYKRALKKVDRNVGKEVSRFLFEAAAFPGRKLDLASLNIQRGRDHGLAPYIKYRTFCGLPPVKGFDDQEALGPYVRDLAKVYRSIADIDLFVGLLYEPVKGDASVGSTLTCLLGIQFFSLKFGDRFFFDTTDETLGFNDEQLLSLRNMTLAKVMCQNTNVQELQLDVFTLPSKQNPTYPCHQLRSESLDLTLFTQHD

>Lsta_jg31272.t1 gene=jg31272 CDS=1-4701protein length = 1566 strand = +

MKSWSLLLTGFIVCCSVQTLIGIEQERSPGDGWYNNLLHPDWGAIDTHLLRRSKVSYSDGVYEPSGINRPNPLTISRIAFNGTNNLSSVRNRNALLVFFGQQLVEEIMDAQRPGCPIEYLNIPVPKDHKYNPDQIDDLEMPFLRSRYDQRTGFSPNNPRQQLNEITPYMDGNLIYGSGKSVEDAVRSFRDGELLADNDDIKKSFPMKNDIRLPFANPPSPRDHVLRPVSRFRRFGNPRTHENPLLYSLAVVWFRYHNVIARQLKTTFPQLDDEQLFNAARKRVLAQYQKIVMYEWLPAWLSISEKGEKFNITGDYPYNGGGQNPYKGYDPNVHPGISTEFQAAAFRFGHTLVPPGIFTKKFESGTCINSTRPVQAKFTSKPNGAEENVDIEGIRLCNAYWVPQETVETETGMDEIIRGLTFTKATKEDNIIVTDLREDVFGPLDWSRRDLGALNIQRARDLGLPGYNDVREAYGLKRISNWTQINAEGLYGTQLRELKRLYNNSESPDDLDLFVGGLLETVPNGPGPLFQAIILDQFLRIRHGDRFWYENTQNGLFTADEIRDIENTNFYDVLKNVTNAFSMPQLVELGNDVFSCSNVNRTSKECQCVDPFLDIQDPHEQCVPLQHYDYFTGSAFPFIITIAAIVVSLPLTIGIMLLIARLRRMSMTPKTSKTKEKPQNGPNYFYATEWVGRTTYGTLNSRDVKIELDNQRMKILVSNISGQVVRMIDVRQRNNAEQKKPRASVTRSSDKGNRLMMMAAPGEIDLVLHFASRTDRDEAFEKLHEFFKKHGWEYHEAPNMAEQIMWREAMTIDQRKEVLARFFKSILVELSGQDSTLNRDPALVDEALNTRLTRTEFADALGLQPHSLFVRNMFLLVDSSGDGFVSFDEFKTYFGILASGKPEDKAKMFFQMFDTSRTGKLTKDNYKKMIMSLMELNEAGDGQNMNINNMVDAVFKQLGKDKVGFLTLEEFKSIMFSDTDDVWKSAVLNLDVGGETATIGKHKRSTVRDRAKSFIQGYQRNTKATSMVNFRSSQHVRLSSKADTAPKTNYQKFCRYVSNHNRQIFWVTLYTLVTLGIFVERAFYYSFEREHAGLRRMAGYGVSVTRGAASAQMFTYASLLVTMSRNTLTFFRETFLHRYIPFDNAHDMHFYVAGLAVLFTVIHVIGHVINFYHISTQPSSDLNCYFTEFFRPTHVLASFQYWTYNTITGLTGVGLVFVLVVLYVFAIPYARRNVFNFFRATHNLYIVVYIMLFLHGHARLVQVPLWPYYFLGPMVLFVLDKLVSVSRNKILLPVVRATLLPSGVINLIIKRPLTFNYQSGQWVRIACPNLGKGEYHPFTLTSAPHEQHLSLHIRAVGPWTSNFRHLFDPNVQQRSEIPKIYLDGPFGESHQDWYRYPVSVLVGGGIGITPFASILKDIANRSREVGRLPCRKVYFVWVTRTQQSFEWMTEIIRQVEAADTQDFVDINICITQIKEKFDLRTTMLYICERHFQKIAGMSMFTGLRARTHFGRPKFQDFFEALKFVHKEVGEIGVFSCGPPAMTNSVQQACTEQNAFTGPSLIHHFENF

>Lsta_jg60787.t1 gene=jg60787 CDS=1-393protein length = 130 strand = +

MIPGLFQDFTVYKYGGTSIHLCYEYLYIKFSNQNLEKKDALTSIRQKRYRSLDAFENYATTMKLLYKHILDVDLFVGGLLEKPVPGGVVGETFGHIIAHQFKDLKFGDAYFFLSKEEPQRFSNGWYYRNS

>Lsta_jg65571.t1 gene=jg65571 CDS=1-4665protein length = 1554 strand = +

MELSLRGVVLAGFIIALSGGVSFEEVPVQRFDGWYNNPLNPSWGGVGRALERNITPAYADDTYRPSGWDRPNPRWISNELFGADHLPRGQVANGRNLTALFAFFGQIVQHELIDTDDVTCPIEILETPVPRGDPEFDPEAEGNKHLPYERTSYDQNTGQSPNNPRRQLNRASSYIDGRFLLLNKFNSCLRSLNRASSYIDGSFLYGNSLVRTEFLRQPNSGKLACEDKWGKFPMRNNVNLPYHAFSFKFTRSEQLWRLGDTHVFENPAVLALNLAFYRYHNKVVDDILAGGDHRDKNSDELFDLARRQVIANIQNVMVYEWLPTLIGMELPPYTSYKSWVKSDVTDIFDAGAINYIMTLIPSGISQLEKTNCLVRNDGHFPAKRLCKTYWNAMETVYEHEDSTAEIILGLAHQMAENDDTYVVEDYRNKFYGPAYHTNHDAVLLTIMKGRDYGLPDYNTVRRTMGLEPKKSFEDVNIELNRTNPELMEAFKRMHKNDLSTVDMFVGGMMETTPDGPGELFRHILYDQFIRLRDGDRFWFENKENKLFTDDQIKAIRNTTLADILNKATDIPKKRLKDVFVNYGTDKECGPQRFFKTEELDDCPMNTSHDFFAGSEIPYIIIWTCLGLLPLVCIFVAYLLAKCKKWRHQRLLNSLKVEKEKRRILRRTISCSVSVHDALEWRGHDVAPRPVELHLTNKGCLEIFSTAGAHLRSIKVSTMPTLAIKMSSNKKRNVLGIIMPREYDLIVEFPSEPMRIEFGTMLCTFSQEHNVDLSVIELRLQDLYNAATTKEKRNRMLEKFFKTVFSEAFQMDYDPSLDAGSLDMKKHSQEILEVELSKEEFAEAMAVKANSDFVEHFFSLIDSDRNGYISFREFLNAVVLFSKGSIQEKLQTMFYMYDIDGTGYMSTKQIGQMFRSLLELAQSNLDTDDIDCLLESLSSQSGMEKKEQYSFEDFCQLLSPQMDKLWNASFDWKGLSNVMPPKTKSPKKAANGEPNLKKRGSVASLGALENGGSGSRFAISFEAVREKYTPLKAKVKLVKHFIENYRQHIFFMILFYGICAGLFAERFYYYTVEQEHKGFRKLMSYGISFTRGAAASMSFTFSFLLLTMCRNTITFLRSTPLNLFIPFDSHVSFHKIVAWTALFFSAIHIIGYSFNFYHLVSQPTRFLCIFTSLVFRPEFPLSFQQWVFGTMPGFTGVLLVLVLCILYVFATQTARRHIFSLFWLTHKLFIILYVLTIIHGASVVVQKPMFFAYLSGPAILFMIDKLVSLSRKKTELCIINAKNLPSDVTMIEFKRPPRFEYKSGQWVRIACLAQGKDEYHPFTLTSAPHEDTLKLHIRALGPWTWNLRHIFETESLKSSKSYPKLYLDGPYGAGQQDWYQYDVSVLVGAGIGVTPYASILKDFVHMASINMRYKVKCQKLYFIWITGSQRHFEWLLDIIREVEEVDTQGMVSIDIFITQFFQNFDLRTSLLYIFEEHFQKMNGGKSVFTGLKATTHFGRPQMNKIMEAVHRAHPQVRKVGVFSCGPPGVTKGVERACVDTSKVTKAMFEHHFENF
