# Supplementary material for: Mantle Modularity Underlies the Plasticity of the Molluscan Shell: Supporting Data From Cepaea nemoralis
Source: Front Genet. 2021 Feb 5;12:622400. doi: 10.3389/fgene.2021.622400 (PMC7894901; doi:10.3389/fgene.2021.622400)
Supplement: Supplementary file 12 [file Data_Sheet_12.docx]

**BLASTP 2.10.0+**

[**Reference**](https://www.ncbi.nlm.nih.gov/entrez/query.fcgi?db=PubMed&cmd=Retrieve&list_uids=9254694&dopt=Citation)**:**

Stephen F. Altschul, Thomas L. Madden, Alejandro A. Schäffer,

Jinghui Zhang, Zheng Zhang, Webb Miller, and David J. Lipman (1997),

"Gapped BLAST and PSI-BLAST: a new generation of protein database

search programs", Nucleic Acids Res. 25:3389-3402.

**[Reference for](https://www.ncbi.nlm.nih.gov/entrez/query.fcgi?db=PubMed&cmd=Retrieve&list_uids=11452024&dopt=Citation)**

**[composition-based statistics](https://www.ncbi.nlm.nih.gov/entrez/query.fcgi?db=PubMed&cmd=Retrieve&list_uids=11452024&dopt=Citation):**

Alejandro A. Schäffer, L. Aravind, Thomas L. Madden, Sergei

Shavirin, John L. Spouge, Yuri I. Wolf, Eugene V. Koonin, and

Stephen F. Altschul (2001), "Improving the accuracy of PSI-BLAST

protein database searches with composition-based statistics and

other refinements", Nucleic Acids Res. 29:2994-3005.

Database: All non-redundant GenBank CDS translations+PDB+SwissProt+PIR+PRF

excluding environmental samples from WGS projects

321,587,928 sequences; 115,933,041,032 total letters

**Query=** R27072766 chitin binding Periotrophin-A TransAbyss assembly 2

(filtered min reads 10, dedupe95) len=2716 num_reads=5670257

avg_cov=209491.4 contig_cov=100.0% (contig_821 from old CLC assemly

9) cds start = 286 cds stop = 2466 strand = - protein length = 727

strand = +

Length=727

Score E

Sequences producing significant alignments: (Bits) Value

[XP_013087207.1](https://www.ncbi.nlm.nih.gov/protein/XP_013087207.1?report=genbank&log$=prottop&blast_rank=1&RID=) PREDICTED: uncharacterized protein LOC106071610 [B... [348](#XP_013087207.1) 7e-106

[XP_025084945.1](https://www.ncbi.nlm.nih.gov/protein/XP_025084945.1?report=genbank&log$=prottop&blast_rank=2&RID=) uncharacterized protein LOC112558610 [Pomacea cana... [235](#XP_025084945.1) 2e-62

[PVD33241.1](https://www.ncbi.nlm.nih.gov/protein/PVD33241.1?report=genbank&log$=prottop&blast_rank=3&RID=) hypothetical protein C0Q70_04492 [Pomacea canaliculata] [224](#PVD33241.1) 1e-60

[XP_033751871.1](https://www.ncbi.nlm.nih.gov/protein/XP_033751871.1?report=genbank&log$=prottop&blast_rank=4&RID=) uncharacterized protein LOC117335790 [Pecten maximus] [168](#XP_033751871.1) 5e-39

[XP_011422884.2](https://www.ncbi.nlm.nih.gov/protein/XP_011422884.2?report=genbank&log$=prottop&blast_rank=5&RID=) uncharacterized protein LOC105325156 [Crassostrea ... [160](#XP_011422884.2) 2e-36

[XP_021377426.1](https://www.ncbi.nlm.nih.gov/protein/XP_021377426.1?report=genbank&log$=prottop&blast_rank=6&RID=) mucin-17-like [Mizuhopecten yessoensis] [158](#XP_021377426.1) 6e-36

[XP_011456399.2](https://www.ncbi.nlm.nih.gov/protein/XP_011456399.2?report=genbank&log$=prottop&blast_rank=7&RID=) protein PIF isoform X2 [Crassostrea gigas] [155](#XP_011456399.2) 2e-35

[XP_011456398.2](https://www.ncbi.nlm.nih.gov/protein/XP_011456398.2?report=genbank&log$=prottop&blast_rank=8&RID=) protein PIF isoform X1 [Crassostrea gigas] [155](#XP_011456398.2) 2e-35

[XP_009066027.1](https://www.ncbi.nlm.nih.gov/protein/XP_009066027.1?report=genbank&log$=prottop&blast_rank=9&RID=) hypothetical protein LOTGIDRAFT_236718 [Lottia gig... [146](#XP_009066027.1) 2e-34

[AYN73061.1](https://www.ncbi.nlm.nih.gov/protein/AYN73061.1?report=genbank&log$=prottop&blast_rank=10&RID=) VWA and chitin binding domain-containing protein 1 [Pi... [150](#AYN73061.1) 7e-34

[XP_009056819.1](https://www.ncbi.nlm.nih.gov/protein/XP_009056819.1?report=genbank&log$=prottop&blast_rank=11&RID=) hypothetical protein LOTGIDRAFT_239574 [Lottia gig... [149](#XP_009056819.1) 1e-33

[XP_033752252.1](https://www.ncbi.nlm.nih.gov/protein/XP_033752252.1?report=genbank&log$=prottop&blast_rank=12&RID=) protein PIF-like [Pecten maximus] [143](#XP_033752252.1) 1e-31

[XP_009066028.1](https://www.ncbi.nlm.nih.gov/protein/XP_009066028.1?report=genbank&log$=prottop&blast_rank=13&RID=) hypothetical protein LOTGIDRAFT_236719 [Lottia gig... [140](#XP_009066028.1) 3e-30

[XP_033752064.1](https://www.ncbi.nlm.nih.gov/protein/XP_033752064.1?report=genbank&log$=prottop&blast_rank=14&RID=) protein PIF-like [Pecten maximus] [138](#XP_033752064.1) 6e-30

[XP_022318425.1](https://www.ncbi.nlm.nih.gov/protein/XP_022318425.1?report=genbank&log$=prottop&blast_rank=15&RID=) LOW QUALITY PROTEIN: protein PIF-like [Crassostrea... [134](#XP_022318425.1) 8e-29

[XP_034330412.1](https://www.ncbi.nlm.nih.gov/protein/XP_034330412.1?report=genbank&log$=prottop&blast_rank=16&RID=) protein PIF [Crassostrea gigas] [127](#XP_034330412.1) 5e-28

[XP_009051492.1](https://www.ncbi.nlm.nih.gov/protein/XP_009051492.1?report=genbank&log$=prottop&blast_rank=17&RID=) hypothetical protein LOTGIDRAFT_228264 [Lottia gig... [131](#XP_009051492.1) 5e-28

[XP_009045199.1](https://www.ncbi.nlm.nih.gov/protein/XP_009045199.1?report=genbank&log$=prottop&blast_rank=18&RID=) hypothetical protein LOTGIDRAFT_237510 [Lottia gig... [120](#XP_009045199.1) 6e-27

[XP_022339846.1](https://www.ncbi.nlm.nih.gov/protein/XP_022339846.1?report=genbank&log$=prottop&blast_rank=19&RID=) asparagine-rich protein-like [Crassostrea virginica] [116](#XP_022339846.1) 6e-25

[XP_009057764.1](https://www.ncbi.nlm.nih.gov/protein/XP_009057764.1?report=genbank&log$=prottop&blast_rank=20&RID=) hypothetical protein LOTGIDRAFT_233460 [Lottia gig... [122](#XP_009057764.1) 8e-25

[XP_022335717.1](https://www.ncbi.nlm.nih.gov/protein/XP_022335717.1?report=genbank&log$=prottop&blast_rank=21&RID=) mucin-2-like [Crassostrea virginica] [113](#XP_022335717.1) 1e-21

[XP_021338925.1](https://www.ncbi.nlm.nih.gov/protein/XP_021338925.1?report=genbank&log$=prottop&blast_rank=22&RID=) uncharacterized protein LOC110440276 [Mizuhopecten... [112](#XP_021338925.1) 2e-21

[AKV63183.1](https://www.ncbi.nlm.nih.gov/protein/AKV63183.1?report=genbank&log$=prottop&blast_rank=23&RID=) P-U8 [Pinctada fucata] [113](#AKV63183.1) 2e-21

[XP_022290478.1](https://www.ncbi.nlm.nih.gov/protein/XP_022290478.1?report=genbank&log$=prottop&blast_rank=24&RID=) LOW QUALITY PROTEIN: uncharacterized protein LOC11... [108](#XP_022290478.1) 1e-20

[XP_021352990.1](https://www.ncbi.nlm.nih.gov/protein/XP_021352990.1?report=genbank&log$=prottop&blast_rank=25&RID=) uncharacterized protein LOC110450071 [Mizuhopecten... [107](#XP_021352990.1) 1e-19

[XP_021363830.1](https://www.ncbi.nlm.nih.gov/protein/XP_021363830.1?report=genbank&log$=prottop&blast_rank=26&RID=) protein PIF-like [Mizuhopecten yessoensis] [103](#XP_021363830.1) 6e-19

[XP_021377870.1](https://www.ncbi.nlm.nih.gov/protein/XP_021377870.1?report=genbank&log$=prottop&blast_rank=27&RID=) uncharacterized protein LOC110465975 [Mizuhopecten... [103](#XP_021377870.1) 7e-19

[XP_034298995.1](https://www.ncbi.nlm.nih.gov/protein/XP_034298995.1?report=genbank&log$=prottop&blast_rank=28&RID=) mucin-2-like [Crassostrea gigas] [104](#XP_034298995.1) 9e-19

[XP_011437524.2](https://www.ncbi.nlm.nih.gov/protein/XP_011437524.2?report=genbank&log$=prottop&blast_rank=29&RID=) uncharacterized protein LOC105335381 isoform X2 [C... [99.4](#XP_011437524.2) 3e-17

[XP_011437523.2](https://www.ncbi.nlm.nih.gov/protein/XP_011437523.2?report=genbank&log$=prottop&blast_rank=30&RID=) uncharacterized protein LOC105335381 isoform X1 [C... [99.4](#XP_011437523.2) 3e-17

[OWF55652.1](https://www.ncbi.nlm.nih.gov/protein/OWF55652.1?report=genbank&log$=prottop&blast_rank=31&RID=) Protein PIF [Mizuhopecten yessoensis] [95.5](#OWF55652.1) 2e-16

[CAC5411652.1](https://www.ncbi.nlm.nih.gov/protein/CAC5411652.1?report=genbank&log$=prottop&blast_rank=32&RID=) Protein PIF [Mytilus coruscus] [82.0](#CAC5411652.1) 7e-12

[XP_033752829.1](https://www.ncbi.nlm.nih.gov/protein/XP_033752829.1?report=genbank&log$=prottop&blast_rank=33&RID=) protein PIF-like [Pecten maximus] [79.0](#XP_033752829.1) 4e-11

[XP_005183079.1](https://www.ncbi.nlm.nih.gov/protein/XP_005183079.1?report=genbank&log$=prottop&blast_rank=34&RID=) PREDICTED: proteoglycan 4-like [Musca domestica] [63.9](#XP_005183079.1) 1e-06

>[XP_013087207.1](https://www.ncbi.nlm.nih.gov/protein/XP_013087207.1?report=genbank&log$=protalign&blast_rank=1&RID=0) PREDICTED: uncharacterized protein LOC106071610 [Biomphalaria

glabrata]

Length=682

Score = 348 bits (892), Expect = 7e-106, Method: Compositional matrix adjust.

Identities = 182/340 (54%), Positives = 234/340 (69%), Gaps = 14/340 (4%)

Query 269 TTTTTTEVITTPEATTTTTEAEPVTTTTTTTTIGAITTV-----KPSLELCNNCVVHHGV 323

TTTTT TT TTTTT TT TTTT+ + TT KPSL+LC +CV+ HGV

Sbjct 348 TTTTTQPTTTTTTPTTTTTTPTTTTTRPTTTTVTSTTTRTTTTGKPSLDLCRDCVILHGV 407

Query 324 GYAPLPGYCDAYVQCRFYGALPTAVDIRRCPSGNYWNQDKLVCDFQDNVKC-TPVNNCPN 382

GYAP PGYCDAY+QC+F G++P++ IRRCP+G WNQ+KL CDF + V+C + V CP

Sbjct 408 GYAPYPGYCDAYIQCQFSGSVPSSAIIRRCPTGLQWNQNKLTCDFPEIVQCKSVVAKCPR 467

Query 383 HKAIPGDWAAYSIFNGANWTRVACPERRLYNSVTCGCTDITGGFDGNHEICTDKKAIIGD 442

K I G+ Y FNG WT++ CP ++Y+ + CGCT+ GG+ GN E C DK+AI GD

Sbjct 468 QKEIVGNKQEYLSFNGYTWTQLRCPVNQVYSDLVCGCTENWGGY-GNFETCADKRAIAGD 526

Query 443 NTGFMQFTGNGWVRMACPATLGYNEQTCRCTDKLSPDTSISVCPNTKPIAGDKSGYLQFT 502

TG++Q + GW+RM CP TLGY+ TCRCTD L + CPNTKPI GD +GYLQF

Sbjct 527 KTGYLQLSSLGWIRMPCPKTLGYDAATCRCTDILETSDLYTNCPNTKPIPGDVTGYLQFN 586

Query 503 GVSWIRRPCPATLVYHADICVCSYDQTNVVDDDDNKSKQHGVCKATVALNFDNNNATDSS 562

GVSWIR PCPA++ Y + C C+Y T D +D C+ ++AL F++N+ATD+S

Sbjct 587 GVSWIRMPCPASVGYDSRTCQCTYRLTVKGDKEDK-------CEPSLALTFEDNSATDTS 639

Query 563 VNHFWVNNTGVTFNDGKAYFNGKSRLTIPGLSNMEFGSTV 602

+N FWVNNTGVTF +GKAYF+G+SRLT+PG SNM+ G+T

Sbjct 640 INQFWVNNTGVTFRNGKAYFDGQSRLTVPGFSNMDLGNTA 679

>[XP_025084945.1](https://www.ncbi.nlm.nih.gov/protein/XP_025084945.1?report=genbank&log$=protalign&blast_rank=2&RID=0) uncharacterized protein LOC112558610 [Pomacea canaliculata]

Length=854

Score = 235 bits (599), Expect = 2e-62, Method: Compositional matrix adjust.

Identities = 156/435 (36%), Positives = 201/435 (46%), Gaps = 82/435 (19%)

Query 313 LCNNCVVHHGVGYAPLPGYCDAYVQCRF-YGALPTAVDIRRCPSGNYWNQDKLVCDFQDN 371

+C+ C + HGVGY P P C YVQC+ LP +R CP G YWNQDKL CD++ N

Sbjct 481 MCDGCELRHGVGYKPHPSDCTLYVQCQADKNGLPVVAGVRPCPHGLYWNQDKLTCDYRHN 540

Query 372 VKCT----PVNNCPNHKAIPGDWAAYSIFNGANWTRVACPERRLYNSVTCGCTD------ 421

V C KA Y N CP YN CT

Sbjct 541 VNCVDDICRAGAVRKTKASAASCRGYWDCNSGTALAKCCPINYSYNPYIASCTFNPTCRD 600

Query 422 --ITGGFDGNHEICTDKKAIIGDNTGFMQFTGNGWVRMACPATLGYNEQTCRCTDKLSPD 479

+T E + + GD T + TGN W M+CPA LG++ C C L+ +

Sbjct 601 DCLTSTAPFVAECPNGMRPVPGDRTKYEVKTGNTWTLMSCPANLGFSTLPCGCNVHLNTN 660

Query 480 TSISVCPNTKPIAGDKSGYLQFTGVSWIRRPCPATLVYHADICVCSYDQTNVVDDDDNKS 539

P YL F + D D

Sbjct 661 VQQECVPEL---------YLPF-----------------------------ISDTQDQSG 682

Query 540 KQHGVCKATVALNFDNNNATDSSVNHFWVNNTGVTFNDGKAYFNGKSRLTIPGLSNMEFG 599

+Q +V N GV DGKA+F+GKSRLT+P SN +G

Sbjct 683 RQ------------------------VFVKNEGVQVRDGKAFFDGKSRLTVPRFSNTWWG 718

Query 600 STVYILIKYRHSSANSQQ-TLVSNGDCQVRQSLAVCSGKDSVDFYAETKEQISLGKTTVP 658

STVY+ ++Y+ + ++++ LVSNGDCQVR SLAVC+G V+FYAET+ TV

Sbjct 719 STVYVHLRYKSALTSTRRLALVSNGDCQVRPSLAVCAGPAGVEFYAETENSPQPVNFTVS 778

Query 659 T-DVG-----AWQYALYALDNGNLLGSVGVNKIAQPVKGALDRRQRGLVIGGGGGCDNFH 712

T D G WQ LY LD G+L G V +N+ ++ G L+ RQRGLVIG GGGCD+F+

Sbjct 779 TFDQGYGWQDGWQDVLYRLDAGSLYGHVSLNRDSRVALGNLEVRQRGLVIGSGGGCDDFN 838

Query 713 GIIDDVRVYLCKPEL 727

G ID+V VYLC+PEL

Sbjct 839 GFIDEVTVYLCRPEL 853

Score = 187 bits (475), Expect = 7e-46, Method: Compositional matrix adjust.

Identities = 150/441 (34%), Positives = 196/441 (44%), Gaps = 102/441 (23%)

Query 310 SLELCNNCVVHHGVGYAPLPGYCDAYVQCRF-YGALPTAVDIRRCPSGNYWNQDKLVCDF 368

S ++C+ C + HGVGY P P C YVQC+ LP +R

Sbjct 42 SQDMCDGCELRHGVGYKPHPSDCTLYVQCQADKNGLPVVAGVR----------------- 84

Query 369 QDNVKCTPVNNCPNHKAIPGDWAAYSIFNGANWT--RVACPERRLYNSVTCGCTDITGGF 426

CP+ G W ++ C R N C D

Sbjct 85 ----------PCPH---------------GLYWNQDKLTCDYRHNVN-----CVD----- 109

Query 427 DGNHEICT-----DKKAIIGDNTGFMQFTGNGWVRMACPATLGYNEQTCRCT-------D 474

+IC KA G+ + CP YN RCT D

Sbjct 110 ----DICRAGAVRKTKASAASCRGYWDCNSGTALAKCCPINYSYNPYIARCTYNPTCRDD 165

Query 475 KL-SPDTSISVCPN-TKPIAGDKSGYLQFTGVSWIRRPCPATLVYHADICVCS-YDQTNV 531

L S ++ CPN +P+ GD++ Y TG +W CPA L + C C+ + TNV

Sbjct 166 CLTSTAPFVAECPNGMRPVPGDRTKYEVKTGNTWTLMSCPANLGFSTLPCGCNVHLNTNV 225

Query 532 VDDDDNKSKQHGVCKATVALNFDNNNATDSSVNHFWVNNTGVTFNDGKAYFNGKSRLTIP 591

+ C + L F ++ D S +V N GV DGKA+F+GKSRLT+P

Sbjct 226 QQE----------CVPELYLPFISD-TQDQSGRQVFVKNEGVQVRDGKAFFDGKSRLTVP 274

Query 592 GLSNMEFGSTVYILIKYRHSSANSQQTLVSNGDCQVRQSLAVCSGKDSVDFYAETKEQIS 651

SN +GSTV + LVSNGDCQVR SLAVC+G V+FYAET+

Sbjct 275 RFSNTWWGSTV-----------TRRLALVSNGDCQVRPSLAVCAGPAGVEFYAETENSPQ 323

Query 652 LGKTTVPT-DVG-----AWQYALYALDNGNLLGSVGVNKIAQPVKGALDRRQRGLVIGGG 705

TV T D G WQ LY LD G+L G V +N+ ++ L+ RQRGLVIG G

Sbjct 324 PVNFTVSTFDQGYGWQDGWQDVLYRLDAGSLYGHVSLNRDSRVALANLEVRQRGLVIGSG 383

Query 706 GGCDNFHGIIDDVRVYLCKPE 726

GGCD+F+G ID+V VYLC+P+

Sbjct 384 GGCDDFNGFIDEVTVYLCRPK 404

>[PVD33241.1](https://www.ncbi.nlm.nih.gov/protein/PVD33241.1?report=genbank&log$=protalign&blast_rank=3&RID=0) hypothetical protein C0Q70_04492 [Pomacea canaliculata]

Length=547

Score = 224 bits (570), Expect = 1e-60, Method: Compositional matrix adjust.

Identities = 150/428 (35%), Positives = 195/428 (46%), Gaps = 82/428 (19%)

Query 313 LCNNCVVHHGVGYAPLPGYCDAYVQCRF-YGALPTAVDIRRCPSGNYWNQDKLVCDFQDN 371

+C+ C + HGVGY P P C YVQC+ LP +R CP G YWNQDKL CD++ N

Sbjct 181 MCDGCELRHGVGYKPHPSDCTLYVQCQADKNGLPVVAGVRPCPHGLYWNQDKLTCDYRHN 240

Query 372 VKCT----PVNNCPNHKAIPGDWAAYSIFNGANWTRVACPERRLYNSVTCGCTD------ 421

V C KA Y N CP YN CT

Sbjct 241 VNCVDDICRAGAVRKTKASAASCRGYWDCNSGTALAKCCPINYSYNPYIASCTFNPTCRD 300

Query 422 --ITGGFDGNHEICTDKKAIIGDNTGFMQFTGNGWVRMACPATLGYNEQTCRCTDKLSPD 479

+T E + + GD T + TGN W M+CPA LG++ C C L+ +

Sbjct 301 DCLTSTAPFVAECPNGMRPVPGDRTKYEVKTGNTWTLMSCPANLGFSTLPCGCNVHLNTN 360

Query 480 TSISVCPNTKPIAGDKSGYLQFTGVSWIRRPCPATLVYHADICVCSYDQTNVVDDDDNKS 539

P YL F + D D

Sbjct 361 VQQECVPEL---------YLPF-----------------------------ISDTQDQSG 382

Query 540 KQHGVCKATVALNFDNNNATDSSVNHFWVNNTGVTFNDGKAYFNGKSRLTIPGLSNMEFG 599

+Q +V N GV DGKA+F+GKSRLT+P SN +G

Sbjct 383 RQ------------------------VFVKNEGVQVRDGKAFFDGKSRLTVPRFSNTWWG 418

Query 600 STVYILIKYRHSSANSQQ-TLVSNGDCQVRQSLAVCSGKDSVDFYAETKEQISLGKTTVP 658

STVY+ ++Y+ + ++++ LVSNGDCQVR SLAVC+G V+FYAET+ TV

Sbjct 419 STVYVHLRYKSALTSTRRLALVSNGDCQVRPSLAVCAGPAGVEFYAETENSPQPVNFTVS 478

Query 659 T-DVG-----AWQYALYALDNGNLLGSVGVNKIAQPVKGALDRRQRGLVIGGGGGCDNFH 712

T D G WQ LY LD G+L G V +N+ ++ G L+ RQRGLVIG GGGCD+F+

Sbjct 479 TFDQGYGWQDGWQDVLYRLDAGSLYGHVSLNRDSRVALGNLEVRQRGLVIGSGGGCDDFN 538

Query 713 GIIDDVRV 720

G ID+VR+

Sbjct 539 GFIDEVRI 546

>[XP_033751871.1](https://www.ncbi.nlm.nih.gov/protein/XP_033751871.1?report=genbank&log$=protalign&blast_rank=4&RID=0) uncharacterized protein LOC117335790 [Pecten maximus]

Length=1717

Score = 168 bits (425), Expect = 5e-39, Method: Compositional matrix adjust.

Identities = 123/437 (28%), Positives = 175/437 (40%), Gaps = 73/437 (17%)

Query 302 GAITTVKPSLELCNNCVVHHGVGYAPLPGYCDAYVQCRFYGALPTAVDIRRCPSGNYWNQ 361

G + T P C C + +G G+ P P C +VQC F G A + CP G++WNQ

Sbjct 1330 GELETPPPGFNPCLGCKMVNGAGFNPHPTDCSKFVQCFFRGDDVIAF-YKNCPVGHFWNQ 1388

Query 362 DKLVCDFQDNVKCTPVNNCPNHKAIPGDWAAYSIFNGANWTRVACPERRLYNSVTCGCTD 421

+KL CD+ V C

Sbjct 1389 EKLTCDYAFRVNCV---------------------------------------------- 1402

Query 422 ITGGFDGNHEICTDKKAIIGDNTGFMQFTGNGWVRMACPATLGYNEQTCRCTDKLS--PD 479

D H + + A G F ++ CPA YN C S P

Sbjct 1403 ----HDMCHNLLVHRYANRGHCQAFWDCENGHAIQRCCPAGQAYNTSVAMCVKDPSCPPT 1458

Query 480 TSIS--------VCPNTKPIAGDKSGYLQ-FTGVSWIRRPCPATLVYHADICVCSYDQTN 530

S S VC + + + G+ + Y Q G W+ PC + C CS +

Sbjct 1459 CSWSAHAPPVKTVCKD-RAVPGNSAFYEQEVEGHGWLMMPCAPGTAFDQKECRCSEFSAS 1517

Query 531 VVDDDDNKSKQHGVCKATVALNFDNNNATDSSVNHFWVNNTGVTFNDGKAYFNGKSRLTI 590

+ D D + C A V LNFDN+ DSS + WV N GV +G A F+GKS L I

Sbjct 1518 LNDHDSD-------CTAEVILNFDND-VQDSSGKYVWVTNKGVEVKNGSAVFDGKSELLI 1569

Query 591 PGLSNMEFGSTVYILIKYRH--SSANSQQTLVSNGDCQVRQSLAVCSGKDSVDFYAETKE 648

+N +FG T + ++YR S+ S L+SNGDC S+ + + S+ F +T +

Sbjct 1570 QRFTNDDFGFTFIVRMRYRETGSTPYSNSALISNGDCGRDGSIVIATDPVSIRFGVDTDK 1629

Query 649 QISLGKTTVPTDVGAWQYALYALDNGNLLGSVGVNKIAQPVKGALDRRQRGLVIGGGGGC 708

L V W+ + L +G L G ++ V G ++RR + IG G G

Sbjct 1630 TRGLYSFRVKKPATEWKTVEFKLADGKLQGRANELVFSRNVPGRIERRACAIQIGHGWGL 1689

Query 709 DNFHGIIDDVRVYLCKP 725

+NF G ID + +Y CKP

Sbjct 1690 ENFVGDIDQLEIYKCKP 1706

>[XP_011422884.2](https://www.ncbi.nlm.nih.gov/protein/XP_011422884.2?report=genbank&log$=protalign&blast_rank=5&RID=0) uncharacterized protein LOC105325156 [Crassostrea gigas]

[XP_034338493.1](https://www.ncbi.nlm.nih.gov/protein/XP_034338493.1?report=genbank&log$=protalign&blast_rank=5&RID=0) uncharacterized protein LOC105325156 [Crassostrea gigas]

Length=1193

Score = 160 bits (404), Expect = 2e-36, Method: Compositional matrix adjust.

Identities = 127/463 (27%), Positives = 205/463 (44%), Gaps = 65/463 (14%)

Query 280 PEATTTTTEAEPVTTTTTTTTIGAITTVKPSLELCNNCVVHHGVGYAPLPGYCDAYVQCR 339

P T T +A+ + TT + + + + + LC C + +G G+ P CD +V C

Sbjct 774 PPHQTATPKAKIIAKLTTD--VMSEPSPREAKVLCEGCKMINGAGFNSHPNECDLFVHC- 830

Query 340 FYGALPTAVDIRRCPSGNYWNQDKLVCDFQDNVKCTPVNNCP-----NHKAIPGDWAAYS 394

++G L IR+CP G +WNQ L C++ + C P++ C +++A A +

Sbjct 831 YFGELGLRAIIRKCPFGQFWNQTILSCEYSERAYC-PMDRCAYIKDRDYEASENCRAYWE 889

Query 395 IFNGANWTRVACPERRLYNSVTC-GCTDITGGFDGNHEICTDKKAIIGDNTGFMQFTGNG 453

NG +R C R Y V GC A+ DN

Sbjct 890 CSNGH--SRGKCC-RYGYRYVRGEGC------------------ALDSDNI--------- 919

Query 454 WVRMACPATL-----GYNEQTCRCTDKLSPDTSISVCPNTKPIAGDKSGYLQFT-GVSWI 507

+ +CP + YN + DK+ PIA D+S Y Q G ++

Sbjct 920 -CKESCPMEMPTDLHSYNNAPAQSCDKI-------------PIANDRSHYQQLLPGTGYV 965

Query 508 RRPCPATLVYHADICVCSYDQTNVVDDDDNKSKQHGV-CKATVALNFDNNNATDSSVNHF 566

PC Y+ C C+ + + + ++ C+ V L+F N TDSS

Sbjct 966 TMPCAEGTHYNERKCTCTDQEPSYPTFKEPLVQEPPAGCRPEVKLDF-KNGVTDSSGKWT 1024

Query 567 WVNNTGVTFNDGKAYFNGKSRLTIPGLSNMEFGSTVYILIKYRHSSA---NSQQTLVSNG 623

+VNN GV +G+A FNG SRL IP +N+EFG T I ++Y+ N Q LV+NG

Sbjct 1025 YVNNQGVLIQNGEAIFNGDSRLLIPRFTNVEFGKTFVIRLRYKEEEKLKFNESQALVNNG 1084

Query 624 DCQVRQSLAVCSGKDSVDFYAETKEQISLGKTTVPTDVGAWQYALYALDNGNLLGSVGVN 683

DC S+ + + ++S+ + +T ++ S + G W+ Y + +G G +

Sbjct 1085 DCGDFGSIQIFTKRNSIGYVVKTTKEPSHVSLQIHKPHGEWKDVEYIVSDGKFEGYLNGV 1144

Query 684 KIAQPVKGALDRRQRGLVIGGGGGCDNFHGIIDDVRVYLCKPE 726

+ + G+++ RQ + IG G G +NF G + + +Y CKPE

Sbjct 1145 QATKWSMGSVESRQCAVQIGFGHGYNNFRGRLSLLEIYFCKPE 1187

>[XP_021377426.1](https://www.ncbi.nlm.nih.gov/protein/XP_021377426.1?report=genbank&log$=protalign&blast_rank=6&RID=0) mucin-17-like [Mizuhopecten yessoensis]

[OWF54947.1](https://www.ncbi.nlm.nih.gov/protein/OWF54947.1?report=genbank&log$=protalign&blast_rank=6&RID=0) Cell surface glycoprotein 1 [Mizuhopecten yessoensis]

Length=1550

Score = 158 bits (400), Expect = 6e-36, Method: Compositional matrix adjust.

Identities = 122/429 (28%), Positives = 179/429 (42%), Gaps = 59/429 (14%)

Query 302 GAITTVKPSLELCNNCVVHHGVGYAPLPGYCDAYVQCRFYGALPTAVDIRRCPSGNYWNQ 361

G + T P C C + +G G+ P P C YVQC F G TA + CP G +W+Q

Sbjct 1170 GVLETPPPGFNPCLGCKMVNGAGFNPHPTDCSKYVQCFFTGKNVTAF-YKNCPVGQFWSQ 1228

Query 362 DKLVCDFQDNVKCTPVNNCPNHKAIPGDWAAYSIFNGANWTRVACPERRLYNSVTCGCTD 421

++L CD+ V C + C N R N+ C

Sbjct 1229 EELTCDYAVRVNCI-HDMCHNLLV-----------------------HRYANNAHCQA-- 1262

Query 422 ITGGFDGNHEICTDKKAIIGD-NTGFMQFTGNGWVR-MACPATLGYNEQTCRCTDKLSPD 479

+ C D AI G+ +G VR CP T CT +P

Sbjct 1263 --------YWFCEDGHAIQRCCPPGYAYKASDGCVRDRTCPPT---------CTWSYTPP 1305

Query 480 TSISVCPNTKPIAGDKSGYLQ-FTGVSWIRRPCPATLVYHADICVCSYDQTNVVDDDDNK 538

+VC K + G+ + Y + G + PC + C CS ++ + D +

Sbjct 1306 KK-TVC-QDKALPGNAAFYQRNVEGHGLMTLPCAPGTNFDIKECRCSEFSASLSNKDTD- 1362

Query 539 SKQHGVCKATVALNFDNNNATDSSVNHFWVNNTGVTFNDGKAYFNGKSRLTIPGLSNMEF 598

CKA V L+FD + D+S N+ WV N GV +GKA F+GKS L I +N +F

Sbjct 1363 ------CKAEVILDFDKD-VQDTSGNYLWVTNKGVKVENGKAIFDGKSELLIQRFTNDDF 1415

Query 599 GSTVYILIKYRHS--SANSQQTLVSNGDCQVRQSLAVCSGKDSVDFYAETKEQISLGKTT 656

G T + I+YR S +S L+SNGDC S+ + + + F +T +

Sbjct 1416 GHTFTVRIRYREKEGSPHSNSALISNGDCGRDGSIVIATDPIGIRFGVDTDRTRDMYSFR 1475

Query 657 VPTDVGAWQYALYALDNGNLLGSVGVNKIAQPVKGALDRRQRGLVIGGGGGCDNFHGIID 716

+ W+ L +G L G + V G ++RR + IG G G +NF G +D

Sbjct 1476 IKKPKTDWKMVELKLADGKLQGRANERVFTRTVPGLIERRACAIQIGHGWGLNNFIGEMD 1535

Query 717 DVRVYLCKP 725

++ +Y CKP

Sbjct 1536 NLEIYKCKP 1544

>[XP_011456399.2](https://www.ncbi.nlm.nih.gov/protein/XP_011456399.2?report=genbank&log$=protalign&blast_rank=7&RID=0) protein PIF isoform X2 [Crassostrea gigas]

Length=771

Score = 155 bits (392), Expect = 2e-35, Method: Compositional matrix adjust.

Identities = 125/429 (29%), Positives = 195/429 (45%), Gaps = 44/429 (10%)

Query 314 CNNCVVHHGVGYAPLPGYCDAYVQCRFYGALPTAVDIRRCPSGNYWNQDKLVCDFQDNVK 373

C++C + +G G+ P C ++QC F + + CP GN+W+Q L C VK

Sbjct 355 CDSCKMSNGAGFTRHPTDCSKFIQCYFGNNGLKKMSYQECPWGNFWDQSSLTCQPAHRVK 414

Query 374 CTPVNNCPNHKAI----PGDWAAYSIFNGANWTRVACPERRLYNS-VTC----GCTDITG 424

C P + C + + + PG ++ +G + CP Y+S + C C D

Sbjct 415 C-PTDRCLDPEVLTYDLPGSCRSFWACDGGESIPMCCPYGTSYHSGIGCLPDNKCKDPCP 473

Query 425 GFDGNHEICTDKKAIIGDNTGFMQFTGNGWVR-MACPATLGYNEQTCRCTDKLSPDTSIS 483

H + +AI+ D + N +R M+ P KL+

Sbjct 474 PRPTQH---LNPRAIMSDK---IDSKKNAPLREMSLPGL----------KPKLNIKPRKP 517

Query 484 VCPNTKPIAGDKSGYLQFT-GVSWIRRPC-PATLVYHADICVCSYDQTNVVDDDDNKSKQ 541

VC + K + GD + + QF WI+ PC P T AD C C T V NK+ +

Sbjct 518 VC-DKKAVRGDSNSFEQFVERYGWIKMPCAPGTQYSQAD-CEC----TTTVPYSSNKTAE 571

Query 542 HGVCKATVALNFDNNNATDSSVNHFWVNNTGVTFNDGKAYFNGKSRLTIPGLSNMEFGST 601

C + + LNF + + ++ N V+F+ G A F+GKSRL +P LSN+++G +

Sbjct 572 ---CTSKLKLNFSDGFEDERDKRPVYIVNNNVSFSGGVAKFSGKSRLRVPQLSNVDYGDS 628

Query 602 VYILIKYRHS--SANSQQTLVSNGDCQVRQSLAVCSGKDSVDFYAETKEQISLGKTTVPT 659

V + I++R S S+ Q L+SN DC S+ + KD + F A T E + +P

Sbjct 629 VMLRIRFRDSTNSSGRPQALISNADCGNNASILIAKDKDRIIFGAHT-ENGGYNQIELPK 687

Query 660 DVGAWQYALYALDNGNLLGSVGVNKIAQ--PVKGALDRRQRGLVIGGGGGCDNFHGIIDD 717

W+ Y+ + G L GSV +K+ + P G + R L IG G D+F G IDD

Sbjct 688 PKTEWRNVKYSYNLGRLQGSVNTDKVTRWIPGGGKIQSRPCALQIGHGENLDDFEGDIDD 747

Query 718 VRVYL-CKP 725

+ ++ C P

Sbjct 748 LEIFTQCMP 756

>[XP_011456398.2](https://www.ncbi.nlm.nih.gov/protein/XP_011456398.2?report=genbank&log$=protalign&blast_rank=8&RID=0) protein PIF isoform X1 [Crassostrea gigas]

Length=775

Score = 155 bits (392), Expect = 2e-35, Method: Compositional matrix adjust.

Identities = 125/429 (29%), Positives = 195/429 (45%), Gaps = 44/429 (10%)

Query 314 CNNCVVHHGVGYAPLPGYCDAYVQCRFYGALPTAVDIRRCPSGNYWNQDKLVCDFQDNVK 373

C++C + +G G+ P C ++QC F + + CP GN+W+Q L C VK

Sbjct 359 CDSCKMSNGAGFTRHPTDCSKFIQCYFGNNGLKKMSYQECPWGNFWDQSSLTCQPAHRVK 418

Query 374 CTPVNNCPNHKAI----PGDWAAYSIFNGANWTRVACPERRLYNS-VTC----GCTDITG 424

C P + C + + + PG ++ +G + CP Y+S + C C D

Sbjct 419 C-PTDRCLDPEVLTYDLPGSCRSFWACDGGESIPMCCPYGTSYHSGIGCLPDNKCKDPCP 477

Query 425 GFDGNHEICTDKKAIIGDNTGFMQFTGNGWVR-MACPATLGYNEQTCRCTDKLSPDTSIS 483

H + +AI+ D + N +R M+ P KL+

Sbjct 478 PRPTQH---LNPRAIMSDK---IDSKKNAPLREMSLPGL----------KPKLNIKPRKP 521

Query 484 VCPNTKPIAGDKSGYLQFT-GVSWIRRPC-PATLVYHADICVCSYDQTNVVDDDDNKSKQ 541

VC + K + GD + + QF WI+ PC P T AD C C T V NK+ +

Sbjct 522 VC-DKKAVRGDSNSFEQFVERYGWIKMPCAPGTQYSQAD-CEC----TTTVPYSSNKTAE 575

Query 542 HGVCKATVALNFDNNNATDSSVNHFWVNNTGVTFNDGKAYFNGKSRLTIPGLSNMEFGST 601

C + + LNF + + ++ N V+F+ G A F+GKSRL +P LSN+++G +

Sbjct 576 ---CTSKLKLNFSDGFEDERDKRPVYIVNNNVSFSGGVAKFSGKSRLRVPQLSNVDYGDS 632

Query 602 VYILIKYRHS--SANSQQTLVSNGDCQVRQSLAVCSGKDSVDFYAETKEQISLGKTTVPT 659

V + I++R S S+ Q L+SN DC S+ + KD + F A T E + +P

Sbjct 633 VMLRIRFRDSTNSSGRPQALISNADCGNNASILIAKDKDRIIFGAHT-ENGGYNQIELPK 691

Query 660 DVGAWQYALYALDNGNLLGSVGVNKIAQ--PVKGALDRRQRGLVIGGGGGCDNFHGIIDD 717

W+ Y+ + G L GSV +K+ + P G + R L IG G D+F G IDD

Sbjct 692 PKTEWRNVKYSYNLGRLQGSVNTDKVTRWIPGGGKIQSRPCALQIGHGENLDDFEGDIDD 751

Query 718 VRVYL-CKP 725

+ ++ C P

Sbjct 752 LEIFTQCMP 760

>[XP_009066027.1](https://www.ncbi.nlm.nih.gov/protein/XP_009066027.1?report=genbank&log$=protalign&blast_rank=9&RID=0) hypothetical protein LOTGIDRAFT_236718 [Lottia gigantea]

[ESO83271.1](https://www.ncbi.nlm.nih.gov/protein/ESO83271.1?report=genbank&log$=protalign&blast_rank=9&RID=0) hypothetical protein LOTGIDRAFT_236718 [Lottia gigantea]

Length=360

Score = 146 bits (368), Expect = 2e-34, Method: Compositional matrix adjust.

Identities = 113/413 (27%), Positives = 173/413 (42%), Gaps = 63/413 (15%)

Query 319 VHHGVGYAPLPGYCDAYVQCRFYGALPTAVDIRRCPSGNYWNQDKLVCDFQDNVKCTPVN 378

+ +GVG+ + G C ++QC F + + ++ CP+G +W+QDKL C++ V CT

Sbjct 1 MRNGVGFNSVAGSCQKFIQCIFNLEMLISTILKDCPAGLFWDQDKLTCNYASEVDCTED- 59

Query 379 NCPNHKAIPGDWA-------AYSIFNGANWTRVACPERRLYNSVTCGCTDITGGFDGNHE 431

P + GD A Y+ FN ++ + P G+ N

Sbjct 60 --PCYSKPDGDIAHPTNCREYYTCFNSVSFEKCCLP-----------------GYAFN-- 98

Query 432 ICTDKKAIIGDNTGFMQFTGNGWVRMACPATLGYNEQTCRCTDKLSPDTSISVCPNTKPI 491

A G E C D C T+

Sbjct 99 -----------------------------AAAGKCESNSACVDSCKWGNPEEGC--TRRE 127

Query 492 AGDKSGYLQFTGVSWIRRPCPATLVYHADICVCSYDQTNVVDDDDNKSKQHGVCKATVAL 551

DK Y Q G WI CP +Y + C C + V+ + + CK +V L

Sbjct 128 IADKHSYEQAVGDEWIVMSCPLGALYSQEECKCGIYDEDYVNTPPPPAARDAECKPSVQL 187

Query 552 NFDNNNATDSSVNHFWVNNTGVTFNDGKAYFNGKSRLTIPGLSNMEFGSTVYILIKYR-H 610

+FD+ D S N +V N GV G AYF+G+S L IP +N++F TV I +KY+

Sbjct 188 SFDSGT-YDESGNFNYVQNNGVVVESGVAYFDGQSFLRIPRFANVDFRKTVTIKMKYKLD 246

Query 611 SSANSQQTLVSNGDCQVRQSLAVCSGKDSVDFYAETKEQISLGKTTVPTDVGAWQYALYA 670

+A Q+ LV+NGDC +QS+ + + + + + T+ + W Y

Sbjct 247 GAATGQEALVTNGDCGEKQSIYIVAEQSQTVLGLISADSADEKSATIASS-SDWNEVEYK 305

Query 671 LDNGNLLGSVGVNKIAQPVKGALDRRQRGLVIGGGGGCDNFHGIIDDVRVYLC 723

+ +G L+ +V + V G + R+ L IG G G DNF G ID++ VYLC

Sbjct 306 VVDGELISAVNGRQANTIVDGIIKRKHCSLQIGRGDGFDNFKGWIDELSVYLC 358

>[AYN73061.1](https://www.ncbi.nlm.nih.gov/protein/AYN73061.1?report=genbank&log$=protalign&blast_rank=10&RID=0) VWA and chitin binding domain-containing protein 1 [Pinctada

fucata]

Length=668

Score = 150 bits (378), Expect = 7e-34, Method: Compositional matrix adjust.

Identities = 117/422 (28%), Positives = 185/422 (44%), Gaps = 40/422 (9%)

Query 314 CNNCVVHHGVGYAPLPGYCDAYVQCRFYGALPTAVDIRRCPSGNYWNQDKLVCDFQDNVK 373

C+ C + +G G+ P CD +VQC F + ++CP GN+W Q L C V+

Sbjct 259 CDQCKMSNGAGFTRHPTDCDKFVQCYFGNNGLKKMVFQQCPWGNFWEQSSLTCKPAHRVQ 318

Query 374 CTPVNNCPNHKAI----PGDWAAYSIFNGANWTRVACPERRLY-NSVTC----GCTDITG 424

C P + C + + + PG ++ + + + CPE +Y + C C D

Sbjct 319 C-PTDKCRDPEVLTYELPGSCRSFWACDQGDSIPMCCPEGTMYAEGIGCLPDENCKDPCP 377

Query 425 GFDGNHEICTDKKAIIGDNTGFMQFTGNGWVRMACPATLGYNEQTCRCTDKLSPDTSISV 484

++ KK ++ Q + + + A +LG +SP I

Sbjct 378 PRPSGYQSPRTKKKLLS------QLSKDK--KEAVLKSLGA---------PVSPSKVIPK 420

Query 485 CPNTKPIAGDKSGYLQFTG-VSWIRRPCPATLVYHADICVCSYDQTNVVDDDDNKSKQHG 543

+ KP+ D S + QF W++ PC Y C C+ + +

Sbjct 421 VCDKKPVFDDPSHFEQFVDRFGWVKMPCAPGTAYDTKDCECTVRAKYTATKEQS------ 474

Query 544 VCKATVALNFDNNNATDSSVNHFWVNNTGVTFNDGKAYFNGKSRLTIPGLSNMEFGSTVY 603

CKA V LNF + +V N VTF G A F+G SRL +P LSN+++G V

Sbjct 475 -CKAKVKLNFTEGFEDERDKRPVYVVNNNVTFGQGVAKFDGTSRLRVPQLSNVDYGEAVV 533

Query 604 ILIKYRHS--SANSQQTLVSNGDCQVRQSLAVCSGKDSVDFYAETKEQISLGKTTVPTDV 661

+ IK+R + ++N Q ++SNGDC S+ V + + F AET + +P

Sbjct 534 LKIKFRETRDTSNKAQAIISNGDCGNNASILVAKDAEKITFGAETVGG-EYTQVEIPKPK 592

Query 662 GAWQYALYALDNGNLLGSV-GVNKIAQ-PVKGALDRRQRGLVIGGGGGCDNFHGIIDDVR 719

W+ YA ++G L G+V G +K A P G + R L IG G G +F G I+D+

Sbjct 593 TEWKTVKYAYNDGRLQGTVNGHSKSAWIPSGGRIQARPCALQIGHGEGLGDFQGDIEDIE 652

Query 720 VY 721

++

Sbjct 653 IF 654

>[XP_009056819.1](https://www.ncbi.nlm.nih.gov/protein/XP_009056819.1?report=genbank&log$=protalign&blast_rank=11&RID=0) hypothetical protein LOTGIDRAFT_239574 [Lottia gigantea]

[ESO92491.1](https://www.ncbi.nlm.nih.gov/protein/ESO92491.1?report=genbank&log$=protalign&blast_rank=11&RID=0) hypothetical protein LOTGIDRAFT_239574 [Lottia gigantea]

Length=760

Score = 149 bits (377), Expect = 1e-33, Method: Compositional matrix adjust.

Identities = 117/420 (28%), Positives = 177/420 (42%), Gaps = 58/420 (14%)

Query 314 CNNCVVHHGVGYAPLPGYCDAYVQC-RFYGALPTAVDIRRCPSGNYWNQDKLVCDFQDNV 372

CN C+ +GVG+ C + +C R A +++ CP G YWN + CD+ N

Sbjct 386 CNRCIYRNGVGFLSHTSDCTKFFKCQRLSNGGFRAAELQ-CPFGLYWNNEIFSCDYPRNT 444

Query 373 KCT--PVNNCPNHKA-IPGDWAAYSIFNGANWTRVACPERRLYNSVTCGCTDITGGFDGN 429

CT P N A + G A Y + A CP+ F +

Sbjct 445 NCTNHPCTNTNTRYAEMTGHCAGYWRCDWATPVAYCCPQGHR--------------FQQS 490

Query 430 HEIC-TDKKAIIGDNTGFMQFTGNGWVRMACPATLGYNEQTCRCTDKLSPDTSISVCPNT 488

++C D+ D+ Q +G V C DK S

Sbjct 491 SQLCEVDRTRTCRDDCSGPQQKPSGVVEKLC--------------DKRS----------- 525

Query 489 KPIAGDKSGYLQFTGVS-WIRRPCPATLVYHADICVCSYDQTN--VVDDDDNKSKQHGVC 545

++ K+ + Q V+ WIR C + ++ C+CS D+ N V NK C

Sbjct 526 --VSDSKTAFEQRVPVNRWIRLDCAPSTGFNPKTCLCS-DRVNAPVTQKTSNKD-----C 577

Query 546 KATVALNFDNNNATDSSVNHFWVNNTGVTFNDGKAYFNGKSRLTIPGLSNMEFGSTVYIL 605

+ ++F NN D S + FW+ N GVT G FNG ++L + +N G + I

Sbjct 578 DPMLHISF-NNGVRDESRHRFWIENVGVTSKAGVGLFNGNNKLLVNRFANAPLGRDLVIE 636

Query 606 IKYRHSSANSQQTLVSNGDCQVRQSLAVCSGKDSVDFYAETKEQISLGKTTVPTDV-GAW 664

+ Y + + LVSNGDC V+ SL + +G V F +T + TVP +

Sbjct 637 VVYEPADRRRDEVLVSNGDCGVKPSLYIVTGPTGVTFSVKTTRSSTPSVVTVPISMPKGL 696

Query 665 QYALYALDNGNLLGSVGVNKIAQPVKGALDRRQRGLVIGGGGGCDNFHGIIDDVRVYLCK 724

A +L NG L G VG + P G+++ R+ L+IG G G F GI+DDV+++ C+

Sbjct 697 IKARLSLANGRLTGEVGGLSKSTPASGSVELRKSSLIIGSGDGMKKFDGIMDDVKMFFCQ 756

>[XP_033752252.1](https://www.ncbi.nlm.nih.gov/protein/XP_033752252.1?report=genbank&log$=protalign&blast_rank=12&RID=0) protein PIF-like [Pecten maximus]

Length=668

Score = 143 bits (361), Expect = 1e-31, Method: Compositional matrix adjust.

Identities = 127/429 (30%), Positives = 189/429 (44%), Gaps = 43/429 (10%)

Query 311 LELCNNCVVHHGVGYAPLPGYCDAYVQCRFYGALPTAVDIRRCPSGNYWNQDKLVCDFQD 370

L+ C+ C + +G G+ P CD +VQC F I+ CP GN+W+QD + C

Sbjct 266 LDPCDGCRMKNGAGFNRHPTDCDKFVQCYFTADGKRQHSIQECPWGNFWDQDSMTCQPAH 325

Query 371 NVKCTPVNNC--PNHKAIP--GDWAAYSIFNGANWTRVACPERRLYNSVTCGCTDITGGF 426

V C + C P +A D ++ N + CP + GC

Sbjct 326 MVYCL-TDKCGDPYMQAYKYTADCRSFWYCNSGKSFPMCCPANTSFIE-NVGCVPD---- 379

Query 427 DGNHEICTDKKAIIGDNTGFMQFTGNGWVRMACPATLGYNEQTCRCTDKLSPDTSISVCP 486

+G E C + + G+ F++ N A + +T T + C

Sbjct 380 NGCQEHCPPRDS--GNPPRFVKRNHNPDEPPVLAAPVPRTGET---------PTKQTEC- 427

Query 487 NTKPIAGDKSGYLQFTGVS-WIRRPCPATLVYHADICVCSYDQTNVVDDDDNKSKQHGVC 545

+ K I+GD + Q S W+R PC Y+ C C+ + D+ C

Sbjct 428 DKKEISGDPKHFEQLVPRSGWVRMPCAPGTSYNQRDCQCTVMSEYIKKDEG--------C 479

Query 546 KATVALNFDN--NNATDSSVNHFWVNNTGVTFNDGKAYFNGKSRLTIPGLSNMEFGSTVY 603

K+ V+LNF D SV +V N V+ G A F+GKS L +P LSNM+ G TV

Sbjct 480 KSKVSLNFATYIEETKDKSV---YVVNENVSVAHGVAIFDGKSSLRVPQLSNMDLGDTVV 536

Query 604 ILIKY-----RHSSANSQ-QTLVSNGDCQVRQSLAVCSGKDSVDFYAETKEQISLGKTTV 657

+ I+Y RH A S+ Q L+SNGDC S+ + S+ F AET ++ ++

Sbjct 537 LKIRYKDKPRRHPRATSRPQALISNGDCGNNASVLLAKDDSSIMFGAET-DKGGYTSFSI 595

Query 658 PTDVGAWQYALYALDNGNLLGSVGVNKIAQPVKGALDRRQRGLVIGGGGGCDNFHGIIDD 717

PT W+ Y ++G L G V AQ +G ++RR L IG G D+F G +DD

Sbjct 596 PTPDTEWKDVTYFFNDGVLEGKVNDVIYAQRSEGLIERRPCALQIGHGQDMDDFEGEMDD 655

Query 718 VRVYLCKPE 726

+ + CKP+

Sbjct 656 IEFFTCKPD 664

>[XP_009066028.1](https://www.ncbi.nlm.nih.gov/protein/XP_009066028.1?report=genbank&log$=protalign&blast_rank=13&RID=0) hypothetical protein LOTGIDRAFT_236719 [Lottia gigantea]

[ESO83272.1](https://www.ncbi.nlm.nih.gov/protein/ESO83272.1?report=genbank&log$=protalign&blast_rank=13&RID=0) hypothetical protein LOTGIDRAFT_236719 [Lottia gigantea]

Length=1260

Score = 140 bits (353), Expect = 3e-30, Method: Compositional matrix adjust.

Identities = 124/439 (28%), Positives = 178/439 (41%), Gaps = 53/439 (12%)

Query 290 EPVTTTTTTTTIGAITTVKPSLELCNNCVVHHGVGYAPLPGYCDAYVQC--RFYGALPTA 347

P +TT I A T PS +C+ C+ +GV P C+ ++QC ++ G+

Sbjct 870 RPGSTTLNIIRISA-TPSFPSY-VCDGCLYKNGVYLKDHPTDCNKFLQCNQKYDGSYD-- 925

Query 348 VDIRRCPSGNYWNQDKLVCDFQDNVKCTPVNNCPNHKAIPGDWAAYSIFNGANWTRVACP 407

V ++ CP G +W+QD L+C++ +N CT + C D++ YS C

Sbjct 926 VIVKDCPQGLFWDQDLLLCNYPENTNCTK-DPCYTL----ADFSTYSDIENNCRQYFKCV 980

Query 408 ERRLYNSVTCGCTDITGGFDGNHEICTDKKAIIGDNTGFMQFTGNGWVRMACPATLGYNE 467

Y C + GFD RM P+T

Sbjct 981 NGISY----LECCESGYGFDK-------------------------VTRMCSPST----- 1006

Query 468 QTCRCTDKLSPDTSISVCPNTKPIAGDKSGYLQ-FTGVSWIRRPCPATLVYHADICVCS- 525

CT+ + VC + + + G+ + Q WIR C ++ C CS

Sbjct 1007 ---TCTEACPSAYVMDVC-DKRAVVGNALVFEQKIPSFGWIRMNCALGTAFNETACYCSS 1062

Query 526 YDQTNVVDDDDNKSKQHGVCKATVALNFDNNNATDSSVNHFWVNNTGVTFNDGKAYFNGK 585

+ + + VC + L FDNN D S N F+V N GV DG AYF+G

Sbjct 1063 FVDVGYLIPVSSGPTPSNVCTPELYLPFDNN-TRDQSGNGFYVQNNGVVVEDGVAYFDGN 1121

Query 586 SRLTIPGLSNMEFGSTVYILIKYRHSSANSQ-QTLVSNGDCQVRQSLAVCSGKDSVDFYA 644

S L IP +N++FG+ + I KY+ S Q LV+N DC SL + S D V F

Sbjct 1122 SSLRIPRFTNVDFGTKLRITFKYKVDGIRSTPQALVTNSDCGEEGSLYIVSDPDQVTFST 1181

Query 645 ETKEQISLGKTTVPTDVGAWQYALYALDNGNLLGSVGVNKIAQPVKGALDRRQRGLVIGG 704

T S W + DNG G+V Q + G++ R L IG

Sbjct 1182 RTDNLNSPLPALTTLSSSEWNEVEFKYDNGVFTGTVNGASNTQIIMGSIKRNHYALQIGR 1241

Query 705 GGGCDNFHGIIDDVRVYLC 723

G NF G ID+V VY+C

Sbjct 1242 GDQFSNFKGWIDEVYVYMC 1260

>[XP_033752064.1](https://www.ncbi.nlm.nih.gov/protein/XP_033752064.1?report=genbank&log$=protalign&blast_rank=14&RID=0) protein PIF-like [Pecten maximus]

Length=662

Score = 138 bits (347), Expect = 6e-30, Method: Compositional matrix adjust.

Identities = 133/437 (30%), Positives = 186/437 (43%), Gaps = 86/437 (20%)

Query 314 CNNCVVHHGVGYAPLPGYCDAYVQCRF--YGALPTAVDIRRCPSGNYWNQDKLVC--DFQ 369

C++C + +GVGY P P C + QC F +G + A R CP G +++ + L C FQ

Sbjct 286 CDDCSMMNGVGYNPHPKICSKFTQCYFGPHGNMRAAY--RECPFGFFFDTNVLSCRLSFQ 343

Query 370 ---DNVKCT--PVNNCPNHKAIPGDWAAYSIFNGANWTRVACPERRLYNSVTCGCTDITG 424

+N KC P +K I A + NG + R

Sbjct 344 SHCNNDKCMLRPKLKSYPYKGIQNCRAYWLCKNGRSAAR--------------------- 382

Query 425 GFDGNHEICTDKKAIIGDNTGFMQFTGNGWVRMACPATLGYNEQTCRCTDKLSPD-TSIS 483

C DK GF G + C G C D+ P TSIS

Sbjct 383 --------CCDK--------GFRYRRWGG--KHKCVKDPG-------CQDECPPTITSIS 417

Query 484 VCPNTKPIAGDKSGYLQFTGV--SWIRRPCPATLVYHADICVCSYDQTNVVDDDDNKSKQ 541

+C T+ + G+ Y QF WI RPC Y+AD C C+ T+V D

Sbjct 418 IC-ETRLVFGNPKIYEQFIKGWNMWIPRPCAPGSQYNADKCACTGHTTHVPDKR------ 470

Query 542 HGVCKATVALNFDNNNATDSSVNHFWVNNTGVT-FNDGKAYFNGKSRLTIPGLSNMEFGS 600

CK V L F N D S +H +V N V +G AYFNG+S+L IP SN EF

Sbjct 471 --TCKPEVYLPF-TKNLKDQSGSHSYVQNYNVRQTKEGYAYFNGRSKLIIPRYSNAEFKD 527

Query 601 TVYILIKYRHSSANSQQTL---------VSNGDC-QVRQSLAVCSGKDSVDFYAETKEQI 650

V IK R + S TL VSN DC SL + GK S+ + A ++ +

Sbjct 528 LV---IKIRFKAKMSHTTLAMRRKLTALVSNSDCCNDDVSLLMILGKSSLHYMAISQNR- 583

Query 651 SLGKTTVPTDVGAWQYALYALDNGNLLGSVGVNKIAQPVKGALDRRQRGLVIGGGGGCDN 710

+ K +P W A + D L G + +P++G + R G+ IG G G ++

Sbjct 584 KMSKFILPV-CDDWNTAYFVHDTKTLYGRCNSKEKTRPLRGNIKRTHTGIHIGYGRGFNS 642

Query 711 FHGIIDDVRVYLCKPEL 727

F G ID++++Y C+P+L

Sbjct 643 FKGYIDEIKIYRCQPDL 659

>[XP_022318425.1](https://www.ncbi.nlm.nih.gov/protein/XP_022318425.1?report=genbank&log$=protalign&blast_rank=15&RID=0) LOW QUALITY PROTEIN: protein PIF-like [Crassostrea virginica]

Length=601

Score = 134 bits (336), Expect = 8e-29, Method: Compositional matrix adjust.

Identities = 119/428 (28%), Positives = 190/428 (44%), Gaps = 43/428 (10%)

Query 314 CNNCVVHHGVGYAPLPGYCDAYVQCRFYGALPTAVDIRRCPSGNYWNQDKLVCDFQDNVK 373

C++C + +G G+ P C ++QC F + + C GN+W+Q L C VK

Sbjct 188 CDSCKMSNGAGFTRHPTDCSKFIQCYFGNNGLKKMSYQECXWGNFWDQTSLTCQPAHRVK 247

Query 374 CTPVNNCPNHKAI----PGDWAAYSIFNGANWTRVACPERRLYNSVTCGCTDITGGFDGN 429

C P + C + + + PG ++ +G + CP Y+S GC D

Sbjct 248 C-PTDRCLDPELLTYDLPGSCRSFWXCDGGESIPMCCPYGTSYHSGX-GCLPDNKCKDPC 305

Query 430 HEICT---DKKAIIGDNTGFMQFTGNGWVRMACPATLGYNEQTCRCTDKLSPDTSISVCP 486

T + +AI+ D T + ++ P KL VC

Sbjct 306 PPRPTQYLNPRAIMSDKTNPEK--NAPLSELSLPGQ----------KPKLKIKPRKPVC- 352

Query 487 NTKPIAGDKSGYLQFT-GVSWIRRPC-PATLVYHADICVCSYDQTNVVDDDDNKSKQHGV 544

+ K + GD + + QF WI+ PC P T D C C T V NK+ +

Sbjct 353 DKKAVRGDSNSFEQFVERYXWIKMPCAPGTQFSQGD-CEC----TTTVPYSSNKTAE--- 404

Query 545 CKATVALNFDNNNATDSSVNHFWVNNTGVTFNDGKAYFNGKSRLTIPGLSNMEFGSTVYI 604

C + V LNF + + ++ N V+F+ G A F+G SRL +P LSN+++G++V +

Sbjct 405 CTSKVKLNFSDGFEDERDKRPVYIVNNNVSFSGGVAKFSGSSRLRVPQLSNVDYGNSVML 464

Query 605 LIKYRHS--SANSQQTLVSNGDCQVRQSLAVCSGKDSVDFYAETKEQISLGKTTVPTDVG 662

I++R S S+ Q + SN DC S+ + KD + F A+T E + +P

Sbjct 465 KIRFRDSNNSSGRPQAIXSNADCGNNASILIAKDKDRIIFGAQT-ENGDYTQIELPKPKT 523

Query 663 AWQYALYALDNGNLLGSVGVNKIAQ----PVKGALDRRQRGLVIGGGGGCDNFHGIIDDV 718

W+ Y+ + G L G+V +K+ + VK + D R L IG G ++F G IDD+

Sbjct 524 EWKNVKYSYNLGRLQGTVNTDKVTRWIPVEVKSSPD---RALQIGHGENFEDFEGDIDDL 580

Query 719 RVYL-CKP 725

++ C P

Sbjct 581 EIFTQCMP 588

>[XP_034330412.1](https://www.ncbi.nlm.nih.gov/protein/XP_034330412.1?report=genbank&log$=protalign&blast_rank=16&RID=0) protein PIF [Crassostrea gigas]

Length=367

Score = 127 bits (320), Expect = 5e-28, Method: Compositional matrix adjust.

Identities = 112/413 (27%), Positives = 171/413 (41%), Gaps = 62/413 (15%)

Query 322 GVGYAPLPGYCDAYVQCRFYGALPTAVDIRRCPSGNYWNQDKLVCDFQDNVKCTPVNNCP 381

GVG+ P CD YV+C F T + R+CP G YW+Q+++ C V+C P + C

Sbjct 4 GVGFLKHPEDCDKYVECYFGKNGKTEAEYRQCPFGMYWDQEEIKCRASAEVQC-PKDKC- 61

Query 382 NHKAIPGDWAAYSIFNGANWTRVACPERRLYNSVTCGCTDITGGFDGNHEICTDKKAIIG 441

+IP + V+ P + N H +C+ K+

Sbjct 62 ---SIP--------------SIVSYPFGNMENCKA-------------HWVCSHGKS--- 88

Query 442 DNTGFMQFTGNGWVRMACPATLGYNE-QTC----RCTDKLSPDTSISVCPNTKPIAGDKS 496

V M CP GY + C C D + P C N + +

Sbjct 89 -------------VPMCCPEGQGYVPFKGCVPQSNCKD-ICPLEKKEEC-NKRMTWDELD 133

Query 497 GYLQF-TGVSWIRRPCPATLVYHADICVCSYDQTNVVDDDDNKSKQHGVCKATVALNFDN 555

Y +F G W+++ C ++ +D C C + DN ++ VC+A V + FDN

Sbjct 134 KYEEFFYGYGWVKKCCKPGHIFDSDSCQCIPLHNGKGYEMDNDKQK--VCRAVVHIPFDN 191

Query 556 NNATDSSVNHFWVNNTGV-TFNDGKAYFNGKSRLTIPGLSNMEFGSTVYILIKYRHSSAN 614

N D S N V N V +G YF+G ++L IP M+ ST I ++Y +

Sbjct 192 N-CLDKSGNQVIVENHNVQLLGNGLGYFDGNAKLVIPRFPEMQSASTFVIKMRYLDAPTK 250

Query 615 SQQTLVSNGDCQVRQ-SLAVCSGKDSVDFYAETKEQISLGKTTVPTDVGAWQYALYALDN 673

Q L+SNGDC+ Q ++ + G+ V+F AE+ + +P W A + D

Sbjct 251 HMQGLLSNGDCEREQPTMFMVKGRAGVNFMAESAKN-KFTTFHLPAKPSCWNEAYFIHDF 309

Query 674 GNLLGSVGVNKIAQPVKGALDRRQRGLVIGGGGGCDNFHGIIDDVRVYLCKPE 726

+ G + + G + Q GL IG G NF G +D V +Y C+ E

Sbjct 310 NTMEGYINGKSCKRWTYGPIKMAQGGLNIGAVRGFQNFQGFMDYVTIYYCQEE 362

>[XP_009051492.1](https://www.ncbi.nlm.nih.gov/protein/XP_009051492.1?report=genbank&log$=protalign&blast_rank=17&RID=0) hypothetical protein LOTGIDRAFT_228264 [Lottia gigantea]

[ESO97635.1](https://www.ncbi.nlm.nih.gov/protein/ESO97635.1?report=genbank&log$=protalign&blast_rank=17&RID=0) hypothetical protein LOTGIDRAFT_228264 [Lottia gigantea]

Length=606

Score = 131 bits (330), Expect = 5e-28, Method: Compositional matrix adjust.

Identities = 86/236 (36%), Positives = 117/236 (50%), Gaps = 10/236 (4%)

Query 489 KPIAGDKSGYLQFT-GVSWIRRPCPATLVYHADICVCSYDQTNVVDDDDNKSKQHGVCKA 547

+ ++ D S Y QF GV W+R+PC + C C+ D G CKA

Sbjct 380 RAVSDDISSYEQFVRGVGWVRKPCAPGSAFSPVECSCTVAI-------DPLPINAGECKA 432

Query 548 TVALNFDNNNATDSSVNHFWVNNTGVTFNDGKAYFNGKSRLTIPGLSNMEFGSTVYILIK 607

V + FD++ A D S N +V N GV GK YFNG S L IP SN+EFGS V I ++

Sbjct 433 EVYIPFDDDVAIDKSGNGNYVENEGVFVIGGKGYFNGTSGLRIPRFSNIEFGSKVVITMR 492

Query 608 YRHSSANSQQTLVSNGDCQVRQSLAVCSGKDSVDFYAETKEQISLGKTTVPTDVGAWQYA 667

Y+ S Q L+SNGDC SL V + F +T + G T+P+ G W

Sbjct 493 YKAESIYGSQGLISNGDCGKPGSLLVAIDNTNTLFGLQTVSGTA-GIVTIPSANG-WNEI 550

Query 668 LYALDNGNLLGSVGVNKIAQPVKGALDRRQRGLVIGGGGGCDNFHGIIDDVRVYLC 723

+Y ++ L GSV N + + GA+ R Q L +G NF G +D++ VYLC

Sbjct 551 IYQVEGDVLTGSVNGNSAHKTIDGAVKRSQCALQVGRATHLSNFRGYVDELTVYLC 606

Score = 64.7 bits (156), Expect = 1e-06, Method: Compositional matrix adjust.

Identities = 54/177 (31%), Positives = 80/177 (45%), Gaps = 18/177 (10%)

Query 314 CNNCVVHHGVGYAPLPGYCDAYVQCRFYGALPTAVD--IRRCPSGNYWNQDKLVCDFQDN 371

C NC + +G+G+ P P CD Y QC F +L V+ +R+C G +W+QD L C++

Sbjct 252 CANCKMSNGIGFNPHPTDCDKYFQCEF--SLEGLVNSVLRQCGQGLFWDQDLLTCNYPAA 309

Query 372 VKCTPVNNCPNHKAIPGDWAA-----YSIFNGANWTRVACPERRLYNSVTC----GCTDI 422

V+C + C N+ A YS NG + C + Y S C C

Sbjct 310 VQCR-ADPCQNYHISSYKKAGNCREYYSCSNGTSMPE-CCKKGFAYVSGQCVPSYNCNAH 367

Query 423 TGGFDGNHEICTDKKAIIGDNTGFMQFT-GNGWVRMACPATLGYNEQTCRCTDKLSP 478

G D + C + +A+ D + + QF G GWVR C ++ C CT + P

Sbjct 368 CKG-DFINPYC-EMRAVSDDISSYEQFVRGVGWVRKPCAPGSAFSPVECSCTVAIDP 422

>[XP_009045199.1](https://www.ncbi.nlm.nih.gov/protein/XP_009045199.1?report=genbank&log$=protalign&blast_rank=18&RID=0) hypothetical protein LOTGIDRAFT_237510 [Lottia gigantea]

[ESP04114.1](https://www.ncbi.nlm.nih.gov/protein/ESP04114.1?report=genbank&log$=protalign&blast_rank=18&RID=0) hypothetical protein LOTGIDRAFT_237510 [Lottia gigantea]

Length=213

Score = 120 bits (301), Expect = 6e-27, Method: Compositional matrix adjust.

Identities = 78/218 (36%), Positives = 115/218 (53%), Gaps = 11/218 (5%)

Query 510 PCPATLVYHADICVCSYDQTNVVDDDDNKSKQHGVCKATVALNFDNNNATDSSVNHFWVN 569

PC Y D C CS V + Q C+ + L FD++ D S N+ +V

Sbjct 2 PCAPGTNYFMDTCSCSTFAKPV-----SYQPQKPDCRPLLYLPFDSD-VRDHSGNYNYVQ 55

Query 570 NTGVTFNDGKAYFNGKSRLTIPGLSNMEFGSTVYILIKYRHSS-ANSQQTLVSNGDCQVR 628

N GVT ++G AYFNG + L IP +NM+FGS + I +Y+ + +Q ++SNGDC

Sbjct 56 NDGVTIHNGAAYFNGHTGLRIPRFANMDFGSQLMIKFRYKKETQITRKQAVISNGDCSTS 115

Query 629 QSLAVCSGKDSVDFYAETKEQISLGKTTVPTDVGAWQYALYALDNGNLL-GSVGVNKIAQ 687

SL + + F +T + TVP++ W+ ++ LD GNLL GSV + +Q

Sbjct 116 GSLYIIAADKFTSFGIKTISHRA-AAVTVPSEDTEWRDTMFYLD-GNLLSGSVNGDS-SQ 172

Query 688 PVKGALDRRQRGLVIGGGGGCDNFHGIIDDVRVYLCKP 725

+ G + R+ + IG G G NF G IDD+++YLCKP

Sbjct 173 TLAGPIQRKNCAIQIGRGTGFGNFKGYIDDLKIYLCKP 210

>[XP_022339846.1](https://www.ncbi.nlm.nih.gov/protein/XP_022339846.1?report=genbank&log$=protalign&blast_rank=19&RID=0) asparagine-rich protein-like [Crassostrea virginica]

Length=268

Score = 116 bits (290), Expect = 6e-25, Method: Compositional matrix adjust.

Identities = 76/243 (31%), Positives = 120/243 (49%), Gaps = 8/243 (3%)

Query 490 PIAGDKSGYLQFT-GVSWIRRPCPATLVYHADICVCSYDQTNVVDDDDNKSKQHGV--CK 546

PIA D Y Q G ++ PC Y+ C C+ + + S Q V C+

Sbjct 22 PIANDMKHYQQLLPGTGYVTMPCAEGTHYNERKCKCTQQEPGYPTFKE-PSVQEPVAGCQ 80

Query 547 ATVALNFDNNNATDSSVNHFWVNNTGVTFNDGKAYFNGKSRLTIPGLSNMEFGSTVYILI 606

V L+F N TDSS +VNN GV +G+A+FNG+ RL IP +N++FG T I +

Sbjct 81 PEVRLDF-KNGVTDSSGKWTYVNNQGVEVKNGEAFFNGQGRLLIPRFTNVDFGKTFVIRL 139

Query 607 KYRHSSA---NSQQTLVSNGDCQVRQSLAVCSGKDSVDFYAETKEQISLGKTTVPTDVGA 663

+YR N Q LV+NGDC S+ + + ++S+ + +T ++ S +

Sbjct 140 RYRELEKLKFNESQALVNNGDCGDFGSIQIFTKRNSIGYVVKTTKEPSHVSLQLHKPHTE 199

Query 664 WQYALYALDNGNLLGSVGVNKIAQPVKGALDRRQRGLVIGGGGGCDNFHGIIDDVRVYLC 723

W+ Y + + G + + + G+++ RQ + IG G G +NF G I + +YLC

Sbjct 200 WKDVEYVVSDEKFEGYLNGEQATKWSLGSVESRQCAIQIGFGHGYNNFRGYISMLEIYLC 259

Query 724 KPE 726

KP+

Sbjct 260 KPD 262

>[XP_009057764.1](https://www.ncbi.nlm.nih.gov/protein/XP_009057764.1?report=genbank&log$=protalign&blast_rank=20&RID=0) hypothetical protein LOTGIDRAFT_233460 [Lottia gigantea]

[ESO91711.1](https://www.ncbi.nlm.nih.gov/protein/ESO91711.1?report=genbank&log$=protalign&blast_rank=20&RID=0) hypothetical protein LOTGIDRAFT_233460 [Lottia gigantea]

Length=640

Score = 122 bits (305), Expect = 8e-25, Method: Compositional matrix adjust.

Identities = 123/432 (28%), Positives = 186/432 (43%), Gaps = 56/432 (13%)

Query 313 LCNNCVVHHGVGYAPLPGYCDAYVQCRFYGALPTAVDIRRCPSGNYWNQDKLVCDFQDNV 372

LC+ C +G+G+ P P C ++QC F I+ C G +W+QD C + V

Sbjct 244 LCDGCKEDNGIGFLPHPTDCHRFIQCIFDKDGSAEGMIKNCGMGLFWDQDDFTCKYPSQV 303

Query 373 KCTPVNNCPNHKAIPGDWAAYSIFNGANW--------TRVACPERRLYN--SVTC----G 418

C VN+ N+ PG SI N + + CP+ Y+ S C

Sbjct 304 TC--VNDKCNN---PGTTHFKSIHNCRQYYSCDDRFGAALCCPQGTSYSDTSRQCVSDNT 358

Query 419 CTDITGGFDG---NHEICTDKKAIIGDNTGFMQ-FTGNGWVRMACPATLGYNEQTCRCTD 474

C D+ G + +K+A+ G +M+ G G ++ C ++ C C+

Sbjct 359 CNDLCGTSSNPAPTAQAQCNKEAVPGLPGYYMEDVPGFGKIQRPCAVGTTFSPTKCDCST 418

Query 475 KLSPDTSISVCPNTKPIAGDKSGYLQFTGVSWIRRPCPATLVYHADICVCSYDQTNVVDD 534

+ + P P + +F W+ P S D V

Sbjct 419 FYDSNPA---APTRFPPVNN-----EFLNCDWVTTCSPTARQR-------SVDSVTV--- 460

Query 535 DDNKSKQHGVCKATVALNFDNNNATDSSVNHFWVNNTGVTFNDGKAYFNGKSRLTIPGLS 594

H CK + L F+N+ A D+S N+ WV N GV+F +G AYFNG + L +P S

Sbjct 461 -------H--CKPILDLQFEND-ARDTSGNNNWVQNVGVSFQNGWAYFNGDAILRVPRFS 510

Query 595 NMEFGSTVYILIKYR-HSSANSQQTLVSNGDCQVRQSLAVCSGKDSVDFYAETKEQISLG 653

N GST + +KYR + QQ L +N DC S+A+ + + D + E L

Sbjct 511 NFGLGSTFMVKMKYRAEAPIQQQQALFTNRDCGKPGSVAILLNRGT-DTFLMRNEASELK 569

Query 654 KTTVPT-DVGAWQYALYALDNGNLLGSV-GVNKIAQPVKGALDRRQRGLVIGGGGGCDNF 711

V D W+ +Y +DNG L G+V GV++ Q V G + + + L IG G +NF

Sbjct 570 TVDVNVQDQTQWREVIYRVDNGLLKGTVDGVSQQIQIV-GDIRKSECALQIGHGNTYENF 628

Query 712 HGIIDDVRVYLC 723

G ID V+V +C

Sbjct 629 VGYIDYVQVSMC 640

>[XP_022335717.1](https://www.ncbi.nlm.nih.gov/protein/XP_022335717.1?report=genbank&log$=protalign&blast_rank=21&RID=0) mucin-2-like [Crassostrea virginica]

Length=1573

Score = 113 bits (283), Expect = 1e-21, Method: Compositional matrix adjust.

Identities = 164/606 (27%), Positives = 257/606 (42%), Gaps = 112/606 (18%)

Query 149 QQTITPEDSEEQTTQQE---QQTTTPANSEEDTTLEQQTTTPADSEEDTTQQEEQTTTPA 205

Q++ TPE + ++ T+ E Q+ TTP + ++ T +QTT S E TTQ+ TTP

Sbjct 1034 QKSTTPEPTTQKPTKPEPTSQKPTTPEPTTQEPTTPEQTTQELTSPEPTTQEP---TTPE 1090

Query 206 DSEEDTTQQEQQTTTPADSEEEKQDSDDTDGDDNSDEDST-ETTTSTTTLAPATTTETTT 264

+ + T E T P E T + E +T E TTS + +TT E+T+

Sbjct 1091 PTTQKPTTSEPTTQKPTAPE-------PTTLKPTTPEPTTQEPTTSEPSTRKSTTPESTS 1143

Query 265 TTTTTTTTTTEVITTPEATTTTTEAEPVT---------TTTTTTTIGAITTVKPSLE-LC 314

TT TT+ TTP+ TT +P+T +T +TT P LE LC

Sbjct 1144 QKPTTPGPTTQKPTTPKPTT----QKPITPEPSVQAAVVSTKAPEAIVVTTKAPPLEDLC 1199

Query 315 NNCVVHHGVGYAPLPGYCDAYVQCRFYGALPTAVDIRRCPSGNYWNQDKLVCDFQDNVKC 374

N V +GY PG+C+ +VQC F T +R CP+G +W+QD +C + V C

Sbjct 1200 YNSVYIDSIGYNKYPGHCNKFVQC-FSNYEQTKAVLRECPAGLFWHQDYAMCKSPEKVPC 1258

Query 375 TPVNNCPNHKAIPGDWAAYSIFNGANWTRVACPERRLYNSVTCGCTDITGGFDGNHEICT 434

++C N G +A Y G C R Y S G + T C

Sbjct 1259 Y-EDHCLNL----GVYA-YKRSGG-------C---RSYYSCDVGVSVPT---------CC 1293

Query 435 DKKAIIGDNTGFMQFTGNGWVR-MACPATLGYNEQTCRCTDKLSPDTSISVCPNTKPIAG 493

K GF +F G V+ +C C+ + L S +C P

Sbjct 1294 KK--------GF-RFDGQTCVQDSSC-------NDPCQTPEDLKRRLSQQMC-RFLPDKD 1336

Query 494 DKSGYL--QFTGVSWIRRPCPATLVYHADICVCSYDQTNVVDDDDNKSKQHGVCKATVAL 551

++ GYL + +G+ + R CP V+ A C C + Q ++ + VCK +

Sbjct 1337 NRFGYLTLEHSGIRF--RACPYGTVFSARQCGCIWFQIAF------QTPRKEVCKPDFKM 1388

Query 552 NFDNNNATDSSVNH--FWVNNTGVTFNDGKAYFNGKSRLTIPGLSNMEFGSTVYILIKYR 609

NFD N+ + S ++ F+V N V +G A F G ++TI G N E G + ++++

Sbjct 1389 NFDTNSFRELSGSNMAFYVENGAV--QNGAAKFGGNGKITIWGFMNKELGHDFAVRVRFK 1446

Query 610 -HSSANSQQTLVSNGDCQVRQSLAVCSGKDSVDFYAETKEQISLGKTTVPTDVGAWQYAL 668

+ + LVSN + G +V+ + K+ + K+ +Y +

Sbjct 1447 PYQTEGGYGMLVSNCGHE---------GLPTVEISMQDKKARLIAKSVHSNAPSILEYHI 1497

Query 669 YALD--------NGN-LLGSVGVNKIAQPVKGALDRRQRGLVIGG----GGGCDNFHGII 715

+ NGN + N I++ + G ++ + IGG G G F+G+I

Sbjct 1498 LPYEWNEISYHYNGNTFTAEINGNSISEQLIGGIETSPNPMFIGGCPQPGSG---FNGLI 1554

Query 716 DDVRVY 721

D V +Y

Sbjct 1555 DYVEIY 1560

>[XP_021338925.1](https://www.ncbi.nlm.nih.gov/protein/XP_021338925.1?report=genbank&log$=protalign&blast_rank=22&RID=0) uncharacterized protein LOC110440276 [Mizuhopecten yessoensis]

[OWF37338.1](https://www.ncbi.nlm.nih.gov/protein/OWF37338.1?report=genbank&log$=protalign&blast_rank=22&RID=0) Protein PIF [Mizuhopecten yessoensis]

Length=811

Score = 112 bits (280), Expect = 2e-21, Method: Compositional matrix adjust.

Identities = 138/491 (28%), Positives = 198/491 (40%), Gaps = 91/491 (19%)

Query 270 TTTTTEVITTPEATTTTTEAEPVTTTTTTTTIGAITTVKPSLEL---------------C 314

TTTTT TT TTTT + E ++ T+ + K +++ C

Sbjct 372 TTTTTPTTTTTTPTTTTIKQEIYHASSFRKTLKIVNIRKLAIKQQKEQRKKTTGWIKGPC 431

Query 315 NNCVVHHGVGYAPLPGYCDAYVQCRFYGALPTAVDIRRCPSGNYWNQDKLVC--DFQDNV 372

++C + +GVGY P C ++QC F R CP G +++ D + C +Q N

Sbjct 432 DDCSMMNGVGYNSHPNICSKFIQCYFGPHGKMRASYRECPFGFFFDPDVMACRLSYQCNC 491

Query 373 KCTPVNNCPNHKAIPGDWAAYSIFNGANWTRVACPERRLYNSVTCGCTDITGGFDGNHEI 432

K P+ K+ P + G R +R ++ C C

Sbjct 492 KRDKCKMRPHLKSYP--------YKGIQNCRAYWLCKRDVSTARC-CAK----------- 531

Query 433 CTDKKAIIGDNTGFMQFTGNGWVRMACPATLGYNEQTCR----CTDKLSPD-TSISVCPN 487

G + R GY + C C D+ P TS+S C

Sbjct 532 ------------------GYRYRR-------GYGKNKCVKDPGCQDECPPTLTSVSKC-E 565

Query 488 TKPIAGDKSGYLQF-TGVS-WIRRPCPATLVYHADICVCSYDQTNVVDDDDNKSKQHGVC 545

T+ + + Y QF TG + W+ RPC +Y AD C C T V K K C

Sbjct 566 TRMVFSNAKVYEQFITGWNMWVPRPCAPGSLYDADKCAC----TGHVSHIPPKRK----C 617

Query 546 KATVALNFDNNNATDSSVNHFWVNNTGVTF-NDGKAYFNGKSRLTIPGLSNMEFGSTVYI 604

K V L F + D S +H +V N V G AYFNG+S+L IP SN +F V I

Sbjct 618 KPEVYLPF-TKDLKDHSGSHSYVQNYNVKRTKKGYAYFNGRSKLIIPRYSNADFKEIV-I 675

Query 605 LIKYRHSSANS-------QQTLVSNGD-CQVRQSLAVCSGKDSVDFYAETKEQISLGKTT 656

I+++ S LVSN D C SL + + S+ + A TK L

Sbjct 676 KIRFKVKKCKSVPRRRPKLMALVSNSDCCNDDASLMMVLSQFSIHYMAITKHH-ELASFH 734

Query 657 VPTDVGAWQYALYALDNGNLLGSVGVNKIAQPVKGALDRRQRGLVIGGGGGCDNFHGIID 716

+ + G W A + D NL G P G++ R G+ IG G G DNF G ID

Sbjct 735 IAKNDG-WNDAYFIHDTKNLCGRCNGAVKTAPSPGSIKRTHTGIHIGYGRGFDNFKGYID 793

Query 717 DVRVYLCKPEL 727

++++Y C+P+

Sbjct 794 EIKIYRCRPDF 804

>[AKV63183.1](https://www.ncbi.nlm.nih.gov/protein/AKV63183.1?report=genbank&log$=protalign&blast_rank=23&RID=0) P-U8, partial [Pinctada fucata]

Length=1923

Score = 113 bits (282), Expect = 2e-21, Method: Compositional matrix adjust.

Identities = 79/261 (30%), Positives = 121/261 (46%), Gaps = 16/261 (6%)

Query 472 CTDKLSPDTSISVCPNTKPIAGDKSGYLQFT-GVSWIRRPCPATLVYHADICVCSYDQTN 530

C+D P T +++ I GD Y ++ G W++ PCP Y C C+

Sbjct 1673 CSDACPPVTYEHSSCDSREITGDPKRYERYIPGQGWVKMPCPMGTHYQHSSCGCA----- 1727

Query 531 VVDDDDNKSKQHGVCKATVALNFDNNNATDSSVNHFWVNNTGVTFNDGKAYFNGKSRLTI 590

V N++ Q VC+ + L FD + D S N+ V N GV +G AYFNG+++L I

Sbjct 1728 VHVSFKNETTQ-AVCRPELYLPFDTD-LRDKSGNNLHVENHGVVIREGAAYFNGQAQLVI 1785

Query 591 PGLSNMEFGSTVYILIKYRHSSANSQQTLVSNGD-CQVRQSLAVCSGKDSVDFYAETKEQ 649

P S+ ++ V + Y + + L+SN + C+ SLA+ K S+ F+A +

Sbjct 1786 PRFSSFQYRDLVITMKFYENPPTGNLVPLMSNSNFCKSNISLALIKSKQSIHFFA----K 1841

Query 650 ISLGKTT---VPTDVGAWQYALYALDNGNLLGSVGVNKIAQPVKGALDRRQRGLVIGGGG 706

+ GK+T +P VG W Y D L GS K + G L + IG G

Sbjct 1842 LDTGKSTTFGLPMKVGKWNQVYYVHDGQILEGSSNGMKAEKSAIGKLQSTHTPIHIGFGK 1901

Query 707 GCDNFHGIIDDVRVYLCKPEL 727

G F G +D++ +Y C+P

Sbjct 1902 GRSKFKGYMDEIAIYSCRPSF 1922

>[XP_022290478.1](https://www.ncbi.nlm.nih.gov/protein/XP_022290478.1?report=genbank&log$=protalign&blast_rank=24&RID=0) LOW QUALITY PROTEIN: uncharacterized protein LOC111102114 [Crassostrea

virginica]

Length=550

Score = 108 bits (270), Expect = 1e-20, Method: Compositional matrix adjust.

Identities = 100/386 (26%), Positives = 157/386 (41%), Gaps = 66/386 (17%)

Query 351 RRCPSGNYWNQDKLVCDFQDNVKCTPVNNC--PNHKAIPG----DWAAYSIFNGANWTRV 404

R+CP G YW+Q+++ C V C P + C P+ + P + A+ + + +

Sbjct 216 RQCPFGMYWDQEEITCMPAAQVPC-PKDKCSIPSILSYPFGNMENCKAHWVCSHGKSIPM 274

Query 405 ACPERRLYNSVTCGCTDITGGFDGNHEICTDKKAIIGDNTGFMQFTGNGWVRMACPATLG 464

CP+ + Y GC G C D CP

Sbjct 275 CCPKGQAYVPFK-GCVSQPG--------CND----------------------PCPL--- 300

Query 465 YNEQTCRCTDKLSPDTSISVCPNTKPIAGDKSGYLQFT-GVSWIRRPCPATLVYHADICV 523

+Q C ++ D DK Y +F G W++ C ++ +D C

Sbjct 301 --QQRRECGKRMMWDEX------------DK--YEEFVYGYGWVKSXCEHGFIFDSDSCQ 344

Query 524 C-SYDQTNVVDDDDNKSKQHGVCKATVALNFDNNNATDSSVNHFWVNNTGV-TFNDGKAY 581

C + N +D+K K VCKA V + FDN+ D+S NH V N V +G Y

Sbjct 345 CLPLNNGNNFKMEDDKKK---VCKAVVHIPFDNS-CLDTSGNHVVVENHNVQLLGNGVGY 400

Query 582 FNGKSRLTIPGLSNMEFGSTVYILIKYRHSSANSQQTLVSNGDCQVRQ-SLAVCSGKDSV 640

F+G ++L IP M+ ST I ++Y + Q L+SNGDC+ + ++ + G+ V

Sbjct 401 FDGNAKLVIPKFPEMQSASTFVIKMRYLDVPSRHMQGLLSNGDCEREEPTMFMVKGRAGV 460

Query 641 DFYAETKEQISLGKTTVPTDVGAWQYALYALDNGNLLGSVGVNKIAQPVKGALDRRQRGL 700

+F AE+ + +P+ W + D L G + + G + GL

Sbjct 461 NFMAESAKH-KFTTFHLPSKPSCWNEXYFIHDFNTLEGYINGKSCKRNTYGPIKMAHGGL 519

Query 701 VIGGGGGCDNFHGIIDDVRVYLCKPE 726

IG G NF G +D V +Y C+ E

Sbjct 520 NIGTVKGFQNFQGFMDYVTIYYCQEE 545

>[XP_021352990.1](https://www.ncbi.nlm.nih.gov/protein/XP_021352990.1?report=genbank&log$=protalign&blast_rank=25&RID=0) uncharacterized protein LOC110450071 [Mizuhopecten yessoensis]

Length=1502

Score = 107 bits (266), Expect = 1e-19, Method: Compositional matrix adjust.

Identities = 109/442 (25%), Positives = 174/442 (39%), Gaps = 88/442 (20%)

Query 306 TVKPSLELCNNCVVHHGVGYAPLPGYCDAYVQCRFYGALPTAVDIRRCPSGNYWNQDKLV 365

T+ P++ C C + G+GY P CD +V C + ++ CP G +W+ +

Sbjct 1112 TLSPAV--CLGCTMDGGLGYIRHPSRCDQFVVCYPDQSGLFKPRVQDCPYGQFWSNIDVT 1169

Query 366 C----------DF----QDNVKCTPVNNCPNHKAIPGDW---AAYSIFNGANWTRVACPE 408

C DF D + + NC + W A +S+ CPE

Sbjct 1170 CKPSKDVNCEHDFCKKLADKFQFSHTTNCVAY------WQCQAGHSVMR-------CCPE 1216

Query 409 RRLYNSVTCGCTDITGGFDGNHEICTDKKAIIGDNTGFMQFTGNGWVRMACPATLGYNEQ 468

+++ GC +C D+ + + +QF W ++

Sbjct 1217 GKIFVQGE-GCIF--------SSVCADRCPV----SSPLQFM---W------------QE 1248

Query 469 TCRCTDKLSPDTSISVCPNTKPIAGDKSGYLQFTGVSWIRRPCPATLVYHADICVCSYDQ 528

C N +P+ D + Y + W+ C +Y +IC C

Sbjct 1249 NC----------------NKRPVTSDVTAYEKQISSGWVSEKCSVGQIYKGEICDC---- 1288

Query 529 TNVVDDDDNKSKQH-GVCKATVALNFDNNNATDSSVNHFWVNNTGVTFNDGKAYFNGKSR 587

+ N + GVCK V L FD + S N F V VT ++G A F+G S

Sbjct 1289 --IPAAMGNPTYSFKGVCKPEVFLPFDTDFRDYSGSNTF-VRTENVTLSNGAACFDGTSV 1345

Query 588 LTIPGLSNMEFGSTVYILIKYRHSSAN-SQQTLVSNGDCQVRQSLAVCSGKDSVDFYAET 646

+++P +NM+FG TV++ I+Y+ + L+ NGDC+ +L V + F +T

Sbjct 1346 ISMPKFANMDFGKTVFMNIRYKQMDLKIDTEALLYNGDCEEIPTLVVGTKPSGNSFSVKT 1405

Query 647 KEQISLGKTTVPTDV--GAWQYALYALDNGNLLGSVGVNKIAQPVKGALDRRQRGLVIGG 704

+ VP+ V W+ + NG L G G+N V G + R G+ +G

Sbjct 1406 NTG-AFQTAWVPSSVQTSDWRDVTITISNGRLEGQSGINARDIDVSGGIARSHCGIKLGW 1464

Query 705 GGGCDNFHGIIDDVRVYLCKPE 726

G NF G ID+V +Y C P

Sbjct 1465 GKKFKNFVGCIDEVSIYRCIPN 1486

>[XP_021363830.1](https://www.ncbi.nlm.nih.gov/protein/XP_021363830.1?report=genbank&log$=protalign&blast_rank=26&RID=0) protein PIF-like [Mizuhopecten yessoensis]

Length=625

Score = 103 bits (258), Expect = 6e-19, Method: Compositional matrix adjust.

Identities = 103/357 (29%), Positives = 146/357 (41%), Gaps = 58/357 (16%)

Query 311 LELCNNCVVHHGVGYAPLPGYCDAYVQCRFYGALPTAVDIRRCPSGNYWNQDKLVCDFQD 370

++ C+ C + +G G+ P CD +VQC F I+ CP GN+W+Q+ + C

Sbjct 288 IDPCDGCRMRNGAGFNRHPTDCDKFVQCYFTADGKRQHSIQECPWGNFWDQETMTCKPAH 347

Query 371 NVKCT------PVNNCPNHKAIPGDWAAYSIFNGANWTRVACPERRLYNSVTCGCTDITG 424

V C P H + D ++ N + CP + GC

Sbjct 348 MVFCLTDKCGDPYMQAHKHTS---DCRSFWYCNNRKSFPMCCPMNTSFVE-NVGCVPD-- 401

Query 425 GFDGNHEICTDKKA-----IIGDNTGFMQFTGNGWVRMACPAT-LGYNEQTCRCTDKLSP 478

+ HE C + + + N G N MA P +G+ T + DK

Sbjct 402 --NSCHEHCPPRDSGTPVRFVNRNQG-----KNDPPIMAAPVPGVGHAPPTQKECDK--- 451

Query 479 DTSISVCPNTKPIAGDKSGYLQFTGVS-WIRRPCPATLVYHADICVCSYDQTNVVDDDDN 537

K IA + QF S WIR PC Y+ C C+ V D+

Sbjct 452 ----------KEIADAPMHFEQFVARSGWIRMPCAPGTSYNQRDCQCTVISEYVKKDEG- 500

Query 538 KSKQHGVCKATVALNFDN--NNATDSSVNHFWVNNTGVTFNDGKAYFNGKSRLTIPGLSN 595

CK+ V+LNF +D SV +V N V+ G A F+GKS L +P LSN

Sbjct 501 -------CKSKVSLNFATYIEENSDKSV---YVVNENVSVAHGVAIFDGKSSLRVPQLSN 550

Query 596 MEFGSTVYILIKY-----RHSSANSQ-QTLVSNGDCQVRQSLAVCSGKDSVDFYAET 646

M+ G TV + I+Y RH A + Q L+SNGDC S+ + S+ F AET

Sbjct 551 MDLGDTVVLKIRYKDTPKRHPRATDRPQALISNGDCGNNASVLLAKDDTSIMFGAET 607

>[XP_021377870.1](https://www.ncbi.nlm.nih.gov/protein/XP_021377870.1?report=genbank&log$=protalign&blast_rank=27&RID=0) uncharacterized protein LOC110465975 [Mizuhopecten yessoensis]

[OWF38728.1](https://www.ncbi.nlm.nih.gov/protein/OWF38728.1?report=genbank&log$=protalign&blast_rank=27&RID=0) Protein PIF [Mizuhopecten yessoensis]

Length=698

Score = 103 bits (257), Expect = 7e-19, Method: Compositional matrix adjust.

Identities = 113/437 (26%), Positives = 176/437 (40%), Gaps = 83/437 (19%)

Query 312 ELCNNCVVHHGVGYAPLPGYCDAYVQCRFYGALPTAVDIRRCPSGNYWNQDKLVCDFQDN 371

+LC NC++ GY P PG C Y+ G +++CP G++W+ L C + +

Sbjct 312 DLCPNCIIGRNWGYMPYPGDCTRYISMFPDGRGGRVSSVQKCPFGSWWDSYSLSCRYPVD 371

Query 372 VKCTPVNNCP----NHKAIP------GDWAAYSIFNGANWTRVACP---ERRLYNSVTCG 418

V C N+ P ++ A P G W Y I NG + CP +R + N

Sbjct 372 VTC---NDDPCRGVSYGAFPMADVCSGFW--YCI-NGKAYPS-CCPKQNQRFIGNGPQLK 424

Query 419 CTDITGGFDGNHEICTDKKAIIGDNTGFMQFTGNGWVRMACPATLGYNEQTCRCTDKLSP 478

C+D ++ C D + N R+ P

Sbjct 425 CSD--------NDFCPDSCPPVDSN------------RLVGP------------------ 446

Query 479 DTSISVCPNTKPIAGDKSGYLQFT-GVSWIRRPCPATLVYHADICVCSYDQTNVVDDDDN 537

+VC + P A D++ YL++ G I R C + ++ C C+ +DD +

Sbjct 447 ----TVCA-SYPHASDRTKYLEYAPGSGNITRDCATGTYFVSNDCACTG-----IDDLPS 496

Query 538 KSKQHGVCKATVALNFDNNNATDSSVNHFWVNNTGVTFNDGKAYFNGKSRLTIPGLSNME 597

+S Q C V NFD+N D S N V + +A F+G + + SN +

Sbjct 497 QSLQ--TCNPEVYFNFDDNFG-DMSGNEIAAGLENVELDTKEAKFSGDGMINMWRFSNSD 553

Query 598 FGSTVYILIKYRHSSANSQ---QTLVSNGDC------QVRQSLAVCSGKDSVDFYAETKE 648

F ++ I K+R ++ S Q LV+N C + +++V G D Y +T+

Sbjct 554 FRRSLVITFKFRPENSGSPGPIQALVTN--CIFSDQEEASLAISVVPGSDPSVLYFKTQT 611

Query 649 QISLGKTTVPTDVGAWQYALYALDNGNLLGSVGVNKIAQPVKGALDRRQRGLVIGGGGGC 708

+ VP D +Y D L G V + G + RRQ G+VIG G

Sbjct 612 TRGTAELFVPYDPTVMSSGVYIYDGVTLTGRVNNEVRTASLTGDILRRQSGIVIGAGSMM 671

Query 709 DNFHGIIDDVRVYLCKP 725

N+ G IDD ++Y C P

Sbjct 672 ANYRGRIDDFKLYTCFP 688

>[XP_034298995.1](https://www.ncbi.nlm.nih.gov/protein/XP_034298995.1?report=genbank&log$=protalign&blast_rank=28&RID=0) mucin-2-like [Crassostrea gigas]

[XP_034298996.1](https://www.ncbi.nlm.nih.gov/protein/XP_034298996.1?report=genbank&log$=protalign&blast_rank=28&RID=0) mucin-2-like [Crassostrea gigas]

Length=2199

Score = 104 bits (259), Expect = 9e-19, Method: Compositional matrix adjust.

Identities = 110/438 (25%), Positives = 176/438 (40%), Gaps = 89/438 (20%)

Query 306 TVKPSLELCNNCVVHHGVGYAPLPGYCDAYVQCRFYGALPTAVDIRRCPSGNYWNQDKLV 365

T P LC N V +GY PG C+ +VQC + T +R CP+G +W+QD +

Sbjct 1819 TAAPLENLCVNSVYIDSIGYNKYPGSCNKFVQC-YNNYQNTKAVLRECPAGLFWHQDHAM 1877

Query 366 CDFQDNVKCTPVNNCPNHKAIPGDWAAYSIFNGANWTRVACPERRLYNSVTCGCTDIT-- 423

C D V C ++C N AY G C R Y S G + T

Sbjct 1878 CKSPDKVPCF-EDHCLNL-----GVDAYKRSGG-------C---RSYFSCEYGVSVPTCC 1921

Query 424 -GGFDGNHEICTDKKAIIGDNT-GFMQFTGNGWVRMACPATLGYNEQTCRCTDKLSPDTS 481

GF D KA + D++ + T N R ++Q CR PD

Sbjct 1922 KKGFRF------DGKACVKDSSCNDICVTPNDLKR-------KLSQQMCR----FLPD-- 1962

Query 482 ISVCPNTKPIAGDKSGY--LQFTGVSWIRRPCPATLVYHADICVCSYDQTNVVDDDDNKS 539

++ GY L+ +G+ + R CP + A C C++ Q +

Sbjct 1963 ----------KNNRFGYLTLEHSGIRF--RACPYGTEFSARQCGCTWIQFTAYQNP---- 2006

Query 540 KQHGVCKATVALNFDNNNATDSSVNH--FWVNNTGVTFNDGKAYFNGKSRLTIPGLSNME 597

+ VCK +NFD N+ + S ++ F+V N +G A F G ++TI G N E

Sbjct 2007 -RKEVCKPDFKMNFDTNSFRELSGSNMAFYVEN--AVAQNGAAKFRGNGKITIWGFMNKE 2063

Query 598 FGSTVYILIKYR-HSSANSQQTLVSNGDCQVRQSLAVCSGKDSVDFYAETKEQISLGKTT 656

G + I+++ ++ LVSN + G +V+ + K+ + K+

Sbjct 2064 LGHDFAVRIRFKPFNTEGGYGMLVSNCGHE---------GLPTVEISMQDKKARLIAKSM 2114

Query 657 VPTDVGAWQYALYALD--------NGN-LLGSVGVNKIAQPVKGALDRRQRGLVIGG--- 704

+ +Y + + NGN + N I++ + G ++ + IGG

Sbjct 2115 HSSSPSVLEYHIVPYEWNEISYHYNGNTFTAEINGNSISEQLIGGIETSPNPMFIGGCPK 2174

Query 705 -GGGCDNFHGIIDDVRVY 721

G G F+G+ID+V +Y

Sbjct 2175 PGSG---FNGLIDNVEIY 2189

>[XP_011437524.2](https://www.ncbi.nlm.nih.gov/protein/XP_011437524.2?report=genbank&log$=protalign&blast_rank=29&RID=0) uncharacterized protein LOC105335381 isoform X2 [Crassostrea

gigas]

Length=2408

Score = 99.4 bits (246), Expect = 3e-17, Method: Composition-based stats.

Identities = 73/226 (32%), Positives = 112/226 (50%), Gaps = 13/226 (6%)

Query 506 WIRRPCPATLVYHADICVCSYDQTNVVDDDDNKSKQHGVCKATVALNFDNNNATDSSV-N 564

W+ + C +H ++C C V D +K VC V L F +N +SS

Sbjct 2187 WMLQECSPGTEFHPEVCDC-----RVSGDPAALNKIKHVCSPDVYLPFTHNLKDESSSRT 2241

Query 565 HFWVNNTGVTFNDGKAYFNGKSRLTIPGLSNMEFGSTVYILIKYRH-SSANSQQTLVSNG 623

H +N + G A F G+S L +P +NME GS +Y+ + YRH SS+ + LV NG

Sbjct 2242 HVRADNVSLA-TSGSACFIGRSALAMPKFANMELGSYLYVKLTYRHMSSSPKNEVLVYNG 2300

Query 624 DCQVRQSLAVCSGKDS--VDFYAETKEQISLG-KTTVPTDVGAWQYALYALDNGNLLGSV 680

DC+ + SL + S D V + EQ +L K++VP W+ +DNG++ +

Sbjct 2301 DCERKPSLILGSTADGNFVSVVTTSGEQHTLHVKSSVPAS--EWRTVSLVIDNGHIRVTS 2358

Query 681 GVNKIAQPVKGALDRRQRGLVIGGGGGCDNFHGIIDDVRVYLCKPE 726

+ G L+R GL +G G G +NF+G ID+ +Y C+P+

Sbjct 2359 DSQTEQKNTFGVLERAPCGLKLGWGEGYENFNGCIDEFTLYRCRPD 2404

>[XP_011437523.2](https://www.ncbi.nlm.nih.gov/protein/XP_011437523.2?report=genbank&log$=protalign&blast_rank=30&RID=0) uncharacterized protein LOC105335381 isoform X1 [Crassostrea

gigas]

Length=2416

Score = 99.4 bits (246), Expect = 3e-17, Method: Composition-based stats.

Identities = 73/226 (32%), Positives = 112/226 (50%), Gaps = 13/226 (6%)

Query 506 WIRRPCPATLVYHADICVCSYDQTNVVDDDDNKSKQHGVCKATVALNFDNNNATDSSV-N 564

W+ + C +H ++C C V D +K VC V L F +N +SS

Sbjct 2195 WMLQECSPGTEFHPEVCDC-----RVSGDPAALNKIKHVCSPDVYLPFTHNLKDESSSRT 2249

Query 565 HFWVNNTGVTFNDGKAYFNGKSRLTIPGLSNMEFGSTVYILIKYRH-SSANSQQTLVSNG 623

H +N + G A F G+S L +P +NME GS +Y+ + YRH SS+ + LV NG

Sbjct 2250 HVRADNVSLA-TSGSACFIGRSALAMPKFANMELGSYLYVKLTYRHMSSSPKNEVLVYNG 2308

Query 624 DCQVRQSLAVCSGKDS--VDFYAETKEQISLG-KTTVPTDVGAWQYALYALDNGNLLGSV 680

DC+ + SL + S D V + EQ +L K++VP W+ +DNG++ +

Sbjct 2309 DCERKPSLILGSTADGNFVSVVTTSGEQHTLHVKSSVPAS--EWRTVSLVIDNGHIRVTS 2366

Query 681 GVNKIAQPVKGALDRRQRGLVIGGGGGCDNFHGIIDDVRVYLCKPE 726

+ G L+R GL +G G G +NF+G ID+ +Y C+P+

Sbjct 2367 DSQTEQKNTFGVLERAPCGLKLGWGEGYENFNGCIDEFTLYRCRPD 2412

>[OWF55652.1](https://www.ncbi.nlm.nih.gov/protein/OWF55652.1?report=genbank&log$=protalign&blast_rank=31&RID=0) Protein PIF [Mizuhopecten yessoensis]

Length=582

Score = 95.5 bits (236), Expect = 2e-16, Method: Compositional matrix adjust.

Identities = 100/355 (28%), Positives = 141/355 (40%), Gaps = 83/355 (23%)

Query 308 KPSLELCNNCVVHHGVGYAPLPGYCDAYVQCRFYGALPTAVDIRRCPSGNYWNQDKLVCD 367

KP ++ C+ C + +G G+ P CD +VQC F I+ CP GN+W+

Sbjct 277 KP-IDPCDGCRMRNGAGFNRHPTDCDKFVQCYFTADGKRQHSIQECPWGNFWD------- 328

Query 368 FQDNVKCTPVNNCPNHKAIPGDWAAYSIFNGANWTRVACPERRLYNSVTCGCTDITGGFD 427

Q+ + C P A+ +F +T C D +

Sbjct 329 -QETMTCKP---------------AHMVF-----------------CLTDKCGD---PYM 352

Query 428 GNHEICTDKKAIIGDNTGFMQFTGNGWVRMACPATLGYNEQTCRCTDKLSPDTSIS-VCP 486

H+ +D ++ F M CP + E PD S CP

Sbjct 353 QAHKHTSDCRS-------FWYCNNRKSFPMCCPMNTSFVENV-----GCVPDNSCHEHCP 400

Query 487 ------NTKPIAGDKSGYLQFTGVS-WIRRPCPATLVYHADICVCSYDQTNVVDDDDNKS 539

+ K IA + QF S WIR PC Y+ C C+ V D+

Sbjct 401 PRDSECDKKEIADAPMHFEQFVARSGWIRMPCAPGTSYNQRDCQCTVISEYVKKDEG--- 457

Query 540 KQHGVCKATVALNFDN--NNATDSSVNHFWVNNTGVTFNDGKAYFNGKSRLTIPGLSNME 597

CK+ V+LNF +D SV +V N V+ G A F+GKS L +P LSNM+

Sbjct 458 -----CKSKVSLNFATYIEENSDKSV---YVVNENVSVAHGVAIFDGKSSLRVPQLSNMD 509

Query 598 FGSTVYILIKY-----RHSSANSQ-QTLVSNGDCQVRQSLAVCSGKDSVDFYAET 646

G TV + I+Y RH A + Q L+SNGDC S+ + S+ F AET

Sbjct 510 LGDTVVLKIRYKDTPKRHPRATDRPQALISNGDCGNNASVLLAKDDTSIMFGAET 564

>[CAC5411652.1](https://www.ncbi.nlm.nih.gov/protein/CAC5411652.1?report=genbank&log$=protalign&blast_rank=32&RID=0) Protein PIF [Mytilus coruscus]

Length=1907

Score = 82.0 bits (201), Expect = 7e-12, Method: Compositional matrix adjust.

Identities = 88/325 (27%), Positives = 128/325 (39%), Gaps = 80/325 (25%)

Query 303 AITTVKPSLELCNNCVVHHGVGYAPLPGYCDAYVQCRFYGALPTAVDIRRCPSGNYWNQD 362

++ KP +LC N + GVGY P CD ++QC F G T ++ C G YW+Q

Sbjct 912 SLLRKKPE-DLCRNSKMIRGVGYKVHPTDCDKFIQCYFDGFGNTRASVKSCSFGEYWDQY 970

Query 363 KLVC-DFQDNVKCTPVNNCPNH---------KAIPGDWAAY-SIFNGANWTRVACPERRL 411

+ C D Q + NCPN +A +Y G ++ + CPE+

Sbjct 971 NVACVDAQ-------IANCPNDPCKAPALFTRASKNSCRSYWGCHKGQSFVK-CCPEKMS 1022

Query 412 YN-SVTC----GCTDITGGFDGNHEICTDKKAIIGDNTGFMQFTGNG-WVRMAC-PATLG 464

Y C C +G DKK + G + Q+ +G WV M C P TL

Sbjct 1023 YQPRKGCVPDPDCVRECPPEEGPEYGTCDKKPLFGKPYLYKQWIESGHWVEMLCAPGTL- 1081

Query 465 YNEQTCRCTDKLSPDTSISVCPNTKPIAGDKSGYLQFTGVSWIRRPCPATLVYHADICVC 524

++E C CT + + TS+ V + IA + C L + C

Sbjct 1082 FDENDCGCTMRTTHMTSLGV----RAIA---------------KETCKPEL--YLPFCQG 1120

Query 525 SYDQTNVVDDDDNKSKQHGVCKATVALNFDNNNATDSSVNHFWVNNTGVTFNDGKAYFNG 584

D +N K T+ N D+ V +GKAYF+G

Sbjct 1121 FKDWSN---------------KNTLVQNEDDK----------------VVVVNGKAYFDG 1149

Query 585 KSRLTIPGLSNMEFGSTVYILIKYR 609

KS L IP S +G ++YI I+Y+

Sbjct 1150 KSSLFIPRFSGAHYGESIYIKIRYK 1174

>[XP_033752829.1](https://www.ncbi.nlm.nih.gov/protein/XP_033752829.1?report=genbank&log$=protalign&blast_rank=33&RID=0) protein PIF-like [Pecten maximus]

Length=639

Score = 79.0 bits (193), Expect = 4e-11, Method: Compositional matrix adjust.

Identities = 68/249 (27%), Positives = 104/249 (42%), Gaps = 27/249 (11%)

Query 487 NTKPIAGDKSGYLQFT-GVSWIRRPCPATLVYHADICVCSYDQTNVVDDDDNKSKQHGVC 545

+T + G+ Y QF G W+ RPC Y+ IC C ++ + + C

Sbjct 407 DTMMVVGNPKVYKQFVNGFGWMERPCAPGTHYNPLICGCQNYESLIPGRE---------C 457

Query 546 KATVALNF--DNNNATDSSVNHFWVNNTGVTFNDGKAYFNGKSRLTIPGLSNMEFGSTVY 603

K V + F D N D S+ H N + N+ A+FNG S+L IP +NMEF +

Sbjct 458 KPVVNIPFEGDLKNHADGSI-HVENENVKLIPNN-YAFFNGSSKLYIPFFANMEFYTDFV 515

Query 604 ILIKYRHSSANSQQTLVSNGDCQVRQSLAVCSGKDSVDFY------AETKEQISLGKTTV 657

+++K+ Q L+SN DC + K S + Y AE + LG

Sbjct 516 VIMKFLPVKPGV-QALLSNSDC-CSNGATINILKTSRNVYFRAKADAEQSASVRLGLLH- 572

Query 658 PTDVGAWQYALYALDNGNLLGSVGVNKIAQPVKGALDRRQRGLVIGGGGGCDNFHGIIDD 717

G W + D+ L + G + G+ IG G D F G +D

Sbjct 573 ----GNWNKIYFIHDHYRLEARSNERRSEADAFGPIRTSSTGISIGYSKGFDGFVGFMDY 628

Query 718 VRVYLCKPE 726

V++Y+C+P+

Sbjct 629 VKIYMCRPD 637

>[XP_005183079.1](https://www.ncbi.nlm.nih.gov/protein/XP_005183079.1?report=genbank&log$=protalign&blast_rank=34&RID=0) PREDICTED: proteoglycan 4-like [Musca domestica]

Length=444

Score = 63.9 bits (154), Expect = 1e-06, Method: Compositional matrix adjust.

Identities = 47/112 (42%), Positives = 64/112 (57%), Gaps = 2/112 (2%)

Query 130 VTQEQQTTPAESVEQV-KQEQQTITPEDSEEQTTQQEQQTTTPANSEEDTT-LEQQTTTP 187

V +EQ TTP + K E+Q TP+ EE T++ E+Q TTP EE T+ EQQ TTP

Sbjct 96 VPEEQPTTPKAPEDTTSKPEEQPTTPKAPEETTSKPEEQPTTPKAPEETTSKPEQQPTTP 155

Query 188 ADSEEDTTQQEEQTTTPADSEEDTTQQEQQTTTPADSEEEKQDSDDTDGDDN 239

E+ T++ EEQ TTP E+ TT+ E+Q TTP E+ ++ DN

Sbjct 156 KAPEDTTSKPEEQPTTPKAPEDTTTKPEEQPTTPKAPEDSTTKPEEQPTKDN 207

Lambda K H a alpha

0.311 0.126 0.375 0.792 4.96

Gapped

Lambda K H a alpha sigma

0.267 0.0410 0.140 1.90 42.6 43.6

Effective search space used: 34364252109600

**Query=** R27073283 TransAbyss assembly 2 (filtered min reads 10, dedupe95)

len=1878 num_reads=1258727 avg_cov=63568.7 contig_cov=100.0%

(contig_1265 from old CLC assemly 9) cds start = 227 cds stop = 1453

strand = - protein length = 409 strand = +

Length=409

Score E

Sequences producing significant alignments: (Bits) Value

[BBD49796.1](https://www.ncbi.nlm.nih.gov/protein/BBD49796.1?report=genbank&log$=prottop&blast_rank=1&RID=) extension-2-like [Euhadra quaesita] [294](#BBD49796.1) 1e-95

[RUS70679.1](https://www.ncbi.nlm.nih.gov/protein/RUS70679.1?report=genbank&log$=prottop&blast_rank=2&RID=) hypothetical protein EGW08_021555 [Elysia chlorotica] [144](#RUS70679.1) 2e-33

[XP_035828554.1](https://www.ncbi.nlm.nih.gov/protein/XP_035828554.1?report=genbank&log$=prottop&blast_rank=3&RID=) uncharacterized protein LOC118478649 [Aplysia cali... [115](#XP_035828554.1) 1e-25

>[BBD49796.1](https://www.ncbi.nlm.nih.gov/protein/BBD49796.1?report=genbank&log$=protalign&blast_rank=1&RID=0) extension-2-like, partial [Euhadra quaesita]

Length=199

Score = 294 bits (753), Expect = 1e-95, Method: Compositional matrix adjust.

Identities = 149/199 (75%), Positives = 163/199 (82%), Gaps = 0/199 (0%)

Query 211 KSRVAQEYRNIGRNSYIEDRVSFGSGMYGDSYPGKKKREVENYIAYNEAGMGPEALAEGK 270

KSRVAQEYRNIGRNSYI DRVSFGSGMYGDSYP K+KR+ +IAYNEA M P A

Sbjct 1 KSRVAQEYRNIGRNSYIGDRVSFGSGMYGDSYPVKRKRKSATHIAYNEAEMRPAASDGSN 60

Query 271 LRKKRFMFVTEPSEFPATPSLFSIFQGTALDPNTGKRLEFGGDVMKRAFGSTLMSFLPPG 330

LR+KRF+F EP+E TPSLFSI QGTALDPN+GKRLEFGGDVMKRAFGSTLMSFLPPG

Sbjct 61 LRRKRFLFNMEPTEVLQTPSLFSIHQGTALDPNSGKRLEFGGDVMKRAFGSTLMSFLPPG 120

Query 331 FQPPPGREPGDFIKSVAPLLLYNSASPGSAKKQNGNGLFGDTLPGKKKRKRSPDASYKDV 390

FQPPPG EPGDFIK+V+PLLL N+ + K ++ NGLFGD LPGK+KRKRSPD S KDV

Sbjct 121 FQPPPGTEPGDFIKTVSPLLLANNGGGVNIKTRHRNGLFGDALPGKRKRKRSPDDSSKDV 180

Query 391 FRFGDVPVDLFGIKGLARF 409

R GDV VDLFG KGL RF

Sbjct 181 VRLGDVAVDLFGRKGLDRF 199

>[RUS70679.1](https://www.ncbi.nlm.nih.gov/protein/RUS70679.1?report=genbank&log$=protalign&blast_rank=2&RID=0) hypothetical protein EGW08_021555 [Elysia chlorotica]

Length=971

Score = 144 bits (363), Expect = 2e-33, Method: Compositional matrix adjust.

Identities = 126/435 (29%), Positives = 183/435 (42%), Gaps = 90/435 (21%)

Query 29 GRTVEERACDELRRELAMLLRLVYDKKAWPNNFKKLLPHWVESHMPASCGKRRSYGKHIS 88

G + CD++R+E A + Y K + +L WVES +P C K G +

Sbjct 32 GAAAALKTCDQIRQETAQAFQSTYGPKVPAHLINAMLGLWVESFLPHRCLKTLDVGGPFN 91

Query 89 RPSMDAKGIYAMSSKIRHTREVVDGNFENEVHRLKRSILTADDDELKAILRGRRNTHRGH 148

PS D+ + + + + V + +EV ++ T E +R +R

Sbjct 92 -PSSDSVNVGPLGT-VGGPSTVYTTSARDEVGGARQKRSTTASKEASPHVRAKR------ 143

Query 149 AANDKDGSAVLYRHKRFIVPGSSVPVPDFTSMSLGSANPGPGREISESAQMMSNLLMLTK 208

+ PG SM++ PG +E+SESAQMM LL+ +K

Sbjct 144 ----------FLSDLSMMFPG--------MSMAMPGGQPGAQKEVSESAQMMKTLLLFSK 185

Query 209 LQKSRVAQEYRNI--GRNSYIEDRVSFGSGMYGDSYPGKKKREVENYIAYNEAGMGPEAL 266

+QK R Q + G+ Y D+ GSG+YGDSY GK+KR + A E +++

Sbjct 186 MQKMRAMQSAKQYAAGQKVYSGDKYDSGSGIYGDSYSGKRKRRSVD--AQTEPTSVTDSV 243

Query 267 AEGKL-----RKKRFMFVTEPSEFPAT---PSLFSIFQGTALDPNTGKRLEFGGDVMKRA 318

A G RKKRF+F P A PS+ SI GTA DPN+GK++EFGGD MK

Sbjct 244 ASGSSKEPLSRKKRFLFGDAPDAPAAGPAMPSMLSILMGTARDPNSGKKVEFGGDYMKNM 303

Query 319 FGSTLMSFLPP-GFQPPPGREPGDFIKSVAPLLLYN------------------------ 353

S+ M+ +P PG+EP D +K++AP+ ++

Sbjct 304 MRSSFMAAMPHLAGNTAPGKEPPDVVKNLAPIWYFSRMQRAMMPRRAAPMMQGYESFQMM 363

Query 354 -SASPGSAKKQNGNGLFGDTLPG------------------------KKKRKRSPDASYK 388

ASP S K +N +G +GD PG ++ KR

Sbjct 364 RRASPASIKSRNRDGYYGDHFPGMMGKRRRRSADSRSVGLDSDHVTPSRRVKRQSGEGSN 423

Query 389 DVFRFGDV--PVDLF 401

DV GDV P D F

Sbjct 424 DVITIGDVSIPTDFF 438

>[XP_035828554.1](https://www.ncbi.nlm.nih.gov/protein/XP_035828554.1?report=genbank&log$=protalign&blast_rank=3&RID=0) uncharacterized protein LOC118478649 [Aplysia californica]

Length=271

Score = 115 bits (288), Expect = 1e-25, Method: Compositional matrix adjust.

Identities = 88/202 (44%), Positives = 116/202 (57%), Gaps = 27/202 (13%)

Query 158 VLYRHKRFIVPGSSVPVPDFTSMSLGSA-NPGPGREISESAQMMSNLLMLTKLQKSRVAQ 216

+L R KRF+ M+LG GP +E+SESAQMM+ ++ML K Q S+ +

Sbjct 86 LLSREKRFL-----------NGMTLGGPPTGGPKKEVSESAQMMNMMMMLAKFQSSQAHR 134

Query 217 EYRNIGRNSYI-EDRVSFGSGMYGDSYPG-KKKREVENYIAYNEAGMGPEALAEGKL--R 272

YR ++S I +RVSFGSGMYGD Y G + KRE A A E ++G R

Sbjct 135 TYRKPPQDSSIYGERVSFGSGMYGDQYAGYRGKREAVGPAAEGGAN---EVTSDGAALSR 191

Query 273 KKRFMF--VTEPSEFPATPSL---FSIFQGTALDPNTGKRLEFGGDVMKRAFGSTLMSFL 327

KKRF++ EP E PA + I GTA DPN GK++EFGGD MKR S+ M+

Sbjct 192 KKRFLYGAGAEPGEGPAVNPMNQFMGIMMGTARDPN-GKKIEFGGDYMKRMMRSSFMAST 250

Query 328 PPGFQPPPGREPGDFIKSVAPL 349

G Q PPG+EP DF+K++AP+

Sbjct 251 --GVQTPPGKEPSDFVKNMAPM 270

Lambda K H a alpha

0.318 0.136 0.401 0.792 4.96

Gapped

Lambda K H a alpha sigma

0.267 0.0410 0.140 1.90 42.6 43.6

Effective search space used: 15624495356928

**Query=** R27072837 TransAbyss assembly 2 (filtered min reads 10, dedupe95)

len=2379 num_reads=1735589 avg_cov=69372.3 contig_cov=100.0%

(contig_123 from old CLC assemly 9) cds start = 623 cds stop = 1261

strand = - protein length = 213 strand = +

Length=213

Score E

Sequences producing significant alignments: (Bits) Value

[BBD49810.1](https://www.ncbi.nlm.nih.gov/protein/BBD49810.1?report=genbank&log$=prottop&blast_rank=1&RID=) extensin-like [Euhadra quaesita] [212](#BBD49810.1) 2e-66

[XP_013061848.1](https://www.ncbi.nlm.nih.gov/protein/XP_013061848.1?report=genbank&log$=prottop&blast_rank=2&RID=) PREDICTED: extensin-like isoform X4 [Biomphalaria ... [120](#XP_013061848.1) 9e-31

[XP_013061846.1](https://www.ncbi.nlm.nih.gov/protein/XP_013061846.1?report=genbank&log$=prottop&blast_rank=3&RID=) PREDICTED: extensin-like isoform X2 [Biomphalaria ... [120](#XP_013061846.1) 2e-30

[XP_013061847.1](https://www.ncbi.nlm.nih.gov/protein/XP_013061847.1?report=genbank&log$=prottop&blast_rank=4&RID=) PREDICTED: formin-like protein 2 isoform X3 [Biomp... [97.8](#XP_013061847.1) 1e-21

[XP_013061844.1](https://www.ncbi.nlm.nih.gov/protein/XP_013061844.1?report=genbank&log$=prottop&blast_rank=5&RID=) PREDICTED: extensin-like isoform X1 [Biomphalaria ... [97.4](#XP_013061844.1) 2e-21

>[BBD49810.1](https://www.ncbi.nlm.nih.gov/protein/BBD49810.1?report=genbank&log$=protalign&blast_rank=1&RID=0) extensin-like, partial [Euhadra quaesita]

Length=182

Score = 212 bits (539), Expect = 2e-66, Method: Compositional matrix adjust.

Identities = 124/144 (86%), Positives = 129/144 (90%), Gaps = 6/144 (4%)

Query 70 NDGLFMDLPHAGGAHYGGGAPYGGGPQYGGGPQYGGGPYMGPPVMRSYHYCPPGPTTADH 129

NDG+FMD+P+AGG YG GA YGGG YGGGPYMGPPVMRSYHYCPPGPTTADH

Sbjct 45 NDGIFMDMPNAGGGAYGAGA------PYGGGAPYGGGPYMGPPVMRSYHYCPPGPTTADH 98

Query 130 CKDQKLQEALYFPDGTPRYNWVPPKNPWDTSLPDTVKETAKNILMMKVNSRPSRIPTPKE 189

CKDQKLQEALYFPDGTPRYNWVPP+NPWDTSL DTVKETAKNILMMKVNSRPSR PTPKE

Sbjct 99 CKDQKLQEALYFPDGTPRYNWVPPRNPWDTSLLDTVKETAKNILMMKVNSRPSRTPTPKE 158

Query 190 WELMSLLGDPKEQGPAANNPFAGR 213

WELMSLLGDPKEQGPA +NPFAGR

Sbjct 159 WELMSLLGDPKEQGPAGSNPFAGR 182

>[XP_013061848.1](https://www.ncbi.nlm.nih.gov/protein/XP_013061848.1?report=genbank&log$=protalign&blast_rank=2&RID=0) PREDICTED: extensin-like isoform X4 [Biomphalaria glabrata]

Length=152

Score = 120 bits (301), Expect = 9e-31, Method: Compositional matrix adjust.

Identities = 54/64 (84%), Positives = 58/64 (91%), Gaps = 0/64 (0%)

Query 118 HYCPPGPTTADHCKDQKLQEALYFPDGTPRYNWVPPKNPWDTSLPDTVKETAKNILMMKV 177

YCPPGPT+ D CKDQKLQEALY PDG PRYNWVPP+NPWDTSLPDTVK+TA N+LMMKV

Sbjct 82 RYCPPGPTSNDACKDQKLQEALYHPDGRPRYNWVPPRNPWDTSLPDTVKDTALNVLMMKV 141

Query 178 NSRP 181

NSRP

Sbjct 142 NSRP 145

>[XP_013061846.1](https://www.ncbi.nlm.nih.gov/protein/XP_013061846.1?report=genbank&log$=protalign&blast_rank=3&RID=0) PREDICTED: extensin-like isoform X2 [Biomphalaria glabrata]

Length=174

Score = 120 bits (301), Expect = 2e-30, Method: Compositional matrix adjust.

Identities = 54/64 (84%), Positives = 58/64 (91%), Gaps = 0/64 (0%)

Query 118 HYCPPGPTTADHCKDQKLQEALYFPDGTPRYNWVPPKNPWDTSLPDTVKETAKNILMMKV 177

YCPPGPT+ D CKDQKLQEALY PDG PRYNWVPP+NPWDTSLPDTVK+TA N+LMMKV

Sbjct 104 RYCPPGPTSNDACKDQKLQEALYHPDGRPRYNWVPPRNPWDTSLPDTVKDTALNVLMMKV 163

Query 178 NSRP 181

NSRP

Sbjct 164 NSRP 167

>[XP_013061847.1](https://www.ncbi.nlm.nih.gov/protein/XP_013061847.1?report=genbank&log$=protalign&blast_rank=4&RID=0) PREDICTED: formin-like protein 2 isoform X3 [Biomphalaria glabrata]

Length=169

Score = 97.8 bits (242), Expect = 1e-21, Method: Compositional matrix adjust.

Identities = 69/135 (51%), Positives = 77/135 (57%), Gaps = 23/135 (17%)

Query 47 PSSPFLPPPMQMAPPGPPQPRTVNDGLFMDLPHAGGAHYGGGAPYGGGPQYGGGPQYGGG 106

P P P G+ MDLP+ GG + GG Y

Sbjct 51 PPQ---PFQPGPPMAPPAPMPGPAGGVLMDLPNTGGGYAGGYPGYAPP------------ 95

Query 107 PYMGPPVMRSYHYCPPGPTTADHCKDQKLQEALYFPDGTPRYNWVPPKNPWDTSLPDTVK 166

+MR YCPPGPT+ D CKDQKLQEALY PDG PRYNWVPP+NPWDTSLPDTVK

Sbjct 96 -----VMMR---YCPPGPTSNDACKDQKLQEALYHPDGRPRYNWVPPRNPWDTSLPDTVK 147

Query 167 ETAKNILMMKVNSRP 181

+TA N+LMMKVNSRP

Sbjct 148 DTALNVLMMKVNSRP 162

>[XP_013061844.1](https://www.ncbi.nlm.nih.gov/protein/XP_013061844.1?report=genbank&log$=protalign&blast_rank=5&RID=0) PREDICTED: extensin-like isoform X1 [Biomphalaria glabrata]

Length=191

Score = 97.4 bits (241), Expect = 2e-21, Method: Compositional matrix adjust.

Identities = 69/135 (51%), Positives = 76/135 (56%), Gaps = 23/135 (17%)

Query 47 PSSPFLPPPMQMAPPGPPQPRTVNDGLFMDLPHAGGAHYGGGAPYGGGPQYGGGPQYGGG 106

P P P G+ MDLP+ GG + GG Y

Sbjct 73 PPQ---PFQPGPPMAPPAPMPGPAGGVLMDLPNTGGGYAGGYPGYAPP------------ 117

Query 107 PYMGPPVMRSYHYCPPGPTTADHCKDQKLQEALYFPDGTPRYNWVPPKNPWDTSLPDTVK 166

VM YCPPGPT+ D CKDQKLQEALY PDG PRYNWVPP+NPWDTSLPDTVK

Sbjct 118 ------VM--MRYCPPGPTSNDACKDQKLQEALYHPDGRPRYNWVPPRNPWDTSLPDTVK 169

Query 167 ETAKNILMMKVNSRP 181

+TA N+LMMKVNSRP

Sbjct 170 DTALNVLMMKVNSRP 184

Lambda K H a alpha

0.315 0.140 0.461 0.792 4.96

Gapped

Lambda K H a alpha sigma

0.267 0.0410 0.140 1.90 42.6 43.6

Effective search space used: 4003805282880

**Query=** R27075188 TransAbyss assembly 2 (filtered min reads 10, dedupe95)

len=1827 num_reads=212895 avg_cov=10763.9 contig_cov=100.0%

(contig_7508 from old CLC assemly 9) cds start = 390 cds stop = 1112

strand = + protein length = 241 strand = +

Length=241

Score E

Sequences producing significant alignments: (Bits) Value

[RUS89244.1](https://www.ncbi.nlm.nih.gov/protein/RUS89244.1?report=genbank&log$=prottop&blast_rank=1&RID=) hypothetical protein EGW08_002987 [Elysia chlorotica] [292](#RUS89244.1) 2e-96

[XP_013085779.1](https://www.ncbi.nlm.nih.gov/protein/XP_013085779.1?report=genbank&log$=prottop&blast_rank=2&RID=) PREDICTED: uncharacterized protein LOC106070421 [B... [194](#XP_013085779.1) 2e-59

[XP_025082765.1](https://www.ncbi.nlm.nih.gov/protein/XP_025082765.1?report=genbank&log$=prottop&blast_rank=3&RID=) uncharacterized protein LOC112557240 isoform X1 [P... [147](#XP_025082765.1) 4e-39

[XP_025082766.1](https://www.ncbi.nlm.nih.gov/protein/XP_025082766.1?report=genbank&log$=prottop&blast_rank=4&RID=) uncharacterized protein LOC112557240 isoform X2 [P... [141](#XP_025082766.1) 5e-37

[XP_025082768.1](https://www.ncbi.nlm.nih.gov/protein/XP_025082768.1?report=genbank&log$=prottop&blast_rank=5&RID=) uncharacterized protein LOC112557240 isoform X3 [P... [138](#XP_025082768.1) 5e-36

[XP_009060461.1](https://www.ncbi.nlm.nih.gov/protein/XP_009060461.1?report=genbank&log$=prottop&blast_rank=6&RID=) hypothetical protein LOTGIDRAFT_234386 [Lottia gig... [128](#XP_009060461.1) 9e-32

[PVD36893.1](https://www.ncbi.nlm.nih.gov/protein/PVD36893.1?report=genbank&log$=prottop&blast_rank=7&RID=) hypothetical protein C0Q70_03884 [Pomacea canaliculata] [110](#PVD36893.1) 2e-25

[XP_009060462.1](https://www.ncbi.nlm.nih.gov/protein/XP_009060462.1?report=genbank&log$=prottop&blast_rank=8&RID=) hypothetical protein LOTGIDRAFT_234387 [Lottia gig... [107](#XP_009060462.1) 4e-24

>[RUS89244.1](https://www.ncbi.nlm.nih.gov/protein/RUS89244.1?report=genbank&log$=protalign&blast_rank=1&RID=0) hypothetical protein EGW08_002987 [Elysia chlorotica]

Length=258

Score = 292 bits (748), Expect = 2e-96, Method: Compositional matrix adjust.

Identities = 149/271 (55%), Positives = 180/271 (66%), Gaps = 52/271 (19%)

Query 7 IVAVSSLAWF-----IAGVASQATDASSHCSYLINSVSRYQPNAAAFKIYTRSRSPRVTS 61

+ A+SSL + V Q +S C+YL+NS SR Q A FK+YTRSR+PR+TS

Sbjct 4 VAAISSLLMVFLTANLISVQCQQNLVNSQCNYLVNSRSRQQGTVAPFKVYTRSRTPRITS 63

Query 62 GEPIEVTIGPFSSSLNFFNFTDFILYATPSNIANLEVEFIGPT-----SPHVGVFQLFDK 116

P+E + LYATP+N +E+EF G + +PHVGVFQLFDK

Sbjct 64 KSPVE----------------EAPLYATPANSMGMEMEFWGGSGGQSATPHVGVFQLFDK 107

Query 117 WRAGAGGLNCNPRSRAEDSVGAFEDRLLATFKQYYPNNPMLLRYHAPARNQVSVLWWPTK 176

WRAGAG L+CNPRSR+EDSVGAFEDRLL F++YYPNNPML+RY PARNQVSVLWWPT

Sbjct 108 WRAGAGALSCNPRSRSEDSVGAFEDRLLTVFRRYYPNNPMLMRYQPPARNQVSVLWWPTD 167

Query 177 EALMYPEIKFVANIKSMGNWFKLQSTPWKVNRPVDQWAN--------------------- 215

+AL YPEIKFVAN+ SMGNWFK++STPWKVNRPVDQWA+

Sbjct 168 DALAYPEIKFVANVYSMGNWFKIESTPWKVNRPVDQWASEYEKQTKLKHGAKTPNPKLIV 227

Query 216 -----TESMLAQYQAMQNNMRAMERRLEQPI 241

TE ML Q+ A+Q+NMRAMERRL+QP+

Sbjct 228 TENIVTERMLNQFHALQSNMRAMERRLDQPV 258

>[XP_013085779.1](https://www.ncbi.nlm.nih.gov/protein/XP_013085779.1?report=genbank&log$=protalign&blast_rank=2&RID=0) PREDICTED: uncharacterized protein LOC106070421 [Biomphalaria

glabrata]

Length=132

Score = 194 bits (492), Expect = 2e-59, Method: Compositional matrix adjust.

Identities = 91/125 (73%), Positives = 105/125 (84%), Gaps = 1/125 (1%)

Query 8 VAVSSLAWFIAGVASQ-ATDASSHCSYLINSVSRYQPNAAAFKIYTRSRSPRVTSGEPIE 66

+ V +A V+SQ A D +SHC+YL+NS S+YQ NAA FK+YTRSR PR+TSGEPIE

Sbjct 8 LGVCVVAIMAVTVSSQGAADPTSHCNYLVNSKSQYQENAAPFKLYTRSRQPRITSGEPIE 67

Query 67 VTIGPFSSSLNFFNFTDFILYATPSNIANLEVEFIGPTSPHVGVFQLFDKWRAGAGGLNC 126

VTIGPFS LNFFNFTDFILYATPSN ANLE+EFIG +PHVGVFQ+FD+WRAGAGGL+C

Sbjct 68 VTIGPFSQHLNFFNFTDFILYATPSNTANLEIEFIGTPNPHVGVFQMFDQWRAGAGGLSC 127

Query 127 NPRSR 131

NPRSR

Sbjct 128 NPRSR 132

>[XP_025082765.1](https://www.ncbi.nlm.nih.gov/protein/XP_025082765.1?report=genbank&log$=protalign&blast_rank=3&RID=0) uncharacterized protein LOC112557240 isoform X1 [Pomacea canaliculata]

Length=279

Score = 147 bits (370), Expect = 4e-39, Method: Compositional matrix adjust.

Identities = 82/229 (36%), Positives = 125/229 (55%), Gaps = 17/229 (7%)

Query 28 SSHCSYLINSVSRYQPNAAAFKIYTRSRSPRVTSGEPIEVTIGPFSSSLNFFNFTDFILY 87

SSHC YLI + S + NA+ FKIYTRS S V PIEV+I P L FNFTDF L+

Sbjct 45 SSHCRYLITAPSNWGVNASPFKIYTRSYSGLVRRSYPIEVSILPSHPRLGGFNFTDFALW 104

Query 88 ATPS-----NIANLEVEFIGPT-------SPHVGVFQLFDKWRAGAGGLNCNPRSRAEDS 135

A P +E+E + + +G + + ++ G + CN + ++D+

Sbjct 105 AEPDLPPFPAAPRIELEPVAGAQQAPPVATTQLGTWHVLPRFAPGTSSMKCNMYTASDDT 164

Query 136 VGAFEDRLLATFKQYYPNNPMLLRYHAPARNQVSVLWWPTKEALMYPEIKFVANIKSMGN 195

VG+FE+R+ K+ +P+ P L+ + A +R+ + LW+P +AL IKFVA +KSMG

Sbjct 165 VGSFEERMTDLLKRQFPHEPRLVSFQAASRSGAAFLWYPDDKALAQSTIKFVAKLKSMGG 224

Query 196 WFKLQSTPWKVNRPVD-----QWANTESMLAQYQAMQNNMRAMERRLEQ 239

WF L+ST W V +P W+ + + ++RA+ER+LE+

Sbjct 225 WFHLESTAWNVYKPPPPPQPFSWSQMTNTAERMADTMQSLRALERKLER 273

>[XP_025082766.1](https://www.ncbi.nlm.nih.gov/protein/XP_025082766.1?report=genbank&log$=protalign&blast_rank=4&RID=0) uncharacterized protein LOC112557240 isoform X2 [Pomacea canaliculata]

Length=270

Score = 141 bits (355), Expect = 5e-37, Method: Compositional matrix adjust.

Identities = 80/223 (36%), Positives = 119/223 (53%), Gaps = 16/223 (7%)

Query 28 SSHCSYLINSVSRYQPNAAAFKIYTRSRSPRVTSGEPIEVTIGPFSSSLNFFNFTDFILY 87

SSHC YLI + S + NA+ FKIYTRS S V PIEV+I P L FNFTDF L+

Sbjct 45 SSHCRYLITAPSNWGVNASPFKIYTRSYSGLVRRSYPIEVSILPSHPRLGGFNFTDFALW 104

Query 88 ATPS-----NIANLEVEFIGPT-------SPHVGVFQLFDKWRAGAGGLNCNPRSRAEDS 135

A P +E+E + + +G + + ++ G + CN + ++D+

Sbjct 105 AEPDLPPFPAAPRIELEPVAGAQQAPPVATTQLGTWHVLPRFAPGTSSMKCNMYTASDDT 164

Query 136 VGAFEDRLLATFKQYYPNNPMLLRYHAPARNQVSVLWWPTKEALMYPEIKFVANIKSMGN 195

VG+FE+R+ K+ +P+ P L+ + A +R+ + LW+P +AL IKFVA +KSMG

Sbjct 165 VGSFEERMTDLLKRQFPHEPRLVSFQAASRSGAAFLWYPDDKALAQSTIKFVAKLKSMGG 224

Query 196 WFKLQSTPWKVNRPVDQWANTESMLAQYQAMQNNMRAMERRLE 238

WF L+ST W V +P + YQ ++ ME+ E

Sbjct 225 WFHLESTAWNVYKP----PPPPQPFSDYQGLRRLHTGMEKLYE 263

>[XP_025082768.1](https://www.ncbi.nlm.nih.gov/protein/XP_025082768.1?report=genbank&log$=protalign&blast_rank=5&RID=0) uncharacterized protein LOC112557240 isoform X3 [Pomacea canaliculata]

Length=262

Score = 138 bits (347), Expect = 5e-36, Method: Compositional matrix adjust.

Identities = 74/193 (38%), Positives = 109/193 (56%), Gaps = 12/193 (6%)

Query 28 SSHCSYLINSVSRYQPNAAAFKIYTRSRSPRVTSGEPIEVTIGPFSSSLNFFNFTDFILY 87

SSHC YLI + S + NA+ FKIYTRS S V PIEV+I P L FNFTDF L+

Sbjct 45 SSHCRYLITAPSNWGVNASPFKIYTRSYSGLVRRSYPIEVSILPSHPRLGGFNFTDFALW 104

Query 88 ATPS-----NIANLEVEFIGPT-------SPHVGVFQLFDKWRAGAGGLNCNPRSRAEDS 135

A P +E+E + + +G + + ++ G + CN + ++D+

Sbjct 105 AEPDLPPFPAAPRIELEPVAGAQQAPPVATTQLGTWHVLPRFAPGTSSMKCNMYTASDDT 164

Query 136 VGAFEDRLLATFKQYYPNNPMLLRYHAPARNQVSVLWWPTKEALMYPEIKFVANIKSMGN 195

VG+FE+R+ K+ +P+ P L+ + A +R+ + LW+P +AL IKFVA +KSMG

Sbjct 165 VGSFEERMTDLLKRQFPHEPRLVSFQAASRSGAAFLWYPDDKALAQSTIKFVAKLKSMGG 224

Query 196 WFKLQSTPWKVNR 208

WF L+ST W V +

Sbjct 225 WFHLESTAWNVYK 237

>[XP_009060461.1](https://www.ncbi.nlm.nih.gov/protein/XP_009060461.1?report=genbank&log$=protalign&blast_rank=6&RID=0) hypothetical protein LOTGIDRAFT_234386 [Lottia gigantea]

[ESO88788.1](https://www.ncbi.nlm.nih.gov/protein/ESO88788.1?report=genbank&log$=protalign&blast_rank=6&RID=0) hypothetical protein LOTGIDRAFT_234386 [Lottia gigantea]

Length=306

Score = 128 bits (322), Expect = 9e-32, Method: Compositional matrix adjust.

Identities = 77/220 (35%), Positives = 116/220 (53%), Gaps = 10/220 (5%)

Query 28 SSHCSYLINSVSRYQPNAAAFKIYTRSRSPRVTSGEPIEVTIGPFSSSLNFFNFTDFILY 87

S CSYL + S + N A FK+ T V G PIEV I P+S NFTDF++Y

Sbjct 88 SDACSYLKSRTSIWGKNNAPFKLETDGFYGDVRRGYPIEVAIKPWSQ-FQPDNFTDFVIY 146

Query 88 AT---PSNIANLEVEFI----GPTSPHVGVFQLFDKWRAGAGGLNCNPRSRAEDSVGAFE 140

AT S + E + + GPT +GVF++ + + AGA G C+PR+ D+ G+ E

Sbjct 147 ATVAGASGVGAFEAQVMQGMPGPT--LLGVFKVVNDYAAGARGFMCDPRAMGPDAFGSAE 204

Query 141 DRLLATFKQYYPNNPMLLRYHAPARNQVSVLWWPTKEALMYPEIKFVANIKSMGNWFKLQ 200

+R + + P N R + LW+PT+ A+ I+F A +KS G+W++L+

Sbjct 205 ERRILINRALKPENQYWKTRQPAIRQYAAALWYPTEMAMGAANIRFTAKVKSDGHWYELK 264

Query 201 STPWKVNRPVDQWANTESMLAQYQAMQNNMRAMERRLEQP 240

S P D W + + ++QYQ MQ +MR +ER+ + P

Sbjct 265 SKVLTPYNPPDPWQSMAASISQYQRMQQHMRQLERQAKGP 304

>[PVD36893.1](https://www.ncbi.nlm.nih.gov/protein/PVD36893.1?report=genbank&log$=protalign&blast_rank=7&RID=0) hypothetical protein C0Q70_03884 [Pomacea canaliculata]

Length=253

Score = 110 bits (275), Expect = 2e-25, Method: Compositional matrix adjust.

Identities = 62/172 (36%), Positives = 94/172 (55%), Gaps = 12/172 (7%)

Query 28 SSHCSYLINSVSRYQPNAAAFKIYTRSRSPRVTSGEPIEVTIGPFSSSLNFFNFTDFILY 87

SSHC YLI + S + NA+ FKIYTRS S V PIEV+I P L FNFTDF L+

Sbjct 45 SSHCRYLITAPSNWGVNASPFKIYTRSYSGLVRRSYPIEVSILPSHPRLGGFNFTDFALW 104

Query 88 ATPS-----NIANLEVEFIGP-------TSPHVGVFQLFDKWRAGAGGLNCNPRSRAEDS 135

A P +E+E + + +G + + ++ G + CN + ++D+

Sbjct 105 AEPDLPPFPAAPRIELEPVAGAQQAPPVATTQLGTWHVLPRFAPGTSSMKCNMYTASDDT 164

Query 136 VGAFEDRLLATFKQYYPNNPMLLRYHAPARNQVSVLWWPTKEALMYPEIKFV 187

VG+FE+R+ K+ +P+ P L+ + A +R+ + LW+P +AL IKFV

Sbjct 165 VGSFEERMTDLLKRQFPHEPRLVSFQAASRSGAAFLWYPDDKALAQSTIKFV 216

>[XP_009060462.1](https://www.ncbi.nlm.nih.gov/protein/XP_009060462.1?report=genbank&log$=protalign&blast_rank=8&RID=0) hypothetical protein LOTGIDRAFT_234387 [Lottia gigantea]

[ESO88789.1](https://www.ncbi.nlm.nih.gov/protein/ESO88789.1?report=genbank&log$=protalign&blast_rank=8&RID=0) hypothetical protein LOTGIDRAFT_234387 [Lottia gigantea]

Length=254

Score = 107 bits (266), Expect = 4e-24, Method: Compositional matrix adjust.

Identities = 75/228 (33%), Positives = 121/228 (53%), Gaps = 11/228 (5%)

Query 17 IAGVASQATDASSHCSYLINSVSRYQPNAAAFKI-YTRSRSPRVTSGEPIEVTIGPFSSS 75

+ G + TD+ C+YL S + N++ F T ++ SG+P+EVTI +SS

Sbjct 26 LLGGQRKLTDS---CTYLQTRSSIWGENSSPFYFNITGFYGYQIRSGQPLEVTIKRWSS- 81

Query 76 LNFFNFTDFILYATPS---NIANLEVEFIGPTSP--HVGVFQLFDKWRAGAGGLNCNPRS 130

+ NFTDF +YATP+ IA + + + P P ++GVF+L + G G C+PR+

Sbjct 82 ITPDNFTDFFMYATPTAPRGIAQMNGQGV-PGMPVLYLGVFKLVPPYNTGGRGFTCDPRA 140

Query 131 RAEDSVGAFEDRLLATFKQYYPNNPMLLRYHAPARNQVSVLWWPTKEALMYPEIKFVANI 190

D+ G+ E+R + + PN P R SVLW+PT+ A+ P I+F+ +

Sbjct 141 IGYDAFGSVEERRILIKRAMEPNKPYWHNRKPYVRKYTSVLWFPTEMAMNVPSIQFIGKV 200

Query 191 KSMGNWFKLQSTPWKVNRPVDQWANTESMLAQYQAMQNNMRAMERRLE 238

KS G+W++++S + P D + S L+Q +M NMR ER+ +

Sbjct 201 KSDGHWYEIKSRSFVPYIPPDPMSTMYSSLSQVSSMSQNMRQFERQAD 248

Lambda K H a alpha

0.320 0.131 0.406 0.792 4.96

Gapped

Lambda K H a alpha sigma

0.267 0.0410 0.140 1.90 42.6 43.6

Effective search space used: 5590052562440

**Query=** Cnem_R37432942 Gly_rich3 len=697 num_reads=360970 avg_cov=65037.1

contig_cov=100.0% ORF=98

Length=99

***** No hits found *****

Lambda K H a alpha

0.325 0.163 0.530 0.792 4.96

Gapped

Lambda K H a alpha sigma

0.267 0.0410 0.140 1.90 42.6 43.6

Effective search space used: 2916016919768

**Query=** Cnem_Gly_rich2

Length=81

***** No hits found *****

Lambda K H a alpha

0.318 0.154 0.480 0.792 4.96

Gapped

Lambda K H a alpha sigma

0.267 0.0410 0.140 1.90 42.6 43.6

Effective search space used: 2877103594504

**Query=** Cnem_R37577449 Peroxidase len=2200 num_reads=4834 avg_cov=229.2

contig_cov=99.8% ORF=628

Length=628

Score E

Sequences producing significant alignments: (Bits) Value

[RUS88352.1](https://www.ncbi.nlm.nih.gov/protein/RUS88352.1?report=genbank&log$=prottop&blast_rank=1&RID=) hypothetical protein EGW08_003864 [Elysia chlorotica] [523](#RUS88352.1) 5e-178

[XP_025107474.1](https://www.ncbi.nlm.nih.gov/protein/XP_025107474.1?report=genbank&log$=prottop&blast_rank=2&RID=) myeloperoxidase-like [Pomacea canaliculata] [389](#XP_025107474.1) 6e-124

[XP_013088542.1](https://www.ncbi.nlm.nih.gov/protein/XP_013088542.1?report=genbank&log$=prottop&blast_rank=3&RID=) PREDICTED: lactoperoxidase-like [Biomphalaria glab... [357](#XP_013088542.1) 1e-114

[PVD24488.1](https://www.ncbi.nlm.nih.gov/protein/PVD24488.1?report=genbank&log$=prottop&blast_rank=4&RID=) hypothetical protein C0Q70_14971 [Pomacea canaliculata] [359](#PVD24488.1) 2e-114

[XP_005110224.1](https://www.ncbi.nlm.nih.gov/protein/XP_005110224.1?report=genbank&log$=prottop&blast_rank=5&RID=) peroxidasin isoform X1 [Aplysia californica] [361](#XP_005110224.1) 1e-112

[XP_025083738.1](https://www.ncbi.nlm.nih.gov/protein/XP_025083738.1?report=genbank&log$=prottop&blast_rank=6&RID=) chorion peroxidase-like [Pomacea canaliculata] [351](#XP_025083738.1) 1e-108

[PVD36952.1](https://www.ncbi.nlm.nih.gov/protein/PVD36952.1?report=genbank&log$=prottop&blast_rank=7&RID=) hypothetical protein C0Q70_03945 [Pomacea canaliculata] [350](#PVD36952.1) 5e-108

[XP_013088968.1](https://www.ncbi.nlm.nih.gov/protein/XP_013088968.1?report=genbank&log$=prottop&blast_rank=8&RID=) PREDICTED: peroxidasin-like [Biomphalaria glabrata] [345](#XP_013088968.1) 5e-108

[XP_025096385.1](https://www.ncbi.nlm.nih.gov/protein/XP_025096385.1?report=genbank&log$=prottop&blast_rank=9&RID=) myeloperoxidase-like [Pomacea canaliculata] [355](#XP_025096385.1) 7e-108

[QIQ54711.1](https://www.ncbi.nlm.nih.gov/protein/QIQ54711.1?report=genbank&log$=prottop&blast_rank=10&RID=) capsule gland specific secretory protein [Reishia bronni] [348](#QIQ54711.1) 1e-107

[XP_005099074.2](https://www.ncbi.nlm.nih.gov/protein/XP_005099074.2?report=genbank&log$=prottop&blast_rank=11&RID=) chorion peroxidase [Aplysia californica] [348](#XP_005099074.2) 2e-107

[XP_035828376.1](https://www.ncbi.nlm.nih.gov/protein/XP_035828376.1?report=genbank&log$=prottop&blast_rank=12&RID=) chorion peroxidase [Aplysia californica] [338](#XP_035828376.1) 1e-103

[XP_013079813.1](https://www.ncbi.nlm.nih.gov/protein/XP_013079813.1?report=genbank&log$=prottop&blast_rank=13&RID=) PREDICTED: myeloperoxidase-like [Biomphalaria glab... [321](#XP_013079813.1) 3e-101

[XP_034310730.1](https://www.ncbi.nlm.nih.gov/protein/XP_034310730.1?report=genbank&log$=prottop&blast_rank=14&RID=) eosinophil peroxidase-like [Crassostrea gigas] [333](#XP_034310730.1) 3e-101

[ODM94915.1](https://www.ncbi.nlm.nih.gov/protein/ODM94915.1?report=genbank&log$=prottop&blast_rank=15&RID=) Chorion peroxidase [Orchesella cincta] [336](#ODM94915.1) 7e-101

[XP_011426440.2](https://www.ncbi.nlm.nih.gov/protein/XP_011426440.2?report=genbank&log$=prottop&blast_rank=16&RID=) eosinophil peroxidase [Crassostrea gigas] [331](#XP_011426440.2) 2e-100

[XP_025107410.1](https://www.ncbi.nlm.nih.gov/protein/XP_025107410.1?report=genbank&log$=prottop&blast_rank=17&RID=) chorion peroxidase-like [Pomacea canaliculata] [329](#XP_025107410.1) 2e-100

[XP_035828707.1](https://www.ncbi.nlm.nih.gov/protein/XP_035828707.1?report=genbank&log$=prottop&blast_rank=18&RID=) peroxidase-like protein 3 isoform X2 [Aplysia cali... [319](#XP_035828707.1) 2e-98

[OWF41499.1](https://www.ncbi.nlm.nih.gov/protein/OWF41499.1?report=genbank&log$=prottop&blast_rank=19&RID=) Peroxidase-like protein [Mizuhopecten yessoensis] [325](#OWF41499.1) 4e-98

[XP_009049107.1](https://www.ncbi.nlm.nih.gov/protein/XP_009049107.1?report=genbank&log$=prottop&blast_rank=20&RID=) hypothetical protein LOTGIDRAFT_238662 [Lottia gig... [323](#XP_009049107.1) 5e-98

[XP_033756163.1](https://www.ncbi.nlm.nih.gov/protein/XP_033756163.1?report=genbank&log$=prottop&blast_rank=21&RID=) uncharacterized protein LOC117338907 [Pecten maximus] [335](#XP_033756163.1) 8e-97

[RUS72077.1](https://www.ncbi.nlm.nih.gov/protein/RUS72077.1?report=genbank&log$=prottop&blast_rank=22&RID=) hypothetical protein EGW08_020159 [Elysia chlorotica] [314](#RUS72077.1) 4e-95

[OQV26124.1](https://www.ncbi.nlm.nih.gov/protein/OQV26124.1?report=genbank&log$=prottop&blast_rank=23&RID=) Chorion peroxidase [Hypsibius dujardini] [323](#OQV26124.1) 5e-95

[XP_009052457.1](https://www.ncbi.nlm.nih.gov/protein/XP_009052457.1?report=genbank&log$=prottop&blast_rank=24&RID=) hypothetical protein LOTGIDRAFT_115464 [Lottia gig... [317](#XP_009052457.1) 5e-95

[RUS84195.1](https://www.ncbi.nlm.nih.gov/protein/RUS84195.1?report=genbank&log$=prottop&blast_rank=25&RID=) hypothetical protein EGW08_008035 [Elysia chlorotica] [311](#RUS84195.1) 7e-95

[XP_002425239.1](https://www.ncbi.nlm.nih.gov/protein/XP_002425239.1?report=genbank&log$=prottop&blast_rank=26&RID=) Chorion peroxidase precursor, putative [Pediculus ... [320](#XP_002425239.1) 2e-94

[XP_033761598.1](https://www.ncbi.nlm.nih.gov/protein/XP_033761598.1?report=genbank&log$=prottop&blast_rank=27&RID=) myeloperoxidase-like [Pecten maximus] [315](#XP_033761598.1) 1e-93

[RUS74146.1](https://www.ncbi.nlm.nih.gov/protein/RUS74146.1?report=genbank&log$=prottop&blast_rank=28&RID=) hypothetical protein EGW08_018101 [Elysia chlorotica] [311](#RUS74146.1) 4e-93

[XP_033762435.1](https://www.ncbi.nlm.nih.gov/protein/XP_033762435.1?report=genbank&log$=prottop&blast_rank=29&RID=) lactoperoxidase-like [Pecten maximus] [310](#XP_033762435.1) 2e-92

[XP_012940602.1](https://www.ncbi.nlm.nih.gov/protein/XP_012940602.1?report=genbank&log$=prottop&blast_rank=30&RID=) chorion peroxidase [Aplysia californica] [306](#XP_012940602.1) 4e-92

[XP_034310733.1](https://www.ncbi.nlm.nih.gov/protein/XP_034310733.1?report=genbank&log$=prottop&blast_rank=31&RID=) eosinophil peroxidase [Crassostrea gigas] [308](#XP_034310733.1) 7e-92

[XP_035658181.1](https://www.ncbi.nlm.nih.gov/protein/XP_035658181.1?report=genbank&log$=prottop&blast_rank=32&RID=) peroxidase mlt-7-like [Branchiostoma floridae] [308](#XP_035658181.1) 1e-91

[XP_021361183.1](https://www.ncbi.nlm.nih.gov/protein/XP_021361183.1?report=genbank&log$=prottop&blast_rank=33&RID=) myeloperoxidase-like [Mizuhopecten yessoensis] [307](#XP_021361183.1) 3e-91

[XP_009043737.1](https://www.ncbi.nlm.nih.gov/protein/XP_009043737.1?report=genbank&log$=prottop&blast_rank=34&RID=) hypothetical protein LOTGIDRAFT_152002 [Lottia gig... [305](#XP_009043737.1) 4e-91

[XP_021373766.1](https://www.ncbi.nlm.nih.gov/protein/XP_021373766.1?report=genbank&log$=prottop&blast_rank=35&RID=) chorion peroxidase-like [Mizuhopecten yessoensis] [306](#XP_021373766.1) 8e-91

[XP_019638393.1](https://www.ncbi.nlm.nih.gov/protein/XP_019638393.1?report=genbank&log$=prottop&blast_rank=36&RID=) PREDICTED: peroxidasin homolog [Branchiostoma belc... [306](#XP_019638393.1) 8e-91

[XP_023706652.1](https://www.ncbi.nlm.nih.gov/protein/XP_023706652.1?report=genbank&log$=prottop&blast_rank=37&RID=) peroxidase [Cryptotermes secundus] [310](#XP_023706652.1) 1e-90

[XP_022246593.1](https://www.ncbi.nlm.nih.gov/protein/XP_022246593.1?report=genbank&log$=prottop&blast_rank=38&RID=) peroxidase-like isoform X2 [Limulus polyphemus] [306](#XP_022246593.1) 2e-90

[XP_019633295.1](https://www.ncbi.nlm.nih.gov/protein/XP_019633295.1?report=genbank&log$=prottop&blast_rank=39&RID=) PREDICTED: peroxidasin homolog [Branchiostoma belc... [303](#XP_019633295.1) 2e-90

[XP_022246592.1](https://www.ncbi.nlm.nih.gov/protein/XP_022246592.1?report=genbank&log$=prottop&blast_rank=40&RID=) peroxidase-like isoform X1 [Limulus polyphemus] [306](#XP_022246592.1) 2e-90

[XP_021373773.1](https://www.ncbi.nlm.nih.gov/protein/XP_021373773.1?report=genbank&log$=prottop&blast_rank=41&RID=) myeloperoxidase-like [Mizuhopecten yessoensis] [308](#XP_021373773.1) 5e-90

[XP_034310072.1](https://www.ncbi.nlm.nih.gov/protein/XP_034310072.1?report=genbank&log$=prottop&blast_rank=42&RID=) eosinophil peroxidase [Crassostrea gigas] [303](#XP_034310072.1) 9e-90

[KAE8751717.1](https://www.ncbi.nlm.nih.gov/protein/KAE8751717.1?report=genbank&log$=prottop&blast_rank=43&RID=) Chorion peroxidase-like-2 [Frankliniella occidentalis] [306](#KAE8751717.1) 1e-89

[XP_026286195.1](https://www.ncbi.nlm.nih.gov/protein/XP_026286195.1?report=genbank&log$=prottop&blast_rank=44&RID=) peroxidase-like [Frankliniella occidentalis] [308](#XP_026286195.1) 2e-89

[XP_033761812.1](https://www.ncbi.nlm.nih.gov/protein/XP_033761812.1?report=genbank&log$=prottop&blast_rank=45&RID=) peroxidase-like [Pecten maximus] [303](#XP_033761812.1) 2e-89

[XP_034310729.1](https://www.ncbi.nlm.nih.gov/protein/XP_034310729.1?report=genbank&log$=prottop&blast_rank=46&RID=) eosinophil peroxidase-like [Crassostrea gigas] [301](#XP_034310729.1) 3e-89

[XP_019616874.1](https://www.ncbi.nlm.nih.gov/protein/XP_019616874.1?report=genbank&log$=prottop&blast_rank=47&RID=) PREDICTED: peroxidasin-like [Branchiostoma belcheri] [298](#XP_019616874.1) 3e-89

[XP_013419078.1](https://www.ncbi.nlm.nih.gov/protein/XP_013419078.1?report=genbank&log$=prottop&blast_rank=48&RID=) peroxidasin-like [Lingula anatina] [303](#XP_013419078.1) 4e-89

[RUS85745.1](https://www.ncbi.nlm.nih.gov/protein/RUS85745.1?report=genbank&log$=prottop&blast_rank=49&RID=) hypothetical protein EGW08_006459 [Elysia chlorotica] [297](#RUS85745.1) 7e-89

[TRY68556.1](https://www.ncbi.nlm.nih.gov/protein/TRY68556.1?report=genbank&log$=prottop&blast_rank=50&RID=) hypothetical protein TCAL_04086 [Tigriopus californicus] [303](#TRY68556.1) 1e-88

[XP_033756984.1](https://www.ncbi.nlm.nih.gov/protein/XP_033756984.1?report=genbank&log$=prottop&blast_rank=51&RID=) peroxidasin-like [Pecten maximus] [300](#XP_033756984.1) 1e-88

[XP_036355162.1](https://www.ncbi.nlm.nih.gov/protein/XP_036355162.1?report=genbank&log$=prottop&blast_rank=52&RID=) peroxidase-like protein isoform X1 [Octopus vulgaris] [303](#XP_036355162.1) 2e-88

[XP_029656077.1](https://www.ncbi.nlm.nih.gov/protein/XP_029656077.1?report=genbank&log$=prottop&blast_rank=53&RID=) peroxidase-like protein isoform X2 [Octopus vulgaris] [303](#XP_029656077.1) 2e-88

[XP_013422154.2](https://www.ncbi.nlm.nih.gov/protein/XP_013422154.2?report=genbank&log$=prottop&blast_rank=54&RID=) chorion peroxidase-like [Lingula anatina] [305](#XP_013422154.2) 2e-88

[XP_018902871.1](https://www.ncbi.nlm.nih.gov/protein/XP_018902871.1?report=genbank&log$=prottop&blast_rank=55&RID=) PREDICTED: peroxidase-like [Bemisia tabaci] [305](#XP_018902871.1) 3e-88

[XP_022240998.1](https://www.ncbi.nlm.nih.gov/protein/XP_022240998.1?report=genbank&log$=prottop&blast_rank=56&RID=) chorion peroxidase-like [Limulus polyphemus] [299](#XP_022240998.1) 5e-88

[XP_034244813.1](https://www.ncbi.nlm.nih.gov/protein/XP_034244813.1?report=genbank&log$=prottop&blast_rank=57&RID=) peroxidase-like [Thrips palmi] [304](#XP_034244813.1) 5e-88

[XP_011505800.1](https://www.ncbi.nlm.nih.gov/protein/XP_011505800.1?report=genbank&log$=prottop&blast_rank=58&RID=) PREDICTED: peroxidase-like [Ceratosolen solmsi mar... [302](#XP_011505800.1) 5e-88

[XP_025096592.1](https://www.ncbi.nlm.nih.gov/protein/XP_025096592.1?report=genbank&log$=prottop&blast_rank=59&RID=) chorion peroxidase-like [Pomacea canaliculata] [304](#XP_025096592.1) 7e-88

[XP_025078500.1](https://www.ncbi.nlm.nih.gov/protein/XP_025078500.1?report=genbank&log$=prottop&blast_rank=60&RID=) chorion peroxidase-like [Pomacea canaliculata] [296](#XP_025078500.1) 8e-88

[XP_021371886.1](https://www.ncbi.nlm.nih.gov/protein/XP_021371886.1?report=genbank&log$=prottop&blast_rank=61&RID=) uncharacterized protein LOC110462299 [Mizuhopecten... [308](#XP_021371886.1) 8e-88

[XP_019879548.1](https://www.ncbi.nlm.nih.gov/protein/XP_019879548.1?report=genbank&log$=prottop&blast_rank=62&RID=) PREDICTED: LOW QUALITY PROTEIN: chorion peroxidase... [308](#XP_019879548.1) 1e-87

[KAF2367819.1](https://www.ncbi.nlm.nih.gov/protein/KAF2367819.1?report=genbank&log$=prottop&blast_rank=63&RID=) hypothetical protein FHG87_001411 [Trinorchestia lon... [299](#KAF2367819.1) 2e-87

[EEC15885.1](https://www.ncbi.nlm.nih.gov/protein/EEC15885.1?report=genbank&log$=prottop&blast_rank=64&RID=) peroxinectin, putative [Ixodes scapularis] [295](#EEC15885.1) 2e-87

[XP_021365517.1](https://www.ncbi.nlm.nih.gov/protein/XP_021365517.1?report=genbank&log$=prottop&blast_rank=65&RID=) peroxidase-like protein [Mizuhopecten yessoensis] [297](#XP_021365517.1) 2e-87

[XP_023210206.1](https://www.ncbi.nlm.nih.gov/protein/XP_023210206.1?report=genbank&log$=prottop&blast_rank=66&RID=) chorion peroxidase-like [Centruroides sculpturatus] [308](#XP_023210206.1) 2e-87

[KXJ70077.1](https://www.ncbi.nlm.nih.gov/protein/KXJ70077.1?report=genbank&log$=prottop&blast_rank=67&RID=) hypothetical protein RP20_CCG024878 [Aedes albopictus] [300](#KXJ70077.1) 3e-87

[XP_029850035.1](https://www.ncbi.nlm.nih.gov/protein/XP_029850035.1?report=genbank&log$=prottop&blast_rank=68&RID=) thyroid peroxidase [Ixodes scapularis] [306](#XP_029850035.1) 3e-87

[XP_009058281.1](https://www.ncbi.nlm.nih.gov/protein/XP_009058281.1?report=genbank&log$=prottop&blast_rank=69&RID=) hypothetical protein LOTGIDRAFT_163526 [Lottia gig... [295](#XP_009058281.1) 4e-87

[OWF44553.1](https://www.ncbi.nlm.nih.gov/protein/OWF44553.1?report=genbank&log$=prottop&blast_rank=70&RID=) Peroxidase-like protein [Mizuhopecten yessoensis] [293](#OWF44553.1) 4e-87

[XP_021963148.1](https://www.ncbi.nlm.nih.gov/protein/XP_021963148.1?report=genbank&log$=prottop&blast_rank=71&RID=) peroxidase isoform X1 [Folsomia candida] [301](#XP_021963148.1) 5e-87

[GAU92485.1](https://www.ncbi.nlm.nih.gov/protein/GAU92485.1?report=genbank&log$=prottop&blast_rank=72&RID=) hypothetical protein RvY_04560 [Ramazzottius varieorna... [292](#GAU92485.1) 5e-87

[XP_021963149.1](https://www.ncbi.nlm.nih.gov/protein/XP_021963149.1?report=genbank&log$=prottop&blast_rank=73&RID=) peroxidase isoform X2 [Folsomia candida] [301](#XP_021963149.1) 6e-87

[XP_029714173.1](https://www.ncbi.nlm.nih.gov/protein/XP_029714173.1?report=genbank&log$=prottop&blast_rank=74&RID=) LOW QUALITY PROTEIN: chorion peroxidase-like [Aede... [299](#XP_029714173.1) 1e-86

[XP_022701473.1](https://www.ncbi.nlm.nih.gov/protein/XP_022701473.1?report=genbank&log$=prottop&blast_rank=75&RID=) peroxidase-like isoform X2 [Varroa jacobsoni] [296](#XP_022701473.1) 1e-86

[XP_029733783.1](https://www.ncbi.nlm.nih.gov/protein/XP_029733783.1?report=genbank&log$=prottop&blast_rank=76&RID=) LOW QUALITY PROTEIN: chorion peroxidase-like [Aede... [300](#XP_029733783.1) 1e-86

[XP_025194360.1](https://www.ncbi.nlm.nih.gov/protein/XP_025194360.1?report=genbank&log$=prottop&blast_rank=77&RID=) peroxidase-like [Melanaphis sacchari] [300](#XP_025194360.1) 1e-86

[XP_019544703.2](https://www.ncbi.nlm.nih.gov/protein/XP_019544703.2?report=genbank&log$=prottop&blast_rank=78&RID=) chorion peroxidase [Aedes albopictus] [298](#XP_019544703.2) 1e-86

[XP_033761418.1](https://www.ncbi.nlm.nih.gov/protein/XP_033761418.1?report=genbank&log$=prottop&blast_rank=79&RID=) myeloperoxidase-like [Pecten maximus] [299](#XP_033761418.1) 1e-86

[XP_034310503.1](https://www.ncbi.nlm.nih.gov/protein/XP_034310503.1?report=genbank&log$=prottop&blast_rank=80&RID=) peroxidasin-like isoform X1 [Crassostrea gigas] [295](#XP_034310503.1) 1e-86

[XP_029733779.1](https://www.ncbi.nlm.nih.gov/protein/XP_029733779.1?report=genbank&log$=prottop&blast_rank=81&RID=) chorion peroxidase-like [Aedes albopictus] [299](#XP_029733779.1) 1e-86

[XP_021351643.1](https://www.ncbi.nlm.nih.gov/protein/XP_021351643.1?report=genbank&log$=prottop&blast_rank=82&RID=) chorion peroxidase-like [Mizuhopecten yessoensis] [298](#XP_021351643.1) 2e-86

[XP_022650762.1](https://www.ncbi.nlm.nih.gov/protein/XP_022650762.1?report=genbank&log$=prottop&blast_rank=83&RID=) peroxidase-like isoform X3 [Varroa destructor] [295](#XP_022650762.1) 2e-86

[XP_021375133.1](https://www.ncbi.nlm.nih.gov/protein/XP_021375133.1?report=genbank&log$=prottop&blast_rank=84&RID=) lactoperoxidase-like [Mizuhopecten yessoensis] [294](#XP_021375133.1) 2e-86

[XP_015373617.1](https://www.ncbi.nlm.nih.gov/protein/XP_015373617.1?report=genbank&log$=prottop&blast_rank=85&RID=) PREDICTED: peroxidase-like [Diuraphis noxia] [298](#XP_015373617.1) 3e-86

[XP_023335820.1](https://www.ncbi.nlm.nih.gov/protein/XP_023335820.1?report=genbank&log$=prottop&blast_rank=86&RID=) chorion peroxidase-like [Eurytemora affinis] [298](#XP_023335820.1) 3e-86

[VEN39256.1](https://www.ncbi.nlm.nih.gov/protein/VEN39256.1?report=genbank&log$=prottop&blast_rank=87&RID=) unnamed protein product [Callosobruchus maculatus] [296](#VEN39256.1) 4e-86

[CAC5395001.1](https://www.ncbi.nlm.nih.gov/protein/CAC5395001.1?report=genbank&log$=prottop&blast_rank=88&RID=) PXDN [Mytilus coruscus] [295](#CAC5395001.1) 4e-86

[XP_012253358.1](https://www.ncbi.nlm.nih.gov/protein/XP_012253358.1?report=genbank&log$=prottop&blast_rank=89&RID=) uncharacterized protein LOC105684530 isoform X2 [A... [305](#XP_012253358.1) 4e-86

[XP_012253356.1](https://www.ncbi.nlm.nih.gov/protein/XP_012253356.1?report=genbank&log$=prottop&blast_rank=90&RID=) uncharacterized protein LOC105684530 isoform X1 [A... [305](#XP_012253356.1) 5e-86

[XP_019761035.1](https://www.ncbi.nlm.nih.gov/protein/XP_019761035.1?report=genbank&log$=prottop&blast_rank=91&RID=) PREDICTED: peroxidasin-like [Dendroctonus ponderosae] [302](#XP_019761035.1) 5e-86

[XP_022173288.1](https://www.ncbi.nlm.nih.gov/protein/XP_022173288.1?report=genbank&log$=prottop&blast_rank=92&RID=) peroxidase-like [Myzus persicae] [298](#XP_022173288.1) 5e-86

[XP_029845375.1](https://www.ncbi.nlm.nih.gov/protein/XP_029845375.1?report=genbank&log$=prottop&blast_rank=93&RID=) peroxidase [Ixodes scapularis] [294](#XP_029845375.1) 6e-86

[XP_029850024.1](https://www.ncbi.nlm.nih.gov/protein/XP_029850024.1?report=genbank&log$=prottop&blast_rank=94&RID=) peroxidase [Ixodes scapularis] [293](#XP_029850024.1) 7e-86

[OQR78414.1](https://www.ncbi.nlm.nih.gov/protein/OQR78414.1?report=genbank&log$=prottop&blast_rank=95&RID=) peroxidasin-like [Tropilaelaps mercedesae] [294](#OQR78414.1) 7e-86

[EAT35336.2](https://www.ncbi.nlm.nih.gov/protein/EAT35336.2?report=genbank&log$=prottop&blast_rank=96&RID=) AAEL012481-PA [Aedes aegypti] [293](#EAT35336.2) 9e-86

[EAT35940.1](https://www.ncbi.nlm.nih.gov/protein/EAT35940.1?report=genbank&log$=prottop&blast_rank=97&RID=) AAEL011941-PA [Aedes aegypti] [295](#EAT35940.1) 1e-85

[XP_027206755.1](https://www.ncbi.nlm.nih.gov/protein/XP_027206755.1?report=genbank&log$=prottop&blast_rank=98&RID=) myeloperoxidase-like [Penaeus vannamei] [296](#XP_027206755.1) 1e-85

[XP_001662097.2](https://www.ncbi.nlm.nih.gov/protein/XP_001662097.2?report=genbank&log$=prottop&blast_rank=99&RID=) chorion peroxidase [Aedes aegypti] [295](#XP_001662097.2) 2e-85

[XP_019919199.2](https://www.ncbi.nlm.nih.gov/protein/XP_019919199.2?report=genbank&log$=prottop&blast_rank=100&RID=) peroxidase-like protein [Crassostrea gigas] [293](#XP_019919199.2) 2e-85

[XP_031620162.1](https://www.ncbi.nlm.nih.gov/protein/XP_031620162.1?report=genbank&log$=prottop&blast_rank=101&RID=) chorion peroxidase [Contarinia nasturtii] [294](#XP_031620162.1) 2e-85

[AIZ68325.1](https://www.ncbi.nlm.nih.gov/protein/AIZ68325.1?report=genbank&log$=prottop&blast_rank=102&RID=) peroxinectin [Caligus rogercresseyi] [293](#AIZ68325.1) 2e-85

[KAE9531205.1](https://www.ncbi.nlm.nih.gov/protein/KAE9531205.1?report=genbank&log$=prottop&blast_rank=103&RID=) hypothetical protein AGLY_010411 [Aphis glycines] [296](#KAE9531205.1) 2e-85

[KAF6029143.1](https://www.ncbi.nlm.nih.gov/protein/KAF6029143.1?report=genbank&log$=prottop&blast_rank=104&RID=) hypothetical protein EB796_012540 [Bugula neritina] [290](#KAF6029143.1) 2e-85

[XP_025416768.1](https://www.ncbi.nlm.nih.gov/protein/XP_025416768.1?report=genbank&log$=prottop&blast_rank=105&RID=) chorion peroxidase-like [Sipha flava] [296](#XP_025416768.1) 3e-85

[XP_034310732.1](https://www.ncbi.nlm.nih.gov/protein/XP_034310732.1?report=genbank&log$=prottop&blast_rank=106&RID=) eosinophil peroxidase isoform X2 [Crassostrea gigas] [291](#XP_034310732.1) 4e-85

[RWS06037.1](https://www.ncbi.nlm.nih.gov/protein/RWS06037.1?report=genbank&log$=prottop&blast_rank=107&RID=) Peroxidase-like protein [Dinothrombium tinctorium] [289](#RWS06037.1) 4e-85

[XP_013079906.1](https://www.ncbi.nlm.nih.gov/protein/XP_013079906.1?report=genbank&log$=prottop&blast_rank=108&RID=) PREDICTED: peroxidase-like [Biomphalaria glabrata] [288](#XP_013079906.1) 4e-85

[VVC40615.1](https://www.ncbi.nlm.nih.gov/protein/VVC40615.1?report=genbank&log$=prottop&blast_rank=109&RID=) Proteinase, regulatory CLIP domain,Haem peroxidase,Hae... [295](#VVC40615.1) 6e-85

[XP_026811832.1](https://www.ncbi.nlm.nih.gov/protein/XP_026811832.1?report=genbank&log$=prottop&blast_rank=110&RID=) peroxidase-like [Rhopalosiphum maidis] [295](#XP_026811832.1) 6e-85

[RZF32946.1](https://www.ncbi.nlm.nih.gov/protein/RZF32946.1?report=genbank&log$=prottop&blast_rank=111&RID=) hypothetical protein LSTR_LSTR000816 [Laodelphax stria... [302](#RZF32946.1) 6e-85

[XP_026725385.1](https://www.ncbi.nlm.nih.gov/protein/XP_026725385.1?report=genbank&log$=prottop&blast_rank=112&RID=) chorion peroxidase-like isoform X2 [Trichoplusia ni] [299](#XP_026725385.1) 6e-85

[XP_021358062.1](https://www.ncbi.nlm.nih.gov/protein/XP_021358062.1?report=genbank&log$=prottop&blast_rank=113&RID=) peroxidase-like [Mizuhopecten yessoensis] [292](#XP_021358062.1) 6e-85

[KAF2366503.1](https://www.ncbi.nlm.nih.gov/protein/KAF2366503.1?report=genbank&log$=prottop&blast_rank=114&RID=) hypothetical protein FHG87_002743 [Trinorchestia lon... [296](#KAF2366503.1) 7e-85

[XP_019641123.1](https://www.ncbi.nlm.nih.gov/protein/XP_019641123.1?report=genbank&log$=prottop&blast_rank=115&RID=) PREDICTED: peroxidasin homolog [Branchiostoma belc... [291](#XP_019641123.1) 7e-85

[XP_022650759.1](https://www.ncbi.nlm.nih.gov/protein/XP_022650759.1?report=genbank&log$=prottop&blast_rank=116&RID=) peroxidase-like isoform X1 [Varroa destructor] [291](#XP_022650759.1) 9e-85

[XP_013775632.1](https://www.ncbi.nlm.nih.gov/protein/XP_013775632.1?report=genbank&log$=prottop&blast_rank=117&RID=) peroxidase-like [Limulus polyphemus] [293](#XP_013775632.1) 9e-85

[XP_027222616.1](https://www.ncbi.nlm.nih.gov/protein/XP_027222616.1?report=genbank&log$=prottop&blast_rank=118&RID=) LOW QUALITY PROTEIN: chorion peroxidase-like [Pena... [294](#XP_027222616.1) 1e-84

[XP_034327622.1](https://www.ncbi.nlm.nih.gov/protein/XP_034327622.1?report=genbank&log$=prottop&blast_rank=119&RID=) peroxidase-like protein [Crassostrea gigas] [290](#XP_034327622.1) 1e-84

[PVD30062.1](https://www.ncbi.nlm.nih.gov/protein/PVD30062.1?report=genbank&log$=prottop&blast_rank=120&RID=) hypothetical protein C0Q70_09323 [Pomacea canaliculata] [292](#PVD30062.1) 1e-84

[XP_027849543.1](https://www.ncbi.nlm.nih.gov/protein/XP_027849543.1?report=genbank&log$=prottop&blast_rank=121&RID=) chorion peroxidase-like [Aphis gossypii] [294](#XP_027849543.1) 2e-84

[XP_021378191.1](https://www.ncbi.nlm.nih.gov/protein/XP_021378191.1?report=genbank&log$=prottop&blast_rank=122&RID=) myeloperoxidase-like [Mizuhopecten yessoensis] [291](#XP_021378191.1) 2e-84

[XP_015912051.1](https://www.ncbi.nlm.nih.gov/protein/XP_015912051.1?report=genbank&log$=prottop&blast_rank=123&RID=) peroxidase-like [Parasteatoda tepidariorum] [290](#XP_015912051.1) 2e-84

[KAA0203707.1](https://www.ncbi.nlm.nih.gov/protein/KAA0203707.1?report=genbank&log$=prottop&blast_rank=124&RID=) Peroxidase-like [Hyalella azteca] [294](#KAA0203707.1) 2e-84

[RZF43124.1](https://www.ncbi.nlm.nih.gov/protein/RZF43124.1?report=genbank&log$=prottop&blast_rank=125&RID=) hypothetical protein LSTR_LSTR001302 [Laodelphax stria... [296](#RZF43124.1) 2e-84

[XP_021355679.1](https://www.ncbi.nlm.nih.gov/protein/XP_021355679.1?report=genbank&log$=prottop&blast_rank=126&RID=) myeloperoxidase-like isoform X1 [Mizuhopecten yess... [290](#XP_021355679.1) 2e-84

[XP_024215952.1](https://www.ncbi.nlm.nih.gov/protein/XP_024215952.1?report=genbank&log$=prottop&blast_rank=127&RID=) peroxidase-like isoform X2 [Halyomorpha halys] [293](#XP_024215952.1) 3e-84

[XP_026725384.1](https://www.ncbi.nlm.nih.gov/protein/XP_026725384.1?report=genbank&log$=prottop&blast_rank=128&RID=) uncharacterized protein LOC113492216 isoform X1 [T... [300](#XP_026725384.1) 3e-84

[XP_018024847.1](https://www.ncbi.nlm.nih.gov/protein/XP_018024847.1?report=genbank&log$=prottop&blast_rank=129&RID=) PREDICTED: peroxidase-like [Hyalella azteca] [293](#XP_018024847.1) 3e-84

[EEC08358.1](https://www.ncbi.nlm.nih.gov/protein/EEC08358.1?report=genbank&log$=prottop&blast_rank=130&RID=) peroxinectin, putative [Ixodes scapularis] [285](#EEC08358.1) 4e-84

[KAA0198233.1](https://www.ncbi.nlm.nih.gov/protein/KAA0198233.1?report=genbank&log$=prottop&blast_rank=131&RID=) Peroxinectin-like [Hyalella azteca] [289](#KAA0198233.1) 4e-84

[GFG35367.1](https://www.ncbi.nlm.nih.gov/protein/GFG35367.1?report=genbank&log$=prottop&blast_rank=132&RID=) hypothetical protein Cfor_10110 [Coptotermes formosanus] [299](#GFG35367.1) 4e-84

[XP_025832243.1](https://www.ncbi.nlm.nih.gov/protein/XP_025832243.1?report=genbank&log$=prottop&blast_rank=133&RID=) chorion peroxidase isoform X2 [Agrilus planipennis] [287](#XP_025832243.1) 5e-84

[XP_028967949.1](https://www.ncbi.nlm.nih.gov/protein/XP_028967949.1?report=genbank&log$=prottop&blast_rank=134&RID=) peroxidase [Galendromus occidentalis] [289](#XP_028967949.1) 6e-84

[XP_001946672.2](https://www.ncbi.nlm.nih.gov/protein/XP_001946672.2?report=genbank&log$=prottop&blast_rank=135&RID=) peroxidase isoform X2 [Acyrthosiphon pisum] [292](#XP_001946672.2) 6e-84

[KAF7496118.1](https://www.ncbi.nlm.nih.gov/protein/KAF7496118.1?report=genbank&log$=prottop&blast_rank=136&RID=) Chorion peroxidase [Sarcoptes scabiei] [287](#KAF7496118.1) 6e-84

[XP_002426138.1](https://www.ncbi.nlm.nih.gov/protein/XP_002426138.1?report=genbank&log$=prottop&blast_rank=137&RID=) conserved hypothetical protein [Pediculus humanus ... [298](#XP_002426138.1) 7e-84

[XP_023243052.1](https://www.ncbi.nlm.nih.gov/protein/XP_023243052.1?report=genbank&log$=prottop&blast_rank=138&RID=) chorion peroxidase-like [Centruroides sculpturatus] [288](#XP_023243052.1) 7e-84

[XP_022650761.1](https://www.ncbi.nlm.nih.gov/protein/XP_022650761.1?report=genbank&log$=prottop&blast_rank=139&RID=) peroxidase-like isoform X2 [Varroa destructor] [288](#XP_022650761.1) 7e-84

[XP_021935610.1](https://www.ncbi.nlm.nih.gov/protein/XP_021935610.1?report=genbank&log$=prottop&blast_rank=140&RID=) uncharacterized protein LOC110837612 [Zootermopsis... [299](#XP_021935610.1) 7e-84

[XP_014275345.1](https://www.ncbi.nlm.nih.gov/protein/XP_014275345.1?report=genbank&log$=prottop&blast_rank=141&RID=) peroxidase-like isoform X1 [Halyomorpha halys] [292](#XP_014275345.1) 7e-84

[XP_030545812.1](https://www.ncbi.nlm.nih.gov/protein/XP_030545812.1?report=genbank&log$=prottop&blast_rank=142&RID=) peroxidase-like [Rhodamnia argentea] [290](#XP_030545812.1) 8e-84

[RWS06153.1](https://www.ncbi.nlm.nih.gov/protein/RWS06153.1?report=genbank&log$=prottop&blast_rank=143&RID=) Peroxidase-like protein [Dinothrombium tinctorium] [286](#RWS06153.1) 1e-83

[XP_013778012.1](https://www.ncbi.nlm.nih.gov/protein/XP_013778012.1?report=genbank&log$=prottop&blast_rank=144&RID=) chorion peroxidase-like [Limulus polyphemus] [289](#XP_013778012.1) 1e-83

[XP_028032358.1](https://www.ncbi.nlm.nih.gov/protein/XP_028032358.1?report=genbank&log$=prottop&blast_rank=145&RID=) chorion peroxidase-like [Bombyx mandarina] [295](#XP_028032358.1) 2e-83

[XP_011297271.1](https://www.ncbi.nlm.nih.gov/protein/XP_011297271.1?report=genbank&log$=prottop&blast_rank=146&RID=) PREDICTED: peroxidase [Fopius arisanus] [288](#XP_011297271.1) 3e-83

[KZS19004.1](https://www.ncbi.nlm.nih.gov/protein/KZS19004.1?report=genbank&log$=prottop&blast_rank=147&RID=) Uncharacterized protein APZ42_014696 [Daphnia magna] [290](#KZS19004.1) 3e-83

[OWF40679.1](https://www.ncbi.nlm.nih.gov/protein/OWF40679.1?report=genbank&log$=prottop&blast_rank=148&RID=) Myeloperoxidase [Mizuhopecten yessoensis] [296](#OWF40679.1) 3e-83

[OXA43787.1](https://www.ncbi.nlm.nih.gov/protein/OXA43787.1?report=genbank&log$=prottop&blast_rank=149&RID=) Chorion peroxidase [Folsomia candida] [291](#OXA43787.1) 3e-83

[KFM65699.1](https://www.ncbi.nlm.nih.gov/protein/KFM65699.1?report=genbank&log$=prottop&blast_rank=150&RID=) Chorion peroxidase [Stegodyphus mimosarum] [286](#KFM65699.1) 3e-83

[XP_034310731.1](https://www.ncbi.nlm.nih.gov/protein/XP_034310731.1?report=genbank&log$=prottop&blast_rank=151&RID=) eosinophil peroxidase isoform X1 [Crassostrea gigas] [286](#XP_034310731.1) 3e-83

[EFX81795.1](https://www.ncbi.nlm.nih.gov/protein/EFX81795.1?report=genbank&log$=prottop&blast_rank=152&RID=) hypothetical protein DAPPUDRAFT_196036 [Daphnia pulex] [287](#EFX81795.1) 3e-83

[GBM04516.1](https://www.ncbi.nlm.nih.gov/protein/GBM04516.1?report=genbank&log$=prottop&blast_rank=153&RID=) Chorion peroxidase [Araneus ventricosus] [286](#GBM04516.1) 3e-83

[XP_032798431.1](https://www.ncbi.nlm.nih.gov/protein/XP_032798431.1?report=genbank&log$=prottop&blast_rank=154&RID=) chorion peroxidase-like [Daphnia magna] [290](#XP_032798431.1) 3e-83

[XP_023288991.1](https://www.ncbi.nlm.nih.gov/protein/XP_023288991.1?report=genbank&log$=prottop&blast_rank=155&RID=) uncharacterized protein LOC105701425 isoform X1 [O... [297](#XP_023288991.1) 3e-83

[XP_023289006.1](https://www.ncbi.nlm.nih.gov/protein/XP_023289006.1?report=genbank&log$=prottop&blast_rank=156&RID=) uncharacterized protein LOC105701425 isoform X2 [O... [297](#XP_023289006.1) 4e-83

[CAC5357316.1](https://www.ncbi.nlm.nih.gov/protein/CAC5357316.1?report=genbank&log$=prottop&blast_rank=157&RID=) PXDN [Mytilus coruscus] [285](#CAC5357316.1) 4e-83

[XP_002423001.1](https://www.ncbi.nlm.nih.gov/protein/XP_002423001.1?report=genbank&log$=prottop&blast_rank=158&RID=) conserved hypothetical protein [Pediculus humanus ... [290](#XP_002423001.1) 4e-83

[XP_028127877.1](https://www.ncbi.nlm.nih.gov/protein/XP_028127877.1?report=genbank&log$=prottop&blast_rank=159&RID=) thyroid peroxidase-like [Diabrotica virgifera virg... [294](#XP_028127877.1) 4e-83

[XP_023289007.1](https://www.ncbi.nlm.nih.gov/protein/XP_023289007.1?report=genbank&log$=prottop&blast_rank=160&RID=) uncharacterized protein LOC105701425 isoform X3 [O... [297](#XP_023289007.1) 5e-83

[RUS82931.1](https://www.ncbi.nlm.nih.gov/protein/RUS82931.1?report=genbank&log$=prottop&blast_rank=161&RID=) hypothetical protein EGW08_009315 [Elysia chlorotica] [284](#RUS82931.1) 6e-83

[XP_035214640.1](https://www.ncbi.nlm.nih.gov/protein/XP_035214640.1?report=genbank&log$=prottop&blast_rank=162&RID=) peroxidase-like [Stegodyphus dumicola] [286](#XP_035214640.1) 6e-83

[GAU92502.1](https://www.ncbi.nlm.nih.gov/protein/GAU92502.1?report=genbank&log$=prottop&blast_rank=163&RID=) hypothetical protein RvY_04578 [Ramazzottius varieorna... [292](#GAU92502.1) 7e-83

[KZS09340.1](https://www.ncbi.nlm.nih.gov/protein/KZS09340.1?report=genbank&log$=prottop&blast_rank=164&RID=) Chorion peroxidase [Daphnia magna] [285](#KZS09340.1) 7e-83

[XP_032792837.1](https://www.ncbi.nlm.nih.gov/protein/XP_032792837.1?report=genbank&log$=prottop&blast_rank=165&RID=) peroxidase-like [Daphnia magna] [285](#XP_032792837.1) 8e-83

[XP_033756913.1](https://www.ncbi.nlm.nih.gov/protein/XP_033756913.1?report=genbank&log$=prottop&blast_rank=166&RID=) chorion peroxidase-like [Pecten maximus] [287](#XP_033756913.1) 8e-83

[XP_015113381.1](https://www.ncbi.nlm.nih.gov/protein/XP_015113381.1?report=genbank&log$=prottop&blast_rank=167&RID=) chorion peroxidase [Diachasma alloeum] [287](#XP_015113381.1) 8e-83

[XP_021921010.1](https://www.ncbi.nlm.nih.gov/protein/XP_021921010.1?report=genbank&log$=prottop&blast_rank=168&RID=) peroxidase-like [Zootermopsis nevadensis] [289](#XP_021921010.1) 9e-83

[XP_033760036.1](https://www.ncbi.nlm.nih.gov/protein/XP_033760036.1?report=genbank&log$=prottop&blast_rank=169&RID=) myeloperoxidase-like [Pecten maximus] [285](#XP_033760036.1) 1e-82

[TRY75389.1](https://www.ncbi.nlm.nih.gov/protein/TRY75389.1?report=genbank&log$=prottop&blast_rank=170&RID=) hypothetical protein TCAL_06346 [Tigriopus californicus] [289](#TRY75389.1) 1e-82

[EFX81608.1](https://www.ncbi.nlm.nih.gov/protein/EFX81608.1?report=genbank&log$=prottop&blast_rank=171&RID=) hypothetical protein DAPPUDRAFT_317232 [Daphnia pulex] [283](#EFX81608.1) 1e-82

[XP_031335618.1](https://www.ncbi.nlm.nih.gov/protein/XP_031335618.1?report=genbank&log$=prottop&blast_rank=172&RID=) peroxidase mlt-7 [Photinus pyralis] [286](#XP_031335618.1) 1e-82

[XP_033757668.1](https://www.ncbi.nlm.nih.gov/protein/XP_033757668.1?report=genbank&log$=prottop&blast_rank=173&RID=) peroxidase-like protein [Pecten maximus] [286](#XP_033757668.1) 1e-82

[XP_014367795.1](https://www.ncbi.nlm.nih.gov/protein/XP_014367795.1?report=genbank&log$=prottop&blast_rank=174&RID=) PREDICTED: chorion peroxidase-like isoform X2 [Pap... [292](#XP_014367795.1) 1e-82

[XP_025832242.1](https://www.ncbi.nlm.nih.gov/protein/XP_025832242.1?report=genbank&log$=prottop&blast_rank=175&RID=) chorion peroxidase isoform X1 [Agrilus planipennis] [286](#XP_025832242.1) 1e-82

[XP_021946283.1](https://www.ncbi.nlm.nih.gov/protein/XP_021946283.1?report=genbank&log$=prottop&blast_rank=176&RID=) peroxidasin homolog [Folsomia candida] [286](#XP_021946283.1) 2e-82

[XP_030755866.1](https://www.ncbi.nlm.nih.gov/protein/XP_030755866.1?report=genbank&log$=prottop&blast_rank=177&RID=) peroxidase-like [Sitophilus oryzae] [286](#XP_030755866.1) 3e-82

[XP_019930628.2](https://www.ncbi.nlm.nih.gov/protein/XP_019930628.2?report=genbank&log$=prottop&blast_rank=178&RID=) peroxidase [Crassostrea gigas] [283](#XP_019930628.2) 3e-82

[XP_018332236.1](https://www.ncbi.nlm.nih.gov/protein/XP_018332236.1?report=genbank&log$=prottop&blast_rank=179&RID=) peroxidasin [Agrilus planipennis] [285](#XP_018332236.1) 3e-82

[OXA58944.1](https://www.ncbi.nlm.nih.gov/protein/OXA58944.1?report=genbank&log$=prottop&blast_rank=180&RID=) Peroxidasin [Folsomia candida] [287](#OXA58944.1) 3e-82

[XP_027205136.1](https://www.ncbi.nlm.nih.gov/protein/XP_027205136.1?report=genbank&log$=prottop&blast_rank=181&RID=) peroxidase-like [Dermatophagoides pteronyssinus] [289](#XP_027205136.1) 3e-82

[XP_014241110.1](https://www.ncbi.nlm.nih.gov/protein/XP_014241110.1?report=genbank&log$=prottop&blast_rank=182&RID=) uncharacterized protein LOC106661888 [Cimex lectul... [293](#XP_014241110.1) 4e-82

[XP_035903264.1](https://www.ncbi.nlm.nih.gov/protein/XP_035903264.1?report=genbank&log$=prottop&blast_rank=183&RID=) chorion peroxidase isoform X2 [Anopheles stephensi] [286](#XP_035903264.1) 5e-82

[XP_008194604.1](https://www.ncbi.nlm.nih.gov/protein/XP_008194604.1?report=genbank&log$=prottop&blast_rank=184&RID=) PREDICTED: chorion peroxidase isoform X3 [Triboliu... [289](#XP_008194604.1) 5e-82

[XP_008194602.1](https://www.ncbi.nlm.nih.gov/protein/XP_008194602.1?report=genbank&log$=prottop&blast_rank=185&RID=) PREDICTED: chorion peroxidase isoform X1 [Triboliu... [290](#XP_008194602.1) 6e-82

[XP_018018958.1](https://www.ncbi.nlm.nih.gov/protein/XP_018018958.1?report=genbank&log$=prottop&blast_rank=186&RID=) PREDICTED: uncharacterized protein LOC108675451 [H... [293](#XP_018018958.1) 6e-82

[XP_008194603.1](https://www.ncbi.nlm.nih.gov/protein/XP_008194603.1?report=genbank&log$=prottop&blast_rank=187&RID=) PREDICTED: chorion peroxidase isoform X2 [Triboliu... [290](#XP_008194603.1) 6e-82

[XP_012546139.2](https://www.ncbi.nlm.nih.gov/protein/XP_012546139.2?report=genbank&log$=prottop&blast_rank=188&RID=) uncharacterized protein LOC101744777 [Bombyx mori] [293](#XP_012546139.2) 6e-82

[PCG80471.1](https://www.ncbi.nlm.nih.gov/protein/PCG80471.1?report=genbank&log$=prottop&blast_rank=189&RID=) hypothetical protein B5V51_7221 [Heliothis virescens] [293](#PCG80471.1) 7e-82

[XP_033762656.1](https://www.ncbi.nlm.nih.gov/protein/XP_033762656.1?report=genbank&log$=prottop&blast_rank=190&RID=) uncharacterized protein LOC117344118 [Pecten maximus] [291](#XP_033762656.1) 7e-82

[XP_031786308.1](https://www.ncbi.nlm.nih.gov/protein/XP_031786308.1?report=genbank&log$=prottop&blast_rank=191&RID=) uncharacterized protein LOC100123757 isoform X2 [N... [292](#XP_031786308.1) 7e-82

[XP_023022972.1](https://www.ncbi.nlm.nih.gov/protein/XP_023022972.1?report=genbank&log$=prottop&blast_rank=192&RID=) peroxidasin-like protein [Leptinotarsa decemlineata] [291](#XP_023022972.1) 7e-82

[XP_001607463.4](https://www.ncbi.nlm.nih.gov/protein/XP_001607463.4?report=genbank&log$=prottop&blast_rank=193&RID=) uncharacterized protein LOC100123757 isoform X1 [N... [293](#XP_001607463.4) 7e-82

[XP_029343239.1](https://www.ncbi.nlm.nih.gov/protein/XP_029343239.1?report=genbank&log$=prottop&blast_rank=194&RID=) peroxidase isoform X1 [Acyrthosiphon pisum] [286](#XP_029343239.1) 8e-82

[XP_014367794.1](https://www.ncbi.nlm.nih.gov/protein/XP_014367794.1?report=genbank&log$=prottop&blast_rank=195&RID=) PREDICTED: uncharacterized protein LOC106718259 is... [292](#XP_014367794.1) 8e-82

[XP_035903263.1](https://www.ncbi.nlm.nih.gov/protein/XP_035903263.1?report=genbank&log$=prottop&blast_rank=196&RID=) chorion peroxidase isoform X1 [Anopheles stephensi] [286](#XP_035903263.1) 1e-81

[XP_025095126.1](https://www.ncbi.nlm.nih.gov/protein/XP_025095126.1?report=genbank&log$=prottop&blast_rank=197&RID=) peroxidase-like protein 3 [Pomacea canaliculata] [278](#XP_025095126.1) 1e-81

[XP_011300775.1](https://www.ncbi.nlm.nih.gov/protein/XP_011300775.1?report=genbank&log$=prottop&blast_rank=198&RID=) PREDICTED: chorion peroxidase-like isoform X1 [Fop... [289](#XP_011300775.1) 1e-81

[XP_021192811.1](https://www.ncbi.nlm.nih.gov/protein/XP_021192811.1?report=genbank&log$=prottop&blast_rank=199&RID=) uncharacterized protein LOC110378050 [Helicoverpa ... [292](#XP_021192811.1) 1e-81

[XP_018341877.1](https://www.ncbi.nlm.nih.gov/protein/XP_018341877.1?report=genbank&log$=prottop&blast_rank=200&RID=) PREDICTED: chorion peroxidase [Trachymyrmex septen... [284](#XP_018341877.1) 1e-81

[XP_011300776.1](https://www.ncbi.nlm.nih.gov/protein/XP_011300776.1?report=genbank&log$=prottop&blast_rank=201&RID=) PREDICTED: chorion peroxidase-like isoform X2 [Fop... [289](#XP_011300776.1) 1e-81

[KPJ08099.1](https://www.ncbi.nlm.nih.gov/protein/KPJ08099.1?report=genbank&log$=prottop&blast_rank=202&RID=) Chorion peroxidase [Papilio machaon] [292](#KPJ08099.1) 1e-81

[XP_011065332.1](https://www.ncbi.nlm.nih.gov/protein/XP_011065332.1?report=genbank&log$=prottop&blast_rank=203&RID=) PREDICTED: peroxidase [Acromyrmex echinatior] [284](#XP_011065332.1) 2e-81

[XP_018575206.1](https://www.ncbi.nlm.nih.gov/protein/XP_018575206.1?report=genbank&log$=prottop&blast_rank=204&RID=) uncharacterized protein LOC108913999 [Anoplophora ... [291](#XP_018575206.1) 2e-81

[KPM03996.1](https://www.ncbi.nlm.nih.gov/protein/KPM03996.1?report=genbank&log$=prottop&blast_rank=205&RID=) hypothetical protein QR98_0024350 [Sarcoptes scabiei] [287](#KPM03996.1) 2e-81

[XP_014241240.1](https://www.ncbi.nlm.nih.gov/protein/XP_014241240.1?report=genbank&log$=prottop&blast_rank=206&RID=) peroxidase-like [Cimex lectularius] [285](#XP_014241240.1) 2e-81

[XP_021376270.1](https://www.ncbi.nlm.nih.gov/protein/XP_021376270.1?report=genbank&log$=prottop&blast_rank=207&RID=) peroxidase-like [Mizuhopecten yessoensis] [282](#XP_021376270.1) 2e-81

[CAC5420744.1](https://www.ncbi.nlm.nih.gov/protein/CAC5420744.1?report=genbank&log$=prottop&blast_rank=208&RID=) PXDN [Mytilus coruscus] [283](#CAC5420744.1) 2e-81

[ADF87945.1](https://www.ncbi.nlm.nih.gov/protein/ADF87945.1?report=genbank&log$=prottop&blast_rank=209&RID=) peroxinectin [Eriocheir sinensis] [283](#ADF87945.1) 2e-81

[XP_018048483.1](https://www.ncbi.nlm.nih.gov/protein/XP_018048483.1?report=genbank&log$=prottop&blast_rank=210&RID=) PREDICTED: peroxidase [Atta colombica] [283](#XP_018048483.1) 3e-81

[AIZ68326.1](https://www.ncbi.nlm.nih.gov/protein/AIZ68326.1?report=genbank&log$=prottop&blast_rank=211&RID=) peroxinectin [Caligus rogercresseyi] [283](#AIZ68326.1) 3e-81

[ASK06160.1](https://www.ncbi.nlm.nih.gov/protein/ASK06160.1?report=genbank&log$=prottop&blast_rank=212&RID=) haem peroxidase [Nilaparvata lugens] [291](#ASK06160.1) 3e-81

[XP_021000657.1](https://www.ncbi.nlm.nih.gov/protein/XP_021000657.1?report=genbank&log$=prottop&blast_rank=213&RID=) chorion peroxidase [Parasteatoda tepidariorum] [281](#XP_021000657.1) 3e-81

[XP_030550422.1](https://www.ncbi.nlm.nih.gov/protein/XP_030550422.1?report=genbank&log$=prottop&blast_rank=214&RID=) peroxidase-like [Rhodamnia argentea] [283](#XP_030550422.1) 3e-81

[XP_023309880.1](https://www.ncbi.nlm.nih.gov/protein/XP_023309880.1?report=genbank&log$=prottop&blast_rank=215&RID=) myeloperoxidase [Anoplophora glabripennis] [282](#XP_023309880.1) 4e-81

[KAF2367818.1](https://www.ncbi.nlm.nih.gov/protein/KAF2367818.1?report=genbank&log$=prottop&blast_rank=216&RID=) hypothetical protein FHG87_001410 [Trinorchestia lon... [284](#KAF2367818.1) 4e-81

[XP_011422168.2](https://www.ncbi.nlm.nih.gov/protein/XP_011422168.2?report=genbank&log$=prottop&blast_rank=217&RID=) heme peroxidase 2 [Crassostrea gigas] [284](#XP_011422168.2) 5e-81

[XP_022195983.1](https://www.ncbi.nlm.nih.gov/protein/XP_022195983.1?report=genbank&log$=prottop&blast_rank=218&RID=) uncharacterized protein LOC111053403 [Nilaparvata ... [291](#XP_022195983.1) 5e-81

[XP_014665270.1](https://www.ncbi.nlm.nih.gov/protein/XP_014665270.1?report=genbank&log$=prottop&blast_rank=219&RID=) PREDICTED: uncharacterized protein LOC106807453 [P... [290](#XP_014665270.1) 6e-81

[XP_035778075.1](https://www.ncbi.nlm.nih.gov/protein/XP_035778075.1?report=genbank&log$=prottop&blast_rank=220&RID=) chorion peroxidase-like isoform X2 [Anopheles albi... [283](#XP_035778075.1) 6e-81

[KAF5308960.1](https://www.ncbi.nlm.nih.gov/protein/KAF5308960.1?report=genbank&log$=prottop&blast_rank=221&RID=) hypothetical protein FQR65_LT00042 [Abscondita termi... [281](#KAF5308960.1) 7e-81

[KAA0198232.1](https://www.ncbi.nlm.nih.gov/protein/KAA0198232.1?report=genbank&log$=prottop&blast_rank=222&RID=) Heme binding peroxidase-like 2 [Hyalella azteca] [281](#KAA0198232.1) 7e-81

[XP_019868210.1](https://www.ncbi.nlm.nih.gov/protein/XP_019868210.1?report=genbank&log$=prottop&blast_rank=223&RID=) PREDICTED: chorion peroxidase [Aethina tumida] [281](#XP_019868210.1) 8e-81

[XP_018017997.1](https://www.ncbi.nlm.nih.gov/protein/XP_018017997.1?report=genbank&log$=prottop&blast_rank=224&RID=) PREDICTED: peroxidase-like [Hyalella azteca] [283](#XP_018017997.1) 8e-81

[XP_018316403.1](https://www.ncbi.nlm.nih.gov/protein/XP_018316403.1?report=genbank&log$=prottop&blast_rank=225&RID=) PREDICTED: chorion peroxidase [Trachymyrmex zeteki] [282](#XP_018316403.1) 8e-81

[XP_035778074.1](https://www.ncbi.nlm.nih.gov/protein/XP_035778074.1?report=genbank&log$=prottop&blast_rank=226&RID=) chorion peroxidase-like isoform X1 [Anopheles albi... [283](#XP_035778074.1) 9e-81

[RUS74145.1](https://www.ncbi.nlm.nih.gov/protein/RUS74145.1?report=genbank&log$=prottop&blast_rank=227&RID=) hypothetical protein EGW08_018100 [Elysia chlorotica] [276](#RUS74145.1) 9e-81

[OXU30832.1](https://www.ncbi.nlm.nih.gov/protein/OXU30832.1?report=genbank&log$=prottop&blast_rank=228&RID=) hypothetical protein TSAR_009871 [Trichomalopsis sarco... [290](#OXU30832.1) 1e-80

[EDO30120.1](https://www.ncbi.nlm.nih.gov/protein/EDO30120.1?report=genbank&log$=prottop&blast_rank=229&RID=) predicted protein [Nematostella vectensis] [276](#EDO30120.1) 1e-80

[ANN45955.1](https://www.ncbi.nlm.nih.gov/protein/ANN45955.1?report=genbank&log$=prottop&blast_rank=230&RID=) byssal peroxidase-like protein 1 [Mytilus coruscus] [280](#ANN45955.1) 1e-80

[XP_034944630.1](https://www.ncbi.nlm.nih.gov/protein/XP_034944630.1?report=genbank&log$=prottop&blast_rank=231&RID=) chorion peroxidase-like [Chelonus insularis] [283](#XP_034944630.1) 1e-80

[XP_031619883.1](https://www.ncbi.nlm.nih.gov/protein/XP_031619883.1?report=genbank&log$=prottop&blast_rank=232&RID=) chorion peroxidase [Contarinia nasturtii] [281](#XP_031619883.1) 1e-80

[EFX77941.1](https://www.ncbi.nlm.nih.gov/protein/EFX77941.1?report=genbank&log$=prottop&blast_rank=233&RID=) hypothetical protein DAPPUDRAFT_320790 [Daphnia pulex] [281](#EFX77941.1) 1e-80

[XP_008549528.1](https://www.ncbi.nlm.nih.gov/protein/XP_008549528.1?report=genbank&log$=prottop&blast_rank=234&RID=) PREDICTED: peroxidase-like isoform X3 [Microplitis... [281](#XP_008549528.1) 1e-80

[KAF2903413.1](https://www.ncbi.nlm.nih.gov/protein/KAF2903413.1?report=genbank&log$=prottop&blast_rank=235&RID=) hypothetical protein ILUMI_02766 [Ignelater luminosus] [288](#KAF2903413.1) 1e-80

[XP_013401628.1](https://www.ncbi.nlm.nih.gov/protein/XP_013401628.1?report=genbank&log$=prottop&blast_rank=236&RID=) peroxidasin isoform X4 [Lingula anatina] [284](#XP_013401628.1) 1e-80

[ODN02715.1](https://www.ncbi.nlm.nih.gov/protein/ODN02715.1?report=genbank&log$=prottop&blast_rank=237&RID=) Peroxidasin [Orchesella cincta] [285](#ODN02715.1) 1e-80

[XP_004536056.1](https://www.ncbi.nlm.nih.gov/protein/XP_004536056.1?report=genbank&log$=prottop&blast_rank=238&RID=) chorion peroxidase [Ceratitis capitata] [282](#XP_004536056.1) 2e-80

[AWB50958.1](https://www.ncbi.nlm.nih.gov/protein/AWB50958.1?report=genbank&log$=prottop&blast_rank=239&RID=) peroxinectin [Portunus trituberculatus] [280](#AWB50958.1) 2e-80

[XP_028165481.1](https://www.ncbi.nlm.nih.gov/protein/XP_028165481.1?report=genbank&log$=prottop&blast_rank=240&RID=) chorion peroxidase-like [Ostrinia furnacalis] [286](#XP_028165481.1) 2e-80

[KAF7286457.1](https://www.ncbi.nlm.nih.gov/protein/KAF7286457.1?report=genbank&log$=prottop&blast_rank=241&RID=) hypothetical protein GWI33_005099 [Rhynchophorus fer... [286](#KAF7286457.1) 2e-80

[XP_012055015.1](https://www.ncbi.nlm.nih.gov/protein/XP_012055015.1?report=genbank&log$=prottop&blast_rank=242&RID=) PREDICTED: peroxidase [Atta cephalotes] [281](#XP_012055015.1) 2e-80

[XP_017752530.1](https://www.ncbi.nlm.nih.gov/protein/XP_017752530.1?report=genbank&log$=prottop&blast_rank=243&RID=) PREDICTED: uncharacterized protein LOC108545399 is... [288](#XP_017752530.1) 3e-80

[KAF7286459.1](https://www.ncbi.nlm.nih.gov/protein/KAF7286459.1?report=genbank&log$=prottop&blast_rank=244&RID=) hypothetical protein GWI33_005099 [Rhynchophorus fer... [286](#KAF7286459.1) 3e-80

[XP_013169411.1](https://www.ncbi.nlm.nih.gov/protein/XP_013169411.1?report=genbank&log$=prottop&blast_rank=245&RID=) PREDICTED: chorion peroxidase-like isoform X2 [Pap... [286](#XP_013169411.1) 3e-80

[XP_017752469.1](https://www.ncbi.nlm.nih.gov/protein/XP_017752469.1?report=genbank&log$=prottop&blast_rank=246&RID=) PREDICTED: uncharacterized protein LOC108545399 is... [288](#XP_017752469.1) 3e-80

[XP_018363174.1](https://www.ncbi.nlm.nih.gov/protein/XP_018363174.1?report=genbank&log$=prottop&blast_rank=247&RID=) PREDICTED: chorion peroxidase [Trachymyrmex cornetzi] [280](#XP_018363174.1) 3e-80

[XP_309656.4](https://www.ncbi.nlm.nih.gov/protein/XP_309656.4?report=genbank&log$=prottop&blast_rank=248&RID=) AGAP003502-PA [Anopheles gambiae str. PEST] [281](#XP_309656.4) 3e-80

[KAF2896233.1](https://www.ncbi.nlm.nih.gov/protein/KAF2896233.1?report=genbank&log$=prottop&blast_rank=249&RID=) hypothetical protein ILUMI_09939 [Ignelater luminosus] [288](#KAF2896233.1) 3e-80

[KAF7286458.1](https://www.ncbi.nlm.nih.gov/protein/KAF7286458.1?report=genbank&log$=prottop&blast_rank=250&RID=) hypothetical protein GWI33_005099 [Rhynchophorus fer... [286](#KAF7286458.1) 3e-80

[EFX88723.1](https://www.ncbi.nlm.nih.gov/protein/EFX88723.1?report=genbank&log$=prottop&blast_rank=251&RID=) hypothetical protein DAPPUDRAFT_304755 [Daphnia pulex] [287](#EFX88723.1) 3e-80

[XP_017752503.1](https://www.ncbi.nlm.nih.gov/protein/XP_017752503.1?report=genbank&log$=prottop&blast_rank=252&RID=) PREDICTED: uncharacterized protein LOC108545399 is... [288](#XP_017752503.1) 4e-80

[XP_035432248.1](https://www.ncbi.nlm.nih.gov/protein/XP_035432248.1?report=genbank&log$=prottop&blast_rank=253&RID=) chorion peroxidase-like [Spodoptera frugiperda] [285](#XP_035432248.1) 4e-80

[XP_030021119.1](https://www.ncbi.nlm.nih.gov/protein/XP_030021119.1?report=genbank&log$=prottop&blast_rank=254&RID=) peroxidasin-like [Manduca sexta] [276](#XP_030021119.1) 5e-80

[KAF5299451.1](https://www.ncbi.nlm.nih.gov/protein/KAF5299451.1?report=genbank&log$=prottop&blast_rank=255&RID=) hypothetical protein FQR65_LT01031 [Abscondita termi... [280](#KAF5299451.1) 5e-80

[XP_018012596.1](https://www.ncbi.nlm.nih.gov/protein/XP_018012596.1?report=genbank&log$=prottop&blast_rank=256&RID=) PREDICTED: uncharacterized protein LOC108669703 [H... [288](#XP_018012596.1) 5e-80

[XP_029839677.1](https://www.ncbi.nlm.nih.gov/protein/XP_029839677.1?report=genbank&log$=prottop&blast_rank=257&RID=) chorion peroxidase-like isoform X1 [Ixodes scapula... [276](#XP_029839677.1) 5e-80

[XP_022291683.1](https://www.ncbi.nlm.nih.gov/protein/XP_022291683.1?report=genbank&log$=prottop&blast_rank=258&RID=) chorion peroxidase-like [Crassostrea virginica] [280](#XP_022291683.1) 5e-80

[KAA0189935.1](https://www.ncbi.nlm.nih.gov/protein/KAA0189935.1?report=genbank&log$=prottop&blast_rank=259&RID=) Heme binding peroxidase-like 3 [Hyalella azteca] [287](#KAA0189935.1) 5e-80

[XP_030022207.1](https://www.ncbi.nlm.nih.gov/protein/XP_030022207.1?report=genbank&log$=prottop&blast_rank=260&RID=) chorion peroxidase-like [Manduca sexta] [285](#XP_030022207.1) 6e-80

[XP_034941467.1](https://www.ncbi.nlm.nih.gov/protein/XP_034941467.1?report=genbank&log$=prottop&blast_rank=261&RID=) uncharacterized protein LOC118068282 [Chelonus ins... [287](#XP_034941467.1) 6e-80

[XP_029675685.1](https://www.ncbi.nlm.nih.gov/protein/XP_029675685.1?report=genbank&log$=prottop&blast_rank=262&RID=) chorion peroxidase [Formica exsecta] [280](#XP_029675685.1) 6e-80

[RXG73264.1](https://www.ncbi.nlm.nih.gov/protein/RXG73264.1?report=genbank&log$=prottop&blast_rank=263&RID=) Chorion peroxidase [Armadillidium vulgare] [276](#RXG73264.1) 6e-80

[XP_008549527.1](https://www.ncbi.nlm.nih.gov/protein/XP_008549527.1?report=genbank&log$=prottop&blast_rank=264&RID=) PREDICTED: peroxidase-like isoform X2 [Microplitis... [281](#XP_008549527.1) 7e-80

[XP_014234958.1](https://www.ncbi.nlm.nih.gov/protein/XP_014234958.1?report=genbank&log$=prottop&blast_rank=265&RID=) peroxidase-like [Trichogramma pretiosum] [282](#XP_014234958.1) 8e-80

[KAF7630826.1](https://www.ncbi.nlm.nih.gov/protein/KAF7630826.1?report=genbank&log$=prottop&blast_rank=266&RID=) hypothetical protein Mgra_00008924 [Meloidogyne gram... [284](#KAF7630826.1) 8e-80

[XP_033215658.1](https://www.ncbi.nlm.nih.gov/protein/XP_033215658.1?report=genbank&log$=prottop&blast_rank=267&RID=) uncharacterized protein LOC117172036 [Belonocnema ... [287](#XP_033215658.1) 8e-80

[XP_013169410.1](https://www.ncbi.nlm.nih.gov/protein/XP_013169410.1?report=genbank&log$=prottop&blast_rank=268&RID=) PREDICTED: uncharacterized protein LOC106119092 is... [286](#XP_013169410.1) 1e-79

[XP_032791493.1](https://www.ncbi.nlm.nih.gov/protein/XP_032791493.1?report=genbank&log$=prottop&blast_rank=269&RID=) peroxidase-like [Daphnia magna] [279](#XP_032791493.1) 1e-79

[XP_015513244.1](https://www.ncbi.nlm.nih.gov/protein/XP_015513244.1?report=genbank&log$=prottop&blast_rank=270&RID=) PREDICTED: uncharacterized protein LOC107219512 [N... [287](#XP_015513244.1) 1e-79

[XP_035206682.1](https://www.ncbi.nlm.nih.gov/protein/XP_035206682.1?report=genbank&log$=prottop&blast_rank=271&RID=) chorion peroxidase-like [Stegodyphus dumicola] [277](#XP_035206682.1) 1e-79

[XP_022293705.1](https://www.ncbi.nlm.nih.gov/protein/XP_022293705.1?report=genbank&log$=prottop&blast_rank=272&RID=) chorion peroxidase-like [Crassostrea virginica] [280](#XP_022293705.1) 1e-79

[XP_022308082.1](https://www.ncbi.nlm.nih.gov/protein/XP_022308082.1?report=genbank&log$=prottop&blast_rank=273&RID=) peroxidase-like protein 3 [Crassostrea virginica] [272](#XP_022308082.1) 1e-79

[XP_028967480.1](https://www.ncbi.nlm.nih.gov/protein/XP_028967480.1?report=genbank&log$=prottop&blast_rank=274&RID=) chorion peroxidase [Galendromus occidentalis] [277](#XP_028967480.1) 1e-79

[XP_035432488.1](https://www.ncbi.nlm.nih.gov/protein/XP_035432488.1?report=genbank&log$=prottop&blast_rank=275&RID=) uncharacterized protein LOC118264174 [Spodoptera f... [286](#XP_035432488.1) 1e-79

[RWS17093.1](https://www.ncbi.nlm.nih.gov/protein/RWS17093.1?report=genbank&log$=prottop&blast_rank=276&RID=) hypothetical protein B4U79_05995 [Dinothrombium tincto... [281](#RWS17093.1) 1e-79

[XP_008549526.1](https://www.ncbi.nlm.nih.gov/protein/XP_008549526.1?report=genbank&log$=prottop&blast_rank=277&RID=) PREDICTED: peroxidase-like isoform X1 [Microplitis... [280](#XP_008549526.1) 1e-79

[KAF7285164.1](https://www.ncbi.nlm.nih.gov/protein/KAF7285164.1?report=genbank&log$=prottop&blast_rank=278&RID=) hypothetical protein GWI33_011703 [Rhynchophorus fer... [279](#KAF7285164.1) 1e-79

[XP_022650763.1](https://www.ncbi.nlm.nih.gov/protein/XP_022650763.1?report=genbank&log$=prottop&blast_rank=279&RID=) chorion peroxidase-like isoform X4 [Varroa destruc... [274](#XP_022650763.1) 1e-79

[XP_012545875.1](https://www.ncbi.nlm.nih.gov/protein/XP_012545875.1?report=genbank&log$=prottop&blast_rank=280&RID=) peroxidase [Bombyx mori] [279](#XP_012545875.1) 1e-79

[XP_001622220.2](https://www.ncbi.nlm.nih.gov/protein/XP_001622220.2?report=genbank&log$=prottop&blast_rank=281&RID=) peroxidasin [Nematostella vectensis] [276](#XP_001622220.2) 1e-79

[CAC5420741.1](https://www.ncbi.nlm.nih.gov/protein/CAC5420741.1?report=genbank&log$=prottop&blast_rank=282&RID=) PXDN [Mytilus coruscus] [276](#CAC5420741.1) 2e-79

[XP_034116419.1](https://www.ncbi.nlm.nih.gov/protein/XP_034116419.1?report=genbank&log$=prottop&blast_rank=283&RID=) chorion peroxidase-like [Drosophila albomicans] [276](#XP_034116419.1) 2e-79

[XP_023246916.1](https://www.ncbi.nlm.nih.gov/protein/XP_023246916.1?report=genbank&log$=prottop&blast_rank=284&RID=) peroxidasin homolog [Copidosoma floridanum] [286](#XP_023246916.1) 2e-79

[XP_023343852.1](https://www.ncbi.nlm.nih.gov/protein/XP_023343852.1?report=genbank&log$=prottop&blast_rank=285&RID=) peroxidase-like [Eurytemora affinis] [278](#XP_023343852.1) 2e-79

[RWS11097.1](https://www.ncbi.nlm.nih.gov/protein/RWS11097.1?report=genbank&log$=prottop&blast_rank=286&RID=) Peroxidase-like protein [Dinothrombium tinctorium] [275](#RWS11097.1) 2e-79

[XP_029671830.1](https://www.ncbi.nlm.nih.gov/protein/XP_029671830.1?report=genbank&log$=prottop&blast_rank=287&RID=) uncharacterized protein LOC115240676 isoform X1 [F... [286](#XP_029671830.1) 2e-79

[XP_022830589.1](https://www.ncbi.nlm.nih.gov/protein/XP_022830589.1?report=genbank&log$=prottop&blast_rank=288&RID=) uncharacterized protein LOC111359323 [Spodoptera l... [286](#XP_022830589.1) 2e-79

[XP_022242090.1](https://www.ncbi.nlm.nih.gov/protein/XP_022242090.1?report=genbank&log$=prottop&blast_rank=289&RID=) chorion peroxidase-like [Limulus polyphemus] [277](#XP_022242090.1) 2e-79

[KPJ02570.1](https://www.ncbi.nlm.nih.gov/protein/KPJ02570.1?report=genbank&log$=prottop&blast_rank=290&RID=) Chorion peroxidase [Papilio xuthus] [285](#KPJ02570.1) 2e-79

[XP_036053079.1](https://www.ncbi.nlm.nih.gov/protein/XP_036053079.1?report=genbank&log$=prottop&blast_rank=291&RID=) myeloperoxidase [Onychomys torridus] [276](#XP_036053079.1) 2e-79

[XP_029671831.1](https://www.ncbi.nlm.nih.gov/protein/XP_029671831.1?report=genbank&log$=prottop&blast_rank=292&RID=) uncharacterized protein LOC115240676 isoform X2 [F... [285](#XP_029671831.1) 2e-79

[XP_012280985.1](https://www.ncbi.nlm.nih.gov/protein/XP_012280985.1?report=genbank&log$=prottop&blast_rank=293&RID=) uncharacterized protein LOC105700025 isoform X1 [O... [286](#XP_012280985.1) 2e-79

[XP_031332480.1](https://www.ncbi.nlm.nih.gov/protein/XP_031332480.1?report=genbank&log$=prottop&blast_rank=294&RID=) chorion peroxidase [Photinus pyralis] [278](#XP_031332480.1) 2e-79

[XP_023290618.1](https://www.ncbi.nlm.nih.gov/protein/XP_023290618.1?report=genbank&log$=prottop&blast_rank=295&RID=) uncharacterized protein LOC105700025 isoform X2 [O... [286](#XP_023290618.1) 2e-79

[EEC10299.1](https://www.ncbi.nlm.nih.gov/protein/EEC10299.1?report=genbank&log$=prottop&blast_rank=296&RID=) peroxinectin, putative [Ixodes scapularis] [278](#EEC10299.1) 3e-79

[XP_018010131.1](https://www.ncbi.nlm.nih.gov/protein/XP_018010131.1?report=genbank&log$=prottop&blast_rank=297&RID=) PREDICTED: peroxidase-like [Hyalella azteca] [281](#XP_018010131.1) 3e-79

[XP_022288923.1](https://www.ncbi.nlm.nih.gov/protein/XP_022288923.1?report=genbank&log$=prottop&blast_rank=298&RID=) peroxidasin-like [Crassostrea virginica] [275](#XP_022288923.1) 3e-79

[XP_013103774.1](https://www.ncbi.nlm.nih.gov/protein/XP_013103774.1?report=genbank&log$=prottop&blast_rank=299&RID=) PREDICTED: uncharacterized protein LOC106084563 [S... [286](#XP_013103774.1) 3e-79

[ETN62073.1](https://www.ncbi.nlm.nih.gov/protein/ETN62073.1?report=genbank&log$=prottop&blast_rank=300&RID=) chorion peroxidase [Anopheles darlingi] [277](#ETN62073.1) 3e-79

[KAF7490862.1](https://www.ncbi.nlm.nih.gov/protein/KAF7490862.1?report=genbank&log$=prottop&blast_rank=301&RID=) Chorion peroxidase [Sarcoptes scabiei] [277](#KAF7490862.1) 4e-79

[XP_029848717.1](https://www.ncbi.nlm.nih.gov/protein/XP_029848717.1?report=genbank&log$=prottop&blast_rank=302&RID=) chorion peroxidase [Ixodes scapularis] [277](#XP_029848717.1) 4e-79

[XP_024874317.1](https://www.ncbi.nlm.nih.gov/protein/XP_024874317.1?report=genbank&log$=prottop&blast_rank=303&RID=) uncharacterized protein LOC112456170 [Temnothorax ... [285](#XP_024874317.1) 4e-79

[XP_034335319.1](https://www.ncbi.nlm.nih.gov/protein/XP_034335319.1?report=genbank&log$=prottop&blast_rank=304&RID=) chorion peroxidase-like [Crassostrea gigas] [278](#XP_034335319.1) 5e-79

[XP_033222693.1](https://www.ncbi.nlm.nih.gov/protein/XP_033222693.1?report=genbank&log$=prottop&blast_rank=305&RID=) uncharacterized protein LOC117176547 [Belonocnema ... [285](#XP_033222693.1) 5e-79

[XP_034937754.1](https://www.ncbi.nlm.nih.gov/protein/XP_034937754.1?report=genbank&log$=prottop&blast_rank=306&RID=) peroxidasin homolog [Chelonus insularis] [276](#XP_034937754.1) 5e-79

[KZS15559.1](https://www.ncbi.nlm.nih.gov/protein/KZS15559.1?report=genbank&log$=prottop&blast_rank=307&RID=) Dual oxidase 2 [Daphnia magna] [285](#KZS15559.1) 6e-79

[KAF0773711.1](https://www.ncbi.nlm.nih.gov/protein/KAF0773711.1?report=genbank&log$=prottop&blast_rank=308&RID=) chorion peroxidase-like [Aphis craccivora] [270](#KAF0773711.1) 6e-79

[XP_013401627.1](https://www.ncbi.nlm.nih.gov/protein/XP_013401627.1?report=genbank&log$=prottop&blast_rank=309&RID=) peroxidasin homolog isoform X3 [Lingula anatina] [283](#XP_013401627.1) 6e-79

[ETN58535.1](https://www.ncbi.nlm.nih.gov/protein/ETN58535.1?report=genbank&log$=prottop&blast_rank=310&RID=) oxidase/peroxidase [Anopheles darlingi] [278](#ETN58535.1) 6e-79

[EFX84548.1](https://www.ncbi.nlm.nih.gov/protein/EFX84548.1?report=genbank&log$=prottop&blast_rank=311&RID=) hypothetical protein DAPPUDRAFT_314899 [Daphnia pulex] [271](#EFX84548.1) 6e-79

[XP_017781292.1](https://www.ncbi.nlm.nih.gov/protein/XP_017781292.1?report=genbank&log$=prottop&blast_rank=312&RID=) PREDICTED: thyroid peroxidase-like [Nicrophorus ve... [283](#XP_017781292.1) 6e-79

[AID47197.1](https://www.ncbi.nlm.nih.gov/protein/AID47197.1?report=genbank&log$=prottop&blast_rank=313&RID=) peroxinectin [Macrophthalmus japonicus] [276](#AID47197.1) 6e-79

[XP_031825686.1](https://www.ncbi.nlm.nih.gov/protein/XP_031825686.1?report=genbank&log$=prottop&blast_rank=314&RID=) uncharacterized protein LOC116423983 isoform X1 [N... [284](#XP_031825686.1) 7e-79

[ROT82938.1](https://www.ncbi.nlm.nih.gov/protein/ROT82938.1?report=genbank&log$=prottop&blast_rank=315&RID=) putative chorion peroxidase-like [Penaeus vannamei] [271](#ROT82938.1) 7e-79

[XP_020291060.1](https://www.ncbi.nlm.nih.gov/protein/XP_020291060.1?report=genbank&log$=prottop&blast_rank=316&RID=) uncharacterized protein LOC109858336 [Pseudomyrmex... [284](#XP_020291060.1) 7e-79

[XP_031825691.1](https://www.ncbi.nlm.nih.gov/protein/XP_031825691.1?report=genbank&log$=prottop&blast_rank=317&RID=) uncharacterized protein LOC116423983 isoform X2 [N... [284](#XP_031825691.1) 8e-79

[KAF5284358.1](https://www.ncbi.nlm.nih.gov/protein/KAF5284358.1?report=genbank&log$=prottop&blast_rank=318&RID=) hypothetical protein FQR65_LT13575 [Abscondita termi... [284](#KAF5284358.1) 8e-79

[XP_022907999.1](https://www.ncbi.nlm.nih.gov/protein/XP_022907999.1?report=genbank&log$=prottop&blast_rank=319&RID=) chorion peroxidase [Onthophagus taurus] [276](#XP_022907999.1) 8e-79

[XP_032778842.1](https://www.ncbi.nlm.nih.gov/protein/XP_032778842.1?report=genbank&log$=prottop&blast_rank=320&RID=) uncharacterized protein LOC116917487 [Daphnia magna] [284](#XP_032778842.1) 8e-79

[XP_013401625.1](https://www.ncbi.nlm.nih.gov/protein/XP_013401625.1?report=genbank&log$=prottop&blast_rank=321&RID=) peroxidasin homolog isoform X1 [Lingula anatina] [283](#XP_013401625.1) 9e-79

[XP_022648139.1](https://www.ncbi.nlm.nih.gov/protein/XP_022648139.1?report=genbank&log$=prottop&blast_rank=322&RID=) chorion peroxidase-like isoform X5 [Varroa destruc... [283](#XP_022648139.1) 9e-79

[XP_027204018.1](https://www.ncbi.nlm.nih.gov/protein/XP_027204018.1?report=genbank&log$=prottop&blast_rank=323&RID=) chorion peroxidase-like [Dermatophagoides pteronys... [275](#XP_027204018.1) 1e-78

[XP_029839678.1](https://www.ncbi.nlm.nih.gov/protein/XP_029839678.1?report=genbank&log$=prottop&blast_rank=324&RID=) chorion peroxidase-like isoform X2 [Ixodes scapula... [276](#XP_029839678.1) 1e-78

[XP_035303436.1](https://www.ncbi.nlm.nih.gov/protein/XP_035303436.1?report=genbank&log$=prottop&blast_rank=325&RID=) myeloperoxidase [Cricetulus griseus] [274](#XP_035303436.1) 1e-78

[XP_012152649.1](https://www.ncbi.nlm.nih.gov/protein/XP_012152649.1?report=genbank&log$=prottop&blast_rank=326&RID=) PREDICTED: uncharacterized protein LOC100875470 [M... [283](#XP_012152649.1) 1e-78

[KAF5280247.1](https://www.ncbi.nlm.nih.gov/protein/KAF5280247.1?report=genbank&log$=prottop&blast_rank=327&RID=) hypothetical protein FQA39_LY18070 [Lamprigera yunnana] [283](#KAF5280247.1) 1e-78

[KYM82732.1](https://www.ncbi.nlm.nih.gov/protein/KYM82732.1?report=genbank&log$=prottop&blast_rank=328&RID=) Chorion peroxidase [Atta colombica] [276](#KYM82732.1) 1e-78

[XP_023719849.2](https://www.ncbi.nlm.nih.gov/protein/XP_023719849.2?report=genbank&log$=prottop&blast_rank=329&RID=) uncharacterized protein LOC111871180 [Cryptotermes... [284](#XP_023719849.2) 1e-78

[XP_015923131.1](https://www.ncbi.nlm.nih.gov/protein/XP_015923131.1?report=genbank&log$=prottop&blast_rank=330&RID=) thyroid peroxidase-like [Parasteatoda tepidariorum] [282](#XP_015923131.1) 1e-78

[XP_020294335.1](https://www.ncbi.nlm.nih.gov/protein/XP_020294335.1?report=genbank&log$=prottop&blast_rank=331&RID=) uncharacterized protein LOC109859976 isoform X1 [P... [284](#XP_020294335.1) 1e-78

[XP_003401346.1](https://www.ncbi.nlm.nih.gov/protein/XP_003401346.1?report=genbank&log$=prottop&blast_rank=332&RID=) uncharacterized protein LOC100646756 isoform X1 [B... [283](#XP_003401346.1) 1e-78

[CRK94469.1](https://www.ncbi.nlm.nih.gov/protein/CRK94469.1?report=genbank&log$=prottop&blast_rank=333&RID=) CLUMA_CG007975, isoform A [Clunio marinus] [276](#CRK94469.1) 1e-78

[XP_012172522.1](https://www.ncbi.nlm.nih.gov/protein/XP_012172522.1?report=genbank&log$=prottop&blast_rank=334&RID=) uncharacterized protein LOC100646756 isoform X2 [B... [283](#XP_012172522.1) 2e-78

[KYN39525.1](https://www.ncbi.nlm.nih.gov/protein/KYN39525.1?report=genbank&log$=prottop&blast_rank=335&RID=) Chorion peroxidase [Trachymyrmex septentrionalis] [276](#KYN39525.1) 2e-78

[XP_019920120.2](https://www.ncbi.nlm.nih.gov/protein/XP_019920120.2?report=genbank&log$=prottop&blast_rank=336&RID=) peroxidase-like protein isoform X1 [Crassostrea gi... [271](#XP_019920120.2) 2e-78

[XP_023289009.1](https://www.ncbi.nlm.nih.gov/protein/XP_023289009.1?report=genbank&log$=prottop&blast_rank=337&RID=) uncharacterized protein LOC105701425 isoform X4 [O... [283](#XP_023289009.1) 2e-78

[XP_023013946.1](https://www.ncbi.nlm.nih.gov/protein/XP_023013946.1?report=genbank&log$=prottop&blast_rank=338&RID=) chorion peroxidase isoform X2 [Leptinotarsa deceml... [275](#XP_023013946.1) 2e-78

[XP_033350565.1](https://www.ncbi.nlm.nih.gov/protein/XP_033350565.1?report=genbank&log$=prottop&blast_rank=339&RID=) uncharacterized protein LOC117233934 [Bombus vosne... [283](#XP_033350565.1) 2e-78

[XP_029039457.1](https://www.ncbi.nlm.nih.gov/protein/XP_029039457.1?report=genbank&log$=prottop&blast_rank=340&RID=) uncharacterized protein LOC114874394 isoform X1 [O... [282](#XP_029039457.1) 2e-78

[KAF7395046.1](https://www.ncbi.nlm.nih.gov/protein/KAF7395046.1?report=genbank&log$=prottop&blast_rank=341&RID=) hypothetical protein [Vespula vulgaris] [283](#KAF7395046.1) 2e-78

[XP_020294336.1](https://www.ncbi.nlm.nih.gov/protein/XP_020294336.1?report=genbank&log$=prottop&blast_rank=342&RID=) uncharacterized protein LOC109859976 isoform X2 [P... [283](#XP_020294336.1) 2e-78

[TGZ51157.1](https://www.ncbi.nlm.nih.gov/protein/TGZ51157.1?report=genbank&log$=prottop&blast_rank=343&RID=) Peroxidasin [Temnothorax longispinosus] [282](#TGZ51157.1) 2e-78

[GFG33352.1](https://www.ncbi.nlm.nih.gov/protein/GFG33352.1?report=genbank&log$=prottop&blast_rank=344&RID=) hypothetical protein Cfor_04063 [Coptotermes formosanus] [272](#GFG33352.1) 3e-78

[XP_029039459.1](https://www.ncbi.nlm.nih.gov/protein/XP_029039459.1?report=genbank&log$=prottop&blast_rank=345&RID=) uncharacterized protein LOC114874394 isoform X3 [O... [282](#XP_029039459.1) 3e-78

[XP_033191019.1](https://www.ncbi.nlm.nih.gov/protein/XP_033191019.1?report=genbank&log$=prottop&blast_rank=346&RID=) uncharacterized protein LOC117157238 [Bombus vanco... [282](#XP_033191019.1) 3e-78

[XP_022693151.1](https://www.ncbi.nlm.nih.gov/protein/XP_022693151.1?report=genbank&log$=prottop&blast_rank=347&RID=) chorion peroxidase-like isoform X7 [Varroa jacobsoni] [281](#XP_022693151.1) 3e-78

[XP_015595581.1](https://www.ncbi.nlm.nih.gov/protein/XP_015595581.1?report=genbank&log$=prottop&blast_rank=348&RID=) uncharacterized protein LOC107267903 isoform X1 [C... [283](#XP_015595581.1) 3e-78

[XP_022693150.1](https://www.ncbi.nlm.nih.gov/protein/XP_022693150.1?report=genbank&log$=prottop&blast_rank=349&RID=) chorion peroxidase-like isoform X6 [Varroa jacobsoni] [282](#XP_022693150.1) 3e-78

[XP_012153486.1](https://www.ncbi.nlm.nih.gov/protein/XP_012153486.1?report=genbank&log$=prottop&blast_rank=350&RID=) PREDICTED: uncharacterized protein LOC105664258 is... [282](#XP_012153486.1) 3e-78

[XP_032523882.1](https://www.ncbi.nlm.nih.gov/protein/XP_032523882.1?report=genbank&log$=prottop&blast_rank=351&RID=) heme peroxidase 2 [Danaus plexippus plexippus] [281](#XP_032523882.1) 3e-78

[XP_036149555.1](https://www.ncbi.nlm.nih.gov/protein/XP_036149555.1?report=genbank&log$=prottop&blast_rank=352&RID=) LOW QUALITY PROTEIN: uncharacterized protein LOC10... [282](#XP_036149555.1) 3e-78

[XP_029039458.1](https://www.ncbi.nlm.nih.gov/protein/XP_029039458.1?report=genbank&log$=prottop&blast_rank=353&RID=) uncharacterized protein LOC114874394 isoform X2 [O... [282](#XP_029039458.1) 3e-78

[XP_013401626.1](https://www.ncbi.nlm.nih.gov/protein/XP_013401626.1?report=genbank&log$=prottop&blast_rank=354&RID=) peroxidasin homolog isoform X2 [Lingula anatina] [281](#XP_013401626.1) 4e-78

[KAF5294389.1](https://www.ncbi.nlm.nih.gov/protein/KAF5294389.1?report=genbank&log$=prottop&blast_rank=355&RID=) hypothetical protein FQR65_LT10754 [Abscondita termi... [281](#KAF5294389.1) 4e-78

[OQV26126.1](https://www.ncbi.nlm.nih.gov/protein/OQV26126.1?report=genbank&log$=prottop&blast_rank=356&RID=) Peroxidasin [Hypsibius dujardini] [280](#OQV26126.1) 4e-78

[KAF7422026.1](https://www.ncbi.nlm.nih.gov/protein/KAF7422026.1?report=genbank&log$=prottop&blast_rank=357&RID=) hypothetical protein [Vespula pensylvanica] [282](#KAF7422026.1) 4e-78

[OWR44794.1](https://www.ncbi.nlm.nih.gov/protein/OWR44794.1?report=genbank&log$=prottop&blast_rank=358&RID=) Peroxidasin protein [Danaus plexippus plexippus] [280](#OWR44794.1) 4e-78

[XP_015795697.1](https://www.ncbi.nlm.nih.gov/protein/XP_015795697.1?report=genbank&log$=prottop&blast_rank=359&RID=) peroxidase isoform X3 [Tetranychus urticae] [271](#XP_015795697.1) 4e-78

[GBP71459.1](https://www.ncbi.nlm.nih.gov/protein/GBP71459.1?report=genbank&log$=prottop&blast_rank=360&RID=) Chorion peroxidase [Eumeta japonica] [282](#GBP71459.1) 4e-78

[XP_015181423.1](https://www.ncbi.nlm.nih.gov/protein/XP_015181423.1?report=genbank&log$=prottop&blast_rank=361&RID=) PREDICTED: uncharacterized protein LOC107069022 [P... [282](#XP_015181423.1) 4e-78

[XP_033219685.1](https://www.ncbi.nlm.nih.gov/protein/XP_033219685.1?report=genbank&log$=prottop&blast_rank=362&RID=) uncharacterized protein LOC117174570 [Belonocnema ... [282](#XP_033219685.1) 4e-78

[XP_015595580.2](https://www.ncbi.nlm.nih.gov/protein/XP_015595580.2?report=genbank&log$=prottop&blast_rank=363&RID=) uncharacterized protein LOC107267903 isoform X2 [C... [282](#XP_015595580.2) 4e-78

[AZS64104.1](https://www.ncbi.nlm.nih.gov/protein/AZS64104.1?report=genbank&log$=prottop&blast_rank=364&RID=) haem peroxidase [Lygus hesperus] [273](#AZS64104.1) 4e-78

[OQV20791.1](https://www.ncbi.nlm.nih.gov/protein/OQV20791.1?report=genbank&log$=prottop&blast_rank=365&RID=) Chorion peroxidase [Hypsibius dujardini] [278](#OQV20791.1) 5e-78

[XP_012153484.1](https://www.ncbi.nlm.nih.gov/protein/XP_012153484.1?report=genbank&log$=prottop&blast_rank=366&RID=) PREDICTED: uncharacterized protein LOC105664258 is... [281](#XP_012153484.1) 5e-78

[XP_034179939.1](https://www.ncbi.nlm.nih.gov/protein/XP_034179939.1?report=genbank&log$=prottop&blast_rank=367&RID=) uncharacterized protein LOC117604198 isoform X2 [O... [281](#XP_034179939.1) 5e-78

[RZC43164.1](https://www.ncbi.nlm.nih.gov/protein/RZC43164.1?report=genbank&log$=prottop&blast_rank=368&RID=) chorion peroxidase-like [Asbolus verrucosus] [280](#RZC43164.1) 5e-78

[XP_022177224.1](https://www.ncbi.nlm.nih.gov/protein/XP_022177224.1?report=genbank&log$=prottop&blast_rank=369&RID=) chorion peroxidase-like isoform X2 [Myzus persicae] [271](#XP_022177224.1) 5e-78

[XP_034179940.1](https://www.ncbi.nlm.nih.gov/protein/XP_034179940.1?report=genbank&log$=prottop&blast_rank=370&RID=) uncharacterized protein LOC117604198 isoform X3 [O... [281](#XP_034179940.1) 5e-78

[XP_034179938.1](https://www.ncbi.nlm.nih.gov/protein/XP_034179938.1?report=genbank&log$=prottop&blast_rank=371&RID=) uncharacterized protein LOC117604198 isoform X1 [O... [281](#XP_034179938.1) 5e-78

[XP_021361140.1](https://www.ncbi.nlm.nih.gov/protein/XP_021361140.1?report=genbank&log$=prottop&blast_rank=372&RID=) peroxidase-like protein [Mizuhopecten yessoensis] [273](#XP_021361140.1) 5e-78

[XP_025015959.1](https://www.ncbi.nlm.nih.gov/protein/XP_025015959.1?report=genbank&log$=prottop&blast_rank=373&RID=) peroxidase isoform X2 [Tetranychus urticae] [271](#XP_025015959.1) 6e-78

[XP_022693148.1](https://www.ncbi.nlm.nih.gov/protein/XP_022693148.1?report=genbank&log$=prottop&blast_rank=374&RID=) uncharacterized protein LOC111262832 isoform X4 [V... [281](#XP_022693148.1) 6e-78

[XP_022286508.1](https://www.ncbi.nlm.nih.gov/protein/XP_022286508.1?report=genbank&log$=prottop&blast_rank=375&RID=) myeloperoxidase-like isoform X1 [Crassostrea virgi... [271](#XP_022286508.1) 7e-78

[KYM95318.1](https://www.ncbi.nlm.nih.gov/protein/KYM95318.1?report=genbank&log$=prottop&blast_rank=376&RID=) Peroxidasin [Cyphomyrmex costatus] [280](#KYM95318.1) 7e-78

[EFN63376.1](https://www.ncbi.nlm.nih.gov/protein/EFN63376.1?report=genbank&log$=prottop&blast_rank=377&RID=) Peroxidasin [Camponotus floridanus] [280](#EFN63376.1) 7e-78

[XP_019699110.1](https://www.ncbi.nlm.nih.gov/protein/XP_019699110.1?report=genbank&log$=prottop&blast_rank=378&RID=) peroxidase [Harpegnathos saltator] [274](#XP_019699110.1) 7e-78

[EFX81607.1](https://www.ncbi.nlm.nih.gov/protein/EFX81607.1?report=genbank&log$=prottop&blast_rank=379&RID=) hypothetical protein DAPPUDRAFT_317231 [Daphnia pulex] [270](#EFX81607.1) 7e-78

[XP_022693149.1](https://www.ncbi.nlm.nih.gov/protein/XP_022693149.1?report=genbank&log$=prottop&blast_rank=380&RID=) uncharacterized protein LOC111262832 isoform X5 [V... [281](#XP_022693149.1) 7e-78

[XP_022648134.1](https://www.ncbi.nlm.nih.gov/protein/XP_022648134.1?report=genbank&log$=prottop&blast_rank=381&RID=) uncharacterized protein LOC111244879 isoform X1 [V... [281](#XP_022648134.1) 7e-78

[XP_032791579.1](https://www.ncbi.nlm.nih.gov/protein/XP_032791579.1?report=genbank&log$=prottop&blast_rank=382&RID=) chorion peroxidase-like [Daphnia magna] [273](#XP_032791579.1) 7e-78

[XP_022648135.1](https://www.ncbi.nlm.nih.gov/protein/XP_022648135.1?report=genbank&log$=prottop&blast_rank=383&RID=) uncharacterized protein LOC111244879 isoform X2 [V... [281](#XP_022648135.1) 7e-78

[XP_006563003.1](https://www.ncbi.nlm.nih.gov/protein/XP_006563003.1?report=genbank&log$=prottop&blast_rank=384&RID=) uncharacterized protein LOC410515 [Apis mellifera] [281](#XP_006563003.1) 7e-78

[XP_022693147.1](https://www.ncbi.nlm.nih.gov/protein/XP_022693147.1?report=genbank&log$=prottop&blast_rank=385&RID=) uncharacterized protein LOC111262832 isoform X3 [V... [281](#XP_022693147.1) 8e-78

[XP_006611124.2](https://www.ncbi.nlm.nih.gov/protein/XP_006611124.2?report=genbank&log$=prottop&blast_rank=386&RID=) LOW QUALITY PROTEIN: uncharacterized protein LOC10... [281](#XP_006611124.2) 8e-78

[XP_028734066.1](https://www.ncbi.nlm.nih.gov/protein/XP_028734066.1?report=genbank&log$=prottop&blast_rank=387&RID=) myeloperoxidase [Peromyscus leucopus] [272](#XP_028734066.1) 8e-78

[XP_022693145.1](https://www.ncbi.nlm.nih.gov/protein/XP_022693145.1?report=genbank&log$=prottop&blast_rank=388&RID=) uncharacterized protein LOC111262832 isoform X1 [V... [281](#XP_022693145.1) 9e-78

[XP_012246127.1](https://www.ncbi.nlm.nih.gov/protein/XP_012246127.1?report=genbank&log$=prottop&blast_rank=389&RID=) uncharacterized protein LOC100743516 isoform X2 [B... [281](#XP_012246127.1) 9e-78

[XP_022693146.1](https://www.ncbi.nlm.nih.gov/protein/XP_022693146.1?report=genbank&log$=prottop&blast_rank=390&RID=) uncharacterized protein LOC111262832 isoform X2 [V... [281](#XP_022693146.1) 9e-78

[XP_003492825.1](https://www.ncbi.nlm.nih.gov/protein/XP_003492825.1?report=genbank&log$=prottop&blast_rank=391&RID=) uncharacterized protein LOC100743516 isoform X1 [B... [281](#XP_003492825.1) 9e-78

[GBN95850.1](https://www.ncbi.nlm.nih.gov/protein/GBN95850.1?report=genbank&log$=prottop&blast_rank=392&RID=) Chorion peroxidase [Araneus ventricosus] [267](#GBN95850.1) 9e-78

[XP_029829674.1](https://www.ncbi.nlm.nih.gov/protein/XP_029829674.1?report=genbank&log$=prottop&blast_rank=393&RID=) uncharacterized protein LOC8025910 [Ixodes scapula... [281](#XP_029829674.1) 1e-77

[KFD68253.1](https://www.ncbi.nlm.nih.gov/protein/KFD68253.1?report=genbank&log$=prottop&blast_rank=394&RID=) hypothetical protein M514_19515 [Trichuris suis] [280](#KFD68253.1) 1e-77

[XP_029824581.1](https://www.ncbi.nlm.nih.gov/protein/XP_029824581.1?report=genbank&log$=prottop&blast_rank=395&RID=) peroxidase-like isoform X4 [Ixodes scapularis] [273](#XP_029824581.1) 1e-77

[XP_031343691.1](https://www.ncbi.nlm.nih.gov/protein/XP_031343691.1?report=genbank&log$=prottop&blast_rank=396&RID=) uncharacterized protein LOC116171143 [Photinus pyr... [280](#XP_031343691.1) 1e-77

[XP_022648137.1](https://www.ncbi.nlm.nih.gov/protein/XP_022648137.1?report=genbank&log$=prottop&blast_rank=397&RID=) uncharacterized protein LOC111244879 isoform X4 [V... [281](#XP_022648137.1) 1e-77

[CRK86945.1](https://www.ncbi.nlm.nih.gov/protein/CRK86945.1?report=genbank&log$=prottop&blast_rank=398&RID=) CLUMA_CG000762, isoform A [Clunio marinus] [273](#CRK86945.1) 1e-77

[XP_015032578.1](https://www.ncbi.nlm.nih.gov/protein/XP_015032578.1?report=genbank&log$=prottop&blast_rank=399&RID=) chorion peroxidase [Drosophila willistoni] [274](#XP_015032578.1) 1e-77

[XP_015795696.1](https://www.ncbi.nlm.nih.gov/protein/XP_015795696.1?report=genbank&log$=prottop&blast_rank=400&RID=) peroxidase isoform X1 [Tetranychus urticae] [272](#XP_015795696.1) 1e-77

[XP_022648136.1](https://www.ncbi.nlm.nih.gov/protein/XP_022648136.1?report=genbank&log$=prottop&blast_rank=401&RID=) uncharacterized protein LOC111244879 isoform X3 [V... [281](#XP_022648136.1) 1e-77

[XP_023705793.1](https://www.ncbi.nlm.nih.gov/protein/XP_023705793.1?report=genbank&log$=prottop&blast_rank=402&RID=) peroxidase [Cryptotermes secundus] [276](#XP_023705793.1) 1e-77

[XP_030746842.1](https://www.ncbi.nlm.nih.gov/protein/XP_030746842.1?report=genbank&log$=prottop&blast_rank=403&RID=) chorion peroxidase-like [Sitophilus oryzae] [270](#XP_030746842.1) 1e-77

[XP_031622091.1](https://www.ncbi.nlm.nih.gov/protein/XP_031622091.1?report=genbank&log$=prottop&blast_rank=404&RID=) uncharacterized protein LOC116340035 isoform X1 [C... [281](#XP_031622091.1) 1e-77

[XP_035781522.1](https://www.ncbi.nlm.nih.gov/protein/XP_035781522.1?report=genbank&log$=prottop&blast_rank=405&RID=) chorion peroxidase-like [Anopheles albimanus] [274](#XP_035781522.1) 1e-77

[KZS09294.1](https://www.ncbi.nlm.nih.gov/protein/KZS09294.1?report=genbank&log$=prottop&blast_rank=406&RID=) Cardinal [Daphnia magna] [272](#KZS09294.1) 1e-77

[XP_018403790.1](https://www.ncbi.nlm.nih.gov/protein/XP_018403790.1?report=genbank&log$=prottop&blast_rank=407&RID=) PREDICTED: uncharacterized protein LOC108780533 [C... [280](#XP_018403790.1) 1e-77

[XP_011641998.2](https://www.ncbi.nlm.nih.gov/protein/XP_011641998.2?report=genbank&log$=prottop&blast_rank=408&RID=) uncharacterized protein LOC105430236 [Pogonomyrmex... [280](#XP_011641998.2) 1e-77

[CAB0019328.1](https://www.ncbi.nlm.nih.gov/protein/CAB0019328.1?report=genbank&log$=prottop&blast_rank=409&RID=) unnamed protein product [Nesidiocoris tenuis] [269](#CAB0019328.1) 1e-77

[XP_031622178.1](https://www.ncbi.nlm.nih.gov/protein/XP_031622178.1?report=genbank&log$=prottop&blast_rank=410&RID=) uncharacterized protein LOC116340035 isoform X2 [C... [280](#XP_031622178.1) 2e-77

[RZC31819.1](https://www.ncbi.nlm.nih.gov/protein/RZC31819.1?report=genbank&log$=prottop&blast_rank=411&RID=) uncharacterized protein BDFB_007318 [Asbolus verrucosus] [278](#RZC31819.1) 2e-77

[XP_015511362.1](https://www.ncbi.nlm.nih.gov/protein/XP_015511362.1?report=genbank&log$=prottop&blast_rank=412&RID=) PREDICTED: uncharacterized protein LOC107218115 [N... [280](#XP_015511362.1) 2e-77

[XP_030762973.1](https://www.ncbi.nlm.nih.gov/protein/XP_030762973.1?report=genbank&log$=prottop&blast_rank=413&RID=) peroxidasin homolog isoform X1 [Sitophilus oryzae] [279](#XP_030762973.1) 2e-77

[XP_026488127.1](https://www.ncbi.nlm.nih.gov/protein/XP_026488127.1?report=genbank&log$=prottop&blast_rank=414&RID=) uncharacterized protein LOC113394887 [Vanessa tame... [280](#XP_026488127.1) 2e-77

[XP_006983285.1](https://www.ncbi.nlm.nih.gov/protein/XP_006983285.1?report=genbank&log$=prottop&blast_rank=415&RID=) PREDICTED: myeloperoxidase [Peromyscus maniculatus... [271](#XP_006983285.1) 2e-77

[XP_022239909.1](https://www.ncbi.nlm.nih.gov/protein/XP_022239909.1?report=genbank&log$=prottop&blast_rank=416&RID=) peroxidase-like isoform X2 [Limulus polyphemus] [276](#XP_022239909.1) 2e-77

[XP_013772984.1](https://www.ncbi.nlm.nih.gov/protein/XP_013772984.1?report=genbank&log$=prottop&blast_rank=417&RID=) peroxidase-like isoform X1 [Limulus polyphemus] [276](#XP_013772984.1) 2e-77

[XP_017778752.1](https://www.ncbi.nlm.nih.gov/protein/XP_017778752.1?report=genbank&log$=prottop&blast_rank=418&RID=) PREDICTED: chorion peroxidase [Nicrophorus vespill... [266](#XP_017778752.1) 2e-77

[GBL77087.1](https://www.ncbi.nlm.nih.gov/protein/GBL77087.1?report=genbank&log$=prottop&blast_rank=419&RID=) Peroxidasin [Araneus ventricosus] [280](#GBL77087.1) 2e-77

[XP_035781037.1](https://www.ncbi.nlm.nih.gov/protein/XP_035781037.1?report=genbank&log$=prottop&blast_rank=420&RID=) uncharacterized protein LOC118460670 [Anopheles al... [280](#XP_035781037.1) 2e-77

[XP_030762974.1](https://www.ncbi.nlm.nih.gov/protein/XP_030762974.1?report=genbank&log$=prottop&blast_rank=421&RID=) peroxidasin homolog isoform X2 [Sitophilus oryzae] [278](#XP_030762974.1) 2e-77

[XP_015594875.1](https://www.ncbi.nlm.nih.gov/protein/XP_015594875.1?report=genbank&log$=prottop&blast_rank=422&RID=) uncharacterized protein LOC107267540 isoform X1 [C... [280](#XP_015594875.1) 3e-77

[ODN03143.1](https://www.ncbi.nlm.nih.gov/protein/ODN03143.1?report=genbank&log$=prottop&blast_rank=423&RID=) Chorion peroxidase [Orchesella cincta] [269](#ODN03143.1) 3e-77

[EDO39424.1](https://www.ncbi.nlm.nih.gov/protein/EDO39424.1?report=genbank&log$=prottop&blast_rank=424&RID=) predicted protein [Nematostella vectensis] [268](#EDO39424.1) 3e-77

[XP_011263565.1](https://www.ncbi.nlm.nih.gov/protein/XP_011263565.1?report=genbank&log$=prottop&blast_rank=425&RID=) uncharacterized protein LOC105255775 [Camponotus f... [279](#XP_011263565.1) 3e-77

[XP_018012857.1](https://www.ncbi.nlm.nih.gov/protein/XP_018012857.1?report=genbank&log$=prottop&blast_rank=426&RID=) PREDICTED: chorion peroxidase-like [Hyalella azteca] [273](#XP_018012857.1) 3e-77

[XP_022322239.1](https://www.ncbi.nlm.nih.gov/protein/XP_022322239.1?report=genbank&log$=prottop&blast_rank=427&RID=) peroxidase-like protein [Crassostrea virginica] [268](#XP_022322239.1) 3e-77

[XP_035312651.1](https://www.ncbi.nlm.nih.gov/protein/XP_035312651.1?report=genbank&log$=prottop&blast_rank=428&RID=) myeloperoxidase [Cricetulus griseus] [270](#XP_035312651.1) 3e-77

[XP_013780360.2](https://www.ncbi.nlm.nih.gov/protein/XP_013780360.2?report=genbank&log$=prottop&blast_rank=429&RID=) peroxidase-like isoform X1 [Limulus polyphemus] [271](#XP_013780360.2) 3e-77

[XP_015190723.1](https://www.ncbi.nlm.nih.gov/protein/XP_015190723.1?report=genbank&log$=prottop&blast_rank=430&RID=) PREDICTED: uncharacterized protein LOC107074126 is... [279](#XP_015190723.1) 4e-77

[XP_023243050.1](https://www.ncbi.nlm.nih.gov/protein/XP_023243050.1?report=genbank&log$=prottop&blast_rank=431&RID=) peroxidase-like [Centruroides sculpturatus] [270](#XP_023243050.1) 4e-77

[XP_003089867.1](https://www.ncbi.nlm.nih.gov/protein/XP_003089867.1?report=genbank&log$=prottop&blast_rank=432&RID=) hypothetical protein CRE_15111 [Caenorhabditis rem... [276](#XP_003089867.1) 4e-77

[CAA62752.1](https://www.ncbi.nlm.nih.gov/protein/CAA62752.1?report=genbank&log$=prottop&blast_rank=433&RID=) peroxinectin [Pacifastacus leniusculus] [272](#CAA62752.1) 4e-77

[XP_021913752.1](https://www.ncbi.nlm.nih.gov/protein/XP_021913752.1?report=genbank&log$=prottop&blast_rank=434&RID=) thyroid peroxidase-like [Zootermopsis nevadensis] [277](#XP_021913752.1) 4e-77

[KAF6029145.1](https://www.ncbi.nlm.nih.gov/protein/KAF6029145.1?report=genbank&log$=prottop&blast_rank=435&RID=) hypothetical protein EB796_012542 [Bugula neritina] [270](#KAF6029145.1) 4e-77

[CAB3373840.1](https://www.ncbi.nlm.nih.gov/protein/CAB3373840.1?report=genbank&log$=prottop&blast_rank=436&RID=) Hypothetical predicted protein [Cloeon dipterum] [280](#CAB3373840.1) 4e-77

[KAF5295133.1](https://www.ncbi.nlm.nih.gov/protein/KAF5295133.1?report=genbank&log$=prottop&blast_rank=437&RID=) hypothetical protein FQA39_LY13284 [Lamprigera yunnana] [272](#KAF5295133.1) 4e-77

[XP_024940632.1](https://www.ncbi.nlm.nih.gov/protein/XP_024940632.1?report=genbank&log$=prottop&blast_rank=438&RID=) uncharacterized protein LOC107267540 isoform X2 [C... [279](#XP_024940632.1) 4e-77

[XP_022248251.1](https://www.ncbi.nlm.nih.gov/protein/XP_022248251.1?report=genbank&log$=prottop&blast_rank=439&RID=) peroxidase-like isoform X2 [Limulus polyphemus] [271](#XP_022248251.1) 4e-77

[CAB3381413.1](https://www.ncbi.nlm.nih.gov/protein/CAB3381413.1?report=genbank&log$=prottop&blast_rank=440&RID=) Hypothetical predicted protein [Cloeon dipterum] [274](#CAB3381413.1) 4e-77

[XP_022290044.1](https://www.ncbi.nlm.nih.gov/protein/XP_022290044.1?report=genbank&log$=prottop&blast_rank=441&RID=) myeloperoxidase-like isoform X1 [Crassostrea virgi... [269](#XP_022290044.1) 4e-77

[XP_029824578.1](https://www.ncbi.nlm.nih.gov/protein/XP_029824578.1?report=genbank&log$=prottop&blast_rank=442&RID=) peroxidase-like isoform X3 [Ixodes scapularis] [273](#XP_029824578.1) 4e-77

[XP_022660030.1](https://www.ncbi.nlm.nih.gov/protein/XP_022660030.1?report=genbank&log$=prottop&blast_rank=443&RID=) peroxidase-like isoform X4 [Varroa destructor] [270](#XP_022660030.1) 4e-77

[XP_028171515.1](https://www.ncbi.nlm.nih.gov/protein/XP_028171515.1?report=genbank&log$=prottop&blast_rank=444&RID=) uncharacterized protein LOC114360871 isoform X2 [O... [278](#XP_028171515.1) 5e-77

[CAB0038966.1](https://www.ncbi.nlm.nih.gov/protein/CAB0038966.1?report=genbank&log$=prottop&blast_rank=445&RID=) unnamed protein product [Trichogramma brassicae] [279](#CAB0038966.1) 5e-77

[XP_029827448.1](https://www.ncbi.nlm.nih.gov/protein/XP_029827448.1?report=genbank&log$=prottop&blast_rank=446&RID=) peroxidase [Ixodes scapularis] [274](#XP_029827448.1) 5e-77

[XP_015190722.1](https://www.ncbi.nlm.nih.gov/protein/XP_015190722.1?report=genbank&log$=prottop&blast_rank=447&RID=) PREDICTED: uncharacterized protein LOC107074126 is... [279](#XP_015190722.1) 5e-77

[XP_022287047.1](https://www.ncbi.nlm.nih.gov/protein/XP_022287047.1?report=genbank&log$=prottop&blast_rank=448&RID=) myeloperoxidase-like [Crassostrea virginica] [270](#XP_022287047.1) 5e-77

[KAF6216391.1](https://www.ncbi.nlm.nih.gov/protein/KAF6216391.1?report=genbank&log$=prottop&blast_rank=449&RID=) hypothetical protein [Apolygus lucorum] [269](#KAF6216391.1) 5e-77

[XP_023337651.1](https://www.ncbi.nlm.nih.gov/protein/XP_023337651.1?report=genbank&log$=prottop&blast_rank=450&RID=) peroxidase-like [Eurytemora affinis] [272](#XP_023337651.1) 5e-77

[KAE9552822.1](https://www.ncbi.nlm.nih.gov/protein/KAE9552822.1?report=genbank&log$=prottop&blast_rank=451&RID=) hypothetical protein FO519_003979 [Halicephalobus sp... [279](#KAE9552822.1) 5e-77

[XP_029172750.1](https://www.ncbi.nlm.nih.gov/protein/XP_029172750.1?report=genbank&log$=prottop&blast_rank=452&RID=) uncharacterized protein LOC114941788 [Nylanderia f... [278](#XP_029172750.1) 5e-77

[XP_022290045.1](https://www.ncbi.nlm.nih.gov/protein/XP_022290045.1?report=genbank&log$=prottop&blast_rank=453&RID=) myeloperoxidase-like isoform X2 [Crassostrea virgi... [269](#XP_022290045.1) 5e-77

[XP_022660029.1](https://www.ncbi.nlm.nih.gov/protein/XP_022660029.1?report=genbank&log$=prottop&blast_rank=454&RID=) peroxidase-like isoform X3 [Varroa destructor] [270](#XP_022660029.1) 5e-77

[XP_022660028.1](https://www.ncbi.nlm.nih.gov/protein/XP_022660028.1?report=genbank&log$=prottop&blast_rank=455&RID=) peroxidase-like isoform X2 [Varroa destructor] [270](#XP_022660028.1) 5e-77

[XP_022707611.1](https://www.ncbi.nlm.nih.gov/protein/XP_022707611.1?report=genbank&log$=prottop&blast_rank=456&RID=) peroxidase-like isoform X2 [Varroa jacobsoni] [270](#XP_022707611.1) 5e-77

[XP_029850036.1](https://www.ncbi.nlm.nih.gov/protein/XP_029850036.1?report=genbank&log$=prottop&blast_rank=457&RID=) chorion peroxidase [Ixodes scapularis] [267](#XP_029850036.1) 5e-77

[XP_014237867.1](https://www.ncbi.nlm.nih.gov/protein/XP_014237867.1?report=genbank&log$=prottop&blast_rank=458&RID=) uncharacterized protein LOC106659704 [Trichogramma... [279](#XP_014237867.1) 5e-77

[XP_011418027.2](https://www.ncbi.nlm.nih.gov/protein/XP_011418027.2?report=genbank&log$=prottop&blast_rank=459&RID=) peroxidase-like protein isoform X2 [Crassostrea gi... [267](#XP_011418027.2) 5e-77

[XP_013385492.1](https://www.ncbi.nlm.nih.gov/protein/XP_013385492.1?report=genbank&log$=prottop&blast_rank=460&RID=) chorion peroxidase [Lingula anatina] [269](#XP_013385492.1) 5e-77

[XP_032676914.1](https://www.ncbi.nlm.nih.gov/protein/XP_032676914.1?report=genbank&log$=prottop&blast_rank=461&RID=) uncharacterized protein LOC116846764 [Odontomachus... [278](#XP_032676914.1) 5e-77

[XP_015127618.1](https://www.ncbi.nlm.nih.gov/protein/XP_015127618.1?report=genbank&log$=prottop&blast_rank=462&RID=) thyroid peroxidase [Diachasma alloeum] [277](#XP_015127618.1) 6e-77

[XP_026673359.1](https://www.ncbi.nlm.nih.gov/protein/XP_026673359.1?report=genbank&log$=prottop&blast_rank=463&RID=) uncharacterized protein LOC108629732 [Ceratina cal... [278](#XP_026673359.1) 6e-77

[XP_022660027.1](https://www.ncbi.nlm.nih.gov/protein/XP_022660027.1?report=genbank&log$=prottop&blast_rank=464&RID=) peroxidase-like isoform X1 [Varroa destructor] [270](#XP_022660027.1) 6e-77

[XP_025017550.1](https://www.ncbi.nlm.nih.gov/protein/XP_025017550.1?report=genbank&log$=prottop&blast_rank=465&RID=) chorion peroxidase isoform X2 [Tetranychus urticae] [277](#XP_025017550.1) 6e-77

[KAF4520680.1](https://www.ncbi.nlm.nih.gov/protein/KAF4520680.1?report=genbank&log$=prottop&blast_rank=466&RID=) hypothetical protein B566_EDAN006356 [Ephemera danica] [278](#KAF4520680.1) 6e-77

[XP_024509916.1](https://www.ncbi.nlm.nih.gov/protein/XP_024509916.1?report=genbank&log$=prottop&blast_rank=467&RID=) Peroxidasin-like protein [Strongyloides ratti] [278](#XP_024509916.1) 6e-77

[XP_014470094.1](https://www.ncbi.nlm.nih.gov/protein/XP_014470094.1?report=genbank&log$=prottop&blast_rank=468&RID=) PREDICTED: uncharacterized protein LOC106742026 [D... [278](#XP_014470094.1) 6e-77

[XP_022707610.1](https://www.ncbi.nlm.nih.gov/protein/XP_022707610.1?report=genbank&log$=prottop&blast_rank=469&RID=) peroxidase-like isoform X1 [Varroa jacobsoni] [270](#XP_022707610.1) 6e-77

[XP_015789927.1](https://www.ncbi.nlm.nih.gov/protein/XP_015789927.1?report=genbank&log$=prottop&blast_rank=470&RID=) chorion peroxidase isoform X1 [Tetranychus urticae] [277](#XP_015789927.1) 7e-77

[OQR70374.1](https://www.ncbi.nlm.nih.gov/protein/OQR70374.1?report=genbank&log$=prottop&blast_rank=471&RID=) peroxidasin-like [Tropilaelaps mercedesae] [267](#OQR70374.1) 7e-77

[XP_034830773.1](https://www.ncbi.nlm.nih.gov/protein/XP_034830773.1?report=genbank&log$=prottop&blast_rank=472&RID=) chorion peroxidase-like [Aphantopus hyperantus] [276](#XP_034830773.1) 7e-77

[XP_022289209.1](https://www.ncbi.nlm.nih.gov/protein/XP_022289209.1?report=genbank&log$=prottop&blast_rank=473&RID=) myeloperoxidase-like [Crassostrea virginica] [269](#XP_022289209.1) 7e-77

[XP_017797675.1](https://www.ncbi.nlm.nih.gov/protein/XP_017797675.1?report=genbank&log$=prottop&blast_rank=474&RID=) PREDICTED: uncharacterized protein LOC108578790 [H... [278](#XP_017797675.1) 7e-77

[XP_017785026.1](https://www.ncbi.nlm.nih.gov/protein/XP_017785026.1?report=genbank&log$=prottop&blast_rank=475&RID=) PREDICTED: uncharacterized protein LOC108568445 [N... [278](#XP_017785026.1) 7e-77

[XP_018345288.1](https://www.ncbi.nlm.nih.gov/protein/XP_018345288.1?report=genbank&log$=prottop&blast_rank=476&RID=) PREDICTED: uncharacterized protein LOC108750375 is... [278](#XP_018345288.1) 7e-77

[KOC69351.1](https://www.ncbi.nlm.nih.gov/protein/KOC69351.1?report=genbank&log$=prottop&blast_rank=477&RID=) Chorion peroxidase [Habropoda laboriosa] [278](#KOC69351.1) 7e-77

[XP_012256198.1](https://www.ncbi.nlm.nih.gov/protein/XP_012256198.1?report=genbank&log$=prottop&blast_rank=478&RID=) lactoperoxidase [Athalia rosae] [278](#XP_012256198.1) 7e-77

[XP_029169910.1](https://www.ncbi.nlm.nih.gov/protein/XP_029169910.1?report=genbank&log$=prottop&blast_rank=479&RID=) uncharacterized protein LOC114939685 [Nylanderia f... [278](#XP_029169910.1) 8e-77

[XP_029824577.1](https://www.ncbi.nlm.nih.gov/protein/XP_029824577.1?report=genbank&log$=prottop&blast_rank=480&RID=) peroxidase-like isoform X2 [Ixodes scapularis] [273](#XP_029824577.1) 8e-77

[XP_035729311.1](https://www.ncbi.nlm.nih.gov/protein/XP_035729311.1?report=genbank&log$=prottop&blast_rank=481&RID=) uncharacterized protein LOC118444789 isoform X1 [V... [278](#XP_035729311.1) 8e-77

[XP_018026833.1](https://www.ncbi.nlm.nih.gov/protein/XP_018026833.1?report=genbank&log$=prottop&blast_rank=482&RID=) PREDICTED: LOW QUALITY PROTEIN: peroxidase-like [H... [271](#XP_018026833.1) 8e-77

[KAF0313953.1](https://www.ncbi.nlm.nih.gov/protein/KAF0313953.1?report=genbank&log$=prottop&blast_rank=483&RID=) Peroxidasin [Amphibalanus amphitrite] [270](#KAF0313953.1) 8e-77

[KYN37231.1](https://www.ncbi.nlm.nih.gov/protein/KYN37231.1?report=genbank&log$=prottop&blast_rank=484&RID=) Peroxidasin [Trachymyrmex septentrionalis] [278](#KYN37231.1) 9e-77

[KYM82275.1](https://www.ncbi.nlm.nih.gov/protein/KYM82275.1?report=genbank&log$=prottop&blast_rank=485&RID=) Peroxidasin [Atta colombica] [278](#KYM82275.1) 9e-77

[XP_014357630.1](https://www.ncbi.nlm.nih.gov/protein/XP_014357630.1?report=genbank&log$=prottop&blast_rank=486&RID=) PREDICTED: uncharacterized protein LOC106710154 [P... [278](#XP_014357630.1) 9e-77

[XP_018345289.1](https://www.ncbi.nlm.nih.gov/protein/XP_018345289.1?report=genbank&log$=prottop&blast_rank=487&RID=) PREDICTED: uncharacterized protein LOC108750375 is... [278](#XP_018345289.1) 9e-77

[KYN19818.1](https://www.ncbi.nlm.nih.gov/protein/KYN19818.1?report=genbank&log$=prottop&blast_rank=488&RID=) Chorion peroxidase [Trachymyrmex cornetzi] [271](#KYN19818.1) 9e-77

[XP_035721160.1](https://www.ncbi.nlm.nih.gov/protein/XP_035721160.1?report=genbank&log$=prottop&blast_rank=489&RID=) uncharacterized protein LOC118441204 isoform X1 [V... [278](#XP_035721160.1) 9e-77

[XP_021930754.1](https://www.ncbi.nlm.nih.gov/protein/XP_021930754.1?report=genbank&log$=prottop&blast_rank=490&RID=) peroxidase-like isoform X2 [Zootermopsis nevadensis] [274](#XP_021930754.1) 9e-77

[XP_035721162.1](https://www.ncbi.nlm.nih.gov/protein/XP_035721162.1?report=genbank&log$=prottop&blast_rank=491&RID=) uncharacterized protein LOC118441204 isoform X2 [V... [278](#XP_035721162.1) 9e-77

[KAF7389163.1](https://www.ncbi.nlm.nih.gov/protein/KAF7389163.1?report=genbank&log$=prottop&blast_rank=492&RID=) hypothetical protein [Vespula vulgaris] [278](#KAF7389163.1) 9e-77

[KAB0793638.1](https://www.ncbi.nlm.nih.gov/protein/KAB0793638.1?report=genbank&log$=prottop&blast_rank=493&RID=) hypothetical protein PPYR_13258 [Photinus pyralis] [276](#KAB0793638.1) 1e-76

[XP_031354736.1](https://www.ncbi.nlm.nih.gov/protein/XP_031354736.1?report=genbank&log$=prottop&blast_rank=494&RID=) chorion peroxidase-like [Photinus pyralis] [275](#XP_031354736.1) 1e-76

[KPJ15657.1](https://www.ncbi.nlm.nih.gov/protein/KPJ15657.1?report=genbank&log$=prottop&blast_rank=495&RID=) Peroxidasin-like [Papilio machaon] [277](#KPJ15657.1) 1e-76

[XP_012056359.1](https://www.ncbi.nlm.nih.gov/protein/XP_012056359.1?report=genbank&log$=prottop&blast_rank=496&RID=) PREDICTED: uncharacterized protein LOC105619452 [A... [278](#XP_012056359.1) 1e-76

[XP_022657122.1](https://www.ncbi.nlm.nih.gov/protein/XP_022657122.1?report=genbank&log$=prottop&blast_rank=497&RID=) uncharacterized protein LOC111248667 [Varroa destr... [278](#XP_022657122.1) 1e-76

[XP_035721167.1](https://www.ncbi.nlm.nih.gov/protein/XP_035721167.1?report=genbank&log$=prottop&blast_rank=498&RID=) uncharacterized protein LOC118441204 isoform X3 [V... [278](#XP_035721167.1) 1e-76

[XP_013398465.1](https://www.ncbi.nlm.nih.gov/protein/XP_013398465.1?report=genbank&log$=prottop&blast_rank=499&RID=) myeloperoxidase-like [Lingula anatina] [268](#XP_013398465.1) 1e-76

[XP_018049119.1](https://www.ncbi.nlm.nih.gov/protein/XP_018049119.1?report=genbank&log$=prottop&blast_rank=500&RID=) PREDICTED: uncharacterized protein LOC108687711 [A... [278](#XP_018049119.1) 1e-76

>[RUS88352.1](https://www.ncbi.nlm.nih.gov/protein/RUS88352.1?report=genbank&log$=protalign&blast_rank=1&RID=0) hypothetical protein EGW08_003864, partial [Elysia chlorotica]

Length=479

Score = 523 bits (1347), Expect = 5e-178, Method: Compositional matrix adjust.

Identities = 252/482 (52%), Positives = 338/482 (70%), Gaps = 9/482 (2%)

Query 36 LDFVSEADLEHCSRLTYDQLRYRQIDGRCNHPRNYGSTGRPVKRYLRPHYQDKFGENLPR 95

+D +S D C + + RYR++DGRCNHP ++GS +PVKRY++P YQD G + PR

Sbjct 1 MDILSPGDQTPC-HMRPKKERYREMDGRCNHPLDFGSAMKPVKRYIKPQYQDPEGYDTPR 59

Query 96 VYSVTG--QLLPSPRMVSWKLHPDQTAHDNNTMLVMQMGQFIDHDITRAPELSGRNASIK 153

++SV G Q LPSPR +S +LH D+ D +TM +MQ+GQFIDHDIT AP + NASIK

Sbjct 60 MFSVRGTNQPLPSPRAISRRLHTDRNLMDRHTMWLMQLGQFIDHDITSAPVPTEHNASIK 119

Query 154 CCGVPPKERLPDCFPIDIPPGDPVFEDCMEFFRSSPAVDNDGNIIYPREQINALTSFIDG 213

CCGVPP + +CFPI +P DPVF CMEF RS P D +G I YPREQ+NALT+F+DG

Sbjct 120 CCGVPPDQVPKECFPISVPTDDPVFPPCMEFVRSEPWRDTEGKIKYPREQMNALTAFVDG 179

Query 214 SAVYGSDLDTYTWIRSENGTGVFLNTHLVHGRERLPSHPHLGPESCVSSNTAESYCQLAG 273

SA+YGSD + +R G G L T +V+G+ERLP + C++S++ S+CQL+G

Sbjct 180 SAIYGSDKEMMARLRVGGGKGALLKTVVVNGKERLPHDTQANSDMCLASHSTVSFCQLSG 239

Query 274 DMRVNEQPGLGSIHLLFHLHHNHIVRLLVAGILKKRGQPSSPERIAKFIQESSSALKEQI 333

D RVNEQPGLGS HLLFHL HN+IV+ L GIL K +P+S + ++ AL+E I

Sbjct 240 DGRVNEQPGLGSAHLLFHLFHNYIVKALTKGILGKHRRPNSELDVELYLHGEPDALQETI 299

Query 334 FQEVRKMLGAIIQKLTYCDWLPMILGPYLIDKFQLGCTRRSRYNSDLDPRVANSFLSAAL 393

FQE RK++GA++QK+TYCDWLP+ILGP LI K+ LGC RS YN +DPR+AN+FL+AA

Sbjct 300 FQESRKIVGAVLQKITYCDWLPLILGPRLIRKYSLGCHGRSSYNHFVDPRIANAFLTAAF 359

Query 394 RFGHTLIPNVYNFGDKRIHLKDTFNIPDASIRYYDNIIQCLIKEGSEEAYDRYVSSAVSE 453

RFGH+LIPN + ++ LKD F +PD + +++ I++ + GS++ +DR+V AV++

Sbjct 360 RFGHSLIPNQLSINQEKRDLKDQFMVPDNVLLHFETIMEGMQSAGSQQKFDRHVVDAVTD 419

Query 454 HLFESTRGHKHALDLIAVNIQRGRDHGIPAYHYWRQYYRLRRIISLDEFGEAGIAMKKAY 513

HLFEST+G K ALDLIA+NIQRGRDHGIP Y+ WR+YY L R+ + GI ++

Sbjct 420 HLFESTKGPKGALDLIALNIQRGRDHGIPPYYAWRKYYGLSRL------EKNGIEFRRTM 473

Query 514 RD 515

RD

Sbjct 474 RD 475

>[XP_025107474.1](https://www.ncbi.nlm.nih.gov/protein/XP_025107474.1?report=genbank&log$=protalign&blast_rank=2&RID=0) myeloperoxidase-like [Pomacea canaliculata]

Length=606

Score = 389 bits (1000), Expect = 6e-124, Method: Compositional matrix adjust.

Identities = 207/482 (43%), Positives = 299/482 (62%), Gaps = 18/482 (4%)

Query 34 GSLDFVSEADLEHCSRLTYDQLRYRQIDGRCNHPRNYGSTGRPVKRYLRPHYQDKFGENL 93

G LD + + + CS+ T ++ RYR DG CN+P N G+T +KR + P Y + G

Sbjct 63 GGLDMLQKLPRQDCSKRTTEEDRYRTYDGTCNNPGNRGATFSFLKRLVPPAYHESNG--- 119

Query 94 PRVYSVTGQLLPSPRMVSWKLHPDQTAHDNNTMLVMQMGQFIDHDITRAPELSGRNASIK 153

PR Y + LP+PR+VS +H ++ N+T+LVMQ+GQ +DHD++ + ++ +IK

Sbjct 120 PRQYGKDFKKLPNPRLVSRTIHTGKSDPSNHTLLVMQIGQLLDHDLSVIGVPTEKDKTIK 179

Query 154 CCGVPPKER---------LPDCFPIDIPPGDPVFEDCMEFFRSSPAVDNDGNIIYPREQI 204

CCGVP +R L DC PI+IPPGD F+DCMEF RS PA +G+II PR

Sbjct 180 CCGVPLDKRISVSSEPVTLHDCLPIEIPPGDLYFKDCMEFVRSEPARLRNGSIINPRRHE 239

Query 205 NALTSFIDGSAVYGSDLDTYTWIRSENGTGVFLNTHLVHGRERLPSHPHLGPESCVSSNT 264

N LTSFID S VYGSDL +R G L T G+ERLP P+ C++ +

Sbjct 240 NVLTSFIDASMVYGSDLSKVLQLRDNGGKEALLKTSFYRGKERLPQGK---PDVCIN-DG 295

Query 265 AESYCQLAGDMRVNEQPGLGSIHLLFHLHHNHIVRLLVAGILKKRGQPSSPERIAKFIQE 324

+ YC LAGD RVNEQPGL +IH +FHL HN +VR LVA IL +G+ S+P IA +I+

Sbjct 296 PDHYCALAGDDRVNEQPGLTAIHTVFHLEHNRLVRKLVADILVAQGRRSTPLDIAAYIKT 355

Query 325 SSSALKEQIFQEVRKMLGAIIQKLTYCDWLPMILGPYLIDKFQLGCTRRSRYNSDLDPRV 384

+ +KE++FQ VRK+L A+ QK+ Y ++LP+ILGP ++ KFQL R ++ +DP +

Sbjct 356 APLLVKEKLFQTVRKILIAVWQKVVYGEYLPIILGPDIMTKFQLWTGSRVNFDPHVDPSI 415

Query 385 ANSFLSAALRFGHTLIPNVYNFGDKRIHLKDTFNIPDASIRYYDNIIQCLIK-EGSEEAY 443

+N+F SAA RFGHTLI NV++ DK LKD F+ + Y+++ + L+ + E +

Sbjct 416 SNAFSSAAFRFGHTLIANVFHM-DKDHILKDMFSESSHILEKYESMCEGLVSWNNAAETF 474

Query 444 DRYVSSAVSEHLFESTRGHKHALDLIAVNIQRGRDHGIPAYHYWRQYYRLRRIISLDEFG 503

+R+++ +++ HLF + R +K LDL ++N+QRGRDHG + +R+Y+ LR FG

Sbjct 475 NRHLADSITNHLFANNRMNKTGLDLASLNLQRGRDHGTAGINAYRKYFGLRPYQISTSFG 534

Query 504 EA 505

A

Sbjct 535 TA 536

>[XP_013088542.1](https://www.ncbi.nlm.nih.gov/protein/XP_013088542.1?report=genbank&log$=protalign&blast_rank=3&RID=0) PREDICTED: lactoperoxidase-like [Biomphalaria glabrata]

Length=377

Score = 357 bits (917), Expect = 1e-114, Method: Compositional matrix adjust.

Identities = 177/350 (51%), Positives = 238/350 (68%), Gaps = 4/350 (1%)

Query 20 PDIYARRGDLGDSTGSLDFVSEADLEHCSRLTYDQLRYRQIDGRCNHPRNYGSTGRPVKR 79

PD+Y + G G + + + D C++ LRYR++DGRCNHP++YGST +P+KR

Sbjct 30 PDLYYANVN-GLIPGGYNNLIKGDQSPCTK-DIASLRYRELDGRCNHPKDYGSTLKPLKR 87

Query 80 YLRPHYQDKFGENLPRVYSVTGQL-LPSPRMVSWKLHPDQTAHDNNTMLVMQMGQFIDHD 138

YL Y D+ G N PR+YSV G + LPSPR++SWKL PD + + + +M MQ GQF+ HD

Sbjct 88 YLPADYHDEKGWNTPRLYSVVGNVALPSPRLISWKLFPDISINSDLSMFTMQFGQFLSHD 147

Query 139 ITRAPELSGRNASIKCCGVPPKERLPDCFPIDIPPGDPVFEDCMEFFRSSPAVDNDGNII 198

I AP + N +I CC V + DCFPI IP GDP F+ CMEF RS A D+DGN I

Sbjct 148 IGVAPVPTSPNHTITCCQVSSRYMNRDCFPIPIPKGDPRFQKCMEFVRSEAAKDDDGNQI 207

Query 199 YPREQINALTSFIDGSAVYGSDLDTYTWIRSENGTGVFLNTHLVHGRERLPSHPHLGPES 258

PREQ+NALTSF+D S +YGS+L T +R+ENG G L T L+HG+ERLP+ P +

Sbjct 208 NPREQLNALTSFVDSSNIYGSNLGTSLRLRTENGKGALLVTTLIHGKERLPNDTS-SPPA 266

Query 259 CVSSNTAESYCQLAGDMRVNEQPGLGSIHLLFHLHHNHIVRLLVAGILKKRGQPSSPERI 318

C+ + + SYCQL+GD RVN+QP L + HL FHL+HN+IVR L GILK++G +SP +

Sbjct 267 CLRTESPTSYCQLSGDGRVNQQPVLSTQHLSFHLYHNYIVRQLAKGILKRKGFKTSPAHV 326

Query 319 AKFIQESSSALKEQIFQEVRKMLGAIIQKLTYCDWLPMILGPYLIDKFQL 368

K+I+ S +KE +FQE RK++GAI QK+ +C++LP I+GP LI KF L

Sbjct 327 EKYIKTVSEKVKEMLFQEARKIVGAIFQKIAFCEYLPYIVGPELIVKFDL 376

>[PVD24488.1](https://www.ncbi.nlm.nih.gov/protein/PVD24488.1?report=genbank&log$=protalign&blast_rank=4&RID=0) hypothetical protein C0Q70_14971 [Pomacea canaliculata]

Length=441

Score = 359 bits (921), Expect = 2e-114, Method: Compositional matrix adjust.

Identities = 194/448 (43%), Positives = 277/448 (62%), Gaps = 10/448 (2%)

Query 182 MEFFRSSPAVDNDGNIIYPREQINALTSFIDGSAVYGSDLDTYTWIRSENGTGVFLNTHL 241

MEF RS PA +G+II PR N LTSFID S VYGSDL +R G L T

Sbjct 1 MEFVRSEPARLRNGSIINPRRHENVLTSFIDASMVYGSDLSKVLQLRDNGGKEALLKTSF 60

Query 242 VHGRERLPSHPHLGPESCVSSNTAESYCQLAGDMRVNEQPGLGSIHLLFHLHHNHIVRLL 301

G+ERLP P+ C++ + + YC LAGD RVNEQPGL +IH +FHL HN +VR L

Sbjct 61 YRGKERLPQGK---PDVCIN-DGPDHYCALAGDDRVNEQPGLTAIHTVFHLEHNRLVRKL 116

Query 302 VAGILKKRGQPSSPERIAKFIQESSSALKEQIFQEVRKMLGAIIQKLTYCDWLPMILGPY 361

VA IL +G+ S+P IA +I+ + +KE++FQ VRK+L A+ QK+ Y ++LP+ILGP

Sbjct 117 VADILVAQGRRSTPLDIAAYIKTAPLLVKEKLFQTVRKILIAVWQKVVYGEYLPIILGPD 176

Query 362 LIDKFQLGCTRRSRYNSDLDPRVANSFLSAALRFGHTLIPNVYNFGDKRIHLKDTFNIPD 421

++ KFQL R ++ +DP ++N+F SAA RFGHTLI NV++ DK LKD F+

Sbjct 177 IMTKFQLWTGSRVNFDPHVDPSISNAFSSAAFRFGHTLIANVFHM-DKDHILKDMFSESS 235

Query 422 ASIRYYDNIIQCLIK-EGSEEAYDRYVSSAVSEHLFESTRGHKHALDLIAVNIQRGRDHG 480

+ Y+++ + L+ + E ++R+++ +++ HLF + R +K LDL ++N+QRGRDHG

Sbjct 236 HILEKYESMCEGLVSWNNAAETFNRHLADSITNHLFANNRMNKTGLDLASLNLQRGRDHG 295

Query 481 IPAYHYWRQYYRLRRIISLDEFGEAGIAMKKAYRDIRDVDLFPGGLLEPSMPGGVVGETF 540

+ +R+Y+ LR FG A +++ + D DVDLF GG E S+ G+VGETF

Sbjct 296 TAGINAYRKYFGLRPYQISTSFGTA-LSIFEPSTD--DVDLFTGGTCERSVKKGIVGETF 352

Query 541 GHILANQFADLKFGDTYFFLHQQAPQGFRAAQIKAILSVTMSSIICANSAVTQAQPDPFY 600

HIL QF DLKFGD YFF GF AQ+ +I ++MS I+C+N ++ Q PF+

Sbjct 353 AHILGQQFHDLKFGDKYFFETSDQRYGFTTAQLISITKLSMSKILCSNGGISYIQEKPFH 412

Query 601 MASQLNLPR-PCSDYSEMDVEPWLIHFS 627

A+ P PC + ++D+ W+ FS

Sbjct 413 QANYYYNPLVPCDELPDIDLTYWVSEFS 440

>[XP_005110224.1](https://www.ncbi.nlm.nih.gov/protein/XP_005110224.1?report=genbank&log$=protalign&blast_rank=5&RID=0) peroxidasin isoform X1 [Aplysia californica]

Length=634

Score = 361 bits (926), Expect = 1e-112, Method: Compositional matrix adjust.

Identities = 221/582 (38%), Positives = 307/582 (53%), Gaps = 61/582 (10%)

Query 56 RYRQIDGRCNHPRNYGSTGRPVKRYLRPHYQDKFGENLPRVYSVTGQLLPSPRMVSWKLH 115

+YR IDG CN+ RN G+ +R L P Y D PR +V+G+ LPS R VS H

Sbjct 75 KYRTIDGTCNNWRNQGAARTQARRLLPPAYDDV--NQKPRQTAVSGKPLPSARAVSIAAH 132

Query 116 PDQTAHDNNTMLVMQMGQFIDHDITRAPELSGRNASIKCCG----VPPKERLPDCFPIDI 171

P T ++MQ GQFIDHD+T P + +++IKCCG +P + +CFPI +

Sbjct 133 PPTPKELGLTNMIMQWGQFIDHDVTAFPVATELDSTIKCCGSNSSIPQLCKDENCFPILL 192

Query 172 PPGDPVFE-DCMEFFRSSPAVDNDGNIIYPREQINALTSFIDGSAVYGSDLDTYTWIRSE 230

P D F CMEF RS A D GNI+ PR+QIN++TSFIDGS +YGS + +R

Sbjct 193 PANDGDFRGTCMEFVRSVAARDPQGNILNPRQQINSVTSFIDGSQIYGSSEELLKKLREP 252

Query 231 NGTGVFLNTHLVHGRERLPSHPHLGPESCVSSNTAESYCQLAGDMRVNEQPGLGSIHLLF 290

N L T L + LP E C+ + YC LAGD RVNE P L S+H L+

Sbjct 253 NS--FLLKTKL---GKFLPEATDEA-EGCILRENSNDYCFLAGDSRVNEHPALASMHTLW 306

Query 291 HLHHNHIVRLLVAGILKKRGQPSSPERIAKFIQESSSALKEQIFQEVRKMLGAIIQKLTY 350

HN I R L K R Q S+ E+IFQ RK++GA++QK+TY

Sbjct 307 MREHNRIARELA----KLRPQDST----------------EEIFQLTRKIVGALLQKITY 346

Query 351 CDWLPMILGPYLIDKFQLGCTRRSRYNSDLDPRVANSFLSAALRFGHTLIPNVYNFGDKR 410

DWLP+ILG + + RS N +DPR++NSF +A RFGH+L+P G++

Sbjct 347 NDWLPIILGTVATQGKLVSKSGRSTPNLAVDPRISNSFSTATFRFGHSLVPAEILIGERN 406

Query 411 IHLKDTFNIPDASIRYYDNII----------------QCLIKEGSEEAYDRYVSSAVSEH 454

+HL+D FN P + D+++ CL+ E DR V+ +++

Sbjct 407 VHLRDLFNRPAEVLDNLDDVLAGLAAVSRIHGPVACTSCLL-----EDVDREVAEDLTKF 461

Query 455 LFE---STRGHKHALDLIAVNIQRGRDHGIPAYHYWRQYYRLRRIISLDE--FGEAGIAM 509

LFE S RG DL+++NIQRGRDHGIP Y +R++ L + ++ G+ G +

Sbjct 462 LFEPPNSPRG--SGFDLVSLNIQRGRDHGIPPYTTFREFCNLPALTGFNDRALGQHGSRL 519

Query 510 KKAYRDIRDVDLFPGGLLEPSMPGGVVGETFGHILANQFADLKFGDTYFFLHQQAPQGFR 569

Y + D+DLF G L EP + GG++GET ++ NQF LK D +FF + GF

Sbjct 520 ATVYESVDDIDLFTGLLYEPHVYGGIIGETLMCLIGNQFIHLKSADRFFFDTSELEFGFT 579

Query 570 AAQIKAILSVTMSSIICANSAVTQAQPDPFYMASQLNLPRPC 611

Q++ I T++SI+C N + Q D F + S N C

Sbjct 580 DDQLENIRKTTLASIMCENLEMPQLVVDVFRLVSNNNKLTDC 621

>[XP_025083738.1](https://www.ncbi.nlm.nih.gov/protein/XP_025083738.1?report=genbank&log$=protalign&blast_rank=6&RID=0) chorion peroxidase-like [Pomacea canaliculata]

Length=645

Score = 351 bits (901), Expect = 1e-108, Method: Compositional matrix adjust.

Identities = 213/596 (36%), Positives = 302/596 (51%), Gaps = 46/596 (8%)

Query 47 CSRLTYDQLRYRQIDGRCNHPRNYGSTGRPVKRYLRPHYQDKFGENLPRVYSVTGQLLPS 106

C Y+ + YR DG CNHP N G++ +P+ R L Y+D G +PR++ G LP+

Sbjct 80 CDPFQYESI-YRTADGTCNHPFNLGASTKPLSRLLPARYED--GREVPRIHGKLGLPLPN 136

Query 107 PRMVSWKLHPDQTAHDNNTMLVMQMGQFIDHDITRAP-ELSGRNASIKCCGVPPKERLP- 164

R+VS +HPD T+ VMQ GQ +DHD+ P + +IKCC LP

Sbjct 137 ARLVSKVIHPDIRTSTPFTVFVMQWGQLMDHDMVSTPLPVDDTRRTIKCCSDDKTHVLPN 196

Query 165 ---DCFPIDIPPGDPVFEDCMEFFRSSPAVDNDGNIIYPREQINALTSFIDGSAVYGSDL 221

+CFPI P + CMEF RS P D G + PRE +NA+TSFID S VYGS

Sbjct 197 ASSECFPIRFGPDESFLGSCMEFVRSMPIKDKHGTVKLPREHMNAVTSFIDASVVYGSTA 256

Query 222 DTYTWIRSENGTGVFLNTHLVHGRERLPSHPHLGPESCVSSNTAESYCQLAGDMRVNEQP 281

+ +R +GTG LN V + LPS G C+ T YC LAGD RVNE P

Sbjct 257 ERLKEVRDNHGTGYLLN---VTEDDYLPSS---GSADCIRP-TKSDYCFLAGDERVNEHP 309

Query 282 GLGSIHLLFHLHHNHIVRLLVAGILKKRGQPSSPERIAKFIQESSSALKEQIFQEVRKML 341

L IH + HN I +L K +P P+ E IFQ R ++

Sbjct 310 SLTLIHTILVRLHNRIAHIL------KYMRPFYPD--------------EDIFQRARAIV 349

Query 342 GAIIQKLTYCDWLPMILGPYLIDKFQLGCT---RRSRYNSDLDPRVANSFLSAALRFGHT 398

AI QK+ Y DWLP++LG + + L +RS+Y+S LDP + SF +A RFGHT

Sbjct 350 IAITQKIMYADWLPIVLGEQTMKTYGLAVGPKFKRSKYDSLLDPTIPTSFSTAVFRFGHT 409

Query 399 LIPNVYNFGDKRIHLKDTFNIPDASIRYYDNIIQCLIKEGSEEAY----DRYVSSAVSEH 454

LIP G + L++ F P + ++ +A D ++ V+ H

Sbjct 410 LIPRSVPIGGSQRLLRELFFNPSLIKNNIEKMVDSFTIGSDPDARSQMPDTFIVEEVTNH 469

Query 455 LFESTRGHKHALDLIAVNIQRGRDHGIPAYHYWRQYYRLRRIISLDE--FGEAGIAMKKA 512

LFES G + DLIA+NIQR RDHG+P Y+ +R+Y L RI S D+ G + A+

Sbjct 470 LFESEEGIGKSFDLIALNIQRARDHGLPTYNDFREYCGLHRIKSFDDSALGVSQKALASI 529

Query 513 YRDIRDVDLFPGGLLEPSMPGGVVGETFGHILANQFADLKFGDTYFFLHQQAPQGFRAAQ 572

Y D++LF GG+LEP GG+VGETF ++ F LK+GD +F+ F +

Sbjct 530 YSHPDDIELFSGGILEPMAYGGLVGETFNCLMREVFYKLKYGDRFFYESYGEKGSFNNDE 589

Query 573 IKAILSVTMSSIICANSAVTQAQPDPFYMASQLNLPRPCSD--YSEMDVEPWLIHF 626

+ I VT++ ++C + + + Q + F++ N C + +DV ++ H+

Sbjct 590 LAEIRKVTLAHVLCQTTGIHEIQVNAFFLPGPKNPRVSCEKLLLNGLDVGAFVRHW 645

>[PVD36952.1](https://www.ncbi.nlm.nih.gov/protein/PVD36952.1?report=genbank&log$=protalign&blast_rank=7&RID=0) hypothetical protein C0Q70_03945 [Pomacea canaliculata]

Length=688

Score = 350 bits (899), Expect = 5e-108, Method: Compositional matrix adjust.

Identities = 212/596 (36%), Positives = 302/596 (51%), Gaps = 46/596 (8%)

Query 47 CSRLTYDQLRYRQIDGRCNHPRNYGSTGRPVKRYLRPHYQDKFGENLPRVYSVTGQLLPS 106

C Y+ + YR DG CNHP N G++ +P+ R L Y+D G +PR++ G LP+

Sbjct 123 CDPFQYESI-YRTADGTCNHPFNLGASTKPLSRLLPARYED--GREVPRIHGKLGLPLPN 179

Query 107 PRMVSWKLHPDQTAHDNNTMLVMQMGQFIDHDITRAP-ELSGRNASIKCCGVPPKERLP- 164

R+VS +HPD T+ VMQ GQ +DHD+ P + +IKCC LP

Sbjct 180 ARLVSKVIHPDIRTSTPFTVFVMQWGQLMDHDMVSTPLPVDDTRRTIKCCSDDKTHVLPN 239

Query 165 ---DCFPIDIPPGDPVFEDCMEFFRSSPAVDNDGNIIYPREQINALTSFIDGSAVYGSDL 221

+CFPI P + CMEF RS P D G + PRE +NA+TSFID S VYGS

Sbjct 240 ASSECFPIRFGPDESFLGSCMEFVRSMPIKDKHGTVKLPREHMNAVTSFIDASVVYGSTA 299

Query 222 DTYTWIRSENGTGVFLNTHLVHGRERLPSHPHLGPESCVSSNTAESYCQLAGDMRVNEQP 281

+ +R +GTG LN V + LPS G C+ T YC LAGD RVNE P

Sbjct 300 ERLKEVRDNHGTGYLLN---VTEDDYLPSS---GSADCIRP-TKSDYCFLAGDERVNEHP 352

Query 282 GLGSIHLLFHLHHNHIVRLLVAGILKKRGQPSSPERIAKFIQESSSALKEQIFQEVRKML 341

L IH + HN I +L K +P P+ E IFQ R ++

Sbjct 353 SLTLIHTILVRLHNRIAHIL------KYMRPFYPD--------------EDIFQRARAIV 392

Query 342 GAIIQKLTYCDWLPMILGPYLIDKFQLGCT---RRSRYNSDLDPRVANSFLSAALRFGHT 398

AI QK+ Y DWLP++LG + + L +RS+Y+S LDP + SF +A RFGHT

Sbjct 393 IAITQKIMYADWLPIVLGEQTMKTYGLAVGPKFKRSKYDSLLDPTIPTSFSTAVFRFGHT 452

Query 399 LIPNVYNFGDKRIHLKDTFNIPDASIRYYDNIIQCLI----KEGSEEAYDRYVSSAVSEH 454

LIP G + L++ F P + ++ + + D ++ V+ H

Sbjct 453 LIPRSVPIGGSQRLLRELFFNPSLIKNNIEKMVDSFTIGSDPDARSQMPDTFIVEEVTNH 512

Query 455 LFESTRGHKHALDLIAVNIQRGRDHGIPAYHYWRQYYRLRRIISLDE--FGEAGIAMKKA 512

LFES G + DLIA+NIQR RDHG+P Y+ +R+Y L RI S D+ G + A+

Sbjct 513 LFESEEGIGKSFDLIALNIQRARDHGLPTYNDFREYCGLHRIKSFDDSALGVSQKALASI 572

Query 513 YRDIRDVDLFPGGLLEPSMPGGVVGETFGHILANQFADLKFGDTYFFLHQQAPQGFRAAQ 572

Y D++LF GG+LEP GG+VGETF ++ F LK+GD +F+ F +

Sbjct 573 YSHPDDIELFSGGILEPMAYGGLVGETFNCLMREVFYKLKYGDRFFYESYGEKGSFNNDE 632

Query 573 IKAILSVTMSSIICANSAVTQAQPDPFYMASQLNLPRPCSD--YSEMDVEPWLIHF 626

+ I VT++ ++C + + + Q + F++ N C + +DV ++ H+

Sbjct 633 LAEIRKVTLAHVLCQTTGIHEIQVNAFFLPGPKNPRVSCEKLLLNGLDVGAFVRHW 688

>[XP_013088968.1](https://www.ncbi.nlm.nih.gov/protein/XP_013088968.1?report=genbank&log$=protalign&blast_rank=8&RID=0) PREDICTED: peroxidasin-like, partial [Biomphalaria glabrata]

Length=500

Score = 345 bits (884), Expect = 5e-108, Method: Compositional matrix adjust.

Identities = 200/520 (38%), Positives = 282/520 (54%), Gaps = 48/520 (9%)

Query 56 RYRQIDGRCNHPRNYGSTGRPVKRYLRPHYQDKFGENLPRVYSVTGQLLPSPRMVSWKLH 115

+YR +DG CNHP+N+G++ PV R L P Y D G PR LP+PR +S +H

Sbjct 9 KYRTVDGNCNHPKNWGTSLTPVARILPPAYDDNVGS--PRTLGKDNIPLPNPRTISSYVH 66

Query 116 PDQTAHDNNTMLVMQMGQFIDHDITRAPELSGRNASIKCCGVPPKERLP------DCFPI 169

P + + T+++MQ GQ++DHD+T P ++ N + KCCG P P +CF I

Sbjct 67 PSTSDPETRTIMIMQWGQWLDHDVTGFPVVTEANGATKCCG--PNGTKPIGAVNQNCFAI 124

Query 170 DIPPGDPVFE-DCMEFFRSSPAVDNDGNIIYPREQINALTSFIDGSAVYGSDLDTYTWIR 228

+P + F CMEF RS A D DG ++ PREQ+N+LTSFIDGS +YGS + +R

Sbjct 125 MLPCHEVNFAGSCMEFVRSVGATDKDGCLLNPREQVNSLTSFIDGSQIYGSTEELAAKLR 184

Query 229 SENGTGVFLNTHLVHGRERLPSHPHLGPESCVSSNTAESYCQLAGDMRVNEQPGLGSIHL 288

+GTG+ L T LP SC+ YC LAGD+RVNE P LG++H

Sbjct 185 --DGTGILLKT---KNDTFLPEDVS---ASCILRPGTNDYCFLAGDVRVNEHPALGAMHT 236

Query 289 LFHLHHNHIVRLLVAGILKKRGQPSSPERIAKFIQESSSALKEQIFQEVRKMLGAIIQKL 348

++ HN I + L + +P+ + E+IFQ RK++GA+ Q +

Sbjct 237 IWLRAHNSIAKEL------RTRRPADTD--------------EEIFQMTRKIIGALQQVI 276

Query 349 TYCDWLPMILGPYLIDKFQLGCTRRSRYNSDLDPRVANSFLSAALRFGHTLIPNVYNFGD 408

TY +WLP+ILG T R++ S +DPR+ N F +AA+RFGH+ IP V G

Sbjct 277 TYNEWLPIILGTEATKLKLTSKTGRTKRLSGVDPRILNEFSTAAMRFGHSFIPEVIPIGT 336

Query 409 KRIHLKDTFNIPDASIRYYDNIIQCLIKEGS-----EEAYDRYVSSAVSEHLFESTRGHK 463

+R+ L+ FN P + D++I + G+ E+ DR ++ HLFE K

Sbjct 337 RRVPLRTLFNRPAEVLDNLDDLIAGVAGVGTPGRNMAESLDRNFVEEITNHLFEPPAAPK 396

Query 464 -HALDLIAVNIQRGRDHGIPAYHYWRQYYRLRRIISLDE---FGEAGIAMKKAYRDIRDV 519

LDLI++N+QRGRDHGIP Y +R LR I+ D+ G + + YR + D+

Sbjct 397 GRGLDLISLNLQRGRDHGIPPYTAYRAVCGLRPIVDFDDTEALGPFASQLGRVYRSVDDI 456

Query 520 DLFPGGLLEPSMPGGVVGETFGHILANQFADLKFGDTYFF 559

DLF G + EP G +VG T IL QF +LKFGD +FF

Sbjct 457 DLFTGLVHEPPAKGALVGPTLSCILGTQFYNLKFGDRFFF 496

>[XP_025096385.1](https://www.ncbi.nlm.nih.gov/protein/XP_025096385.1?report=genbank&log$=protalign&blast_rank=9&RID=0) myeloperoxidase-like [Pomacea canaliculata]

Length=879

Score = 355 bits (912), Expect = 7e-108, Method: Compositional matrix adjust.

Identities = 213/581 (37%), Positives = 310/581 (53%), Gaps = 51/581 (9%)

Query 56 RYRQIDGRCN---HPRNYGSTGRPVKRYLRPHYQDKFGENLPRVYSVTGQ----LLPSPR 108

+ R DG CN HP GS +P KRYL P Y D G + GQ LPSPR

Sbjct 322 KTRTADGTCNNLLHPLK-GSAFQPFKRYLPPAYSDGVGAPRNQSKRTNGQRQPMALPSPR 380

Query 109 MVSWKLHPDQTAHDNNTMLVMQMGQFIDHDITRAPELSGRNASIKCCGVPPKERLPD--C 166

+VS +H + A + + L+MQ GQF+ HD+T P + + + CCG + RL C

Sbjct 381 LVSRTVHLVKDAPSDFSGLLMQFGQFLAHDLTGTPTVG--DGGLSCCG---EARLSSEAC 435

Query 167 FPIDIPPGDPVFED--CMEFFRSSPAVDNDGNIIYPREQINALTSFIDGSAVYGSDLDTY 224

FPIDIP GD F D CM F RS PA D+ + PREQ N TSF+DGS +YGS +

Sbjct 436 FPIDIPAGDTYFTDSTCMSFLRSLPATDHRNTPLRPREQRNEATSFLDGSVIYGSTDEQS 495

Query 225 TWIRSENGTGVFLNTHLVHGRERLPSHPHLGPESCVSSNTAESYCQLAGDMRVNEQPGLG 284

+R F L +P G S N + C +GD+RVNE PGL

Sbjct 496 QALRE------FRQGLLKSSSGDMPILQDDG-----SCNAEDKLCLKSGDIRVNEVPGLS 544

Query 285 SIHLLFHLHHNHIVRLLVAGILKKRGQPSSPERIAKFIQESSSALKEQIFQEVRKMLGAI 344

++H+LF HN + + LVA + + S +A+ E +FQ R +L A

Sbjct 545 ALHVLFQRQHNRLAKELVA-------------LLPSVLSSSPAAVDEWVFQTARSILAAQ 591

Query 345 IQKLTYCDWLPMILGPYLIDKFQLGCTRRSRYNSDLDPRVANSFLSAALRFGHTLIPNVY 404

+Q +TY WLP++LG I + +L + Y+ ++DP +AN F +AA RFGH+L+P+

Sbjct 592 MQHITYTLWLPLVLGRSTIQRQRLDDVLVT-YDDNMDPAIANVFATAAFRFGHSLVPDFL 650

Query 405 NFGDKRIHLKDTFNIPDASIRYYDNIIQCLIKEGSEEAYDRYVSSAVSEHLFESTRGHKH 464

+GD+ + L TF P + + +++ L+ ++ + D + S + LFES +

Sbjct 651 KYGDEHVPLHQTFANPKFVRKDLEKVLEGLLATRAQ-SLDAFFVSTLRNRLFESI--PRQ 707

Query 465 ALDLIAVNIQRGRDHGIPAYHYWRQYYRLRRIISLD--EFGEAGIAMKKAYRDIRDVDLF 522

LDL+A+NIQRGRDHG+P Y+ WRQ+ L + D GEA ++++ Y + D+DLF

Sbjct 708 GLDLVALNIQRGRDHGLPPYNVWRQFCGLPPLTGFDPVALGEASASLREVYMSVDDIDLF 767

Query 523 PGGLLEPSMPGGVVGETFGHILANQFADLKFGDTYFFLHQQAPQGFRAAQIKAILSVTMS 582

GG+ EP + G +VG TF I+ QF DLKFGD +F+ Q P F Q+ I T++

Sbjct 768 TGGIAEPPLEGALVGPTFACIIGRQFFDLKFGDRFFY---QTPGVFTPDQLHFIQGSTLA 824

Query 583 SIICANSAVTQAQPDPFYMASQLNLPRPCSDY-SEMDVEPW 622

+++CAN+A+ Q +PF S N PCS + + ++ W

Sbjct 825 AMMCANTAIKAVQRNPFVKESDENPRLPCSQLPTSLSLDAW 865

>[QIQ54711.1](https://www.ncbi.nlm.nih.gov/protein/QIQ54711.1?report=genbank&log$=protalign&blast_rank=10&RID=0) capsule gland specific secretory protein [Reishia bronni]

Length=652

Score = 348 bits (894), Expect = 1e-107, Method: Compositional matrix adjust.

Identities = 224/585 (38%), Positives = 304/585 (52%), Gaps = 54/585 (9%)

Query 20 PDIYARRGDLGDSTGSLDFVSEADLEHCSRL---TYDQLRYRQIDGRCNHPRNYGSTGRP 76

PD+ R +TG++ F ++ ++ T RYR DG CNH N G+ P

Sbjct 39 PDLVMSR-----TTGAIKFKPLPLIQEHRKICEETKSNRRYRTADGTCNHAFNLGAANTP 93

Query 77 VKRYLRPHYQDKFGENLPRVYSVTGQLLPSPRMVSWKLHPDQTAHDNNTMLVMQMGQFID 136

+ R L P Y D N PR V G LP+P +S +H Q D T+L+MQ GQFID

Sbjct 94 LNRMLPPEYDDL--NNSPRKKGVHGFELPAPTDISRLMHRPQNDFDGRTVLLMQWGQFID 151

Query 137 HDITRAP---ELSGRNASIKCCGVPPKERLPDCFPIDIPPGDPVFE-DCMEFFRSSPAVD 192

HD ++P + CC K+R C PI + GD F CMEF RS PA +

Sbjct 152 HDFAQSPVEVPEGLKEGDPYCCLPNVKDREGVCMPIYLHQGDTRFRHTCMEFMRSVPAKN 211

Query 193 NDGNIIYPREQINALTSFIDGSAVYGSDLDTYTWIRS-ENGTGVFLNTHLVHGRERLPSH 251

G I+PREQIN LTSFIDGS +YGS+L+ +R + FL + G

Sbjct 212 QHGYNIFPREQINVLTSFIDGSPIYGSNLEVQKKVRCLQQSKSAFLKMNSTTG------- 264

Query 252 PHLGPESCVSSNTAE--SYCQLAGDMRVNEQPGLGSIHLLFHLHHNHIVRLLVAGILKKR 309

L PES E YC LAGD RVNEQPGLG +H L + HN I A LK +

Sbjct 265 --LLPESDEGDCMHEPGQYCFLAGDRRVNEQPGLGVLHTLLNKLHNRI-----ASKLKDK 317

Query 310 GQPSSPERIAKFIQESSSALKEQIFQEVRKMLGAIIQKLTYCDWLPMILGPYLIDKFQLG 369

+ + PE +FQE RK++ AIIQ + Y +WLP IL ++ K+ L

Sbjct 318 LRNADPE---------------IVFQETRKVVIAIIQNIHYGEWLPQILSDSVMRKYDLH 362

Query 370 CTRRSRYNSDLDPRVANSFLSAALRFGHTLIPNVYNFGDKRIHLKDTFNIPDASIRYYDN 429

RR Y+ +DPR+ NSF +AA RFGH+LIP Y K + L+ TFN P +

Sbjct 363 TGRRVAYSGSVDPRILNSFSTAAFRFGHSLIPEHYVVNGKAVRLRKTFNNPALVFDNFKG 422

Query 430 IIQCLIKEGSE-EAYDRYVSSAVSEHLFESTRGHKHA----LDLIAVNIQRGRDHGIPAY 484

+++ L++ G+E + D ++ V+ HLFE ++A LDL+A NIQRGRDHG+P Y

Sbjct 423 MLKALMEPGAESQKIDSHIVEEVTRHLFEPEDQVENAPSRGLDLVAFNIQRGRDHGLPPY 482

Query 485 HYWRQYYRLRRIISLDEF---GEAGIAMKKAYRDIRDVDLFPGGLLEPSMPGGVVGETFG 541

+ +RQ+ L I F G + Y + D+DLF G LLE G VG TFG

Sbjct 483 NKYRQHCGLPPITDFYNFTANSSVGQQLSSLYNSVDDIDLFTGALLEDPEEFGKVGPTFG 542

Query 542 HILANQFADLKFGDTYFFLHQQAPQGFRAAQIKAILSVTMSSIIC 586

I+A QF LKFGD ++F + +GF Q+ ++ VTM+ ++C

Sbjct 543 CIMAVQFHALKFGDRFYFETCRHLEGFTDGQLASLRKVTMAHVMC 587

>[XP_005099074.2](https://www.ncbi.nlm.nih.gov/protein/XP_005099074.2?report=genbank&log$=protalign&blast_rank=11&RID=0) chorion peroxidase [Aplysia californica]

Length=637

Score = 348 bits (892), Expect = 2e-107, Method: Compositional matrix adjust.

Identities = 211/575 (37%), Positives = 300/575 (52%), Gaps = 44/575 (8%)

Query 56 RYRQIDGRCNHPRNYGSTGRPVKRYLRPHYQDKFGENLPRVYSVTGQLLPSPRMVSWKLH 115

+YR IDG CN+ RN G+ +R L P Y D PR +V+G+ LPS R VS H

Sbjct 75 KYRTIDGTCNNWRNQGAARTQARRLLPPAYDDV--NQKPRQTAVSGKPLPSARAVSIAAH 132

Query 116 PDQTAHDNNTMLVMQMGQFIDHDITRAPELSGRNASIKCCG----VPPKERLPDCFPIDI 171

P T ++MQ GQFIDHD+T P + +++IKCCG +P + +CFPI +

Sbjct 133 PPTPKELGLTNMIMQWGQFIDHDVTAFPVATELDSTIKCCGSNSSIPQLCKDENCFPILL 192

Query 172 PPGDPVFE-DCMEFFRSSPAVDNDGNIIYPREQINALTSFIDGSAVYGSDLDTYTWIRSE 230

P D F CMEF RS A D GNI+ PR+QIN++TSFIDGS +YGS + +R

Sbjct 193 PANDGDFRGTCMEFVRSVAARDPQGNILNPRQQINSVTSFIDGSQIYGSSEELLKKLRRP 252

Query 231 NGTGVFLNTHLVHGRERLPSHPHLGPESCVSSNTAESYCQLAGDMRVNEQPGLGSIHLLF 290

N + L T LP E C+ + YC LAGD RVNE P L S+H L+

Sbjct 253 NSSR--LKTKKTKLGLFLPEATDEA-EGCILRENSNDYCFLAGDSRVNEHPALASMHTLW 309

Query 291 HLHHNHIVRLLVAGILKKRGQPSSPERIAKFIQESSSALKEQIFQEVRKMLGAIIQKLTY 350

HN I R L K R Q S+ E+IFQ RK++GA++QK+TY

Sbjct 310 VREHNRIARKLA----KLRPQDST----------------EEIFQLTRKIVGALLQKITY 349

Query 351 CDWLPMILGPYLIDKFQLGCTRRSRYNSDLDPRVANSFLSAALRFGHTLIPNVYNFGDKR 410

DWLP+I+G + T RS N LDP + NSF +A RFGH+++P G++

Sbjct 350 NDWLPIIIGRVATLAELVSKTGRSTPNLALDPSITNSFSTATFRFGHSMVPAEMLIGERT 409

Query 411 IHLKDTFNIPDASIRYYDNIIQCLI-----------KEGSEEAYDRYVSSAVSEHLFEST 459

++L+D FN P + D+++ L + E D+ V+ +++ LF+

Sbjct 410 VYLRDLFNRPAEVLDNLDDVLAGLAGVSRIHGLVGYRSRLVEDVDKEVAEDLTKFLFQPP 469

Query 460 RGHK-HALDLIAVNIQRGRDHGIPAYHYWRQYYRLRRIISLDE--FGEAGIAMKKAYRDI 516

+ DL+++NIQRGRDHGIP Y +R+ + ++ G G + Y +

Sbjct 470 NAPRGSGFDLVSLNIQRGRDHGIPPYTKFREICNYPPLTGFNDKALGPHGSKLATIYESV 529

Query 517 RDVDLFPGGLLEPSMPGGVVGETFGHILANQFADLKFGDTYFFLHQQAPQGFRAAQIKAI 576

D+DLF G L EP + GG+VG T ++ +QF LK GD +FF + GF Q++ I

Sbjct 530 DDIDLFTGLLYEPHIQGGIVGATLNCLIVHQFTYLKTGDRFFFDTSEKECGFTDDQLENI 589

Query 577 LSVTMSSIICANSAVTQAQPDPFYMASQLNLPRPC 611

T++SI+C N + Q + F + S+ NL C

Sbjct 590 RETTLASIMCENLEMPQLPVNVFRLVSKYNLLTDC 624

>[XP_035828376.1](https://www.ncbi.nlm.nih.gov/protein/XP_035828376.1?report=genbank&log$=protalign&blast_rank=12&RID=0) chorion peroxidase [Aplysia californica]

Length=651

Score = 338 bits (867), Expect = 1e-103, Method: Compositional matrix adjust.

Identities = 221/610 (36%), Positives = 317/610 (52%), Gaps = 77/610 (13%)

Query 49 RLTYD-QLRYRQIDGRCN---HPRNYGSTGRPVKRYLRPHYQDKFGENLPRVYSVTGQLL 104

+LT D + +YR DG CN HP +G+ G P KR L P Y D G+N PR Y V+G+ L

Sbjct 79 KLTCDPRAKYRTPDGYCNNLKHP-AWGTPGWPFKRILAPAYDD--GQNAPRAYGVSGRPL 135

Query 105 PSPRMVSWKLH---PDQTAHDNNTMLVMQMGQFIDHDITRAPELSGRNASIKCCGV---- 157

PSPR+VS +H D D T + GQF+DHD+ P L+ ++ + CC

Sbjct 136 PSPRVVSRIVHTADSDNVLTDRLTSMFTHFGQFLDHDLDLTP-LTNSDSELDCCEEMIQF 194

Query 158 --------PPKERLP----------DCFPIDIPPGDPVFED-CMEFFRSSPAVDNDGNII 198

PP L CFPI IP D F CM F RS +

Sbjct 195 PVRGFYTHPPTSSLAYLLKSRPLPGTCFPIPIPQPDGHFNSSCMNFVRSKVFAPSFKCKS 254

Query 199 YPREQINALTSFIDGSAVYGSDLDTYTWIRSENGTGVFLNTHLVHGRERLPSHPHLGPES 258

REQ N +TSFID S VYGS +R G + L + + LPS+ +

Sbjct 255 STREQGNGITSFIDASQVYGSSKAKQDSLRERVGGRL-----LSYKSDLLPSN---STTT 306

Query 259 CVSSNTAESYCQLAGDMRVNEQPGLGSIHLLFHLHHNHIVRLLVA-GILKKRGQPSSPER 317

C S++ C AGD RVNEQP L ++H++FH +HNH+ L + IL

Sbjct 307 CRSTSIPGKVCFGAGDTRVNEQPALSALHIVFHRYHNHLANFLSSRNILWD--------- 357

Query 318 IAKFIQESSSALKEQIFQEVRKMLGAIIQKLTYCDWLPMILGPYLIDKFQLGCTRRSRYN 377

E IFQEVRK++GAI+Q + Y ++LP++LGP+ + ++L T R RY

Sbjct 358 ------------DETIFQEVRKIMGAILQHIVYKEYLPLLLGPHYMTAYELTPTLRHRYE 405

Query 378 SDLDPRVANSFLSAALRFGHTLIPNVYNFGDKRIHLKDTFNIP----DASIRYYDNIIQC 433

DPR+ NSF +AA RFGH+++P FG K+ L+ F P D + + D++I+

Sbjct 406 PSTDPRIMNSFATAAFRFGHSMVPGNLIFGVKKQPLESAFFCPHLIQDDARKGLDDLIRG 465

Query 434 LIKEGSEEAYDRYVSSAVSEHLFESTRGHKHALDLIAVNIQRGRDHGIPAYHYWRQYYRL 493

+ + S+ + DRY S +S +LF R +LDL+++NIQRGRDHG+P ++ +R+ L

Sbjct 466 TLSDMSQGS-DRYFSHTLSRNLF---RNGGRSLDLVSLNIQRGRDHGLPPHNDFRKMCGL 521

Query 494 RRIISLDEFGEAGIAMKKAYRDIRDVDLFPGGLLEPSMPGGVVGETFGHILANQFADLKF 553

R+ S E + + Y D+ DVDLF G L E + GGVVG TF ++A QF DLKF

Sbjct 522 SRLSSSPE-----APLLEVYEDMNDVDLFSGALTEEHVEGGVVGPTFACLIARQFRDLKF 576

Query 554 GDTYFFLHQQAPQGFRAAQIKAILSVTMSSIICANSAVTQAQPDPFYMASQLNLPRPCSD 613

GD +++ + GF AQ +AI V+++S++C + + PF S N C+D

Sbjct 577 GDRFWYENPDPHTGFSRAQFRAIKKVSLASLLCTVTNLETVPKSPFETVSFRNRELSCTD 636

Query 614 YSEMDVEPWL 623

+D+ W+

Sbjct 637 VPSLDLGHWM 646

>[XP_013079813.1](https://www.ncbi.nlm.nih.gov/protein/XP_013079813.1?report=genbank&log$=protalign&blast_rank=13&RID=0) PREDICTED: myeloperoxidase-like, partial [Biomphalaria glabrata]

Length=316

Score = 321 bits (822), Expect = 3e-101, Method: Compositional matrix adjust.

Identities = 153/303 (50%), Positives = 209/303 (69%), Gaps = 3/303 (1%)

Query 90 GENLPRVYSVTG-QLLPSPRMVSWKLHPDQTAHDNNTMLVMQMGQFIDHDITRAPELSGR 148

G N PR+YSV G + LPSPR++S KL PD + +M MQ GQF+ HDI +P +G

Sbjct 15 GMNTPRIYSVVGKEYLPSPRLISLKLFPDVMKESDLSMFTMQFGQFVSHDIGVSPVPTGS 74

Query 149 NASIKCCGVPPKERLPDCFPIDIPPGDPVFEDCMEFFRSSPAVDNDGNIIYPREQINALT 208

N +IKCCGV K+ +CFPI IP D FE CMEF RS A D GN +YPREQINALT

Sbjct 75 NGTIKCCGVSAKQMSKECFPIPIPKEDSRFEYCMEFVRSEAAKDEYGNQMYPREQINALT 134

Query 209 SFIDGSAVYGSDLDTYTWIRSENGTGVFLNTHLVHGRERLPSHPHLGPESCVSSNTAESY 268

SFIDGS +YGSD+ T +R+ENG G+ L+T +V+G+ERLP+ +CV + + SY

Sbjct 135 SFIDGSNIYGSDIITTQRLRTENGKGILLSTTIVNGKERLPNDTS-SKAACVRTTSPTSY 193

Query 269 CQLAGDMRVNEQPGLGSIHLLFHLHHNHIVRLLVAGIL-KKRGQPSSPERIAKFIQESSS 327

CQL+GD RVN+QP LG+ HL FHL+HN + R +V GIL KK G +PE++ K+I +

Sbjct 194 CQLSGDKRVNQQPVLGTQHLTFHLYHNELARRIVEGILVKKTGVKPTPEQVEKYIATAPD 253

Query 328 ALKEQIFQEVRKMLGAIIQKLTYCDWLPMILGPYLIDKFQLGCTRRSRYNSDLDPRVANS 387

+KE +FQEV+++ AI Q++ CD+LP ++G ++ ++L CT+RSRYN +DPR+AN

Sbjct 254 NVKEILFQEVKRINNAIFQRIAVCDYLPFVIGRSYMEMYKLTCTKRSRYNPYVDPRLANG 313

Query 388 FLS 390

F++

Sbjct 314 FIA 316

>[XP_034310730.1](https://www.ncbi.nlm.nih.gov/protein/XP_034310730.1?report=genbank&log$=protalign&blast_rank=14&RID=0) eosinophil peroxidase-like [Crassostrea gigas]

Length=691

Score = 333 bits (854), Expect = 3e-101, Method: Compositional matrix adjust.

Identities = 222/596 (37%), Positives = 317/596 (53%), Gaps = 70/596 (12%)

Query 57 YRQIDGRCN---HPRNYGSTGRPVKRYLRPHYQDKFGENLPRVYSVTGQLLPSPRMVSWK 113

YR IDGRCN HP ++G+ P RYL Y D G + PR + G LPSPR++S

Sbjct 135 YRSIDGRCNNLIHP-SWGAAITPQPRYLPAEYDD--GISTPRNRAKNGSPLPSPRLISNN 191

Query 114 LH---PDQTAHDN-NTMLVMQMGQFIDHDITRAPELSGR-NASIKCCGVPPKERLPDCFP 168

L D T D+ T++VM GQFIDHD+T P + G I CCG + R P CFP

Sbjct 192 LFRAPGDCTETDHARTLMVMAWGQFIDHDLTHTPTMKGDGEVPITCCGENVQNR-PQCFP 250

Query 169 IDIPPGDPVFED-CMEFFRSSPAVDNDGNIIYPREQINALTSFIDGSAVYGSDLDTYTWI 227

I IP DP F+D CMEF RS+P+ DG + P+EQIN +TSFIDG +VYGS + +

Sbjct 251 ISIPSDDPNFDDSCMEFVRSAPSPPGDGCQLGPQEQINQITSFIDGGSVYGSSKEKMEEL 310

Query 228 RSENGTGVFLNTHLVHGRERLPSHPHLGP---ESCVSSNTAESYCQLAGDMRVNEQPGLG 284

++ + G+ R L P ++C SS + +CQ AGD+RVNE P LG

Sbjct 311 KNTD-----------TGQMRTSPGDLLPPAVNDTCESSAETD-FCQNAGDLRVNEIPSLG 358

Query 285 SIHLLFHLHHNHIVRLLVAGILKKRGQPSSPERIAKFIQESSSALKEQIFQEVRKMLGAI 344

HLLF HN IV G L+K +Q S+LK ++QE RK++GA+

Sbjct 359 GNHLLFVREHNRIV-----GELRK-------------VQPKWSSLK--LYQEARKIIGAL 398

Query 345 IQKLTYCDWLPMILGPYLIDKFQLGCTR---RSRYNSDLDPRVANSFLSAALRFGHTLIP 401

+Q++TY ++LP IL ++ +L + Y+S +P N+F +A RFGH+LIP

Sbjct 399 LQQVTYGEFLPSILSKQDLENHKLKLRNSGFSNNYDSSRNPATKNAFNAAVFRFGHSLIP 458

Query 402 N-----VYNFGDK--RIHLKDTFNIPDASI----RYYDNIIQCLIKEGSEEAYDRYVSSA 450

+Y+F + ++ F P I R ++ + ++ S + D + A

Sbjct 459 PNLAYLLYDFMSRVNSTTIESIFFNPHLLITEGGRRVSDLARFIVTSNSMKV-DNQLEGA 517

Query 451 VSEHLFESTRGHKHALDLIAVNIQRGRDHGIPAYHYWRQYYRLRRIISL----DEFGEAG 506

V +HLFE+ H +DL A+N+QRGRDHG+P Y+ WR++ L S D E

Sbjct 518 VRDHLFEN--AHGKGMDLGALNLQRGRDHGLPPYNAWRKWCGLTVATSFSNLPDITDEKK 575

Query 507 IAMKKAYRDIRDVDLFPGGLLEPSMPGGVVGETFGHILANQFADLKFGDTYFFLHQQAPQ 566

+ Y + D+DLF GG+ E + G VG F I+ NQF DLK GD Y++ + +

Sbjct 576 AVLAALYSGVDDIDLFAGGVAETPLDGAAVGPLFSCIIGNQFRDLKDGDRYWY-ENRGVE 634

Query 567 GFRAAQIKAILSVTMSSIICANSAVTQAQPDPFYMASQLNLPRPCSDYSEMDVEPW 622

GF+ AQ++ I V ++ I+C N V QPD F++ S N + CS + E+D W

Sbjct 635 GFKQAQLREIRKVKLAKIVCTNLGVDPIQPDVFHVPSPSNNWQSCSQFPEIDFARW 690

>[ODM94915.1](https://www.ncbi.nlm.nih.gov/protein/ODM94915.1?report=genbank&log$=protalign&blast_rank=15&RID=0) Chorion peroxidase [Orchesella cincta]

Length=841

Score = 336 bits (862), Expect = 7e-101, Method: Compositional matrix adjust.

Identities = 226/585 (39%), Positives = 322/585 (55%), Gaps = 62/585 (11%)

Query 57 YRQIDGRCNHPRN--YGSTGRPVKRYLRPHYQDKFGENLPRVYSVTGQLLPSPRMVSWKL 114

YR IDG CN+ N +G + +R L+P Y D G N PRV + TGQ LPSPR+VS +

Sbjct 248 YRTIDGSCNNQENVDWGKSQTAFQRILQPKYGD--GLNSPRV-TATGQPLPSPRLVSDRF 304

Query 115 HPDQT-AHDNNTMLVMQMGQFIDHDITRAPELSGRNAS-IKCC--GVPPKERL--PDCFP 168

PD+ + N T++VMQ GQF+DHD+T P + G N++ I CC G P + L PDCFP

Sbjct 305 IPDENRPYRNLTLMVMQWGQFVDHDLTHTPIVKGENSTGISCCENGQPLEASLRHPDCFP 364

Query 169 IDIPPGDPVFED----CMEFFRSSPAVDNDGNIIYPREQINALTSFIDGSAVYGSDLDTY 224

I+I P D ++ CMEF RS PA + N+ PREQ+ +T +DGS +YGS

Sbjct 365 IEITPEDSFYKQFNQRCMEFVRSLPAPRKECNL-GPREQMTQVTHLLDGSMIYGSTERRA 423

Query 225 TWIRSENGTGVFLNTHLVHGRERLPSHPHLGPESCVSSNTAESYCQLAGDMRVNEQPGLG 284

T +R G + + T+ GRE +P++P+ C +N E +C AGD+RVNEQ L

Sbjct 424 TSLREGVGGRLLVQTN--GGREHMPANPN----ECSDAN-QEKFCFRAGDLRVNEQVHLA 476

Query 285 SIHLLFHLHHNHIVRLLVAGILKKRGQPSSPERIAKFIQESSSALKEQIFQEVRKMLGAI 344

IH ++ HN + R L IL + E ++QE R+++ A

Sbjct 477 LIHTIWFREHNRVARELE--IL------------------NPHWADETLYQETRRIVIAE 516

Query 345 IQKLTYCDWLPMILGPYLIDKFQLGCTRR---SRYNSDLDPRVANSFLSAALRFGHTLIP 401

+Q +TY +WLP+ILGP + F+L T++ YN +DP V N+F SAA RFGH++I

Sbjct 517 LQHITYNEWLPLILGPNYMRTFELQPTQKGYTQLYNPSIDPSVTNAFASAAFRFGHSMIQ 576

Query 402 NVYN----FGDKRIH-LKDTFNIPDASIRYYDNIIQCLIKEGSEEAYDRYVSSAVSEHLF 456

V FG R T P D++++ L + S+ DR+ S AV+ HLF

Sbjct 577 GVVKMFNPFGASRNKPFSQTQFQPFEVYDSCDDLVRGLANQESQR-MDRFASQAVTNHLF 635

Query 457 ESTRGHKHALDLIAVNIQRGRDHGIPAYHYWRQYYRLRRIISLDEF-GEAGIAMKKA--- 512

E G LDL A+NIQRGRDH +P Y+ WR+ L+R+ S +F A AM +

Sbjct 636 EGQSGF--GLDLFALNIQRGRDHALPPYNDWREVCGLKRLNSWRDFEAVADKAMADSLST 693

Query 513 -YRDIRDVDLFPGGLLEPSMPGGVVGETFGHILANQFADLKFGDTYFFLHQQAPQGFRAA 571

Y ++ +VDLF GG+ E + G V+G TF I+ +QFA L+ GD +F+ ++ F A

Sbjct 694 LYPNVDEVDLFIGGVSEKPLDGAVLGPTFTCIVGDQFARLRRGDRFFY--EEVTARFTEA 751

Query 572 QIKAILSVTMSSIICANS-AVTQAQPDPFYMASQLNLPRPCSDYS 615

Q+ I V+++ ++C NS +T QP F AS LN PC+ S

Sbjct 752 QLDQIKKVSLARLLCDNSDDITVIQPLIFQKASFLNQRMPCNSES 796

>[XP_011426440.2](https://www.ncbi.nlm.nih.gov/protein/XP_011426440.2?report=genbank&log$=protalign&blast_rank=16&RID=0) eosinophil peroxidase [Crassostrea gigas]

[XP_034310073.1](https://www.ncbi.nlm.nih.gov/protein/XP_034310073.1?report=genbank&log$=protalign&blast_rank=16&RID=0) eosinophil peroxidase [Crassostrea gigas]

Length=691

Score = 331 bits (849), Expect = 2e-100, Method: Compositional matrix adjust.

Identities = 222/596 (37%), Positives = 316/596 (53%), Gaps = 70/596 (12%)

Query 57 YRQIDGRCN---HPRNYGSTGRPVKRYLRPHYQDKFGENLPRVYSVTGQLLPSPRMVSWK 113

YR IDGRCN HP ++G+ P RYL Y D G + PR + G LPSPR +S

Sbjct 135 YRSIDGRCNNLVHP-SWGAAITPQPRYLPAEYDD--GISTPRNRAKNGSPLPSPRRISNN 191

Query 114 LH---PDQTAHDN-NTMLVMQMGQFIDHDITRAPELSGR-NASIKCCGVPPKERLPDCFP 168

L D T D+ T++VM GQFIDHD+T P + G I CCG + R P CFP

Sbjct 192 LFRAPGDCTETDHARTLMVMAWGQFIDHDLTHTPTMKGDGEVPITCCGENVQNR-PQCFP 250

Query 169 IDIPPGDPVFED-CMEFFRSSPAVDNDGNIIYPREQINALTSFIDGSAVYGSDLDTYTWI 227

I IP DP F+D CMEF RS+P+ DG + P+EQIN +TSFIDG +VYGS + +

Sbjct 251 ISIPSDDPHFDDSCMEFVRSAPSPPGDGCQLGPQEQINQITSFIDGGSVYGSSKEKMEEL 310

Query 228 RSENGTGVFLNTHLVHGRERLPSHPHLGP---ESCVSSNTAESYCQLAGDMRVNEQPGLG 284

++ + G+ R L P ++C SS + +CQ AGD+RVNE P LG

Sbjct 311 KNTD-----------TGQMRTSPGDLLPPAVDDTCESSAETD-FCQNAGDLRVNEIPSLG 358

Query 285 SIHLLFHLHHNHIVRLLVAGILKKRGQPSSPERIAKFIQESSSALKEQIFQEVRKMLGAI 344

HLLF HN IV G L+K +Q S+LK ++QE RK++GA+

Sbjct 359 GNHLLFVREHNRIV-----GELRK-------------VQPKWSSLK--LYQEARKIIGAL 398

Query 345 IQKLTYCDWLPMILGPYLIDKFQLGCTR---RSRYNSDLDPRVANSFLSAALRFGHTLIP 401

+Q++TY ++LP IL ++ +L + Y+S +P N+F +A RFGH+LIP

Sbjct 399 LQQVTYGEFLPSILSKQDLENHKLKLRNSGFSNNYDSSRNPATKNAFNAAVFRFGHSLIP 458

Query 402 N-----VYNFGDK--RIHLKDTFNIPDASI----RYYDNIIQCLIKEGSEEAYDRYVSSA 450

+Y+F + ++ F P I R ++ + ++ S + D + A

Sbjct 459 PNLAYLLYDFMSRVNSTTIESIFFNPHLLITEGGRRVSDLARFIVTSNSMKV-DNQLEGA 517

Query 451 VSEHLFESTRGHKHALDLIAVNIQRGRDHGIPAYHYWRQYYRLRRIISL----DEFGEAG 506

V +HLFE+ H +DL A+N+QRGRDHG+P Y+ WR++ L S D E

Sbjct 518 VRDHLFEN--AHGKGMDLGALNLQRGRDHGLPPYNAWRKWCGLTVATSFSNLPDITDEKK 575

Query 507 IAMKKAYRDIRDVDLFPGGLLEPSMPGGVVGETFGHILANQFADLKFGDTYFFLHQQAPQ 566

+ Y + D+DLF GG+ E + G VG F I+ NQF DLK GD Y++ + +

Sbjct 576 TVLADLYSGVDDIDLFAGGVAETPLDGAAVGPLFSCIIGNQFRDLKDGDRYWY-ENRGVE 634

Query 567 GFRAAQIKAILSVTMSSIICANSAVTQAQPDPFYMASQLNLPRPCSDYSEMDVEPW 622

GF+ AQ++ I V ++ I+C N V QPD F++ S N + CS + E+D W

Sbjct 635 GFKQAQLREIRKVKLAKIVCTNLGVDPIQPDVFHVPSPSNNWQSCSQFPEIDFARW 690

>[XP_025107410.1](https://www.ncbi.nlm.nih.gov/protein/XP_025107410.1?report=genbank&log$=protalign&blast_rank=17&RID=0) chorion peroxidase-like [Pomacea canaliculata]

Length=628

Score = 329 bits (843), Expect = 2e-100, Method: Compositional matrix adjust.

Identities = 207/574 (36%), Positives = 298/574 (52%), Gaps = 47/574 (8%)

Query 56 RYRQIDGRCNHPRNYGSTGRPVKRYLRPHYQDKFGENLPRVYSVTGQLLPSPRMVSWKLH 115

RYR DG NH N G P R L P Y++ G + PR V G+ LP+ VS +H

Sbjct 74 RYRTADGSGNHVFNLGMANTPPSRILPPDYEN--GVDSPRTLGVRGRPLPAALDVSRFIH 131

Query 116 PDQTAHDNNTMLVMQMGQFIDHDITRAPELSGRNASIKCC--GVPPKERLPDCFPIDIPP 173

D N T+++M GQF+DHD+ S +N SI CC G PDCFP+

Sbjct 132 TDVVDFQNFTVMLMAWGQFMDHDLAAFILSSRQNGSIACCVNGTVNPVPNPDCFPMIKSD 191

Query 174 GDPVFE-DCMEFFRSSPAVDNDGNIIYPREQINALTSFIDGSAVYGSDLDTYTWIRSENG 232

DP FE C+EF RS A PR+QIN TSFID S VYGS + +R +

Sbjct 192 NDPRFESSCLEFSRSLAARTKMAP--GPRQQINTATSFIDASMVYGSSQEVQLNLREKLS 249

Query 233 TGVFLNTHLVHGR--ERLPSHPHLGPESCVSSNTAESYCQLAGDMRVNEQPGLGSIHLLF 290

+G +HL+ E LP P ++C+ S +C LAGD RV+EQPGL +H LF

Sbjct 250 SGE--PSHLLRSESGELLPRRPQ---DNCIHS--PGRFCFLAGDDRVSEQPGLTVVHTLF 302

Query 291 HLHHNHIVRLLVAGILKKRGQPSSPERIAKFIQESSSALKEQIFQEVRKMLGAIIQKLTY 350

HN I R L ++ S E+IFQ RK++GAI+Q + Y

Sbjct 303 LQLHNKIARDLAT------------------LRPYDS--HEEIFQLTRKIVGAIVQNIQY 342

Query 351 CDWLPMILGPYLIDKFQLGCTRRSRYNSDLDPRVANSFLSAALRFGHTLIPNVYNFGDKR 410

+WLP+ L ++ +F L R+RY +DP + N+F +A R GHTLIP +N +

Sbjct 343 SEWLPIFLPKSILSQFSLLTGSRTRYLPTVDPSIYNAFATAVFRMGHTLIPAFFNVSGRV 402

Query 411 IHLKDTFNIPDASIRYYDNIIQCLIK---EGSEEAYDRYVSSAVSEHLFESTR-----GH 462

I L+ FN PD + ++ L+ A+DR+ + ++ HLFE

Sbjct 403 IPLRQLFNKPDIIFEDFKGVLLSLVSPLFTNGARAFDRFFTEEITGHLFEPRTQPPAPAR 462

Query 463 KHALDLIAVNIQRGRDHGIPAYHYWRQYYRLRRIISLDEFGEAGIAMKKA--YRDIRDVD 520

LDLIA+N+QRGRDHG+P+Y+ +R+ L+++ S +F + + +K A Y ++ D+D

Sbjct 463 SRGLDLIALNLQRGRDHGLPSYNNYREICGLKKLTSFSQFVPSAVGLKLATVYDNVDDID 522

Query 521 LFPGGLLEPSMPGGVVGETFGHILANQFADLKFGDTYFFLHQQAPQGFRAAQIKAILSVT 580

LF G + E + GG+VG T G +L QF LKFGD ++F +GF Q++AI VT

Sbjct 523 LFTGLMSEKPLAGGLVGPTLGCLLGIQFHSLKFGDRFYFETVSELEGFTNDQLRAIRYVT 582

Query 581 MSSIICANSAVTQAQPDPFYMASQLNLPR-PCSD 613

++ +IC + V Q F + +++ P CS+

Sbjct 583 LAHVICMSRRVNSVQLKAFEVFNEITNPEVDCSE 616

>[XP_035828707.1](https://www.ncbi.nlm.nih.gov/protein/XP_035828707.1?report=genbank&log$=protalign&blast_rank=18&RID=0) peroxidase-like protein 3 isoform X2 [Aplysia californica]

Length=491

Score = 319 bits (818), Expect = 2e-98, Method: Compositional matrix adjust.

Identities = 195/511 (38%), Positives = 272/511 (53%), Gaps = 59/511 (12%)

Query 127 LVMQMGQFIDHDITRAPELSGRNASIKCCG----VPPKERLPDCFPIDIPPGDPVFE-DC 181

+VMQ GQFIDHDIT P + +++IKCCG +P + +CFPI +P D F C

Sbjct 1 MVMQWGQFIDHDITAFPVATELDSTIKCCGSNSSIPQLCKDENCFPILLPANDGDFRGTC 60

Query 182 MEFFRSSPAVDNDGNIIYPREQINALTSFIDGSAVYGSDLDTYTWIRSENGTGVFLNTHL 241

MEF RS A D GNI+ PR+QIN++TSFIDGS +YGS + +R N L T L

Sbjct 61 MEFVRSVAARDPQGNILNPRQQINSVTSFIDGSQIYGSSEELLKKLREPNS--FLLKTKL 118

Query 242 VHGRERLPSHPHLGPESCVSSNTAESYCQLAGDMRVNEQPGLGSIHLLFHLHHNHIVRLL 301

+ LP E C+ + YC LAGD RVNE P L S+H L+ HN I R L

Sbjct 119 ---GKFLPEATD-EAEGCILRENSNDYCFLAGDSRVNEHPALASMHTLWMREHNRIAREL 174

Query 302 VAGILKKRGQPSSPERIAKFIQESSSALKEQIFQEVRKMLGAIIQKLTYCDWLPMILGPY 361

K R Q S+ E+IFQ RK++GA++QK+TY DWLP+ILG

Sbjct 175 A----KLRPQDST----------------EEIFQLTRKIVGALLQKITYNDWLPIILGTV 214

Query 362 LIDKFQLGCTRRSRYNSDLDPRVANSFLSAALRFGHTLIPNVYNFGDKRIHLKDTFNIPD 421

+ + RS N +DPR++NSF +A RFGH+L+P G++ +HL+D FN P

Sbjct 215 ATQGKLVSKSGRSTPNLAVDPRISNSFSTATFRFGHSLVPAEILIGERNVHLRDLFNRPA 274

Query 422 ASIRYYDNII----------------QCLIKEGSEEAYDRYVSSAVSEHLFE---STRGH 462

+ D+++ CL+ E DR V+ +++ LFE S RG

Sbjct 275 EVLDNLDDVLAGLAAVSRIHGPVACTSCLL-----EDVDREVAEDLTKFLFEPPNSPRG- 328

Query 463 KHALDLIAVNIQRGRDHGIPAYHYWRQYYRLRRIISLDE--FGEAGIAMKKAYRDIRDVD 520

DL+++NIQRGRDHGIP Y +R++ L + ++ G+ G + Y + D+D

Sbjct 329 -SGFDLVSLNIQRGRDHGIPPYTTFREFCNLPALTGFNDRALGQHGSRLATVYESVDDID 387

Query 521 LFPGGLLEPSMPGGVVGETFGHILANQFADLKFGDTYFFLHQQAPQGFRAAQIKAILSVT 580

LF G L EP + GG++GET ++ NQF LK D +FF + GF Q++ I T

Sbjct 388 LFTGLLYEPHVYGGIIGETLMCLIGNQFIHLKSADRFFFDTSELEFGFTDDQLENIRKTT 447

Query 581 MSSIICANSAVTQAQPDPFYMASQLNLPRPC 611

++SI+C N + Q D F + S N C

Sbjct 448 LASIMCENLEMPQLVVDVFRLVSNNNKLTDC 478

>[OWF41499.1](https://www.ncbi.nlm.nih.gov/protein/OWF41499.1?report=genbank&log$=protalign&blast_rank=19&RID=0) Peroxidase-like protein [Mizuhopecten yessoensis]

Length=701

Score = 325 bits (833), Expect = 4e-98, Method: Compositional matrix adjust.

Identities = 209/597 (35%), Positives = 308/597 (52%), Gaps = 68/597 (11%)

Query 56 RYRQIDGRCNHPRN--YGSTGRPVKRYLRPHYQDKFGENLPRVYSVTGQLLPSPRMVSWK 113

RYR DG CN+ N GS+ RP +R L P Y+D G + PR+ + G +LPS R+VS

Sbjct 140 RYRSADGTCNNIHNPLSGSSFRPHRRLLPPDYED--GIDSPRIRADNGAILPSARLVSNT 197

Query 114 LHPDQTAHDNNTMLVMQM--GQFIDHDITRAPELSGRNAS-IKCCGVPPKERLPDCFPID 170

LH H + + VM M GQF+DHD+T L N + I CC CFPI

Sbjct 198 LHNAGRTHTSESFTVMYMSFGQFLDHDLTSTASLKDENNNPIDCCSNSNAALSSACFPIK 257

Query 171 IPPGDPVF-EDCMEFFRSSPAVDNDGNIIYPREQINALTSFIDGSAVYGSDLDTYTWIRS 229

IP D F + CM F RS+ AV N + Y R+QIN LTS++D S VYGS L++ +R

Sbjct 258 IPINDTRFTKSCMSFTRSAAAVKNGCDPDY-RQQINQLTSYLDASNVYGSTLESQERLRE 316

Query 230 ENGTGVFLNTHLVHGRERLPSHPHLGP----ESCVSSNTAESYCQLAGDMRVNEQPGLGS 285

++G GR R+ L P +C+ +N ++++C AGD R +E P L +

Sbjct 317 KSG-----------GRMRVSDQGDLLPSYKNNTCILTNPSKTHCFDAGDERNSEVPTLTT 365

Query 286 IHLLFHLHHNHIVRLLVAGILKKRGQPSSPERIAKFIQESSSALKEQIFQEVRKMLGAII 345

+H+ F HN I L + I + E +FQEVRK++GA++

Sbjct 366 LHIAFLREHNRIANTL------------------RLINRDWN--DELVFQEVRKIVGALL 405

Query 346 QKLTYCDWLPMILGPYLIDKFQLGCTR---RSRYNSDLDPRVANSFLSAALRFGHTLIPN 402

Q + Y ++LP ++GP +D + L T RS YN +D V N F +AA RFGH+ IP+

Sbjct 406 QHIAYNEYLPKVIGPSFMDMYDLKPTPTGFRSVYNDTIDATVTNVFATAAFRFGHSQIPD 465

Query 403 VYNFGDKRIHLKDTFNI------PDASIRYYDNIIQCLIK---EGSEEAYDRYVSSAVSE 453

+KR K N+ PD + + N + L + ++ D + S+V

Sbjct 466 KMMLINKRFKKKAIMNLEKQYFKPD--LMFEKNGPEWLARWQVSYAQTKEDNSIQSSVRN 523

Query 454 HLFESTRGHKHALDLIAVNIQRGRDHGIPAYHYWRQYYRL--------RRIISLDEFGEA 505

LF ++ +LDL A+N+QRGRDHG+P+Y+ WR + L R+ ++ +

Sbjct 524 FLFLDSKND--SLDLAALNMQRGRDHGLPSYNAWRSWCGLNPASDFSHRKSGLVNHYRTG 581

Query 506 GIAMKKAYRDIRDVDLFPGGLLEPSMPGGVVGETFGHILANQFADLKFGDTYFFLHQQAP 565

+ + Y D+DL+ GGLLE +PG +VG TF I++ QF DL+ GD +++ +

Sbjct 582 RNRLSRIYSSPNDIDLYSGGLLEKHLPGALVGPTFACIISRQFRDLQKGDRFWYENDAPY 641

Query 566 QGFRAAQIKAILSVTMSSIICANSAVTQAQPDPFYMASQLNLPRPCSDYSEMDVEPW 622

GF AQ+ I +T+SSI+C N + + Q D F M S N R C+ MD+ W

Sbjct 642 TGFSEAQLNEIKKMTLSSILCTNLHLVKTQLDSFQMKSDTNQRRECNSLPTMDLTKW 698

>[XP_009049107.1](https://www.ncbi.nlm.nih.gov/protein/XP_009049107.1?report=genbank&log$=protalign&blast_rank=20&RID=0) hypothetical protein LOTGIDRAFT_238662 [Lottia gigantea]

[ESP00222.1](https://www.ncbi.nlm.nih.gov/protein/ESP00222.1?report=genbank&log$=protalign&blast_rank=20&RID=0) hypothetical protein LOTGIDRAFT_238662 [Lottia gigantea]

Length=631

Score = 323 bits (827), Expect = 5e-98, Method: Compositional matrix adjust.

Identities = 206/601 (34%), Positives = 317/601 (53%), Gaps = 72/601 (12%)

Query 47 CSRLTYDQLRYRQIDGRCNHPRN--YGSTGRPVKRYLRPHYQDKFGENLPRVYSVTGQLL 104

C R + YR DG CN+ R+ G + +R + Y D G P+ V LL

Sbjct 69 CRRCNQGKRLYRTADGSCNNVRDPSMGQSLGSFQRLMPAQYGD--GVCTPKTLGVNCGLL 126

Query 105 PSPRMVSWKLHPDQTAHDNNTMLVMQMGQFIDHDITRAPELSGRNASIKCCG-VPPKERL 163

P+ R VS +HP + + NT+++MQ G+F+DHD+T A ++ + + CC V + L

Sbjct 127 PTAREVSKAIHPALSVNAENTVMLMQWGEFMDHDMTGAA-INQPHVNRGCCNHVDVQRGL 185

Query 164 P---------DCFPIDIPPGDPVFE-DCMEFFRSSPAVDNDG--NIIYPREQINALTSFI 211

P +CFPI IP DP F +C+E RS A+ N N PREQ N LTS+I

Sbjct 186 PHPDIATCNGECFPIIIPKDDPYFNINCIEMVRSR-AIQNISYLNSSNPREQQNLLTSYI 244

Query 212 DGSAVYGSDLDTYTWIRSENGTGVFLNTHLVH-GRER----LPSHPHLGPESCVSSNTAE 266

D S VYG++ + +R+ENG +L+ GR++ PS PH

Sbjct 245 DASNVYGTNEEHTKKLRAENGRMATSAGNLLPIGRDQDCILGPSVPH------------- 291

Query 267 SYCQLAGDMRVNEQPGLGSIHLLFHLHHNHIVRLLVAGILKKRGQPSSPERIAKFIQESS 326

C GD R + P L +H +F HN IV L E++

Sbjct 292 --CFATGDERAHAYPPLTGLHTIFVRLHNIIVAEL---------------------DENT 328

Query 327 SALKEQIFQEVRKMLGAIIQKLTYCDWLPMILGPYLIDKFQLGCTRRS--RYNSDLDPRV 384

+ ++FQE RK++GAI+QK TY DWLP IL P + K L RR +Y+ +++ R+

Sbjct 329 NWGGNRLFQETRKIIGAILQKTTYFDWLPYILSPKTLKKNGL---RRGEHKYDINVNARI 385

Query 385 ANSFLSAALRFGHTLIPNVYNFGDKRIHLKDTFNIPDASIRYYDNIIQCLIKEGSEEAYD 444

A+ F +AA R GH+LIP+ +GD++I ++ FN P +++ +I + + A D

Sbjct 386 ASEFATAAYRMGHSLIPDFVKYGDEKILSRNLFNNPKTVFENLTLLMEGVINQPAL-ARD 444

Query 445 RYVSSAVSEHLFESTRGHKHALDLIAVNIQRGRDHGIPAYHYWRQYYRLRRIISL--DEF 502

R+++ +++HLFE+ G + DL++ NI RGRDH I Y+ R+Y LR++ S E

Sbjct 445 RFITEEITDHLFETKNG---SFDLVSFNINRGRDHAIATYNQMREYCGLRKLTSFYDKEV 501

Query 503 GEAGIAMKKAYRDIRDVDLFPGGLLEPSMPGGVVGETFGHILANQFADLKFGDTYFFLHQ 562

G AG+ + K Y + D++LFPGG+ EP++ ++GETF IL QF LKFGD ++ +

Sbjct 502 GNAGVELAKIYDHVDDIELFPGGMSEPNLKNSLLGETFNCILGEQFRCLKFGDAFWHETR 561

Query 563 QAPQGFRAAQIKAILSVTMSSIICANSAVTQAQPDPFYMASQL-NLPRPCSDYSEMDVEP 621

+GF + Q A+ + + I+C + QPDPF + + N +PCS+Y ++++ P

Sbjct 562 DKRRGFSSCQRLALKRFSFAKILCDTLNFNEVQPDPFSLPNDSNNRVKPCSEYDKLNLSP 621

Query 622 W 622

W

Sbjct 622 W 622

>[XP_033756163.1](https://www.ncbi.nlm.nih.gov/protein/XP_033756163.1?report=genbank&log$=protalign&blast_rank=21&RID=0) uncharacterized protein LOC117338907 [Pecten maximus]

Length=1414

Score = 335 bits (858), Expect = 8e-97, Method: Compositional matrix adjust.

Identities = 212/595 (36%), Positives = 318/595 (53%), Gaps = 64/595 (11%)

Query 56 RYRQIDGRCNHPRN--YGSTGRPVKRYLRPHYQDKFGENLPRVYSVTGQLLPSPRMVSWK 113

+YR +G CN+ N YGS+ RP +R L P Y D G + PRV + G LPS R++S

Sbjct 847 KYRSANGTCNNILNPFYGSSFRPHRRSLPPDYDD--GIDSPRVRAENGSPLPSARVISNT 904

Query 114 LHPDQTAHDNNTMLVMQM--GQFIDHDITRAPELSGRNAS-IKCCGVPPKERLPDCFPID 170

LH + + T VM M GQF+DHDIT P + +N + I CC E+ CFPI+

Sbjct 905 LHSTGKSKSSATFTVMYMSFGQFLDHDITSTPSMKDQNNNPIDCCINNNAEQSSACFPIE 964

Query 171 IPPGDPVF-EDCMEFFRSSPAVDNDGNIIYPREQINALTSFIDGSAVYGSDLDTYTWIRS 229

+P D F E CM F RS+ AV+N + Y R+QIN LTS+ID S VYGS L+T +R

Sbjct 965 VPDNDERFAETCMSFTRSAAAVNNGCDPDY-RQQINQLTSYIDASNVYGSTLETQDRLRE 1023

Query 230 ENGTGVFLNTHLVHGRERLPSHPHLGP----ESCVSSNTAESYCQLAGDMRVNEQPGLGS 285

++G GR R+ L P ++C+ NT ++C AGD R +E P L +

Sbjct 1024 KSG-----------GRMRISDQGDLLPSYRNDTCILPNTPGTHCFDAGDERNSEVPTLTT 1072

Query 286 IHLLFHLHHNHIVRLLVAGILKKRGQPSSPERIAKFIQESSSALKEQIFQEVRKMLGAII 345

+H+ F HN I L K + + + EQIFQE RK++GA++

Sbjct 1073 LHIAFLREHNRIADSL------------------KLLNHTWT--DEQIFQEARKIVGALL 1112

Query 346 QKLTYCDWLPMILGPYLIDKFQLGCTR---RSRYNSDLDPRVANSFLSAALRFGHTLIPN 402

Q +TY ++LP ++GP L+D ++L + RS YN +D + N+F AA R+GH+ IP+

Sbjct 1113 QHITYSEYLPRVIGPELMDLYELTPSPSGFRSVYNDTIDATITNAFAGAAFRYGHSQIPD 1172

Query 403 VYNFGDKRIHLKDTFNIPDA----SIRYYDNIIQCLIK---EGSEEAYDRYVSSAVSEHL 455

+KR K+ N+ + I + N + L + ++ D+ + S+V L

Sbjct 1173 QMMLVNKRYKKKEIMNLENQYFKPDIMFEKNGPEWLGRWQVTHAQNKEDKSIQSSVRNFL 1232

Query 456 FESTRGHKHALDLIAVNIQRGRDHGIPAYHYWR--------QYYRLRRIISLDEFGEAGI 507

F ++ +LDL A+N+QRGRDHG+P+Y+ WR +++ R+ +D A

Sbjct 1233 FLDSK--NDSLDLAALNMQRGRDHGLPSYNAWRLWCGLNPVEHFGYRKGGLVDHSRNARK 1290

Query 508 AMKKAYRDIRDVDLFPGGLLEPSMPGGVVGETFGHILANQFADLKFGDTYFFLHQQAPQG 567

+ Y D+DL+ GGL E +PGG+VG TF I++ QF DL+ GD +++ + G

Sbjct 1291 KISSLYSSPNDIDLYSGGLSEEQLPGGLVGPTFACIISRQFRDLQKGDRFWYENDAPHTG 1350

Query 568 FRAAQIKAILSVTMSSIICANSAVTQAQPDPFYMASQLNLPRPCSDYSEMDVEPW 622

F AQ+ I +T+SSI+C N + + Q D F M ++ N + CS MD+ W

Sbjct 1351 FLEAQLNEIKKMTLSSILCTNLNLGKTQQDSFRMKTKTNNRQECSKLPSMDLTKW 1405

Score = 242 bits (618), Expect = 1e-64, Method: Compositional matrix adjust.

Identities = 180/546 (33%), Positives = 268/546 (49%), Gaps = 67/546 (12%)

Query 54 QLRYRQIDGRCNHPRNY--GSTGRPVKRYLRPHYQDKFGENLPRVYSVTGQLLPSPRMVS 111

Q YR G CNH ++ G+ +R + Y+D G + PRV S TG+ LPS R+VS

Sbjct 191 QYPYRTASGYCNHLQDTTQGAALTRQRRMMLNSYED--GIDYPRVRSKTGKELPSARLVS 248

Query 112 WKLHPD---QTAHDNNTMLVMQMGQFIDHDITRAPELSGRN-ASIKCCGVPPK--ERLPD 165

+H + T+++MQ GQFI+HD+ P +SG + A I CCG P

Sbjct 249 NIMHKAGECSLSSKQFTVMIMQFGQFIEHDVISTPMISGTDGADIMCCGGAPNVTAMRSA 308

Query 166 CFPIDIPPGDPVF-EDCMEFFRSSPAVDNDGNIIYPREQINALTSFIDGSAVYGSDLDTY 224

CFPI IPP D F E CM F RS+P + + ++ R +N ++F+DGS +YG+ +

Sbjct 309 CFPISIPPADGRFSESCMTFVRSTPGLKLNCDMGI-RSPMNQASAFMDGSQIYGTSEEET 367

Query 225 TWIRSENGTGVFLNTHLVHGRERLPSHPHLGPESCVSSNTAESYCQLAGDMRVNEQPGLG 284

+R+ G G+ T L LP P E C+ + YC LAGD RVN PGL

Sbjct 368 KGLRALIG-GMLKMTTL-----GLP--PPSQEEMCIKEAPGD-YCMLAGDFRVNHVPGLT 418

Query 285 SIHLLFHLHHNHIVRLLVAGILKKRGQPSSPERIAKFIQESSSALKEQIFQEVRKMLGAI 344

+H F HN I + F + + E+ FQE RK++ A+

Sbjct 419 VLHTTFLREHNRIA--------------------SHFTRINPGWDDEKTFQETRKLIIAM 458

Query 345 IQKLTYCDWLPMILGPYLIDKFQLGCTRRS---RYNSDLDPRVANSFLSAALRFGHTLIP 401

+Q + + + LP IL + ++ + + R Y+ + D + F +AA+RF HT IP

Sbjct 459 LQHMVFNELLPSILNEDHLVRYNIRSSPRGYGDTYDPNTDASIMMGFSAAAMRFPHTRIP 518

Query 402 NVY-----NFGDKRIHLK-DTFNIPDASI----RYYDNIIQCLIKEGSEEAYDRYVSSAV 451

+V +F +R +L TF+ P + + ++ + LI E DR+V V

Sbjct 519 DVQGMVDDSFSSQRNNLIFATFDKPRFILERMGQALNDFARWLISFPVMED-DRFVEDGV 577

Query 452 SEHLFESTRGHKHALDLIAVNIQRGRDHGIPAYHYWRQYYRLRRIISLDEFGEAGIA--- 508

+ LF RGH + DL+A+NIQR RD GIP Y+ WR+ L + + G G+

Sbjct 578 RDFLFLDNRGH--SFDLVALNIQRARDQGIPTYNEWRKLCGLVPA-TFFKAGPGGLVDHE 634

Query 509 ------MKKAYRDIRDVDLFPGGLLEPSMPGGVVGETFGHILANQFADLKFGDTYFFLHQ 562

+ Y D+ D+DLF GGL E ++PG G TF I+A QF +K GD +++ +

Sbjct 635 PDVVRLLSTVYHDVDDIDLFTGGLSEITLPGAATGPTFACIIATQFRSVKVGDRFWYENM 694

Query 563 QAPQGF 568

GF

Sbjct 695 HPITGF 700

>[RUS72077.1](https://www.ncbi.nlm.nih.gov/protein/RUS72077.1?report=genbank&log$=protalign&blast_rank=22&RID=0) hypothetical protein EGW08_020159 [Elysia chlorotica]

Length=589

Score = 314 bits (804), Expect = 4e-95, Method: Compositional matrix adjust.

Identities = 203/572 (35%), Positives = 294/572 (51%), Gaps = 51/572 (9%)

Query 56 RYRQIDGRCNHPRN--YGSTGRPVKRYLRPHYQDKFGENLPRVYSVTGQLLPSPRMVSWK 113

++R+ DGRCN+ N +G+ G + R+ Y + G + PR V+ LP R +S

Sbjct 45 KFRRPDGRCNNLDNPDWGAAGSIMPRFQPSAYAN--GMDSPRTNGVSSNALPGARKISLD 102

Query 114 LHPDQTAHDNNTMLVMQMGQFIDHDITRAPELSGRNASIKCCGVPPKERLPDCFPIDIPP 173

+H A T++VMQ GQF+DHD+T P S CC E CF I IPP

Sbjct 103 VHGPNDATAKITLMVMQWGQFLDHDMTIVPHPS---VDPDCCTNVQAE----CFNIAIPP 155

Query 174 GDPVF--EDCMEFFRSSPA-VDNDGNIIYPREQINALTSFIDGSAVYGSDLDTYTWIRSE 230

D F DCM F RSS VD G EQ N++T++IDGS VYGS + +R

Sbjct 156 EDGYFTGRDCMPFVRSSQTKVDGVG------EQTNSITAYIDGSMVYGSSEEHMQELRGT 209

Query 231 NGTGVFLNTHLVHGRERLPSHPHLGPESCVSSNTAESYCQLAGDMRVNEQPGLGSIHLLF 290

NG L T H + LP+ G +C N +C LAGD RVNEQPGL ++H +F

Sbjct 210 NGR---LRTLQQHRSDYLPA---AGDSAC--RNETGEHCFLAGDERVNEQPGLAAMHTIF 261

Query 291 HLHHNHIVRLLVAGILKKRGQPSSPERIAKFIQESSSALKEQIFQEVRKMLGAIIQKLTY 350

HN I AG L+K S +QIFQ R ++ A IQ +TY

Sbjct 262 VREHNRI-----AGELRKVNTDWS---------------SDQIFQVARDIVIAEIQHITY 301

Query 351 CDWLPMILGPYLIDKFQLGCTRRSRYNSDLDPRVANSFLSAALRFGHTLIPNVYNFGDKR 410

+LP++LGP ++++ L + YN+ +D + N F +AA RFGH+LIP ++

Sbjct 302 DAFLPLVLGPVFMERYNLKTNQPYTYNTSIDAGIRNEFATAAYRFGHSLIPEDITVNNEA 361

Query 411 IHLKDTFNIPDASIRYYDNIIQCLIKEGSEEAYDRYVSSAVSEHLFESTRGHKHALDLIA 470

+ K+ F P + + + L +E S A DR + +V++HLF+ + DL+A

Sbjct 362 LMFKNLFLKPSTVLESMGGLAESLAREPSM-AMDRQFAHSVTQHLFQEE--ERQGSDLVA 418

Query 471 VNIQRGRDHGIPAYHYWRQYYRLRRIISLDEFGEAGIAMKKAYRDIRDVDLFPGGLLEPS 530

+NIQRGRDHG+ + +R+ SL + + Y DI DVDLF GG+ E +

Sbjct 419 LNIQRGRDHGLQPLNAYRKICSGNPFTSLAQLFPRDTTAQATYEDIDDVDLFTGGVAEDA 478

Query 531 MPGGVVGETFGHILANQFADLKFGDTYFFLHQQAPQGFRAAQIKAILSVTMSSIICANSA 590

+ G+VG+TF +LA QF+ L+ GD +F+L++ P F Q++ I VT+ IIC N+

Sbjct 479 VDEGLVGQTFACLLATQFSFLRHGDRFFYLNRDRPNRFNREQMEEIRQVTLGKIICDNTE 538

Query 591 VTQAQPDPFYMASQLNLPRPCSDYSEMDVEPW 622

+ Q D F A N + CS+ +D+ W

Sbjct 539 NDRIQSDVFLPAKVGNERKECSNLPSLDLRKW 570

>[OQV26124.1](https://www.ncbi.nlm.nih.gov/protein/OQV26124.1?report=genbank&log$=protalign&blast_rank=23&RID=0) Chorion peroxidase [Hypsibius dujardini]

Length=945

Score = 323 bits (827), Expect = 5e-95, Method: Compositional matrix adjust.

Identities = 214/602 (36%), Positives = 318/602 (53%), Gaps = 66/602 (11%)

Query 56 RYRQIDGRCNHPRN--YGSTGRPVKRYLRPHYQDKFGENLPRVYSVTGQLLPSPRMVSWK 113

++R+IDG CN+P + +G + P +R+L P Y+D G PR +S+TG +LPS R+VS

Sbjct 342 KFREIDGFCNNPYHPEWGRSFTPFQRFLPPLYED--GIEAPRAHSITGNVLPSARLVSSV 399

Query 114 LHPD-QTAHDNNTMLVMQMGQFIDHDITRAPELS----GRNASIKCCGVP---PKERLPD 165

LHPD H + T+L+M GQF+DHDI P + G+ A+I+CC VP P P

Sbjct 400 LHPDVDRPHCHYTLLLMHFGQFMDHDINHTPATTLTGYGKAANIQCCDVPYGLPAH--PA 457

Query 166 CFPIDIPPGDPVFED----CMEFFRSSPAVDNDGNIIYPREQINALTSFIDGSAVYGSDL 221

CFPI+IP DP + CM F RS A G + PREQ+N LTS++D S +YG+

Sbjct 458 CFPIEIPYSDPYYSKYSLKCMNFVRSE-AAPQPGCSLGPREQLNQLTSYLDASQIYGNTY 516

Query 222 DTYTWIRS-ENGTGVFLNTHLVHGRERLPSHPHLGPESCVSSNTAESYCQLAGDMRVNEQ 280

+ IRS + G + H ++ LP P L C +N A C AGD RVNE

Sbjct 517 EDMKNIRSYQYGRLRTVFVDYCH-KDVLP--PDLETSDCPGAN-ATLPCFRAGDSRVNEN 572

Query 281 PGLGSIHLLFHLHHNHIVRLLVAGILKKRGQPSSPERIAKFIQESSSALKEQIFQEVRKM 340

GL ++H +F HN I L + + + E++FQE RK+

Sbjct 573 TGLATLHTIFTREHNRIADELY--------------YLNPYWDD------ERLFQEARKI 612

Query 341 LGAIIQKLTYCDWLPMILGP---YLIDKFQLGCTRRSRYNSDLDPRVANSFLSAALRFGH 397

LGA++Q + Y +WLP++LG Y D Y+ +++P +AN F +AALRFGH

Sbjct 613 LGAMLQHILYNEWLPLVLGKDVMYQNDLLPQPVGYYGGYDREINPTIANVFATAALRFGH 672

Query 398 TLIPNVYNFGDKRIHLKDTFNIPDASIRYY--------DNIIQCLIKEGSEEAYDRYVSS 449

TLIP+ + F +K + DA + Y D + +I + ++ D +++S

Sbjct 673 TLIPSWFRFFNKHHEYIGQKQLRDAFFKPYPLYQPGVIDMYLLGMINDNIQKR-DSFITS 731

Query 450 AVSEHLFESTRGHKHALDLIAVNIQRGRDHGIPAYHYWRQYYRLRRIISLDEF-----GE 504

V+EHLFE+ + H +DL A+N+QRGRDHGIP Y+ WR++ L + + D+ E

Sbjct 732 EVTEHLFENI-PYAHGVDLAAINVQRGRDHGIPPYNAWREFCGLPKAYTFDDLKDVMRSE 790

Query 505 AGIAMKKAYRDIRDVDLFPGGLLEPSMPGGVVGETFGHILANQFADLKFGDTYFFLHQQA 564

+K Y ++ D+DLFP G+ E + G++G TF IL QF LK GD +++ +

Sbjct 791 IVERLKTVYENVDDIDLFPAGIAEEPLHDGLLGPTFTCILTKQFVHLKRGDRFWYENDIQ 850

Query 565 PQGFRAAQIKAILSVTMSSIICANS-AVTQAQPDPF-YMASQLNLPRPCSD--YSEMDVE 620

PQ F Q++ I ++ +IC NS V QP F ++ +N PC +D+

Sbjct 851 PQAFTPDQLREIRRSNIARVICDNSDDVETIQPHAFLHVLPTVNERVPCKGPFLPRIDLS 910

Query 621 PW 622

W

Sbjct 911 AW 912

>[XP_009052457.1](https://www.ncbi.nlm.nih.gov/protein/XP_009052457.1?report=genbank&log$=protalign&blast_rank=24&RID=0) hypothetical protein LOTGIDRAFT_115464 [Lottia gigantea]

[ESO96969.1](https://www.ncbi.nlm.nih.gov/protein/ESO96969.1?report=genbank&log$=protalign&blast_rank=24&RID=0) hypothetical protein LOTGIDRAFT_115464 [Lottia gigantea]

Length=700

Score = 317 bits (812), Expect = 5e-95, Method: Compositional matrix adjust.

Identities = 212/600 (35%), Positives = 315/600 (53%), Gaps = 72/600 (12%)

Query 57 YRQIDGRCNHPRN--YGSTGRPVKRYLRPHYQDKFGENLPRVYSVTGQLLPSPRMVSWKL 114

YR DG CN+ RN +GS P++R+ P Y D E PR SV G LPS R+VS ++

Sbjct 2 YRTADGTCNNLRNSKWGSAFIPMRRFQPPEYADGVSE--PRTTSVDGSQLPSARLVSREV 59

Query 115 HPD---QTAHDNNTMLVMQMGQFIDHDITRAP-----ELSGRNA-SIKCCGVPPKERLPD 165

H Q + T ++MQ GQF+DHDIT AP + G N I V

Sbjct 60 HETDRGQQEMSSLTHMLMQWGQFVDHDITSAPHQTDPQRKGINVYKIYHKHVSVSLFREA 119

Query 166 CFPIDIPPGDPVFE--DCMEFFRSSPAVDNDGNIIYPREQINALTSFIDGSAVYGSDLDT 223

CFPID+PPGD F C+ F RS D + P EQ+N +T+++DGS VYGS +

Sbjct 120 CFPIDVPPGDRKFRFTSCLNFVRSLQTTDANCRT-EPVEQLNQITAYLDGSMVYGSSQEE 178

Query 224 YTWIRSENGTGVFLNTHLVHGRERLPSH---PHLGPESCVSSNTAESYCQLAGDMRVNEQ 280

+R+ +G GR ++ H P ESCV + A +C AGD RVNEQ

Sbjct 179 QNNLRAFSG-----------GRLKVSDHDLLPEDREESCVKTR-ARDFCFKAGDGRVNEQ 226

Query 281 PGLGSIHLLFHLHHNHIVRLLVAGILKKRGQPSSPERIAKFIQESSSALKEQIFQEVRKM 340

GL S+H ++ HN I LV + + S +++FQE RK+

Sbjct 227 MGLASLHTVWMREHNRIADELV--------------------KLNPSWSDDKVFQEARKI 266

Query 341 LGAIIQKLTYCDWLPMILGPYLIDKFQLGCTRRS---RYNSDLDPRVANSFLSAALRFGH 397

+GA+IQ +T+ +WLP+IL + L R YN ++D + N F +AA RFGH

Sbjct 267 VGALIQHITFTEWLPIILNEEYMGNNNLFVKPRGFYDPYNPNMDASIRNVFATAAFRFGH 326

Query 398 TLIPNVYN-----FGDKRIHL-KDTF----NIPDASIRYYDNIIQCLIKEGSEEAYDRYV 447

+L+ ++ F D HL K+ F +I + + I+ ++ + ++ DR++

Sbjct 327 SLVNTFFSQLSQGFRDNGRHLIKEAFGRTAHILANNGEGVNTYIRGMLIDRPQKG-DRFI 385

Query 448 SSAVSEHLFESTRGHKHALDLIAVNIQRGRDHGIPAYHYWRQYYRLRRIISLDEFG--EA 505

++ +++HLFE T G +LDL ++NIQRGRDHG+P Y+ WR + L S +

Sbjct 386 TTQLTDHLFEDTFGK--SLDLASLNIQRGRDHGLPGYNVWRNWCGLSTSNSFQGMQGLQQ 443

Query 506 GIAMK--KAYRDIRDVDLFPGGLLEPSMPGGVVGETFGHILANQFADLKFGDTYFFLHQQ 563

G A K YR DVD+FPGGL E ++PGG+VG TF I+ANQF ++ GD +++ +

Sbjct 444 GAAQKFSSLYRFTDDVDVFPGGLSEINIPGGMVGSTFACIIANQFRAIREGDRFWY-ERP 502

Query 564 APQGFRAAQIKAILSVTMSSIICANSAVTQAQPDPFYMASQLNLPRPCSDYSEMDVEPWL 623

GF AQ+ +I +S ++CAN+ + + QP+ F+ +Q PC Y ++D+ W+

Sbjct 503 EKTGFTEAQLNSIKRTGLSRVLCANTNIQKIQPNAFWQPTQRKPLEPCESYPDVDLTLWV 562

>[RUS84195.1](https://www.ncbi.nlm.nih.gov/protein/RUS84195.1?report=genbank&log$=protalign&blast_rank=25&RID=0) hypothetical protein EGW08_008035, partial [Elysia chlorotica]

Length=510

Score = 311 bits (796), Expect = 7e-95, Method: Compositional matrix adjust.

Identities = 214/581 (37%), Positives = 294/581 (51%), Gaps = 89/581 (15%)

Query 57 YRQIDGRCNHPRN--YGSTGRPVKRYLRPHYQDKFGENLPRVYSVTGQLLPSPRMVSWKL 114

+R IDG CN+ N +G+ G P KR L Y D N PR SVTG LPS R VS L

Sbjct 1 FRTIDGYCNNLVNPVWGTPGWPFKRILPSRYDDS--SNAPRTKSVTGTHLPSARTVSRLL 58

Query 115 HPDQ---TAHDNNTMLVMQMGQFIDHDITRAPELSGRNASIKCCGVPPKERLP-DCFPID 170

H T + T + Q GQF+DHDI P+ + + LP CF I

Sbjct 59 HTRDSVDTESEKLTSMFTQFGQFLDHDIGLTPK-------TRVSFLMSSRPLPGTCFTIA 111

Query 171 IPPGDPVF-EDCMEFFRS---SPAVDNDGNIIYPREQINALTSFIDGSAVYGSDLDTYTW 226

IP D F + CM F RS SP+ +EQ NA+TSFIDGS +YGS L

Sbjct 112 IPQPDTTFNKTCMNFVRSKVFSPSF---------KEQGNAVTSFIDGSQIYGSSLQQQKS 162

Query 227 IRSENGTGVFLNTHLVHGRERLPSHPHLGPESCVSSNTAESYCQLAGDMRVNEQPGLGSI 286

+R G G E +P GD+RVNEQP L ++

Sbjct 163 LREFRG-----------GNECIPP---------------------PGDVRVNEQPMLSAL 190

Query 287 HLLFHLHHNHIVRLLVAGILKKRGQPSSPERIAKFIQESSSALKEQIFQEVRKMLGAIIQ 346

HL+FH +HNHI LL A + + E++FQE RK++GAI+Q

Sbjct 191 HLVFHRYHNHIADLLSA---------------YNIVWDD-----ERVFQETRKIMGAILQ 230

Query 347 KLTYCDWLPMILGPYLIDKFQLGCTRRSRYNSDLDPRVANSFLSAALRFGHTLIPNVYNF 406

+ Y ++LP +LGP ++ ++L + RY D DPR+ NSF +AA RFGH+++P F

Sbjct 231 HIVYSEYLPPLLGPVIMGNYRLRESIFYRYKPDTDPRMINSFATAAFRFGHSMVPGHLMF 290

Query 407 GDKRIHLKDTFNIPDASIRYYDNIIQCLIK---EGSEEAYDRYVSSAVSEHLFESTRGHK 463

GDKR L F P ++++ LI+ + DR+ S AVS +LF S

Sbjct 291 GDKRKPLDSLFFSPHHIQHSENSVLAGLIRGTVTSPSQKSDRFFSPAVSHNLF-SDEATG 349

Query 464 HALDLIAVNIQRGRDHGIPAYHYWRQYYRLRRIISLDEFGEAGIA-MKKAYRDIRDVDLF 522

+LDL+++NIQRGRDHG+P+Y +R RL + E I K Y DI D+DLF

Sbjct 350 RSLDLVSLNIQRGRDHGLPSYTAFRGACRLPALTG----AEPEIQDFLKVYSDINDIDLF 405

Query 523 PGGLLEPSMPGGVVGETFGHILANQFADLKFGDTYFFLHQQAPQGFRAAQIKAILSVTMS 582

GGL EP + GG VG TF I+A QF DLK+GD ++F + GF Q+K+I V+++

Sbjct 406 SGGLSEPHVYGGAVGPTFACIIAQQFKDLKYGDRFWFETTDSTTGFTTDQLKSIKKVSLA 465

Query 583 SIICANSAVTQAQPDPFYMASQLNLPRPCSDYSEMDVEPWL 623

SI+CA S + F S +N CS+ ++++ W+

Sbjct 466 SILCAASGIKSTPWFVFRKTSIMNPEAACSEIHQLNLSHWI 506

>[XP_002425239.1](https://www.ncbi.nlm.nih.gov/protein/XP_002425239.1?report=genbank&log$=protalign&blast_rank=26&RID=0) Chorion peroxidase precursor, putative [Pediculus humanus corporis]

[EEB12501.1](https://www.ncbi.nlm.nih.gov/protein/EEB12501.1?report=genbank&log$=protalign&blast_rank=26&RID=0) Chorion peroxidase precursor, putative [Pediculus humanus corporis]

Length=924

Score = 320 bits (821), Expect = 2e-94, Method: Compositional matrix adjust.

Identities = 210/600 (35%), Positives = 333/600 (56%), Gaps = 68/600 (11%)

Query 57 YRQIDGRCNHPRN--YGSTGRPVKRYLRPHYQDKFGENLPRVYSVTGQLLPSPRMVSWKL 114

YR +DG CN+ ++ +GS +P+KR+L PHY+D E + R S+ G LPS R+VS ++

Sbjct 316 YRTMDGTCNNLKHPWWGSRFQPLKRFLAPHYEDGL-EAIRR--SIKGGPLPSARLVSTEV 372

Query 115 HPDQTAHDNN-TMLVMQMGQFIDHDITRAPELSGRNASI-KCCGVPPKERLPDCFPIDIP 172

H D+ + T L+MQ GQF+DHD+T + + G S+ +CC PP + PDC PI++

Sbjct 373 HWDKNVESKSVTHLLMQWGQFLDHDMTSSSQSRGFGGSVPRCCDAPPDLQHPDCLPIEVL 432

Query 173 PGDPVFED----CMEFFRSSPAVDNDGNIIYPREQINALTSFIDGSAVYGSDLDTYTWIR 228

P D F C+EF RS+P+ G + PREQIN +TS+IDGS +YG+ + +R

Sbjct 433 PDDRFFSRFGIRCLEFLRSAPS-SRVGCALGPREQINQVTSYIDGSMIYGNSERESSKLR 491

Query 229 SENGTGVFLNTHLVHGR--ERLPSHP--HLGPESCVSSNTAESYCQLAGDMRVNEQPGLG 284

+F N L + R +RLP P E C S + + +C +GD R+NEQPGL

Sbjct 492 ------LFRNGMLKYTRMPQRLPLLPIDRDKGEFCRKS-SPDFFCLHSGDSRMNEQPGLL 544

Query 285 SIHLLFHLHHNHIVRLLVAGILKKRGQPSSPERIAKFIQESSSALKEQIFQEVRKMLGAI 344

+IH++F HN + R L +PE E++FQE RK++GAI

Sbjct 545 AIHIVFLRLHNRLTRNLAH---------LNPE-----------WNDERLFQETRKIVGAI 584

Query 345 IQKLTYCDWLPMILGPYLIDKFQLGCTRR---SRYNSDLDPRVANSFLSAALRFGHTLIP 401

IQ +TY ++LP++LG ++ F + + Y+ + ANSF +AA RFGH+++

Sbjct 585 IQHITYREFLPIVLGQDVMSIFGIDLLKTGYYQNYSESVSSTAANSFATAAFRFGHSMVQ 644

Query 402 NVY-NFGDKR------IHLKDTFNIPDASIRY--YDNIIQCLIKEGSEEAYDRYVSSAVS 452

+ + D R + L D + R+ DN++ + ++G+++ D + + ++

Sbjct 645 GKFLKWDDIRKEEIINVSLHDELLDTGSLHRFGAVDNLMLGMCRQGAQKR-DEHFTVELT 703

Query 453 EHLFESTRGHKHALDLIAVNIQRGRDHGIPAYHYWRQYYRLRRIISLDEF-----GEAGI 507

HLF+ T LDL A+NIQRGRDHG+P+Y+ WR LR++ + ++ E+

Sbjct 704 NHLFQ-TPNFPAGLDLAAINIQRGRDHGLPSYNSWRNPCGLRKMKNWNDLLNVMSQESRD 762

Query 508 AMKKAYRDIRDVDLFPGGLLEPSMPGGVVGETFGHILANQFADLKFGDTYFFLHQQAPQG 567

A+++ YRD+ DVDL+ GGL E S+ GG+VG TF I+ F +L+ GD +++ +

Sbjct 763 ALRRIYRDVNDVDLYTGGLAEFSVRGGLVGSTFACIIGQHFRNLRKGDRFWYENGGFESS 822

Query 568 FRAAQIKAILSVTMSSIICANSAVTQAQPDPFYMASQLNLPRP-----CSDYSEMDVEPW 622

F AQ+KAI VT++ I+C N +++ + PF ++ NL P + +D+ PW

Sbjct 823 FTVAQLKAIRRVTLARILCDNLDLSE-EIQPFVFLTEDNLRNPKISCRSREIPFIDLTPW 881

>[XP_033761598.1](https://www.ncbi.nlm.nih.gov/protein/XP_033761598.1?report=genbank&log$=protalign&blast_rank=27&RID=0) myeloperoxidase-like [Pecten maximus]

Length=770

Score = 315 bits (807), Expect = 1e-93, Method: Compositional matrix adjust.

Identities = 200/588 (34%), Positives = 297/588 (51%), Gaps = 58/588 (10%)

Query 57 YRQIDGRCNHPRN--YGSTGRPVKRYLRPHYQDKFGENLPRVYSVTGQLLPSPRMVSWKL 114

+R G CN+ N YG+ R+L P Y D G PR Q LPS R+VS +

Sbjct 217 FRSASGTCNNLANPEYGAAATAQSRFLPPQYGDGIGS--PRTQGKNSQPLPSARVVSNSV 274

Query 115 HPDQTAHDNN--TMLVMQMGQFIDHDITRAPELSGRNASIKCCGVPPKERLPDCFPIDIP 172

+++VM GQF+DHDIT P SG ++ C LP+CFPI IP

Sbjct 275 FKSSKEKKETKLSLMVMAWGQFLDHDITLTPSTSGAEGTLTHCCNSSVATLPECFPITIP 334

Query 173 PGDPVF--EDCMEFFRSSPAVDNDGNIIYP--REQINALTSFIDGSAVYGSDLDTYTWIR 228

D F +CMEF RS+ + P REQ NA+T+F+DGS VYGS LD +R

Sbjct 335 NNDDHFTNSNCMEFVRSAAVTE----ACSPNHREQFNAITAFVDGSNVYGSSLDQMNGLR 390

Query 229 SENGTGVFLNTHLVHGRERLPSHPHLGPESCVSSNTAESYCQLAGDMRVNEQPGLGSIHL 288

+ N G+ + + G LP P P SCV ++T E +C GD+R N P LG+ H+

Sbjct 391 AFN-KGLLKTSSVSSG---LP--PEGDPHSCVLNSTTE-FCINTGDIRANVVPHLGANHV 443

Query 289 LFHLHHNHIVRLLVAGILKKRGQPSSPERIAKFIQESSSALKEQIFQEVRKMLGAIIQKL 348

LF HN I +L + ++ E++FQE RK++ I+Q++

Sbjct 444 LFFREHNRIAEILSS--------------------MNTYWNDERVFQETRKIVSGILQQI 483

Query 349 TYCDWLPMILGPYLIDKFQLGCTRRSRYNSDLDPRVANSFLSAALRFGHTLIPNV----- 403

+Y +WLP IL P + KF+L + + Y D++P + N F AA+RFGH+LIP +

Sbjct 484 SYYEWLPSILSPSYLGKFELKKSNKDLYKPDVNPNIRNEFAVAAMRFGHSLIPPLEGYLL 543

Query 404 --YNFGDKRIHLKDTFNIPDASIRYYDNIIQCLIK---EGSEEAYDRYVSSAVSEHLFES 458

Y + + +++TF P I I L + S DR + V + LF

Sbjct 544 HDYATWEVQTPIEETFFRPSMVIGNAGQDIPKLARWMCTNSSMKVDRRFEAGVKDMLFLD 603

Query 459 TRGHKHALDLIAVNIQRGRDHGIPAYHYWRQYYRLRRIISLDEFG--EAGIA--MKKAYR 514

+ G + DL A+NIQRGRDHGIP Y+ + + + L+++ + + GI +K Y

Sbjct 604 SNG--MSFDLAALNIQRGRDHGIPPYNAYMERFGLKKLKNFKKMTNHNDGIKDMLKAVYS 661

Query 515 DIRDVDLFPGGLLEPSMPGGVVGETFGHILANQFADLKFGDTYFFLHQQAPQGFRAAQIK 574

+ D+DL+ GG+ E P G +G TF I+A QF +K GD ++F + AP+GF + K

Sbjct 662 HVDDIDLYVGGMTEKKKPNGNLGPTFSEIIARQFKKIKIGDRFWF-ERPAPEGFSPVKRK 720

Query 575 AILSVTMSSIICANSAVTQAQPDPFYMASQLNLPRPCSDYSEMDVEPW 622

I +T++ ++C N + D F++ S+ N + C EMD+ W

Sbjct 721 EIRKMTLAKVMCTNFGMDLITKDVFHVQSERNKLKYCDSIEEMDLTKW 768

>[RUS74146.1](https://www.ncbi.nlm.nih.gov/protein/RUS74146.1?report=genbank&log$=protalign&blast_rank=28&RID=0) hypothetical protein EGW08_018101 [Elysia chlorotica]

Length=668

Score = 311 bits (797), Expect = 4e-93, Method: Compositional matrix adjust.

Identities = 200/589 (34%), Positives = 300/589 (51%), Gaps = 63/589 (11%)

Query 45 EHCSRLTYDQLRYRQIDGRCNHPRN--YGSTGRPVKRYLRPHY---QDKFGENLPRVYSV 99

E + +T D YR DG CN+ N +GS R L P Y D FG L +V

Sbjct 112 ETDASITCDSDLYRSADGSCNNKANPFWGSRDSAFLRTLEPVYTEANDVFGPVL--ASTV 169

Query 100 TGQLLPSPRMVSWKLHPDQTAHDNNTMLVMQMGQFIDHDITRAPELSGRNASIKCC---- 155

G LPSPR++S +H D ++ MQ GQF+DHDIT P N S + C

Sbjct 170 DGYALPSPRLISRIVHDPDDFPDRRPVMNMQWGQFLDHDITSTPV----NPSTETCCYDG 225

Query 156 ----GV---PPKERLPDCFPIDIPPGDPVFED----CMEFFRSSPAVDNDGNIIYPREQI 204

GV P + CFPI IP GD F CMEF RS + N EQI

Sbjct 226 IVASGVAQHPQVAKNGPCFPILIPAGDNHFTTLETRCMEFKRSYQTMVNGK-----PEQI 280

Query 205 NALTSFIDGSAVYGSDLDTYTWIRSEN-GTGVFLNTHLVHGRERLPSHPHLGPESCVSSN 263

N T+F+DGS +YGS + +RS G G L+ + + LP + + CV SN

Sbjct 281 NLNTAFVDGSQIYGSTEEEMDELRSSALGRGRLLDIN-----DNLPKNTD---DVCVVSN 332

Query 264 TAESYCQLAGDMRVNEQPGLGSIHLLFHLHHNHIVRLLVAGILKKRGQPSSPERIAKFIQ 323

+ YC AGD RVN PGLG++H +F HN I L A

Sbjct 333 STTDYCLRAGDSRVNVYPGLGALHTVFLRMHNRIADGLAA-------------------- 372

Query 324 ESSSALKEQIFQEVRKMLGAIIQKLTYCDWLPMILGPYLIDKFQLGCTRRSRYNSDLDPR 383

++ S E++FQ+ R+++GA++QK+TY +WLP ++GP ++L T Y D +P

Sbjct 373 QNKSLTDEEVFQKARRIVGALLQKITYSEWLPTVVGPDAATTYRLASTVPYAYEPDTNPT 432

Query 384 VANSFLSAALRFGHTLIPNVYNFGDKRIHLKDTFNIPDASIRYYDNIIQCLIKEGSEEAY 443

+ + F +AA R+GH+ + + + I + FN P + D+++ ++ +

Sbjct 433 LHSVFSTAAFRYGHSQVTDTLTINNVAIESVNLFNNPHHVLHSIDSLLAGILGNPGQRV- 491

Query 444 DRYVSSAVSEHLFESTRGHKHALDLIAVNIQRGRDHGIPAYHYWRQYYRLRRIISLDEFG 503

DR+ S ++ H+FE+ LD++++NIQRGRDHG+P Y+ WR+ L I S D+ G

Sbjct 492 DRWYSDGMTNHVFETV--PFKGLDIVSLNIQRGRDHGLPRYNQWRKECGLDPITSFDDMG 549

Query 504 EAGIAMKKAYRDIRDVDLFPGGLLEPSMPGGVVGETFGHILANQFADLKFGDTYFFLHQQ 563

G ++ YR+ D+DL+ G L E + GVVG+T+ +++ QF LKFGD +++ +

Sbjct 550 RHGRPFQRVYRNTDDIDLYSGALHEAPVHLGVVGDTYACLISRQFEKLKFGDRFWYQNPD 609

Query 564 APQGFRAAQIKAILSVTMSSIICANSAVTQAQPDPFYMASQLNLPRPCS 612

AP+ F +AQIK I ++++IIC + + Q + F ++ N CS

Sbjct 610 APKAFTSAQIKEIEKQSLATIICQTTGLESVQSNAFMNVARNNRLVYCS 658

>[XP_033762435.1](https://www.ncbi.nlm.nih.gov/protein/XP_033762435.1?report=genbank&log$=protalign&blast_rank=29&RID=0) lactoperoxidase-like [Pecten maximus]

Length=712

Score = 310 bits (795), Expect = 2e-92, Method: Compositional matrix adjust.

Identities = 203/597 (34%), Positives = 302/597 (51%), Gaps = 68/597 (11%)

Query 57 YRQIDGRCN---HPRNYGSTGRPVKRYLRPHYQDKFGENLPRVYSV-TGQLLPSPRMVSW 112

YR IDG+CN HP +G+ G RYL +Y D G PR S LPSPR+VS

Sbjct 150 YRTIDGQCNNLKHP-TWGAAGTQQGRYLPANYSDGIGA--PRSESSRDSSPLPSPRLVSN 206

Query 113 KLHPDQTAHDNN---TMLVMQMGQFIDHDITRAPELSGRNAS-IKCCGVPPKERL----- 163

+ + ++ +++VM GQF+DHDIT P SG S I+CC R

Sbjct 207 VVFANTSSEKKEKKLSLMVMAWGQFLDHDITLTPTASGEEGSQIECCDETGSGRYGAYKW 266

Query 164 ----PDCFPIDIPPGDPVFE-DCMEFFRSSPAVDNDGNIIYPREQINALTSFIDGSAVYG 218

DCFPIDIP D F+ CM F RSS VD+ G + R+Q+NA+T+F+DGS VYG

Sbjct 267 EPSRDDCFPIDIPKNDNHFKTSCMSFVRSSAVVDDCGPAM--RQQVNAITAFVDGSNVYG 324

Query 219 SDLDTYTWIRSENGTGVFLNTHLVHGRERLPSHPHLGPESCVSSNTAESYCQLAGDMRVN 278

S + + +R +F N L+ LP P +SC+ S + +C AGD+R N

Sbjct 325 SSEEEVSSLR------LFTNGMLLSSPGNLP--PKGSEDSCIISKPND-FCIKAGDVRAN 375

Query 279 EQPGLGSIHLLFHLHHNHIVRLLVAGILKKRGQPSSPERIAKFIQESSSALKEQIFQEVR 338

P LG+ H+LF HN I R L L + E+ FQ R

Sbjct 376 VVPHLGANHVLFFREHNRIAREL--STLNPKWS------------------DEETFQTTR 415

Query 339 KMLGAIIQKLTYCDWLPMILGPYLIDKFQLGCTRRSRYNSDLDPRVANSFLSAALRFGHT 398

K++ A++Q+++Y +WLP +L +D + L ++ Y+S +DP + NSF AA+RFGH+

Sbjct 416 KIVSALLQQISYYEWLPSLLPSEFLDSYNLTRSKGDPYHSTVDPSIKNSFAVAAMRFGHS 475

Query 399 LIPN-----VYNFGDKRIH-LKDTFNIPDASIRYYDNIIQCLIK---EGSEEAYDRYVSS 449

L+ +Y++ ++ +++TF P + N + L + DR

Sbjct 476 LVSGFQATLLYDYMTYKVTPIEETFFRPSMVVGDSGNDVPLLARWVCANESMRRDRIFER 535

Query 450 AVSEHLFESTRGHKHALDLIAVNIQRGRDHGIPAYHYWRQYYRLRRIISLDEFGEAGI-- 507

+ + LF + G H+ DL A+N+QRGRDHGIP+Y+ WRQ+ + R + E

Sbjct 536 GIRDLLFLDSEG--HSFDLGALNLQRGRDHGIPSYNDWRQWVGIPRATTFSELKNHSKRE 593

Query 508 --AMKKAYRDIRDVDLFPGGLLEPSMPGGVVGETFGHILANQFADLKFGDTYFFLHQQAP 565

+K Y+ + D+DL+ GG+ E + GG +G F HILA QF +LK GD +F+ +

Sbjct 594 KRLLKSVYKHVDDIDLYAGGISEDDVKGGHLGPVFSHILARQFHELKAGDRFFY-ERPTK 652

Query 566 QGFRAAQIKAILSVTMSSIICANSAVTQAQPDPFYMASQLNLPRPCSDYSEMDVEPW 622

+GF+ AQ++ I V +S I+C N + D F + + C MD+ W

Sbjct 653 EGFKKAQLEQIRKVKLSKIMCENFGLDLIPEDVFKIVDSGKTLKSCESLPGMDLSKW 709

>[XP_012940602.1](https://www.ncbi.nlm.nih.gov/protein/XP_012940602.1?report=genbank&log$=protalign&blast_rank=30&RID=0) chorion peroxidase [Aplysia californica]

Length=597

Score = 306 bits (784), Expect = 4e-92, Method: Compositional matrix adjust.

Identities = 206/610 (34%), Positives = 306/610 (50%), Gaps = 57/610 (9%)

Query 35 SLDFVSEADLEHCSRLTYD-----QLRYRQIDGRCNHPR--NYGSTGRPVKRYLRPHYQD 87

S F +E ++ C R D YRQ DG CN+ + G T P +R L P Y

Sbjct 18 SFTFSAERAIDGCQRAISDISCDVSYPYRQADGSCNNIKQPTRGRTNSPFRRLLSPVYDQ 77

Query 88 KFGENLPRVYSVT--GQLLPSPRMVSWKLHPDQTA-HDNNTMLVMQMGQFIDHDITRAPE 144

G N PR+ SV LPSPR VS +HP A D+ T+++MQ GQF+DHDIT P+

Sbjct 78 NDG-NTPRLVSVAPGNPPLPSPRTVSTNVHPPGNALSDDATVMLMQWGQFLDHDITHTPQ 136

Query 145 LSGRNASIKCCGVPPKERL-PD------CFPIDIPPGDPVFED----CMEFFRSSPAVDN 193

S N + C G+ PD C PI P DP F C+ F RS +D+

Sbjct 137 TSSPN-KLCCSGLTNGSDFHPDVRSGGPCHPIMAPEDDPYFNTQNTRCISFTRSEGVLDD 195

Query 194 DGNIIYPREQINALTSFIDGSAVYGSDLDTYTWIRS-ENGTGVFLNTHLVHGRERLPSHP 252

+ R+Q + +T F+DGS +YGS +RS ENG L +++G E LP

Sbjct 196 ----MSIRQQYSDITPFLDGSQIYGSSETHARSLRSFENGK---LRAKIINGEEFLPKWE 248

Query 253 HLGPESCVSSNTAESYCQLAGDMRVNEQPGLGSIHLLFHLHHNHIVRLLVAGILKKRGQP 312

C + YC LAGD+RVN PGL ++H +F +HN + L A

Sbjct 249 D--DSQCFKLEPGD-YCFLAGDVRVNVYPGLNALHTVFLRYHNLLCERLKAA-------- 297

Query 313 SSPERIAKFIQESSSALKEQIFQEVRKMLGAIIQKLTYCDWLPMILGPYLIDKFQL-GCT 371

E+++Q+ R+++GAI+QK++Y D+L +LG + + L

Sbjct 298 ------------HEDWTDEKLYQQARRIVGAILQKVSYSDYLKTLLGETVAKIYDLLPGD 345

Query 372 RRSRYNSDLDPRVANSFLSAALRFGHTLIPNVYNFGDKRIHLKDTFNIPDASIRYYDNII 431

YN +DP ++N+F +AA RFGH+ IP+ G++ + + P + D++I

Sbjct 346 GDYAYNDIVDPTLSNAFSTAAYRFGHSSIPDDLQIGNETVPTGKLYLRPKFVLNGLDDVI 405

Query 432 QCLIKEGSEEAYDRYVSSAVSEHLFESTRGHKHALDLIAVNIQRGRDHGIPAYHYWRQYY 491

+ L EG ++ DR S V++ LFE + DL+A+NIQRGRDHG+P Y+ +R++

Sbjct 406 KAL-SEGKQQRVDRTYSRGVTDQLFEPPKRPGKGFDLVALNIQRGRDHGLPPYNAFRRFC 464

Query 492 RLRRIISLDEFGEAGIAMKKAYRDIRDVDLFPGGLLEPSMPGGVVGETFGHILANQFADL 551

L + K Y D+DL+ GGL E + GG +GE F ILA QF DL

Sbjct 465 GLPAYARFSDLQSGQSEFSKVYASPDDIDLYSGGLSEKPVQGGRIGEVFTCILALQFRDL 524

Query 552 KFGDTYFFLHQQAP-QGFRAAQIKAILSVTMSSIICANSAVTQAQPDPFYMASQLNLPRP 610

KFGD +++ +Q P F AQI + S +++ +IC + +++ QP+ F N

Sbjct 525 KFGDRFWYENQADPTTAFTPAQIAQVNSFSLAKVICDVTGISRIQPNLFLTPVGSNTRMR 584

Query 611 CSDYSEMDVE 620

CS Y ++++E

Sbjct 585 CSGYRDLNIE 594

>[XP_034310733.1](https://www.ncbi.nlm.nih.gov/protein/XP_034310733.1?report=genbank&log$=protalign&blast_rank=31&RID=0) eosinophil peroxidase [Crassostrea gigas]

Length=693

Score = 308 bits (790), Expect = 7e-92, Method: Compositional matrix adjust.

Identities = 213/592 (36%), Positives = 291/592 (49%), Gaps = 65/592 (11%)

Query 57 YRQIDGRCNHPRN--YGSTGRPVKRYLRPHYQDKFGENLPRVYSVTGQLLPSPRMVS--- 111

YR +DG CN+ N +G+ P RY Y D G N PR + G LPSPR++S

Sbjct 138 YRSVDGSCNNLNNPHWGAAVTPQPRYQPAQYDD--GVNSPRTTATDGSPLPSPRLISNNL 195

Query 112 WKLHPDQTAHDN-NTMLVMQMGQFIDHDITRAPELSGRNASIKCCGVPPKERLPDCFPID 170

++ D T D+ T++VM GQFIDHDI P G A I CCG + R+ DC PI

Sbjct 196 FRAPGDCTETDHARTLMVMAWGQFIDHDIVATPVTQGDGAPITCCGTEVQNRV-DCLPIP 254

Query 171 IPPGDPVFED-CMEFFRSSPAVDNDGNIIYPREQINALTSFIDGSAVYGSDLDTYTWIR- 228

IP DP F D CMEF RS+PA DG PREQIN +TSFIDG VYG W+

Sbjct 255 IPSDDPHFNDTCMEFVRSAPAPAADGCEAGPREQINQITSFIDGGVVYGD--TNMKWLEL 312

Query 229 SENGTGVFLNTHLVHGRERLPSHPHLGPESCVSSNTAESYCQLAGDMRVNEQPGLGSIHL 288

+ TG L + + LPS C S++ E +CQLAGD RVN P LG HL

Sbjct 313 VDTNTGSMLTSD----GDLLPSG-----GGCRLSDS-EDFCQLAGDHRVNVIPSLGGNHL 362

Query 289 LFHLHHNHIVRLLVAGILKKRGQPSSPERIAKFIQESSSALKEQIFQEVRKMLGAIIQKL 348

+F HN IV L +R +P +FQE RK++GA++Q++

Sbjct 363 VFVREHNRIVEEL------RRVRPDWD--------------AATVFQETRKIIGALLQQI 402

Query 349 TYCDWLPMILGPYLIDKFQLG---CTRRSRYNSDLDPRVANSFLSAALRFGHTLIPN--V 403

Y ++LP IL + K+ L S YNS +P N F +AA RFGH+ +P

Sbjct 403 NYREFLPSILREEDLVKYNLKLQLTGHSSSYNSSRNPGAKNVFNAAAFRFGHSQVPGSIA 462

Query 404 YNFGDKRIHLKDTFN---------IPDASIRYYDNIIQCLIKEGSEEAYDRYVSSAVSEH 454

Y D L+ T + S R ++ + ++ S + D + A+ +H

Sbjct 463 YVLRDFMTRLESTPTESTLLDPHMLITRSGRNVADLARFVVTSNSMKV-DSHFEDALRDH 521

Query 455 LFESTRGHKHALDLIAVNIQRGRDHGIPAYHYWRQYYRLRRIISLDEF----GEAGIAMK 510

LFE G DL A+N+QRGRDHG+P Y+ WRQ+ L S + E

Sbjct 522 LFEGPDG--VGFDLGALNLQRGRDHGLPPYNAWRQWCGLPVATSFSDLPDMSDENKAVFA 579

Query 511 KAYRDIRDVDLFPGGLLEPSMPGGVVGETFGHILANQFADLKFGDTYFFLHQQAPQGFRA 570

Y D+ D+D+F GG+ E + G VG F I+ NQF D+K GD Y++ + +GFR

Sbjct 580 DLYSDVDDIDVFAGGVAETPLDGAAVGPLFSCIIGNQFRDMKEGDRYWY-ENRGREGFRR 638

Query 571 AQIKAILSVTMSSIICANSAVTQAQPDPFYMASQLNLPRPCSDYSEMDVEPW 622

Q+ I V + I+C N V QPD F++ + N + C ++ W

Sbjct 639 EQLAEIRKVRFAKILCDNLGVDPIQPDVFHVPNPNNSWQSCQSLPGINFSAW 690

>[XP_035658181.1](https://www.ncbi.nlm.nih.gov/protein/XP_035658181.1?report=genbank&log$=protalign&blast_rank=32&RID=0) peroxidase mlt-7-like [Branchiostoma floridae]

Length=709

Score = 308 bits (789), Expect = 1e-91, Method: Compositional matrix adjust.

Identities = 210/621 (34%), Positives = 317/621 (51%), Gaps = 86/621 (14%)

Query 47 CSRLTY--DQLRYRQIDGRCNHPRN--YGSTGRPVKRYLRPHYQDKFGENLPRVYSVTGQ 102

C L + D +R DG N+ +GST + +KR L P Y + +N PR + G+

Sbjct 128 CEELQHMEDGKEFRTADGCGNNEDKSWWGSTQQCMKRLLEPQYGN---DNAPRTTGLDGK 184

Query 103 LLPSPRMVSWKLHPD-QTAHDNNTMLVMQMGQFIDHDITRAPELSGRNASIKCCGVPPKE 161

LP R VS +H D + ++ ++++MQ GQF H+IT +P +A+ C P+

Sbjct 185 SLPKARHVSRVMHEDLRKSNHETSLMLMQFGQFTSHEITMSPV---SDATCSCGSSDPQ- 240

Query 162 RLPDCFPIDIPPGDPVFE--DCMEFFRS--SPAVDNDGNIIYPREQINALTSFIDGSAVY 217

CF I+IP GDP F C++F RS P V+NDG + PR+Q + +TSF+D S VY

Sbjct 241 ----CFNIEIPHGDPDFRGRTCLKFTRSVPCPKVENDGGCMGPRQQFDQITSFLDASNVY 296

Query 218 GSDLDTYTWIRSENGTGVFLNTHLVHGRERLPSHPHLGPESCVSSNTAES---------Y 268

G +R G L G S L PE+ + A S

Sbjct 297 GLTKKDMDDLRH----GFLLTPRSNSGN----SKKELLPEAVDNDKFAMSCEGFTDDVHK 348

Query 269 CQLAGDMRVNEQPGLGSIHLLFHLHHNHIVRLLVAGILKKRGQPSSPERIAKFIQESSSA 328

C AGD+R NE PGL S+H LF HN I R L K G P+R+

Sbjct 349 CSRAGDIRANENPGLTSLHTLFMREHNRIARKLNTLNSPKWG----PDRV---------- 394

Query 329 LKEQIFQEVRKMLGAIIQKLTYCDWLPMILGPYLIDKFQLGCTRRSRY----NSDLDPRV 384

F E RK++GA+ QK+ Y D+LP++LGP + KF L T+ +Y + ++P +

Sbjct 395 -----FFEARKIVGALFQKIAYGDYLPLVLGPDFMTKFVLTLTQPDKYFQGYDKTVNPGI 449

Query 385 ANSFLSAALRFGHTLIPNVYN-----FGDKRIHLKDTFN----------IPDASIRYYDN 429

N+F +AA RFGH+++ N ++ F D R++ + N I D D+

Sbjct 450 YNAFNTAANRFGHSMVQNEFDRYSKGFPDARLNSQHPINLAFSFFNPSYILDDDQGGPDS 509

Query 430 IIQCLIKEGSEEAYDRYVSSAVSEHLFESTRGHKHALDLIAVNIQRGRDHGIPAYHYWRQ 489

I++ LI S + +DR++ S +++HLFE + + DL A+NIQRGRDHG+P Y+ +R+

Sbjct 510 ILRGLITAQSRQDFDRFIVSGLTKHLFECPKIPNISFDLAAINIQRGRDHGLPGYNAFRE 569

Query 490 YYRLRRIISLDEF------GEAGIAMKKAYRDIRDVDLFPGGLLEPSMPGGVVGETFGHI 543

L R D+ ++ YRD+ D+DLF GGL E S+PGG+VG TF ++

Sbjct 570 KCGLHRAPRFDDLSPEIPDATTREKLQTLYRDVDDIDLFVGGLAEKSVPGGIVGPTFAYL 629

Query 544 LANQFADLKFGDTYFFLHQQAPQGFRAAQIKAILSVTMSSIICANSAVTQAQPDPFYMAS 603

+ QF D++ GD ++F + P F Q+K I +++SI+C N+ T QP+ F +

Sbjct 630 IGMQFHDIRKGDRFWF---ENPGQFSEDQLKEIKKHSLASILCDNTDTTDIQPNVFLQHT 686

Query 604 QLNLPR-PCSD-YSEMDVEPW 622

Q R C++ +MD+ W

Sbjct 687 QPGNDRVKCTEILPKMDLSLW 707

>[XP_021361183.1](https://www.ncbi.nlm.nih.gov/protein/XP_021361183.1?report=genbank&log$=protalign&blast_rank=33&RID=0) myeloperoxidase-like [Mizuhopecten yessoensis]

Length=711

Score = 307 bits (787), Expect = 3e-91, Method: Compositional matrix adjust.

Identities = 212/607 (35%), Positives = 314/607 (52%), Gaps = 70/607 (12%)

Query 48 SRLTYDQL-RYRQIDGRCNHP--RNYGSTGRPVKRYLRPHYQDKFGENLPRVYSVTGQLL 104

+ LT + L RYR IDG CN+P +G G RYLRP Y D G N PR+ G L

Sbjct 130 ANLTCNALSRYRTIDGSCNNPFQPTWGMAGTQQTRYLRPVYND--GINSPRLVGRDGIPL 187

Query 105 PSPRMVSWKLHPDQTAHDNN---TMLVMQMGQFIDHDITRAPELSGRN-ASIKCCGVPPK 160

PS RM+S +H + + + +VMQ GQF+DHDI P L N +S +CC

Sbjct 188 PSARMISNIVHSSKGFVRQDCHLSAMVMQWGQFLDHDIVGTPVLKKDNGSSYECCEA--D 245

Query 161 ERLPDCFPIDIPPGDPVFE-DCMEFFRSSPAVDNDGNIIYPREQINALTSFIDGSAVYGS 219

CFP+DIPP D FE CM RS+PAV D I REQ+N +TS+ID SAVYGS

Sbjct 246 SSFEGCFPVDIPPNDDHFEGKCMNMVRSAPAVGPDC-AIGAREQMNKVTSYIDASAVYGS 304

Query 220 DLDTYTWIRSENGTGVFLNTHLVHGRERLPSHPHLGPESCVSSNTAESYCQLAGDMRVNE 279

+ ++R+ N L G RL P G ++C+ A+ C AGD R N

Sbjct 305 SREEEAFLRAYN------RGMLKEGDNRL--LPPSGRDNCIQRTPAD-VCMTAGDDRPNV 355

Query 280 QPGLGSIHLLFHLHHNHIVRLLVAGILKKRGQPSSPERIAKFIQESSSALKEQIFQEVRK 339

P LGSIH LF HN I +L G+L + F + E+IFQE R+

Sbjct 356 VPSLGSIHTLFMREHNRIAAML--GVLNR------------FWDD------ERIFQETRQ 395

Query 340 MLGAIIQKLTYCDWLPMILGPYLIDKFQLGCTRRSR---YNSDLDPRVANSFLSAALRFG 396

++ A++Q +TY ++LP+ILG +++ +++ T + YN+ ++ +AN F AA RFG

Sbjct 396 IMSALLQHITYYEYLPLILGKRIMEYYKILPTTEYKANIYNTSINAGIANVFAVAAFRFG 455

Query 397 HTLIPNVYNFGDKRIHLKDTFNIPDASIRYY----------DNIIQCLIKEGSEEAYDRY 446

H+ IP++ F R + F I R + +++++ L+K+ E +R

Sbjct 456 HSQIPSIQTFLGPRESYRHDFRIETTYHRPFFAQFKNGQGTEDVLRWLVKDSQPET-NRV 514

Query 447 VSSAVSEHLFESTRGHKHALDLIAVNIQRGRDHGIPAYHYWRQYYRLR--RIISLDEFG- 503

V + LF + +LDL A+NIQRGRDHG+P Y+ WR++ L R ++ G

Sbjct 515 FVRGVRDQLFLR---NNVSLDLAAINIQRGRDHGVPPYNAWRKWCGLAPARHFGVEPDGL 571

Query 504 -----EAGIAMKKAYRDIRDVDLFPGGLLEPSMPGGVVGETFGHILANQFADLKFGDTYF 558

+ +++AYRD D+D+FPG + E + G VG TF IL QF +LK GD ++

Sbjct 572 IHHTIASARLLQRAYRDPDDIDVFPGAISERHLKGASVGPTFACILGYQFQELKQGDRFW 631

Query 559 FLHQQAPQGFRAAQIKAILSVTMSSIICANSAVTQAQPDPFYMAS---QLNLPRPCSDYS 615

+ GF Q+++I + +S I+C N +T Q + F + + + + PC

Sbjct 632 YERAHPITGFTERQLRSIRRMRLSKILCENFNITPLQKNVFLLPNSKVKSSHLVPCHSLP 691

Query 616 EMDVEPW 622

++D+ W

Sbjct 692 DLDIRAW 698

>[XP_009043737.1](https://www.ncbi.nlm.nih.gov/protein/XP_009043737.1?report=genbank&log$=protalign&blast_rank=34&RID=0) hypothetical protein LOTGIDRAFT_152002 [Lottia gigantea]

[ESP05192.1](https://www.ncbi.nlm.nih.gov/protein/ESP05192.1?report=genbank&log$=protalign&blast_rank=34&RID=0) hypothetical protein LOTGIDRAFT_152002 [Lottia gigantea]

Length=616

Score = 305 bits (780), Expect = 4e-91, Method: Compositional matrix adjust.

Identities = 196/597 (33%), Positives = 311/597 (52%), Gaps = 41/597 (7%)

Query 32 STGSLDFVSEADLEHCSRLTYDQLRYRQIDGRCN---HPRNYGSTGRPVKRYLRPHYQDK 88

+ G F+ +H + ++R DG CN HP +G G R + HY D

Sbjct 48 ANGETPFLGSLTSKHTCSEEQRKSKFRSADGSCNNLEHPA-WGQAGASYLRMISDHYDDD 106

Query 89 FGENLPRVYSVTGQLLPSPRMVSWKLHPDQTAHDNN-TMLVMQMGQFIDHDITRAPELSG 147

E++PR S G LLPS R VS ++ ++ T + MQ GQF+DHD+ P G

Sbjct 107 --ESVPRQTSELGSLLPSARTVSQEVFNSESHPSARLTQMAMQWGQFLDHDLASTPAEIG 164

Query 148 RNASIKCCGVPPKERLPDCFPIDIPPGDPVF-EDCMEFFRSSPAVDNDGNIIY-PREQIN 205

IK + +E DCFPI I GD F DCM F RS +G+ Y R+Q N

Sbjct 165 ----IKWHFMHLRE---DCFPIYIEEGDSYFTSDCMGFARSQRY---EGHTTYGKRQQFN 214

Query 206 ALTSFIDGSAVYGSDLDTYTWIRSENGTGVFLNTHLVHGRERLPSHPHLGPESCVSSNTA 265

+TSFIDGS VYGS + +R+ G+ T + G LP+ P +C + +

Sbjct 215 TITSFIDGSMVYGSSEEEMLMLRA-GEDGLMSTTDVPGGESFLPNDS--SPGACKNQDDV 271

Query 266 ESYCQLAGDMRVNEQPGLGSIHLLFHLHHNHIVRLLVAGILKKRGQPSSPERIAKFIQES 325

+C LAGD RVN PGL ++H LF HN I L S + + K + ES

Sbjct 272 GIFCFLAGDARVNVIPGLQAMHTLFVRFHNRIATELKT--------ISEVKNLFK-MTES 322

Query 326 SSALKEQIFQEVRKMLGAIIQKLTYCDWLPMILGPYLIDKFQLGCTRRSRYNSDLDPRVA 385

+S E ++Q RK++ A+IQ++TY D++P + G + +F+L + Y ++P++

Sbjct 323 ASNNDEFLYQTARKIVAAVIQRITYKDYIPSVFGREFVHQFKLD--QPYEYKPSVNPQII 380

Query 386 NSFLSAALRFGHTLIPNVYNFGDKRIHLKDTFNIPDASIRYYDNIIQCLIKEGSEEAYDR 445

N F +AA R+GH+LI + K + L + + ++ + +I LI EG E D+

Sbjct 381 NEFTTAAFRYGHSLITDSMKLNGKDVPLHELYFNTVPVLKNFSQVITNLI-EGRSENSDK 439

Query 446 YVSSAVSEHLFESTRGHKHALDLIAVNIQRGRDHGIPAYHYWRQYYRLRRIISLDEFGEA 505

Y S +++ LF +++G +LD++++NIQRGRDHG+P++ +R+ +L + ++FG+

Sbjct 440 YFSKEMTDKLFLTSKG---SLDIVSLNIQRGRDHGLPSFAEYRKICKLPPVKRFEDFGDC 496

Query 506 GIAMKKAYRDIRDVDLFPGGLLEPSMPGGVVGETFGHILANQFADLKFGDTYFFLHQQAP 565

G A++ Y+ DVDL+ G + E G VG TF I+ Q LK GD +F+

Sbjct 497 GRALRNVYKSPMDVDLYAGAICEKPHGGNEVGPTFACIIGTQLYTLKNGDRFFYTTLNHN 556

Query 566 QGFRAAQIKAILSVTMSSIICANSAVTQAQPDPFYMASQLNLPRPCSDYSEMDVEPW 622

GF A Q++ I +T+SS+ C + + Q + F+ ++++N R C++++ PW

Sbjct 557 LGFTAKQLQFINKITLSSLFCEFGEIGEVQSNVFFPSNRINRVRKCNNFA----VPW 609

>[XP_021373766.1](https://www.ncbi.nlm.nih.gov/protein/XP_021373766.1?report=genbank&log$=protalign&blast_rank=35&RID=0) chorion peroxidase-like [Mizuhopecten yessoensis]

Length=701

Score = 306 bits (783), Expect = 8e-91, Method: Compositional matrix adjust.

Identities = 199/590 (34%), Positives = 294/590 (50%), Gaps = 64/590 (11%)

Query 57 YRQIDGRCNHPRN--YGSTGRPVKRYLRPHYQDKFGENLPRVYSVTGQL-LPSPRMVSWK 113

YR +G+CN+ +N +GS G +RYL Y D G LPR S + LPS R+VS

Sbjct 148 YRTSNGQCNNLQNPDWGSAGISQRRYLEAKYSDGIG--LPRNMSSRDSIALPSARVVSNV 205

Query 114 LHPDQTAHDNNT---MLVMQMGQFIDHDITRAPELSGRNAS-IKCCGVPPKERLPDCFPI 169

L + ++ ++ ++VM GQF+DHDIT P +G S I CC R +CFPI

Sbjct 206 LFVNTSSQRTDSEMSLMVMAWGQFLDHDITLTPTAAGEEGSQIDCCDTTGSAR-DECFPI 264

Query 170 DIPPGDPVFE-DCMEFFRSSPAVDNDGNIIYPREQINALTSFIDGSAVYGSDLDTYTWIR 228

IP D F +CM F RSS AVD+ + R+Q NA+TSF+DGS VYGS T +R

Sbjct 265 TIPTNDQHFRTNCMSFVRSSAAVDSCDPAM--RQQTNAITSFVDGSNVYGSSAAETTNLR 322

Query 229 SENGTGVFLNTHLVHGRERLPSHPHLGPESCVSSNTAESYCQLAGDMRVNEQPGLGSIHL 288

F L + LP P +SC+ S T + +C GD+R N P LG+ H+

Sbjct 323 Q------FRKGKLRSSQGNLP--PAGSEDSCIVSTTGD-FCIETGDVRANVIPHLGANHV 373

Query 289 LFHLHHNHIVRLLVAGILKKRGQPSSPERIAKFIQESSSALKEQIFQEVRKMLGAIIQKL 348

LF HN I L L + E+ FQE RK++ A++Q++

Sbjct 374 LFFREHNRIANEL--STLNPQWN------------------DEKTFQETRKIVSALLQQI 413

Query 349 TYCDWLPMILGPYLIDKFQLGCTRRSRYNSDLDPRVANSFLSAALRFGHTLIPNVYNFGD 408

TY +WLP IL P ++ + L + R Y++ +DP + NSF AA+R+GH+L+ F

Sbjct 414 TYYEWLPSILAPEFLEIYNLKRSNRDPYSASVDPTIKNSFAVAAMRYGHSLVSGFQAF-- 471

Query 409 KRIH---------LKDTFNIPDASIRYYDNIIQCLIK---EGSEEAYDRYVSSAVSEHLF 456

+H +++TF P + N + L + DR + + + LF

Sbjct 472 -LLHDYMTYEVKPIEETFFKPSMVVGNSGNDVPMLARWVCANESMKRDRILERGIRDLLF 530

Query 457 ESTRGHKHALDLIAVNIQRGRDHGIPAYHYWRQYYRLRRIISLDEFGEAGI----AMKKA 512

+ G H+ DL A+N+QRGRDHG+P+Y+ WR++ L R + + E ++

Sbjct 531 LDSEG--HSFDLGALNVQRGRDHGVPSYNDWREWAGLPRATAFSQLSEHSKREIRLLQNV 588

Query 513 YRDIRDVDLFPGGLLEPSMPGGVVGETFGHILANQFADLKFGDTYFFLHQQAPQGFRAAQ 572

Y + D+DLF GG+ E + GG +G F HI A QF++LK GD +F+ A +GF Q

Sbjct 589 YNHVDDIDLFAGGISERDVSGGHLGRVFSHIFARQFSELKAGDRFFYERPTA-EGFTKDQ 647

Query 573 IKAILSVTMSSIICANSAVTQAQPDPFYMASQLNLPRPCSDYSEMDVEPW 622

+ I V +S ++C N + + F M + C MD+ W

Sbjct 648 LAEIRKVKLSKVMCENFGIDLIPENVFKMVGIARTKKSCESLPGMDLSKW 697

>[XP_019638393.1](https://www.ncbi.nlm.nih.gov/protein/XP_019638393.1?report=genbank&log$=protalign&blast_rank=36&RID=0) PREDICTED: peroxidasin homolog [Branchiostoma belcheri]

Length=693

Score = 306 bits (783), Expect = 8e-91, Method: Compositional matrix adjust.

Identities = 209/608 (34%), Positives = 311/608 (51%), Gaps = 67/608 (11%)

Query 47 CSRLTYDQLRYRQIDGRCNHPRN--YGSTGRPVKRYLRPHYQDKFGENLPRVYSVTGQLL 104

C RL + +R +GRCN+ N +GST +P+KR L P Y D G PR+ G L

Sbjct 114 CPRLRPPRAEFRSANGRCNNRDNPLWGSTEQPLKRLLEPDYND--GLMRPRITGHGGAPL 171

Query 105 PSPRMVSWKLHPD-QTAHDNNTMLVMQMGQFIDHDITRAPELSGRNASIKCCGVPPKERL 163

PS R+VS +H D + + NT +VMQ GQF+DHD T P + + C

Sbjct 172 PSARLVSTVMHEDLRKSSPVNTHMVMQFGQFLDHDFTLTPSVQEEGITCTC-----DSED 226

Query 164 PDCFPIDIPPGDPVFED--CMEFFRSSPAVDNDGNIIYPREQINALTSFIDGSAVYGSDL 221

CF IDIP DP F C++F RS A N+G + R+Q+N +T+F+D S VYGS

Sbjct 227 EHCFNIDIPSDDPDFAGTPCLQFARSK-ASPNEGCHMGRRQQLNQITAFVDASNVYGSSD 285

Query 222 DTYTWIRSENGTGVFL----NTHLVHGRERLPSHPHLGPESCVSSNTAESYCQLAGDMRV 277

+ +R +G L N + +E LP E C AE+ C AGD+RV

Sbjct 286 EEIEALREHSGGRGLLKSRPNPADANKKELLPGAMAEAFE-CAEFTGAET-CSQAGDVRV 343

Query 278 NEQPGLGSIHLLFHLHHNHIVRLLVAGILKKRGQPSSPERIAKFIQESSSALKEQIFQEV 337

NEQPGL S+H +F HN I R + Q + +++F E

Sbjct 344 NEQPGLTSMHTVFLREHNRIAR--------------------RLSQLNPRWDDDRVFFET 383

Query 338 RKMLGAIIQKLTYCDWLPMILGPYLIDKFQLGCTRR---SRYNSDLDPRVANSFLSAALR 394

R ++GA++QK+TY ++LP ++GP + KF L S Y++ ++P ++N F +AA R

Sbjct 384 RHIVGALMQKITYGEFLPRVVGPDAMTKFHLTLATNGYFSGYDASVNPTISNVFATAAFR 443

Query 395 FGHTLIPNV-------YNFGDK-RIHLKDTFNIPDASIRYY----DNIIQCLIKEGSEEA 442

FGH+L+ N+ +N G + L F P D+I++ + + ++

Sbjct 444 FGHSLVQNLLLRFDPDFNQGSRCPFQLALGFFNPTQIFNNNEGGPDSILRGMTTQPHQD- 502

Query 443 YDRYVSSAVSEHLFESTRGHKHALDLIAVNIQRGRDHGIPAYHYWRQYYRLRRIISLDEF 502

+DR++ S +++HLF G LDL A+NIQRGRDHG+P Y+ WR+ L + S DE

Sbjct 503 FDRFMVSGLTKHLFADPPG-SLGLDLAALNIQRGRDHGLPGYNAWRERCGLVKANSFDEL 561

Query 503 ------GEAGIAMKKAYRDIRDVDLFPGGLLEPSMPGGVVGETFGHILANQFADLKFGDT 556

++ Y + D+DLF G L E S+PGGVVG TF ++ QF DL+ GD

Sbjct 562 VFEIPDPYTRKRLQHVYSHVDDIDLFAGALAEESVPGGVVGPTFACLIGLQFQDLRKGDR 621

Query 557 YFFLHQQAPQGFRAAQIKAILSVTMSSIICANS-AVTQAQPDPFYMASQLNLPR-PCSDY 614

++F ++ F AQ+ I +++ I+C N+ T QPD F + +Q R CS

Sbjct 622 FWFENRVQ---FTGAQLAEIRQTSLARILCDNTDGTTHMQPDVFRLPTQPGNERVACSSL 678

Query 615 SEMDVEPW 622

++D+ W

Sbjct 679 PQVDLTKW 686

>[XP_023706652.1](https://www.ncbi.nlm.nih.gov/protein/XP_023706652.1?report=genbank&log$=protalign&blast_rank=37&RID=0) peroxidase [Cryptotermes secundus]

[PNF34740.1](https://www.ncbi.nlm.nih.gov/protein/PNF34740.1?report=genbank&log$=protalign&blast_rank=37&RID=0) Chorion peroxidase [Cryptotermes secundus]

Length=886

Score = 310 bits (794), Expect = 1e-90, Method: Compositional matrix adjust.

Identities = 204/599 (34%), Positives = 320/599 (53%), Gaps = 67/599 (11%)

Query 56 RYRQIDGRCNHPRN--YGSTGRPVKRYLRPHYQDKFGENLPRVYSVTGQLLPSPRMVSWK 113

+YR DG CN+ +N +G G ++R L P Y D G N PRV + TG+ LPSPR++S +

Sbjct 317 KYRSADGSCNNLQNERWGRAGTALQRILPPKYGD--GVNSPRV-AATGRELPSPRLISTQ 373

Query 114 LHPDQTA-HDNNTMLVMQMGQFIDHDITRAPELSGRNAS-IKCCG----VPPKERLPDCF 167

++ A +N T+L+MQ GQF+DHD+T P G++ S + CC + P+ R PDCF

Sbjct 374 FATERDAPSENYTLLLMQWGQFLDHDLTHTPISRGQSGSGLSCCRDGDIIKPELRHPDCF 433

Query 168 PIDIPPGDPVF----EDCMEFFRSSPAVDNDGNIIYPREQINALTSFIDGSAVYGSDLDT 223

PI +P D +F + CMEF RS PA + N PREQ+N +T + DGS +YGS L

Sbjct 434 PITLPRNDHIFARLGQRCMEFVRSLPAPRPECNF-GPREQMNQITGYQDGSNIYGSSLSA 492

Query 224 YTWIRSENGTGVFLNTHLVHGRERLPSHPHLGPESCVSSNTAESYCQLAGDMRVNEQPGL 283

+R G G L V GR+ LP++ + + C AGD RVNEQ L

Sbjct 493 QRELRE--GRGGRLAIQNVGGRQLLPAN-----RGECTDDAERLACFKAGDTRVNEQVEL 545

Query 284 GSIHLLFHLHHNHIVRLLVAGILKKRGQPSSPERIAKFIQESSSALKEQIFQEVRKMLGA 343

+H L+ HN + A+ + + S E +FQE R+++ A

Sbjct 546 VVMHTLWMREHNRVA--------------------AELAKLNPSWGDEALFQEARRIVVA 585

Query 344 IIQKLTYCDWLPMILGPYLIDKFQL-----GCTRRSRYNSDLDPRVANSFLSAALRFGHT 398

+Q +TY ++LP+ILG ++KF+L G TR Y+ L+P + N F +AA RFGHT

Sbjct 586 EMQHITYNEFLPLILGRDYMEKFELSPRDSGATRL--YDESLNPSITNVFATAAFRFGHT 643

Query 399 LIPN----VYNFGDKRIHLKDTFNIPDASIRYYDNIIQCLIK---EGSEEAYDRYVSSAV 451

LI + FG+ R +L+ + + + Y + ++ L++ S + +DR+ S+ +

Sbjct 644 LIQSRILGFSRFGNVRENLELSQHQFAPFVLYENGVLDNLVRGLSTQSSQRFDRFFSNQL 703

Query 452 SEHLFESTRGHKHALDLIAVNIQRGRDHGIPAYHYWRQYYRLRRIISLDEF-----GEAG 506

++HLF+ LDL+A+N QRGRDHG+P Y+ WR+ L R S D ++

Sbjct 704 TDHLFQGD--LDFGLDLVALNTQRGRDHGLPPYNDWREVCGLPRARSWDSLLDVMDSQSV 761

Query 507 IAMKKAYRDIRDVDLFPGGLLEPSMPGGVVGETFGHILANQFADLKFGDTYFFLHQQAPQ 566

A+++ Y + ++DLF + E +PG ++G+TF ++ +QFA L+ GD +++ P

Sbjct 762 AALQELYSSVDEIDLFVAAVAEKPLPGALLGQTFVCLVGDQFARLRRGDRFYYEEGGQPS 821

Query 567 GFRAAQIKAILSVTMSSIICANS-AVTQAQPDPFYMASQLNLPRPCSDYS--EMDVEPW 622

F Q+ + +++ I+C NS + QP F+ AS +N C+ S +D+ W

Sbjct 822 SFSPQQLVQLRKASLARILCDNSDDIALMQPLAFFRASFVNQRVACASESIPRVDLSAW 880

>[XP_022246593.1](https://www.ncbi.nlm.nih.gov/protein/XP_022246593.1?report=genbank&log$=protalign&blast_rank=38&RID=0) peroxidase-like isoform X2 [Limulus polyphemus]

Length=740

Score = 306 bits (784), Expect = 2e-90, Method: Compositional matrix adjust.

Identities = 213/603 (35%), Positives = 319/603 (53%), Gaps = 73/603 (12%)

Query 56 RYRQIDGRCNHPR--NYGSTGRPVKRYLRPHYQDKFGENLPRVYSVTGQLLPSPRMVSWK 113

++R IDG CN+ + ++GS + R L P Y D G + PR S +G LP+PR+VS

Sbjct 175 KFRTIDGSCNNVKHHDWGSALNCMNRILPPDYAD--GVSNPRKAS-SGNELPNPRLVSTT 231

Query 114 LHPDQTAHDNN-TMLVMQMGQFIDHDITRAPELS--------GRNASIKCCGVPPKERLP 164

+H ++ + T L+MQ G F+ HDIT P S I CC P

Sbjct 232 IHFEKDVPAGDFTHLLMQWGHFLSHDITFTPSRSFPRGDVALALRKIIDCCA-PEDRHNS 290

Query 165 DCFPIDIPPGDPVFED----CMEFFRSSPAVDNDGNIIYPREQINALTSFIDGSAVYGSD 220

CFP IPP DP F CM+F RS+ + + REQIN +TSFIDGS +YGS

Sbjct 291 QCFPFAIPPDDPFFTHYNIRCMDFRRSARCLRSGPG---QREQINQITSFIDGSQIYGSF 347

Query 221 LDTYTWIRS-ENGTGVFLNTHL-VHGRERLPSHPHLGPESCVSSNTAESYCQLAGDMRVN 278

L+ IR+ +NG L T G E LP P+ + C S + C +AGD+RVN

Sbjct 348 LNISLAIRTFQNG---LLKTQTDNQGGEILPGSPNAESDFC-SDVANNNICFMAGDIRVN 403

Query 279 EQPGLGSIHLLFHLHHNHIVRLLVAGILKKRGQPSSPERIAKFIQESSSALKEQIFQEVR 338

+QPGL S+H+++ HN + R L K+ PS + E ++QE R

Sbjct 404 QQPGLTSMHIIWLRQHNRLARQL------KQLNPSWDD--------------ETLYQEAR 443

Query 339 KMLGAIIQKLTYCDWLPMILGPYLIDKFQLG--CTRRSRYNSDLDPRVANSFLSAALRFG 396

+++GA +Q +TY ++LP++LGP F L + + Y + DP + N F +AA RFG

Sbjct 444 RIVGAQLQMITYNEFLPVVLGPLFYLTFHLKPLSSGYTSYLKEKDPTILNEFSTAAFRFG 503

Query 397 HTLIPN----VYNFGDK-RIHLKDTFNIP-DASIRYYDNIIQCLIKEGSEEAYDRYVSSA 450

HTLI N +Y+ + + L+D F P + D+I++ L+K+ +E +D + S++

Sbjct 504 HTLIQNNFSQIYSSNNSSNMELQDNFFFPFELYYGQLDSILRGLMKDKGQE-FDTFFSNS 562

Query 451 VSEHLFESTRGHKHALDLIAVNIQRGRDHGIPAYHYWRQYYRLRRI-----ISLDEFGEA 505

V+ HLF+ RG +H LD+ A+NIQRGRDHG+ +Y + +Y L+ + + L +A

Sbjct 563 VTNHLFKR-RGEEHGLDIAALNIQRGRDHGLRSYVDYLEYCFLKHLTKFHHLQLTMTKDA 621

Query 506 GIAMKKAYRDIRDVDLFPGGLLEPSMPGGVVGETFGHILANQFADLKFGDTYFFLHQQAP 565

+ Y + D+DLF GG+ E + G+VG TFG I+ QF LKFGD Y+F H +

Sbjct 622 QNHFENLYEKVEDIDLFSGGVNERPLNKGIVGPTFGCIIGVQFGLLKFGDRYYFEHGKQS 681

Query 566 QGFRAAQIKAILSVTMSSIICANSAVTQA------QPDPFYMASQLNLPRPCSDYSEMDV 619

F Q++ I T++ I+C NS Q+ +P+ F +N C+D E+++

Sbjct 682 GSFTPDQLREIRKTTLARILCDNSDGFQSISRFPFRPENFERNEVIN----CNDLPEINL 737

Query 620 EPW 622

E W

Sbjct 738 ELW 740

>[XP_019633295.1](https://www.ncbi.nlm.nih.gov/protein/XP_019633295.1?report=genbank&log$=protalign&blast_rank=39&RID=0) PREDICTED: peroxidasin homolog [Branchiostoma belcheri]

Length=629

Score = 303 bits (776), Expect = 2e-90, Method: Compositional matrix adjust.

Identities = 211/603 (35%), Positives = 302/603 (50%), Gaps = 69/603 (11%)

Query 57 YRQIDGRCNHPRN--YGSTGRPVKRYLRPHYQDKFGENLPRVYSVTGQLLPSPRMVSWKL 114

+R DGRCN+ R +GST + +KR L P Y D F PR LP+ R VS +

Sbjct 61 FRSADGRCNNQRRPLWGSTEQCLKRLLPPEYDDGFMS--PRTIGRNRARLPTARRVSTVM 118

Query 115 HPD-QTAHDNNTMLVMQMGQFIDHDITRAPELSGRNASIKCCGVPPKERLPDCFPIDIPP 173

H D + + NT +VMQ GQF+DHD T P C + + CF I+IP

Sbjct 119 HEDLRKSSQVNTHMVMQFGQFLDHDFTLTPSFQEEGLDCDCDSMDER-----CFNINIPS 173

Query 174 GDPVF--EDCMEFFRSSPAVDNDGNIIYPREQINALTSFIDGSAVYGSDLDTYTWIRSE- 230

D F C+ F RS + N+G + R+Q+N +T+F+D S VYGS +R

Sbjct 174 DDRDFAGRRCLGFARSR-SCPNEGCRMGRRQQLNQITAFVDASNVYGSSDKEIQELRGRS 232

Query 231 --NGTGVFLNTHL----VHGRERLPSHPHLGPESCVSSNTAESYCQLAGDMRVNEQPGLG 284

+GT L + L +E LP G E T C AGD+RVNEQPGL

Sbjct 233 QRDGTRGQLKSRLNPAKADKKELLPGAFTEGFE--CDEFTGSETCSQAGDVRVNEQPGLT 290

Query 285 SIHLLFHLHHNHIVRLLVAGILKKRGQPSSPERIAKFIQESSSALKEQIFQEVRKMLGAI 344

S+H +F HN I R L Q + +++F E RK++GA+

Sbjct 291 SMHTVFLREHNRIARRLS--------------------QINPRWSDDRVFFETRKIVGAL 330

Query 345 IQKLTYCDWLPMILGPYLIDKFQLGCTRRS---RYNSDLDPRVANSFLSAALRFGHTLIP 401

+QK+TY ++LP +LGP + ++L R Y+S ++P ++N F +AA RFGH+L+

Sbjct 331 MQKITYGEFLPRVLGPTTMTTYRLRLLRSGFYGGYDSRVNPTISNVFATAAFRFGHSLVQ 390

Query 402 NVY-----NFGDKR---IHLKDTFNIPDASIRYY----DNIIQCLIKEGSEEAYDRYVSS 449

N+ +F + I L +F P D+I++ L + ++ +DR++ S

Sbjct 391 NLLLRYSSDFNEASACPIRLAFSFFNPSHIFNNDQGGPDSILRGLTAQPHQD-FDRFMVS 449

Query 450 AVSEHLFESTRGHKHALDLIAVNIQRGRDHGIPAYHYWRQYYRLRRIISLDEFGE----- 504

+++ LF G LDL A+NIQRGRDHG+P Y+ WR LRR S +

Sbjct 450 GLTKDLFADPPGSGRGLDLAALNIQRGRDHGLPGYNAWRVKVGLRRARSFADLAREIPDV 509

Query 505 -AGIAMKKAYRDIRDVDLFPGGLLEPSMPGGVVGETFGHILANQFADLKFGDTYFFLHQQ 563

++ Y + D+DLF GGL E SMPGGVVG TF +LA QF DL+ GD +F ++

Sbjct 510 NTRQKLESVYSHVDDIDLFVGGLAEESMPGGVVGPTFACLLAMQFQDLRKGDRLWFENRG 569

Query 564 APQGFRAAQIKAILSVTMSSIICANS-AVTQAQPDPFYMASQLNLPR-PCSDYSEMDVEP 621

F AAQI I +++ I+C N+ T QPD F + +Q R CS S MD+

Sbjct 570 Q---FTAAQIAEIRKTSLARILCDNTDGTTHMQPDVFRLPTQPGNERVRCSSLSRMDLTK 626

Query 622 WLI 624

W +

Sbjct 627 WRV 629

>[XP_022246592.1](https://www.ncbi.nlm.nih.gov/protein/XP_022246592.1?report=genbank&log$=protalign&blast_rank=40&RID=0) peroxidase-like isoform X1 [Limulus polyphemus]

Length=741

Score = 306 bits (783), Expect = 2e-90, Method: Compositional matrix adjust.

Identities = 213/603 (35%), Positives = 319/603 (53%), Gaps = 73/603 (12%)

Query 56 RYRQIDGRCNHPR--NYGSTGRPVKRYLRPHYQDKFGENLPRVYSVTGQLLPSPRMVSWK 113

++R IDG CN+ + ++GS + R L P Y D G + PR S +G LP+PR+VS

Sbjct 175 KFRTIDGSCNNVKHHDWGSALNCMNRILPPDYAD--GVSNPRKAS-SGNELPNPRLVSTT 231

Query 114 LHPDQTAHDNN-TMLVMQMGQFIDHDITRAPELS--------GRNASIKCCGVPPKERLP 164

+H ++ + T L+MQ G F+ HDIT P S I CC P

Sbjct 232 IHFEKDVPAGDFTHLLMQWGHFLSHDITFTPSRSFPRGDVALALRKIIDCCA-PEDRHNS 290

Query 165 DCFPIDIPPGDPVFED----CMEFFRSSPAVDNDGNIIYPREQINALTSFIDGSAVYGSD 220

CFP IPP DP F CM+F RS+ + + REQIN +TSFIDGS +YGS

Sbjct 291 QCFPFAIPPDDPFFTHYNIRCMDFRRSARCLRSGPG---QREQINQITSFIDGSQIYGSF 347

Query 221 LDTYTWIRS-ENGTGVFLNTHL-VHGRERLPSHPHLGPESCVSSNTAESYCQLAGDMRVN 278

L+ IR+ +NG L T G E LP P+ + C S + C +AGD+RVN

Sbjct 348 LNISLAIRTFQNG---LLKTQTDNQGGEILPGSPNAESDFC-SDVANNNICFMAGDIRVN 403

Query 279 EQPGLGSIHLLFHLHHNHIVRLLVAGILKKRGQPSSPERIAKFIQESSSALKEQIFQEVR 338

+QPGL S+H+++ HN + R L K+ PS + E ++QE R

Sbjct 404 QQPGLTSMHIIWLRQHNRLARQL------KQLNPSWDD--------------ETLYQEAR 443

Query 339 KMLGAIIQKLTYCDWLPMILGPYLIDKFQLG--CTRRSRYNSDLDPRVANSFLSAALRFG 396

+++GA +Q +TY ++LP++LGP F L + + Y + DP + N F +AA RFG

Sbjct 444 RIVGAQLQMITYNEFLPVVLGPLFYLTFHLKPLSSGYTSYLKEKDPTILNEFSTAAFRFG 503

Query 397 HTLIPN----VYNFGDK-RIHLKDTFNIP-DASIRYYDNIIQCLIKEGSEEAYDRYVSSA 450

HTLI N +Y+ + + L+D F P + D+I++ L+K+ +E +D + S++

Sbjct 504 HTLIQNNFSQIYSSNNSSNMELQDNFFFPFELYYGQLDSILRGLMKDKGQE-FDTFFSNS 562

Query 451 VSEHLFESTRGHKHALDLIAVNIQRGRDHGIPAYHYWRQYYRLRRI-----ISLDEFGEA 505

V+ HLF+ RG +H LD+ A+NIQRGRDHG+ +Y + +Y L+ + + L +A

Sbjct 563 VTNHLFKR-RGEEHGLDIAALNIQRGRDHGLRSYVDYLEYCFLKHLTKFHHLQLTMTKDA 621

Query 506 GIAMKKAYRDIRDVDLFPGGLLEPSMPGGVVGETFGHILANQFADLKFGDTYFFLHQQAP 565

+ Y + D+DLF GG+ E + G+VG TFG I+ QF LKFGD Y+F H +

Sbjct 622 QNHFENLYEKVEDIDLFSGGVNERPLNKGIVGPTFGCIIGVQFGLLKFGDRYYFEHGKQS 681

Query 566 QGFRAAQIKAILSVTMSSIICANSAVTQA------QPDPFYMASQLNLPRPCSDYSEMDV 619

F Q++ I T++ I+C NS Q+ +P+ F +N C+D E+++

Sbjct 682 GSFTPDQLREIRKTTLARILCDNSDGFQSISRFPFRPENFERNEVIN----CNDLPEINL 737

Query 620 EPW 622

E W

Sbjct 738 ELW 740

>[XP_021373773.1](https://www.ncbi.nlm.nih.gov/protein/XP_021373773.1?report=genbank&log$=protalign&blast_rank=41&RID=0) myeloperoxidase-like [Mizuhopecten yessoensis]

Length=876

Score = 308 bits (789), Expect = 5e-90, Method: Compositional matrix adjust.

Identities = 199/589 (34%), Positives = 300/589 (51%), Gaps = 60/589 (10%)

Query 57 YRQIDGRCNHPRN--YGSTGRPVKRYLRPHYQDKFGENLPR-VYSVTGQLLPSPRMVSWK 113

YR +G+CN+ +N +GS G +RYL Y D G LPR + S LPS R+VS

Sbjct 146 YRTSNGQCNNLQNPDWGSAGISQRRYLDAKYSDGIG--LPRNISSRDSTALPSARVVSNV 203

Query 114 LHPDQTAHDNNT---MLVMQMGQFIDHDITRAPELSGRNAS-IKCCGVPPKERLPDCFPI 169

L + ++ ++ ++VM GQF+DHDIT P +G S I CC R +CFPI

Sbjct 204 LFVNTSSQRTDSEMSLMVMAWGQFLDHDITLTPTAAGEEGSQIDCCDTTGTGR-DECFPI 262

Query 170 DIPPGDPVFE-DCMEFFRSSPAVDNDGNIIYPREQINALTSFIDGSAVYGSDLDTYTWIR 228

IP D F +CM F RSS AVD+ + R+Q NA+TSF+DGS VYGS T +R

Sbjct 263 TIPTNDQHFRTNCMSFVRSSAAVDSCDPAM--RQQTNAITSFVDGSNVYGSSAAETTNLR 320

Query 229 SENGTGVFLNTHLVHGRERLPSHPHLGPESCVSSNTAESYCQLAGDMRVNEQPGLGSIHL 288

F L + LP P +SC+ S T + +C GD+R N P LG+ H+

Sbjct 321 Q------FRKGKLKSSQGNLP--PAGSEDSCIVSKTGD-FCIETGDVRANVVPHLGANHV 371

Query 289 LFHLHHNHIVRLLVAGILKKRGQPSSPERIAKFIQESSSALK-EQIFQEVRKMLGAIIQK 347

LF HN RIAK + + E+ F+E RK++ A++Q+

Sbjct 372 LFFREHN---------------------RIAKELSTLNPQWNDEKTFEETRKIISALLQQ 410

Query 348 LTYCDWLPMILGPYLIDKFQLGCTRRSRYNSDLDPRVANSFLSAALRFGHTLIPNV---- 403

+TY +WLP IL P ++ + L + R Y++ +DP + NSF AA+R+GH+L+ V

Sbjct 411 ITYYEWLPSILAPEFLEIYNLKRSNRDPYSASVDPTIKNSFAVAAMRYGHSLVSGVQAFL 470

Query 404 -YNFGDKRIH-LKDTFNIPDASIRYYDNIIQCLIK---EGSEEAYDRYVSSAVSEHLFES 458

++F + +++TF P + N + L + DR + + + LF

Sbjct 471 LHDFMTYEVKPIEETFFKPSMVVGNRGNDVPMLARWVCANESMKRDRILERGIRDLLFLD 530

Query 459 TRGHKHALDLIAVNIQRGRDHGIPAYHYWRQYYRLRRIISLDEFGEAGIA----MKKAYR 514

+ G H+ DL A+N+QRGRDHG+P+Y+ WR++ L R + + E ++K Y

Sbjct 531 SEG--HSFDLGALNVQRGRDHGVPSYNDWREWAGLPRATAFSQLSEHTKREIRLLQKVYN 588

Query 515 DIRDVDLFPGGLLEPSMPGGVVGETFGHILANQFADLKFGDTYFFLHQQAPQGFRAAQIK 574

+ D+DLF GG+ E + GG +G F HI A QF++LK GD +F+ A +GF Q+

Sbjct 589 HVDDIDLFAGGISERDVSGGHLGRVFSHIFARQFSELKAGDRFFYERPTA-EGFTKDQLA 647

Query 575 AILSVTMSSIICANSAVTQAQPDPFYMASQLNLPRPCSDYSEMDVEPWL 623

I V +S ++C N + + F + + C MD+ W+

Sbjct 648 EIRKVKLSKVMCENFGLDLIPENVFKLVGIARTKKSCESLPGMDLSKWI 696

>[XP_034310072.1](https://www.ncbi.nlm.nih.gov/protein/XP_034310072.1?report=genbank&log$=protalign&blast_rank=42&RID=0) eosinophil peroxidase [Crassostrea gigas]

Length=694

Score = 303 bits (776), Expect = 9e-90, Method: Compositional matrix adjust.

Identities = 213/593 (36%), Positives = 300/593 (51%), Gaps = 63/593 (11%)

Query 57 YRQIDGRCN---HPRNYGSTGRPVKRYLRPHYQDKFGENLPRVYSVTGQLLPSPRMVSWK 113

YR IDGRCN HP +G+ P RY Y D G + PR G LPSPR +S K

Sbjct 135 YRSIDGRCNNLIHPL-WGAANTPQPRYSPAEYDD--GISTPRSRGKDGSPLPSPRQISNK 191

Query 114 LH--PDQTAHDNN--TMLVMQMGQFIDHDITRAPELSGR-NASIKCCGVPPKERLPDCFP 168

L P + ++ T++VM GQFIDHD+ P + G + I CCG + R P CFP

Sbjct 192 LFRAPRECTETDHARTLMVMAWGQFIDHDLAHTPTMKGDGDVPITCCGENVQNR-PQCFP 250

Query 169 IDIPPGDPVFED-CMEFFRSSPAVDNDGNIIYPREQINALTSFIDGSAVYGSDLDTYTWI 227

I IP DP F D CMEF RS+P+ DG + PREQIN +TSFIDG +VYG+ +

Sbjct 251 ISIPSDDPHFNDTCMEFVRSAPSPPGDGCQLGPREQINQITSFIDGGSVYGNSAKKMAEL 310

Query 228 RSENGTGVFLNTHLVHGRERLPSHPHLGPESCVSSNTAESYCQLAGDMRVNEQPGLGSIH 287

+++ + LP P + + +NT + +CQ AGD RVNE P LG H

Sbjct 311 KNK-----YTGQMRTSAGNLLP--PAVNGTCELPANTTD-FCQNAGDSRVNEVPFLGGNH 362

Query 288 LLFHLHHNHIVRLLVAGILKKRGQPSSPERIAKFIQESSSALKEQIFQEVRKMLGAIIQK 347

L+F HN IVR L + +Q S+LK ++QE RK++GA++Q+

Sbjct 363 LMFVREHNRIVREL------------------RKVQPRWSSLK--LYQEARKIIGALLQQ 402

Query 348 LTYCDWLPMILGPYLIDKFQLGCTR---RSRYNSDLDPRVANSFLSAALRFGHTLIP--N 402

+TY ++LP IL ++K +L + YN L+P N F +A RFGH+LIP

Sbjct 403 VTYREFLPSILRKQDLEKHKLKLRNWGFSNSYNCSLNPGTKNVFNAAVFRFGHSLIPLDL 462

Query 403 VYNFGDKRIHL-----KDTFNIPDASI----RYYDNIIQCLIKEGSEEAYDRYVSSAVSE 453

Y D HL + TF P I R ++ + ++ S + D + AV

Sbjct 463 AYLLYDFMSHLNSTPIESTFMNPHLLITKGGRRVSDLARFIVTSNSMK-LDNQLEGAVRN 521

Query 454 HLFESTRGHKHALDLIAVNIQRGRDHGIPAYHYWRQYYRLRRIISL----DEFGEAGIAM 509

LFE+ +G +DL A+N+ RGRDHG+P Y+ WR++ L S D E

Sbjct 522 RLFENKQG--KGMDLGALNLARGRDHGLPPYNAWRKWCGLPVATSFSNLPDISDEKKAIF 579

Query 510 KKAYRDIRDVDLFPGGLLEPSMPGGVVGETFGHILANQFADLKFGDTYFFLHQQAPQGFR 569

Y ++ D+D+F GG+ E + G VG F I+ NQF DLK GD Y++ + +GF

Sbjct 580 ADLYSNVGDIDVFAGGIAETPLDGAAVGPLFSCIIGNQFRDLKDGDRYWY-ENRGVEGFT 638

Query 570 AAQIKAILSVTMSSIICANSAVTQAQPDPFYMASQLNLPRPCSDYSEMDVEPW 622

Q++ I V ++ IIC N V Q D F++ S N + C ++ W

Sbjct 639 LGQLQQIRRVKLAKIICQNLGVDPIQRDVFHVPSPRNRWQRCRRLPGINFYYW 691

>[KAE8751717.1](https://www.ncbi.nlm.nih.gov/protein/KAE8751717.1?report=genbank&log$=protalign&blast_rank=43&RID=0) Chorion peroxidase-like-2 [Frankliniella occidentalis]

Length=832

Score = 306 bits (783), Expect = 1e-89, Method: Compositional matrix adjust.

Identities = 205/608 (34%), Positives = 319/608 (52%), Gaps = 78/608 (13%)

Query 56 RYRQIDGRCNHPRN--YGSTGRPVKRYLRPHYQDKFGENLPRVYSVTGQLLPSPRMVSWK 113

+YR D CN+ N +GS G ++R L P Y D G N PR + G LPS R+VS +

Sbjct 254 KYRTPDATCNNVVNSRWGSQGAALQRLLPPKYGD--GVNSPRAM-LNGAPLPSARLVSNR 310

Query 114 L-HPDQTAHDNNTMLVMQMGQFIDHDITRAPELSGRNA-SIKCCG----VPPKERLPDCF 167

L DN T+L+MQ GQF+DHD+T P G+N I CC P+ PDCF

Sbjct 311 LAQESDRPSDNVTLLLMQWGQFLDHDLTHTPISRGQNGVGISCCQDGQVSDPRVSHPDCF 370

Query 168 PIDIPPGD----PVFEDCMEFFRSSPAVDNDGNIIYPREQINALTSFIDGSAVYGSDLDT 223

PI +P D P E CMEF RS PA + N PREQ+N +T F+DGS VYGSD +

Sbjct 371 PIPVPRDDRFLAPFGERCMEFVRSLPAPRPECNF-GPREQMNQITGFLDGSNVYGSDANK 429

Query 224 YTWIRSENGTGVFLNTHLVHGRERLPSHPHLGPESCVSSNTAESYCQLAGDMRVNEQPGL 283

+R NG L V GR LP++P C +T+ + C ++GD RVNEQP L

Sbjct 430 LRQLRLFNGGR--LREQNVRGRSMLPANP----TEC--QDTSGAACFVSGDGRVNEQPDL 481

Query 284 GSIHLLFHLHHNHIVRLLVAGILKKRGQPSSPERIAKFIQESSSALKEQIFQEVRKMLGA 343

+H ++ HN + L Q PE E++FQE R+++ A

Sbjct 482 ALMHTVWLREHNRVASAL---------QALRPEWT-----------DEELFQEARRIVVA 521

Query 344 IIQKLTYCDWLPMILGPYLIDKFQL-----GCTRRSRYNSDLDPRVANSFLSAALRFGHT 398

+Q +TY ++LP++LG +D+ ++ G T + Y+ +L+P + N+F +AA RFGHT

Sbjct 522 EMQHITYNEFLPIVLGKPYMDRAEMSPKDSGYT--ALYDRELNPGITNAFATAAFRFGHT 579

Query 399 LIPNVYNFGD--------KRIHLKDTFNIPDASIRYY--------DNIIQCLIKEGSEEA 442

L+ + + D + +++ F + ++ + + D++++ L + S++

Sbjct 580 LLVSNLQYDDDYDDSGVGRFGNVRKNFALSKSAFKPFMLYEEEGLDDMLRGLTTQSSQK- 638

Query 443 YDRYVSSAVSEHLFESTRGHKHALDLIAVNIQRGRDHGIPAYHYWRQYYRLRRIISLDEF 502

+DR+ + ++ HLF++ LDL+A+N+QRGRDHG+P Y WRQ LRR S ++

Sbjct 639 FDRFFTKEITNHLFQND--LPFGLDLVALNLQRGRDHGLPGYPEWRQVCGLRRPRSWEDL 696

Query 503 G-----EAGIAMKKAYRDIRDVDLFPGGLLEPSMPGGVVGETFGHILANQFADLKFGDTY 557

+A ++ Y + +VDLF G+ E PG ++G TF I+ +QF L+ GD +

Sbjct 697 QGIMDPDAISVLQSLYPSVEEVDLFAAGVSERPAPGALLGPTFTCIVGDQFGRLRRGDRF 756

Query 558 FFLHQQAPQGFRAAQIKAILSVTMSSIICANS-AVTQAQPDPFYMASQLNLPRPCSDYS- 615

F+ P F+ Q++ I +++ ++C NS + QP F+ AS +N C+ +

Sbjct 757 FYEEGNQPSSFKPEQLQQIRRTSLARVLCDNSDNIALMQPLAFFHASFVNQRVACNSDAI 816

Query 616 -EMDVEPW 622

+D+ W

Sbjct 817 PRLDIRAW 824

>[XP_026286195.1](https://www.ncbi.nlm.nih.gov/protein/XP_026286195.1?report=genbank&log$=protalign&blast_rank=44&RID=0) peroxidase-like [Frankliniella occidentalis]

Length=937

Score = 308 bits (788), Expect = 2e-89, Method: Compositional matrix adjust.

Identities = 204/600 (34%), Positives = 317/600 (53%), Gaps = 70/600 (12%)

Query 56 RYRQIDGRCNHPRN--YGSTGRPVKRYLRPHYQDKFGENLPRVYSVTGQLLPSPRMVSWK 113

+YR D CN+ N +GS G ++R L P Y D G N PR + G LPS R+VS +

Sbjct 367 KYRTPDATCNNVVNSRWGSQGAALQRLLPPKYGD--GVNSPRAM-LNGAPLPSARLVSNR 423

Query 114 L-HPDQTAHDNNTMLVMQMGQFIDHDITRAPELSGRNA-SIKCCG----VPPKERLPDCF 167

L DN T+L+MQ GQF+DHD+T P G+N I CC P+ PDCF

Sbjct 424 LAQESDRPSDNVTLLLMQWGQFLDHDLTHTPISRGQNGVGISCCQDGQVSDPRVSHPDCF 483

Query 168 PIDIPPGD----PVFEDCMEFFRSSPAVDNDGNIIYPREQINALTSFIDGSAVYGSDLDT 223

PI +P D P E CMEF RS PA + N PREQ+N +T F+DGS VYGSD +

Sbjct 484 PIPVPRDDRFLAPFGERCMEFVRSLPAPRPECNF-GPREQMNQITGFLDGSNVYGSDANK 542

Query 224 YTWIRSENGTGVFLNTHLVHGRERLPSHPHLGPESCVSSNTAESYCQLAGDMRVNEQPGL 283

+R NG L V GR LP++P C +T+ + C ++GD RVNEQP L

Sbjct 543 LRQLRLFNGGR--LREQNVRGRSMLPANP----TEC--QDTSGAACFVSGDGRVNEQPDL 594

Query 284 GSIHLLFHLHHNHIVRLLVAGILKKRGQPSSPERIAKFIQESSSALKEQIFQEVRKMLGA 343

+H ++ HN + L Q PE E++FQE R+++ A

Sbjct 595 ALMHTVWLREHNRVASAL---------QALRPEWT-----------DEELFQEARRIVVA 634

Query 344 IIQKLTYCDWLPMILGPYLIDKFQL-----GCTRRSRYNSDLDPRVANSFLSAALRFGHT 398

+Q +TY ++LP++LG +D+ ++ G T + Y+ +L+P + N+F +AA RFGHT

Sbjct 635 EMQHITYNEFLPIVLGKPYMDRAEMSPKDSGYT--ALYDRELNPGITNAFATAAFRFGHT 692

Query 399 LIPNVYNFGDKRIHLKDTFNIPDASIRYY--------DNIIQCLIKEGSEEAYDRYVSSA 450

L+ + + +++ F + ++ + + D++++ L + S++ +DR+ +

Sbjct 693 LLVSNLQGVGRFGNVRKNFALSKSAFKPFMLYEEEGLDDMLRGLTTQSSQK-FDRFFTKE 751

Query 451 VSEHLFESTRGHKHALDLIAVNIQRGRDHGIPAYHYWRQYYRLRRIISLDEFG-----EA 505

++ HLF++ LDL+A+N+QRGRDHG+P Y WRQ LRR S ++ +A

Sbjct 752 ITNHLFQND--LPFGLDLVALNLQRGRDHGLPGYPEWRQVCGLRRPRSWEDLQGIMDPDA 809

Query 506 GIAMKKAYRDIRDVDLFPGGLLEPSMPGGVVGETFGHILANQFADLKFGDTYFFLHQQAP 565

++ Y + +VDLF G+ E PG ++G TF I+ +QF L+ GD +F+ P

Sbjct 810 ISVLQSLYPSVEEVDLFAAGVSERPAPGALLGPTFTCIVGDQFGRLRRGDRFFYEEGNQP 869

Query 566 QGFRAAQIKAILSVTMSSIICANS-AVTQAQPDPFYMASQLNLPRPCSDYS--EMDVEPW 622

F+ Q++ I +++ ++C NS + QP F+ AS +N C+ + +D+ W

Sbjct 870 SSFKPEQLQQIRRTSLARVLCDNSDNIALMQPLAFFHASFVNQRVACNSDAIPRLDIRAW 929

>[XP_033761812.1](https://www.ncbi.nlm.nih.gov/protein/XP_033761812.1?report=genbank&log$=protalign&blast_rank=45&RID=0) peroxidase-like [Pecten maximus]

Length=727

Score = 303 bits (775), Expect = 2e-89, Method: Compositional matrix adjust.

Identities = 213/608 (35%), Positives = 307/608 (50%), Gaps = 69/608 (11%)

Query 48 SRLTYD-QLRYRQIDGRCN---HPRNYGSTGRPVKRYLRPHYQDKFGENLPRVYSVTGQL 103

+++T D + YR +G CN HP ++G P +RYL+P Y D +LPR G+L

Sbjct 141 TKITCDPKTLYRSANGSCNNLNHP-SWGMAFTPQQRYLQPSYDDWI--DLPRKKCHAGRL 197

Query 104 LPSPRMVSWKLHPDQTAH----DNNTMLVMQMGQFIDHDITRAPELSGRNAS-IKCCGVP 158

LPSPR +S L ++ N+T++VM GQFIDHDI P G N S I CC

Sbjct 198 LPSPRKISNTLFCEENHKPRIAKNHTIMVMAWGQFIDHDIVFTPLPKGANGSAISCCNKD 257

Query 159 PKERL-PDCFPIDIPPGDPVF--EDCMEFFRSSPAVDNDGNIIYPREQINALTSFIDGSA 215

ERL P+CFPI+IP DP F + CM F RS+ A + + PREQ+N +TSFID S

Sbjct 258 SSERLRPECFPIEIPKEDPYFGNKTCMNFIRST-ASPSHSCLPAPREQLNQVTSFIDAST 316

Query 216 VYGSDLDTYTWIRSENGTGVFLNTHLVHGRERLPSHPHLGPESCVSSNTAESYCQLAGDM 275

VYGS +R+ G+ L + + LP P +SCV ++ E YC AGD

Sbjct 317 VYGSSEKETNSLRAMK-KGLLLTS-----KGGLP--PVGKEKSCVLTHPGE-YCFRAGDK 367

Query 276 RVNEQPGLGSIHLLFHLHHNHIVRLLVAGILKKRGQPSSPERIAKFIQESSSALKEQIFQ 335

RVN P L H+LF HN I + R+A+ + + E +FQ

Sbjct 368 RVNVVPNLSVTHILFIKQHNRIAK-----------------RLARMNRHWTD---EIVFQ 407

Query 336 EVRKMLGAIIQKLTYCDWLPMILGPYLIDKFQLG---CTRRSRYNSDLDPRVANSFLSAA 392

E RK++ A++Q +TY ++LP L + ++ L YN ++P NSF +AA

Sbjct 408 ETRKIIIALMQHITYREYLPKTLNAEHMSRYGLNYYPGKFNDVYNPLINPGTRNSFAAAA 467

Query 393 LRFGHTLIPNVYNFGD------KRIHLKDTFNIPDASIRYYDNIIQCLIKEGSEE---AY 443

R+GH+ IP + K L++T++ P + + L++ S E

Sbjct 468 FRYGHSQIPPTQSVLSSDHVTYKHYPLEETYHKPFLAQEEKGKNLAGLLRWLSTEPTTCN 527

Query 444 DRYVSSAVSEHLFESTRGHKHALDLIAVNIQRGRDHGIPAYHYWRQYYRLRRIISLDEFG 503

DR V + LF +G+ +LDL A+NIQRGRDHG+P Y+ WR++ L S D G

Sbjct 528 DRVFEPQVRDLLFLDKKGN--SLDLPAINIQRGRDHGLPCYNEWREWCGLPVAKSFDT-G 584

Query 504 EAGIA---------MKKAYRDIRDVDLFPGGLLEPSMPGGVVGETFGHILANQFADLKFG 554

E G+ +KKAY + D+DL+ G + E + G VG TF I+A QF K+G

Sbjct 585 EGGLVDHDPVCAGLLKKAYGHVNDIDLYAGAISEKHITDGEVGPTFACIIARQFHAYKYG 644

Query 555 DTYFFLHQQAPQGFRAAQIKAILSVTMSSIICANSAVTQAQPDPFYMASQLNLPRPCSDY 614

D +++ H P F AQ++ I +T+S I C N + + Q D F S N C +

Sbjct 645 DRFWYEHAGKPTAFTRAQLRQIKKITLSKITCDNFKIKKIQKDSFTTLSPSNALTACKNL 704

Query 615 SEMDVEPW 622

+M + W

Sbjct 705 PKMKLRAW 712

>[XP_034310729.1](https://www.ncbi.nlm.nih.gov/protein/XP_034310729.1?report=genbank&log$=protalign&blast_rank=46&RID=0) eosinophil peroxidase-like [Crassostrea gigas]

Length=694

Score = 301 bits (772), Expect = 3e-89, Method: Compositional matrix adjust.

Identities = 213/592 (36%), Positives = 296/592 (50%), Gaps = 61/592 (10%)

Query 57 YRQIDGRCN---HPRNYGSTGRPVKRYLRPHYQDKFGENLPRVYSVTGQLLPSPRMVSWK 113

YR IDGRCN HP +G+ P RY Y D G + PR G LPSPR +S K

Sbjct 135 YRSIDGRCNNLIHPL-WGAANTPQPRYSPAEYDD--GISTPRSRGKDGSPLPSPRQISNK 191

Query 114 LH--PDQTAHDNN--TMLVMQMGQFIDHDITRAPELSGR-NASIKCCGVPPKERLPDCFP 168

L P + ++ T++VM GQFIDHD+ P + G + I CCG + R P CFP

Sbjct 192 LFRAPRECTETDHARTLMVMAWGQFIDHDLAHTPTMKGDGDVPITCCGENVQNR-PQCFP 250

Query 169 IDIPPGDPVFED-CMEFFRSSPAVDNDGNIIYPREQINALTSFIDGSAVYGSDLDTYTWI 227

I IP DP F D CMEF RS+P+ DG + PREQIN +TSFIDG +VYG+ +

Sbjct 251 ISIPSDDPHFNDTCMEFVRSAPSPPGDGCQLGPREQINQITSFIDGGSVYGNSAKKMAEL 310

Query 228 RSENGTGVFLNTHLVHGRERLPSHPHLGPESCVSSNTAESYCQLAGDMRVNEQPGLGSIH 287

+++ + LP P + + +NT + +CQ AGD RVNE P LG H

Sbjct 311 KNK-----YTGQMRTSAGNLLP--PAVNGTCELPANTTD-FCQNAGDSRVNEVPFLGGNH 362

Query 288 LLFHLHHNHIVRLLVAGILKKRGQPSSPERIAKFIQESSSALKEQIFQEVRKMLGAIIQK 347

L+F HN IVR L + +Q S+LK ++QE RK++GA++Q+

Sbjct 363 LMFVREHNRIVREL------------------RKVQPRWSSLK--LYQEARKIIGALLQQ 402

Query 348 LTYCDWLPMILGPYLIDKFQLGCTR---RSRYNSDLDPRVANSFLSAALRFGHTLIP--N 402

+TY ++LP IL ++K +L + YN L+P N F +A RFGH+LIP

Sbjct 403 VTYREFLPSILRKQDLEKHKLKLRNWGFSNSYNCSLNPGTKNVFNAAVFRFGHSLIPLDL 462

Query 403 VYNFGDKRIHL-----KDTFNIPDASIRYYDNIIQCL---IKEGSEEAYDRYVSSAVSEH 454

Y D HL + TF P I + L I + D + AV

Sbjct 463 AYLLYDFMSHLNSTPIESTFMNPHLLITKGGRRVSDLARFIATANSMKLDNQLEGAVRNR 522

Query 455 LFESTRGHKHALDLIAVNIQRGRDHGIPAYHYWRQYYRLRRIISL----DEFGEAGIAMK 510

LFE+ +G +DL A+N+ RGRDHG+P Y+ WR++ L S D E

Sbjct 523 LFENEQG--KGMDLGALNLARGRDHGLPPYNAWRKWCGLPVATSFSNLPDISDEKKAIFA 580

Query 511 KAYRDIRDVDLFPGGLLEPSMPGGVVGETFGHILANQFADLKFGDTYFFLHQQAPQGFRA 570

Y ++ D+D+F GG+ E + G VG F I+ NQF DLK GD Y++ + +GF

Sbjct 581 DLYSNVDDIDVFAGGIAETPLDGAAVGPLFSCIIGNQFRDLKDGDRYWY-ENRGVEGFTL 639

Query 571 AQIKAILSVTMSSIICANSAVTQAQPDPFYMASQLNLPRPCSDYSEMDVEPW 622

Q++ I V ++ IIC N V Q D F++ S N + C ++ W

Sbjct 640 GQLQQIRRVKLAKIICQNLGVDPIQRDVFHVPSPRNRWQRCRRLPGINFYYW 691

>[XP_019616874.1](https://www.ncbi.nlm.nih.gov/protein/XP_019616874.1?report=genbank&log$=protalign&blast_rank=47&RID=0) PREDICTED: peroxidasin-like [Branchiostoma belcheri]

Length=570

Score = 298 bits (763), Expect = 3e-89, Method: Compositional matrix adjust.

Identities = 208/596 (35%), Positives = 309/596 (52%), Gaps = 71/596 (12%)

Query 57 YRQIDGRCNHPRN---YGSTGRPVKRYLRPHYQDKFGENLPRVYSVTGQLLPSPRMVSWK 113

+R +GRCN+ RN +GS+ +P++R L+P Y D G PR G LPS R+VS

Sbjct 14 FRSANGRCNN-RNHPLWGSSEQPLRRLLQPEYGD--GLMTPRTTGHDGAPLPSARLVSTT 70

Query 114 LHPD-QTAHDNNTMLVMQMGQFIDHDITRAPELSGRNASIKCCGVPPKERLPDCFPIDIP 172

+H D + + NT +VMQ GQF+DHDIT P C KE CF I+IP

Sbjct 71 MHEDLRKSSPVNTNMVMQFGQFLDHDITLTPNFQEEGLVCTCGTNCEKEE--RCFNINIP 128

Query 173 PGDPVF---EDCMEFFRSSPAVDNDGNIIYPREQINALTSFIDGSAVYGSDLDTYTWIRS 229

DP F C+ F RS + N+G + R+Q+N +T+F+D S VYGS + +R

Sbjct 129 SDDPDFAAESRCLPFARSR-SCPNEGCHMGRRQQLNQITAFVDASNVYGSSDEEIEELRE 187

Query 230 ENGTGVFLNTHLVHGRERLPSHPHLGPESCVSSNTAESYCQLAGDMRVNEQPGLGSIHLL 289

G + +E LP+ + E + C AGD+RVNEQPGL S+H +

Sbjct 188 HAGN--------ANKKELLPAA--IAEEFECHESPGNETCSQAGDIRVNEQPGLTSMHTV 237

Query 290 FHLHHNHIVRLLVAGILKKRGQPSSPERIAKFIQESSSALKEQIFQEVRKMLGAIIQKLT 349

F HN I R L R P + +++F E RK++GA++QK+T

Sbjct 238 FLREHNRIARRL------SRLNPHWDD--------------DRVFFETRKIVGALMQKIT 277

Query 350 YCDWLPMILGPYLIDKFQLGCTRR---SRYNSDLDPRVANSFLSAALRFGHTLIPNV--- 403

Y + LP +LGP + KF L + S Y++ ++P ++N F +AA RFGH+L+

Sbjct 278 YGEDLPHVLGPAAMTKFHLTLGKSGFFSGYDASVNPTISNVFATAAYRFGHSLVNERQLR 337

Query 404 ----YNFGDK-RIHLKDTFNIP----DASIRYYDNIIQCLIKEGSEEAYDRYVSSAVSEH 454

+N G K I + F P + + D+I++ L + ++ +DR++ S+++ H

Sbjct 338 LTPDFNQGSKCPIQIAFAFFNPSHVLNNDLGGPDSILRGLTAQPHQD-FDRFMVSSLTRH 396

Query 455 LFESTRGHKHALDLIAVNIQRGRDHGIPAYHYWRQYYRLRRIISLDEF------GEAGIA 508

LF G LDL A+NIQRGRDHG+P Y+ WR L + S DE +

Sbjct 397 LFADPPG-SLGLDLAALNIQRGRDHGLPGYNAWRVRCGLPKADSFDELVFEIPDRYTRMR 455

Query 509 MKKAYRDIRDVDLFPGGLLEPSMPGGVVGETFGHILANQFADLKFGDTYFFLHQQAPQGF 568

++ Y + D+D+F GGL E S+ GGVVG TF ++ QF DL+ GD ++F ++ F

Sbjct 456 LEDIYSHVDDIDVFVGGLAEESVRGGVVGPTFACLIGLQFQDLRKGDRFWFENRGQ---F 512

Query 569 RAAQIKAILSVTMSSIICANS-AVTQAQPDPFYMASQLNLPR-PCSDYSEMDVEPW 622

AAQ+ I +++ I+C N+ T QPD F + +Q R CS S+MD+ W

Sbjct 513 TAAQLAEIRKTSLARILCDNTDGTTHMQPDVFSLPTQTGNERVACSSLSQMDLTKW 568

>[XP_013419078.1](https://www.ncbi.nlm.nih.gov/protein/XP_013419078.1?report=genbank&log$=protalign&blast_rank=48&RID=0) peroxidasin-like [Lingula anatina]

Length=754

Score = 303 bits (775), Expect = 4e-89, Method: Compositional matrix adjust.

Identities = 209/617 (34%), Positives = 310/617 (50%), Gaps = 78/617 (13%)

Query 56 RYRQIDGRCNHPR--NYGSTGRPVKRYLRPHYQDKFGENLPRVYSVTGQLLPSPRMVSWK 113

+YR DG CN+ + N+G + P KR+L P Y+D G N R LP+PR VS

Sbjct 80 KYRSFDGSCNNLKKPNWGKSFEPFKRFLHPMYED--GVNTERSKGRFSVPLPNPRAVSSS 137

Query 114 LHPDQTAHDNN---TMLVMQMGQFIDHDITRAPELSGRNAS-IKCCG-----------VP 158

+H N+ T L+M GQF+DHD+T P G N S I CC +P

Sbjct 138 IHEGSNTTLNSKSHTRLLMLFGQFLDHDLTHTPVPKGINGSEINCCADDVLEHNRNVRIP 197

Query 159 PKE------RLPDCFPIDIPPGDPVFE-DCMEFFRSSPAVDNDGNIIYPREQINALTSFI 211

+E + +C PIDIP GD F CM F RS P ++ + Y REQ+N +TS+I

Sbjct 198 REELASLLRKRSECMPIDIPTGDRRFRRSCMNFIRSLPTPNDKCELGY-REQLNQVTSYI 256

Query 212 DGSAVYGSDLDTYTWIRSENGTGVFLNTHLVHGRERLPSHPHLGPESCVS-SNTAESYCQ 270

D S VYGS + +RS G L T +V+G+ +P + CV+ +N E C

Sbjct 257 DASQVYGSTEEQARNLRS--FFGGLLKTSVVNGKMFMPKDNSNQEKECVTPANKPEIKCF 314

Query 271 LAGDMRVNEQPGLGSIHLLFHLHHNHIVRLLVAGILKKRGQPSSPERIAKFIQESSSALK 330

+AGD R NEQ L IH +F HN I L +R P +

Sbjct 315 IAGDERSNEQLTLTMIHTMFVREHNRIATTL------QRYNPHWDD-------------- 354

Query 331 EQIFQEVRKMLGAIIQKLTYCDWLPMILGPYLIDKFQLGCTRR---SRYNSDLDPRVANS 387

E+ +QE R+++GA++Q +TY WLPM+LG +D F++G YN ++DP + N+

Sbjct 355 ERTYQETRRIVGAMLQHITYSMWLPMVLGHRGVDCFEVGVGTSGYFKGYNENIDPSIRNA 414

Query 388 FLSAALRFGHTLI-PNVYNFGDK-----RIHLKDTFNIPDASIRY----YDNIIQCLIKE 437

F +A RFGH+L+ ++ +G I LK+ F P+ ++I + + K+

Sbjct 415 FAAAGFRFGHSLVMEHIARYGRGYTPLPSIPLKNAFFKPEELYNTEQGGMESIARGIFKD 474

Query 438 GSEEAYDRYVSSAVSEHLFESTRGHKHALDLIAVNIQRGRDHGIPAYHYWRQYYRLRR-- 495

E+ DR+++ AV++HLFE + ALDL A+NIQRGRDH +P Y+ WR + L +

Sbjct 475 PMEQC-DRHLTPAVTDHLFEDPHS-RIALDLAALNIQRGRDHALPPYNDWRHWCGLPKAR 532

Query 496 --------IISLDEFGEAGIAMKKAYRDIRDVDLFPGGLLEPSMPGGVVGETFGHILANQ 547

++++D+ I+ + Y D+DLF GG+ E +PGG+VG TF IL Q

Sbjct 533 HFFTSKDGLVNMDDVTAKKIS--EIYNHPDDIDLFTGGMAELPVPGGIVGPTFACILGRQ 590

Query 548 FADLKFGDTYFFLHQQAPQGFRAAQIKAILSVTMSSIICANSAVTQAQPDPFYMASQLNL 607

FA LKFGD +F+ + F Q++ + M+ + C N + + F M+

Sbjct 591 FAALKFGDRFFYENGDEDIRFTEDQLEELRKTDMARLFCDNFNIASIPRNVFLMSDDTTN 650

Query 608 PR-PCSDYSEMDVEPWL 623

P C MD+ W+

Sbjct 651 PLVDCRTVKSMDLRRWI 667

>[RUS85745.1](https://www.ncbi.nlm.nih.gov/protein/RUS85745.1?report=genbank&log$=protalign&blast_rank=49&RID=0) hypothetical protein EGW08_006459, partial [Elysia chlorotica]

Length=564

Score = 297 bits (760), Expect = 7e-89, Method: Compositional matrix adjust.

Identities = 188/582 (32%), Positives = 293/582 (50%), Gaps = 48/582 (8%)

Query 57 YRQIDGRCNHPRN--YGSTGRPVKRYLRPHYQDKFGENLPRVYSVTGQLLPSPRMVSWKL 114

YR DG CN+ N YG TG +R L P Y + PR+ + +G LP+PR +S +

Sbjct 9 YRSFDGSCNNLANPSYGQTGSAFRRILAPAYGGDGTGSTPRLSASSGGDLPNPRAISVLI 68

Query 115 H-PDQTAHDNNTMLVMQMGQFIDHDITRAPELSGRNASIKCCG--VPPKERLPD------ 165

H P HD+ T +VMQ GQF+DHDIT P S NA++ C V E D

Sbjct 69 HDPADFTHDSFTQMVMQWGQFLDHDITDTPTAS--NATVCCYDSLVTSNEVHSDVTTGGA 126

Query 166 CFPIDIPPGDPVFED----CMEFFRSSPAVDNDGNIIYPREQINALTSFIDGSAVYGSDL 221

CFPI I GD F+ CM F+RS P D+DG R+Q NA T +IDGS +YG+

Sbjct 127 CFPIIIAEGDRYFDTVSTRCMVFYRSDPITDSDG----VRQQYNAATPWIDGSQIYGTSE 182

Query 222 DTYTWIRSENGTGVFLNTHLVHGRERLPSHPHLGPESCVSSNTAESYCQLAGDMRVNEQP 281

+RS + + + T + + LP C + E YC LAGD RVN

Sbjct 183 TVAASLRSGSQGKLLVRT--IGDEDFLPEDSSSSDSVCFVRESGE-YCFLAGDHRVNVFA 239

Query 282 GLGSIHLLFHLHHNHIVRLLVAGILKKRGQPSSPERIAKFIQESSSALKEQIFQEVRKML 341

GL + H +F +HN I L+A PS + E FQE R+++

Sbjct 240 GLSAFHTIFVRYHNTICDRLLAL------NPSWDD--------------ETTFQEARRIV 279

Query 342 GAIIQKLTYCDWLPMILGPYLIDKFQLGCTRRS-RYNSDLDPRVANSFLSAALRFGHTLI 400

A++Q +TY +++ ILG + + + L + Y++ +DP ++N F +AA RFGH+++

Sbjct 280 VAVLQVITYKEFMRHILGTTVANTYDLLVGDENYAYDASVDPTLSNVFSAAAYRFGHSML 339

Query 401 PNVYNFGDKRIHLKDTFNIPDASIRYYDNIIQCLIKEGSEEAYDRYVSSAVSEHLFESTR 460

+ D + P ++ ++ L E DR+ + ++++ +FE+

Sbjct 340 SDSLTVNGTATLTSDLYMRPKYTLNSMAAVLDGLQDEKLFRT-DRWYTQSLTDRMFETPE 398

Query 461 GHKHALDLIAVNIQRGRDHGIPAYHYWRQYYRLRRIISLDEFGEAGIAMKKAYRDIRDVD 520

+ D+ A NIQRGRDHG+P+Y+ WR++Y L + S DE + + Y + D+D

Sbjct 399 SPQSGFDVAARNIQRGRDHGLPSYNAWREHYGLGKKTSFDEMDQGSARYSRLYESVDDID 458

Query 521 LFPGGLLEPSMPGGVVGETFGHILANQFADLKFGDTYFFLHQQAPQGFRAAQIKAILSVT 580

L+ GG+ E + GG VGE + I+ QF DLKFGD ++F + F + QI + V+

Sbjct 459 LYSGGVSENLVSGGRVGELYAIIMGEQFKDLKFGDRFWFENTGDVSSFSSKQINQLRRVS 518

Query 581 MSSIICAN-SAVTQAQPDPFYMASQLNLP-RPCSDYSEMDVE 620

++ ++C + + + PF + P PCS+ ++++E

Sbjct 519 LARVLCDTVTGIESVRESPFKALDDASNPLTPCSEILDINIE 560

>[TRY68556.1](https://www.ncbi.nlm.nih.gov/protein/TRY68556.1?report=genbank&log$=protalign&blast_rank=50&RID=0) hypothetical protein TCAL_04086 [Tigriopus californicus]

Length=800

Score = 303 bits (775), Expect = 1e-88, Method: Compositional matrix adjust.

Identities = 214/628 (34%), Positives = 328/628 (52%), Gaps = 80/628 (13%)

Query 34 GSLDFVSEADLEHCSRLTYDQLRYRQIDGRCNHPRN--YGSTGRPVKRYLRPHYQDKFGE 91

GS FV EA + C + L++R IDG CN+ +N +G ++R L P Y+D G

Sbjct 210 GSTGFVREA--KKCRNV---DLKFRSIDGSCNNRQNAIWGQANVALQRLLHPEYED--GI 262

Query 92 NLPRVY---SVTGQLLPSPRMVSWKLHPDQTAHDNN----TMLVMQMGQFIDHDITRAPE 144

+ PR T LP+PR VS L PD +DN T+++MQ GQF+DHD+T P

Sbjct 263 SRPRGQLGDDPTSSSLPTPREVSEALVPD---NDNPSPEFTLILMQWGQFVDHDVTHTPL 319

Query 145 LSGRN-ASIKCCG----VPPKERLPDCFPIDIPPGDPVF----EDCMEFFRSSPAVDNDG 195

+ G + I CC V P+C PI I P DP + + CMEF RS PA + +

Sbjct 320 VEGDDDTEILCCSNGDVVRGSLSHPECLPISISPSDPFYGRAGQRCMEFVRSMPARNAEC 379

Query 196 NIIYPREQINALTSFIDGSAVYGSDLDTYTWIRSENGTGVFLNTHLVHGRERLPSHPHLG 255

++ REQ+N +T+FIDGS +YGSD +R + + + G E LP L

Sbjct 380 HL-GAREQVNQITAFIDGSNIYGSDTSESRLLRLGRRGRLRVTQY--GGAELLP----LD 432

Query 256 PESCVSSNTAESYCQLAGDMRVNEQPGLGSIHLLFHLHHNHIVRLLVAGILKKRGQPSSP 315

P+ C + ++ YC AGD R NEQP L +H L+ HN IV L +L

Sbjct 433 PDEC-ADHSKRQYCFSAGDGRCNEQPQLTVMHTLWMREHNRIVSQL--SLL--------- 480

Query 316 ERIAKFIQESSSALKEQIFQEVRKMLGAIIQKLTYCDWLPMILGPYLIDKFQLGCTRRS- 374

+S E+++QE RK++GA +Q +TY +WLP+ILG +D+ L +R

Sbjct 481 ---------NSHWDDERLYQEARKIVGAELQHITYNEWLPIILGMTYMDRNNLSPSRSGL 531

Query 375 --RYNSDLDPRVANSFLSAALRFGHTLIP-NVYNFGDKRIHLKDTFNIPDASIRYY---- 427

Y+ ++P + N+F +AA RFGH+L+ ++ +FG LK + ++ + Y

Sbjct 532 TQDYDPTIEPSITNAFATAAFRFGHSLVQGHIESFGSFGNRLK-SLDLHSQQLEPYELYQ 590

Query 428 ----DNIIQCLIKEGSEEAYDRYVSSAVSEHLFESTRGHKHALDLIAVNIQRGRDHGIPA 483

D+ ++ L + ++E D S +++HLF+ + H +DL+A+N+QRGRDHG+P

Sbjct 591 QNSIDSFLRGLTTQPAQE-LDTSFSRELTDHLFQESNA-THGMDLVALNLQRGRDHGLPG 648

Query 484 YHYWRQYYRL------RRIISLDEFGEAGIAMKKAYRDIRDVDLFPGGLLEPSMPGGVVG 537

Y +R+ L R ++++ E +++ Y I D+DLF GG+LE S+PG +VG

Sbjct 649 YTAYRKLCGLSPLSSWREMVNVVSSPELVPRLQRLYSSINDIDLFVGGILERSVPGALVG 708

Query 538 ETFGHILANQFADLKFGDTYFFLHQQAPQGFRAAQIKAILSVTMSSIICANS-AVTQAQP 596

TF I+ +QF LKFGD ++F F Q+ A+ ++S IIC N + Q QP

Sbjct 709 PTFQCIVGDQFKRLKFGDRFWFEEGNQLSSFTLDQVNALRESSLSRIICDNGDNIQQVQP 768

Query 597 DPFYMASQLNLPRPCSDYS--EMDVEPW 622

F A+ +N C + +++E W

Sbjct 769 LAFKKANGMNQKASCRGNAIPRVNLEAW 796

>[XP_033756984.1](https://www.ncbi.nlm.nih.gov/protein/XP_033756984.1?report=genbank&log$=protalign&blast_rank=51&RID=0) peroxidasin-like [Pecten maximus]

Length=710

Score = 300 bits (768), Expect = 1e-88, Method: Compositional matrix adjust.

Identities = 205/604 (34%), Positives = 304/604 (50%), Gaps = 78/604 (13%)

Query 56 RYRQIDGRCNHPRN--YGSTGRPVKRYLRPHYQDKFGENLPRVYSVTGQLLPSPRMVSWK 113

R+R IDG CN+P N +G KRYLRP Y D G N PR+ G LPS R+VS

Sbjct 139 RFRTIDGSCNNPFNPTWGMAATQQKRYLRPVYHD--GINSPRLMGRDGLPLPSARLVSNV 196

Query 114 LHPD-----QTAHDNNTMLVMQMGQFIDHDITRAPELSGRN-ASIKCCGVPPKERLPDCF 167

+H Q H + +VMQ GQF+DHD+ P L N +S +CCG CF

Sbjct 197 VHSSGGFVRQDCH--LSAMVMQWGQFLDHDLVGTPVLKKDNGSSYECCGA--DSSFDGCF 252

Query 168 PIDIPPGDPVFE-DCMEFFRSSPAVDNDGNIIYPREQINALTSFIDGSAVYGSDLDTYTW 226

P+DIPP D FE CM RS+PAV + I REQ+N +TS+ID SAVYGS + +

Sbjct 253 PVDIPPNDDHFEGTCMNMVRSAPAVGPECTIGV-REQMNKVTSYIDASAVYGSSREEEDF 311

Query 227 IRSENGTGVFLNTHLVHGRERLPSHPHLGPESCVSSNTAESYCQLAGDMRVNEQPGLGSI 286

+R + L G R+ P G ++C+ T++ C AGD R N P LG+I

Sbjct 312 LR------IKTRGLLKEGENRM--LPPSGKDNCIQ-RTSDDVCMTAGDDRPNVVPSLGAI 362

Query 287 HLLFHLHHNHIVRLLVAGILKKRGQPSSPERIAKFIQESSSALKEQIFQEVRKMLGAIIQ 346

H LF HN I +L G+L + F + E+I+QE R+++ A+IQ

Sbjct 363 HTLFMREHNRIAAML--GVLNR------------FWDD------ERIYQETRRIMSALIQ 402

Query 347 KLTYCDWLPMILGPYLIDKF----QLGCTRRSRYNSDLDPRVANSFLSAALRFGHTLIPN 402

+TY ++LP+ILG +++ + + S YN+ + +AN F AA RFGH+ IP+

Sbjct 403 HITYFEYLPLILGKRIMEYYDILPNIESLEASVYNTSTNAGIANVFAVAAFRFGHSQIPS 462

Query 403 VYNFGDKRIHLKDTFNIPDASIRYY----------DNIIQCLIKEGSEEAYDRYVSSAVS 452

+ F R + F I R Y +++++ L+K+ E +R V

Sbjct 463 IQTFIGPRESYRRDFRIESTYHRPYFAQFNNGQGAEDVLRWLVKDSQPET-NRVFVRGVR 521

Query 453 EHLFESTRGHKHALDLIAVNIQRGRDHGIPAYHYWRQYYRLRRIISLDEFGEAGIA---- 508

+ LF + +LDL A+NIQR RDHG+ Y+ WR++ L + G+ G+

Sbjct 522 DQLFLR---NNVSLDLAAINIQRSRDHGVAPYNSWRKWCGLDSVRHFG-VGQDGLVHHDP 577

Query 509 -----MKKAYRDIRDVDLFPGGLLEPSMPGGVVGETFGHILANQFADLKFGDTYFFLHQQ 563

+++AYRD D+D+ G + E + G VG TF I+ QF +LK GD +++

Sbjct 578 DTARLLQRAYRDPDDIDVLTGAISERRIKGASVGPTFACIIGYQFKELKMGDRFWYERAH 637

Query 564 APQGFRAAQIKAILSVTMSSIICANSAVTQAQPDPFYM-----ASQLNLPRPCSDYSEMD 618

GF Q+++I + +S I+C N +T Q + F + S+ P PC ++D

Sbjct 638 PITGFTDRQLRSIRRMRLSKILCENFNITPLQKNVFLLPDSRGKSKSTHPVPCHSLPDLD 697

Query 619 VEPW 622

+ W

Sbjct 698 IRAW 701

>[XP_036355162.1](https://www.ncbi.nlm.nih.gov/protein/XP_036355162.1?report=genbank&log$=protalign&blast_rank=52&RID=0) peroxidase-like protein isoform X1 [Octopus vulgaris]

Length=825

Score = 303 bits (775), Expect = 2e-88, Method: Compositional matrix adjust.

Identities = 200/610 (33%), Positives = 315/610 (52%), Gaps = 80/610 (13%)

Query 56 RYRQIDGRCNHPRN--YGSTGRPVKRYLRPHYQDKFGENLPRVYSVTGQLLPSPRMVSWK 113

++R+ DG CN+ + +G G P+KR+L P Y D G + PR V G LPS R+VS

Sbjct 182 KFREADGSCNNLQKPLWGKAGMPLKRFLSPWYDD--GVSSPRTLGVFGNPLPSARLVSTT 239

Query 114 LHPDQTAHDNN---TMLVMQMGQFIDHDITRAPELSG-RNASIKCC--GVPP--KERLP- 164

+H + + N + +VMQ GQFIDHDIT P +G ++A I CC +P ++RL

Sbjct 240 IHEADSENTRNPFVSHMVMQWGQFIDHDITNTPVDTGFQHADISCCIDDIPDDVRKRLEV 299

Query 165 ------DCFPIDIPPGDPVFED-CMEFFRSSPAVDNDGNIIYPREQINALTSFIDGSAVY 217

CFPI IP D F+ C+ F RS + ++ N + REQ+N ++++IDG AVY

Sbjct 300 DLRNRESCFPIPIPSNDRHFKKTCLNFVRSMQSPNSKCNFGF-REQVNQISAYIDGGAVY 358

Query 218 GSDLDTYTWIRSEN-GTGVFLNTHLVHGRERLPSHPHLGPESCVSSNTAESYCQLAGDMR 276

S + +R+ + G HL+ P +SCV + ++++YC AGD R

Sbjct 359 ASTKEDQNELRTRSQGLLKESGAHLL---------PKDSQQSCVLT-SSDNYCFRAGDRR 408

Query 277 VNEQPGLGSIHLLFHLHHNHIVRLLVAGILKKRGQPSSPERIAKFIQESSSALKEQIFQE 336

VNEQ GL S+H +F HN I R +F E++FQE

Sbjct 409 VNEQMGLASLHTIFLREHNRIAR--------------------QFNSMGLGWKDERVFQE 448

Query 337 VRKMLGAIIQKLTYCDWLPMILGPYLIDKFQLGCTRRSR---YNSDLDPRVANSFLSAAL 393

++++ A+IQ + Y ++LP IL I+ L RR Y+S +D + N F +AA

Sbjct 449 TKRIISAMIQHINYREFLPAILNRQFINFLNLNGPRRGFHDIYDSTVDATIRNGFSTAAF 508

Query 394 RFGHTLIPNVYN---------FGDKRIHLKDTFNIPDASIR----YYDNIIQCLIKEGSE 440

RFGH+++ + ++ FG + LK + + ++ D ++ L+ + ++

Sbjct 509 RFGHSMVRSFFSKLSSGFNSAFGSPTL-LKTMYGKTEGILQNQPESVDAFVRGLVSDSAQ 567

Query 441 EAYDRYVSSAVSEHLFESTRGHKHALDLIAVNIQRGRDHGIPAYHYWRQYYRLRRIIS-- 498

A DR++S +++HLFE T G+ +LDL + NIQRGRDHGIP Y+ WRQ+ +

Sbjct 568 NA-DRFMSKQLTDHLFEDTFGN--SLDLASFNIQRGRDHGIPPYNVWRQWCDFSTATNFG 624

Query 499 ------LDEFGEAGIAMKKAYRDIRDVDLFPGGLLEPSMPGGVVGETFGHILANQFADLK 552

+D ++ +K Y D+DLF GGL E + GG+VG TF I+ QF +K

Sbjct 625 TGPGGLIDHSFDSANKLKSIYSHPDDIDLFSGGLSENPIRGGIVGPTFACIIGRQFHLIK 684

Query 553 FGDTYFFLHQQAPQGFRAAQIKAILSVTMSSIICANSAVTQAQPDPFYMASQLNLPRPCS 612

GD +++ GF Q+ I ++S+IIC N+ +++ QP+ F +++ N C

Sbjct 685 VGDRFWYERNDPTVGFTLNQLDQIRQTSLSAIICTNTNISRIQPNSFLLSNGNNRLVSCD 744

Query 613 DYSEMDVEPW 622

+ D+ W

Sbjct 745 SLPKFDLSAW 754

>[XP_029656077.1](https://www.ncbi.nlm.nih.gov/protein/XP_029656077.1?report=genbank&log$=protalign&blast_rank=53&RID=0) peroxidase-like protein isoform X2 [Octopus vulgaris]

Length=817

Score = 303 bits (775), Expect = 2e-88, Method: Compositional matrix adjust.

Identities = 200/610 (33%), Positives = 315/610 (52%), Gaps = 80/610 (13%)

Query 56 RYRQIDGRCNHPRN--YGSTGRPVKRYLRPHYQDKFGENLPRVYSVTGQLLPSPRMVSWK 113

++R+ DG CN+ + +G G P+KR+L P Y D G + PR V G LPS R+VS

Sbjct 174 KFREADGSCNNLQKPLWGKAGMPLKRFLSPWYDD--GVSSPRTLGVFGNPLPSARLVSTT 231

Query 114 LHPDQTAHDNN---TMLVMQMGQFIDHDITRAPELSG-RNASIKCC--GVPP--KERLP- 164

+H + + N + +VMQ GQFIDHDIT P +G ++A I CC +P ++RL

Sbjct 232 IHEADSENTRNPFVSHMVMQWGQFIDHDITNTPVDTGFQHADISCCIDDIPDDVRKRLEV 291

Query 165 ------DCFPIDIPPGDPVFED-CMEFFRSSPAVDNDGNIIYPREQINALTSFIDGSAVY 217

CFPI IP D F+ C+ F RS + ++ N + REQ+N ++++IDG AVY

Sbjct 292 DLRNRESCFPIPIPSNDRHFKKTCLNFVRSMQSPNSKCNFGF-REQVNQISAYIDGGAVY 350

Query 218 GSDLDTYTWIRSEN-GTGVFLNTHLVHGRERLPSHPHLGPESCVSSNTAESYCQLAGDMR 276

S + +R+ + G HL+ P +SCV + ++++YC AGD R

Sbjct 351 ASTKEDQNELRTRSQGLLKESGAHLL---------PKDSQQSCVLT-SSDNYCFRAGDRR 400

Query 277 VNEQPGLGSIHLLFHLHHNHIVRLLVAGILKKRGQPSSPERIAKFIQESSSALKEQIFQE 336

VNEQ GL S+H +F HN I R +F E++FQE

Sbjct 401 VNEQMGLASLHTIFLREHNRIAR--------------------QFNSMGLGWKDERVFQE 440

Query 337 VRKMLGAIIQKLTYCDWLPMILGPYLIDKFQLGCTRRSR---YNSDLDPRVANSFLSAAL 393

++++ A+IQ + Y ++LP IL I+ L RR Y+S +D + N F +AA

Sbjct 441 TKRIISAMIQHINYREFLPAILNRQFINFLNLNGPRRGFHDIYDSTVDATIRNGFSTAAF 500

Query 394 RFGHTLIPNVYN---------FGDKRIHLKDTFNIPDASIR----YYDNIIQCLIKEGSE 440

RFGH+++ + ++ FG + LK + + ++ D ++ L+ + ++

Sbjct 501 RFGHSMVRSFFSKLSSGFNSAFGSPTL-LKTMYGKTEGILQNQPESVDAFVRGLVSDSAQ 559

Query 441 EAYDRYVSSAVSEHLFESTRGHKHALDLIAVNIQRGRDHGIPAYHYWRQYYRLRRIIS-- 498

A DR++S +++HLFE T G+ +LDL + NIQRGRDHGIP Y+ WRQ+ +

Sbjct 560 NA-DRFMSKQLTDHLFEDTFGN--SLDLASFNIQRGRDHGIPPYNVWRQWCDFSTATNFG 616

Query 499 ------LDEFGEAGIAMKKAYRDIRDVDLFPGGLLEPSMPGGVVGETFGHILANQFADLK 552

+D ++ +K Y D+DLF GGL E + GG+VG TF I+ QF +K

Sbjct 617 TGPGGLIDHSFDSANKLKSIYSHPDDIDLFSGGLSENPIRGGIVGPTFACIIGRQFHLIK 676

Query 553 FGDTYFFLHQQAPQGFRAAQIKAILSVTMSSIICANSAVTQAQPDPFYMASQLNLPRPCS 612

GD +++ GF Q+ I ++S+IIC N+ +++ QP+ F +++ N C

Sbjct 677 VGDRFWYERNDPTVGFTLNQLDQIRQTSLSAIICTNTNISRIQPNSFLLSNGNNRLVSCD 736

Query 613 DYSEMDVEPW 622

+ D+ W

Sbjct 737 SLPKFDLSAW 746

>[XP_013422154.2](https://www.ncbi.nlm.nih.gov/protein/XP_013422154.2?report=genbank&log$=protalign&blast_rank=54&RID=0) chorion peroxidase-like [Lingula anatina]

Length=920

Score = 305 bits (780), Expect = 2e-88, Method: Compositional matrix adjust.

Identities = 209/621 (34%), Positives = 312/621 (50%), Gaps = 80/621 (13%)

Query 56 RYRQIDGRCNHPR--NYGSTGRPVKRYLRPHYQD----KFGENLPRVYSVTGQLLPSPRM 109

+YR DG CN+ + N+G + P KR+L P Y+D G N R G LP+PR

Sbjct 240 KYRSFDGSCNNLKKPNWGKSFEPFKRFLHPMYEDGRSCAVGVNTERSKGRFGVPLPNPRA 299

Query 110 VSWKLHPDQTAHDNN---TMLVMQMGQFIDHDITRAPELSGRNAS-IKCCG--------- 156

VS +H N+ T L+M GQF+DHD+T P G N S I CC

Sbjct 300 VSSSIHEGSNTTLNSKSHTRLLMLFGQFLDHDLTHTPVPKGINGSEINCCADDVLEHNRN 359

Query 157 --VPPKE------RLPDCFPIDIPPGDPVFE-DCMEFFRSSPAVDNDGNIIYPREQINAL 207

+P +E + +C PIDIP GD F CM F RS P ++ + Y REQ+N +

Sbjct 360 VRIPREELASLLRKRSECMPIDIPTGDRRFRRSCMNFIRSLPTPNDKCELGY-REQLNQV 418

Query 208 TSFIDGSAVYGSDLDTYTWIRSENGTGVFLNTHLVHGRERLPSHPHLGPESCVS-SNTAE 266

TS+ID S VYGS + +R+ +G L T +V+G+ +P + CV+ +N E

Sbjct 419 TSYIDASQVYGSTEEQARNLRA--FSGGLLKTSVVNGKMFMPKDNSNQEKECVTPANKPE 476

Query 267 SYCQLAGDMRVNEQPGLGSIHLLFHLHHNHIVRLLVAGILKKRGQPSSPERIAKFIQESS 326

C +AGD R NEQ L IH +F HN I L +R P +

Sbjct 477 IKCFIAGDERSNEQLTLTMIHTMFVREHNRIATTL------QRYNPHWDD---------- 520

Query 327 SALKEQIFQEVRKMLGAIIQKLTYCDWLPMILGPYLIDKFQLGCTRR---SRYNSDLDPR 383

E+ +QE R+++GA++Q +TY WLPM+LG +D F++G YN ++DP

Sbjct 521 ----ERTYQETRRIVGAMLQHITYSMWLPMVLGHRGVDCFEVGVGTSGYFKGYNENIDPS 576

Query 384 VANSFLSAALRFGHTLI-PNVYNFGDK-----RIHLKDTFNIPDASIRY----YDNIIQC 433

+ N+F +A RFGH+L+ ++ +G I LK+ F P+ ++I +

Sbjct 577 IRNAFAAAGFRFGHSLVMEHIARYGRGYTPLPSIPLKNAFFKPEELYNSEQGGMESIARG 636

Query 434 LIKEGSEEAYDRYVSSAVSEHLFESTRGHKHALDLIAVNIQRGRDHGIPAYHYWRQYYRL 493

+ K+ E+ DR+++ AV++HLFE + ALDL A+NIQRGRDH +P Y+ WR + L

Sbjct 637 IFKDPMEQC-DRHLTPAVTDHLFEDPHS-RIALDLAALNIQRGRDHALPPYNDWRHWCGL 694

Query 494 RR----------IISLDEFGEAGIAMKKAYRDIRDVDLFPGGLLEPSMPGGVVGETFGHI 543

+ ++++D+ I+ + Y D+DLF GG+ E +PGG+VG TF I

Sbjct 695 PKARHFFTSKDGLVNMDDVTAKKIS--EIYNHPDDIDLFTGGMAELPVPGGIVGPTFACI 752

Query 544 LANQFADLKFGDTYFFLHQQAPQGFRAAQIKAILSVTMSSIICANSAVTQAQPDPFYMAS 603

L QFA LKFGD +F+ + F Q++ + M+ + C N + + F M+

Sbjct 753 LGRQFAALKFGDRFFYENGDEDIRFTEDQLEELRKTDMARLFCDNFNIASIPRNVFLMSD 812

Query 604 QLNLPR-PCSDYSEMDVEPWL 623

P C MD+ W+

Sbjct 813 DTTNPLVDCRTVKSMDLRRWI 833

>[XP_018902871.1](https://www.ncbi.nlm.nih.gov/protein/XP_018902871.1?report=genbank&log$=protalign&blast_rank=55&RID=0) PREDICTED: peroxidase-like [Bemisia tabaci]

Length=948

Score = 305 bits (780), Expect = 3e-88, Method: Compositional matrix adjust.

Identities = 202/604 (33%), Positives = 306/604 (51%), Gaps = 63/604 (10%)

Query 49 RLTYDQLRYRQIDGRCNHPRN--YGSTGRPVKRYLRPHYQDKFGENLPRVYSVTGQLLPS 106

+L +L+YR IDG CN+ N +G G +R L P Y D G PR + G+ LPS

Sbjct 371 KLQCPRLKYRSIDGVCNNLVNGHWGRVGTAYQRVLPPKYTD--GITSPRSNGIDGRELPS 428

Query 107 PRMVSWKLHPDQTAHD-NNTMLVMQMGQFIDHDITRAPELSGRNAS-IKCCG----VPPK 160

R+VS + D D N+T++VMQ GQF+DHD+T P G+ S I CC + +

Sbjct 429 ARLVSSIVAQDADVPDANHTLMVMQWGQFLDHDLTHTPISRGQGGSGIACCRGGKVIDKR 488

Query 161 ERLPDCFPIDIPPGDPVF----EDCMEFFRSSPAVDNDGNIIYPREQINALTSFIDGSAV 216

R PDCFPI IP DPVF E CMEF RS PA + N PREQ+N ++ ++D S +

Sbjct 489 LRHPDCFPIGIPRNDPVFAQFGETCMEFVRSLPAARPECNF-GPREQMNQISGYLDASMI 547

Query 217 YGSDLDTYTWIRSENGTGVFLNTHLVHGRERLPSHPHLGPESCVSSNTAESYCQLAGDMR 276

YGS DT +R G L +V G+E LP +P C SS C AGD R

Sbjct 548 YGSTFDTQQRLRLFRGGR--LRAQVVRGKELLPDNP----SECSSSQRLS--CFQAGDGR 599

Query 277 VNEQPGLGSIHLLFHLHHNHIVRLLVAGILKKRGQPSSPERIAKFIQESSSALKEQIFQE 336

VNEQ L +H ++ HN + + E +F+E

Sbjct 600 VNEQIELALMHTIWMREHNRVAE--------------------ELFNMHPDWSDEALFEE 639

Query 337 VRKMLGAIIQKLTYCDWLPMILGPYLIDKFQLG---CTRRSRYNSDLDPRVANSFLSAAL 393

R+++ A +Q +TY ++LP++LG ++KF+L Y+ +L+ + N F +AA

Sbjct 640 SRRIVIAELQHITYNEFLPIVLGRAYVEKFRLTPREVGHTHNYDPNLNGGITNVFAAAAF 699

Query 394 RFGHTLIPNVYNFGDKRIHLKDTFNIPDASIR----YYDNIIQCLIKE---GSEEAYDRY 446

RFGH+ I ++ K H+++ F + + Y D I ++ S + +DR+

Sbjct 700 RFGHSQIQGNFHGYGKFGHIRENFQLSKQHFQPFVLYADGAIDDFVRGLSFQSSQKFDRF 759

Query 447 VSSAVSEHLFESTRGHKHALDLIAVNIQRGRDHGIPAYHYWRQYYRLRRIISLDEF---- 502

+ V++HLF+ + LDL+A NIQRGRDHG+ Y+ WR+ LRR+ + ++

Sbjct 760 FTREVTDHLFQGN--QRFGLDLVAFNIQRGRDHGLQPYNQWREVCGLRRLRNWNDAESVM 817

Query 503 -GEAGIAMKKAYRDIRDVDLFPGGLLEPSMPGGVVGETFGHILANQFADLKFGDTYFFLH 561

+ + Y I DVDL+ G + E +PG ++G TF ++ +QFA L+ GD +F+

Sbjct 818 DSQTIGRLANVYGSIDDVDLYIGAVSENPLPGAILGPTFVCLVGDQFARLRAGDRFFYEE 877

Query 562 QQAPQGFRAAQIKAILSVTMSSIICANS-AVTQAQPDPFYMASQLNLPRPCS--DYSEMD 618

F AQ+ I +++ ++C NS + QP F+ S LN PC+ + M+

Sbjct 878 GGDLSSFSTAQLNEIRKTSLARVLCDNSDDIVLMQPLAFWKPSYLNKRVPCNSPNIPAMN 937

Query 619 VEPW 622

+ W

Sbjct 938 LRAW 941

>[XP_022240998.1](https://www.ncbi.nlm.nih.gov/protein/XP_022240998.1?report=genbank&log$=protalign&blast_rank=56&RID=0) chorion peroxidase-like [Limulus polyphemus]

Length=726

Score = 299 bits (766), Expect = 5e-88, Method: Compositional matrix adjust.

Identities = 212/617 (34%), Positives = 314/617 (51%), Gaps = 84/617 (14%)

Query 56 RYRQIDGRCNH---PRNYGSTGRPVKRYLRPHYQDKFGENLPRVYSVTGQLLPSPRMVSW 112

++R IDG CN+ PR +GS+ R+L P+Y D G + PR + +G LP+PR++S

Sbjct 142 KFRTIDGTCNNLIKPR-WGSSFNCFNRFLHPNYAD--GLSSPR-RAFSGNELPNPRLIST 197

Query 113 KLHPDQTAHDNN-TMLVMQMGQFIDHDITRAPE---------LSGRNASIKCCGVPPKER 162

LH D + + T LVMQ GQF+DHDIT P L N + CC + +

Sbjct 198 TLHSDSSVLARDFTHLVMQWGQFLDHDITLTPFSSFPGEDLILGNPNRLLDCCALENQHN 257

Query 163 LPDCFPIDIPPGDPVF----EDCMEFFRSSPAVDNDGNIIYPREQINALTSFIDGSAVYG 218

CF IP DP F + CM F RS+ + + REQIN LTSF+DGS +YG

Sbjct 258 -SQCFAFSIPQKDPFFSQFQQRCMNFRRSARCLHSQ---TAQREQINQLTSFVDGSQIYG 313

Query 219 SDLDTYTWIR---------------SENGTGVFLNTHLVHGRERLPSHPHLGPESCVSSN 263

S L+ +R S + G+ G+E LP P + C S

Sbjct 314 SYLNDSLALRTLKHDLPLCYEFYFSSRSLKGLLRTQTDNQGKELLPVTPDTEKDLC-SDK 372

Query 264 TAESYCQLAGDMRVNEQPGLGSIHLLFHLHHNHIVRLLVAGILKKRGQPSSPERIAKFIQ 323

+ C AGD RVNEQPGL S+H ++ HN + R L ++ PS +

Sbjct 373 ESNKICFRAGDERVNEQPGLTSMHTIWLRQHNALARQL------RQLNPSWDD------- 419

Query 324 ESSSALKEQIFQEVRKMLGAIIQKLTYCDWLPMILGPYLIDKFQLGCTR--RSRYNSDLD 381

E+++QE R++L A +Q +TY ++L ++LGP F+L R +RYN + +

Sbjct 420 -------ERLYQEARRILVAQLQMITYNEFLLVVLGPLYHRFFRLKPLRFTYTRYNPNNN 472

Query 382 PRVANSFLSAALRFGHTLIPNVYNFGDKR-----IHLKDTFNIPDASIR-YYDNIIQCLI 435

P V N+F +AA RFGHTLI N +N + + + L++ F P D+ ++ L+

Sbjct 473 PTVMNAFATAAYRFGHTLIQNRFNQVNSKGITSKMVLENNFFFPFGLYEGELDSTLKGLM 532

Query 436 KEGSEEAYDRYVSSAVSEHLFESTRGHKHALDLIAVNIQRGRDHGIPAYHYWRQYYRLRR 495

+ ++ A+D ++S ++ HL+ R K LDLIA NIQRGRDHG+ +Y Y R

Sbjct 533 TQNAQ-AFDTFISKGITNHLYR-LRDEKFGLDLIAFNIQRGRDHGLRSYT---DYVRFCF 587

Query 496 IISLDEFGE--------AGIAMKKAYRDIRDVDLFPGGLLEPSMPGGVVGETFGHILANQ 547

+S+ F + A +K Y +++D+DLF G+ E +PG VVG TFG I+ Q

Sbjct 588 GLSMTRFSQLLWFMPSSARDKFQKLYENVQDIDLFSAGINERPLPGSVVGPTFGCIIGFQ 647

Query 548 FADLKFGDTYFFLHQQAPQGFRAAQIKAILSVTMSSIICANS-AVTQAQPDPFYMASQL- 605

FA LK GD ++F H+ F AQ++ I +++ I+C NS PF S +

Sbjct 648 FALLKKGDRFYFEHKGQAGSFTRAQLREIRKTSLARILCDNSDGFRSVMRLPFRTVSFMG 707

Query 606 NLPRPCSDYSEMDVEPW 622

N C D ++++EPW

Sbjct 708 NRVVQCRDLPQLNLEPW 724

>[XP_034244813.1](https://www.ncbi.nlm.nih.gov/protein/XP_034244813.1?report=genbank&log$=protalign&blast_rank=57&RID=0) peroxidase-like [Thrips palmi]

Length=952

Score = 304 bits (779), Expect = 5e-88, Method: Compositional matrix adjust.

Identities = 200/598 (33%), Positives = 318/598 (53%), Gaps = 66/598 (11%)

Query 56 RYRQIDGRCNHPRN--YGSTGRPVKRYLRPHYQDKFGENLPRVYSVTGQLLPSPRMVSWK 113

+YR DG CN+ N +GSTG ++R L P Y D G N PR + G LPS R+VS +

Sbjct 382 KYRTADGTCNNVVNSRWGSTGSALQRLLPPKYGD--GVNSPRAQA-NGAPLPSARLVSNR 438

Query 114 LHPDQ-TAHDNNTMLVMQMGQFIDHDITRAPELSGRNA-SIKCCG----VPPKERLPDCF 167

L + DN T+++MQ GQF+DHD+T P G+N I CC P+ PDCF

Sbjct 439 LAQESDRPSDNVTLILMQWGQFLDHDLTHTPISRGQNGVGISCCQDGQVSDPRVSHPDCF 498

Query 168 PIDIPPGD----PVFEDCMEFFRSSPAVDNDGNIIYPREQINALTSFIDGSAVYGSDLDT 223

I IP D P E CMEF RS PA + N PREQ+N +T ++DGS +YGSD +

Sbjct 499 AIPIPRDDRFLAPFGERCMEFVRSLPAPRPECNF-GPREQMNQITGYMDGSNIYGSDANK 557

Query 224 YTWIRSENGTGVFLNTHLVHGRERLPSHPHLGPESCVSSNTAESYCQLAGDMRVNEQPGL 283

+R G L V GR LP++P+ C +T+ + C ++GD RVNEQP L

Sbjct 558 QQQLRLFRGGR--LREQNVRGRSMLPANPN----EC--QDTSGAACFVSGDGRVNEQPDL 609

Query 284 GSIHLLFHLHHNHIVRLLVAGILKKRGQPSSPERIAKFIQESSSALKEQIFQEVRKMLGA 343

+H ++ HN + L Q P+ E++FQE R+++ A

Sbjct 610 ALMHTVWLREHNRVANQL---------QALHPQWT-----------DEEVFQEARRIVVA 649

Query 344 IIQKLTYCDWLPMILGPYLIDKFQLG---CTRRSRYNSDLDPRVANSFLSAALRFGHTLI 400

+Q +TY ++LP++LG +D+ ++ + Y+ +L+ + N+F +AA RFGHTL+

Sbjct 650 EMQHITYNEFLPIVLGKPYMDRAEMSPKDSGYTALYDRELNGGITNAFATAAFRFGHTLL 709

Query 401 PNVYNFGDKRIHLKDTFNIPDASIRYY--------DNIIQCLIKEGSEEAYDRYVSSAVS 452

+ + +++ F + ++ + + D++++ L + S++ +DR+ + V+

Sbjct 710 VSNLQGVGRFGNVRKNFPLSKSAFKPFMLYEEEGIDDMVRGLTTQSSQK-FDRFFTKEVT 768

Query 453 EHLFESTRGHKHALDLIAVNIQRGRDHGIPAYHYWRQYYRLRRIISLDEFG-----EAGI 507

HLF++ LDL+A+N+QRGRDHG+P Y WRQ LRR S ++ ++

Sbjct 769 NHLFQND--LPFGLDLVALNLQRGRDHGLPGYTEWRQVCGLRRPRSWEDLQGIMEPDSVA 826

Query 508 AMKKAYRDIRDVDLFPGGLLEPSMPGGVVGETFGHILANQFADLKFGDTYFFLHQQAPQG 567

++ Y + +VDLF G+ E + PG ++G TF I+ +QF L+ GD +F+ P

Sbjct 827 VLQSLYPSVDEVDLFAAGVSEKAAPGALLGPTFTCIVGDQFGRLRRGDRFFYEEGNQPSS 886

Query 568 FRAAQIKAILSVTMSSIICANS-AVTQAQPDPFYMASQLNLPRPCSDYS--EMDVEPW 622

F+ Q++ I +++ I+C NS + QP F+ AS +N CS + ++D+ W

Sbjct 887 FKPEQLQQIRKASLARILCDNSDNIALMQPLAFFHASFVNQRVACSSDTIPKVDIRAW 944

>[XP_011505800.1](https://www.ncbi.nlm.nih.gov/protein/XP_011505800.1?report=genbank&log$=protalign&blast_rank=58&RID=0) PREDICTED: peroxidase-like [Ceratosolen solmsi marchali]

Length=871

Score = 302 bits (774), Expect = 5e-88, Method: Compositional matrix adjust.

Identities = 201/612 (33%), Positives = 312/612 (51%), Gaps = 82/612 (13%)

Query 57 YRQIDGRCNHPRNYGSTGRPVKRYLRPHYQDKFGENLPRVYSVTGQLLPSPRMVSWKLHP 116

YR DG CNHP G P R L P Y D G +PRV S+ G LPS R +S ++HP

Sbjct 215 YRTFDGSCNHPLQLGRAFTPYSRVLPPDYAD--GIEIPRV-SLFGNPLPSARKISLQVHP 271

Query 117 -DQTAHDNNTMLVMQMGQFIDHDITRAPELSGRN-ASIKCCGVPPKERLPDCFPIDIPPG 174

+ + + T+++ GQF+DHDIT G N +S+ CC PP P+CFP+ + PG

Sbjct 272 PSPSINPSFTVMLAVFGQFLDHDITATAISQGTNGSSLSCC--PPSGDHPECFPVKVGPG 329

Query 175 DPVFE----DCMEFFRSSPAVDNDGNIIYPREQINALTSFIDGSAVYGSDLDTYTWIRSE 230

DPV++ CMEF RS+PA I PR+Q+N +T+FIDGS +YGSD+DT +R

Sbjct 330 DPVYDIAGSSCMEFVRSAPAA---LCTIGPRQQLNQVTAFIDGSVIYGSDVDTAKSLREF 386

Query 231 NGTGVFLNTHLVHGRERL---PSHPHLGPESCVSSNTAES--------YCQLAGDMRVNE 279

+G GR R+ P L P S +++ YC GD R NE

Sbjct 387 SG-----------GRLRMQITPDKRSLLPASRNANDGCNRQTEFVRGRYCFATGDARANE 435

Query 280 QPGLGSIHLLFHLHHNHIVRLLVAGILKKRGQPSSPERIAKFIQESSSALKEQIFQEVRK 339

L ++HLL+ HN ++A L K + E+I+QE R+

Sbjct 436 NLHLTTMHLLWARQHN-----VLANELTKLNPMWN---------------DEKIYQESRR 475

Query 340 MLGAIIQKLTYCDWLPMILGPYLIDKFQLGCTRRS----RYNSDLDPRVANSFLSAALRF 395

++GA +Q +TY ++LP++LG I + L + NSD++P +AN F SAA RF

Sbjct 476 IVGAQLQHITYNEFLPIVLGETEISRRNLKPLSKGFKLLNNNSDVNPAIANHFASAAFRF 535

Query 396 GHTLIPNVYNFGDKRIHLKDTFN-------IPDASIRYYDNIIQCLIKEGSEEAYDR--- 445

HTL+P + DK+ K TF+ + + Y + ++ + + +

Sbjct 536 AHTLLPGLMKVTDKQ---KGTFSYIQLHKILFNPYSLYNEGGVENSVSTATSNLIQKTST 592

Query 446 YVSSAVSEHLFESTRGHKH---ALDLIAVNIQRGRDHGIPAYHYWRQYYRLRRIISLDEF 502

+V+S +++HLF+ ++ LDL+++NIQRGRDHG+P + WR+Y LR+I++ ++

Sbjct 593 HVTSQLTKHLFQDKMVNQTLPCGLDLVSLNIQRGRDHGLPGFTKWREYCGLRKILNFEDL 652

Query 503 GE-----AGIAMKKAYRDIRDVDLFPGGLLEPSMPGGVVGETFGHILANQFADLKFGDTY 557

E A + K Y ++ D+DL+ G L E G++G TF ++A+QF L+ GD Y

Sbjct 653 KEEMDVEALDEISKLYNNVDDIDLYTGALAELPKSDGLIGPTFTCLIADQFERLQVGDKY 712

Query 558 FFLHQQAPQGFRAAQIKAILSVTMSSIICANS-AVTQAQPDPFYMASQLNLPRPCSDYSE 616

++ F Q+K + ++++ IIC S +T+ Q N C D

Sbjct 713 WYESANQSGSFNEDQLKELRKISLARIICETSDNITEIQAQVMRSIGPNNPIISCEDIPS 772

Query 617 MDVEPWLIHFSD 628

+ ++PW I ++

Sbjct 773 VSLDPWKIETTN 784

>[XP_025096592.1](https://www.ncbi.nlm.nih.gov/protein/XP_025096592.1?report=genbank&log$=protalign&blast_rank=59&RID=0) chorion peroxidase-like [Pomacea canaliculata]

Length=955

Score = 304 bits (778), Expect = 7e-88, Method: Compositional matrix adjust.

Identities = 204/603 (34%), Positives = 305/603 (51%), Gaps = 67/603 (11%)

Query 54 QLRYRQIDGRCNHPRN--YGSTGRPVKRYLRPHYQDKFGENLPRVYSV-TGQLLPSPRMV 110

+L++R DG CN+ + +GS P++R L+P Y D G + PR S + LPS R +

Sbjct 216 ELKFRTPDGTCNNLVHNLWGSALSPLRRILQPQYGD--GVSAPRTTSTHSRNPLPSARAI 273

Query 111 SWKLHPDQTAH--DNNTMLVMQMGQFIDHDITRAP-ELSGRNASIKCCG--VPPKERL-- 163

S +HP + + T +VMQ GQ++DHDIT P + + +++ CC PP

Sbjct 274 SVLIHPSGPGNLMTSLTHMVMQWGQYLDHDITSTPIQTAADGSALTCCDDEAPPGSNTRF 333

Query 164 -----PDCFPIDIPPGDPVFE--DCMEFFRSSPAVDNDGNIIYPREQINALTSFIDGSAV 216

CFPI IP DP F+ C F RS +++ + P EQ+N LT+++D S V

Sbjct 334 SVSNRTACFPIPIPSYDPYFKGRSCFSFTRSLQVTNSNCKQV-PVEQLNQLTAYVDASQV 392

Query 217 YGSDLDTYTWIRSENGTGVFLNTHLVHGRERLPSHPHLGPESCVSSNTAESYCQLAGDMR 276

YGS + +R+ G + G + LP ESCV +N YC AGD+R

Sbjct 393 YGSSAEEQKNLRTFKGGQM-----TTSGNDLLPKDKA---ESCVLNNPNRDYCFKAGDLR 444

Query 277 VNEQPGLGSIHLLFHLHHNHIVRLLVAGILKKRGQPSSPERIAKFIQESSSALKEQIFQE 336

VNEQ GL S+H ++ HN +V+ + A + E++FQE

Sbjct 445 VNEQMGLASMHTVWMREHNRLVQKMAA--------------------VNPHWNDERLFQE 484

Query 337 VRKMLGAIIQKLTYCDWLPMIL--GPYLIDKFQL--GCTRRSRYNSDLDPRVANSFLSAA 392

RK++GA+ QK+TY DWLP++L + + QL + + YN +D V N F SAA

Sbjct 485 TRKIVGALYQKITYGDWLPIVLDRNRFAANNLQLLPSGQQSNIYNPSVDAGVKNVFASAA 544

Query 393 LRFGHTLIPNVYNFGDKRIH------LKDTFNIPDASIRYYDNIIQCLIKEGSEEA---Y 443

RFGH+LI + + + H L+ F I + I+ + +A

Sbjct 545 FRFGHSLIRSTMSQMTEEYHEAGEVRLQYVFGNTSQIIEGQGLGVTMYIRGMTRDAPNVV 604

Query 444 DRYVSSAVSEHLFESTRGHKHALDLIAVNIQRGRDHGIPAYHYWRQYYRLRRIISLDEFG 503

DR+ + + HLF+ RG+ +LDL+A NIQRGRDHG+P Y+ WR+Y +L + S

Sbjct 605 DRFFTEEIKNHLFQDKRGN--SLDLVAFNIQRGRDHGLPPYNEWRKYCKLPVMTSFSNMP 662

Query 504 E----AGIAMKKAYRDIRDVDLFPGGLLEPSMPGGVVGETFGHILANQFADLKFGDTYFF 559

+ G A + Y D+DLF G + E G+VG TF IL+ QF +LKFGD ++F

Sbjct 663 DHDELTGQAFSQIYEHPDDIDLFSGAISERPEGNGLVGPTFACILSEQFRNLKFGDRFWF 722

Query 560 LHQQAPQGFRAAQIKAILSVTMSSIICANSAVTQAQPDPFYMASQLNLPRPCSDYSEMDV 619

+ GF Q++ I +++ +ICAN+ Q +PF S N C+D +M +

Sbjct 723 ETRDPVTGFSLEQLEEIRRHSLARVICANTGGGHIQGNPFVQPSDSNPMVNCNDLPDMTM 782

Query 620 EPW 622

EPW

Sbjct 783 EPW 785

>[XP_025078500.1](https://www.ncbi.nlm.nih.gov/protein/XP_025078500.1?report=genbank&log$=protalign&blast_rank=60&RID=0) chorion peroxidase-like [Pomacea canaliculata]

Length=623

Score = 296 bits (757), Expect = 8e-88, Method: Compositional matrix adjust.

Identities = 208/606 (34%), Positives = 315/606 (52%), Gaps = 80/606 (13%)

Query 48 SRLTYDQLRYRQIDGRCNHPRN--YGSTGRPVKRYLRPHYQDKFGENLPRVYSV-TGQLL 104

SR + RY +DG CN+ ++ +G+T +P R L Y D G+ +PRVYS +G+ L

Sbjct 67 SRDCNTRTRYYSLDGWCNNLQHHQWGATFQPFVRLLPNQYSD--GKVVPRVYSEGSGEPL 124

Query 105 PSPRMVSWKLHP---DQTAHDNN---TMLVMQMGQFIDHDITRAPELSGRNASIKCCGVP 158

S R+VS + P ++T D + + ++MQ GQF HD P L G + C G+

Sbjct 125 TSTRLVSTECMPCVRNETMEDVSRIHSQMLMQFGQFQSHDTGATPALEGADC---CKGLN 181

Query 159 PKERLPD------CFPIDIPPGDPVFEDCMEFFRSSPAVDNDGNIIYPREQINALTSFID 212

PD CFPI +P D F C+ F RS +D +G +QIN+ TSF+D

Sbjct 182 KDGFHPDMYNRGPCFPIPVPEHDRYFNRCIPFTRSLFVLDENG----VAQQINSATSFVD 237

Query 213 GSAVYGSDLDTYTWIRSENGTGVFLNTHL-VHGRERLPSHPHLGPESCVSSNTAESYCQL 271

S VYGS L ++ N + HL + GR LP + C+ +N S C L

Sbjct 238 ASVVYGSSL------KASNALRAWTRGHLKLSGRNMLPLSKK---QKCMVTNHKNS-CPL 287

Query 272 AGDMRVNEQPGLGSIHLLFHLHHNHIVRLLVAGILKKRGQPSSPERIAKFIQESSSALKE 331

AGDMRVN PGL ++HLL+H HN + A +L+ R ER+

Sbjct 288 AGDMRVNVFPGLTAMHLLWHREHNRL-----ADMLRSRHDDWDDERL------------- 329

Query 332 QIFQEVRKMLGAIIQKLTYCDWLPMILGPYLIDKFQLGCTRRSRYNSDLDPRVANSFLSA 391

FQE R++L A +Q + Y ++LP+ILGP++ + F+L ++YN +L+P NSF +

Sbjct 330 --FQEARRILIAEMQSVVYGEYLPIILGPWVTNFFRLNSP--AKYNPNLNPTTINSFTTT 385

Query 392 ALRFGHTLIPNVY--NFGDKRIHLKDTFNIPDASIRYYDNIIQ----CLIKEGSEEAYDR 445

A RFGH+ I N + G + +K + PD + + CL+ + +++ DR

Sbjct 386 AYRFGHSQINNEFLVQHGGFAVPIKSMYFRPDLVYNQGEETLTGLLICLLNKAGQKSDDR 445

Query 446 YVSSAVSEHLFESTRGHKHALDLIAVNIQRGRDHGIPAYHYWRQYYRLRRIISLDEFGEA 505

+ S VS+ LF + DL+A N+QRGRDHG+P Y+ QY I ++ FG +

Sbjct 446 F-SEGVSDFLFFGVDRPGASSDLMARNMQRGRDHGLPPYY---QYM----IQRMEAFGVS 497

Query 506 GI--------AMKKAYRDIRDVDLFPGGLLEPSMPGGVVGETFGHILANQFADLKFGDTY 557

+ K YR +RDVDLF GG+ E + GG VG TF +I+ QF + + GD +

Sbjct 498 RFRSRLRLSSCVDKVYRSLRDVDLFLGGMEEKLVRGGEVGPTFAYIIGKQFQNFREGDRF 557

Query 558 FFLHQQAPQGFRAAQIKAILSVTMSSIICANSAVTQAQPDPFYMASQLNLPR-PCSDYSE 616

F+ Q + GF AQI++I ++T++++IC NS +T+ Q F++ + P C D +

Sbjct 558 FYDIQGSAAGFTEAQIRSIKTITIANLICRNSEITRIQKKAFFLPNVKRNPEVDCKDIPD 617

Query 617 MDVEPW 622

+D W

Sbjct 618 LDFTLW 623

>[XP_021371886.1](https://www.ncbi.nlm.nih.gov/protein/XP_021371886.1?report=genbank&log$=protalign&blast_rank=61&RID=0) uncharacterized protein LOC110462299 [Mizuhopecten yessoensis]

Length=1265

Score = 308 bits (789), Expect = 8e-88, Method: Compositional matrix adjust.

Identities = 199/577 (34%), Positives = 296/577 (51%), Gaps = 66/577 (11%)

Query 74 GRPVKRYLRPHYQDKFGENLPRVYSVTGQLLPSPRMVSWKLHPDQTAHDNNTMLVMQM-- 131

G P +R L P Y+D G + PR+ + G +LPS R+VS LH H + + VM M

Sbjct 724 GSPHRRLLPPDYED--GIDSPRIRADNGAILPSARLVSNTLHNAGRTHTSESFTVMYMSF 781

Query 132 GQFIDHDITRAPELSGRNAS-IKCCGVPPKERLPDCFPIDIPPGDPVF-EDCMEFFRSSP 189

GQF+DHD+T L N + I CC CFPI IP D F + CM F RS+

Sbjct 782 GQFLDHDLTSTASLKDENNNPIDCCSNSNAALSSACFPIKIPINDTRFTKSCMSFTRSAA 841

Query 190 AVDNDGNIIYPREQINALTSFIDGSAVYGSDLDTYTWIRSENGTGVFLNTHLVHGRERLP 249

AV N + Y R+QIN LTS++D S VYGS L++ +R ++G GR R+

Sbjct 842 AVKNGCDPDY-RQQINQLTSYLDASNVYGSTLESQERLREKSG-----------GRMRVS 889

Query 250 SHPHLGP----ESCVSSNTAESYCQLAGDMRVNEQPGLGSIHLLFHLHHNHIVRLLVAGI 305

L P +C+ +N ++++C AGD R +E P L ++H+ F HN I L

Sbjct 890 DQGDLLPSYKNNTCILTNPSKTHCFDAGDERNSEVPTLTTLHIAFLREHNRIANTL---- 945

Query 306 LKKRGQPSSPERIAKFIQESSSALKEQIFQEVRKMLGAIIQKLTYCDWLPMILGPYLIDK 365

+ I + E +FQEVRK++GA++Q + Y ++LP ++GP +D

Sbjct 946 --------------RLINRDWN--DELVFQEVRKIVGALLQHIAYNEYLPKVIGPSFMDM 989

Query 366 FQLGCTR---RSRYNSDLDPRVANSFLSAALRFGHTLIPNVYNFGDKRIHLKDTFNI--- 419

+ L T RS YN +D V N F +AA RFGH+ IP+ +KR K N+

Sbjct 990 YDLKPTPTGFRSVYNDTIDATVTNVFATAAFRFGHSQIPDKMMLINKRFKKKAIMNLEKQ 1049

Query 420 ---PDASIRYYDNIIQCLIK---EGSEEAYDRYVSSAVSEHLFESTRGHKHALDLIAVNI 473

PD + + N + L + ++ D + S+V LF ++ +LDL A+N+

Sbjct 1050 YFKPD--LMFEKNGPEWLARWQVSYAQTKEDNSIQSSVRNFLFLDSK--NDSLDLAALNM 1105

Query 474 QRGRDHGIPAYHYWRQYYRL--------RRIISLDEFGEAGIAMKKAYRDIRDVDLFPGG 525

QRGRDHG+P+Y+ WR + L R+ ++ + + + Y D+DL+ GG

Sbjct 1106 QRGRDHGLPSYNAWRSWCGLNPASDFSHRKSGLVNHYRTGRNRLSRIYSSPNDIDLYSGG 1165

Query 526 LLEPSMPGGVVGETFGHILANQFADLKFGDTYFFLHQQAPQGFRAAQIKAILSVTMSSII 585

LLE +PG +VG TF I++ QF DL+ GD +++ + GF AQ+ I +T+SSI+

Sbjct 1166 LLEKHLPGALVGPTFACIISRQFRDLQKGDRFWYENDAPYTGFSEAQLNEIKKMTLSSIL 1225

Query 586 CANSAVTQAQPDPFYMASQLNLPRPCSDYSEMDVEPW 622

C N + + Q D F M S N R C+ MD+ W

Sbjct 1226 CTNLHLVKTQLDSFQMKSDTNQRRECNSLPTMDLTKW 1262

Score = 278 bits (710), Expect = 6e-77, Method: Compositional matrix adjust.

Identities = 196/599 (33%), Positives = 293/599 (49%), Gaps = 65/599 (11%)

Query 54 QLRYRQIDGRCNHPRNY--GSTGRPVKRYLRPHYQDKFGENLPRVYSVTGQLLPSPRMVS 111

Q YR +G CNH ++ G+ +R + Y+D G + PRV S + + LPS R+VS

Sbjct 158 QYPYRTANGYCNHLQDTTQGAALTRQRRMMLNSYED--GIDYPRVRSKSDRTLPSARLVS 215

Query 112 WKLHPDQT---AHDNNTMLVMQMGQFIDHDITRAPELSGRN-ASIKCCGVPPK--ERLPD 165

+H A T+L+MQ GQFI+HD+ P +SG + A I CCG P +R

Sbjct 216 NIMHKAGECPLASKQFTVLIMQFGQFIEHDVISTPMISGTDGADIMCCGGPQNVTDRRAA 275

Query 166 CFPIDIPPGDPVF-EDCMEFFRSSPAVDNDGNIIYPREQINALTSFIDGSAVYGSDLDTY 224

CFPI IPPGD F E CM F RS+P + + ++ R +N ++F+DGS +YG+ D

Sbjct 276 CFPISIPPGDGRFSESCMTFVRSTPGLKLNCDMGI-RSPMNQASAFMDGSQIYGTSEDET 334

Query 225 TWIRSENGTGVFLNTHLVHGRERLPSHPHLGPESCVSSNTAESYCQLAGDMRVNEQPGLG 284

+RS FL L LP P E C+ + YC LAGD RVN PGL

Sbjct 335 KGLRS------FLGGMLKMTTLGLP--PPSEEEMCIKEAPGD-YCMLAGDFRVNHVPGLT 385

Query 285 SIHLLFHLHHNHIVRLLVAGILKKRGQPSSPERIAKFIQESSSALKEQIFQEVRKMLGAI 344

+H F HN I + F + E+ FQE RK++ A+

Sbjct 386 VLHTTFLREHNRIA--------------------SHFTLINPGWNDERTFQETRKLIIAM 425

Query 345 IQKLTYCDWLPMILGPYLIDKFQLGCTRR---SRYNSDLDPRVANSFLSAALRFGHTLIP 401

+Q + + + LP IL + ++ + + + + Y+ D D + F +AA+RF HT IP

Sbjct 426 LQHMVFNELLPSILNEDHVIRYDIRSSTQGFSATYDPDTDASIMMGFSAAAMRFPHTRIP 485

Query 402 NVY-----NFGDKRIHLK-DTFNIPDASI----RYYDNIIQCLIKEGSEEAYDRYVSSAV 451

+V +F +R +L TF+ P + + ++ + LI E DR+V V

Sbjct 486 DVQGMVDDSFSTQRNNLIFATFDKPRFVLERMGQALNDFARWLISFPVMED-DRFVQDGV 544

Query 452 SEHLFESTRGHKHALDLIAVNIQRGRDHGIPAYHYWRQ--------YYRLRRIISLDEFG 503

+ LF RGH + DL+A+NIQR RD GIP Y+ WR+ Y+ +D

Sbjct 545 RDFLFLDNRGH--SFDLVALNIQRARDQGIPTYNEWRKLCGLVPATYFASGPGGLVDHEP 602

Query 504 EAGIAMKKAYRDIRDVDLFPGGLLEPSMPGGVVGETFGHILANQFADLKFGDTYFFLHQQ 563

E + Y D+ D+DLF GGL E ++PG G TF I+A QF LK GD +++ ++

Sbjct 603 EVVRLLSTVYSDVDDIDLFTGGLSEITLPGAATGPTFACIIATQFRSLKVGDRFWYENKH 662

Query 564 APQGFRAAQIKAILSVTMSSIICANSAVTQAQPDPFYMASQLNLPRPCSDYSEMDVEPW 622

GF Q+ I + +S ++C N QP+ F N PC + +++++ W

Sbjct 663 PITGFTEEQLNEIKKIRLSKVMCNNLDTPFIQPNVFRFVGNGNARIPCGELPDINLDAW 721

>[XP_019879548.1](https://www.ncbi.nlm.nih.gov/protein/XP_019879548.1?report=genbank&log$=protalign&blast_rank=62&RID=0) PREDICTED: LOW QUALITY PROTEIN: chorion peroxidase-like [Aethina

tumida]

Length=1269

Score = 308 bits (789), Expect = 1e-87, Method: Compositional matrix adjust.

Identities = 215/646 (33%), Positives = 337/646 (52%), Gaps = 76/646 (12%)

Query 18 VYPDIYARRGDLGDSTGSLDFVSEADLEHCSRLTYDQLRYRQIDGRCNHPRN--YGSTGR 75

YP+ R +S S + E L R + RYR DG CN+PR G+

Sbjct 140 TYPENVGRDAGFPESVQSRLLLDECPLRGLPRCSQASKRYRTADGTCNNPRQPWRGAAML 199

Query 76 PVKRYLRPHYQDKFGENLPRVYSVTGQLLPSPRMVSWKLHPDQTAH-DNNTMLVMQMGQF 134

P++R++ P YQD +++ R S+ LPSPR +S ++H D+ + T+++MQ GQF

Sbjct 200 PLQRFMPPVYQDGI-QSIRR--SIFDNRLPSPRTISTRVHRDKNHEIQSVTLMLMQWGQF 256

Query 135 IDHDITRAPELSGRNASI-KCCG------VPPKERLPDCFPIDIPPGDPVFED----CME 183

IDHD+T + N SI +CC +PP+ P C PI++P D + C+E

Sbjct 257 IDHDVTSVVKSRSFNGSIPRCCDRGGRGFLPPELMHPACLPIEVPDDDWFYSRFRIRCLE 316

Query 184 FFRSSPAVDNDGNIIYPREQINALTSFIDGSAVYGSDLDTYTWIRSENGTGVFLNTHLVH 243

F RS+P+ D ++ + REQ+N +T FID S +YGSD++T +R+ F N L++

Sbjct 317 FLRSAPSTRIDCDLGW-REQLNQVTPFIDASPIYGSDIETSDSVRT------FRNGKLIY 369

Query 244 GRER--LPSHPHLGP--ESCVSSNTAESYCQLAGDMRVNEQPGLGSIHLLFHLHHNHIVR 299

GR R P +P P E C S S C GD RV+EQPGL ++H ++ +HN I

Sbjct 370 GRSRNQRPLNPPDPPGGEIC-RSGALSSDCFQPGDGRVDEQPGLTAMHTVWVRYHNKI-- 426

Query 300 LLVAGILKKRGQPSSPERIAKFIQESSSALKEQIFQEVRKMLGAIIQKLTYCDWLPMILG 359

A +L K +S E++FQE RK++ ++IQ +TY ++LP+ILG

Sbjct 427 ---ATVLSKL---------------NSHWSDEKVFQETRKIVYSVIQHITYREFLPIILG 468

Query 360 PYLIDKFQLGCTRR---SRYNSDLDPRVANSFLSAALRFGHTLIPNVYN---------FG 407

+I+ F+L R+ + Y+ ++P+VAN+F +AA RFGH+++ N + F

Sbjct 469 QDVIEIFELNLMRKGYYTGYDVRINPQVANAFSAAAYRFGHSMVQNSFIRFNSQHRPLFN 528

Query 408 DKRIH--LKDTFNIPDASIRYYDNIIQCLIKEGSEEAYDRYVSSAVSEHLFESTRGHKHA 465

+ +H L + NI S+ D +I L+ + ++ D +V+ +S HLF+ +

Sbjct 529 NVTLHEDLDNEENI--WSLGSVDRLILGLLNQPAQRR-DEFVADELSNHLFQFS-DSPFG 584

Query 466 LDLIAVNIQRGRDHGIPAYHYWRQYYRLRRIISLDEFG-----EAGIAMKKAYRDIRDVD 520

+DL A+NIQRGRDHG+P Y WR+ L + S + E + Y + D+D

Sbjct 585 MDLSAINIQRGRDHGVPPYTSWREPCGLTAVKSWKDLESIMSFETIQRFRSLYEHVDDLD 644

Query 521 LFPGGLLEPSMPGGVVGETFGHILANQFADLKFGDTYFFLHQQAPQGFRAAQIKAILSVT 580

L+ GGL E + GG+VG TF I+A QF +L+ GD +++ + F AQ++ I VT

Sbjct 645 LYSGGLAEKPLRGGIVGPTFACIIAQQFVNLRKGDRFWYENGDMDSSFTPAQLQQIRHVT 704

Query 581 MSSIICAN-SAVTQAQPDPFYMASQL-NLPRPCSD--YSEMDVEPW 622

++ I+C + QP F N+ PC+ + D+ PW

Sbjct 705 LAQILCQTMEGIETIQPFVFLSHDNFRNVRLPCNSPLINNFDLSPW 750

>[KAF2367819.1](https://www.ncbi.nlm.nih.gov/protein/KAF2367819.1?report=genbank&log$=protalign&blast_rank=63&RID=0) hypothetical protein FHG87_001411 [Trinorchestia longiramus]

Length=780

Score = 299 bits (765), Expect = 2e-87, Method: Compositional matrix adjust.

Identities = 211/601 (35%), Positives = 309/601 (51%), Gaps = 69/601 (11%)

Query 56 RYRQIDGRCNHPRN--YGSTGRPVKRYLRPHYQDKFGENLPRVYSVTGQLLPSPRMVSWK 113

+YR +DG CN+ N +G G R L P Y D G + R S G LPS R +S +

Sbjct 213 KYRSVDGACNNLNNPDWGQAGTTFVRLLPPVYSD--GVSNIRT-STDGSPLPSARTLSAE 269

Query 114 L-HPDQTAHDNNTMLVMQMGQFIDHDITRAPELSGRNAS-IKCCGV----PPKERLPDCF 167

+ + +D+ T+LVMQ GQF+DHDIT P G + S I CC P +E PDC

Sbjct 270 IISENNNVYDDYTLLVMQWGQFLDHDITHTPITKGMDDSVISCCNQGNFRPRQELHPDCL 329

Query 168 PIDIPPGDPVF----EDCMEFFRSSPAVDNDGNIIYPREQINALTSFIDGSAVYGSDLDT 223

PI+I P D + + CM F RS PA+ G PREQ+N +TSFID S VYGS +

Sbjct 330 PIEISPTDRFYSRFGQRCMNFVRSMPAM-RRGCTFGPREQMNQITSFIDASNVYGSTTEE 388

Query 224 YTWIRSENGTGVFLNTHLVHGRERLPSHPHLGPESCVSSNTAESYCQLAGDMRVNEQPGL 283

+R NG L R+ LP P+ E C S T + C AGD RVNEQP L

Sbjct 389 MRNLREFNGG--LLKITQRGRRQLLP--PNHSDEEC-ESPTIDKPCFAAGDTRVNEQPNL 443

Query 284 GSIHLLFHLHHNHIVRLLVAGILKKRGQPSSPERIAKFIQESSSALKEQIFQEVRKMLGA 343

+H ++ HN I + L +L E +F E R+++GA

Sbjct 444 TVLHTVWMRQHNLIAQQL--AVLNPHWN------------------DETLFLETRRIVGA 483

Query 344 IIQKLTYCDWLPMILGPYLIDKFQLGCTRR---SRYNSDLDPRVANSFLSAALRFGHTLI 400

++Q +TY ++LP++LG + ++ F L + + Y+ ++DP V+++F +AA R+GHTLI

Sbjct 484 MMQHITYNEYLPIVLGRHFVETFGLVPLKEGYANTYDPNVDPSVSSAFATAAFRYGHTLI 543

Query 401 -PNVYNF--------GDK--RIHLKDTFNIPDASIRYYDNIIQCLIKEGSEEAYDRYVSS 449

NV + G+K R+ F + + +D++I+ L + S++ +D Y S

Sbjct 544 DSNVQGYSRFGTQEIGEKLRRLQFSPFFLYEEGA---HDSLIRGLAIQPSQQ-FDNYFSE 599

Query 450 AVSEHLFESTRGHKHALDLIAVNIQRGRDHGIPAYHYWRQYYRLRRIISLDEFG-----E 504

++ HLF R + +DL+A+N+QRGRDHG+P Y WRQ+ L I S + +

Sbjct 600 ELTNHLF--ARNNSFGMDLVALNLQRGRDHGLPPYVQWRQFCNLPPIRSFGQLADVMPPD 657

Query 505 AGIAMKKAYRDIRDVDLFPGGLLEPSMPGGVVGETFGHILANQFADLKFGDTYFFLHQQA 564

A + YRD+ D+DLF GG+LE + G +VG TF IL +QF LK GD YF+ +

Sbjct 658 ASRYLSLLYRDVEDIDLFLGGILEFPVAGALVGHTFLCILGDQFYRLKAGDRYFYENGGT 717

Query 565 PQGFRAAQIKAILSVTMSSIICANS-AVTQAQPDPFYMASQLNLPRPCSD--YSEMDVEP 621

F AQ+ I V++ ++C S + QP F A N C + M+++

Sbjct 718 HMAFNPAQLHEIRKVSLGRVLCDTSDNIAVMQPLAFLHAKFTNKRTSCQEDGIPRMNLQA 777

Query 622 W 622

W

Sbjct 778 W 778

>[EEC15885.1](https://www.ncbi.nlm.nih.gov/protein/EEC15885.1?report=genbank&log$=protalign&blast_rank=64&RID=0) peroxinectin, putative [Ixodes scapularis]

Length=614

Score = 295 bits (754), Expect = 2e-87, Method: Compositional matrix adjust.

Identities = 222/627 (35%), Positives = 313/627 (50%), Gaps = 93/627 (15%)

Query 45 EHCSRLTYDQLR---YRQIDGRCNH--PRNYGSTGRPVKRYLRPHYQDKFGENLPRVYSV 99

E C RL+ R +R++DG CN+ ++G ++R L P Y D G + PR+ S

Sbjct 27 EQCHRLSNGICRPGPFREMDGSCNNLDHSDWGVAFSCMRRLLPPRYAD--GVSAPRI-SE 83

Query 100 TGQLLPSPRMVSWKLHPD------QTAHDNNTMLVMQMGQFIDHDITRAP---------E 144

TG LP+PR+VS +H D +T+H ++MQ GQF+DHD AP +

Sbjct 84 TGGELPNPRLVSTTVHVDFDRPSRETSH-----MLMQWGQFLDHDFALAPISSIPGEIID 138

Query 145 LSGRNASIKCCGVPPKERLPDCFPIDIPPGDPVF----EDCMEFFRSS--PAVDNDGNII 198

L N I CC P P CF DIPP D F E CM F RS+ P +

Sbjct 139 LGNPNDVIDCCS-PETRSSPRCFSFDIPPTDHFFGKYGEHCMNFPRSARCPLCS-----L 192

Query 199 YPREQINALTSFIDGSAVYGSDLDTYTWIRSENGTGVFLNTHLVHGRERLPS--HPHLGP 256

PR+QI++LTSF+DGS VYGS L+ +R+ G G G LP+ HPH

Sbjct 193 GPRQQIDSLTSFVDGSQVYGSSLEDSLKLRTLQGDGRLKFDVGRRGDMILPASFHPH--- 249

Query 257 ESCVSSNTAESYCQLAGDMRVNEQPGLGSIHLLFHLHHNHIVRLLVAGILKKRGQPSSPE 316

E S C AGD RVNEQPGL ++H L+ HN VAG L E

Sbjct 250 EDQCSRPEHGDLCFRAGDERVNEQPGLTAMHTLWLRQHN-----FVAGKLAGLNPHWDDE 304

Query 317 RIAKFIQESSSALKEQIFQEVRKMLGAIIQKLTYCDWLPMILGPYLIDKFQLGCT--RRS 374

RI FQE R+++ +Q +TY ++LP+++G +F L +

Sbjct 305 RI---------------FQEARRIVIGQMQMITYDEFLPLVVGKSFHREFGLEVLPYGYT 349

Query 375 RYNSDLDPRVANSFLSAALRFGHTLIPNVYNFGDKR-----IHLKDTFNIPDASIRYYDN 429

YN +DP + N F AA RFGHT++ + D R + L+D F P + D

Sbjct 350 TYNKQIDPSILNEFAGAAYRFGHTILNGDFMQIDSRGRISRVKLQDNFFKP---FEFRDG 406

Query 430 IIQCLIK---EGSEEAYDRYVSSAVSEHLFESTRGHKHALDLIAVNIQRGRDHGIPAY-H 485

+++ +++ + + + +D ++++ V+ HL+ T LDLI++NIQRGRDHGI Y

Sbjct 407 MMERIVRGLAKQTSQTFDNFITNDVTNHLYRLT-NESFGLDLISLNIQRGRDHGIRGYTD 465

Query 486 YWRQYYRLRRIISLDEFGEAGIAM--------KKAYRDIRDVDLFPGGLLEPSMPGGVVG 537

Y + + LR + +F + AM ++ Y + D+DLF GG+ E S+PGGVVG

Sbjct 466 YLKGCFGLR----VTKFEDLDSAMPRPVRERLQRLYTHVNDIDLFTGGVSEYSLPGGVVG 521

Query 538 ETFGHILANQFADLKFGDTYFFLHQQAPQGFRAAQIKAILSVTMSSIICANSAVTQ-AQP 596

TFG IL QF LK+GD Y+F H F AQ+ + T+S IIC NS Q AQ

Sbjct 522 PTFGCILGIQFWRLKYGDRYYFEHGGQAGSFTPAQLTELRRTTLSKIICDNSIGHQSAQR 581

Query 597 DPFYMASQLNLPRPCSDYSEMDVEPWL 623

F S N PCS M+++ W+

Sbjct 582 YVFRTISDSNPEVPCSSLPVMNMDAWI 608

>[XP_021365517.1](https://www.ncbi.nlm.nih.gov/protein/XP_021365517.1?report=genbank&log$=protalign&blast_rank=65&RID=0) peroxidase-like protein [Mizuhopecten yessoensis]

Length=705

Score = 297 bits (760), Expect = 2e-87, Method: Compositional matrix adjust.

Identities = 202/602 (34%), Positives = 304/602 (50%), Gaps = 75/602 (12%)

Query 57 YRQIDGRCNHPRN--YGSTGRPVKRYLRPHYQDKFGENLPRVYSVTGQLLPSPRMVSWKL 114

YR +DG CN+ + GS R ++P Y D G NLPR+ SVTG LP+PR++S

Sbjct 124 YRTVDGTCNNLGSPLLGSRMIAQGRVIQPFYDD--GLNLPRMRSVTGGYLPNPRLISNVF 181

Query 115 HPDQ----TAHDNNTMLVMQMGQFIDHDITRAPELSGRNA-SIKCC--GVPPKERLPDCF 167

H + + NT + GQF+DHDI+ P L ++ +++CC G R CF

Sbjct 182 HSNTGRSPVLNKRNTNMFFAYGQFLDHDISLTPVLQDKDGKNLECCRAGGLHDSR---CF 238

Query 168 PIDIPPGDPVFEDCMEFFRS--SPAVDNDGNIIYPREQINALTSFIDGSAVYGSDLDTYT 225

PI +PP DPVF C F RS +P+ D + PR QIN LTSFIDGS +Y S +

Sbjct 239 PIPVPPNDPVFHVCKNFARSAATPSFQCDPS---PRLQINQLTSFIDGSTIYSSTPEQLD 295

Query 226 WIRSENGTGVFLNTHLVHGRERLPSH--PHLGPESCVSSNTAESYCQLAGDMRVNEQPGL 283

+R + +GR RL + P G + + +C L GD R +E P L

Sbjct 296 ILREKR-----------YGRLRLTARNLPLAGSSDMCTLSCPGHHCFLTGDGRRHEVPTL 344

Query 284 GSIHLLFHLHHNHIVRLLVAGILKKRGQPSSPERIAKFIQESSSALKEQIFQEVRKMLGA 343

++H++F HN + R L +F+ E++FQE R++LGA

Sbjct 345 TTLHVIFAREHNKLARGL------------------QFVNPHWD--DERLFQEARRILGA 384

Query 344 IIQKLTYCDWLPMILGPYLIDKFQLGCTRRSR---YNSDLDPRVANSFLSAALRFGHTLI 400

+Q + + ++LP +LG ++ KF L R Y+ +DP ++F +AA RFGH+ I

Sbjct 385 ELQHIAFFEYLPFVLGNDVMRKFGLTPAARGHREVYDPTVDPSTTSAFSTAAFRFGHSQI 444

Query 401 PNVYNFGDK------RIHLKDTFNIPDA--SIRYYDNIIQCLIKEGSEEAYDRYVSSAVS 452

P+ K + L F PD ++ +N+I+ L EE DR +S +V

Sbjct 445 PSTIGLMAKDHTIIDEMPLHTQFERPDIFFNMDGSENLIRWLTNSNHEEN-DRLISDSVR 503

Query 453 EHLFESTRGHKHALDLIAVNIQRGRDHGIPAYHYWRQYYRLRRIIS--------LDEFGE 504

+LF++ + +DL A NIQRGRDHGIP Y+ WR++ LR ++ +D G

Sbjct 504 NNLFQNMGPMQ--MDLAATNIQRGRDHGIPPYNVWREWCGLRPVVHFGKGPGGLVDHDGI 561

Query 505 AGIAMKKAYRDIRDVDLFPGGLLEPSMPGGVVGETFGHILANQFADLKFGDTYFFLHQQA 564

A I + K YR D+DLFP G+ E ++PG +G TF I+A QFA + GD +++ +

Sbjct 562 ARIFLSKLYRHPNDIDLFPAGVSEKNLPGASIGPTFACIIAKQFARFQRGDRFYYENANT 621

Query 565 PQGFRAAQIKAILSVTMSSIICANSAVTQAQPDPFYMASQLNLPR-PCSDYSEMDVEPWL 623

P F A Q+ + T+++I C NS + QP F + + P+ PC +++ W

Sbjct 622 PGSFTADQLNELKKTTLAAIQCRNSRIQATQPHQFLRPDRRSNPKIPCQMIPSLNLRFWQ 681

Query 624 IH 625

H

Sbjct 682 EH 683

>[XP_023210206.1](https://www.ncbi.nlm.nih.gov/protein/XP_023210206.1?report=genbank&log$=protalign&blast_rank=66&RID=0) chorion peroxidase-like, partial [Centruroides sculpturatus]

Length=1450

Score = 308 bits (790), Expect = 2e-87, Method: Compositional matrix adjust.

Identities = 208/585 (36%), Positives = 307/585 (52%), Gaps = 63/585 (11%)

Query 57 YRQIDGRCNHPRN--YGSTGRPVKRYLRPHYQDKFGENLPRVYSVTGQLLPSPRMVSWKL 114

YR DG CN+ N +G + P +R+L P Y D G N PR+ + G LPS R VS

Sbjct 222 YRTSDGSCNNLNNLSWGKSFTPFQRFLPPKYFD--GINAPRI-AADGGPLPSARDVSDAA 278

Query 115 HPDQTAHDN-NTMLVMQMGQFIDHDITRAPELSGR-NASIKCCGVP----PKERLPDCFP 168

+PD+ + T+++MQ QF+DHDIT + N I CC PK P CFP

Sbjct 279 NPDKDVPNAIFTLIIMQWAQFVDHDITLTAITKNKDNEGILCCDPKIQRNPKLLHPACFP 338

Query 169 IDIPPGDPVF----EDCMEFFRSSPAVDNDGNIIYPREQINALTSFIDGSAVYGSDLDTY 224

I I D + E CMEF RS A + + REQIN LT+F+DGS VYGS +

Sbjct 339 IAISKNDRFYSKFNESCMEFVRSLAAPKPECTL-GSREQINQLTAFLDGSNVYGSTEEEA 397

Query 225 TWIRSENGTGVFLNTHLVHGRERLPSHPHLGPESCVSSNTAESYCQLAGDMRVNEQPGLG 284

+RS G N L HG + LP H + + E C LAGD RVNEQ L

Sbjct 398 NNLRSFK-EGKLKNLEL-HGEQFLPQEQHKSEDCAIR----EQACFLAGDERVNEQINLA 451

Query 285 SIHLLFHLHHNHIVRLLVAGILKKRGQPSSPERIAKFIQESSSALKEQI-FQEVRKMLGA 343

+H L+ HN R+A+ +++ + +QI + E R+++GA

Sbjct 452 IMHTLWMREHN---------------------RVAEELKKQNPGWNDQILYLEARRIVGA 490

Query 344 IIQKLTYCDWLPMILGPYLIDKFQLGCTRRSR---YNSDLDPRVANSFLSAALRFGHTLI 400

IQ +TY ++LP++LG ++ + L + Y+ +L+P +AN F +AA R+GHTL+

Sbjct 491 EIQHITYNEFLPLLLGNNIVSSYGLLPKPYGKIFDYDPELNPSIANGFATAAYRYGHTLV 550

Query 401 PNVYNFGDK------RIHLKDTFNIPDASIR--YYDNIIQCLIKEGSEEAYDRYVSSAVS 452

+ ++ ++ L TF P R Y+ ++ L+ + +++ YDR+++S ++

Sbjct 551 QGFIDLLERNGEVKEKVPLSSTFFNPILLYRPKYFSKFVRGLVGQPAQK-YDRFITSQLT 609

Query 453 EHLFESTRGHKHALDLIAVNIQRGRDHGIPAYHYWRQYYRLRRIISLDEFG-----EAGI 507

HLF+ GH LDL+A+N QRGRDHGIP Y+ WR++ +L+ S DE ++

Sbjct 610 NHLFQP-HGHHFGLDLVALNTQRGRDHGIPPYNEWRRWCKLKPFESFDELNRLMTPQSVQ 668

Query 508 AMKKAYRDIRDVDLFPGGLLEPSMPGGVVGETFGHILANQFADLKFGDTYFFLHQQAPQG 567

A KK YR + D+DLF G+ E S+P G +GETF I+ QF LKFGD +++ + +

Sbjct 669 AYKKLYRSVEDIDLFTAGVSERSVPDGTLGETFACIVGEQFRRLKFGDRFWYENGKLESS 728

Query 568 FRAAQIKAILSVTMSSIICANSAVTQA-QPDPFYMASQLNLPRPC 611

F Q+ I ++S I+C N+ A QP F AS+ N PC

Sbjct 729 FTDDQLAEIRKTSLSRILCDNTKEFDAVQPLAFIRASKWNPRVPC 773

Score = 285 bits (728), Expect = 5e-79, Method: Compositional matrix adjust.

Identities = 186/535 (35%), Positives = 286/535 (53%), Gaps = 54/535 (10%)

Query 57 YRQIDGRCNHPRN--YGSTGRPVKRYLRPHYQDKFGENLPRVYSVTGQLLPSPRMVSWKL 114

YR +DG CN+ +N +G + +R L P Y D G N PRV + G LPS R+VS +L

Sbjct 945 YRSMDGSCNNLKNRLWGKSFTAFERLLPPDYAD--GINKPRV-AKDGSELPSARLVSSRL 1001

Query 115 HPD-QTAHDNNTMLVMQMGQFIDHDITRAPELSGRNAS-IKCCGVPPKERLPDCFPIDIP 172

D AH T L + GQF+DHD+T G N I+CCG + P CFPI +P

Sbjct 1002 SSDHNAAHGYLTTLFVYWGQFLDHDLTLTALSRGNNGRRIECCGRQSRMH-PSCFPISLP 1060

Query 173 PGDPVF----EDCMEFFRSSPAVDNDGNIIYPREQINALTSFIDGSAVYGSDLDTYTWIR 228

D + E CMEF RS + N REQ+N LT++IDGS +YGS + +R

Sbjct 1061 KNDTFYSKFDETCMEFVRSFASPRPKCNF-GAREQLNQLTAYIDGSNIYGSTKEVADELR 1119

Query 229 SENGTGVFLNTHLVHGRERLPSHPHLGPESCVSSNTAESYCQLAGDMRVNEQPGLGSIHL 288

S +G + +N +G LP + E C T E +C ++GD+RVNE L +H

Sbjct 1120 SFSGGKLKINYQ--NGAVMLP-RVNKRNEQCERKQT-ERFCFMSGDIRVNENINLAIMHT 1175

Query 289 LFHLHHNHIVRLLVAGILKKRGQPSSPERIAKFIQE-SSSALKEQIFQEVRKMLGAIIQK 347

++ HN RIA +QE + S E+++QE R+++ A IQ

Sbjct 1176 IWLREHN---------------------RIAGKLQEINPSWDDEKLYQESRRVVAAQIQH 1214

Query 348 LTYCDWLPMILGPYLIDKFQLGCTRRS---RYNSDLDPRVANSFLSAALRFGHTLIPNVY 404

+TY ++LP++LG + + F+L R YN + P + NSF +AA RFGHTL+ +

Sbjct 1215 ITYKEFLPLLLGHTVFNIFKLNLKSRGYSYEYNPSIKPSILNSFATAAYRFGHTLVQGLV 1274

Query 405 NFGDKRIHLKD---TFNIPDASIRYYDNIIQCLIK---EGSEEAYDRYVSSAVSEHLFES 458

+ ++++ + + N+ + ++ Y N I+ L++ + + +DR+++S ++ HLFE

Sbjct 1275 DLKNEKVVTRQFSLSDNLFNPALLYRKNHIEMLLRGMIDQPSQTFDRFITSQLTNHLFER 1334

Query 459 TRGHKHALDLIAVNIQRGRDHGIPAYHYWRQYYRLRRIISLDEFGE-----AGIAMKKAY 513

G K LDLI++NIQRGRDHG+P Y+ WR+ +L I + + E ++ Y

Sbjct 1335 P-GEKFGLDLISMNIQRGRDHGLPGYNQWRKICKLTPITTFSKLEEVTGKQTADIFRRLY 1393

Query 514 RDIRDVDLFPGGLLEPSMPGGVVGETFGHILANQFADLKFGDTYFFLHQQAPQGF 568

+ + D+DLF G+ E M G+VG TF I+A QF LK+GD +++ + + F

Sbjct 1394 KSVDDIDLFAAGIAEYPMSDGIVGPTFACIIAEQFRRLKYGDRFWYENGKQETSF 1448

>[KXJ70077.1](https://www.ncbi.nlm.nih.gov/protein/KXJ70077.1?report=genbank&log$=protalign&blast_rank=67&RID=0) hypothetical protein RP20_CCG024878 [Aedes albopictus]

Length=842

Score = 300 bits (767), Expect = 3e-87, Method: Compositional matrix adjust.

Identities = 217/606 (36%), Positives = 320/606 (53%), Gaps = 83/606 (14%)

Query 56 RYRQIDGRCNHPRN---YGSTGRPVKRYLRPHYQDKFGENLPRVYSVTGQLLPSPRMVSW 112

RYR +G CN+ +N YG P +R L P Y D G + PR SV G+ LPS R VS

Sbjct 211 RYRTNNGTCNNKKNPHTYGVALIPFRRQLTPDYGD--GVSSPR-ESVEGKELPSARQVSL 267

Query 113 KLHPDQTAHDNN-TMLVMQMGQFIDHDITRAPELSGRNA-SIKCCGVPPKERLPDCFPID 170

++H +D N ++++ GQF+DHDIT G +I+CC P + R P+CFP+

Sbjct 268 QIHRPSYHNDPNFSVMLAVWGQFLDHDITSTALNQGVGGKAIECCD-PGQPRHPECFPVP 326

Query 171 IPPGDPVFED----CMEFFRSSPAVDNDGNIIYPREQINALTSFIDGSAVYGSDLDTYTW 226

+ PGDP F D CM F RS PA PR+Q+N T++IDGS VYGSD

Sbjct 327 LGPGDPYFHDYNLTCMNFVRSIPAPTGH---FGPRQQLNQATAYIDGSVVYGSDDAKVKR 383

Query 227 IRSENGTGVFLNTHLV-HGRERLP--SHPHLGPESCVSSNTAESYCQLAGDMRVNEQPGL 283

+R+ G L ++ RE LP + P+ G + N A YC +GD R NE L

Sbjct 384 LRT--GQDGKLRMYVTPDNRELLPISTDPNDGCNE-EAMNAAGKYCFESGDERANENLHL 440

Query 284 GSIHLLFHLHHNHIVRLLVAGILKKRGQPSSPERIAKFIQESSSALKEQIFQEVRKMLGA 343

S+HL++ HHN++ G LKK ER+ FQE R++L A

Sbjct 441 TSMHLIWARHHNNLT-----GELKKVNPDWDDERL---------------FQEARRILAA 480

Query 344 IIQKLTYCDWLPMILGPYLIDKFQLGC---TRRSRYNSDLDPRVANSFLSAALRFGHTLI 400

+Q +TY +++P+I+G ++ +L + R YN +DP VAN F ++A RF HTL+

Sbjct 481 QMQHITYGEFVPVIVGEDTAERMELAPNPESDRDTYNVSVDPSVANVFAASAFRFAHTLL 540

Query 401 PNVYNFGDKRIHLKDTFNIPDASIRYYD---NIIQCLIKEGSEEA-----------YDRY 446

P + K+ H D + P + I + N K G ++A YD+Y

Sbjct 541 PGLM----KKTH--DPTSSP-SGIELHKMLFNPYSLYGKTGLDDAIGGAMTTPLGKYDQY 593

Query 447 VSSAVSEHLFESTRGHKH----ALDLIAVNIQRGRDHGIPAYHYWRQYYRLRRIISLDEF 502

++ ++EHLFE + H LDL+++NIQRGRDHG+P+Y +WR++ RL + + D+

Sbjct 594 FTTELTEHLFEKAQDLLHDRPCGLDLVSLNIQRGRDHGLPSYPHWRRHCRLPPVDTWDQL 653

Query 503 GEAGIA-----MKKAYRDIRDVDLFPGGLLEPSMPGGVVGETFGHILANQFADLKFGDTY 557

+ A M+K Y + +VD++ G L EP + GGVVG +LA+QF LK GD++

Sbjct 654 EKVVDAGSYQQMRKIYGEPENVDVYSGALSEPPVEGGVVGPLITCLLADQFLRLKQGDSF 713

Query 558 FFLHQQAPQGFRAAQIKAILSVTMSSIICANS-AVTQAQPDPFYMASQLNLPR----PCS 612

++ ++ PQ F Q++ I + +SSIIC NS A+TQ+ P Y+ ++N PCS

Sbjct 714 WYERRRGPQRFTRDQLRQIYNTRLSSIICRNSDAITQS---PVYLMRKVNREDNPELPCS 770

Query 613 DYSEMD 618

D

Sbjct 771 QLDTFD 776

>[XP_029850035.1](https://www.ncbi.nlm.nih.gov/protein/XP_029850035.1?report=genbank&log$=protalign&blast_rank=68&RID=0) thyroid peroxidase [Ixodes scapularis]

Length=1195

Score = 306 bits (783), Expect = 3e-87, Method: Compositional matrix adjust.

Identities = 215/596 (36%), Positives = 311/596 (52%), Gaps = 63/596 (11%)

Query 24 ARRGDLGDSTGSLDFVSEADLEHCSRLTYD-QLRYRQIDGRCNHPRN--YGSTGRPVKRY 80

AR D+ S+D + A LT D RYRQ DG CN+ +N +GS G ++R

Sbjct 596 ARADDVDFQAISVDGTALAATCPSPNLTCDPNQRYRQPDGTCNNLKNPGWGSAGSCMQRL 655

Query 81 LRPHYQDKFGENLPRVYSVTGQLLPSPRMVSWKLHPD-QTAHDNNTMLVMQMGQFIDHDI 139

L P YQD G + PRV +++G LP+ R++S +H D N T +VMQ+GQFIDHD

Sbjct 656 LPPAYQD--GISAPRV-AISGGPLPNARLISSTVHSDMNNPAINFTHMVMQIGQFIDHDF 712

Query 140 TRAP---------ELSGRNASIKCCGVPPKERLPDCFPIDIPPGDPVF----EDCMEFFR 186

AP L + I CC P + +CF IDIP GDP F + C+ R

Sbjct 713 ALAPLMPDPGEIVNLGNPDNVIDCCS-PSTRNMSECFSIDIPSGDPFFARFNQTCINMPR 771

Query 187 SSPAVDNDGNIIYPREQINALTSFIDGSAVYGSDLDTYTWIRSENGTGVFLNTHLVHGRE 246

S+P + N+ Y R+Q + LTS++D S VYGS +RS + +L + G E

Sbjct 772 SAPC--SRCNLGY-RDQQDILTSYLDTSQVYGSSAADTQRLRSLSRG--YLKSQRASGLE 826

Query 247 RLPSHPHLGPESCVSSNTAESYCQLAGDMRVNEQPGLGSIHLLFHLHHNHIVRLLVAGIL 306

LP H + C S + YC AGD RVNE PGL SIH LF HN + R

Sbjct 827 LLPRSFHPTMDRC-SDPSNNQYCFRAGDERVNEHPGLTSIHTLFLREHNRLAR------- 878

Query 307 KKRGQPSSPERIAKFIQESSSALKEQIFQEVRKMLGAIIQKLTYCDWLPMILGPYLIDKF 366

I F S E+IF+ ++++ A Q + Y +WLP+I+GP + ++

Sbjct 879 ----------PIGYFHPFYS---DERIFKMTKRIVEATFQHIVYSEWLPVIMGPAEMARY 925

Query 367 QLG--CTRRSRYNSDLDPRVANSFLSAALRFGHTLI------PNVYNFGDKRIHLKDTFN 418

QL T +RYN +D + N F +A R GHTLI PN + G+ + LK+ F

Sbjct 926 QLVLLSTGFTRYNDSVDATMMNEFAAAGFRLGHTLIDGSFNLPNFFGDGNATLDLKENFF 985

Query 419 IP-DASIRYYDNIIQCLIKEGSEEAYDRYVSSAVSEHLFESTRGHKHALDLIAVNIQRGR 477

P + + D +++ L+++ ++ +D++V+ AV+ HL+ R LDLIA+NIQR R

Sbjct 986 FPFELYNQQLDPLLRGLVQQPAQ-TFDKFVTDAVTNHLYR-LRNDSFGLDLIALNIQRAR 1043

Query 478 DHGIPAYHYWRQYYRLRRIISLDEFGE---AGIA--MKKAYRDIRDVDLFPGGLLEPSMP 532

+HG+ AY + I S D+ + + I + Y D+RD+DLF G+ E S+P

Sbjct 1044 EHGVRAYVDYVHLCSGVNITSFDDLLQNIPSSIVDQYRALYADVRDIDLFSAGISERSVP 1103

Query 533 GGVVGETFGHILANQFADLKFGDTYFFLHQQAPQGFRAAQIKAILSVTMSSIICAN 588

GGVVG TF IL + F L+FGD ++F H+ F +AQ+K I +M+ +IC N

Sbjct 1104 GGVVGPTFACILGHMFHRLRFGDRFWFEHEGQAGSFTSAQLKEIRKTSMAKLICDN 1159

Score = 273 bits (699), Expect = 1e-75, Method: Compositional matrix adjust.

Identities = 177/496 (36%), Positives = 260/496 (52%), Gaps = 60/496 (12%)

Query 123 NNTMLVMQMGQFIDHDITRAP---------ELSGRNASIKCCGVPPKERLPDCFPIDIPP 173

N T +VMQ+GQFIDHD AP L N I CC P + +CF IDIPP

Sbjct 7 NFTHMVMQIGQFIDHDFALAPLMPDPGEIVNLGNPNNVIDCCS-PSTRNMSECFSIDIPP 65

Query 174 GDPVF----EDCMEFFRSSPAVDNDGNIIYPREQINALTSFIDGSAVYGSDLDTYTWIRS 229

GDP F + C+ RS+P + N+ Y R+Q + LTS++D S VYGS +RS

Sbjct 66 GDPFFAGFNQTCINMPRSAPC--SRCNLGY-RDQQDILTSYLDNSQVYGSSAADTQRLRS 122

Query 230 ENGTGVFLNTHLVHGRERLPSHPHLGPESCVSSNTAESYCQLAGDMRVNEQPGLGSIHLL 289

+ L + V GRE LP H + C S + YC AGD R NE PGL SIH +

Sbjct 123 LSRG--MLKSQRVSGRELLPRSFHPTMDRC-SDPSKNQYCFRAGDERANEHPGLTSIHTV 179

Query 290 FHLHHNHIVRLLVAGILKKRGQPSSPERIAKFIQESSSALKEQIFQEVRKMLGAIIQKLT 349

F HN + + G+L+ F + E+IFQ ++++ A Q +

Sbjct 180 FLREHNRLAGQI--GLLRP------------FYND------EKIFQTAKRIVEATFQHIV 219

Query 350 YCDWLPMILGPYLIDKFQLGCTRR--SRYNSDLDPRVANSFLSAALRFGHTLI------P 401

Y +WLP+I+GP + ++QL R +RYN ++ + N F +A R GHTLI P

Sbjct 220 YSEWLPVIMGPAEMARYQLVLLRTGFTRYNDSVEATMMNEFAAAGFRLGHTLIDGSFNMP 279

Query 402 NVYNFGDKRIHLKDTFNIPDASIRYYDNIIQCLIK---EGSEEAYDRYVSSAVSEHLFES 458

N + G+ + LK+ F P +Y+ + L++ + + +D++V++AV++HL+

Sbjct 280 NFFGGGNATLDLKENFFFP---FEFYNGQLDPLLRGLVQQPAQTFDKFVTNAVTDHLYR- 335

Query 459 TRGHKHALDLIAVNIQRGRDHGIPAYHYWRQYYRLRRIISLDEFGE---AGIA--MKKAY 513

R LDLI++NIQR R+HG+ AY + I S D+ + + I + Y

Sbjct 336 LRNDSFGLDLISLNIQRAREHGVRAYVDYVNLCTGVNITSFDDLLQNIPSSIVDQYRALY 395

Query 514 RDIRDVDLFPGGLLEPSMPGGVVGETFGHILANQFADLKFGDTYFFLHQQAPQGFRAAQI 573

D+RD+DLF G+ E S+PGGVVG TF IL + F L+FGD ++F H+ F +AQ+

Sbjct 396 ADVRDIDLFSAGISERSVPGGVVGPTFACILGHMFQRLRFGDRFWFEHKDQAGSFTSAQL 455

Query 574 KAILSVTMSSIICANS 589

I +M+ +IC NS

Sbjct 456 GEIRKTSMARLICDNS 471

>[XP_009058281.1](https://www.ncbi.nlm.nih.gov/protein/XP_009058281.1?report=genbank&log$=protalign&blast_rank=69&RID=0) hypothetical protein LOTGIDRAFT_163526 [Lottia gigantea]

[ESO91010.1](https://www.ncbi.nlm.nih.gov/protein/ESO91010.1?report=genbank&log$=protalign&blast_rank=69&RID=0) hypothetical protein LOTGIDRAFT_163526 [Lottia gigantea]

Length=643

Score = 295 bits (754), Expect = 4e-87, Method: Compositional matrix adjust.

Identities = 208/611 (34%), Positives = 314/611 (51%), Gaps = 83/611 (14%)

Query 56 RYRQIDGRCNHPRN--YGSTGRPVKRYLRPHYQDKFGENLPRVYSVTGQLLPSPRMVSWK 113

RYR DG CN+P +G+T ++R +R +Y D E PR + LP+ R +S

Sbjct 66 RYRSPDGICNNPSAPYWGATDTSLRRIVRNYYDDGISE--PRSKGIDNNELPNVRYISNT 123

Query 114 LHPDQTAHDNN--TMLVMQMGQFIDHDI---------------TRAPELS---GRNASIK 153

+ D T N T L GQF+DHDI PEL+ G+++ IK

Sbjct 124 ISVDFTGDVKNPFTNLHQTFGQFLDHDIGHTPNILDKDKKKIKCCTPELTKNGGQHSDIK 183

Query 154 CCGVPPKERLPDCFPIDIPPGDPVFE--DCMEFFRSSPAVDNDGNI-IYPREQINALTSF 210

G CFPI+IP DP F+ CM F RS D REQ+N LTSF

Sbjct 184 TGG--------PCFPIEIPTKDPFFQPRRCMNFVRSIEVTDKQKKCRSAAREQMNKLTSF 235

Query 211 IDGSAVYGSDLDTYTWIRSENGTGVFLNTHLVHGRERLPSHPHLGPESCVSSNTAESYCQ 270

ID S +YG +D +R+ G V + LP + + C N+++ YC

Sbjct 236 IDASNIYGLTVDDGKKLRTFKG-----GLMKVSAYDLLPENIN---GECEKRNSSD-YCF 286

Query 271 LAGDMRVNEQPGLGSIHLLFHLHHNHIVRLLVAGILKKRGQPSSPERIAKFIQESSSALK 330

LAGDMRVNE PGL + H +F +HN I A LK Q + R+

Sbjct 287 LAGDMRVNEHPGLSAFHAIFVRYHNKI-----AANLK---QLNGRNRLWD---------D 329

Query 331 EQIFQEVRKMLGAIIQKLTYCDWLPMILGPYLIDKFQL--GCTRRSRYNSDLDPRVANSF 388

E+I+QE +K++ ++Q + Y ++LP ++G K++L T + R+ S +DPR+ N+F

Sbjct 330 ERIYQETKKIVNGVLQNIVYNEYLPKLVGDVATIKYKLKSSLTGQHRFLSSIDPRIMNAF 389

Query 389 LSAALRFGHTLIPNVYNFGDKRIHLKDTFNIP----DASIRYYDNIIQCLIKEGSEEAYD 444

+AA R+GH+ IPN +++GDK++ L FN P D + D I++ ++K+ S+ A D

Sbjct 390 STAAFRYGHSSIPNEWSYGDKKVPLVAMFNSPFFIQDFKGKGLDMIVEGMVKDSSQPA-D 448

Query 445 RYVSSAVSEHLFESTRGHKHALDLIAVNIQRGRDHGIPAYHYWRQYYRLRRIISLDEFGE 504

+ S+ V LF++ + LDL+A+N+QRGRDHG+ Y+ +R+Y L+ I D +

Sbjct 449 KSFSNGVRNTLFKTFK--VPGLDLVALNLQRGRDHGLAPYNIYREYCGLKPIEKFDGSTD 506

Query 505 AGIAMKKAYRDIRDVDLFPGGLLEPSMPGGVVGETFGHILANQFADLKFGDTYFFLH-QQ 563

+ +AY+ DVDLF GG+ EP++ G VG TF ++A+QF +LK+GD ++F + QQ

Sbjct 507 QIRTLAQAYKHPNDVDLFIGGMTEPTVHNGKVGPTFACLIASQFHNLKYGDRFWFENVQQ 566

Query 564 A-------PQGFRAAQIKAILSVTMSSIICANSAVTQAQPDPFYMASQL--NLPRPCSDY 614

A P F QI+AI V+++ I+C + + F+ S N C DY

Sbjct 567 ASPFLPVNPAAFSIGQIQAIRQVSIARILCDTTDIKYVPRYAFFHPSARLGNRRVSCKDY 626

Query 615 SE---MDVEPW 622

+ + PW

Sbjct 627 YSLPGLTITPW 637

>[OWF44553.1](https://www.ncbi.nlm.nih.gov/protein/OWF44553.1?report=genbank&log$=protalign&blast_rank=70&RID=0) Peroxidase-like protein [Mizuhopecten yessoensis]

Length=604

Score = 293 bits (751), Expect = 4e-87, Method: Compositional matrix adjust.

Identities = 196/572 (34%), Positives = 288/572 (50%), Gaps = 81/572 (14%)

Query 57 YRQIDGRCNHPRN--YGSTGRPVKRYLRPHYQDKFGENLPRVYSVTGQLLPSPRMVSWKL 114

YR +DG CN+ + GS R ++P Y D G NLPR+ SVTG LP+PR++S

Sbjct 24 YRTVDGTCNNLGSPLLGSRMIAQGRVIQPFYDD--GLNLPRMRSVTGGYLPNPRLISNVF 81

Query 115 HPDQ----TAHDNNTMLVMQMGQFIDHDITRAPELSGRNASIKCCGVPPKERLPDCFPID 170

H + + NT + GQF+DHDI+ P L G+N S CFPI

Sbjct 82 HSNTGRSPVLNKRNTNMFFAYGQFLDHDISLTPVLQGKNLS-------------RCFPIP 128

Query 171 IPPGDPVFEDCMEFFRS--SPAVDNDGNIIYPREQINALTSFIDGSAVYGSDLDTYTWIR 228

+PP DPVF C F RS +P+ D + PR QIN LTSFIDGS +Y S + +R

Sbjct 129 VPPNDPVFHVCKNFARSAATPSFQCDPS---PRLQINQLTSFIDGSTIYSSTPEQLDILR 185

Query 229 SENGTGVFLNTHLVHGRERLPSH--PHLGPESCVSSNTAESYCQLAGDMRVNEQPGLGSI 286

+ +GR RL + P G + + +C L GD R +E P L ++

Sbjct 186 EKR-----------YGRLRLTARNLPLAGSSDMCTLSCPGHHCFLTGDGRRHEVPTLTTL 234

Query 287 HLLFHLHHNHIVRLLVAGILKKRGQPSSPERIAKFIQESSSALKEQIFQEVRKMLGAIIQ 346

H++F HN + R L +F+ E++FQE R++LGA +Q

Sbjct 235 HVIFAREHNKLARGL------------------QFVNPHWD--DERLFQEARRILGAELQ 274

Query 347 KLTYCDWLPMILGPYLIDKFQLGCTRRSR---YNSDLDPRVANSFLSAALRFGHTLIPNV 403

+ + ++LP +LG ++ KF L R Y+ +DP ++F +AA RFGH+ IP+

Sbjct 275 HIAFFEYLPFVLGNDVMRKFGLTPAARGHREVYDPTVDPSTTSAFSTAAFRFGHSQIPST 334

Query 404 YNFGDK------RIHLKDTFNIPDA--SIRYYDNIIQCLIKEGSEEAYDRYVSSAVSEHL 455

K + L F PD ++ +N+I+ L EE DR +S +V +L

Sbjct 335 IGLMAKDHTIIDEMPLHTQFERPDIFFNMDGSENLIRWLTNSNHEEN-DRLISDSVRNNL 393

Query 456 FESTRGHKHALDLIAVNIQRGRDHGIPAYHYWRQYYRLRRIIS--------LDEFGEAGI 507

F++ + +DL A NIQRGRDHGIP Y+ WR++ LR ++ +D G A I

Sbjct 394 FQNMGPMQ--MDLAATNIQRGRDHGIPPYNVWREWCGLRPVVHFGKGPGGLVDHDGIARI 451

Query 508 AMKKAYRDIRDVDLFPGGLLEPSMPGGVVGETFGHILANQFADLKFGDTYFFLHQQAPQG 567

+ K YR D+DLFP G+ E ++PG +G TF I+A QFA + GD +++ + P

Sbjct 452 FLSKLYRHPNDIDLFPAGVSEKNLPGASIGPTFACIIAKQFARFQRGDRFYYENANTPGS 511

Query 568 FRAAQIKAILSVTMSSIICANSAVTQAQPDPF 599

F A Q+ + T+++I C NS + QP F

Sbjct 512 FTADQLNELKKTTLAAIQCRNSRIQATQPHQF 543

>[XP_021963148.1](https://www.ncbi.nlm.nih.gov/protein/XP_021963148.1?report=genbank&log$=protalign&blast_rank=71&RID=0) peroxidase isoform X1 [Folsomia candida]

Length=924

Score = 301 bits (771), Expect = 5e-87, Method: Compositional matrix adjust.

Identities = 213/597 (36%), Positives = 323/597 (54%), Gaps = 70/597 (12%)

Query 57 YRQIDGRCNHPRN--YGSTGRPVKRYLRPHYQDKFGENLPRVYSVTGQLLPSPRMVSWKL 114

YR +DG CN+ +N +G + +R L P Y D G N PR + Q LPS R +S +

Sbjct 240 YRTMDGSCNNKQNPDWGKSQTAFQRILPPKYGD--GINSPRAQA-NRQPLPSARTISDRF 296

Query 115 HPDQTAHDNN-TMLVMQMGQFIDHDITRAPELSGRNA-SIKCC--GVPPKERL--PDCFP 168

D+ NN T++VMQ GQF+DHD+T P + G N+ I CC G P E L PDCFP

Sbjct 297 ITDENRPYNNLTLMVMQWGQFLDHDLTHTPIVKGNNSVGISCCQNGQPLPENLRHPDCFP 356

Query 169 IDIPPGDPVF----EDCMEFFRSSPAVDNDGNIIYPREQINALTSFIDGSAVYGSDLDTY 224

I+I D + + CMEF RS PA + N PREQ+N +T++IDGS VYGS +

Sbjct 357 IEISSEDSFYRRFTQRCMEFVRSLPAPRRECNF-GPREQMNQITAYIDGSNVYGSSDNRA 415

Query 225 TWIRSENGTGVFLNTHLVHGRERLPSHPHLGPESCVSSNTAES-YCQLAGDMRVNEQPGL 283

+R G + + T+ E LP++ S SN A+ +C AGD+RVNEQ L

Sbjct 416 RSLREGVGGRLLVQTN--QRTELLPAN------SNECSNAAQDRFCFRAGDLRVNEQVEL 467

Query 284 GSIHLLFHLHHNHIVRLLVAGILKKRGQPSSPERIAKFIQESSSALKEQIFQEVRKMLGA 343

+H ++ HN I R+L G+ P+ + I ++QE R+++ A

Sbjct 468 ALMHTIWLREHNRIARIL-QGL-----NPTWSDEI--------------LYQETRRIVIA 507

Query 344 IIQKLTYCDWLPMILGPYLIDKFQL-----GCTRRSRYNSDLDPRVANSFLSAALRFGHT 398

++ +TY ++LP+ILGP + F++ G TR YN ++D + N+F +AA RFGH+

Sbjct 508 ELEHITYNEFLPLILGPDYMRTFEMQPRQKGYTRL--YNEEIDASITNAFSTAAYRFGHS 565

Query 399 LIPNVY----NFGDKR-IHLKDTFNIPDASIRYYDNIIQCLIKEGSEEAYDRYVSSAVSE 453

++ + FG R + T P D++++ L + S+ D +VS A++

Sbjct 566 MVQGIIQTFSKFGSSRTMPFAKTQFQPFQLYDSLDDLVRGLTNQESQR-MDHFVSEAITN 624

Query 454 HLFESTRGHKHALDLIAVNIQRGRDHGIPAYHYWRQYYRLRRIISLDEF-----GEAGIA 508

HLFE + G LDL+A+NIQRGRDHG+P+Y+ WR+ L++I S E A

Sbjct 625 HLFEGSSGF--GLDLMALNIQRGRDHGLPSYNDWREVCSLKKIGSWQELVGVMDDSAVRI 682

Query 509 MKKAYRDIRDVDLFPGGLLEPSMPGGVVGETFGHILANQFADLKFGDTYFFLHQQAPQGF 568

+ + Y + ++DLF GG+ E G V+G TF I+ +QF+ L+ GD +F+ ++ F

Sbjct 683 LSQLYPSVDEIDLFVGGIGERPQSGAVLGPTFVCIVGDQFSRLRRGDRFFY--EEVSSRF 740

Query 569 RAAQIKAILSVTMSSIICANS-AVTQAQPDPFYMASQLNLPRPCSD--YSEMDVEPW 622

AQ+ I V+++ IIC NS ++ QP F AS LN +PC+ ++D++ W

Sbjct 741 TEAQLDQIRKVSLARIICDNSDDISVIQPLVFQQASFLNQRQPCNTEVIPKLDLKFW 797

>[GAU92485.1](https://www.ncbi.nlm.nih.gov/protein/GAU92485.1?report=genbank&log$=protalign&blast_rank=72&RID=0) hypothetical protein RvY_04560 [Ramazzottius varieornatus]

Length=557

Score = 292 bits (747), Expect = 5e-87, Method: Compositional matrix adjust.

Identities = 192/551 (35%), Positives = 286/551 (52%), Gaps = 59/551 (11%)

Query 103 LLPSPRMVSWKLHPD-QTAHDNNTMLVMQMGQFIDHDITRAPELS----GRNASIKCCGV 157

+LPS R++S +HPD H + T+L+M GQF+DHDI P + G+ ++I+CC +

Sbjct 1 MLPSARLISTVIHPDVDRPHCHYTLLLMHFGQFMDHDINHTPSTTLTGYGKASNIQCCEI 60

Query 158 PPK-ERLPDCFPIDIPPGDPVFED----CMEFFRSSPAVDNDGNIIYPREQINALTSFID 212

P P CFPI+IPP DP + CM F RS A G + PREQ+N LTSF+D

Sbjct 61 PHGLAGHPACFPIEIPPSDPYYAKYYLKCMNFVRSE-AAPRPGCTLGPREQVNQLTSFLD 119

Query 213 GSAVYGSDLDTYTWIR-SENGTGVFLNTHLVHGRERLPSHPHLGPESCVSSNTAESYCQL 271

GS +YGS + IR + G + H ++ LP P L C +N A C

Sbjct 120 GSQIYGSTYEDMKNIRLYQFGRLRTVFVDYCH-KDVLP--PDLETADCPDAN-ATLPCFR 175

Query 272 AGDMRVNEQPGLGSIHLLFHLHHNHIVRLLVAGILKKRGQPSSPERIAKFIQESSSALKE 331

AGD RVNE GL ++H +F HN I L + + + E

Sbjct 176 AGDSRVNENTGLATLHTIFTREHNRIADELY--------------YLNPYWDD------E 215

Query 332 QIFQEVRKMLGAIIQKLTYCDWLPMILGP---YLIDKFQLGCTRRSRYNSDLDPRVANSF 388

++FQE RK++GA++Q + Y +WLP++LG Y D Y+ +++P +AN F

Sbjct 216 RLFQEARKIVGALLQHILYNEWLPLVLGKDVMYQNDLLPTPSGYYGGYDKNINPTIANVF 275

Query 389 LSAALRFGHTLIPNVYNFGDKRIHLKDTFNIPDASIRYY--------DNIIQCLIKEGSE 440

+AALRFGHTLIP+ + F +K + DA + Y D + +I + +

Sbjct 276 ATAALRFGHTLIPSWFRFFNKYHEYIGQKQLRDAFFKPYPLYQPGIIDMYLLGMINDNVQ 335

Query 441 EAYDRYVSSAVSEHLFESTRGHKHALDLIAVNIQRGRDHGIPAYHYWRQYYRLRRIISLD 500

+ D ++SS V+EHLFE+ H +DL A+N+QRGRDHGIP Y+ WR++ L + + D

Sbjct 336 KR-DTFISSEVTEHLFEAMP--THGVDLAAINVQRGRDHGIPPYNAWREFCGLPKAYAFD 392

Query 501 EFGEAGIA-----MKKAYRDIRDVDLFPGGLLEPSMPGGVVGETFGHILANQFADLKFGD 555

+ + A K Y + D+DLFP G+ E + G++G TF IL QF LK GD

Sbjct 393 DLKDVMRAEIVERFKTVYEHVEDIDLFPAGIAEEPLHDGLLGPTFTCILTKQFVHLKRGD 452

Query 556 TYFFLHQQAPQGFRAAQIKAILSVTMSSIICANS-AVTQAQPDPF-YMASQLNLPRPCSD 613

+++ + PQ F Q+ AI + +++ +IC NS + QP F ++ +N PC

Sbjct 453 RFWYENDVQPQAFTPDQLNAIRATSIARVICDNSDDIDTIQPKAFLHVLPSVNERVPCKG 512

Query 614 --YSEMDVEPW 622

+D+ W

Sbjct 513 PFLPRLDLSAW 523

>[XP_021963149.1](https://www.ncbi.nlm.nih.gov/protein/XP_021963149.1?report=genbank&log$=protalign&blast_rank=73&RID=0) peroxidase isoform X2 [Folsomia candida]

Length=921

Score = 301 bits (770), Expect = 6e-87, Method: Compositional matrix adjust.

Identities = 213/597 (36%), Positives = 323/597 (54%), Gaps = 70/597 (12%)

Query 57 YRQIDGRCNHPRN--YGSTGRPVKRYLRPHYQDKFGENLPRVYSVTGQLLPSPRMVSWKL 114

YR +DG CN+ +N +G + +R L P Y D G N PR + Q LPS R +S +

Sbjct 237 YRTMDGSCNNKQNPDWGKSQTAFQRILPPKYGD--GINSPRAQA-NRQPLPSARTISDRF 293

Query 115 HPDQTAHDNN-TMLVMQMGQFIDHDITRAPELSGRNA-SIKCC--GVPPKERL--PDCFP 168

D+ NN T++VMQ GQF+DHD+T P + G N+ I CC G P E L PDCFP

Sbjct 294 ITDENRPYNNLTLMVMQWGQFLDHDLTHTPIVKGNNSVGISCCQNGQPLPENLRHPDCFP 353

Query 169 IDIPPGDPVF----EDCMEFFRSSPAVDNDGNIIYPREQINALTSFIDGSAVYGSDLDTY 224

I+I D + + CMEF RS PA + N PREQ+N +T++IDGS VYGS +

Sbjct 354 IEISSEDSFYRRFTQRCMEFVRSLPAPRRECNF-GPREQMNQITAYIDGSNVYGSSDNRA 412

Query 225 TWIRSENGTGVFLNTHLVHGRERLPSHPHLGPESCVSSNTAES-YCQLAGDMRVNEQPGL 283

+R G + + T+ E LP++ S SN A+ +C AGD+RVNEQ L

Sbjct 413 RSLREGVGGRLLVQTN--QRTELLPAN------SNECSNAAQDRFCFRAGDLRVNEQVEL 464

Query 284 GSIHLLFHLHHNHIVRLLVAGILKKRGQPSSPERIAKFIQESSSALKEQIFQEVRKMLGA 343

+H ++ HN I R+L G+ P+ + I ++QE R+++ A

Sbjct 465 ALMHTIWLREHNRIARIL-QGL-----NPTWSDEI--------------LYQETRRIVIA 504

Query 344 IIQKLTYCDWLPMILGPYLIDKFQL-----GCTRRSRYNSDLDPRVANSFLSAALRFGHT 398

++ +TY ++LP+ILGP + F++ G TR YN ++D + N+F +AA RFGH+

Sbjct 505 ELEHITYNEFLPLILGPDYMRTFEMQPRQKGYTRL--YNEEIDASITNAFSTAAYRFGHS 562

Query 399 LIPNVY----NFGDKR-IHLKDTFNIPDASIRYYDNIIQCLIKEGSEEAYDRYVSSAVSE 453

++ + FG R + T P D++++ L + S+ D +VS A++

Sbjct 563 MVQGIIQTFSKFGSSRTMPFAKTQFQPFQLYDSLDDLVRGLTNQESQR-MDHFVSEAITN 621

Query 454 HLFESTRGHKHALDLIAVNIQRGRDHGIPAYHYWRQYYRLRRIISLDEF-----GEAGIA 508

HLFE + G LDL+A+NIQRGRDHG+P+Y+ WR+ L++I S E A

Sbjct 622 HLFEGSSGF--GLDLMALNIQRGRDHGLPSYNDWREVCSLKKIGSWQELVGVMDDSAVRI 679

Query 509 MKKAYRDIRDVDLFPGGLLEPSMPGGVVGETFGHILANQFADLKFGDTYFFLHQQAPQGF 568

+ + Y + ++DLF GG+ E G V+G TF I+ +QF+ L+ GD +F+ ++ F

Sbjct 680 LSQLYPSVDEIDLFVGGIGERPQSGAVLGPTFVCIVGDQFSRLRRGDRFFY--EEVSSRF 737

Query 569 RAAQIKAILSVTMSSIICANS-AVTQAQPDPFYMASQLNLPRPCSD--YSEMDVEPW 622

AQ+ I V+++ IIC NS ++ QP F AS LN +PC+ ++D++ W

Sbjct 738 TEAQLDQIRKVSLARIICDNSDDISVIQPLVFQQASFLNQRQPCNTEVIPKLDLKFW 794

>[XP_029714173.1](https://www.ncbi.nlm.nih.gov/protein/XP_029714173.1?report=genbank&log$=protalign&blast_rank=74&RID=0) LOW QUALITY PROTEIN: chorion peroxidase-like [Aedes albopictus]

Length=887

Score = 299 bits (766), Expect = 1e-86, Method: Compositional matrix adjust.

Identities = 217/606 (36%), Positives = 320/606 (53%), Gaps = 83/606 (14%)

Query 56 RYRQIDGRCNHPRN---YGSTGRPVKRYLRPHYQDKFGENLPRVYSVTGQLLPSPRMVSW 112

RYR +G CN+ +N YG P +R L P Y D G + PR SV G+ LPS R VS

Sbjct 256 RYRTNNGTCNNKKNPHTYGVALIPFRRQLTPDYGD--GVSSPR-ESVEGKELPSARQVSL 312

Query 113 KLHPDQTAHDNN-TMLVMQMGQFIDHDITRAPELSGRNA-SIKCCGVPPKERLPDCFPID 170

++H +D N ++++ GQF+DHDIT G +I+CC P + R P+CFP+

Sbjct 313 QIHRPSYHNDPNFSVMLAVWGQFLDHDITSTALNQGVGGKAIECCD-PGQPRHPECFPVP 371

Query 171 IPPGDPVFED----CMEFFRSSPAVDNDGNIIYPREQINALTSFIDGSAVYGSDLDTYTW 226

+ PGDP F D CM F RS PA PR+Q+N T++IDGS VYGSD

Sbjct 372 LGPGDPYFHDYNLTCMNFVRSIPAPTGH---FGPRQQLNQATAYIDGSVVYGSDDAKVKR 428

Query 227 IRSENGTGVFLNTHLV-HGRERLP--SHPHLGPESCVSSNTAESYCQLAGDMRVNEQPGL 283

+R+ G L ++ RE LP + P+ G + N A YC +GD R NE L

Sbjct 429 LRT--GQDGKLRMYVTPDNRELLPISTDPNDGCNE-EAMNAAGKYCFESGDERANENLHL 485

Query 284 GSIHLLFHLHHNHIVRLLVAGILKKRGQPSSPERIAKFIQESSSALKEQIFQEVRKMLGA 343

S+HL++ HHN++ G LKK ER+ FQE R++L A

Sbjct 486 TSMHLIWARHHNNLT-----GELKKVNPDWDDERL---------------FQEARRILAA 525

Query 344 IIQKLTYCDWLPMILGPYLIDKFQLGC---TRRSRYNSDLDPRVANSFLSAALRFGHTLI 400

+Q +TY +++P+I+G ++ +L + R YN +DP VAN F ++A RF HTL+

Sbjct 526 QMQHITYGEFVPVIVGEGTAERMELAPNPESDRDTYNVSVDPSVANVFAASAFRFAHTLL 585

Query 401 PNVYNFGDKRIHLKDTFNIPDASIRYYD---NIIQCLIKEGSEEA-----------YDRY 446

P + K+ H D + P + I + N K G ++A YD+Y

Sbjct 586 PGLM----KKTH--DPTSSP-SGIELHKMLFNPYSLYGKTGLDDAIGGAMTTPLGKYDQY 638

Query 447 VSSAVSEHLFESTRGHKH----ALDLIAVNIQRGRDHGIPAYHYWRQYYRLRRIISLDEF 502

++ ++EHLFE + H LDL+++NIQRGRDHG+P+Y +WR++ RL + + D+

Sbjct 639 FTTELTEHLFEKAQDLLHDRPCGLDLVSLNIQRGRDHGLPSYPHWRRHCRLPPVDTWDQL 698

Query 503 GEAGIA-----MKKAYRDIRDVDLFPGGLLEPSMPGGVVGETFGHILANQFADLKFGDTY 557

+ A M+K Y + +VD++ G L EP + GGVVG +LA+QF LK GD++

Sbjct 699 EKVVDAGSYQQMRKIYGEPENVDVYSGALSEPPVEGGVVGPLITCLLADQFLRLKQGDSF 758

Query 558 FFLHQQAPQGFRAAQIKAILSVTMSSIICANS-AVTQAQPDPFYMASQLNLPR----PCS 612

++ ++ PQ F Q++ I + +SSIIC NS A+TQ+ P Y+ ++N PCS

Sbjct 759 WYERRRGPQRFTRDQLRQIYNTRLSSIICRNSDAITQS---PVYLMRKVNREDNPELPCS 815

Query 613 DYSEMD 618

D

Sbjct 816 QLDTFD 821

>[XP_022701473.1](https://www.ncbi.nlm.nih.gov/protein/XP_022701473.1?report=genbank&log$=protalign&blast_rank=75&RID=0) peroxidase-like isoform X2 [Varroa jacobsoni]

[XP_022701474.1](https://www.ncbi.nlm.nih.gov/protein/XP_022701474.1?report=genbank&log$=protalign&blast_rank=75&RID=0) peroxidase-like isoform X2 [Varroa jacobsoni]

Length=724

Score = 296 bits (757), Expect = 1e-86, Method: Compositional matrix adjust.

Identities = 211/614 (34%), Positives = 311/614 (51%), Gaps = 68/614 (11%)

Query 46 HCSRLTYDQLRYRQIDGRCNHPRN--YGSTGRPVKRYLRPHYQDKFGENLPRVYSVTGQL 103

H S L YR DG CN+ + +G + ++R L P Y D G ++PR+ S TG

Sbjct 142 HHSNLICSDKPYRTADGSCNNLEHAEWGKSFTCLRRLLPPRYAD--GVSMPRI-SETGLQ 198

Query 104 LPSPRMVSWKLHPDQTAHDNN-TMLVMQMGQFIDHDITRAP---------ELSGRNASIK 153

LP+PR+VS +H D + + ++MQ GQF+DHD +P +L N +

Sbjct 199 LPNPRLVSTTIHVDLDRPSRHVSHMLMQWGQFLDHDFALSPIMSHPEEIVDLGNPNDVVD 258

Query 154 CCGVPPKERLPDCFPIDIPPGDPVF----EDCMEFFRSSPAVDNDGNIIYPREQINALTS 209

CC P K P CF DIP D F E CM F RS+ + PR+QI+ALTS

Sbjct 259 CCS-PNKRHDPKCFSFDIPENDKFFSKYGEHCMNFPRSARCPQC---ALGPRQQIDALTS 314

Query 210 FIDGSAVYGSDLDTYTWIRSENGTGVFLNTHLVHGRERLPSHPHLGPESCVSSNTAESYC 269

FIDGS +YGS+ + +R+ G G G LP+ H + C + C

Sbjct 315 FIDGSNIYGSNQEDTYRLRTLAGDGRLKFDVGQRGDMILPASFHPTRDRCSRPEEGD-LC 373

Query 270 QLAGDMRVNEQPGLGSIHLLFHLHHNHIVRLLVAGILKKRGQPSSPERIAKFIQESSSAL 329

AGD RVNEQPGL ++H L+ HHN + +++A+ +S

Sbjct 374 FRAGDERVNEQPGLTAMHTLWLRHHNGLA-----------------DKLARL---NSHWE 413

Query 330 KEQIFQEVRKMLGAIIQKLTYCDWLPMILGPYLIDKFQLGCT--RRSRYNSDLDPRVANS 387

E+IFQE R+++ IQ +TY ++LP+ILG +F L + YN ++DP + N

Sbjct 414 DERIFQEARRIVIGQIQHITYQEFLPLILGDAFYREFGLETLPYGYTTYNKNIDPTILNE 473

Query 388 FLSAALRFGHTLIPNVYNFGD-----KRIHLKDTFNIPDASIRY--YDNIIQCLIKEGSE 440

F A RFGHT++ + D KRI L+D F P R+ + I++ L K+ S+

Sbjct 474 FAGAVFRFGHTILNGHFMEVDTHGNIKRIKLQDNFFKP-FEFRHGKMERIMRGLQKQPSQ 532

Query 441 EAYDRYVSSAVSEHLFESTRGHKHALDLIAVNIQRGRDHGIPAYHYWRQYYRLRRIISLD 500

+D +++ V+ HL+ + LDLIA+NIQRGRDHG+ Y Y + I ++

Sbjct 533 -VFDNFITHDVTNHLYRLS-NESFGLDLIALNIQRGRDHGLRGY---TDYLKGCFGIEVN 587

Query 501 EFGEAGIAMKKAYRD--------IRDVDLFPGGLLEPSMPGGVVGETFGHILANQFADLK 552

F + M + R+ + D+DLF GG+ E +PGGVVG TFG I+ QF LK

Sbjct 588 TFEDLDNVMPRPVRERLESLYAHVNDIDLFTGGVSEYQLPGGVVGPTFGCIMGIQFWRLK 647

Query 553 FGDTYFFLHQQAPQGFRAAQIKAILSVTMSSIICANSAVTQ-AQPDPFYMASQLNLPRPC 611

+GD +++ H F +Q+ I +TM+ I+C NS Q +Q M S+ N PC

Sbjct 648 YGDRFYYEHGGQAGSFTPSQLTQIRKITMAKIVCDNSIGQQFSQQWSLQMVSENNPEIPC 707

Query 612 SDYSEMDVEPWLIH 625

+++MD+ W+ H

Sbjct 708 ESFADMDMSNWIEH 721

>[XP_029733783.1](https://www.ncbi.nlm.nih.gov/protein/XP_029733783.1?report=genbank&log$=protalign&blast_rank=76&RID=0) LOW QUALITY PROTEIN: chorion peroxidase-like [Aedes albopictus]

Length=895

Score = 300 bits (767), Expect = 1e-86, Method: Compositional matrix adjust.

Identities = 217/606 (36%), Positives = 320/606 (53%), Gaps = 83/606 (14%)

Query 56 RYRQIDGRCNHPRN---YGSTGRPVKRYLRPHYQDKFGENLPRVYSVTGQLLPSPRMVSW 112

RYR +G CN+ +N YG P +R L P Y D G + PR SV G+ LPS R VS

Sbjct 264 RYRTNNGTCNNKKNPHTYGVALIPFRRQLTPDYGD--GVSSPR-ESVEGKELPSARQVSL 320

Query 113 KLHPDQTAHDNN-TMLVMQMGQFIDHDITRAPELSGRNA-SIKCCGVPPKERLPDCFPID 170

++H +D N ++++ GQF+DHDIT G +I+CC P + R P+CFP+

Sbjct 321 QIHRPSYHNDPNFSVMLAVWGQFLDHDITSTALNQGVGGKAIECCD-PGQPRHPECFPVP 379

Query 171 IPPGDPVFED----CMEFFRSSPAVDNDGNIIYPREQINALTSFIDGSAVYGSDLDTYTW 226

+ PGDP F D CM F RS PA PR+Q+N T++IDGS VYGSD

Sbjct 380 LGPGDPYFHDYNLTCMNFVRSIPAPTGH---FGPRQQLNQATAYIDGSVVYGSDDAKVKR 436

Query 227 IRSENGTGVFLNTHLV-HGRERLP--SHPHLGPESCVSSNTAESYCQLAGDMRVNEQPGL 283

+R+ G L ++ RE LP + P+ G + N A YC +GD R NE L

Sbjct 437 LRT--GQDGKLRMYVTPDNRELLPISTDPNDGCNE-EAMNAAGKYCFESGDERANENLHL 493

Query 284 GSIHLLFHLHHNHIVRLLVAGILKKRGQPSSPERIAKFIQESSSALKEQIFQEVRKMLGA 343

S+HL++ HHN++ G LKK ER+ FQE R++L A

Sbjct 494 TSMHLIWARHHNNLT-----GELKKVNPDWDDERL---------------FQEARRILAA 533

Query 344 IIQKLTYCDWLPMILGPYLIDKFQLGC---TRRSRYNSDLDPRVANSFLSAALRFGHTLI 400

+Q +TY +++P+I+G ++ +L + R YN +DP VAN F ++A RF HTL+

Sbjct 534 QMQHITYGEFVPVIVGEDTAERMELAPNPESDRDTYNVSVDPSVANVFAASAFRFAHTLL 593

Query 401 PNVYNFGDKRIHLKDTFNIPDASIRYYD---NIIQCLIKEGSEEA-----------YDRY 446

P + K+ H D + P + I + N K G ++A YD+Y

Sbjct 594 PGLM----KKTH--DPTSSP-SGIELHKMLFNPYSLYGKTGLDDAIGGAMTTPLGKYDQY 646

Query 447 VSSAVSEHLFESTRGHKH----ALDLIAVNIQRGRDHGIPAYHYWRQYYRLRRIISLDEF 502

++ ++EHLFE + H LDL+++NIQRGRDHG+P+Y +WR++ RL + + D+

Sbjct 647 FTTELTEHLFEKAQDLLHDRPCGLDLVSLNIQRGRDHGLPSYPHWRRHCRLPPVDTWDQL 706

Query 503 GEAGIA-----MKKAYRDIRDVDLFPGGLLEPSMPGGVVGETFGHILANQFADLKFGDTY 557

+ A M+K Y + +VD++ G L EP + GGVVG +LA+QF LK GD++

Sbjct 707 EKVVDAGSYQQMRKIYGEPENVDVYSGALSEPPVEGGVVGPLITCLLADQFLRLKQGDSF 766

Query 558 FFLHQQAPQGFRAAQIKAILSVTMSSIICANS-AVTQAQPDPFYMASQLNLPR----PCS 612

++ ++ PQ F Q++ I + +SSIIC NS A+TQ+ P Y+ ++N PCS

Sbjct 767 WYERRRGPQRFTRDQLRQIYNTRLSSIICRNSDAITQS---PVYLMRKVNREDNPELPCS 823

Query 613 DYSEMD 618

D

Sbjct 824 QLDTFD 829

>[XP_025194360.1](https://www.ncbi.nlm.nih.gov/protein/XP_025194360.1?report=genbank&log$=protalign&blast_rank=77&RID=0) peroxidase-like [Melanaphis sacchari]

Length=901

Score = 300 bits (767), Expect = 1e-86, Method: Compositional matrix adjust.

Identities = 212/617 (34%), Positives = 318/617 (52%), Gaps = 79/617 (13%)

Query 45 EHCSRLTYDQL-RYRQIDGRCNHPRN--YGSTGRPVKRYLRPHYQDKFGENLPRVYSVTG 101

+ C R ++ Q +YR DG CN+ R+ +G ++R L P Y D G N PR + G

Sbjct 317 DTCPRTSFCQPHKYRSTDGSCNNIRHELWGRASTALQRILPPKYGD--GVNSPRSRAANG 374

Query 102 QLLPSPRMVSWKLHPD-QTAHDNNTMLVMQMGQFIDHDITRAPELSGRNAS-IKCC---- 155

LPS R VS D + +N TML+MQ GQF+DHD T P G+ S I CC

Sbjct 375 SPLPSARQVSVTFTQDVDSPSENYTMLLMQWGQFLDHDTTHTPISRGQMGSGISCCRNGR 434

Query 156 GVPPKERLPDCFPIDIPPGDPVF----EDCMEFFRSSPAVDNDGNIIYPREQINALTSFI 211

+ R PDCF I+IP D +F E CMEF RS PA + N PREQ+N +T+++

Sbjct 435 EIESSLRHPDCFQIEIPRNDHMFAPFGERCMEFVRSLPAPRPECNF-GPREQMNQITAYL 493

Query 212 DGSAVYGSDLDTYTWIRSENGTGVFLNTHLVHGRERLPSHPHLGPESCVSSNTAESYCQL 271

DGS +YGS L T +R+ G L + + G++ LP +P S S +T S C

Sbjct 494 DGSNIYGSSLATQQSLRTFRGG--MLQSQNIRGKQLLPGNP-----SECSDDTGRSACFR 546

Query 272 AGDMRVNEQPGLGSIHLLFHLHHNHIVRLLVAGILKKRGQPSSPERIAKFIQESSSALKE 331

AGD RVNEQ L +H ++ HN I L R P + E

Sbjct 547 AGDGRVNEQIDLALLHTIWLREHNRIAFEL------SRLNPRWSD--------------E 586

Query 332 QIFQEVRKMLGAIIQKLTYCDWLPMILGPYLIDKFQL-----GCTRRSRYNSDLDPRVAN 386

IFQE R+++ A IQ +TY ++LP++LG + KF L G TR Y+ DL+ + N

Sbjct 587 AIFQETRRIVIAQIQHITYNEFLPIVLGRSYMSKFGLSPAESGWTR--NYDPDLNAGITN 644

Query 387 SFLSAALRFGHTLIP-NVYNFGDKRIHLKDTFNIPDASIRYYDNIIQCLIKEG------- 438

+F +AA RFGHTLI N++ +G K ++++ + ++ L KEG

Sbjct 645 AFAAAAYRFGHTLIQGNIHGYG-KFGNIRENLVLSKQHFAPFN-----LYKEGALDDFIR 698

Query 439 -----SEEAYDRYVSSAVSEHLFESTRGHKHALDLIAVNIQRGRDHGIPAYHYWRQ---Y 490

S + +DR+ + +++HLF+ LDL+A+N+QRGRDHG+P Y+ WRQ Y

Sbjct 699 GISFQSSQNFDRFFTREITDHLFQGN--LNFGLDLVALNVQRGRDHGLPPYNEWRQVCGY 756

Query 491 YRLRRIISLDEFGEAG--IAMKKAYRDIRDVDLFPGGLLEPSMPGGVVGETFGHILANQF 548

+ R L+E+ E + + + Y + ++DL+ GG+ E + +VG TF I+ +QF

Sbjct 757 EKARNWNDLEEYMEPQTIVRLARLYNSVDEIDLYIGGVSEKPLKDALVGPTFVCIIGDQF 816

Query 549 ADLKFGDTYFFLHQQAPQGFRAAQIKAILSVTMSSIICANS-AVTQAQPDPFYMASQLNL 607

+ L+ GD +F+ P F Q++ + +++ ++C NS + QP F S LN

Sbjct 817 SRLRRGDRFFYEEGGHPSSFDQVQLQELRKSSLARLLCDNSDDMALIQPLAFLKPSFLNQ 876

Query 608 PRPCSDYS--EMDVEPW 622

C+ S ++D+ W

Sbjct 877 RVACASSSIPKVDLRAW 893

>[XP_019544703.2](https://www.ncbi.nlm.nih.gov/protein/XP_019544703.2?report=genbank&log$=protalign&blast_rank=78&RID=0) chorion peroxidase [Aedes albopictus]

[XP_029733800.1](https://www.ncbi.nlm.nih.gov/protein/XP_029733800.1?report=genbank&log$=protalign&blast_rank=78&RID=0) chorion peroxidase [Aedes albopictus]

Length=842

Score = 298 bits (763), Expect = 1e-86, Method: Compositional matrix adjust.

Identities = 217/606 (36%), Positives = 320/606 (53%), Gaps = 83/606 (14%)

Query 56 RYRQIDGRCNHPRN---YGSTGRPVKRYLRPHYQDKFGENLPRVYSVTGQLLPSPRMVSW 112

RYR +G CN+ +N YG P +R L P Y D G + PR SV G+ LPS R VS

Sbjct 211 RYRTNNGTCNNKKNPHTYGVGLIPFRRQLTPDYGD--GVSSPR-ESVEGKELPSARQVSL 267

Query 113 KLHPDQTAHDNN-TMLVMQMGQFIDHDITRAPELSGRNA-SIKCCGVPPKERLPDCFPID 170

++H +D N ++++ GQF+DHDIT G +I+CC P + R P+CFP+

Sbjct 268 QIHRPSYHNDPNFSVMLAVWGQFLDHDITSTALNQGVGGKAIECCD-PGQPRHPECFPVP 326

Query 171 IPPGDPVFED----CMEFFRSSPAVDNDGNIIYPREQINALTSFIDGSAVYGSDLDTYTW 226

+ PGDP F D CM F RS PA PR+Q+N T++IDGS VYGSD

Sbjct 327 LGPGDPYFHDYNLTCMNFVRSIPAPTGH---FGPRQQLNQATAYIDGSVVYGSDDAKVKR 383

Query 227 IRSENGTGVFLNTHLV-HGRERLP--SHPHLGPESCVSSNTAESYCQLAGDMRVNEQPGL 283

+R+ G L ++ RE LP + P+ G + N A YC +GD R NE L

Sbjct 384 LRT--GQDGKLRMYVTPDNRELLPISTDPNDGCNE-EAMNAAGKYCFESGDERANENLHL 440

Query 284 GSIHLLFHLHHNHIVRLLVAGILKKRGQPSSPERIAKFIQESSSALKEQIFQEVRKMLGA 343

S+HL++ HHN++ G LKK ER+ FQE R++L A

Sbjct 441 TSMHLIWARHHNNLT-----GELKKVNPDWDDERL---------------FQEARRILAA 480

Query 344 IIQKLTYCDWLPMILGPYLIDKFQLGC---TRRSRYNSDLDPRVANSFLSAALRFGHTLI 400

+Q +TY +++P+I+G ++ +L + R YN +DP VAN F ++A RF HTL+

Sbjct 481 QMQHITYGEFVPVIVGEDTAERVELAPNPESDRDTYNVSVDPSVANVFAASAFRFAHTLL 540

Query 401 PNVYNFGDKRIHLKDTFNIPDASIRYYD---NIIQCLIKEGSEEA-----------YDRY 446

P + K+ H D + P + I + N K G ++A YD+Y

Sbjct 541 PGLM----KKTH--DPTSSP-SGIELHKMLFNPYSLYGKTGLDDAIGGAMTTPLGKYDQY 593

Query 447 VSSAVSEHLFESTRGHKH----ALDLIAVNIQRGRDHGIPAYHYWRQYYRLRRIISLDEF 502

++ ++EHLFE + H LDL+++NIQRGRDHG+P+Y +WR++ RL + + D+

Sbjct 594 FTTELTEHLFEKAQDLLHDRPCGLDLVSLNIQRGRDHGLPSYPHWRRHCRLPPVDTWDQL 653

Query 503 GEAGIA-----MKKAYRDIRDVDLFPGGLLEPSMPGGVVGETFGHILANQFADLKFGDTY 557

+ A M+K Y + +VD++ G L EP + GGVVG +LA+QF LK GD++

Sbjct 654 EKVVDAGSYQQMRKIYGEPENVDVYSGALSEPPVEGGVVGPLITCLLADQFLRLKQGDSF 713

Query 558 FFLHQQAPQGFRAAQIKAILSVTMSSIICANS-AVTQAQPDPFYMASQLNLPR----PCS 612

++ ++ PQ F Q++ I + +SSIIC NS A+TQ+ P Y+ ++N PCS

Sbjct 714 WYERRRGPQRFTRDQLRQIYNTRLSSIICRNSDAITQS---PVYLMRKVNREDNPELPCS 770

Query 613 DYSEMD 618

D

Sbjct 771 QLDTFD 776

>[XP_033761418.1](https://www.ncbi.nlm.nih.gov/protein/XP_033761418.1?report=genbank&log$=protalign&blast_rank=79&RID=0) myeloperoxidase-like [Pecten maximus]

Length=904

Score = 299 bits (766), Expect = 1e-86, Method: Compositional matrix adjust.

Identities = 202/597 (34%), Positives = 304/597 (51%), Gaps = 66/597 (11%)

Query 56 RYRQIDGRCN---HPRNYGSTGRPVKRYLRPHYQDKFGENLPRVYSVTGQLLPSPRMVS- 111

R+R IDG CN HPR +G + R +RY +P YQD G + PR SVT LPSPR++S

Sbjct 336 RFRTIDGSCNNLKHPR-WGMSFRAQRRYRQPAYQD--GISTPRKKSVTFNDLPSPRVISN 392

Query 112 --WKLHPDQTAHDNNTMLVMQMGQFIDHDITRAPELSGRN-ASIKCCGVPPKERLPDCFP 168

+K P T + M GQF+DHD P + G A I CC + P+CFP

Sbjct 393 KVFKAGPKSELSAGITSMHMSFGQFLDHDFIFTPVIKGEGGAGINCCASMTTAKRPECFP 452

Query 169 IDIPPGDP-VFEDCMEFFRSSPAVDNDGNIIYPREQINALTSFIDGSAVYGSDLDTYTWI 227

I +P D + E CM F RSS AV++ + R+Q+N +TS++D S VYGS +

Sbjct 453 IIVPQSDKDIKESCMNFVRSSAAVED--RLRGYRDQLNEVTSYLDASNVYGSTKKKMLEL 510

Query 228 RSENGTGVFLNTHLVHGRERLPSHPHLGPESCVSSNTAESYCQLAGDMRVNEQPGLGSIH 287

R L V RLP +G +C +T++ YCQ AGD RVN P LG++H

Sbjct 511 RDSVKKRGLLK---VDSYNRLP----VGKTACFK-DTSDDYCQDAGDKRVNVVPNLGAVH 562

Query 288 LLFHLHHNHIVRLLVAGILKKRGQPSSPERIAKFIQESSSALKEQIFQEVRKMLGAIIQK 347

LL+ HN +V++L + + ++F E RK++ A++Q

Sbjct 563 LLWVREHNRVVKILA--------------------NHNPTWDDNRLFYEGRKIIIAMMQH 602

Query 348 LTYCDWLPMILGP-YLID-KFQLGCTRRSRYNSD-LDPRVANSFLSAALRFGHTLIPNVY 404

+ Y ++LP+IL YL + K QL S Y D +D V+N F +AA RFGH+ I N+

Sbjct 603 IVYNEYLPIILNSTYLYNLKLQLRPLGYSYYYRDNIDATVSNVFGAAAFRFGHSQITNIQ 662

Query 405 ------NFGDKRIHLKDTFNIP----DASIRYYDNIIQCLIKEGSEEAYDRYVSSAVSEH 454

+ K + ++ TF+ P + Y+ +++ L + ++ DR+ +

Sbjct 663 AQYSPSHVPTKTVPIEKTFHRPHLCEEMKGNGYEGVLRWLSNAKATKS-DRFFEVGIRNK 721

Query 455 LFESTRGHKHALDLIAVNIQRGRDHGIPAYHYWRQYYRLRRIISLD---EFG------EA 505

LF +G+ +DL A+N+ RGRDHGIPAY+ WR++ L + + FG EA

Sbjct 722 LFPDPKGN--TMDLPAINVNRGRDHGIPAYNVWREWCGLPKAKTFQAGVSFGLLHHTKEA 779

Query 506 GIAMKKAYRDIRDVDLFPGGLLEPSMPGGVVGETFGHILANQFADLKFGDTYFFLHQQAP 565

A++ AY D+DL+ G + E ++ G+VG TF I+ QF LK GD +++ +

Sbjct 780 AAALQSAYSHPEDIDLYAGAMSETAIKNGIVGPTFACIIGYQFQQLKLGDRFWYEYNLGR 839

Query 566 QGFRAAQIKAILSVTMSSIICANSAVTQAQPDPFYMASQLNLPRPCSDYSEMDVEPW 622

+G AQ+ I +++ IIC N VT+ QP F S N CS ++++ W

Sbjct 840 RGLNPAQLSEIRKSSLAKIICNNIQVTKMQPKAFQTVSTTNARIDCSKIPDVNLAFW 896

>[XP_034310503.1](https://www.ncbi.nlm.nih.gov/protein/XP_034310503.1?report=genbank&log$=protalign&blast_rank=80&RID=0) peroxidasin-like isoform X1 [Crassostrea gigas]

Length=695

Score = 295 bits (754), Expect = 1e-86, Method: Compositional matrix adjust.

Identities = 209/596 (35%), Positives = 299/596 (50%), Gaps = 72/596 (12%)

Query 57 YRQIDGRCNHPR--NYGSTGRPVKRYLRPHYQDKFGENLPRVYSVTGQLLPSPRMVSWKL 114

YR DGRCN+ GS G RYL P Y+D G PR S TG+ L SPR++S ++

Sbjct 140 YRSADGRCNNLLYPTLGSRGATQSRYLPPKYED--GITQPRNLSGTGKPLLSPRLISNEM 197

Query 115 ----HPDQ-TAHDNN-TMLVMQMGQFIDHDITRAPELSGRNAS-IKCCGVPPKERLPDCF 167

HP + T D +++ GQF+ HD+ P + + +KCC DC

Sbjct 198 FQGPHPGKPTPKDRKLSLMTFTWGQFMIHDVVLTPVSTNEDGEELKCCDEDADHL--DCL 255

Query 168 PIDIPPGDPVF---EDCMEFFRSSPAVDNDGNIIYPREQINALTSFIDGSAVYGSDLDTY 224

PIDIPP DP F ++CM F RS PA DG REQ+N LTSFID VYG L ++

Sbjct 256 PIDIPPDDPHFRGRQNCMSFSRSIPAESYDGCAPGHREQVNRLTSFIDAGNVYGDSLLSH 315

Query 225 TWIRSENGTGVFLNTHLVHGRERLPSHPHLGPES--CVSSNTAESYCQLAGDMRVNEQPG 282

+ + +G +LNT + +L P C + + +C +AGD RV+E P

Sbjct 316 ALLNAPHG---YLNT----------TKYNLLPPGGVCKTLSNDRDHCPIAGDERVSENPA 362

Query 283 LGSIHLLFHLHHNHIVRLLVAGILKKRGQPSSPERIAKFIQESSSALKEQIFQEVRKMLG 342

LG +H+L+ HNHIVR L ++ S +IFQE RK+LG

Sbjct 363 LGGMHVLWVRLHNHIVRQLA---------------------KNKSWSSYKIFQETRKILG 401

Query 343 AIIQKLTYCDWLPMILGPYLIDKFQLGCTR---RSRYNSDLDPRVANSFLSAALRFGHTL 399

A++Q +TY +LP IL P I + L +RY+ ++DP V N +AA RFGH++

Sbjct 402 AMMQLITYKHYLPKILSPRTIARRNLSLKEHGYETRYDQEIDPSVKNVVAAAAFRFGHSM 461

Query 400 IPNV--YNFGD---KRIHLKDTFNIPDASIR----YYDNIIQCLIKEGSEEAYDRYVSSA 450

IP Y F D + L+D P + Y ++I+ +++ S DR + SA

Sbjct 462 IPPELGYLFHDMTRRTFKLEDILLDPHLVVTQRGVYLPDLIRFVLRNSSG-LVDREIESA 520

Query 451 VSEHLFESTRGHKHALDLIAVNIQRGRDHGIPAYHYWRQYYRLRRIISLDEFGEAGI--- 507

V +LF +G + DL A NIQRGRDHG+P Y WR++ L + + + I

Sbjct 521 VRNNLFVDNQGL--SFDLGAFNIQRGRDHGLPPYADWRKHCGLSVPTTFEHLEDHDIHTR 578

Query 508 -AMKKAYRDIRDVDLFPGGLLEPSMPGGVVGETFGHILANQFADLKFGDTYFFLHQQAPQ 566

+ Y + D+D+F GGL E G V+G F ++ QF DLKFGD Y+F

Sbjct 579 EKLSNVYELVTDIDVFVGGLSELPEEGSVLGPVFNCLIGKQFRDLKFGDRYWF-ENPGVG 637

Query 567 GFRAAQIKAILSVTMSSIICANSAVTQAQPDPFYMASQLNLPRPCSDYSEMDVEPW 622

GF Q+K I SVT++S+IC+ + + Q D F + + N C +++ E W

Sbjct 638 GFTPDQLKVIRSVTLASVICSVLDIGEIQRDAFEIQHKGNPLVSCKKIPKLNFEAW 693

>[XP_029733779.1](https://www.ncbi.nlm.nih.gov/protein/XP_029733779.1?report=genbank&log$=protalign&blast_rank=81&RID=0) chorion peroxidase-like [Aedes albopictus]

Length=895

Score = 299 bits (766), Expect = 1e-86, Method: Compositional matrix adjust.

Identities = 217/606 (36%), Positives = 320/606 (53%), Gaps = 83/606 (14%)

Query 56 RYRQIDGRCNHPRN---YGSTGRPVKRYLRPHYQDKFGENLPRVYSVTGQLLPSPRMVSW 112

RYR +G CN+ +N YG P +R L P Y D G + PR SV G+ LPS R VS

Sbjct 264 RYRTNNGTCNNKKNPHTYGVALIPFRRQLTPDYGD--GVSSPR-ESVEGKELPSARQVSL 320

Query 113 KLHPDQTAHDNN-TMLVMQMGQFIDHDITRAPELSGRNA-SIKCCGVPPKERLPDCFPID 170

++H +D N ++++ GQF+DHDIT G +I+CC P + R P+CFP+

Sbjct 321 QIHRPSYHNDPNFSVMLAVWGQFLDHDITSTALNQGVGGKAIECCD-PGQPRHPECFPVP 379

Query 171 IPPGDPVFED----CMEFFRSSPAVDNDGNIIYPREQINALTSFIDGSAVYGSDLDTYTW 226

+ PGDP F D CM F RS PA PR+Q+N T++IDGS VYGSD

Sbjct 380 LGPGDPYFHDYNLTCMNFVRSIPAPTGH---FGPRQQLNQATAYIDGSVVYGSDDAKVKR 436

Query 227 IRSENGTGVFLNTHLV-HGRERLP--SHPHLGPESCVSSNTAESYCQLAGDMRVNEQPGL 283

+R+ G L ++ RE LP + P+ G + N A YC +GD R NE L

Sbjct 437 LRT--GQDGKLRMYVTPDNRELLPISTDPNDGCNE-EAMNAAGKYCFESGDERANENLHL 493

Query 284 GSIHLLFHLHHNHIVRLLVAGILKKRGQPSSPERIAKFIQESSSALKEQIFQEVRKMLGA 343

S+HL++ HHN++ G LKK ER+ FQE R++L A

Sbjct 494 TSMHLIWARHHNNLT-----GELKKVNPDWDDERL---------------FQEARRILAA 533

Query 344 IIQKLTYCDWLPMILGPYLIDKFQLGC---TRRSRYNSDLDPRVANSFLSAALRFGHTLI 400

+Q +TY +++P+I+G ++ +L + R YN +DP VAN F ++A RF HTL+

Sbjct 534 QMQHITYGEFVPVIVGEDTAERMELAPNPESDRDTYNVSVDPSVANVFAASAFRFAHTLL 593

Query 401 PNVYNFGDKRIHLKDTFNIPDASIRYYD---NIIQCLIKEGSEEA-----------YDRY 446

P + K+ H D + P + I + N K G ++A YD+Y

Sbjct 594 PGLM----KKTH--DPTSSP-SGIELHKMLFNPYSLYGKTGLDDAIGGAMTTPLGKYDQY 646

Query 447 VSSAVSEHLFESTRGHKH----ALDLIAVNIQRGRDHGIPAYHYWRQYYRLRRIISLDEF 502

++ ++EHLFE + H LDL+++NIQRGRDHG+P+Y +WR++ RL + + D+

Sbjct 647 FTTELTEHLFEKAQDLLHDRPCGLDLVSLNIQRGRDHGLPSYPHWRRHCRLPPVDTWDQL 706

Query 503 GEAGIA-----MKKAYRDIRDVDLFPGGLLEPSMPGGVVGETFGHILANQFADLKFGDTY 557

+ A M+K Y + +VD++ G L EP + GGVVG +LA+QF LK GD++

Sbjct 707 EKVVDAGSYQQMRKIYGEPENVDVYSGALSEPPVEGGVVGPLITCLLADQFLRLKQGDSF 766

Query 558 FFLHQQAPQGFRAAQIKAILSVTMSSIICANS-AVTQAQPDPFYMASQLNLPR----PCS 612

++ ++ PQ F Q++ I + +SSIIC NS A+TQ+ P Y+ ++N PCS

Sbjct 767 WYERRRGPQRFTRDQLRQIYNTRLSSIICRNSDAITQS---PVYLMRKVNREDNPELPCS 823

Query 613 DYSEMD 618

D

Sbjct 824 QLDTFD 829

>[XP_021351643.1](https://www.ncbi.nlm.nih.gov/protein/XP_021351643.1?report=genbank&log$=protalign&blast_rank=82&RID=0) chorion peroxidase-like [Mizuhopecten yessoensis]

[XP_021351644.1](https://www.ncbi.nlm.nih.gov/protein/XP_021351644.1?report=genbank&log$=protalign&blast_rank=82&RID=0) chorion peroxidase-like [Mizuhopecten yessoensis]

[OWF51425.1](https://www.ncbi.nlm.nih.gov/protein/OWF51425.1?report=genbank&log$=protalign&blast_rank=82&RID=0) Chorion peroxidase [Mizuhopecten yessoensis]

Length=833

Score = 298 bits (762), Expect = 2e-86, Method: Compositional matrix adjust.

Identities = 201/604 (33%), Positives = 305/604 (50%), Gaps = 80/604 (13%)

Query 57 YRQIDGRCN---HPRNYGSTGRPVKRYLRPHYQDKFGENLPRVYSVTGQLLPSPRMVSWK 113

YR IDG CN HP +G TG P KR+L P Y D F N PR G LP+PR +S +

Sbjct 158 YRTIDGSCNNIGHPA-WGMTGMPQKRFLAPDYDDGF--NSPRSRGQDGAPLPNPRNISNE 214

Query 114 LHP---DQTAHDNNTMLVMQMGQFIDHDITRAPELSGRNA-SIKCCGVPPKERLPDCFPI 169

LH +QT ++++M GQFI+HDI P + + S+ CCG+ R DC P+

Sbjct 215 LHQSNGNQTYESKLSVMLMTWGQFINHDIVGTPLIKDSHGRSLDCCGLDKTNR--DCLPV 272

Query 170 DIPPGDPVFED-CMEFFRSSPAVDNDGNIIYPREQINALTSFIDGSAVYGSDLDTYTWIR 228

DIP DP ++ CM F R++PA + +I R IN +TS++DGSA+YGS + + +R

Sbjct 273 DIPMDDPYYDSSCMNFVRTAPAAGPECSI-GERRPINKVTSYLDGSAIYGSTKEEESRLR 331

Query 229 SENGTGVFLNTHLVHGRERLPSH---PHLGPESCVSSNTAESYCQLAGDMRVNEQPGLGS 285

+ HG R H P G ++C+ + CQLAGD R N +G+

Sbjct 332 -----------QMEHGLLREGHHGLLPPTGNDNCILQKKGQQ-CQLAGDERSNLVASIGA 379

Query 286 IHLLFHLHHNHIVRLLVAGILKKRGQPSSPERIAKFIQESSSALK-EQIFQEVRKMLGAI 344

+H LF HN RIA + E + + + E+++QE RK +GAI

Sbjct 380 LHTLFMREHN---------------------RIALHLHEINPSWEDERLYQETRKTVGAI 418

Query 345 IQKLTYCDWLPMILGPYLIDKFQLGCTR---RSRYNSDLDPRVANSFLSAALRFGHTLIP 401

IQ +TY ++LP+I+G LI+ + L T Y ++ V++ F +AA RFGH+ IP

Sbjct 419 IQHITYHEYLPLIIGKPLINFYNLDDTEFGHVQAYKDYVNAGVSSGFAAAAFRFGHSQIP 478

Query 402 NVYNF------GDKRIHLKDTFNIPDASIRY----YDNIIQCLIKEGSEEAYDRYVSSAV 451

+ K + L+ TF+ P R Y+ I++ LI + + DRY+ V

Sbjct 479 KKQTYIGPKFEKKKGVVLEKTFHKPYYLQRSKHFGYEGILRWLISDPTP-TVDRYLEDGV 537

Query 452 SEHLFESTRGHKHALDLIAVNIQRGRDHGIPAYHYWRQYYRLRRIISL---------DEF 502

+ LF +LDL ++NIQRGR+ G+P+Y+ WR++ L + + D

Sbjct 538 RDRLF---LLQNISLDLASINIQRGREQGVPSYNAWRKWCGLSEVTTFEVKRNGGLEDHD 594

Query 503 GEAGIAMKKAYRDIRDVDLFPGGLLEPSMPGGVVGETFGHILANQFADLKFGDTYFFLHQ 562

E +++ YR D+DL+ GG+ E + GG VG TF I+A QF+ +K GD Y++ +

Sbjct 595 EETAAVLQELYRSPNDIDLYSGGVSEVHVRGGSVGPTFACIIARQFSAMKVGDRYWYENN 654

Query 563 QAPQGFRAAQIKAILSVTMSSIICANSAVTQAQPDPFYMASQLNLPR---PCSDYSEMDV 619

GF Q+ +I ++ +S +IC N + Q F + PCS ++

Sbjct 655 DTTTGFTTGQLDSIKTIELSKLICENMDIPDIQRTAFLTPKISGVHTRHIPCSFLPDLKF 714

Query 620 EPWL 623

+ WL

Sbjct 715 KAWL 718

>[XP_022650762.1](https://www.ncbi.nlm.nih.gov/protein/XP_022650762.1?report=genbank&log$=protalign&blast_rank=83&RID=0) peroxidase-like isoform X3 [Varroa destructor]

[XP_022701468.1](https://www.ncbi.nlm.nih.gov/protein/XP_022701468.1?report=genbank&log$=protalign&blast_rank=83&RID=0) peroxidase-like isoform X1 [Varroa jacobsoni]

[XP_022701469.1](https://www.ncbi.nlm.nih.gov/protein/XP_022701469.1?report=genbank&log$=protalign&blast_rank=83&RID=0) peroxidase-like isoform X1 [Varroa jacobsoni]

[XP_022701470.1](https://www.ncbi.nlm.nih.gov/protein/XP_022701470.1?report=genbank&log$=protalign&blast_rank=83&RID=0) peroxidase-like isoform X1 [Varroa jacobsoni]

[XP_022701471.1](https://www.ncbi.nlm.nih.gov/protein/XP_022701471.1?report=genbank&log$=protalign&blast_rank=83&RID=0) peroxidase-like isoform X1 [Varroa jacobsoni]

[XP_022701472.1](https://www.ncbi.nlm.nih.gov/protein/XP_022701472.1?report=genbank&log$=protalign&blast_rank=83&RID=0) peroxidase-like isoform X1 [Varroa jacobsoni]

Length=732

Score = 295 bits (756), Expect = 2e-86, Method: Compositional matrix adjust.

Identities = 211/614 (34%), Positives = 311/614 (51%), Gaps = 68/614 (11%)

Query 46 HCSRLTYDQLRYRQIDGRCNHPRN--YGSTGRPVKRYLRPHYQDKFGENLPRVYSVTGQL 103

H S L YR DG CN+ + +G + ++R L P Y D G ++PR+ S TG

Sbjct 150 HHSNLICSDKPYRTADGSCNNLEHAEWGKSFTCLRRLLPPRYAD--GVSMPRI-SETGLQ 206

Query 104 LPSPRMVSWKLHPDQTAHDNN-TMLVMQMGQFIDHDITRAP---------ELSGRNASIK 153

LP+PR+VS +H D + + ++MQ GQF+DHD +P +L N +

Sbjct 207 LPNPRLVSTTIHVDLDRPSRHVSHMLMQWGQFLDHDFALSPIMSHPEEIVDLGNPNDVVD 266

Query 154 CCGVPPKERLPDCFPIDIPPGDPVF----EDCMEFFRSSPAVDNDGNIIYPREQINALTS 209

CC P K P CF DIP D F E CM F RS+ + PR+QI+ALTS

Sbjct 267 CCS-PNKRHDPKCFSFDIPENDKFFSKYGEHCMNFPRSARCPQC---ALGPRQQIDALTS 322

Query 210 FIDGSAVYGSDLDTYTWIRSENGTGVFLNTHLVHGRERLPSHPHLGPESCVSSNTAESYC 269

FIDGS +YGS+ + +R+ G G G LP+ H + C + C

Sbjct 323 FIDGSNIYGSNQEDTYRLRTLAGDGRLKFDVGQRGDMILPASFHPTRDRCSRPEEGD-LC 381

Query 270 QLAGDMRVNEQPGLGSIHLLFHLHHNHIVRLLVAGILKKRGQPSSPERIAKFIQESSSAL 329

AGD RVNEQPGL ++H L+ HHN + +++A+ +S

Sbjct 382 FRAGDERVNEQPGLTAMHTLWLRHHNGLA-----------------DKLARL---NSHWE 421

Query 330 KEQIFQEVRKMLGAIIQKLTYCDWLPMILGPYLIDKFQLGCT--RRSRYNSDLDPRVANS 387

E+IFQE R+++ IQ +TY ++LP+ILG +F L + YN ++DP + N

Sbjct 422 DERIFQEARRIVIGQIQHITYQEFLPLILGDAFYREFGLETLPYGYTTYNKNIDPTILNE 481

Query 388 FLSAALRFGHTLIPNVYNFGD-----KRIHLKDTFNIPDASIRY--YDNIIQCLIKEGSE 440

F A RFGHT++ + D KRI L+D F P R+ + I++ L K+ S+

Sbjct 482 FAGAVFRFGHTILNGHFMEVDTHGNIKRIKLQDNFFKP-FEFRHGKMERIMRGLQKQPSQ 540

Query 441 EAYDRYVSSAVSEHLFESTRGHKHALDLIAVNIQRGRDHGIPAYHYWRQYYRLRRIISLD 500

+D +++ V+ HL+ + LDLIA+NIQRGRDHG+ Y Y + I ++

Sbjct 541 -VFDNFITHDVTNHLYRLS-NESFGLDLIALNIQRGRDHGLRGY---TDYLKGCFGIEVN 595

Query 501 EFGEAGIAMKKAYRD--------IRDVDLFPGGLLEPSMPGGVVGETFGHILANQFADLK 552

F + M + R+ + D+DLF GG+ E +PGGVVG TFG I+ QF LK

Sbjct 596 TFEDLDNVMPRPVRERLESLYAHVNDIDLFTGGVSEYQLPGGVVGPTFGCIMGIQFWRLK 655

Query 553 FGDTYFFLHQQAPQGFRAAQIKAILSVTMSSIICANSAVTQ-AQPDPFYMASQLNLPRPC 611

+GD +++ H F +Q+ I +TM+ I+C NS Q +Q M S+ N PC

Sbjct 656 YGDRFYYEHGGQAGSFTPSQLTQIRKITMAKIVCDNSIGQQFSQQWSLQMVSENNPEIPC 715

Query 612 SDYSEMDVEPWLIH 625

+++MD+ W+ H

Sbjct 716 ESFADMDMSNWIEH 729

>[XP_021375133.1](https://www.ncbi.nlm.nih.gov/protein/XP_021375133.1?report=genbank&log$=protalign&blast_rank=84&RID=0) lactoperoxidase-like [Mizuhopecten yessoensis]

[OWF40024.1](https://www.ncbi.nlm.nih.gov/protein/OWF40024.1?report=genbank&log$=protalign&blast_rank=84&RID=0) Peroxidasin [Mizuhopecten yessoensis]

Length=689

Score = 294 bits (752), Expect = 2e-86, Method: Compositional matrix adjust.

Identities = 198/591 (34%), Positives = 294/591 (50%), Gaps = 62/591 (10%)

Query 56 RYRQIDGRCNHPRN--YGSTGRPVKRYLRPHYQDKFGENLPRVYSVTGQLLPSPRMVSWK 113

++R DG CN+ +GS +R+L P Y D G + PR GQ LPSPR VS

Sbjct 135 KFRSADGSCNNLVQPWFGSAATAQERFLPPLYDD--GVSSPRTLGKKGQPLPSPRKVSNS 192

Query 114 LHPDQTAHDNN--TMLVMQMGQFIDHDITRAPELSGRNASIKCCGVPPKERLPDCFPIDI 171

+ +++VM GQF+DHDIT P SG S C LP+CFPI I

Sbjct 193 VFKSSIEKKETELSLMVMAWGQFLDHDITLTPASSGSETSATDCCRNVSTALPECFPIAI 252

Query 172 PPGDPVFE--DCMEFFRSSPAVDNDGNIIYP--REQINALTSFIDGSAVYGSDLDTYTWI 227

P D F CM+F RS+ D P REQ NA+T+F+DGS VYGS + +

Sbjct 253 PSDDLHFTRTKCMDFVRSAAVTD----ACSPNHREQFNAITAFVDGSNVYGSSVAQMNEL 308

Query 228 RS-ENGTGVFLNTHLVHGRERLPSHPHLGPESCVSSNTAESYCQLAGDMRVNEQPGLGSI 286

R+ NG L T V LP P P SC+ N+ +C AGD+R N P LG+

Sbjct 309 RAFRNG---LLATSTVTSS--LP--PAGDPHSCLI-NSNPDFCIKAGDVRANVIPHLGAN 360

Query 287 HLLFHLHHNHIVRLLVAGILKKRGQPSSPERIAKFIQESSSALKEQIFQEVRKMLGAIIQ 346

H+L HN I +L A ++ E++FQE RK++ I+Q

Sbjct 361 HVLLFREHNRIATILSA--------------------MNNGWNDERVFQETRKIISGILQ 400

Query 347 KLTYCDWLPMILGPYLIDKFQLGCTRRSRYNSDLDPRVANSFLSAALRFGHTLIP----- 401

+++Y +WLP IL P+ + KF L + + Y S +P + N F AA+RFGH+LIP

Sbjct 401 QISYYEWLPSILSPFHLSKFDLLSSNQDPYRSRTNPSIRNGFAVAAMRFGHSLIPANECY 460

Query 402 --NVYNFGDKRIHLKDTFNIPDASIRY----YDNIIQCLIKEGSEEAYDRYVSSAVSEHL 455

Y + ++ TF P I+ + + +I S +A DR V L

Sbjct 461 LLRDYVTWEVEKPIQQTFFSPSLVIQNAGQDVPKLARWVIANNSMKA-DRIFEPGVRNLL 519

Query 456 FESTRGHKHALDLIAVNIQRGRDHGIPAYHYWRQYYRLRRIISLDEFGE----AGIAMKK 511

F + G + DL ++NIQRGRDHG+P Y +R+ + L++ S ++ + A +K

Sbjct 520 FLDSNG--SSFDLGSLNIQRGRDHGVPPYVEYRKLFGLKKPKSFNKLTDHNSYAKGLLKD 577

Query 512 AYRDIRDVDLFPGGLLEPSMPGGVVGETFGHILANQFADLKFGDTYFFLHQQAPQGFRAA 571

Y ++D+DLF GG+ E +P G +G F ++ QF D+K GD ++F + +P+GF A

Sbjct 578 VYDSVKDIDLFAGGMSERKVPNGHLGPVFTELIGRQFRDIKLGDRFWF-ERPSPEGFPPA 636

Query 572 QIKAILSVTMSSIICANSAVTQAQPDPFYMASQLNLPRPCSDYSEMDVEPW 622

Q I +T++ ++C N + D F++ S +N C+ ++D+ W

Sbjct 637 QRDEIRKMTLAKVMCTNFGMDLVTKDVFHIQSTINPLTTCNSIPDIDLTLW 687

>[XP_015373617.1](https://www.ncbi.nlm.nih.gov/protein/XP_015373617.1?report=genbank&log$=protalign&blast_rank=85&RID=0) PREDICTED: peroxidase-like [Diuraphis noxia]

Length=902

Score = 298 bits (764), Expect = 3e-86, Method: Compositional matrix adjust.

Identities = 216/614 (35%), Positives = 321/614 (52%), Gaps = 73/614 (12%)

Query 45 EHCSRLTYDQL-RYRQIDGRCNHPRN--YGSTGRPVKRYLRPHYQDKFGENLPRVYSVTG 101

+ C R ++ Q +YR DG CN+ ++ +G ++R L P Y D G N PR +V G

Sbjct 318 DTCPRTSFCQPHKYRSTDGSCNNIKHELWGRASTALQRILPPKYGD--GVNSPRSRAVNG 375

Query 102 QLLPSPRMVSWKLHPD-QTAHDNNTMLVMQMGQFIDHDITRAPELSGRNAS-IKCC---- 155

LPS R VS D + +N TML+MQ GQF+DHD T P G+ S I CC

Sbjct 376 SPLPSARQVSVTFTQDVDSPSENYTMLLMQWGQFLDHDTTHTPISRGQMGSGISCCRNGR 435

Query 156 GVPPKERLPDCFPIDIPPGDPVF----EDCMEFFRSSPAVDNDGNIIYPREQINALTSFI 211

+ R PDCF I+IP D +F E CMEF RS PA + N PREQ+N +T+++

Sbjct 436 EIENSLRHPDCFQIEIPRNDHMFAPFGERCMEFVRSLPAPRPECNF-GPREQMNQITAYL 494

Query 212 DGSAVYGSDLDTYTWIRSENGTGVFLNTHLVHGRERLPSHPHLGPESCVSSNTAESYCQL 271

DGS +YGS L T +R+ G L + + GR+ LP +P S S +T S C

Sbjct 495 DGSNIYGSSLATQQSLRTFRGGT--LQSQNIRGRQLLPGNP-----SECSDDTGRSACFK 547

Query 272 AGDMRVNEQPGLGSIHLLFHLHHNHIVRLLVAGILKKRGQPSSPERIAKFIQESSSALKE 331

AGD RVNEQ L +H ++ HN I L R P + E

Sbjct 548 AGDGRVNEQIDLALLHTIWLREHNRIAFEL------SRLNPRWSD--------------E 587

Query 332 QIFQEVRKMLGAIIQKLTYCDWLPMILGPYLIDKFQL-----GCTRRSRYNSDLDPRVAN 386

IFQE R+++ A +Q +TY ++LP+ILG + KF L G TR Y+ +L+ + N

Sbjct 588 AIFQETRRIVIAQLQHITYNEFLPIILGRSYMTKFGLSPAESGWTR--NYDPELNAGITN 645

Query 387 SFLSAALRFGHTLIP-NVY---NFGDKRIHLKDT------FNIPDASIRYYDNIIQCLIK 436

+F +AA RFGHTLI N++ FG+ R +L + FN+ +D+ I+ +

Sbjct 646 AFAAAAYRFGHTLIQGNIHGYGKFGNIRENLVLSKQHFAPFNLYKEGA--FDDFIRGISF 703

Query 437 EGSEEAYDRYVSSAVSEHLFESTRGHKHALDLIAVNIQRGRDHGIPAYHYWRQ---YYRL 493

+ S+ +DR+ + +++HLF+ LDL+A+N+QRGRDHG+P Y+ WRQ Y +

Sbjct 704 QSSQN-FDRFFTKEITDHLFQGN--LNFGLDLVALNVQRGRDHGLPPYNEWRQVCGYEKA 760

Query 494 RRIISLDEFGEAGIAMKKA--YRDIRDVDLFPGGLLEPSMPGGVVGETFGHILANQFADL 551

R L+E+ + + A Y + ++DL+ GG+ E M +VG TF I+ +QF+ L

Sbjct 761 RSWNDLEEYMDPQTITRLARLYGSVDEIDLYIGGVSEKPMKDALVGPTFVCIIGDQFSRL 820

Query 552 KFGDTYFFLHQQAPQGFRAAQIKAILSVTMSSIICANS-AVTQAQPDPFYMASQLNLPRP 610

+ GD +F+ P F Q++ + +++ ++C NS + QP F S LN

Sbjct 821 RRGDRFFYEEGGHPSSFDQVQLQELRKSSLARLLCDNSDDMALIQPLAFSKPSFLNQRVA 880

Query 611 CSDYS--EMDVEPW 622

C+ S +MD+ W

Sbjct 881 CASSSIPKMDLRAW 894

>[XP_023335820.1](https://www.ncbi.nlm.nih.gov/protein/XP_023335820.1?report=genbank&log$=protalign&blast_rank=86&RID=0) chorion peroxidase-like [Eurytemora affinis]

Length=906

Score = 298 bits (764), Expect = 3e-86, Method: Compositional matrix adjust.

Identities = 197/596 (33%), Positives = 307/596 (52%), Gaps = 58/596 (10%)

Query 57 YRQIDGRCN--HPRNYGSTGRPVKRYLRPHYQDKFGENLPRVYSVTGQLLPSPRMVSWKL 114

+R DG CN H +G++ P R+L P Y D E R SV+ LP+PR++S +

Sbjct 193 FRTPDGTCNNFHHPTWGASFTPFLRFLPPDYSDGI-EAFRR--SVSNGPLPNPRIISSMI 249

Query 115 HPDQTAHDNN-TMLVMQMGQFIDHDITRAPELSGRNASI-KCCGVPPKERLPDCFPIDIP 172

H D + N TM+VMQ GQF+DHDIT P G N SI KCC P+ PDC PI IP

Sbjct 250 HRDVSKDTNQFTMMVMQWGQFLDHDITSTPVTRGFNESILKCCSQSPETMHPDCQPIMIP 309

Query 173 PGDPVFE----DCMEFFRSSPAVDNDGNIIYPREQINALTSFIDGSAVYGSDL-DTYTWI 227

D + CMEF RSSP D + + PR+QIN +TS+ID S VYGS D ++

Sbjct 310 ADDSFYSKFNVSCMEFVRSSPGPRRDCS-LGPRDQINQITSYIDASNVYGSTPDDQHSLR 368

Query 228 RSENGTGVFLNTHLVHGRERLPSHPHLGPESCVSSNTAESYCQLAGDMRVNEQPGLGSIH 287

+ G + + H+ + LP L E + +C AGD RVNEQPGL ++H

Sbjct 369 LLKKGKLKYTDLHI--RKPLLPPLESLEAEEACRIKSPNLHCFHAGDERVNEQPGLATMH 426

Query 288 LLFHLHHNHIVRLLVAGILKKRGQPSSPERIAKFIQESSSALKEQIFQEVRKMLGAIIQK 347

L+ HN + + G L +++F E R+ +GA++Q

Sbjct 427 TLWLREHNRVA--VEMGSLNPHWS------------------DDRVFLETRRFIGAVVQH 466

Query 348 LTYCDWLPMILGPYLIDKFQLGCTRRSR---YNSDLDPRVANSFLSAALRFGHTLIPNVY 404

+TY +WLP+ILGP +++ F+L R YN+ ++P VAN+F +AA RFGH+L+ N

Sbjct 467 ITYNEWLPIILGPRVLEIFELRLLPRGYYRGYNASVNPTVANAFGAAAFRFGHSLVKNTI 526

Query 405 NFGDKR-------IHLKDTFNIPDASIRYYDNIIQCLIKEGSEEAY--DRYVSSAVSEHL 455

+ +K + L N P +++ + ++ + L+ ++ D +++ ++ L

Sbjct 527 SRCNKEFRTVPFHVDLHKEMNNP-SNLHNFGSVDRILLSLCDDKMARRDEFITEELTNRL 585

Query 456 FESTRGHKHALDLIAVNIQRGRDHGIPAYHYWRQYYRLRRI---ISLDEFGEAGIA--MK 510

F++ + +DL+++NIQRGRDHG+ Y+ WR+ L+R I ++ E ++

Sbjct 586 FQTPKS-GFGMDLMSLNIQRGRDHGLAPYNIWREQCGLKRFTAWIQMETVMEKTTVNRLE 644

Query 511 KAYRDIRDVDLFPGGLLEPSMPGGVVGETFGHILANQFADLKFGDTYFFLHQQAPQGFRA 570

Y + D+DLF GG+ E + GG+VG TF IL QF +L+ GD +++ + P F

Sbjct 645 NVYEHVDDIDLFTGGMAEKPVVGGIVGPTFSCILGQQFLNLRKGDRFWYENGDHPGAFTP 704

Query 571 AQIKAILSVTMSSIIC-ANSAVTQAQPDPFYMASQLNLPRPCSD---YSEMDVEPW 622

+Q++ I +++ +IC + QP F Q R M+++PW

Sbjct 705 SQLQEIRKTSLARVICDCLDDIDMLQPFAFLQPDQFANQRTVCKGQGIQRMNLDPW 760

>[VEN39256.1](https://www.ncbi.nlm.nih.gov/protein/VEN39256.1?report=genbank&log$=protalign&blast_rank=87&RID=0) unnamed protein product [Callosobruchus maculatus]

Length=813

Score = 296 bits (758), Expect = 4e-86, Method: Compositional matrix adjust.

Identities = 209/602 (35%), Positives = 314/602 (52%), Gaps = 69/602 (11%)

Query 57 YRQIDGRCN---HPRNYGSTGRPVKRYLRPHYQDKFGENLPRVYSVTGQLLPSPRMVSWK 113

YR DG CN HP +G P + L P Y+D+ E R +SV GQLLPS R VS +

Sbjct 227 YRTPDGSCNNQQHP-TWGQALTPNTKLLLPAYEDRVFE--ARKFSVNGQLLPSARTVSSR 283

Query 114 LHPDQTAHDNN-TMLVMQMGQFIDHDITRAPELSGRNAS-IKCC---GVPPKERLPD--C 166

L ++ + N T+L+ Q GQFI HD++++ + S RN+S I CC G ERL C

Sbjct 284 LMKNKDVFNGNYTLLLAQFGQFIGHDVSQSVDHSFRNSSGISCCTDSGDHWPERLQHFAC 343

Query 167 FPIDIPPGDPVF-----EDCMEFFRSSPAVDNDGNIIYPREQINALTSFIDGSAVYGSDL 221

PID+PP D + CM F RS A D+D + Y + Q++ +T F+D S +YGS +

Sbjct 344 LPIDVPPNDGFYGRHFGRRCMHFMRSVFAPDHDCRLGYAK-QLDKVTHFVDASGIYGSSI 402

Query 222 DTYTWIRS-ENGTGVFLNTHLVHGRERLPSHPHLGPESCVSSNTAESYCQLAGDMRVNEQ 280

+ + +R+ ENG L GR+ LP H GP C+S + S C +GD RVN

Sbjct 403 EQQSDLRTFENGK---LKVFHDFGRKLLPLHEDSGP--CIS-DRGGSACFKSGDTRVNSL 456

Query 281 PGLGSIHLLFHLHHNHIVRLLVAGILKKRGQPSSPERIAKFIQESSSALKEQIFQEVRKM 340

L ++H +FH HN + + L I+ + E +FQE RK+

Sbjct 457 ITLTALHTVFHREHNRLAQAL--------------------IEINPHWTDETVFQEARKI 496

Query 341 LGAIIQKLTYCDWLPMILGPYLID----KFQLGCTRRSRYNSDLDPRVANSFLSAALRFG 396

L A +Q + Y ++LP+++G +D + Q G + Y+ +++P VA+ F + A RFG

Sbjct 497 LIAELQTVLYREFLPLVIGLEAMDLYGLRLQEGPVYATDYDPEIEPSVASEFTAGAFRFG 556

Query 397 HTLIPNVYNFG-----DKRI-HLKDTFNIPDA--SIRYYDNIIQCLIKEGSEEAYDRYVS 448

H+LI + D+ + + +T N P I +D I+ LI + ++ D +

Sbjct 557 HSLIETTISLSKGGRVDQVLAFVPETMNYPSQMRRIDIFDMILVALITQPVQQV-DENFN 615

Query 449 SAVSEHLFESTRGHKHALDLIAVNIQRGRDHGIPAYHYWRQYYRLRRIISLDEFG-EAGI 507

+ +++F G+ +DLI++NIQRGRDHGI Y+ +R+ L++ S +FG +

Sbjct 616 ENLQKYVFRF--GNCFGVDLISINIQRGRDHGIQPYNRYRELLGLQKFTSFADFGPKYAK 673

Query 508 AMKKAYRDIRDVDLFPGGLLEPSMPGGVVGETFGHILANQFADLKFGDTYFFLHQQA--P 565

+ Y + DVDL+ GGLLE PG +VG F HI+A+QFA LK GD YFF + + P

Sbjct 674 TLASIYSSVDDVDLYVGGLLEEKAPGAIVGRVFQHIIADQFARLKKGDRYFFENHPSINP 733

Query 566 QGFRAAQIKAILSVTMSSIICANS---AVTQAQPDPFYMASQLNLPRPCSD--YSEMDVE 620

F AQ+ I +M+ IIC N+ A+ QP+ F + S N P C +D+

Sbjct 734 AYFEPAQLSEIRKTSMARIICDNADRLALGMVQPEVFRVPSPWNQPVDCRSNIIPSIDLS 793

Query 621 PW 622

W

Sbjct 794 KW 795

>[CAC5395001.1](https://www.ncbi.nlm.nih.gov/protein/CAC5395001.1?report=genbank&log$=protalign&blast_rank=88&RID=0) PXDN [Mytilus coruscus]

Length=779

Score = 295 bits (756), Expect = 4e-86, Method: Compositional matrix adjust.

Identities = 202/605 (33%), Positives = 298/605 (49%), Gaps = 83/605 (14%)

Query 57 YRQIDGRCN--HPRNYGSTGRPVKRYLRPHYQDKFGENLPRVYSVTGQLLPSPRMVSWKL 114

YR DG CN H +G R R+L Y D G N+PR++S+TG LPSPR++S +

Sbjct 179 YRTADGGCNNFHVPTWGQAMRAQLRWLPAAYSD--GLNVPRIHSITGASLPSPRLISNVV 236

Query 115 HPDQTA---HDNNTMLVMQMGQFIDHDITRAPELSG-RNASIKCCGVPP--KERLPDCFP 168

H + +ML+MQ GQF+DHD P G N+ I+CC + P +++ CFP

Sbjct 237 HNAHMSDFREPKQSMLMMQFGQFLDHDFVGTPTNRGFNNSEIQCCDLDPTTRDKRGSCFP 296

Query 169 IDIPPGDPVFED-CMEFFRSSPAVDNDGNIIY----PREQINALTSFIDGSAVYGSDLDT 223

I +PP D F + CM F RSS A + +Y PR QIN +SFIDGS +YG+ +

Sbjct 297 IPVPPKDKRFHNPCMNFVRSSIAPE-----LYCKPAPRNQINQQSSFIDGSMIYGNSMKE 351

Query 224 YTWIRSENG------TGVFLNTHLVHGRERLPSHPHLGPESCVSSNTAESYCQLAGDMRV 277

T++R G G FL P C+ + E YC AGD R

Sbjct 352 QTYLRLGKGGLLKMTDGGFL--------------PRYEDGDCIMESPGE-YCFGAGDDRN 396

Query 278 NEQPGLGSIHLLFHLHHNHIVRLLVAGILKKRGQPSSPERIAKFIQESSSALKEQIFQEV 337

P L +H++F HN RL VA + K + E IFQE

Sbjct 397 TIVPSLAFLHIVFLREHN---RLAVA--------------LGKI---NPHWTDEIIFQET 436

Query 338 RKMLGAIIQKLTYCDWLPMILGPYLIDKFQLGCTR---RSRYNSDLDPRVANSFLSAALR 394

RK++G ++Q + Y +WLP++L + + L + YN LDP AN +AA R

Sbjct 437 RKIIGGVLQHIMYSEWLPLVLNDEFMTIYGLKSVYGKYNTVYNEKLDPTTANVMAAAAFR 496

Query 395 FGHTLIPNVYNFGD---KRIH---LKDTFNIPDASIRYY---DNIIQCLIKEGSEEAYDR 445

FGH++IPN +G K+I + T+N P + + ++ I + + S DR

Sbjct 497 FGHSMIPNFLGYGGPGGKKIAEFPIYKTYNRPGVLMSHGGKGNDWIGEWMLDDSHAKSDR 556

Query 446 YVSSAVSEHLFESTRGHKHALDLIAVNIQRGRDHGIPAYHYWRQYYRLRRIIS------- 498

V+ V +LF H + DL++ NIQRGRDHG+P Y+ WR++ L +I

Sbjct 557 VVNDGVRNYLFMDH--HGMSFDLVSFNIQRGRDHGLPPYNAWRKWCGLPPVIHFGHGPGG 614

Query 499 -LDEFGEAGIAMKKAYRDIRDVDLFPGGLLEPSMPGGVVGETFGHILANQFADLKFGDTY 557

+D +A I + K Y+ + D+DL+ GGL E + GG+ G TF ++ FA+ KFGD +

Sbjct 615 LVDHDKDAIIRLSKTYKHVDDIDLYTGGLSERRVKGGLTGPTFQCLIGKSFANWKFGDRF 674

Query 558 FFLHQQAPQGFRAAQIKAILSVTMSSIICANSAVTQAQPDPFYMASQLNLPRPCSDYSEM 617

++ + GF Q++ I +S IIC N+ + Q F + S+ N PC ++

Sbjct 675 WYENDFKLTGFSPRQLETIRHHKISKIICQNTKIDHIQQASFLLPSKHNQKIPCHAIPDI 734

Query 618 DVEPW 622

D+ W

Sbjct 735 DLLAW 739

>[XP_012253358.1](https://www.ncbi.nlm.nih.gov/protein/XP_012253358.1?report=genbank&log$=protalign&blast_rank=89&RID=0) uncharacterized protein LOC105684530 isoform X2 [Athalia rosae]

Length=1444

Score = 305 bits (780), Expect = 4e-86, Method: Compositional matrix adjust.

Identities = 208/605 (34%), Positives = 321/605 (53%), Gaps = 71/605 (12%)

Query 55 LRYRQIDGRCNHPRN--YGSTGRPVKRYLRPHYQDKFGENLPRVYSVTGQLLPSPRMVSW 112

LRYR DG CN+P +GS P++R+L+P YQD + + R SV G+LLPSPR +S

Sbjct 204 LRYRTTDGSCNNPNELWWGSAMSPMQRFLQPTYQDGI-QTIRR--SVNGRLLPSPREISS 260

Query 113 KLHPDQTAH-DNNTMLVMQMGQFIDHDITRAPELSGRNASI-KCCG------VPPKERLP 164

+H D+ + T ++MQ GQF+DHD+T + G N ++ +CC PP+ P

Sbjct 261 FIHQDRDIPLASVTHMLMQWGQFVDHDLTATGQSRGFNGTVPQCCANGGAGFQPPEFMHP 320

Query 165 DCFPIDIPPGD----PVFEDCMEFFRSSPAVDNDGNIIYPREQINALTSFIDGSAVYGSD 220

+C PI +P D P+ C+EF RS A D R+Q++ +TS++D S VY S+

Sbjct 321 ECLPIPVPTNDRFYGPLGVRCLEFVRSGAAPREDCGFGA-RDQLSQVTSYLDASTVYSSN 379

Query 221 LDTYTWIRSENGTGVFLNTHLVHGR--ERLPSHPHLGPESCVSSNTAESYCQLAGDMRVN 278

+ T +R +F N L +GR R P P + C + A S C AGD R++

Sbjct 380 VQTSDSLR------LFRNGLLQYGRIQSRKPLLPRQESDLCRRGSLATS-CFRAGDGRLS 432

Query 279 EQPGLGSIHLLFHLHHNHIVRLLVAGILKKRGQPSSPERIAKFIQESSSALKEQIFQEVR 338

EQP L S+H++F HN I L A + E++FQE R

Sbjct 433 EQPALISMHVIFLRQHNRIATELAA--------------------LNPHWSDEKLFQETR 472

Query 339 KMLGAIIQKLTYCDWLPMILGPYLIDKFQLGCTRR---SRYNSDLDPRVANSFLSAALRF 395

+++GA IQ +TY ++LP++LGP ++ F L TR+ Y+ ++P VANSF +AA RF

Sbjct 473 RIIGAFIQLITYREFLPIVLGPDVMKAFDLEVTRKGYYEGYDPTVNPNVANSFAAAAYRF 532

Query 396 GHTLIPNVYNFGD-------KRIHLKDTFNIPDA--SIRYYDNIIQCLIKEGSEEAYDRY 446

GH+L+ + + D + + D FN P + D ++ L+ + S++ D +

Sbjct 533 GHSLVQHSFVRYDTDHQPIFNNVSIHDEFNNPVNLHTPGSVDRLLLGLVNQPSQKR-DEF 591

Query 447 VSSAVSEHLFESTRGHKHALDLIAVNIQRGRDHGIPAYHYWRQYYRLRRIISLDEF---- 502

++S ++ HLFE T G +DL ++NIQRGRDHG+P Y WR+ L I D+

Sbjct 592 ITSELTNHLFE-TPGFP-GMDLASLNIQRGRDHGLPPYVDWREPCGLSPIKGWDDLDRVM 649

Query 503 -GEAGIAMKKAYRDIRDVDLFPGGLLEPSMPGGVVGETFGHILANQFADLKFGDTYFFLH 561

+ + Y ++D+DLFPGGL E +PGG+VG TF I+A QF++++ GD ++F +

Sbjct 650 PSASARRFRVIYTSVQDIDLFPGGLAEKPVPGGLVGPTFACIIAQQFSNIRKGDRFWFEN 709

Query 562 QQAPQGFRAAQIKAILSVTMSSIICANS-AVTQAQPDPFYMASQLNLPR-PCSD--YSEM 617

F AQ++ I VT++ I+C + ++ QP F R C++ +

Sbjct 710 PNGESSFTPAQLQQIKRVTLAQILCKTADSIETIQPFVFLTPDTFRNQRIECANEIIGSL 769

Query 618 DVEPW 622

D+ PW

Sbjct 770 DLAPW 774

>[XP_012253356.1](https://www.ncbi.nlm.nih.gov/protein/XP_012253356.1?report=genbank&log$=protalign&blast_rank=90&RID=0) uncharacterized protein LOC105684530 isoform X1 [Athalia rosae]

Length=1600

Score = 305 bits (781), Expect = 5e-86, Method: Compositional matrix adjust.

Identities = 208/605 (34%), Positives = 321/605 (53%), Gaps = 71/605 (12%)

Query 55 LRYRQIDGRCNHPRN--YGSTGRPVKRYLRPHYQDKFGENLPRVYSVTGQLLPSPRMVSW 112

LRYR DG CN+P +GS P++R+L+P YQD + + R SV G+LLPSPR +S

Sbjct 360 LRYRTTDGSCNNPNELWWGSAMSPMQRFLQPTYQDGI-QTIRR--SVNGRLLPSPREISS 416

Query 113 KLHPDQTAH-DNNTMLVMQMGQFIDHDITRAPELSGRNASI-KCCG------VPPKERLP 164

+H D+ + T ++MQ GQF+DHD+T + G N ++ +CC PP+ P

Sbjct 417 FIHQDRDIPLASVTHMLMQWGQFVDHDLTATGQSRGFNGTVPQCCANGGAGFQPPEFMHP 476

Query 165 DCFPIDIPPGD----PVFEDCMEFFRSSPAVDNDGNIIYPREQINALTSFIDGSAVYGSD 220

+C PI +P D P+ C+EF RS A D R+Q++ +TS++D S VY S+

Sbjct 477 ECLPIPVPTNDRFYGPLGVRCLEFVRSGAAPREDCGFGA-RDQLSQVTSYLDASTVYSSN 535

Query 221 LDTYTWIRSENGTGVFLNTHLVHGR--ERLPSHPHLGPESCVSSNTAESYCQLAGDMRVN 278

+ T +R +F N L +GR R P P + C + A S C AGD R++

Sbjct 536 VQTSDSLR------LFRNGLLQYGRIQSRKPLLPRQESDLCRRGSLATS-CFRAGDGRLS 588

Query 279 EQPGLGSIHLLFHLHHNHIVRLLVAGILKKRGQPSSPERIAKFIQESSSALKEQIFQEVR 338

EQP L S+H++F HN I L A + E++FQE R

Sbjct 589 EQPALISMHVIFLRQHNRIATELAA--------------------LNPHWSDEKLFQETR 628

Query 339 KMLGAIIQKLTYCDWLPMILGPYLIDKFQLGCTRR---SRYNSDLDPRVANSFLSAALRF 395

+++GA IQ +TY ++LP++LGP ++ F L TR+ Y+ ++P VANSF +AA RF

Sbjct 629 RIIGAFIQLITYREFLPIVLGPDVMKAFDLEVTRKGYYEGYDPTVNPNVANSFAAAAYRF 688

Query 396 GHTLIPNVYNFGD-------KRIHLKDTFNIPDA--SIRYYDNIIQCLIKEGSEEAYDRY 446

GH+L+ + + D + + D FN P + D ++ L+ + S++ D +

Sbjct 689 GHSLVQHSFVRYDTDHQPIFNNVSIHDEFNNPVNLHTPGSVDRLLLGLVNQPSQKR-DEF 747

Query 447 VSSAVSEHLFESTRGHKHALDLIAVNIQRGRDHGIPAYHYWRQYYRLRRIISLDEF---- 502

++S ++ HLFE T G +DL ++NIQRGRDHG+P Y WR+ L I D+
[truncated: 391,178 more chars]
