# Supplementary material for: Mantle Modularity Underlies the Plasticity of the Molluscan Shell: Supporting Data From Cepaea nemoralis
Source: Front Genet. 2021 Feb 5;12:622400. doi: 10.3389/fgene.2021.622400 (PMC7894901; doi:10.3389/fgene.2021.622400)
Supplement: Supplementary file 13 [file Data_Sheet_13.pdf]

```
Cnem_R27072837     1 MMNLL-----------------------TLVLVGLFSTALSQGAGGLAPVPQNYKPDLKPMLPPPMPMKPSSPFLPP--- 80
Lsta_sfc_27a         MKWLISFLFFGVVTSAPQLNNQPLPPAMRLVLADMLKNVL------GNPGPHGFKLDLKPPLPSLVPQKTLAPFFAPVPN
Lsta_sfc_27b         MKWLISFLFFGVVTSAPQLNNQPLPP-----------------------------------------------VFAPVPN

Cnem_R27072837    81 --------PMQMAPPGPPQPRTVNDGLFMDLPHAGGAHYGGGAPYGGGPQYGGGPQYGGGPYMGPPVMRSYHYCPPGPTT 160
Lsta_sfc_27a         FPPQPLPPFQPQPPPGPPGGGVAGNGLFMDLPNSGPGGY------------------------AAPMMRSYHYCPPGPTT
Lsta_sfc_27b         FPPQPLPPFQPQPPPGPPGGGVAGNGLFMDLPNSGPGGY------------------------AAPMMRSYHYCPPGPTT

Cnem_R27072837   161 ADHCKDQKLQEALYFPDGTPRYNWVPPKNPWDTSLPDTVKETAKNILMMKVNSRPSRIPTPKEWELMSLLGDPKEQGPAA 240
Lsta_sfc_27a         SDHCKDQKLMEALYHPDGTPRYNWVPPRNPWDNSLPDTVKDTAMHILMMKVNSRPNRTPTPKEWELIGLLGDPKEQGHAG
Lsta_sfc_27b         SDHCKDQKL-EALYHPDGTPRYNWVPPRNPWDNSLPDTVKDTAMHILMMKVNSRPNRTPTPKEWELIGLLGDPKEQGHAG

Cnem_R27072837   241 NNPFAGR------- 254
Lsta_sfc_27a         HGAFGMGGFPAAPG
Lsta_sfc_27b         HGAFGMGGFPAAPG
```
