# Supplementary material for: Mantle Modularity Underlies the Plasticity of the Molluscan Shell: Supporting Data From Cepaea nemoralis
Source: Front Genet. 2021 Feb 5;12:622400. doi: 10.3389/fgene.2021.622400 (PMC7894901; doi:10.3389/fgene.2021.622400)
Supplement: Supplementary file 14 [file Data_Sheet_14.pdf]

```
Cnem_R27075188     1 -------MATAGLIVAVSSL----AWFIAGVASQATDASSHCSYLINSVSR-----YQPNAAAFKIYTRSRSPRVTSGEP 80
Lsta_sfc_20a         --------MLTSLVVLQVCLQIAFVPIAVV-AQAPADQTSHCSYLVNSKSQ-----FQENAAPFKLYTRSRQPRITSGDP
Lsta_sfc_20b         MRMSSQLLAEKHIPSTQACLLHLVTLLAISRLILCQTVNSHCEYLVGAAPSGAPVPGLQLGSPFKIYTKAKVTEVTPGHP

Cnem_R27075188    81 IEVTIGPFSSSLNFFNFTDFILYATPSNIANLEVEFIGPTSPHVGVFQLFDKWRAGAGGLNCNPRSRAEDSVGAFEDRLL 160
Lsta_sfc_20a         VEVTIGPFSQHLNFFNFTDFILYATPSNTANLEIEIFGGPNPHVGVFQLFDQWRAGAGGLNCNPRSRSEDSVGAFEERLL
Lsta_sfc_20b         IQVTIGPFSQHLNFFNFTDFILYATPSNTANLEIEIFGGPNPHVGVFQLFDQWRAGAGGLNCNPRSRSEDSVGAFEERLL

Cnem_R27075188   161 ATFKQYYPNNPMLLRYHAPARNQVSVLWWPTKEALMYPEIKFVANIKSMGNWFKLQSTPWKVNRPVDQWANTESMLAQYQ 240
Lsta_sfc_20a         SIFKRYYPNNPMLMRYQPPARNQVSVLWWPTKEALAYPEIKFIANLKSMNNWFRIESTTWKVNRPVDQWANTERILTQYQ
Lsta_sfc_20b         SIFKRYYPNNPMLMRYQPPARNQVSVLWWPTKEALAYPEIKFIANLKSMNNWFRIESTTWKVNRPVDQWANTERILTQYQ

Cnem_R27075188   241 AMQNNMRAMERRLEQPI 257
Lsta_sfc_20a         AMQSNMRAMERRLDQPV
Lsta_sfc_20b         AMQSNMRAMERRLDQPV
```
